# Supplementary material for: Pillar[5]arene-catalyzed anti-Markovnikov halogenations through cationic intermediates stabilization in confined spaces
Source: Nat Commun. 2026 Apr 1;17:4668. doi: 10.1038/s41467-026-71201-9 (PMC13201766; doi:10.1038/s41467-026-71201-9)
Supplement: Supplementary file 1 — Supplementary information [file 41467_2026_71201_MOESM1_ESM.pdf]

Supplementary Information

Pillar[5]arene-Catalyzed Anti-Markovnikov Halogenations Through Cationic Intermediates Stabilization in Confined Spaces

Tianyue Xu[1,†], Shengtian Lai[1,†], Manjaly J. Ajitha[2,*], Kuo-Wei Huang[2,*], Ying-Yeung Yeung[1,*]

[1]State Key Laboratory of Synthetic Chemistry, The Chinese University of Hong Kong, Shatin, NT, Hong Kong (China).

[2]Center of Excellence for Renewable Energy and Storage Technologies, KAUST Catalysis Platform, and Division of Physical Sciences and Engineering, King Abdullah University of Science and Technology, Thuwal 23955-6900, Saudi Arabia.

[†]These authors contributed equally.

*Corresponding authors. Email: ajitha.john@kaust.edu.sa; kuowei.huang@kaust.edu.sa; yyyeung@cuhk.edu.hk

Contents

Materials and Methods 2
Supplementary Methods 3
1. Benchmarking studies 3
1.1 Intramolecular size-selectivity 3
1.2 Intermolecular size-selectivity 4
1.3 Size-selectivity of nucleophile 5
1.4 Reaction screening results 6
2. Mechanistic studies 8
2.1 Determination of the binding ratio by Job's plot 8
2.2 Determination of the binding constant by titration experiments 9
2.3 Monitoring the reaction progress 10
2.4 ^{1}H – ^{1}H ROESY NMR 11
2.5 IR Study 12
3. Structural determination 13
4. Computational studies 16
5. Catalyst preparation 32
6. Substrate preparation 38
7. Anti-Markovnikov intermolecular bromoesterification 46
8. Derivatization of the anti-Markovnikov products 57
9. Preparation of tosylate **14** 60
10. NMR spectra 61
11. References 126

Materials and Methods

All reactions that required anhydrous conditions were carried out with standard procedures under nitrogen atmosphere. Commercially available reagents were used as received unless specified. Halogen sources were purified prior to use. The solvents were dried by distillation over the appropriate drying reagents or solvent purification system (Inert PS-MD-7). ^{1}H NMR and ^{13}C NMR spectra were recorded on a Bruker ADVANCE III (400 MHz) or Brucker AMX500 (500 MHz) spectrometer. Chemical shifts (δ) are reported in ppm using residual solvent protons (^{1}H NMR, δH = 7.26 for $CDCl_3$, ^{13}C NMR, δC = 77.16 for $CDCl_3$) as internal standard unless specified. High-resolution mass spectra were obtained on a Therno Finnigan MAT 95 XL spectrometer. X-ray crystallography data were collected on a Bruker AXS Kappa ApexII Duo Diffractometer. Infra-Red spectra were measured by ReactIR 700. Analytical thin layer chromatography (TLC) was performed with Merck pre-coated TLC plates, silica gel 60F-254, layer thickness 0.25 mm. Flash chromatography separations were performed on Merck 60 (0.040-0.063 mm) mesh silica gel.

Supplementary Methods

1. Benchmarking studies

   1.1 Intramolecular size-selectivity

Experiments using bis-olefinic substrate **10a** were conducted to evaluate the intramolecular size-selectivity. With pillar[5]arene catalyst **PA5**, electrophilic halogenation took place preferentially at the less hindered olefinic moiety in an anti-Markovnikov fashion to give **11a** and no Markovnikov product **11a’** was detected. In the benchmarking study using triphenylphosphine sulfide that is a typical Lewis base catalyst, a mixture which consists of multiple halogenated products was obtained.

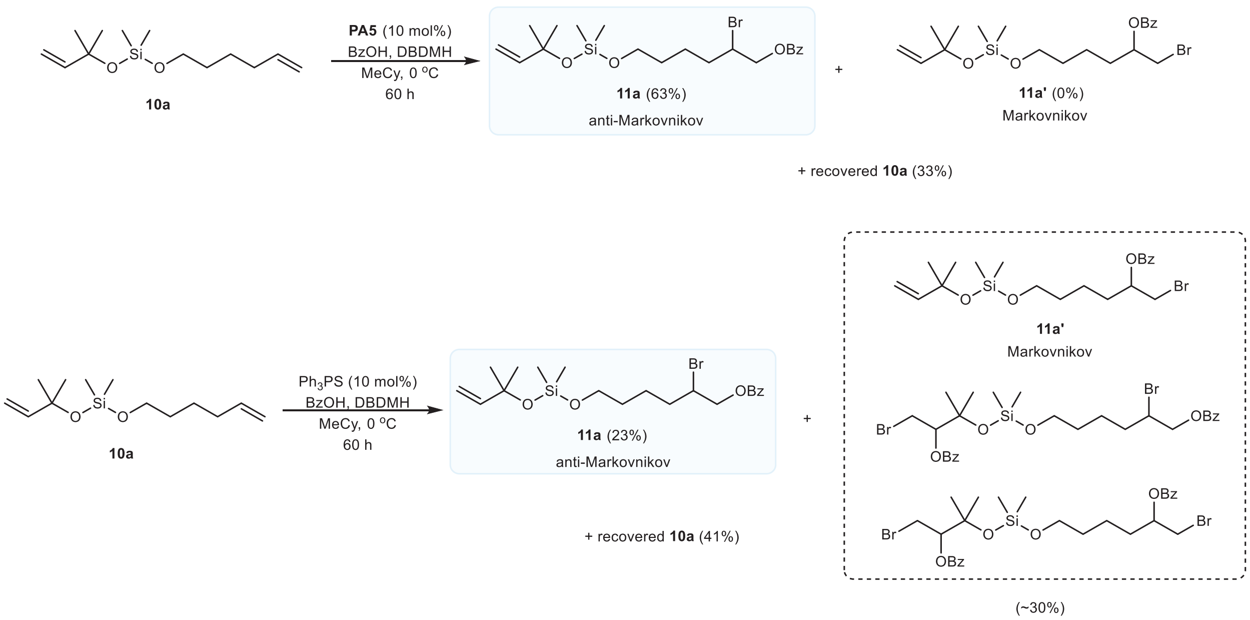

Fig. S1. Benchmarking study of intramolecular size-selectivity.

1.2 Intermolecular size-selectivity

Intermolecular competition experiments of olefinic substrates **1a** and **12a** were conducted to evaluate the size-selectivity. With pillar[5]arene catalyst **PA5**, electrophilic halogenation took place preferentially at the linear olefin **1a** in an anti-Markovnikov fashion to give **3aa** solely and no Markovnikov product **3aa’** was detected. In addition, no halogenation associated with branched olefin **12a** was observed. In the benchmarking study using triphenylphosphine sulfide as the catalyst, a mixture that consists of **3aa’** and **24a’** as the major Markovnikov products was obtained.

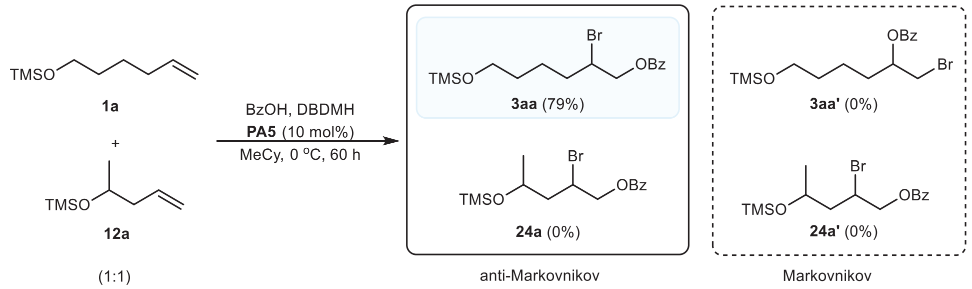

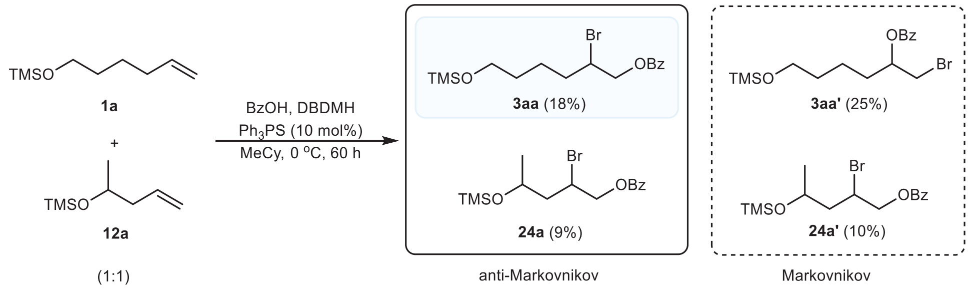

Fig. S2. Benchmarking study of intermolecular size-selectivity.

1.3 Size-selectivity of nucleophile

Intermolecular competition experiments using benzoic acids **2a** and **2h** were conducted to evaluate the size-selectivity of nucleophile. With pillar[5]arene catalyst **PA5**, electrophilic halogenation with the smaller size nucleophile **2a** took place preferentially in an anti-Markovnikov fashion to give **3aa** solely and no Markovnikov product **3aa'** was detected. In addition, no halogenation associated with the bulkier nucleophile **2h** was observed. In the benchmarking study using triphenylphosphine sulfide as the catalyst, both **2a** and **2h** readily reacted to give a product mixture with **3aa'** and **3ah'** as the Markovnikov major products.

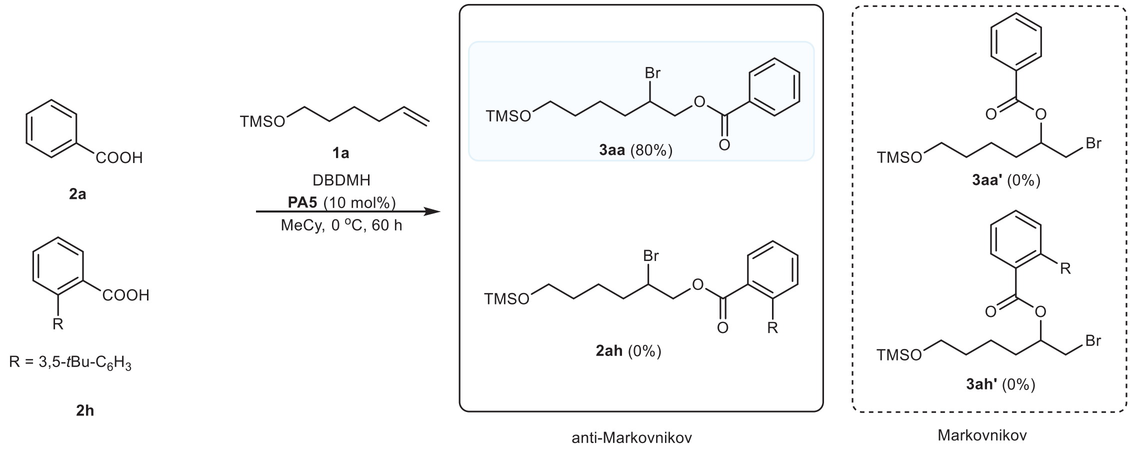

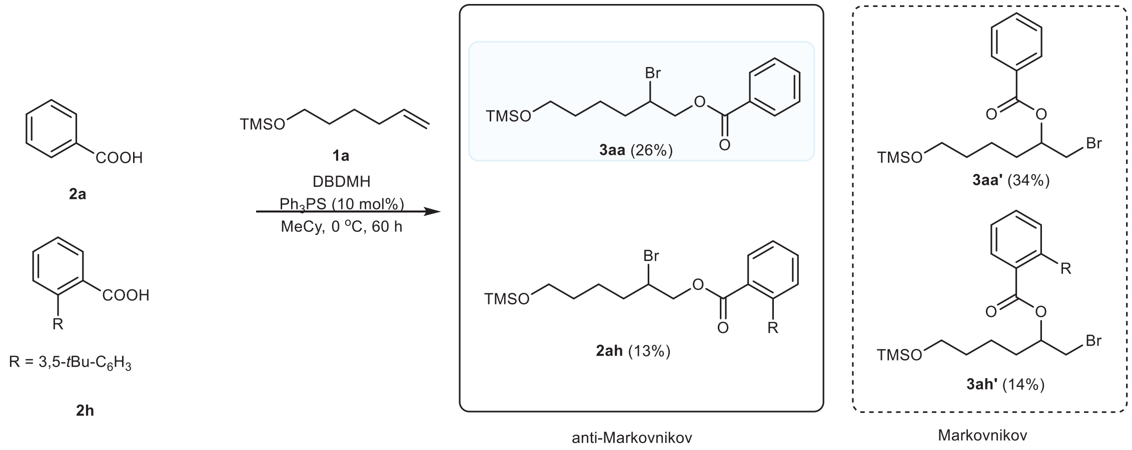

Fig. S3. Benchmarking study of size-selectivity of different nucleophiles.

1.4 Reaction screening results

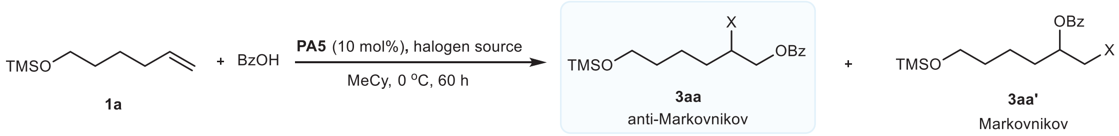

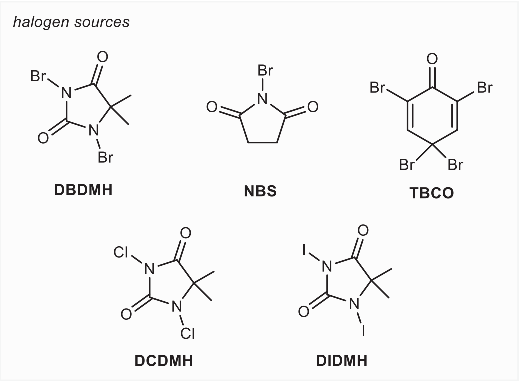

entry	halogen source	X	yield	3aa:3aa'
1	DBDMH	Br	82%	>99:1
2	NBS	Br	0%	NR
3	TBCO	Br	0%	NR
4	DCDMH	Cl	0%	NR
5	DIDMH	I	10%	1:1

Fig. S4. Study on different halogen sources.

*Note: we have examined different halogen sources, and DBDMH was found to be optimal. It appears that the size of chlorine and iodine did not match the size of **PA5**, leading to inefficient reaction.*

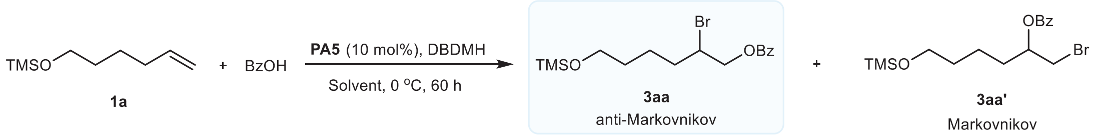

entry	solvent	yield	3aa:3aa'
1	PhMe	99%	0.8:1
2	m-xylene	99%	0.8:1
3	heptane	37%	2.7:1
4	2-methylbutane	44%	14:1
5	MeCy	82%	>20:1

Fig. S5. Study on different solvents.

*Note: the reaction was found to be solvent-dependent, and a more polar solvent gave a high yield but low regioselectivity, attributed to the background reaction happening outside the cavity of **PA5**.*

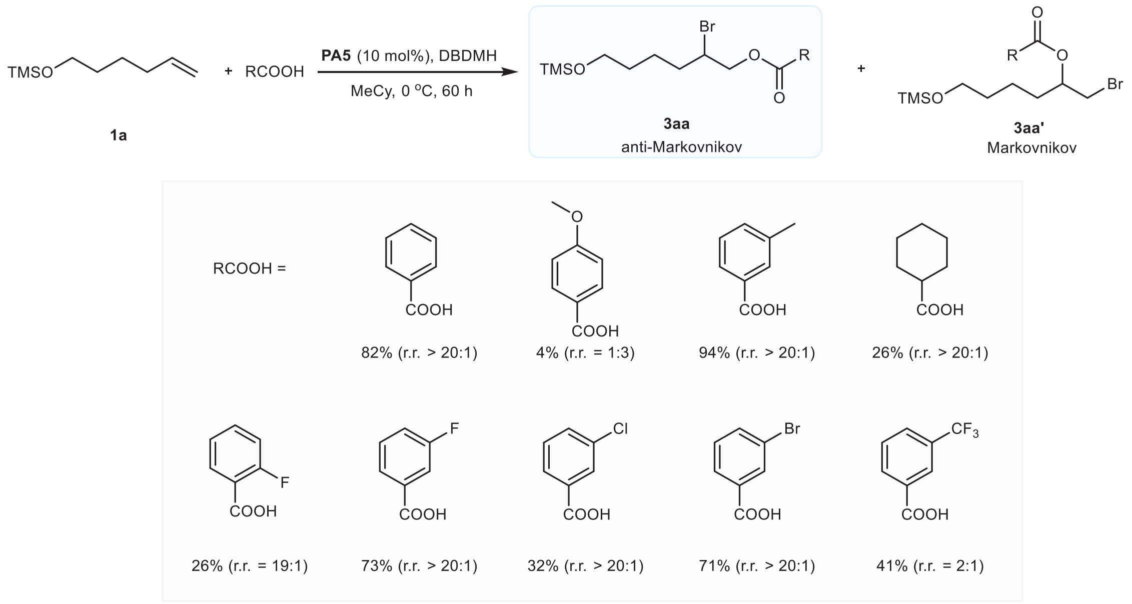

Fig. S6. Study on different carboxylic acids.

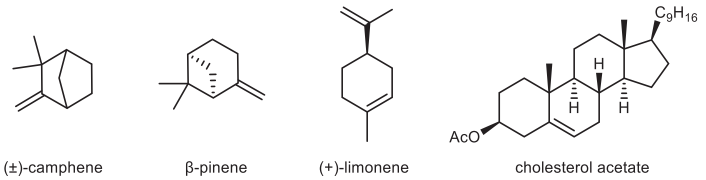

Fig. S7. A list of unreactive substrates.

Note: we have examined some cyclic olefins and no reaction was observed, attributed to steric exclusion from the ***PA5*** *binding pocket.*

2. <u>Mechanistic studies</u>
   <u>2.1 Determination of the binding ratio by Job's plot</u>

Pillar[5]arene **PA5** (0.1 mmol) was dissolved in $CDCl_3$ (1 mL) to give a stock solution at 0.1 mol/L concentration. Similarly, olefin **1c** (0.1 mmol) was added to $CDCl_3$ (1 mL) solution to provide a 0.1 mol/L stock solution. Then, the solutions of **PA5** and olefin **1c** were added to NMR tubes based on the specific ratio in Figure S8. The H_a chemical shift of free olefin **1c** solution (0.1 mol/L) is 2.0486 ppm in ^1H NMR. This Job's plot had a maximum when the mole fraction of olefin **1c** (X_{1c}) equals 0.5, suggesting that the binding stoichiometry between **PA5** and olefin **1c** is 1:1.

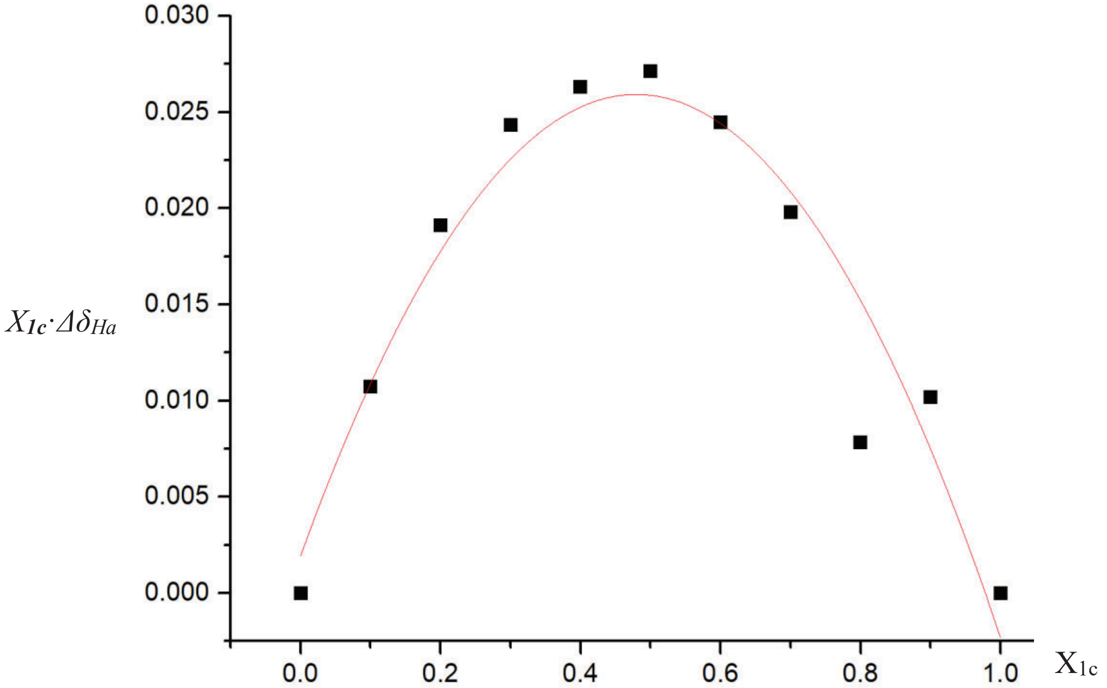

Entry	V_{P5} / µl	V_{1c} / µl	X_{1c}	δ_{Ha} / ppm	$\Delta\delta_{Ha}$ / ppm	$X_{1c}\cdot\Delta\delta_{Ha}$
1	90	10	0.1	1.9412	0.1074	0.0107
2	80	20	0.2	1.9530	0.0956	0.0191
3	70	30	0.3	1.9674	0.0812	0.0244
4	60	40	0.4	1.9828	0.0658	0.0263
5	50	50	0.5	1.9943	0.0543	0.0271
6	40	60	0.6	2.0078	0.0408	0.0245
7	30	70	0.7	2.0203	0.0283	0.0198
8	20	80	0.8	2.0388	0.0098	0.0078
9	10	90	0.9	2.0373	0.0113	0.0102
10	0	100	1.0	2.0486	0	0

Fig. S8. Job's plot of pillar[5]arene **PA5** and olefin **1c**.

2.2 Determination of the binding constant by titration experiments

The experiments were conducted by mixing different ratios of **PA5** and **1a** at 25 °C in NMR tubes. **PA5** (1.0 equiv, 0.01 mmol) and the different equivalents of **1a** (0 equiv, 1.0 equiv, 5.0 equiv, 10.0 equiv, 15.0 equiv, 20.0 equiv, 30.0 equiv) were dissolved in 0.6 mL $CDCl_3$. Then the mixtures were analyzed by ^{1}H NMR. The measured chemical shifts were plotted against the equivalents of **1a**, and the resulting curves were fitted using software in http://supramolecular.org.[12-15] For the calculations of the binding constant (*K*), a 1:1 binding was assumed. The binding constant (*K*), was found to be 6.12 M^{-1} ± 0.24.

Equivalents of **1a**	^{1}H NMR shift (ppm)
0	6.8408
1.0	6.8417
5.0	6.8424
10.0	6.8432
15.0	6.8438
20.0	6.8448
30.0	6.8452

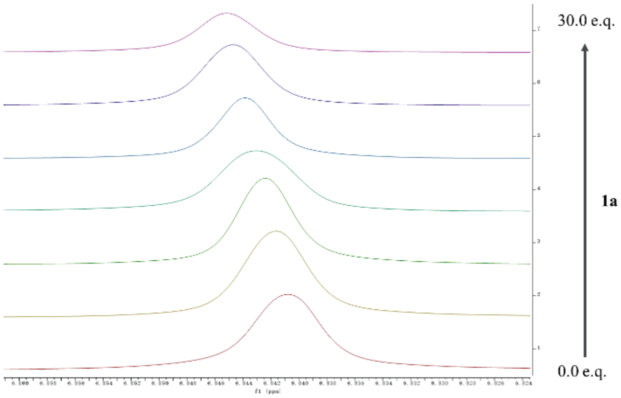

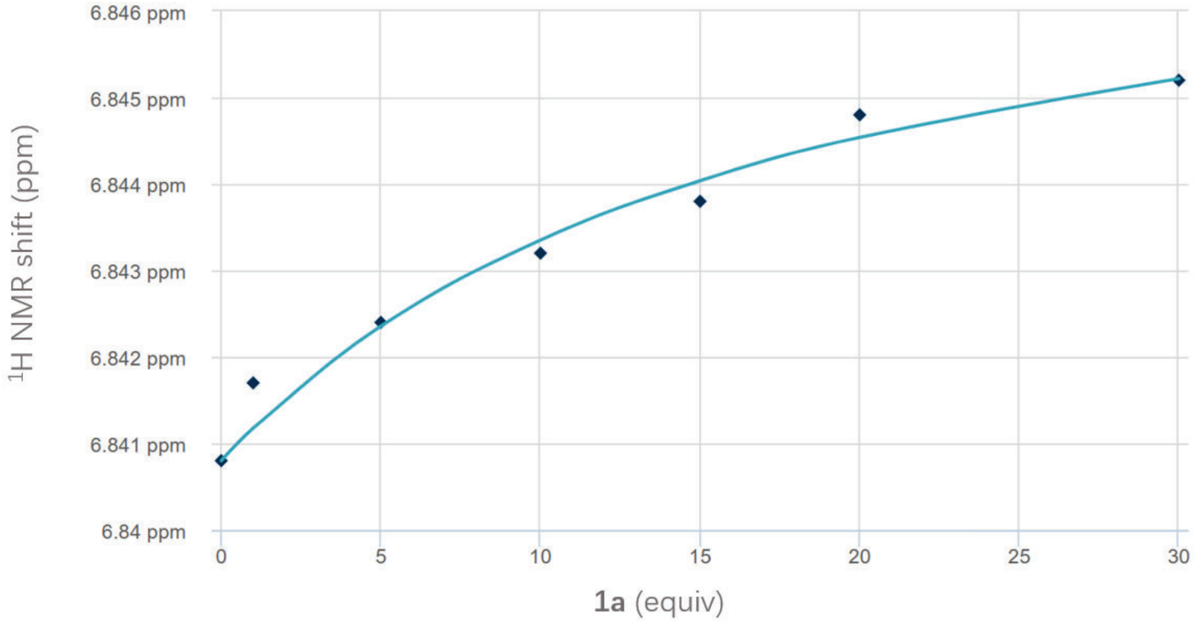

Fig. S9. The variation of the ^{1}H NMR chemical shift of **PA5** against the equivalent of **1a**.

2.3 Monitoring the reaction progress with **1a** and DBDMH with catalyst **PA5**.

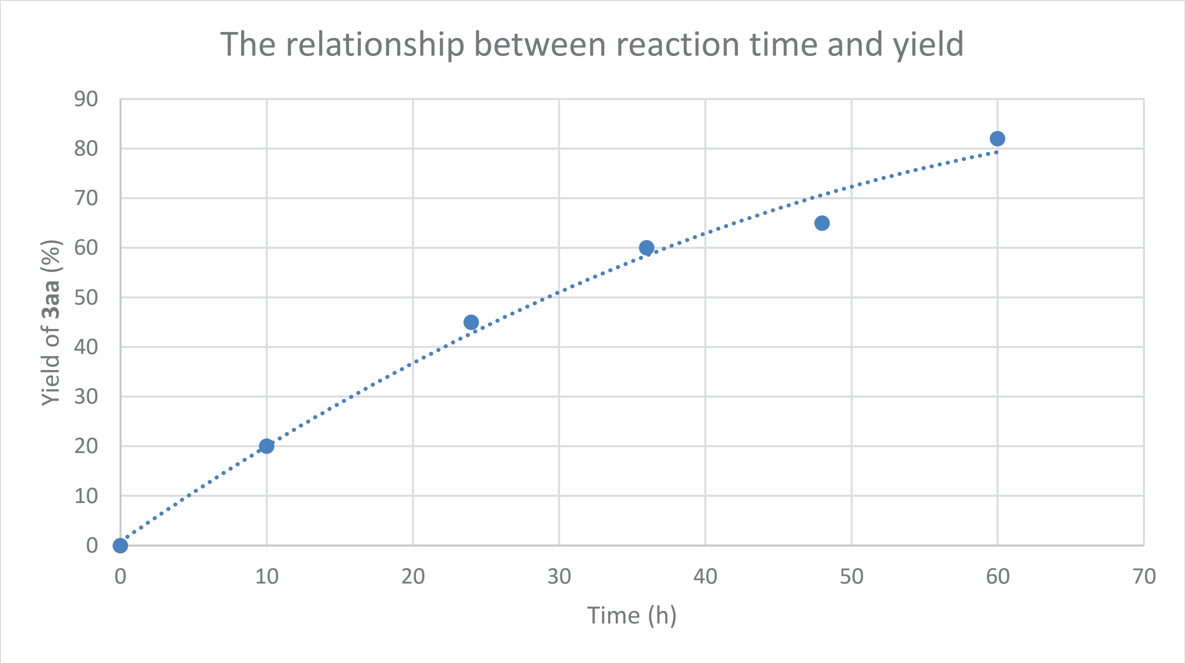

Entry	Time (h)	NMR yield (%)
1	0	0
2	10	20
3	24	45
4	36	60
5	48	65
6	60	80

Fig. S10. Monitoring the reaction progress using ^{1}H NMR.

2.4 $^{1}H - ^{1}H$ ROESY NMR

To a solution of a mixture of bromotosylate **14** (0.5 mmol) in $CDCl_3$ (1 mL) was added and TFA (0.2 mmol) at 23 ºC. Then, pillar[5]arene **PA5** (0.05 mmol) was added to the mixture, and the resultant solution was stirred for 5 minutes. The sample was studied using ^{1}H-^{1}H ROESY NMR.

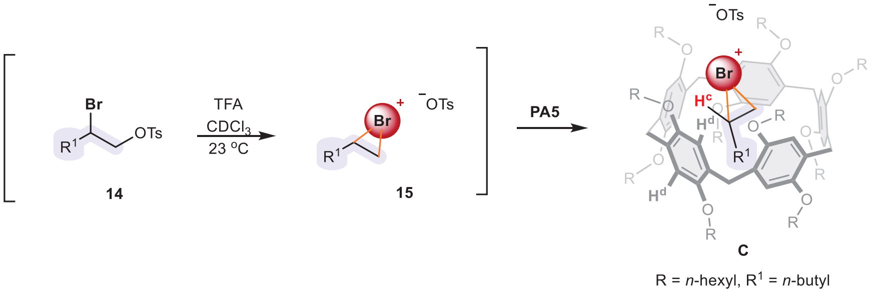

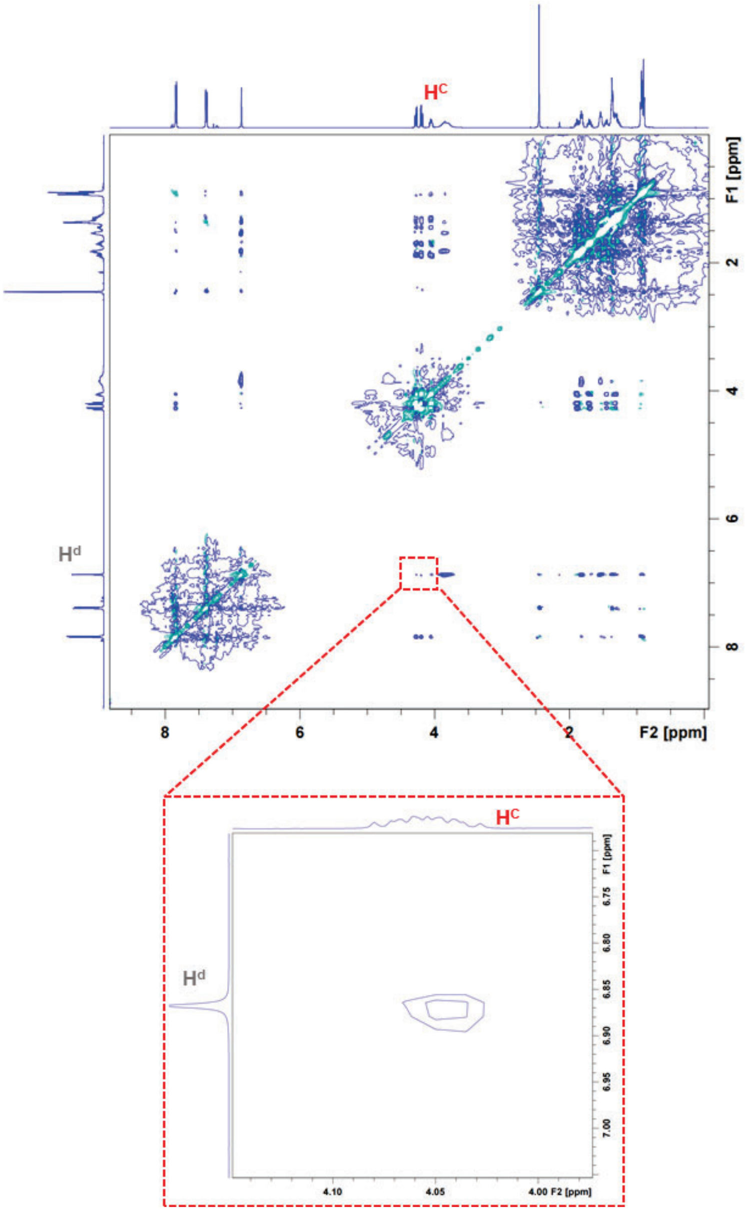

Fig. S11. $^{1}H - ^{1}H$ ROESY NMR of mixture of **14**/TFA/**PA5**.

2.5 IR Study on a mixture of **14**/TFA/**PA5**

To a solution of bromotosylate **14** (2 mmol) in CCl_4 (1 mL) was added TFA (0.2 mmol) at 23 °C. A recognizable absorption at 1360 cm^{-1} emerged, corresponding to the in situ generated bromiranium species. Upon the addition of pillar[5]arene **PA5** (2 mmol) to the in situ generated bromiranium species, a considerable blue-shift of the IR signal at around 1370 cm^{-1} was detected, attributed to the encapsulation of the bromiranium species in **PA5**.

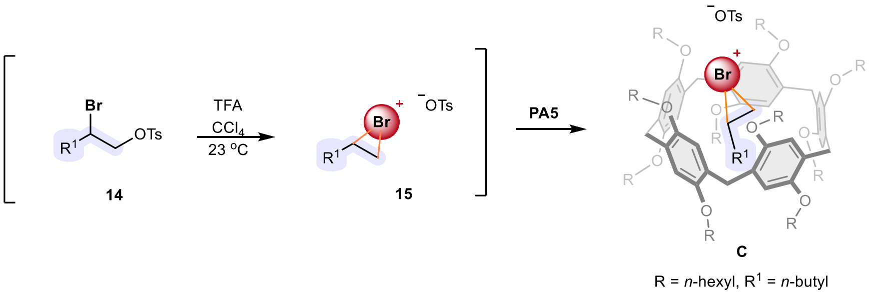

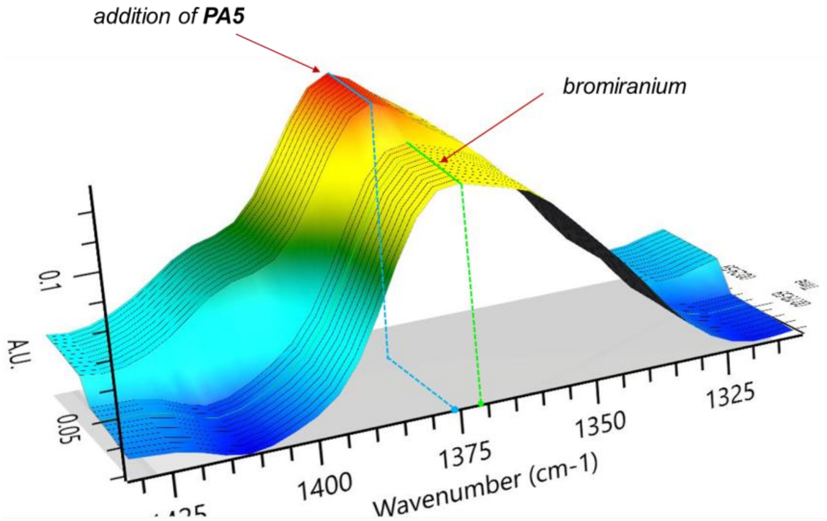

Fig. S12. In situ IR study of **14**/TFA/**PA5**.

3. Structural determination

The known compound **26** was reduced to **26**, which was then reacted with acyl chloride **27** to give **28**. The structure of **28** was unambiguously confirmed by X-ray crystallographic analysis. Compound **26** was also treated with benzoyl chloride to give compound **3ba**, which was identical to the sample of **3ba** obtained from **1b** via **PA5**-catalyzed anti-Markovnikov bromoesterification.

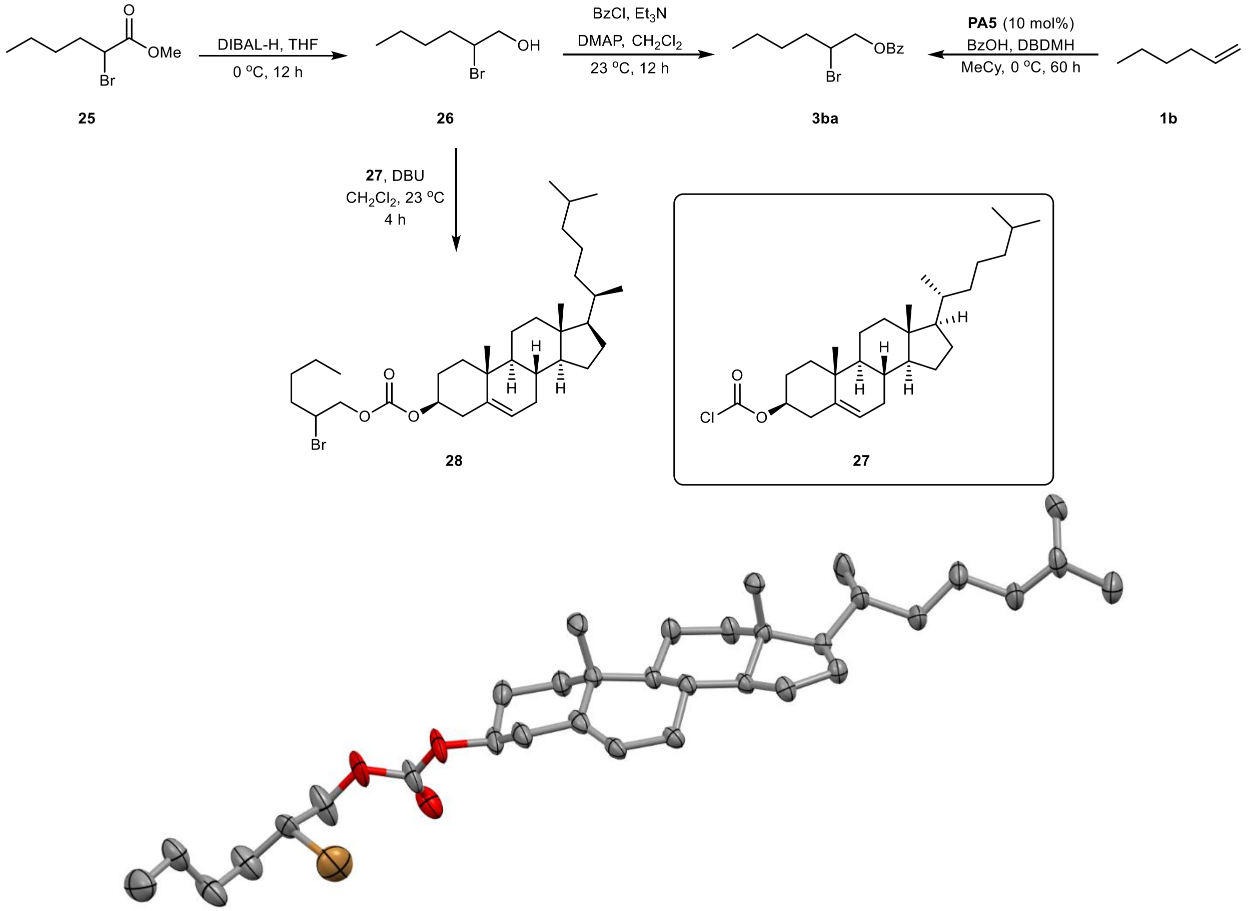

Fig. S13. Synthesis and X-Ray crystal structure of **28** (CCDC 2504582).

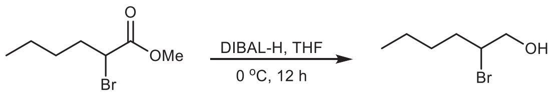

25 26

To a solution of methyl 2-bromohexanoate **25** (0.82 mL, 5 mmol) in THF (20 mL) was added DIBAL-H (1 M in toluene, 12 mL, 12 mmol) dropwise under N_2 atmosphere at 0 ºC. The resultant mixture was stirred at the same temperature for 12 h. Then, a small amount of water was added to quench this reaction. Saturated aqueous sodium tartrate solution was added to the mixture and stirred for 2 h until the mixture turned into a clear solution. The mixture was extracted by ethyl acetate (10 mL × 3). The combined organic phase was washed with brine (10 mL), dried over anhydrous Na_2SO_4, filtered, and concentrated under reduced pressure. The residue was purified over silica gel column chromatography (EtOAc:hexanes, 1:4) to afford the 2-bromohexan-1-ol **26** quantitively as a colorless oil.

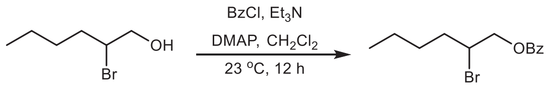

26 3ba

To a solution of 2-bromohexan-1-ol **26** (363 mg, 2 mmol), BzCl (310 mg, 2.2 mmol), and DMAP (24.4 mg, 0.2 mmol) in CH_2Cl_2 (5 mL) was added Et_3N (306 μL, 2.2 mmol). The resultant mixture was stirred at 23 ºC for 12 h. Then, water was added to the solution and the mixture was extracted by ethyl acetate (10 mL × 3). The combined organic phase was washed with saturated aqueous $NaHCO_3$ (5 mL × 3) and brine (5 mL), dried over anhydrous Na_2SO_4, filtered, and concentrated under reduced pressure. The residue was purified over silica gel column chromatography (EtOAc:hexanes, 1:30) to afford the 2-bromohexyl benzoate **3ba** in 90% yield as a colorless oil.

*2-bromohexan-1-ol (**26**)*

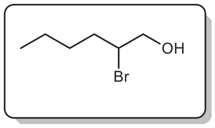

Yield: 99%
Physical state: colorless oil
R_f = 0.3 (1:4 EtOAc: hexane)
^{1}H NMR (500 MHz, $CDCl_3$) δ: 4.17 – 4.09 (m, 1H), 3.83 – 3.70 (m, 2H), 1.92 – 1.76 (m, 2H), 1.58 – 1.46 (m, 1H), 1.36 (ddd, J = 26.7, 14.3, 6.8 Hz, 3H), 0.91 (t, J = 7.2 Hz, 3H).
^{13}C NMR (126 MHz, $CDCl_3$) δ: 67.40, 60.25, 34.70, 29.70, 22.25, 14.01.
HRMS (ESI) calcd for $C_6H_{13}BrO$ $[M + Na]^+$: 203.00420, found: 203.00436.
^{13}C NMR (126 MHz, $CDCl_3$) δ: 145.32, 132.75, 130.10, 128.14, 72.33, 49.94, 34.42, 29.03, 22.10, 21.82, 13.97.
HRMS (ESI) calcd for $C_{13}H_{19}BrO_3S$ $[M + H]^+$: 357.01305, found: 357.01320.

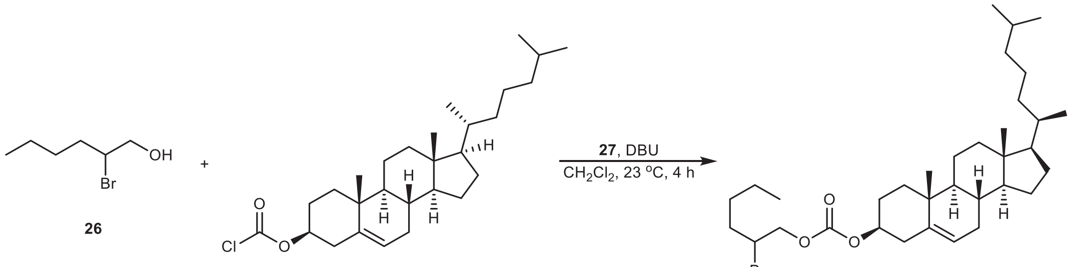

26 27 28

To a solution of 2-bromohexan-1-ol **26** (181 mg, 1 mmol) and **27** (494 mg, 1.1 mmol) in CH_2Cl_2 (5 mL) was added DBU (180 μL, 1.2 mmol). The resultant mixture was stirred at 23 ºC for 4 h. Then, water was added to the solution and the mixture was extracted by CH_2Cl_2 (20 mL × 3). The combined organic phase was washed with brine (5 mL), dried over anhydrous Na_2SO_4, filtered, and concentrated under reduced pressure. The residue was purified over silica gel column chromatography to (EtOAc:hexanes, 1:40) afford the compound **28**.

*2-bromohexyl ((3S,8S,9S,10R,13R,14S,17R)-10,13-dimethyl-17-((R)-6-methylheptan-2-yl)-2,3,4,7,8,9,10,11,12,13,14,15,16,17-tetradecahydro-1H-cyclopenta[a]phenanthren-3-yl) carbonate (**28**)*

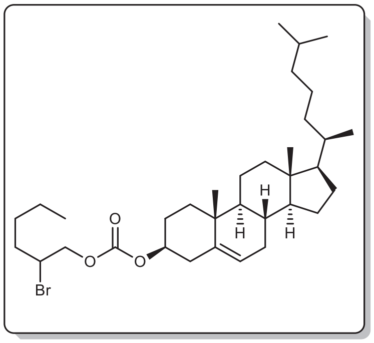

Yield: 71%
Physical state: white solid
R_f = 0.6 (1:40 EtOAc: hexane)
^{1}H NMR (500 MHz, $CDCl_3$) δ: 5.40 (d, *J* = 4.5 Hz, 1H), 4.49 (tt, *J* = 10.6, 5.2 Hz, 1H), 4.41 – 4.26 (m, 2H), 4.12 (dq, *J* = 10.3, 6.0 Hz, 1H), 2.41 (q, *J* = 7.1 Hz, 2H), 2.08 – 1.62 (m, 8H), 1.61 – 1.21 (m, 14H), 1.21 – 0.81 (m, 25H), 0.67 (s, 3H).
^{13}C NMR (126 MHz, $CDCl_3$) δ: 154.24, 139.39, 123.21, 78.51, 70.78, 56.82, 56.26, 51.27, 50.12, 42.45, 39.85, 39.66, 38.10, 36.98, 36.68, 36.32, 35.93, 34.84, 32.04, 31.97, 29.40, 28.37, 28.16, 27.78, 24.42, 23.97, 22.97, 22.71, 21.18, 19.41, 18.86, 14.04, 12.00.
HRMS (ESI) calcd for $C_{34}H_{57}BrO_3$ $[M + Na]^+$: 615.33833 found: 615.33719

4. Computational studies

Geometry optimizations were carried out with the long-range-corrected ωB97XD functional (to address the dispersion interaction between all reactants),[1-3] with the 6-31G(d,p) basis set as implemented in the Gaussian 16 suite of programs.[4] Energies were further refined at the ωB97XD/6-311+G(d,p) level and single-point solvation energy correction (in methylcyclohexane) using Trhular's SMD model.[4] The relative Gibbs energies (ΔG_{rel}) in kcal/mol are provided with respect to the infinitely separated reactants unless stated otherwise. The CM5 charges, which use a single set of parameters to convert Hirshfeld charges into a more accurate representation of the electrostatic potential, free from errors associated with buried atoms in larger molecules, are employed to assess individual atomic charges.[5] All reported free energies involve zero-point vibrational corrections and thermal corrections to the Gibbs free energy on the gas-phase optimized geometries at 298.15 K.

Additionally, non-covalent interaction (NCI) isosurfaces are generated using Multiwfn 3.8 with the independent gradient model (IGM) based on promolecular density. Bader's quantum theory of atoms in molecules (QTAIM) analysis of the binding structures are performed to identify crucial host-guest interactions, using the Multiwfn 3.8.[6] The molecular graphs are visualized using VMD 1.9.3. The energy decomposition analysis (EDA) of the host-guest complexes was also performed at B3LYP-D3/ 6-31G(d) for the interaction energies between the fragments using the most recently proposed sobEDA and sobEDAw (eq. 1) based on the DFT with dispersion correction[7] as implemented in Multiwfn 3.8 in conjunction with Gaussian 16.

$$\Delta E_{\text{int}}=\Delta E_{\text{els}}+\Delta E_{\text{xrep}}+\Delta E_{\text{orb}}+\Delta E_{\text{disp}} \qquad (1)$$

The apparent non-covalent interactions are further assessed by the intrinsic bond stabilization index (IBSI) which is a framework of IGM[8] based on Hirshfeld partition of molecular density as implemented in Multiwfn 3.8 and the orbital contributions are identified through Natural Bond Orbital (NBO) analysis[9] as implemented in Gaussian 16 suite of programs.

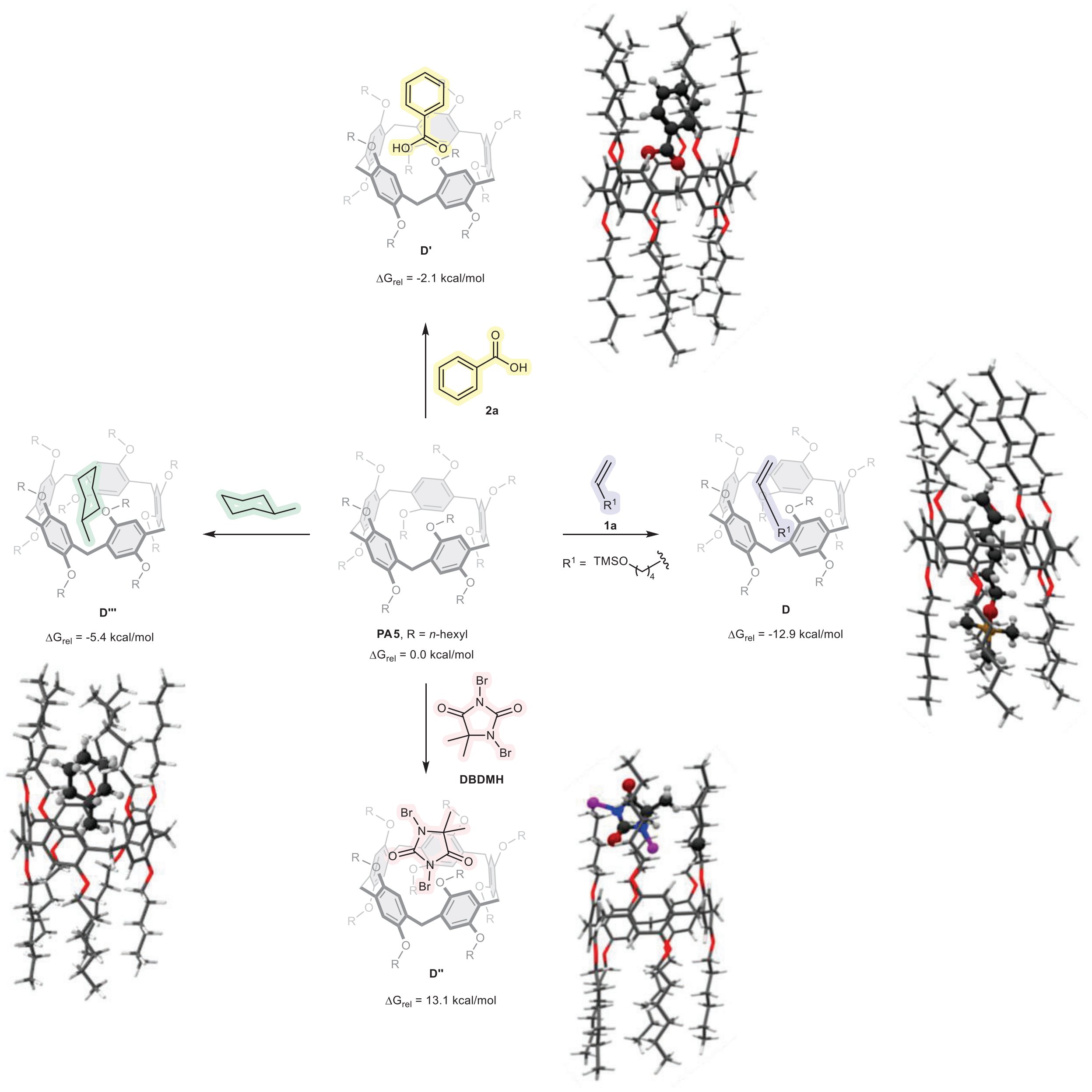

Fig. S14. Relative energies of the encapsulated species.
*Note: The encapsulation of olefin **1a** was found to be more energetically feasible than the encapsulation of benzoic acid (**2a**), 1,3-dibromo-5,5-dimethylhydrantoin (DBDMH), or methylcyclohexane.*

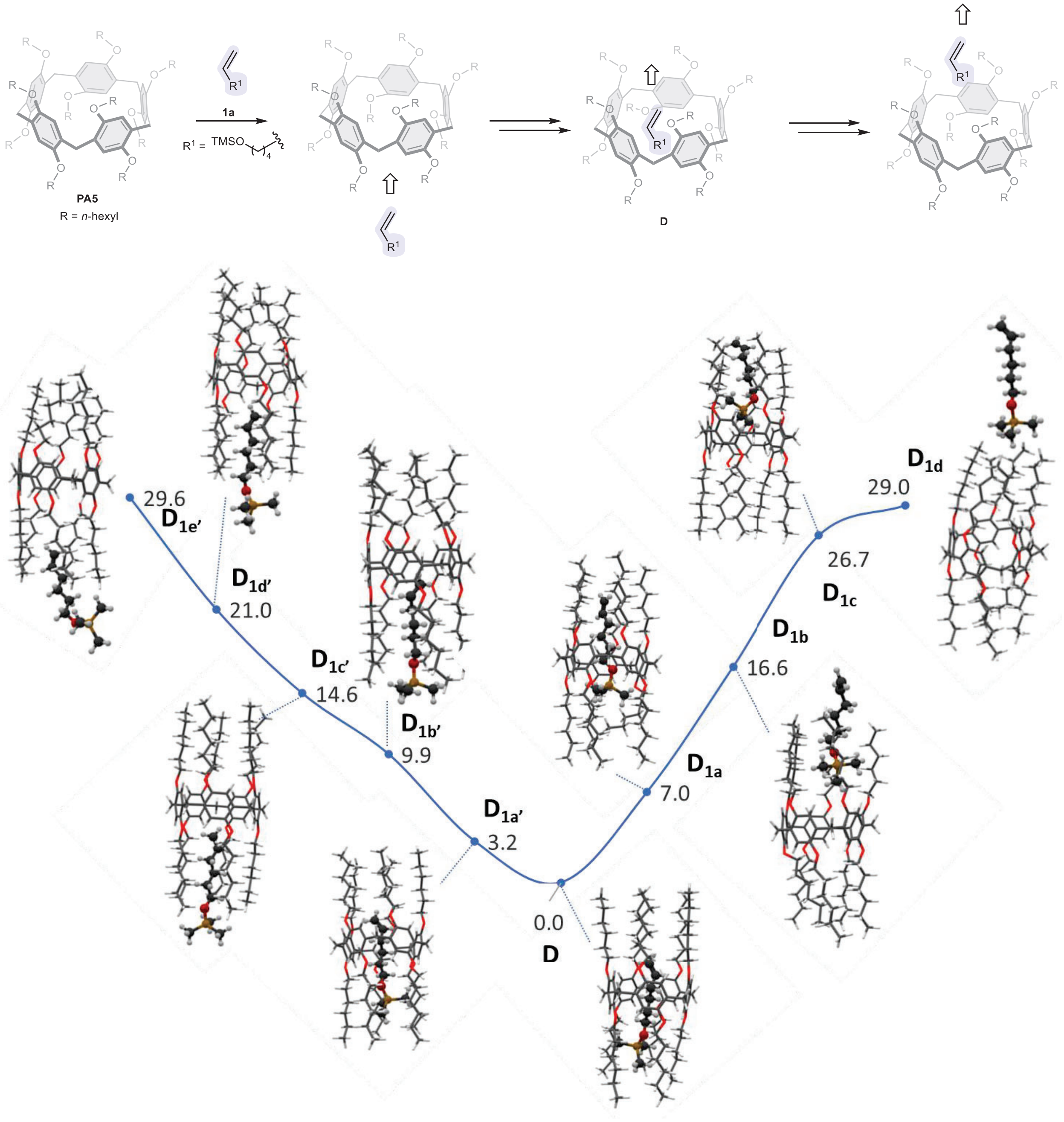

Fig. S15. Potential energy surface (PES) scan of the olefin **1a** inside **PA5**. ΔE_{rel} are given in kcal/mol with respect to the stable geometry of **D**.

Note: The PES scan was conducted based on the distance between the centroid of the pillararene and the terminal olefinic carbon, without imposing any geometrical constraints.

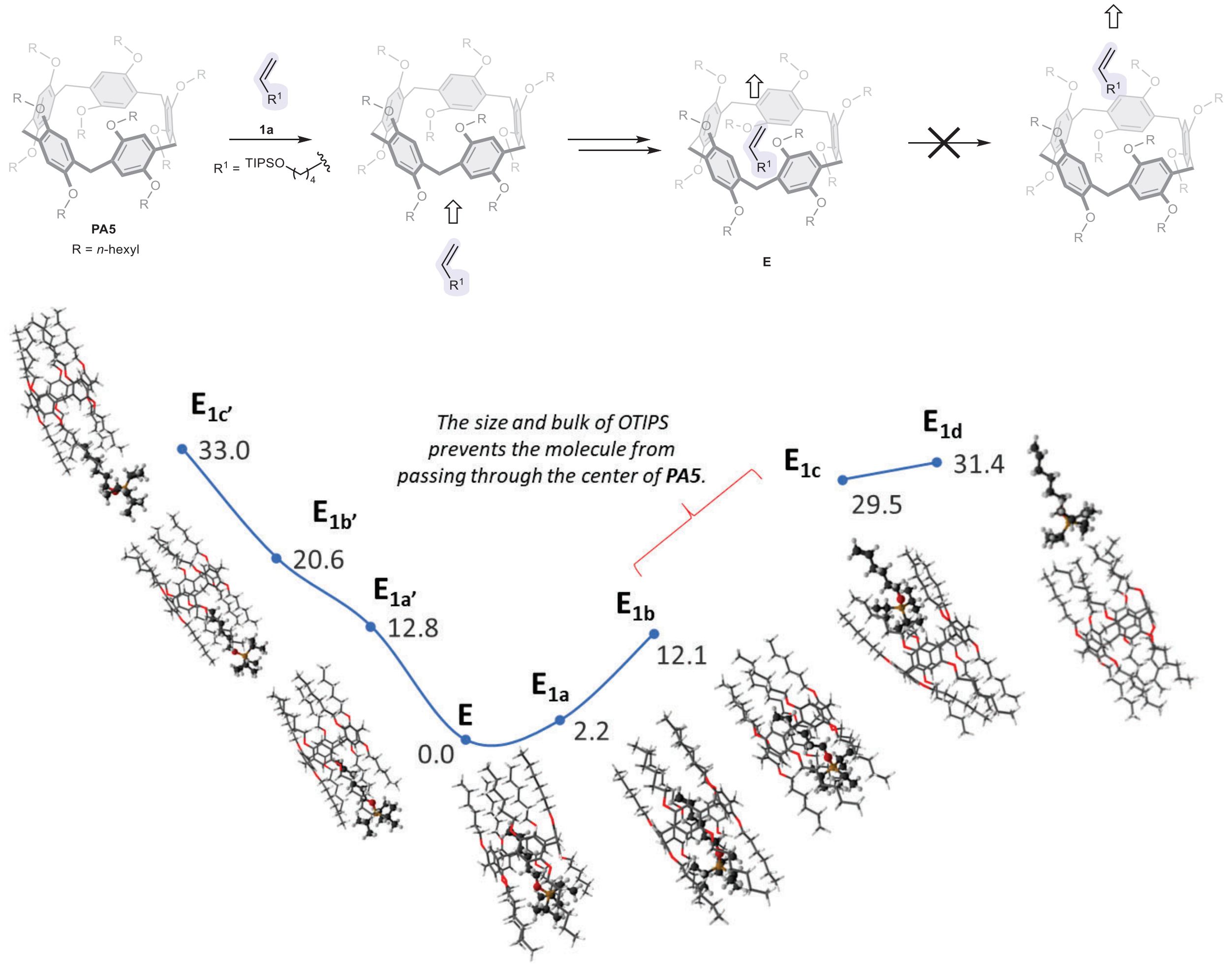

Fig. S16. Potential energy surface (PES) scan of the olefin **1h** inside **PA5**. ΔE_{rel} are given in kcal/mol with respect to the stable geometry of **E**.

Note: The PES scan was conducted based on the distance between the centroid of the pillararene and the terminal olefinic carbon, without imposing any geometrical constraints. ***1h*** *was found to be unable to pass through* ***PA5****, probably due to the steric hindrance of the TIPS group.*

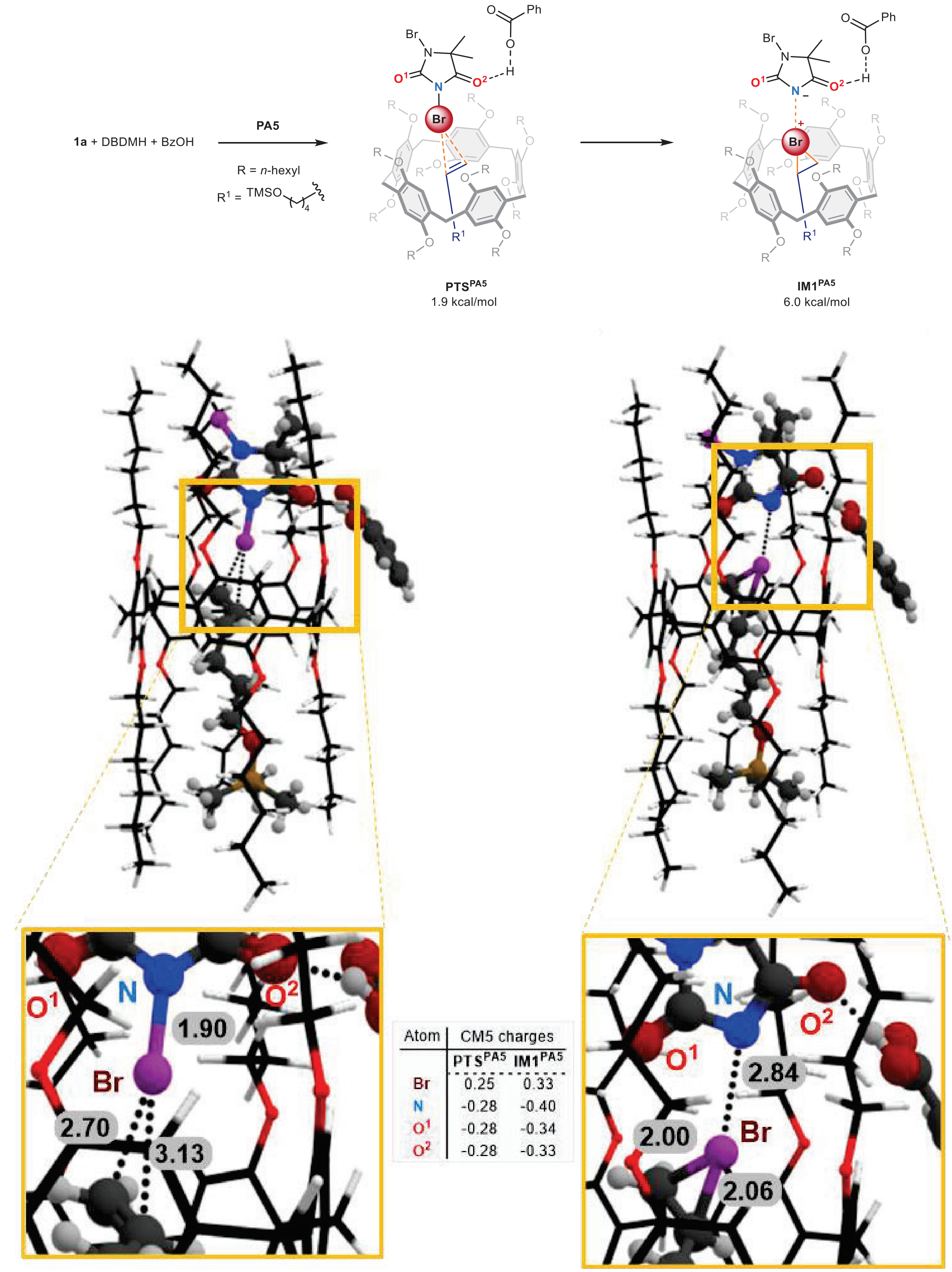

Atom	CM5 charges	
	PTS^{PA5}	$IM1^{PA5}$
Br	0.25	0.33
N	-0.28	-0.40
O^1	-0.28	-0.34
O^2	-0.28	-0.33

Fig. S17. Energetics of the formation of bromiranium ion inside **PA5**.

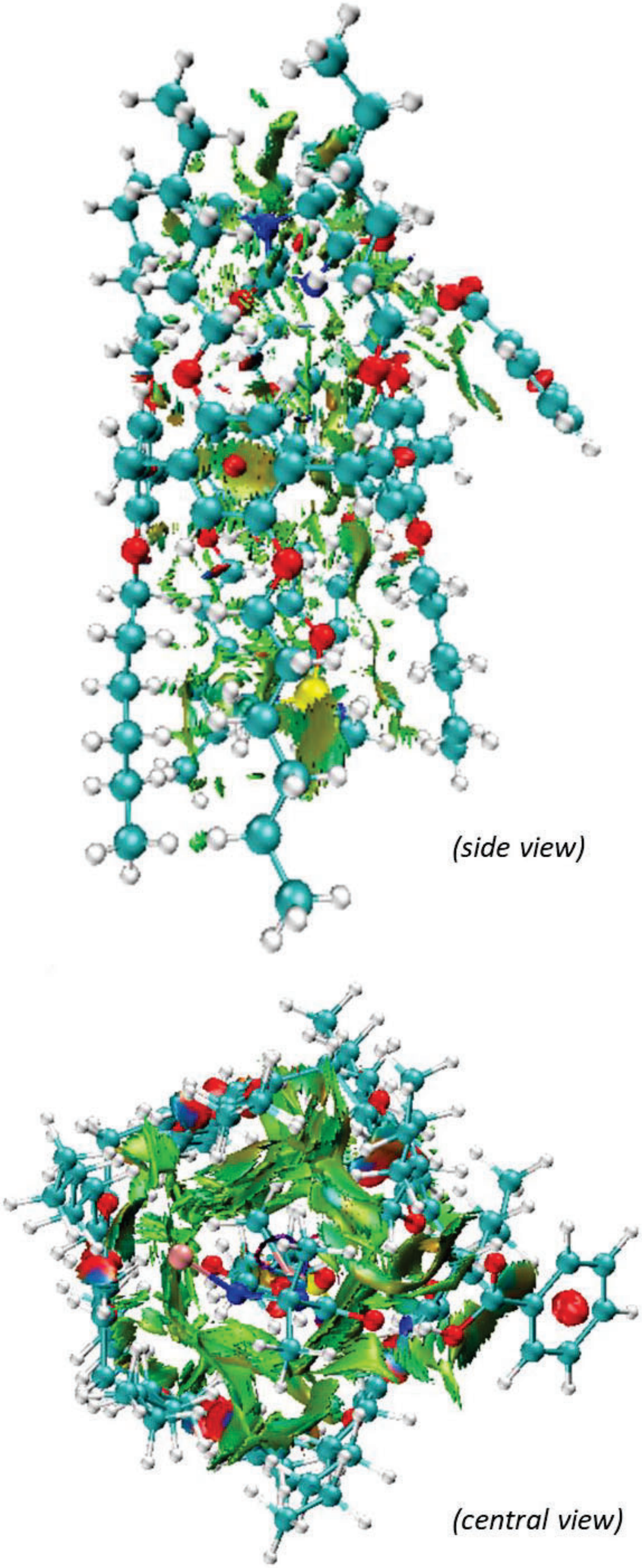

Fig. S18. Plots depicting NCI and AIM analyses of species **IM1^{PA5}**.

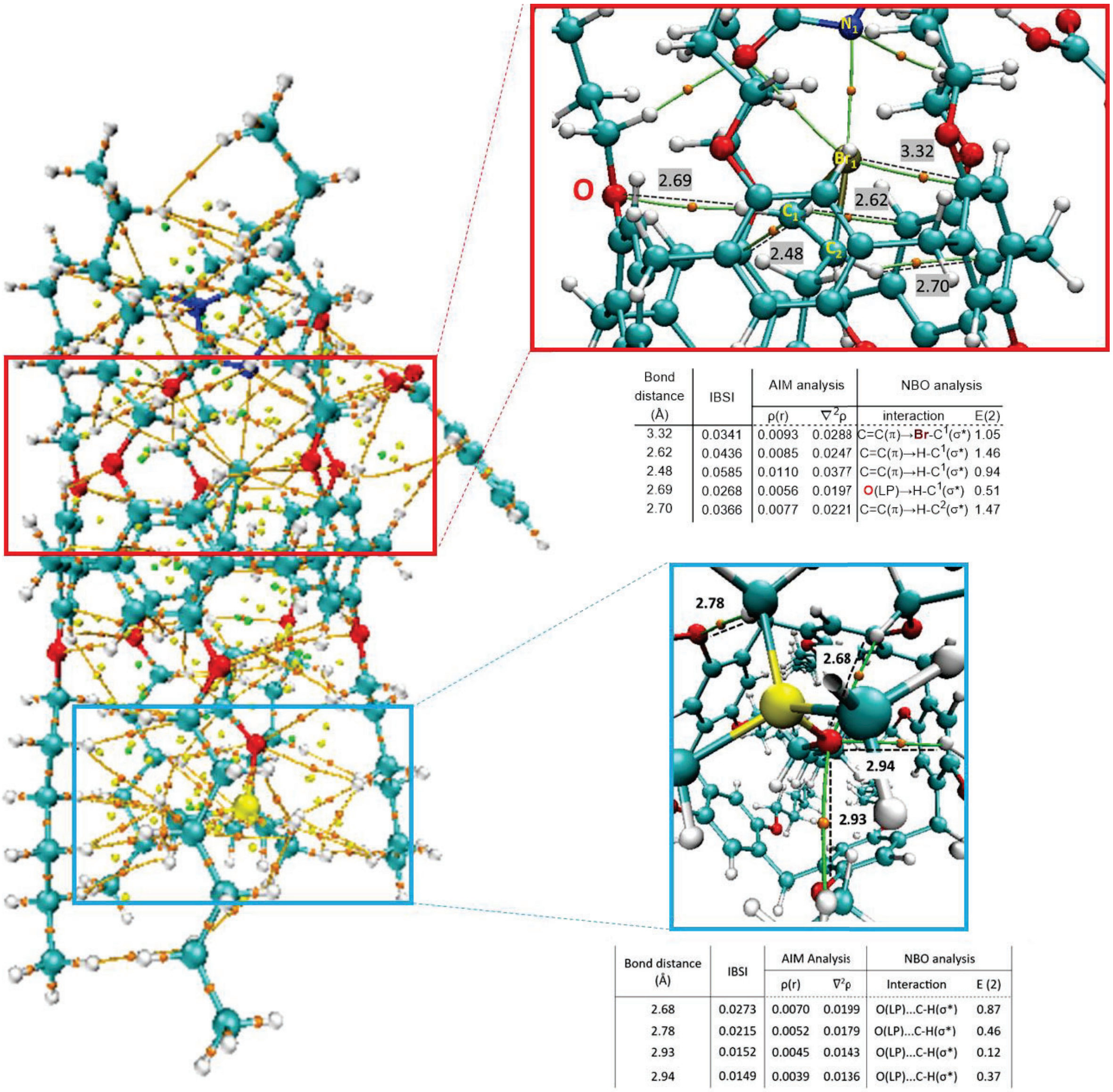

Bond distance (Å)	IBSI	AIM analysis		NBO analysis	
		ρ(r)	$\nabla^2\rho$	interaction	E(2)
3.32	0.0341	0.0093	0.0288	C=C(π)→**Br**-C^1(σ*)	1.05
2.62	0.0436	0.0085	0.0247	C=C(π)→H-C^1(σ*)	1.46
2.48	0.0585	0.0110	0.0377	C=C(π)→H-C^1(σ*)	0.94
2.69	0.0268	0.0056	0.0197	O(LP)→H-C^1(σ*)	0.51
2.70	0.0366	0.0077	0.0221	C=C(π)→H-C^2(σ*)	1.47

Bond distance (Å)	IBSI	AIM Analysis		NBO analysis	
		ρ(r)	$\nabla^2\rho$	Interaction	E (2)
2.68	0.0273	0.0070	0.0199	O(LP)...C-H(σ*)	0.87
2.78	0.0215	0.0052	0.0179	O(LP)...C-H(σ*)	0.46
2.93	0.0152	0.0045	0.0143	O(LP)...C-H(σ*)	0.12
2.94	0.0149	0.0039	0.0136	O(LP)...C-H(σ*)	0.37

Fig. S19. NBO and AIM analyses of the formation of bromiranium ion without **PA5**.

*Note: In species **IM1^{PA5}**, a number of non-covalent interactions between the encapsulated bromiranium cationic species and the phenyl ether groups **PA5** were observed. These include C=C(π)→Br-C(σ*), C=C(π)→H-C(σ *), and O(LP)→H-C(σ *), with ρ(r) values at the BCPs ranging from 0.005 to 0.011 a.u., and $\nabla^2\rho$ values at the BCPs varying from 0.019 to 0.037 a.u. In addition, the OTMS group was found to have multiple O(LP)→H-C(σ *) interactions with n-hexyl groups of **PA5**.*

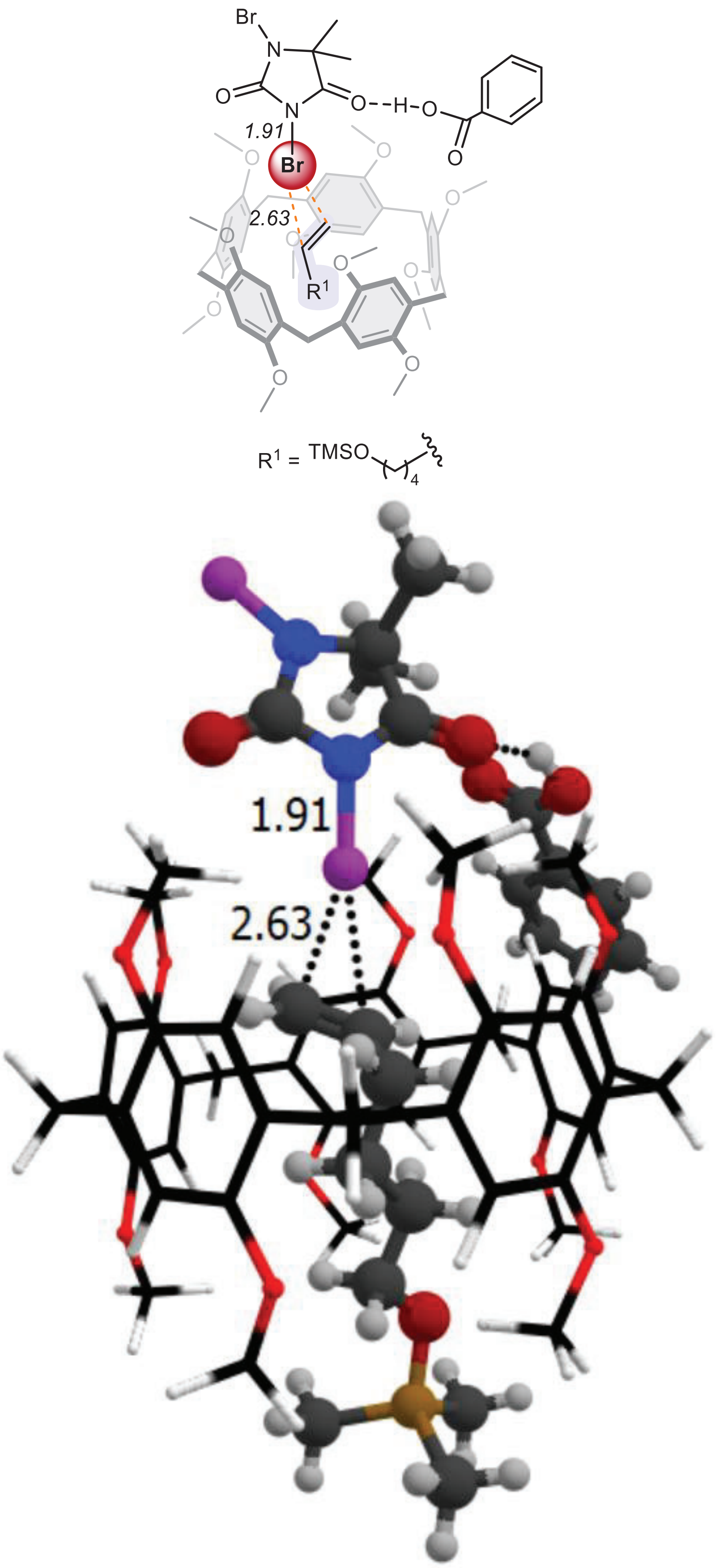

Fig. S20. Optimized geometry of $\mathbf{PTS^{PA1}}$. Bond distances are given in Å.

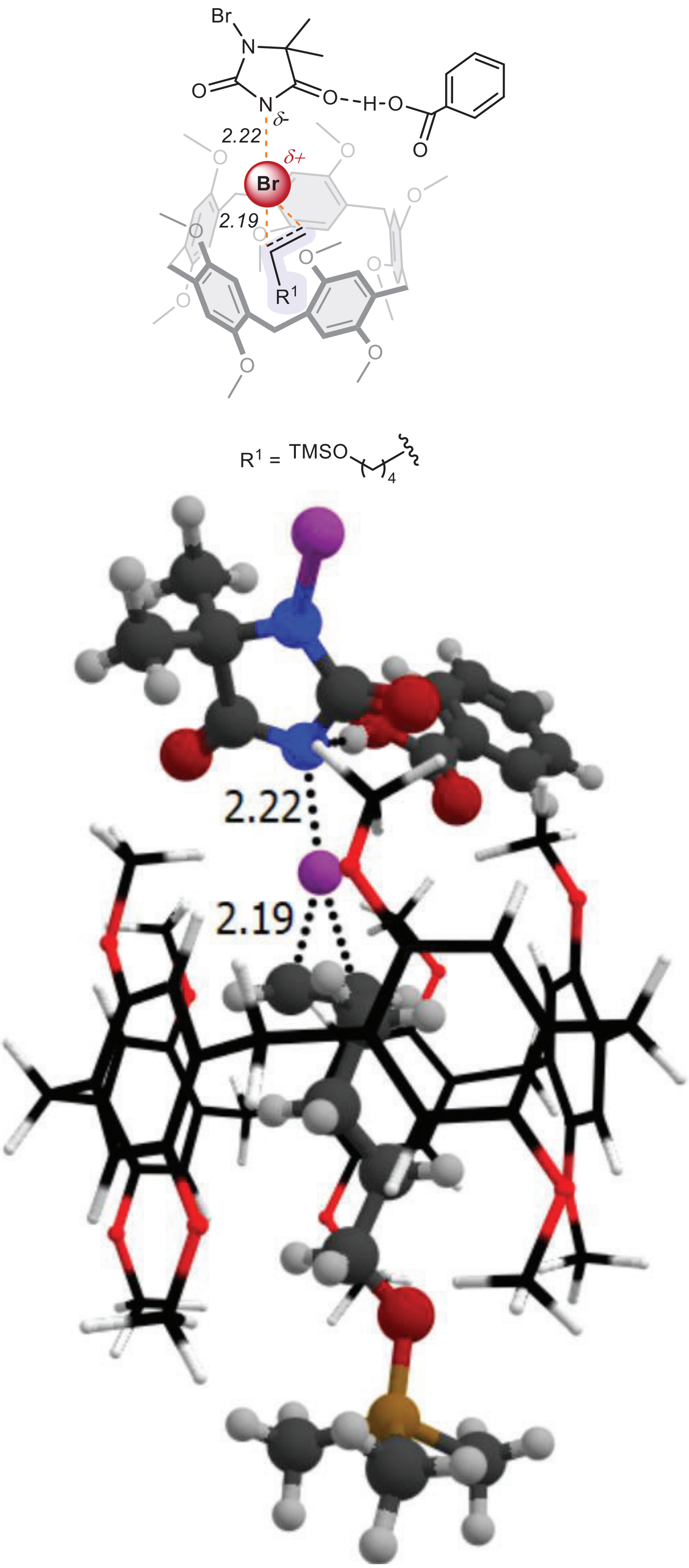

Fig. S21. Optimized geometry of **TS1^{PA1}**. Bond distances are given in Å.

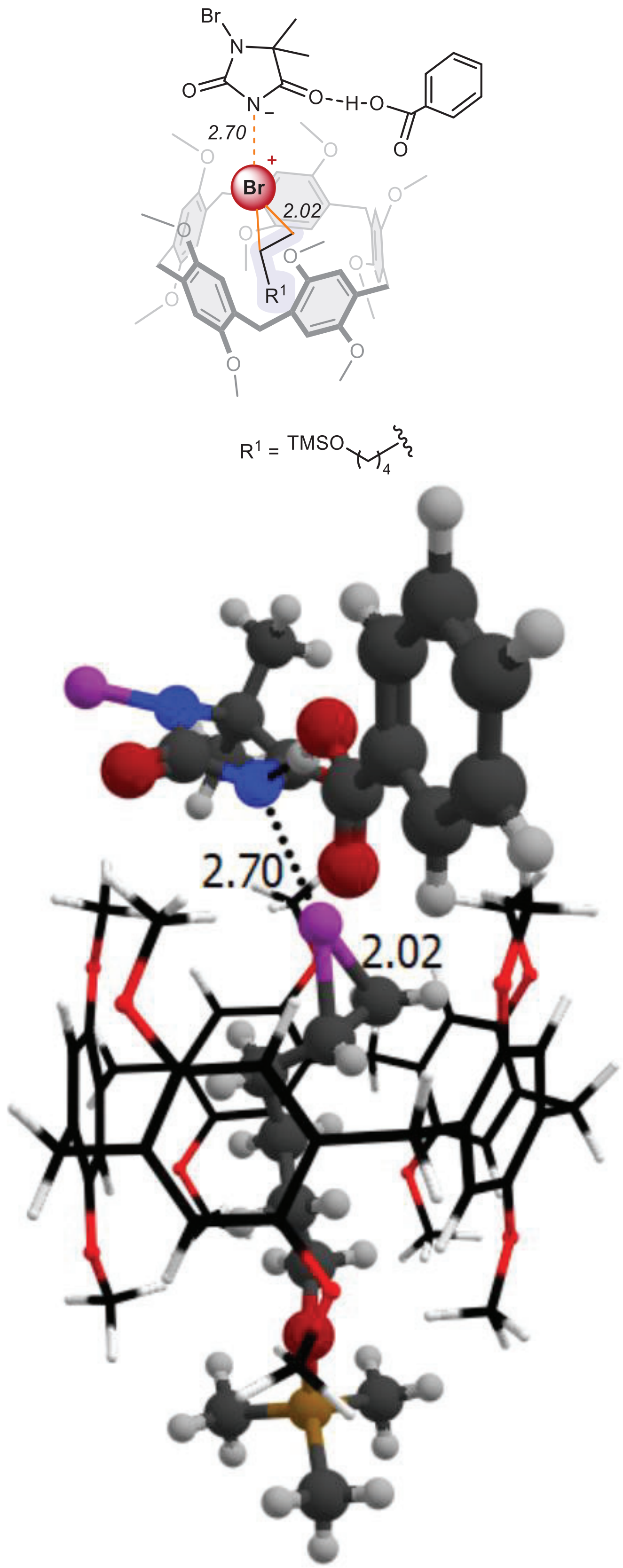

Fig. S22. Optimized geometry of **IM1**$^{\mathbf{PA1}}$. Bond distances are given in Å.

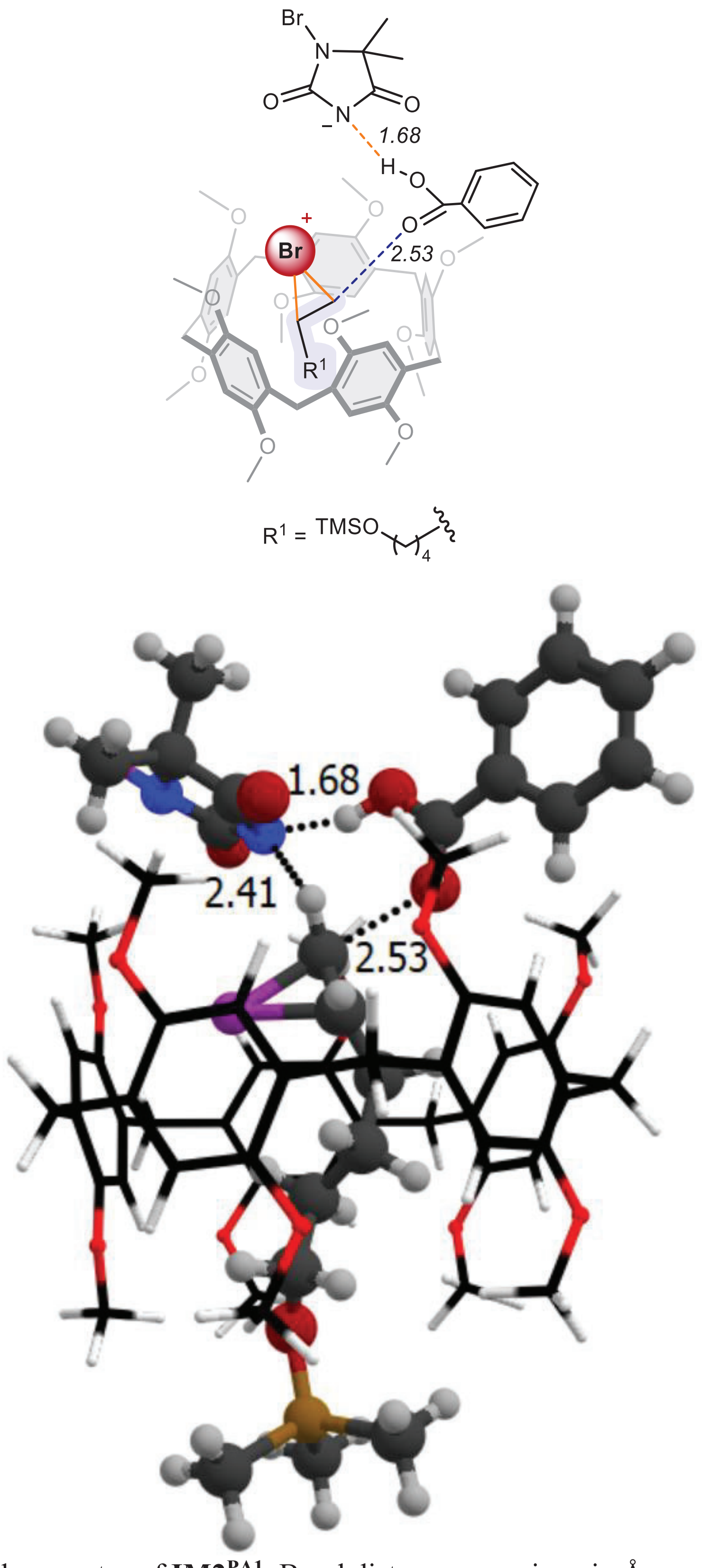

Fig. S23. Optimized geometry of **IM2^{PA1}**. Bond distances are given in Å.

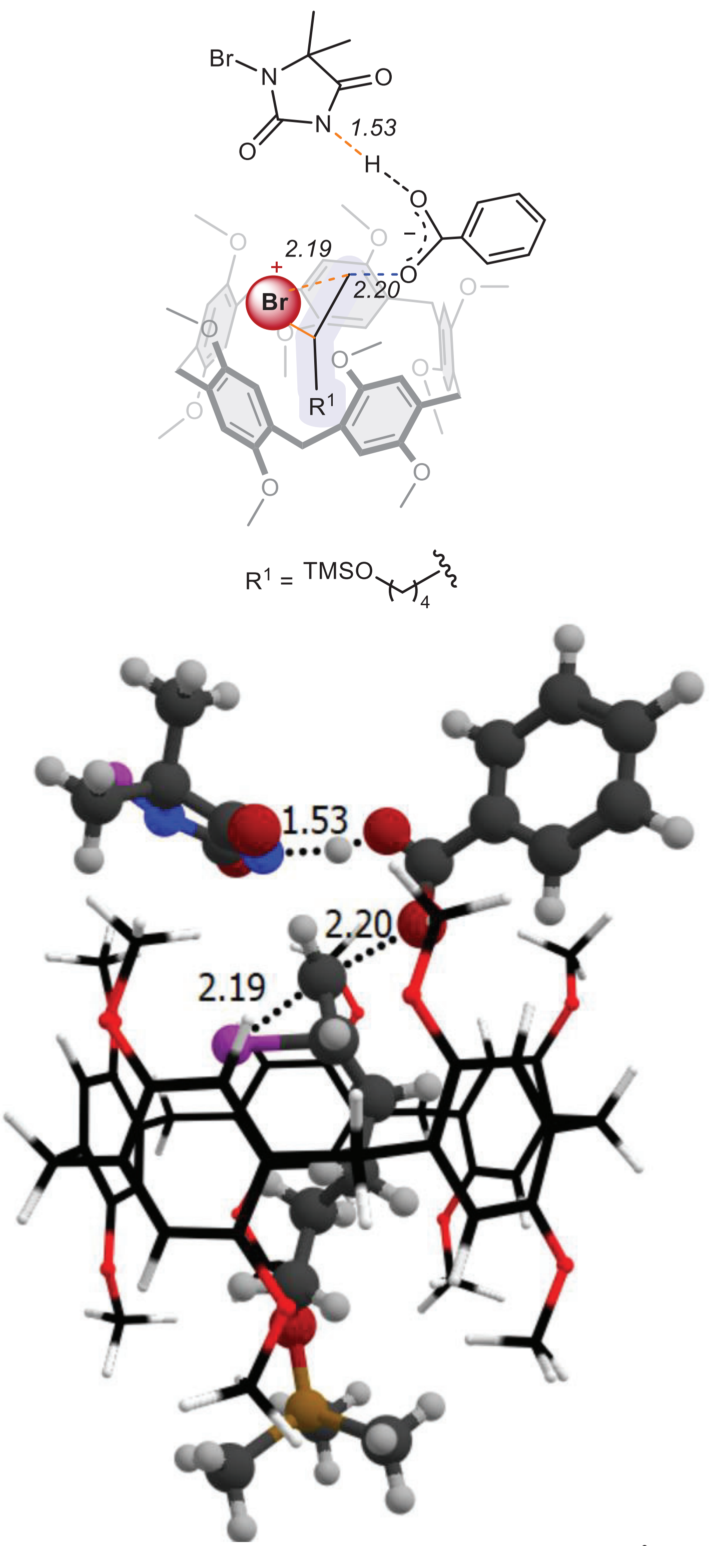

Fig. S24. Optimized geometry of $\mathbf{TS2^{PA1}}$. Bond distances are given in Å.

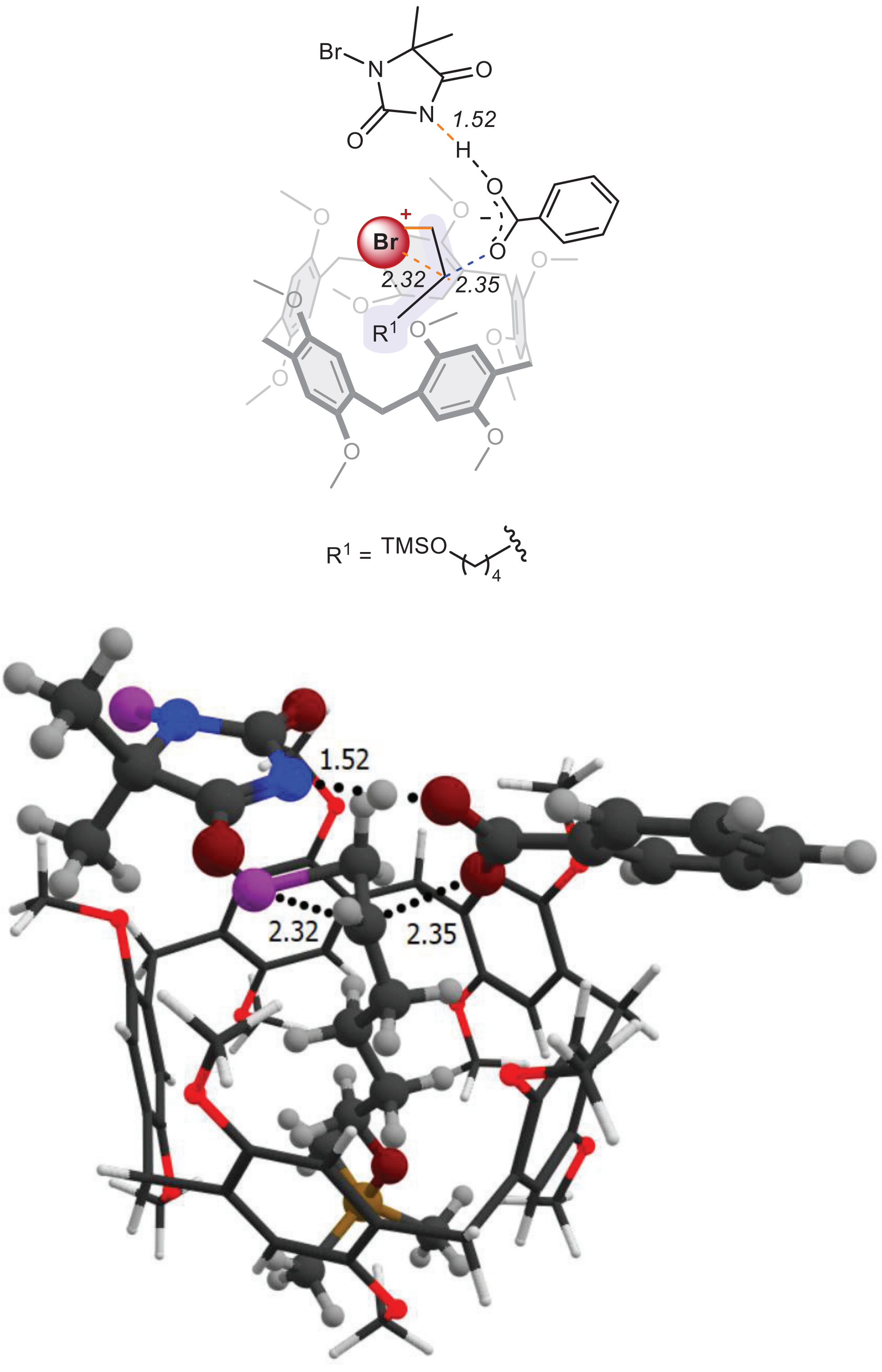

Fig. S25. Optimized geometry of **TS3^{PA1}**. Bond distances are given in Å

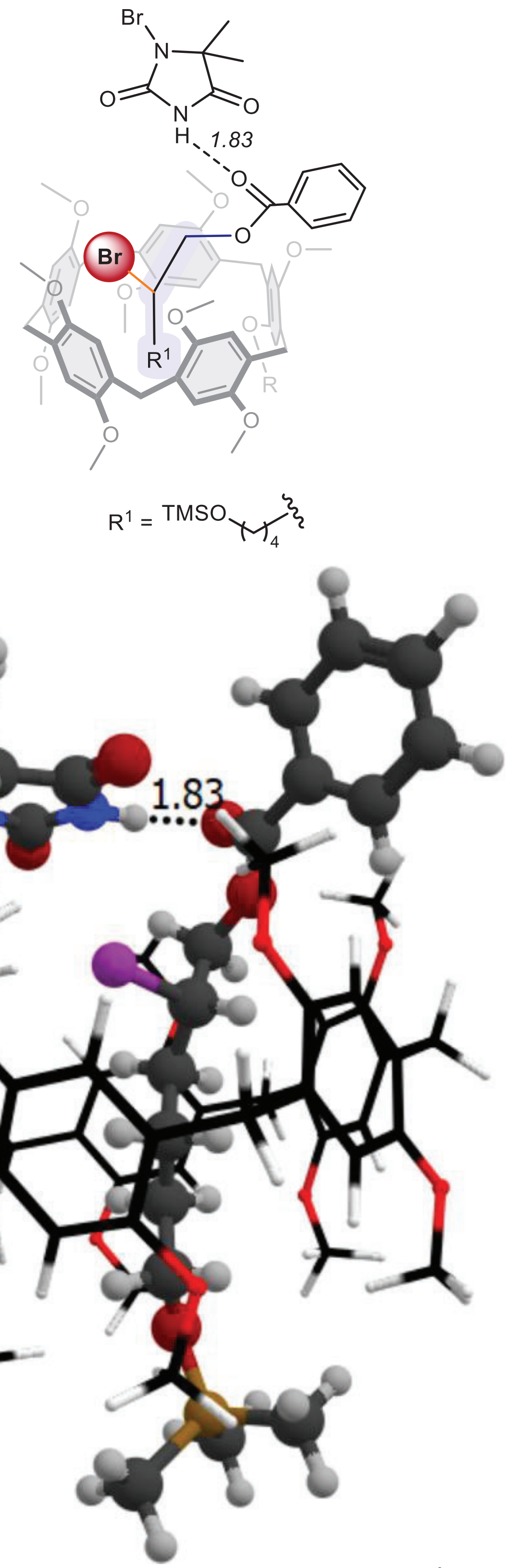

Fig. S26. Optimized geometry of $\mathbf{P^{PA1}}$. Bond distances are given in Å.

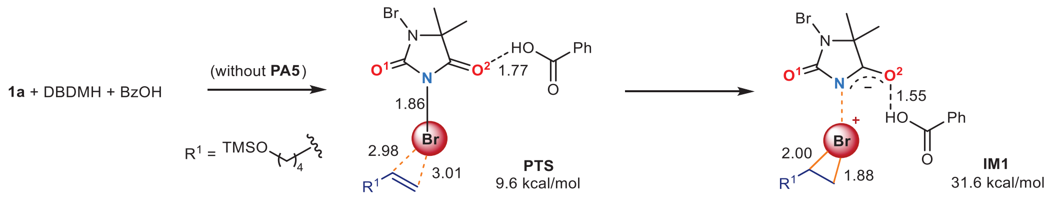

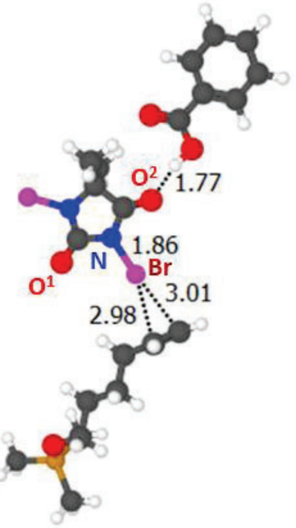

Atom	CM5 charges	
	**PTS**	**IM1**
**Br**	0.19	0.29
**N**	-0.28	-0.43
**O1**	-0.33	-0.37
**O2**	-0.30	-0.35

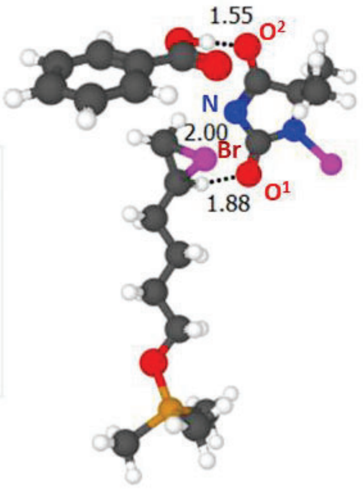

Fig. S27. Energetics of the formation of bromiranium ion without **PA5**.
*Note: In the absence of **PA5** encapsulation, the likelihood of forming the bromiranium species **PTS** is improbable, given its significantly higher energy level (31.6 kcal/mol) compared to the initial substrate species **IM1**.*

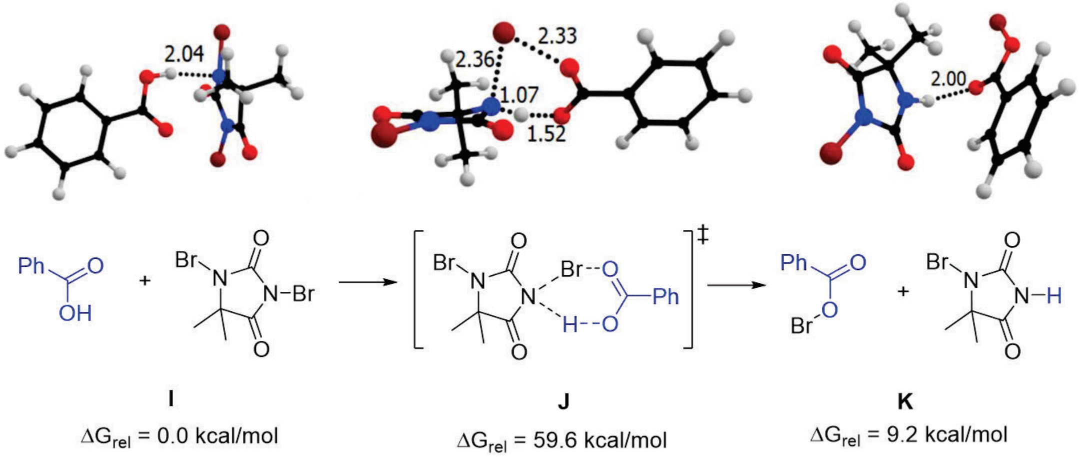

Fig. S28. Exploring an alternative mechanism to identify the origin of the active bromonium ion (Br^{+}). Bond distances in the optimized geometries are given in Å.

	ΔE_{Int}	Δ_{Eles}	ΔE_{Xrep}		ΔE_{orb}	ΔE_{C}		ΔE_{Xrepc}	ΔE_{dis}
			ΔE_{x}	ΔE_{rep}		ΔE_{DFTc}	ΔE_{dc}		
**PTS^{PA5}** (2FN)	-68.66	-60.88	-59.72	261.92	-24.16	-71.12	-114.70	172.26	-155.88
**IM1^{PA5}** (2FN)	-94.22	-81.53	-67.54	286.28	-35.79	-75.29	-120.35	187.60	-164.51
**PTS^{PA5}** (3FN)	-74.32	-74.82	-72.60	305.86	-38.65	-75.88	-118.23	201.68	-162.53
**IM1^{PA5}** (3FC)	-178.50	-166.23	-75.50	315.46	-48.78	-79.28	-124.17	212.19	-175.68
**PTS***(2FN)	-5.63	-16.19	-12.24	42.60	-11.69	-4.58	-3.53	29.75	-7.49
**IM1***(2FC)	-84.73	-79.64	-8.50	29.92	-18.54	-4.16	-3.82	21.42	-7.97
**PTS** (2FN)	-6.50	-11.39	-7.92	28.42	-7.44	-4.09	-4.06	19.59	-7.25
**IM1** (2FC)	-102.30	-107.24	-19.49	71.79	-22.74	-11.51	-13.11	51.69	-24.02

2F means two Fragments, 3F means 3 Fragments, N means Neutral and C means Charged.
PTS* means single point geometry (without any deformation) of the reactant complexes in **PTS^{PA5}** without **PA5** encapsulation.
IM1* means single point geometry (without any deformation) of the reactant complexes in **IM1^{PA5}** without **PA5** encapsulation.

$$\Delta E_{int} = \Delta E_{els} + \Delta E_{x} + \Delta E_{rep} + \Delta E_{orb} + \Delta E_{DFTc} + \Delta E_{dc}$$
$$\Delta E_{int} = \Delta E_{els} + \Delta E_{xrep} + \Delta E_{orb} + \Delta E_{disp}$$

Table S1. The energy decomposition analysis (EDA) of the host-guest complexes.

*Note: The EDA reveals that while electrostatic interaction plays a key role in the formation of the bromonium ion, dispersion forces significantly contribute to the interactions between **PA5** and the reactants, enhancing the host-guest interactions.*

5. Catalyst preparation

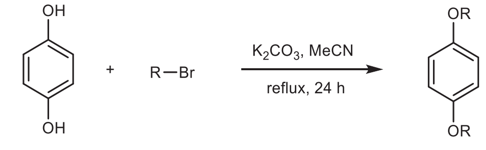

S1

To a stirred solution of hydroquinone (5 mmol, 1.0 equiv) and alkyl bromide (15 mmol, 3.0 equiv) in acetonitrile (10 mL) was added potassium carbonate (15 mmol, 3.0 equiv). The resultant mixture was heated at reflux for 24 h. The mixture was then cooled to room temperature and filtered through a thin plug of Celite® eluted with CH_2Cl_2. The filtrate was washed with aqueous NaOH solution (2 M, 5 mL × 3) and brine (5 mL). The organic layer was dried over anhydrous Na_2SO_4, filtered, and concentrated under reduced pressure. The residue was purified over silica gel column chromatography (EtOAc:hexane, 1:40 to 1:20) to afford the desired 1,4-bisalkoxybenzene **S1**.

*1,4-diisobutoxybenzene (**S1a**)*

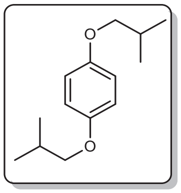

Yield: 86%
Physical state: white solid
R_f = 0.4 (1:20 EtOAc: hexane)
^{1}H NMR (500 MHz, $CDCl_3$) δ: 6.82 (s, 4H), 3.67 (d, *J* = 6.5 Hz, 4H), 2.05 (dp, *J* = 13.2, 6.5 Hz, 2H), 1.01 (d, *J* = 6.7 Hz, 12H).
^{13}C NMR (126 MHz, $CDCl_3$) δ: 153.47, 115.53, 77.41, 77.16, 76.91, 75.32, 28.48, 19.44.
HRMS (APCI) calcd for $C_{14}H_{22}O_2$ $[M + H]^+$: 222.16143, found: 222.16148.

*1,4-bis(3-phenylpropoxy)benzene (**S1b**)*

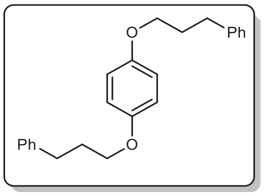

Yield: 91%
Physical state: white solid
R_f = 0.6 (1:20 EtOAc: hexane)
^{1}H NMR (500 MHz, $CDCl_3$) δ: 7.30 (t, *J* = 7.8 Hz, 4H), 7.21 (dd, *J* = 13.2, 7.8 Hz, 6H), 6.83 (s, 4H), 3.92 (t, *J* = 6.2 Hz, 4H), 2.81 (t, *J* = 7.7 Hz, 4H), 2.09 (p, *J* = 6.5 Hz, 4H).
^{13}C NMR (126 MHz, $CDCl_3$) δ: 153.28, 141.76, 128.66, 128.53, 126.03, 115.58, 77.41, 77.16, 76.91, 67.67, 32.32, 31.10.
HRMS (APCI) calcd for $C_{24}H_{26}O_2$ $[M + H]^+$: 369.18250, found: 369.18236.

*1,4-bis(isoamyloxy)benzene (**S1c**)*

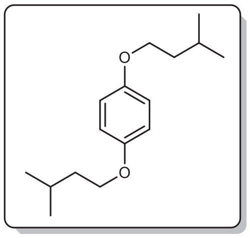

Yield: 90%
Physical state: white solid
R_f = 0.6 (1:20 EtOAc: hexane)
^{1}H NMR (500 MHz, $CDCl_3$) δ: 6.82 (s, 4H), 3.93 (t, *J* = 6.7 Hz, 4H), 1.82 (dp, *J* = 13.3, 6.6 Hz, 2H), 1.65 (q, *J* = 6.7 Hz, 4H), 0.95 (d, *J* = 6.6 Hz, 12H).
^{13}C NMR (126 MHz, $CDCl_3$) δ: 153.32, 115.51, 77.41, 77.16, 76.91, 67.14, 38.28, 25.19, 22.76.
HRMS (APCI) calcd for $C_{16}H_{26}O_2$ $[M + H]^+$: 250.19273, found: 250.19262.

*1,4-bis(hexyloxy)benzene (**13**)*

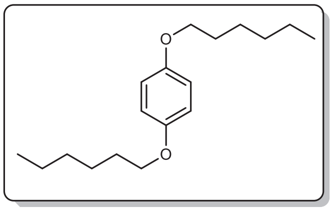

Yield: 89%
Physical state: white solid
R_f = 0.7 (1:20 EtOAc: hexane)
^{1}H NMR (500 MHz, $CDCl_3$) δ: 6.87 (s, 4H), 3.94 (t, *J* = 6.6 Hz, 4H), 1.81 (dt, *J* = 14.6, 6.6 Hz, 4H), 1.52 (p, *J* = 7.2 Hz, 4H), 1.41 (dt, *J* = 7.3, 3.7 Hz, 8H), 0.99 (t, *J* = 7.2 Hz, 6H).
^{13}C NMR (126 MHz, $CDCl_3$) δ: 153.30, 115.34, 68.56, 31.77, 29.52, 25.89, 22.74, 14.10.
HRMS (APCI) calcd for $C_{18}H_{30}O_2$ $[M + H]^+$: 279.2319, found: 279.2326.

Representative procedure for the preparation of pillar[5]arene **PA**

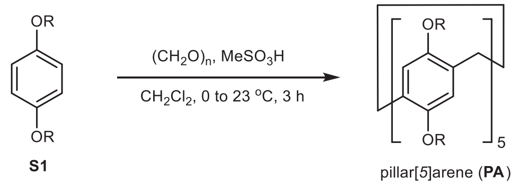

Pillar[5]arene **PA** was prepared according to a modified literature procedure.[10] To a stirred solution of 1,4-bisalkoxybenzene **S1** (5 mmol, 1.0 equiv.) and grinded polyformaldehyde (10 mmol, 2.0 equiv) in CH_2Cl_2 (40 mL) was added methanesulfonic acid (15 mmol, 3.0 equiv) in one portion under N_2 atmosphere at 0 °C. The resultant mixture was warmed to 23 °C and stirred for 3 h. Saturated aqueous $NaHCO_3$ (50 mL) was added, and the mixture was stirred (c.a. 30 min) until the organic layer changed to pale yellow-green. The organic phase was separated, and the aqueous phase was extracted with CH_2Cl_2 (20 mL × 3). The combined organic phase was then washed with brine (20 mL × 3), dried over anhydrous Na_2SO_4, filtered, and concentrated under reduced

pressure. The residue was purified over silica gel column chromatography (CH_2Cl_2: hexane, 1:20 to 1:3) to afford the desired pillar[5]arene **PA**.

*Dimethoxypillar[5]arene (**PA1**)*

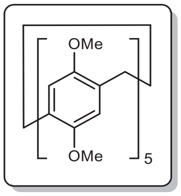

This compound was synthesized based on the literature method.[11]
Yield: 70%
Physical state: white solid
R_f = 0.6 (1:3 CH_2Cl_2: hexane)
^{1}H NMR (500 MHz, $CDCl_3$) δ: 6.88 (s, 10H), 3.76 (s, 10H), 3.74 (s, 30H).
^{13}C NMR (126 MHz, $CDCl_3$) δ: 150.41, 128.28, 113.32, 55.48, 29.33.
HRMS (ESI) calcd for $C_{45}H_{50}O_{10}$ $[M + Na]^+$: 773.3291, found: 773.3296.

*Diisobutoxypillar[5]arene (**PA2**)*

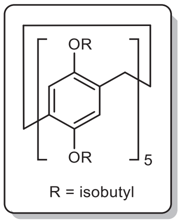

Yield: 39%
Physical state: white solid
R_f = 0.5 (1:4 CH_2Cl_2: hexane)
^{1}H NMR (500 MHz, $CDCl_3$) δ: 6.84 (s, 10H), 3.86 – 3.68 (m, 20H), 3.60 – 3.42 (m, 10H), 2.09 (dp, J = 13.3, 6.6 Hz, 10H), 1.05 (dd, J = 9.2, 6.8 Hz, 60H).
^{13}C NMR (126 MHz, $CDCl_3$) δ: 149.98, 128.31, 115.10, 75.19, 29.53, 28.81, 19.77, 19.62.
HRMS (ESI) calcd for $C_{75}H_{110}O_{10}$ $[M + H]^+$: 1193.79912, found: 1193.79822.

*Di(3-phenylpropoxy)pillar[5]arene (**PA3**)*

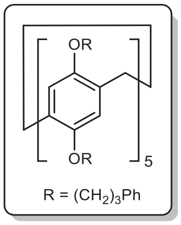

Yield: 51%
Physical state: white solid
R_f = 0.6 (1:5 CH_2Cl_2: hexane)
^{1}H NMR (500 MHz, $CDCl_3$) δ: 7.27 (m, 20H), 7.23 – 7.15 (m, 30H), 6.91 (s, 10H), 3.98 (dt, J = 8.8, 6.0 Hz, 10H), 3.84 (s, 10H), 3.76 (dt, J = 8.7, 6.5 Hz, 10H), 2.79 (t, J = 7.8 Hz, 20H), 2.04 (dhept, J = 13.6, 6.4 Hz, 20H).

^{13}C NMR (126 MHz, $CDCl_3$) δ: 150.03, 141.95, 128.57, 128.52, 128.45, 126.04, 115.15, 67.82, 32.71, 31.86, 31.69.
HRMS (ESI) calcd for $C_{125}H_{130}O_{10}$ $[M + H]^+$: 1814.95901, found: 1814.95814.

*Diisoamyloxypillar[5]arene (**PA4**)*

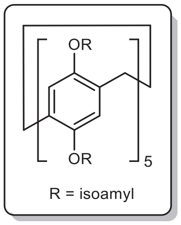

Yield: 50%
Physical state: white solid
R_f = 0.7 (1:4 CH_2Cl_2: hexane)
^{1}H NMR (500 MHz, $CDCl_3$) δ: 6.85 (s, 10H), 3.99 (d, *J* = 6.4 Hz, 10H), 3.77 (d, *J* = 21.5 Hz, 20H), 1.92 (dp, *J* = 13.4, 6.7 Hz, 10H), 1.76 (dt, *J* = 13.3, 6.5 Hz, 10H), 1.67 (dq, *J* = 14.9, 9.7, 7.9 Hz, 10H), 0.99 (t, *J* = 6.9 Hz, 60H).
^{13}C NMR (126 MHz, $CDCl_3$) δ: 149.99, 128.30, 114.95, 66.81, 38.98, 25.42, 22.79.
HRMS (APCI) calcd for $C_{85}H_{130}O_{10}$ $[M + H]^+$: 1333.95562, found: 1333.95518.

*Dihexoxypillar[5]arene (**PA5**)*

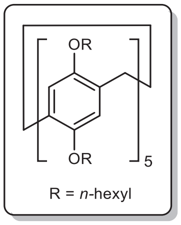

Yield: 58%
Physical state: white solid
R_f = 0.4 (1:4 CH_2Cl_2: hexane)
^{1}H NMR (500 MHz, $CDCl_3$) δ: 6.84 (s, 10H), 3.85 (t, 20H), 3.76 (s, 10H), 1.81 (dt, *J* = 14.7, 6.6 Hz, 20H), 1.53 (p, *J* = 7.1 Hz, 20H), 1.35 (td, *J* = 7.4, 3.5 Hz, 40H), 1.04 – 0.84 (m, 30H).
^{13}C NMR (126 MHz, $CDCl_3$) δ: 150.00, 128.31, 114.97, 68.51, 31.95, 30.05, 29.51, 26.20, 22.77, 14.21.
HRMS (ESI) calcd for $C_{95}H_{150}O_{10}$ $[M + H]^+$: 1453.1336, found: 1453.1321.

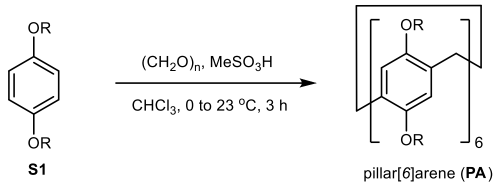

Representative procedure for the preparation of pillar[6]arene **PA**

Pillar[6]arene **PA** was prepared according to a modified literature procedure.[10] To a stirred solution of 1,4-bisalkoxybenzene **S1** (5 mmol, 1.0 equiv.) and grinded polyformaldehyde (10 mmol, 2.0 equiv) in chloroform (40 mL) was added methanesulfonic acid (15 mmol, 3.0 equiv) in one portion under N_2 atmosphere at 0 °C. The resultant mixture was warmed to 23 °C and stirred for 3 h. Saturated aqueous $NaHCO_3$ (50 mL) was added, and the mixture was stirred (c.a. 30 min) until the organic layer changed to pale yellow-green. The organic phase was separated, and the aqueous phase was extracted with $CHCl_3$ (20 mL × 3). The combined organic phase was then washed with brine (20 mL × 3), dried over anhydrous Na_2SO_4, filtered, and concentrated under reduced pressure. The residue was washed with acetone (10 mL × 3) to afford the desired pillar[6]arene product **PA**.

*Dihexoxypillar[6]arene (**PA6**)*

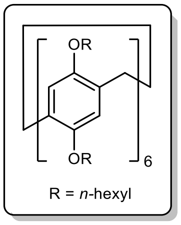

Yield: 60%
Physical state: white solid
R_f = 0.5 (1:4 EtOAc: hexane)
^{1}H NMR (500 MHz, $CDCl_3$) δ: 6.67 (s, 12H), 3.86 (s, 12H), 3.80 (t, *J* = 6.6 Hz, 24H), 1.70 (dt, *J* = 14.2, 6.5 Hz, 24H), 1.46 – 1.35 (m, 24H), 1.34 – 1.19 (m, 48H), 0.87 (t, *J* = 6.7 Hz, 36H).
^{13}C NMR (126 MHz, $CDCl_3$) δ: 150.82, 128.15, 114.83, 68.99, 31.86, 29.82, 26.04, 22.77, 14.19.
HRMS (ESI) calcd for $C_{114}H_{180}O_{12}$ $[M + H]^+$: 1765.34011, found: 1765.33953.

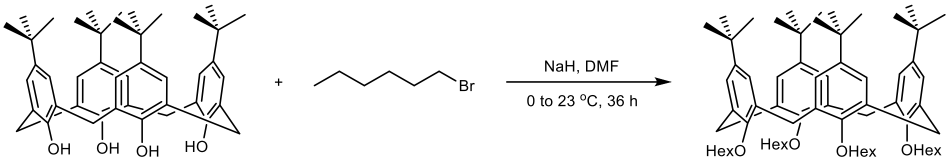

CA

To a stirred solution of *para*-*tert*-butylcalix[4]arene (5 mmol, 1.0 equiv) and NaH (30 mmol, 6.0 equiv.) in DMF (10 mL) was added 1-bromohexane (5.5 mmol, 5.5 equiv) at 0 °C. The resultant mixture was warmed to 23 °C and stirred for 36 h. Then, water (5 mL) was added to quench the reaction. The mixture was extracted by CH_2Cl_2 (5 mL × 3). The combined organic phase was washed with water (5 mL) and brine (5 mL), dried over anhydrous Na_2SO_4, filtered, and concentrated under reduced pressure. The residue was purified over silica gel column chromatography (EtOAc:hexanes, 1:40 to 1:10) to afford the desired product **CA**.

1^5,3^5,5^5,7^5-tetra-tert-butyl-1^2,3^2,5^2,7^2-tetrakis(hexyloxy)-1,3,5,7(1,3)-tetrabenzenacyclooctaphane (**CA**)

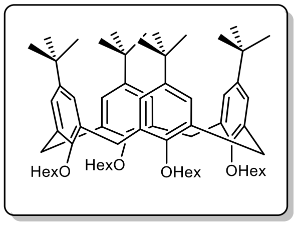

Yield: 78%
Physical state: white solid
R_f = 0.6 (1:20 EtOAc: hexane)
^{1}H NMR (500 MHz, $CDCl_3$) δ: 6.78 (s, 8H), 4.41 (d, *J* = 12.4 Hz, 4H), 3.99 – 3.72 (m, 8H), 3.11 (d, *J* = 12.5 Hz, 4H), 2.08 – 1.96 (m, 8H), 1.38 (m, 24H), 1.08 (s, 36H), 1.01 – 0.83 (m, 12H).
^{13}C NMR (126 MHz, $CDCl_3$) δ: 153.91, 144.25, 134.01, 124.98, 75.60, 33.94, 32.35, 31.61, 31.22, 30.49, 26.12, 23.07, 14.30.
HRMS (APCI) calcd for $C_{68}H_{104}O_4$ $[M + H]^+$: 985.8007, found: 985.8053.

6. Substrate preparation

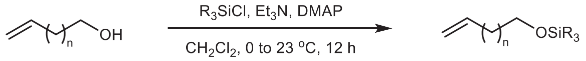

To a stirred solution of trialkylsilyl chloride R_3SiCl (5.5 mmol, 1.1 equiv), Et_3N (6 mmol, 1.2 equiv), and DMAP (0.5 mmol, 10 mol%) in CH_2Cl_2 (7.5 mL) was added hex-5-en-1-ol (5 mmol, 1.0 equiv) slowly at 0 ºC. The reaction was warmed to 23 ºC and stirred for 12 h. Then, a saturated aqueous $NaHCO_3$ solution was added to quench the reaction. The organic phase was separated, and the aqueous phase was extracted with CH_2Cl_2 (5 mL × 3). The combined organic phase was washed with brine (5 mL), dried over anhydrous Na_2SO_4, filtered, and concentrated under reduced pressure. The residue was purified over silica gel column chromatography (EtOAc:hexane, 1:30 to 1:20) to afford the desired product silyl ether product. (Allyloxy)trimethylsilane (**1r**) was commercially available.

*(hex-5-en-1-yloxy)trimethylsilane (**1a**)*

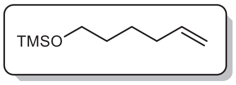

Yield: 94%
Physical state: colorless oil
R_f = 0.6 (1:20 EtOAc: hexane)
^{1}H NMR (500 MHz, $CDCl_3$) δ: 5.81 (ddt, *J* = 16.9, 10.2, 6.7 Hz, 1H), 5.22 – 4.67 (m, 2H), 3.58 (t, *J* = 6.6 Hz, 2H), 2.11 – 2.01 (m, 2H), 1.59 – 1.50 (m, 2H), 1.47 – 1.38 (m, 2H), 0.11 (s, 9H).
^{13}C NMR (126 MHz, $CDCl_3$) δ: 139.01, 114.57, 62.64, 33.68, 32.30, 25.29, -0.32.
HRMS (APCI) calcd for $C_9H_{20}OSi$ $[M + H]^+$: 173.1356, found: 173.1364.

*(dec-9-en-1-yloxy)triisopropylsilane (**1m**)*

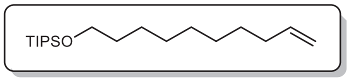

Yield: 96%
Physical state: colorless oil
R_f = 0.7 (1:20 EtOAc: hexane)
^{1}H NMR (500 MHz, $CDCl_3$) δ: 5.90 – 5.72 (m, 1H), 5.08 – 4.82 (m, 2H), 3.66 (t, *J* = 6.7 Hz, 2H), 2.08 – 1.99 (m, 2H), 1.56 – 1.50 (m, 3H), 1.41 – 1.28 (m, 12H), 1.06 (s, 18H).
^{13}C NMR (126 MHz, $CDCl_3$) δ: 139.41, 114.24, 63.65, 33.97, 33.19, 29.63, 29.57, 29.24, 29.07, 25.95, 18.19, 12.17.
HRMS (ESI) calcd for $C_{19}H_{40}OSi$ $[M + H]^+$: 313.29212, found: 313.29204.

*trimethyl(pent-4-en-1-yloxy)silane (**1p**)*

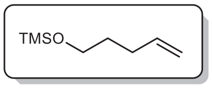

Yield: 40%
Physical state: colorless oil
R_f = 0.4 (1:40 EtOAc: hexane)
^{1}H NMR (400 MHz, $CDCl_3$) δ: 5.82 (ddt, *J* = 16.9, 10.2, 6.6 Hz, 1H), 4.99 – 4.91 (m, 2H), 3.59 (t, *J* = 6.6 Hz, 2H), 2.15 – 2.04 (m, 2H), 1.63 (dq, *J* = 8.4, 6.7 Hz, 2H), 0.11 (s, 9H).

^{13}C NMR (100 MHz, $CDCl_3$) δ: 138.56, 114.73, 62.14, 31.93, 30.16, -0.33.
HRMS (ESI) calcd for $C_{18}H_{18}OSi$ $[M + H]^+$: 159.11268, found: 159.11026.

*(but-3-en-1-yloxy)trimethylsilane (**1q**)*

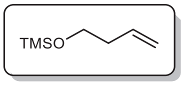

Yield: 80%
Physical state: colorless oil
R_f = 0.5 (1:20 EtOAc: hexane)
^{1}H NMR (500 MHz, $CDCl_3$) δ: 5.80 (ddt, *J* = 17.1, 10.2, 6.8 Hz, 1H), 5.15 – 4.95 (m, 2H), 3.62 (t, *J* = 6.9 Hz, 2H), 2.29 (qt, *J* = 6.9, 1.3 Hz, 2H), 0.11 (s, 9H).
^{13}C NMR (126 MHz, $CDCl_3$) δ: 135.37, 116.58, 62.33, 37.39, -0.32.
HRMS (APCI) calcd for $C_7H_{16}OSi$ $[M + H]^+$: 145.10432, found: 145.10433.

*(hex-5-en-1-yloxy)triisopropylsilane (**1s**)*

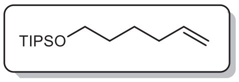

Yield: 84%
Physical state: colorless oil
R_f = 0.5 (1:30 EtOAc: hexane)
^{1}H NMR (500 MHz, $CDCl_3$) δ: 5.82 (ddt, *J* = 16.9, 10.2, 6.7 Hz, 1H), 5.16 – 4.74 (m, 2H), 3.68 (t, *J* = 6.5 Hz, 2H), 2.22 – 1.99 (m, 2H), 1.69 – 1.51 (m, 2H), 1.50 – 1.40 (m, 2H), 1.19 – 0.99 (m, 21H).
^{13}C NMR (126 MHz, $CDCl_3$) δ: 139.19, 114.45, 63.41, 33.73, 32.62, 25.33, 18.18, 12.15.
HRMS (APCI) calcd for $C_{15}H_{32}OSi$ $[M + H]^+$: 257.2295, found: 257.2293.

*trimethyl(pent-4-en-2-yloxy)silane (**12a**)*

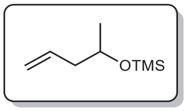

Yield: 77%
Physical state: colorless oil
R_f = 0.5 (1:20 EtOAc: hexane)
^{1}H NMR (500 MHz, $CDCl_3$) δ: 5.79 (ddt, *J* = 17.2, 10.2, 7.2 Hz, 1H), 5.10 – 4.94 (m, 2H), 3.82 (h, *J* = 6.1 Hz, 1H), 2.32 – 2.07 (m, 2H), 1.14 (d, *J* = 6.1 Hz, 3H), 0.11 (s, 9H).
^{13}C NMR (126 MHz, $CDCl_3$) δ: 135.69, 116.75, 68.47, 44.27, 23.60, 0.34.
HRMS (APCI) calcd for $C_8H_{19}OSi$ $[M + H]^+$: 159.1205, found: 159.1212.

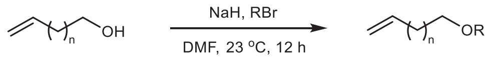

To a stirred solution of hex-5-en-1-ol (5.0 mmol, 1.0 equiv.) in DMF (10 mL) was added sodium hydride (6 mmol, 1.2 equiv.). The solution was stirred for 30 min and alkyl bromide RBr (5.5 mmol, 1.1 equiv.) was added. The reaction was stirred at 23 °C for 12 h. Then, water was added to quench the reaction. The mixture was extracted by diethyl ether (5 mL × 3). The combined organic phase was washed with water (5 mL) and brine (5 mL), dried over anhydrous Na_2SO_4, filtered, and concentrated under reduced pressure. The residue was purified over silica gel column chromatography (EtOAc:hexanes, 1:50) to afford the desired product.

*6-(hexyloxy)hex-1-ene (**1c**)*

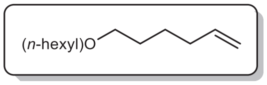

Yield: 96%
Physical state: colorless oil
R_f = 0.8 (1:20 EtOAc: hexane)
^{1}H NMR (500 MHz, $CDCl_3$) δ: 5.81 (ddt, J = 16.9, 10.2, 6.7 Hz, 1H), 5.21 – 4.83 (m, 2H), 3.56 – 3.32 (m, 4H), 2.24 – 1.96 (m, 2H), 1.57 (dtt, J = 13.1, 8.3, 6.5 Hz, 4H), 1.50 – 1.40 (m, 2H), 1.39 – 1.22 (m, 6H), 0.88 (t, J = 6.8 Hz, 3H).
^{13}C NMR (126 MHz, $CDCl_3$) δ: 138.98, 114.59, 71.14, 70.84, 33.73, 31.87, 29.89, 29.38, 26.02, 25.66, 22.78, 14.21.
HRMS (APCI) calcd for $C_{12}H_{24}O$ $[M + H]^+$: 185.1900, found: 185.1898.

*6-(isopentyloxy)hex-1-ene (**1d**)*

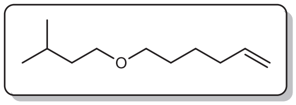

Yield: 79%
Physical state: colorless oil
R_f = 0.7 (1:20 EtOAc: hexane)
^{1}H NMR (500 MHz, $CDCl_3$) δ: 5.81 (ddt, J = 16.9, 10.2, 6.7 Hz, 1H), 5.06 – 4.88 (m, 2H), 3.41 (dt, J = 9.8, 6.7 Hz, 4H), 2.07 (q, J = 7.3 Hz, 2H), 1.69 (dp, J = 13.4, 6.7 Hz, 1H), 1.64 – 1.54 (m, 2H), 1.45 (dd, J = 13.3, 6.5 Hz, 2H), 0.90 (d, J = 6.7 Hz, 6H).
^{13}C NMR (126 MHz, $CDCl_3$) δ: 138.99, 114.59, 70.89, 69.44, 38.73, 33.73, 29.39, 25.66, 25.26, 22.80.
HRMS (APCI) calcd for $C_{11}H_{22}O$ $[M + H]^+$: 171.1743, found: 171.1742.

*(3-(hex-5-en-1-yloxy)propyl)benzene (**1e**)*

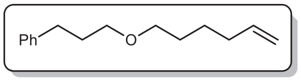

Yield: 88%
Physical state: colorless oil
R_f = 0.5 (1:30 EtOAc: hexane)

^{1}H NMR (500 MHz, $CDCl_3$) δ: 7.30-7.27 (dd, J = 8.2, 6.8 Hz, 2H), 7.20-7.17 (dd, J = 8.6, 7.3 Hz, 3H), 5.82 (ddt, J = 17.0, 10.2, 6.7 Hz, 1H), 5.11 – 4.90 (m, 2H), 3.51 – 3.33 (m, 4H), 2.69 (dd, J = 8.6, 6.8 Hz, 2H), 2.13 – 2.04 (m, 2H), 1.95 – 1.85 (m, 2H), 1.65 – 1.54 (m, 2H), 1.52 – 1.42 (m, 2H).
^{13}C NMR (126 MHz, $CDCl_3$) δ: 142.20, 138.95, 128.62, 128.44, 125.87, 114.64, 70.89, 70.05, 33.73, 32.50, 31.46, 29.38, 25.67.
HRMS (APCI) calcd for $C_{15}H_{22}O$ $[M + H]^+$: 241.1563, found: 241.1561.

*((hex-5-en-1-yloxy)methyl)cyclohexane (**1f**)*

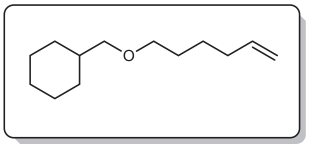

Yield: 84%
Physical state: colorless oil
R_f = 0.5 (1:20 EtOAc: hexane)
^{1}H NMR (400 MHz, $CDCl_3$) δ: 5.86 – 5.72 (m, 1H), 5.04 – 4.88 (m, 2H), 3.37 (td, J = 6.5, 1.7 Hz, 2H), 3.18 (dd, J = 6.6, 1.8 Hz, 2H), 2.09 – 2.01 (m, 2H), 1.81 – 1.65 (m, 5H), 1.59 – 1.51 (m, 3H), 1.48 – 1.36 (m, 2H), 1.27 – 1.15 (m, 3H), 0.95 – 0.83 (m, 2H).
^{13}C NMR (100 MHz, $CDCl_3$) δ: 207.12, 138.99, 114.53, 70.98, 38.16, 33.71, 31.06, 30.28, 29.32, 26.79, 26.01, 25.63.
HRMS (ESI) calcd for $C_{13}H_{24}O$ $[M + H]^+$: 197.18472, found: 197.18041.

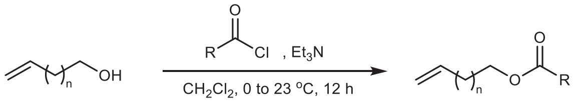

To a stirred solution of acyl chloride RCOCl (5.5 mmol, 1.1 equiv), Et_3N (6 mmol, 1.2 equiv), and DMAP (0.5 mmol, 10 mol%) in CH_2Cl_2 (7.5 mL) was added hex-5-en-1-ol (5 mmol, 1.0 equiv) slowly at 0 ºC. The reaction was warmed to 23 ºC and stirred for 12 h. Then, a saturated aqueous $NaHCO_3$ solution was added to quench the reaction. The organic phase was separated, and the aqueous phase was extracted by CH_2Cl_2 (5 mL × 3). The combined organic phase was washed with brine (5 mL), dried over anhydrous Na_2SO_4, filtered, and concentrated under reduced pressure. The residue was purified over silica gel column chromatography (EtOAc:hexane, 1:30) to afford the desired product ester product.

*hex-5-en-1-yl isobutyrate (**1g**)*

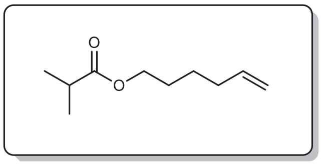

Yield: 78%
Physical state: colorless oil
R_f = 0.7 (1:20 EtOAc: hexane)
^{1}H NMR (500 MHz, $CDCl_3$) δ: 5.85 – 5.73 (m, 1H), 5.05 – 4.92 (m, 2H), 4.06 (t, J = 6.6 Hz, 2H), 2.59 – 2.47 (m, 1H), 2.12 – 2.02 (m, 2H), 1.68 – 1.57 (m, 2H), 1.54 – 1.40 (m, 2H), 1.16 (s, 3H), 1.15 (s, 3H).

^{13}C NMR (126 MHz, $CDCl_3$) δ: 177.39, 138.52, 114.92, 64.29, 34.17, 33.42, 28.22, 25.32, 19.14.
HRMS (ESI) calcd for $C_{10}H_{18}O_2$ $[M + Na]^+$: 193.11990, found: 193.11985.

*hex-5-en-1-yl cyclohexanecarboxylate (**1h**)*

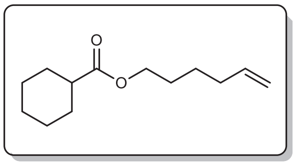

Yield: 45%
Physical state: colorless oil
R_f = 0.7 (1:20 EtOAc: hexane)
^{1}H NMR (400 MHz, $CDCl_3$) δ: 5.79 (m, 1H), 5.06 – 4.92 (m, 2H), 4.05 (t, *J* = 6.6 Hz, 2H), 2.34 – 2.22 (m, 1H), 2.08 (q, *J* = 7.2 Hz, 2H), 1.89 – 1.87 (m, 2H), 1.80 – 1.70 (m, 2H), 1.66 – 1.63 (m, 2H), 1.59 – 1.44 (m, 4H), 1.36 – 1.14 (m, 4H).
^{13}C NMR (126 MHz, $CDCl_3$) δ: 176.37, 138.51, 114.90, 64.14, 43.39, 33.41, 29.16, 28.22, 25.89, 25.58, 25.32.
HRMS (ESI) calcd for $C_{13}H_{22}O_2$ $[M + Na]^+$: 233.15120, found: 233.15122.

*hex-5-en-1-yl pivalate (**1i**)*

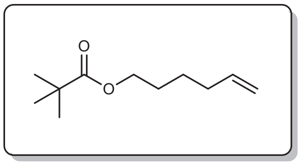

Yield: 50%
Physical state: colorless oil
R_f = 0.6 (1:20 EtOAc: hexane)
^{1}H NMR (500 MHz, $CDCl_3$) δ: 5.86 – 5.73 (m, 1H), 5.05 – 4.92 (m, 2H), 4.05 (t, *J* = 6.6 Hz, 2H), 2.12 – 2.02 (m, 2H), 1.68 – 1.56 (m, 2H), 1.54 – 1.40 (m, 2H), 1.19 (s, 9H).
^{13}C NMR (126 MHz, $CDCl_3$) δ: 178.77, 138.53, 114.90, 64.35, 33.40, 28.19, 27.32, 26.63, 25.34.
HRMS (ESI) calcd for $C_{11}H_{20}O_2$ $[M + Na]^+$: 207.13555, found: 207.13594.

*hex-5-en-1-yl 4-chlorobutanoate (**1j**)*

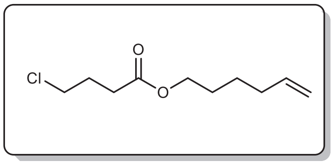

Yield: 53%
Physical state: colorless oil
R_f = 0.3 (1:20 EtOAc: hexane)
^{1}H NMR (400 MHz, $CDCl_3$) δ: 5.77 (ddt, *J* = 16.9, 10.2, 6.7 Hz, 1H), 5.05 – 4.91 (m, 2H), 4.07 (t, *J* = 6.7 Hz, 2H), 3.58 (t, *J* = 6.3 Hz, 2H), 2.48 (t, *J* = 7.3 Hz, 2H), 2.07 (tt, *J* = 7.4, 6.3 Hz, 4H), 1.69 – 1.57 (m, 2H), 1.50 – 1.37 (m, 2H).

^{13}C NMR (126 MHz, $CDCl_3$) δ: 172.76, 138.33, 114.92, 64.56, 44.15, 33.31, 31.27, 28.07, 27.72, 25.22.
HRMS (ESI) calcd for $C_{10}H_{17}ClO_2$ $[M + Na]^+$: 227.08093, found: 227.08109.

*hex-5-en-1-yl 4-bromobutanoate (**1k**)*

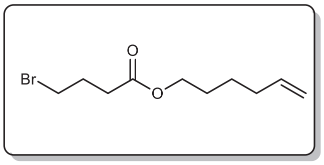

Yield: 30%
Physical state: colorless oil
$R_f = 0.3$ (1:20 EtOAc: hexane)
^{1}H NMR (500 MHz, $CDCl_3$) δ: 5.78 (ddtd, J = 16.9, 10.2, 6.7, 1.4 Hz, 1H), 5.06 – 4.91 (m, 2H), 4.12 – 4.03 (m, 2H), 3.49 – 3.41 (m, 2H), 2.53 – 2.44 (m, 2H), 2.21 – 2.12 (m, 2H), 2.10 – 2.04 (m, 2H), 1.69 – 1.57 (m, 2H), 1.50 – 1.39 (m, 2H).
^{13}C NMR (126 MHz, $CDCl_3$) δ: 172.77, 138.43, 115.02, 64.69, 33.39, 32.91, 32.60, 28.15, 27.89, 25.30.
HRMS (ESI) calcd for $C_{10}H_{17}BrO_2$ $[M + Na]^+$: 271.03041, found: 271.03038.

*non-8-en-2-one (**1l**)*

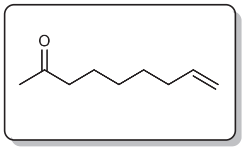

Yield: 90%
Physical state: colorless oil
$R_f = 0.6$ (1:20 EtOAc: hexane)
^{1}H NMR (500 MHz, $CDCl_3$) δ: 5.85 – 5.73 (m, 1H), 5.04 – 4.85 (m, 2H), 2.42 (t, J = 7.4 Hz, 2H), 2.13 (s, 3H), 2.09 – 2.00 (m, 2H), 1.58 (p, J = 7.5 Hzz, 2H), 1.44 – 1.35 (m, 2H), 1.29 (ddd, J = 15.5, 8.8, 5.9 Hz, 2H).
^{13}C NMR (100 MHz, $CDCl_3$) δ: 209.48, 138.97, 114.54, 43.85, 33.69, 30.01, 28.77, 28.74, 23.78.
HRMS (ESI) calcd for $C_9H_{16}O$ $[M + H]^+$: 141.12012, found: 141.12010.

*dec-9-en-1-yl pivalate (**1n**)*

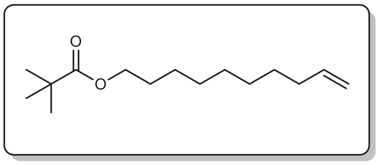

Yield: 95%
Physical state: colorless oil
$R_f = 0.6$ (1:20 EtOAc: hexane)
^{1}H NMR (500 MHz, $CDCl_3$) δ: 5.85 – 5.75 (m, 1H), 5.02 – 4.89 (m, 2H), 4.04 (t, J = 6.6 Hz, 2H), 2.07 – 1.99 (m, 2H), 1.66 – 1.56 (m, 2H), 1.41 – 1.28 (m, 10H), 1.19 (s, 9H).

^{13}**C NMR** (126 MHz, $CDCl_3$) δ: 178.80, 139.27, 114.29, 64.57, 33.92, 29.47, 29.29, 29.14, 29.01, 28.73, 27.33, 26.64, 26.01.
HRMS (ESI) calcd for $C_{15}H_{28}O_2$ $[M + Na]^+$: 263.19815, found: 263.19800.

*tert-butyl hex-5-en-1-ylcarbamate (**1o**)*

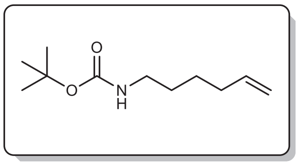

Yield: 77%
Physical state: colorless oil
R_f = 0.4 (1:20 EtOAc: hexane)
^{1}**H NMR** (500 MHz, $CDCl_3$) δ: 5.85 – 5.73 (m, 1H), 5.04 – 4.92 (m, 2H), 3.11 (s, 1H), 2.11 – 2.02 (m, 2H), 1.44 (s, 15H).
^{13}**C NMR** (126 MHz, $CDCl_3$) δ: 155.98, 138.54, 114.70, 33.36, 29.53, 28.44, 26.05.
HRMS (ESI) calcd for $C_{11}H_{21}NO_2$ $[M + Na]^+$: 222.14645, found: 222.14620.

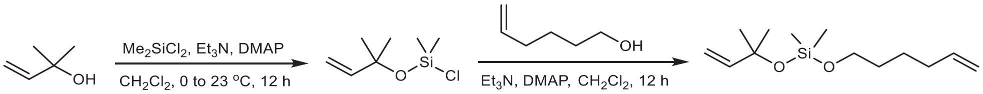

10a

To a stirred solution of dimethylsilyl dichloride Me_2SiCl_2 (5.5 mmol, 1.1 equiv), Et_3N (6 mmol, 1.2 equiv) and DMAP (0.5 mmol, 10 mol%) in CH_2Cl_2 (7.5 mL) was added 2-methylbut-3-en-2-ol (5 mmol, 1.0 equiv) dropwise at 0 ºC. The reaction was warmed to 23 ºC and stirred for 12 h. Then, the reaction was cooled to 0 ºC and a solution of hex-5-en-1-ol (5 mmol, 1.0 equiv) in CH_2Cl_2 (5 mL) was added dropwise. The resultant mixture was warmed to 23 ºC and stirred for 12 h. Then, a saturated aqueous $NaHCO_3$ was added to quench the reaction. The organic phase was separated and the aqueous phase was extracted by CH_2Cl_2 (5 mL × 3). The combined organic phase was then washed with brine (5 mL), dried over anhydrous Na_2SO_4, filtered, and concentrated under reduced pressure. The residue was purified over silica gel column chromatography (EtOAc:hexanes, 1:40) to afford the bis-olefinic silyl ether product **10a**.

*(hex-5-en-1-yloxy)dimethyl((2-methylbut-3-en-2-yl)oxy)silane (**10a**)*

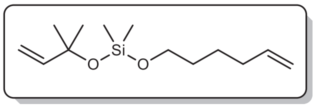

Yield: 64%
Physical state: colorless oil
R_f = 0.7 (1:20 EtOAc: hexane)
^{1}**H NMR** (500 MHz, $CDCl_3$) δ: 5.97 (dd, J = 17.3, 10.6 Hz, 1H), 5.81 (ddt, J = 16.9, 10.2, 6.6 Hz, 1H), 5.30 – 4.84 (m, 4H), 3.66 (t, J = 6.7 Hz, 2H), 2.07 (tdt, J = 7.8, 6.6, 1.4 Hz, 2H), 1.74 – 1.52 (m, 2H), 1.47-1.40 (m, 2H), 1.35 (s, 6H), 0.12 (s, 6H).
^{13}**C NMR** (126 MHz, $CDCl_3$) δ: 146.50, 139.04, 114.55, 110.73, 74.10, 62.35, 33.68, 32.13, 30.09, 25.32, -0.29.
HRMS (APCI) calcd for $C_{13}H_{26}O_2Si$ $[M + Na]^+$: 265.1594, found: 265.1592.

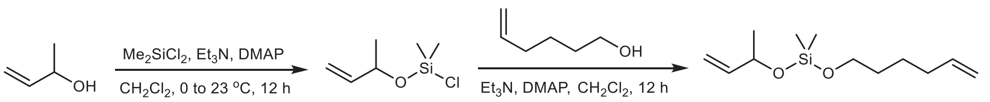

10b

To a stirred solution of dimethylsilyl dichloride Me_2SiCl_2 (5.5 mmol, 1.1 equiv.), TEA (6 mmol, 1.2 equiv.) and DMAP (0.5 mmol, 10 mol%) in CH_2Cl_2 (7.5 mL) was added pent-4-en-2-ol (5 mmol, 1.0 equiv.) dropwise at 0 ºC. The reaction was warmed to 23 ºC and stirred for 12 h. Then, the reaction was cooled to 0 ºC and a solution of hex-5-en-1-ol (5 mmol, 1.0 equiv.) in CH_2Cl_2 (5 mL) was added dropwise. The reaction was warmed to 23 ºC and stirred for 12 h. Then, saturated aqueous $NaHCO_3$ solution was added to quench the reaction. The organic phase was separated and the aqueous phase was extracted by CH_2Cl_2 (5 mL × 3). The combined organic phase was washed with brine (5 mL), dried over anhydrous Na_2SO_4, filtered, and concentrated under reduced pressure. The residue was purified over silica gel column chromatography (EtOAc:hexanes, 1:40) to afford the desired product.

*(hex-5-en-1-yloxy)dimethyl(pent-4-en-2-yloxy)silane (**10b**)*

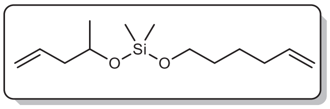

Yield: 62%
Physical state: colorless oil
R_f = 0.7 (1:20 EtOAc: hexane)
^{1}H NMR (500 MHz, $CDCl_3$) δ: 5.8 – 5.77 (m, 2H), 5.15 – 4.79 (m, 4H), 3.97 (h, J = 6.1 Hz, 1H), 3.67 (t, J = 6.6 Hz, 2H), 2.22 (ddt, J = 45.1, 13.6, 7.0 Hz, 2H), 2.07 (q, J = 7.2 Hz, 2H), 1.59 – 1.52 (m, 2H), 1.43 (p, J = 7.5 Hz, 2H), 1.17 (d, J = 6.1 Hz, 3H), 0.12 (s, 6H).
^{13}C NMR (126 MHz, $CDCl_3$) δ: 138.96, 135.45, 116.92, 114.59, 68.29, 62.49, 44.14, 33.66, 32.16, 25.26, 23.40, -2.49.
HRMS (APCI) calcd for $C_{13}H_{26}O_2Si$ $[M + Na]^+$: 265.15943, found: 265.15913.

7. Anti-Markovnikov intermolecular bromoesterification

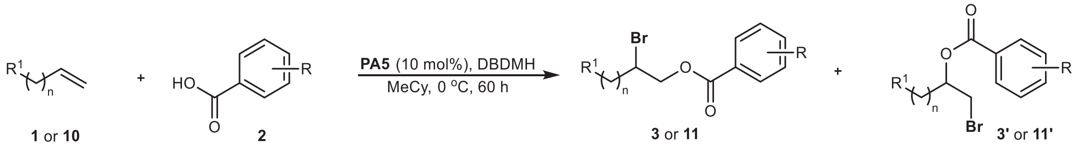

To a stirred solution of carboxylic acid **2** (0.1 mmol, 1 equiv), catalyst **PA5** (0.01 mmol, 10 mol%) in methylcyclohexane (0.35 mL) was added olefinic substrate **1** or **10** (0.1 mmol, 1 equiv). The solution was stirred at 0 ºC and DBDMH (0.1 mmol, 1 equiv) was added. The resultant mixture was stirred for 60 h at the same temperature and quenched with a saturated aqueous Na_2SO_3 solution. The organic phase was separated, and the aqueous phase was extracted with CH_2Cl_2 (2 mL × 3). The combined organic phase was washed with brine (3 mL), dried over anhydrous Na_2SO_4, filtered, and concentrated under reduced pressure. The residue was purified over silica gel column chromatography (EtOAc:hexanes, 1:100 to 1:20) to afford the desired anti-Markovnikov halogenation product **3** or **11**.

*2-bromo-6-((trimethylsilyl)oxy)hexyl benzoate (**3aa**)*

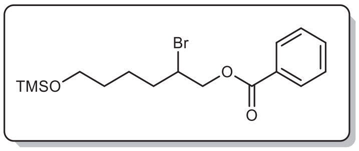

Yield: 82%
Physical state: pale-yellow oil
R_f = 0.3 (1:20 EtOAc: hexane)
^{1}H NMR (500 MHz, $CDCl_3$) δ: 8.07 (dd, *J* = 8.3, 1.2 Hz, 2H), 7.58 (t, *J* = 7.4 Hz, 1H), 7.46 (t, *J* = 7.8 Hz, 2H), 4.75 – 4.46 (m, 2H), 4.37 – 4.17 (m, 1H), 3.60 (t, *J* = 6.2 Hz, 2H), 1.99 (dddd, *J* = 14.1, 9.6, 5.3, 4.2 Hz, 1H), 1.90 (dtd, *J* = 14.4, 9.4, 4.8 Hz, 1H), 1.72 – 1.63 (m, 1H), 1.63 – 1.47 (m, 3H), 0.11 (s, 9H).
^{13}C NMR (126 MHz, $CDCl_3$) δ: 166.15, 133.41, 129.88, 129.81, 128.61, 68.29, 62.33, 51.64, 35.25, 32.08, 23.88, -0.35.
HRMS (APCI) calcd for $C_{16}H_{25}BrO_3Si$ $[M + H]^+$: 375.0810, found: 375.0819.

*2-bromo-6-((trimethylsilyl)oxy)hexyl 2-fluorobenzoate (**3ab**)*

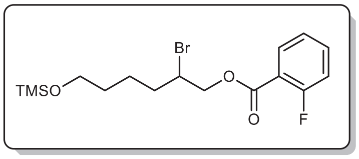

Yield: 76%
Physical state: pale-yellow oil
R_f = 0.3 (1:20 EtOAc: hexane)
^{1}H NMR (500 MHz, $CDCl_3$) δ: 7.96 (td, *J* = 7.5, 1.9 Hz, 1H), 7.67 – 7.43 (m, 1H), 7.22 (td, *J* = 7.6, 1.1 Hz, 1H), 7.15 (ddd, *J* = 10.8, 8.3, 1.1 Hz, 1H), 4.76 – 4.43 (m, 2H), 4.25 (dtd, *J* = 9.1, 6.1, 4.3 Hz, 1H), 3.59 (t, *J* = 6.2 Hz, 2H), 2.00 (dddd, *J* = 14.1, 9.6, 5.5, 4.2 Hz, 1H), 1.89 (dtd, *J* = 14.4, 9.5, 4.8 Hz, 1H), 1.67 (dtd, *J* = 11.0, 6.1, 2.8 Hz, 1H), 1.62 – 1.43 (m, 3H), 0.10 (s, 9H).
^{13}C NMR (126 MHz, $CDCl_3$) δ: 163.88, 163.25, 161.18, 134.93, 132.34, 124.17, 118.35, 117.30, 68.47, 62.31, 51.26, 35.14, 32.04, 23.79, -0.37.
HRMS (ESI) calcd for $C_{16}H_{24}BrFO_3Si$ $[M + Na]^+$: 413.05543, found: 413.05543.

*2-bromo-6-((trimethylsilyl)oxy)hexyl 3-fluorobenzoate (**3ac**)*

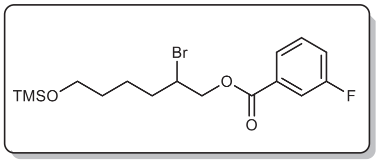

Yield: 77%
Physical state: pale-yellow oil
R_f = 0.3 (1:20 EtOAc: hexane)
^{1}H NMR (500 MHz, $CDCl_3$) δ: 7.86 (d, *J* = 7.8 Hz, 1H), 7.74 (d, *J* = 9.2 Hz, 1H), 7.44 (td, *J* = 8.0, 5.6 Hz, 1H), 7.29 (td, *J* = 8.5, 8.0, 1.9 Hz, 1H), 4.55 (d, *J* = 6.6 Hz, 2H), 4.25 (dq, *J* = 10.6, 6.0 Hz, 1H), 3.60 (t, *J* = 6.1 Hz, 2H), 2.03 – 1.82 (m, 2H), 1.73 – 1.63 (m, 1H), 1.56 – 1.49 (m, 3H), 0.11 (s, 9H).
^{13}C NMR (126 MHz, $CDCl_3$) δ: 165.04, 163.68, 161.71, 131.98, 130.32, 125.65, 120.61, 116.87, 68.60, 62.30, 51.40, 35.21, 32.05, 23.89, -0.36.
HRMS (ESI) calcd for $C_{16}H_{24}BrFO_3Si$ $[M + Na]^+$: 415.0536, found: 415.0530.

*2-bromo-6-((trimethylsilyl)oxy)hexyl 3-bromobenzoate (**3ad**)*

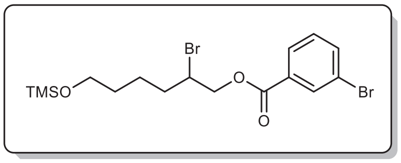

Yield: 71%
Physical state: pale-yellow oil
R_f = 0.3 (1:20 EtOAc: hexane)
^{1}H NMR (500 MHz, $CDCl_3$) δ: 8.18 (s, 1H), 7.99 (d, *J* = 7.8 Hz, 1H), 7.71 (d, *J* = 8.7 Hz, 1H), 7.34 (t, *J* = 7.9 Hz, 1H), 4.65 – 4.47 (m, 2H), 4.25 (dq, *J* = 10.7, 6.0 Hz, 1H), 3.60 (t, *J* = 6.1 Hz, 2H), 2.01 – 1.86 (m, 2H), 1.71 – 1.66 (m, 1H), 1.60 – 1.49 (m, 3H), 0.11 (s, 9H).
^{13}C NMR (126 MHz, $CDCl_3$) δ: 164.82, 136.37, 132.83, 131.72, 130.19, 128.46, 122.69, 68.64, 62.29, 51.38, 35.19, 32.04, 23.88, -0.35.
HRMS (ESI) calcd for $C_{16}H_{24}Br_2O_3Si$ $[M + Na]^+$: 474.97341, found: 474.97348.

*2-bromo-6-((trimethylsilyl)oxy)hexyl 3-methylbenzoate (**3ae**)*

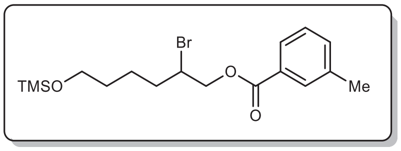

Yield: 73%
Physical state: pale-yellow oil
R_f = 0.3 (1:20 EtOAc: hexane)
^{1}H NMR (500 MHz, $CDCl_3$) δ: 7.86 (d, *J* = 7.5 Hz, 2H), 7.46 – 7.30 (m, 2H), 4.53 (h, *J* = 5.5 Hz, 2H), 4.26 (dq, *J* = 10.6, 5.8 Hz, 1H), 3.60 (t, *J* = 6.0 Hz, 2H), 2.41 (s, 3H), 1.94 (dtt, *J* = 40.5, 9.6, 4.9 Hz, 2H), 1.73 – 1.47 (m, 4H), 0.11 (s, 9H).
^{13}C NMR (126 MHz, $CDCl_3$) δ: 166.34, 138.43, 134.19, 130.39, 129.73, 128.51, 127.03, 68.25, 62.34, 51.71, 35.24, 32.09, 23.88, 21.45, -0.35.
HRMS (APCI) calcd for $C_{17}H_{27}BrO_3Si$ $[M + H]^+$: 389.09670, found: 389.09684.

*2-bromohexyl benzoate (**3ba**)*

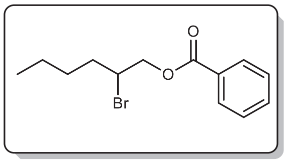

Yield: 90%
Physical state: pale-yellow oil
R_f = 0.5 (1:20 EtOAc: hexane)
^{1}H NMR (500 MHz, $CDCl_3$) δ: 8.34 – 7.91 (m, 2H), 7.67 – 7.53 (m, 1H), 7.53 – 7.38 (m, 2H), 4.78 – 4.35 (m, 2H), 4.32 – 4.20 (m, 1H), 2.04 – 1.83 (m, 2H), 1.67 – 1.53 (m, 1H), 1.51 – 1.29 (m, 3H), 0.93 (t, *J* = 7.3 Hz, 3H).
^{13}C NMR (126 MHz, $CDCl_3$) δ: 166.17, 133.40, 129.88, 129.85, 128.61, 68.35, 51.83, 35.16, 29.49, 22.23, 14.04.
HRMS (APCI) calcd for $C_{13}H_{17}BrO_2$ $[M -Br]^-$: 205.1223, found: 205.1231.

*2-bromohexyl 3-fluorobenzoate (**3bc**)*

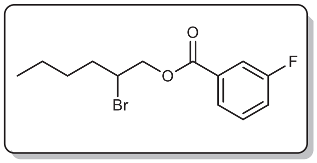

Yield: 92%
Physical state: colorless oil
R_f = 0.5 (1:20 EtOAc: hexane)
^{1}H NMR (500 MHz, $CDCl_3$) δ: 7.86 (d, *J* = 7.8 Hz, 1H), 7.74 (d, *J* = 8.4 Hz, 1H), 7.44 (td, *J* = 8.0, 5.5 Hz, 1H), 7.28 (t, *J* = 7.8 Hz, 1H), 4.55 (d, *J* = 6.0 Hz, 2H), 4.35 – 4.16 (m, 1H), 2.01 – 1.83 (m, 2H), 1.57 (dtd, *J* = 9.6, 5.5, 5.1, 1.8 Hz, 1H), 1.50 – 1.30 (m, 3H), 0.93 (t, *J* = 7.3 Hz, 3H).
^{13}C NMR (126 MHz, $CDCl_3$) δ: 165.05, 163.67, 161.70, 132.00, 130.25, 125.64, 120.58, 116.85, 68.64, 51.56, 35.11, 29.47, 22.21, 14.02.
HRMS (ESI) calcd for $C_{13}H_{16}BrFO_2$ $[M + Na]^+$: 325.0210, found: 325.0208.

*2-bromohexyl 3-methylbenzoate (**3be**)*

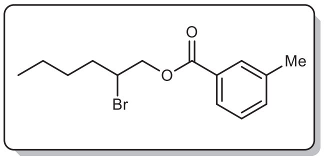

Yield: 93%
Physical state: colorless oil
R_f = 0.5 (1:20 EtOAc: hexane)
^{1}H NMR (500 MHz, $CDCl_3$) δ: 8.04 – 7.81 (m, 2H), 7.42 (d, *J* = 7.4 Hz, 1H), 7.37 (t, *J* = 7.9 Hz, 1H), 4.56 (dd, *J* = 6.1, 2.5 Hz, 2H), 4.28 (dq, *J* = 10.8, 6.0 Hz, 1H), 2.44 (s, 3H), 2.07 – 1.81 (m, 2H), 1.69 – 1.58 (m, 1H), 1.55 – 1.31 (m, 3H), 0.96 (t, *J* = 7.3 Hz, 3H).
^{13}C NMR (126 MHz, $CDCl_3$) δ: 166.34, 138.41, 134.16, 130.37, 129.75, 128.49, 127.00, 68.29, 51.87, 35.14, 29.47, 22.22, 21.43, 14.03.
HRMS (ESI) calcd for $C_{14}H_{19}BrO_2$ $[M + Na]^+$: 321.0461, found: 321.0459.

*2-bromohexyl 2-methylbenzoate (**3bf**)*

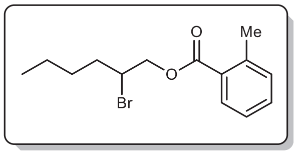

Yield: 99%
Physical state: colorless oil
R_f = 0.5 (1:20 EtOAc: hexane)
^{1}H NMR (500 MHz, $CDCl_3$) δ: 8.09 – 7.90 (m, 1H), 7.42 (td, *J* = 7.5, 1.5 Hz, 1H), 7.32 – 7.21 (m, 2H), 4.53 (d, *J* = 6.0 Hz, 2H), 4.26 (dtd, *J* = 8.8, 6.0, 4.6 Hz, 1H), 2.63 (s, 3H), 2.00 – 1.84 (m, 2H), 1.67 – 1.53 (m, 1H), 1.50 – 1.28 (m, 3H), 0.93 (t, *J* = 7.3 Hz, 3H).
^{13}C NMR (126 MHz, $CDCl_3$) δ: 167.06, 140.65, 132.43, 131.91, 130.96, 129.12, 125.94, 68.27, 51.92, 35.21, 29.49, 22.22, 22.05, 14.03.
HRMS (ESI) calcd for $C_{14}H_{19}BrO_2$ $[M + Na]^+$: 321.0461, found: 321.0460.

*2-bromo-6-(hexyloxy)hexyl benzoate (**3ca**)*

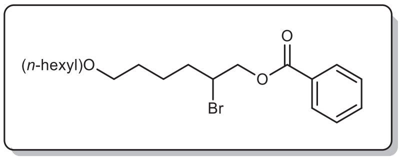

Yield: 98%
Physical state: colorless oil
R_f = 0.7 (1:20 EtOAc: hexane)
^{1}H NMR (500 MHz, $CDCl_3$) δ: 8.07 (d, *J* = 7.2 Hz, 2H), 7.58 (t, *J* = 7.4 Hz, 1H), 7.46 (t, *J* = 7.7 Hz, 2H), 4.66 – 4.48 (m, 2H), 4.34 – 4.21 (m, 2H), 3.40 (dt, *J* = 16.6, 6.5 Hz, 4H), 2.06 – 1.85 (m, 2H), 1.75 – 1.63 (m, 3H), 1.55 (dq, *J* = 8.9, 6.6 Hz, 3H), 1.38 – 1.21 (m, 6H), 0.88 (t, *J* = 6.9 Hz, 3H).
^{13}C NMR (126 MHz, $CDCl_3$) δ: 166.14, 133.41, 129.88, 129.81, 128.61, 71.24, 70.54, 68.28, 51.62, 35.28, 31.84, 29.84, 29.24, 26.01, 24.26, 22.77, 14.20.
HRMS (ESI) calcd for $C_{19}H_{29}BrO_3$ $[M + Na]^+$: 407.1192, found: 407.1189.

*2-bromo-6-(hexyloxy)hexyl 3-methylbenzoate (**3ce**)*

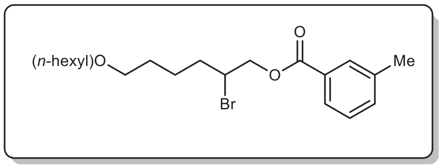

Yield: 88%
Physical state: colorless oil
R_f = 0.7 (1:20 EtOAc: hexane)
^{1}H NMR (500 MHz, $CDCl_3$) δ: 7.86 (d, *J* = 7.6 Hz, 2H), 7.45 – 7.30 (m, 2H), 4.68 – 4.47 (m, 2H), 4.32 – 4.21 (m, 1H), 3.40 (dt, *J* = 16.4, 6.4 Hz, 4H), 2.41 (s, 3H), 1.95 (ddtd, *J* = 51.3, 14.0, 9.5, 9.0, 4.8 Hz, 1H), 1.76 – 1.62 (m, 1H), 1.54 (m, 3H), 1.40 – 1.19 (m, 6H), 0.88 (t, *J* = 6.8 Hz, 3H).
^{13}C NMR (126 MHz, $CDCl_3$) δ: 166.33, 138.43, 134.19, 130.38, 129.73, 128.50, 127.02, 71.25, 70.55, 68.24, 51.67, 35.27, 31.85, 29.85, 29.25, 26.01, 24.25, 22.77, 21.44, 14.21.

HRMS (ESI) calcd for $C_{20}H_{31}BrO_3$ $[M + Na]^+$: 421.1349, found: 421.1345.

*2-bromo-6-(hexyloxy)hexyl 2-methylbenzoate (**3cf**)*

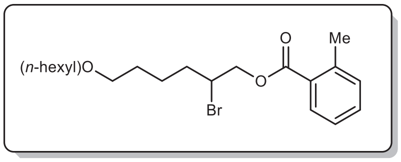

Yield: 74%
Physical state: colorless oil
R_f = 0.7 (1:20 EtOAc: hexane)
^{1}H NMR (500 MHz, $CDCl_3$) δ: 7.97 (d, J = 7.2 Hz, 1H), 7.42 (t, J = 7.5 Hz, 1H), 7.26 (d, J = 14.4 Hz, 2H), 4.53 (d, J = 6.0 Hz, 2H), 4.36 – 4.18 (m, 1H), 3.40 (dt, J = 15.6, 6.4 Hz, 4H), 2.62 (s, 3H), 1.95 (dtt, J = 35.4, 9.4, 4.7 Hz, 2H), 1.73 – 1.63 (m, 3H), 1.59 – 1.50 (m, 3H), 1.30 (tdd, J = 14.2, 6.6, 2.5 Hz, 6H), 0.88 (t, J = 6.8 Hz, 3H).
^{13}C NMR (126 MHz, $CDCl_3$) δ: 167.04, 140.67, 132.46, 131.92, 130.97, 129.09, 125.95, 71.25, 70.54, 68.21, 51.73, 35.34, 31.85, 29.85, 29.24, 26.01, 24.27, 22.77, 22.06, 14.21.
HRMS (ESI) calcd for $C_{20}H_{31}BrO_3$ $[M + Na]^+$: 421.1349, found: 421.1345.

*2-bromo-6-(hexyloxy)hexyl 2,6-difluorobenzoate (**3cg**)*

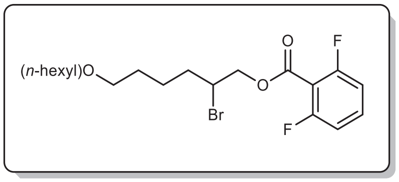

Yield: 68%
Physical state: colorless oil
R_f = 0.7 (1:20 EtOAc: hexane)
^{1}H NMR (500 MHz, $CDCl_3$) δ: 7.51 – 7.40 (m, 1H), 6.97 (t, J = 8.2 Hz, 2H), 4.58 (ddd, J = 51.1, 11.7, 6.2 Hz, 2H), 4.28 – 4.14 (m, 1H), 3.40 (dt, J = 13.7, 6.4 Hz, 4H), 2.01 (dddd, J = 14.1, 9.9, 5.8, 4.1 Hz, 1H), 1.94 – 1.80 (m, 1H), 1.71 – 1.62 (m, 3H), 1.57 – 1.46 (m, 3H), 1.39 – 1.11 (m, 6H), 0.88 (t, J = 6.9 Hz, 3H).
^{13}C NMR (126 MHz, $CDCl_3$) δ: 162.03, 161.14, 159.94, 133.28, 112.17, 71.23, 70.53, 68.93, 50.61, 35.02, 31.85, 29.85, 29.20, 26.01, 24.08, 22.77, 14.20.
HRMS (ESI) calcd for $C_{19}H_{27}BrF_2O_3$ $[M + Na]^+$: 443.1004, found: 443.1000.

*2-bromo-6-(isopentyloxy)hexyl benzoate (**3da**)*

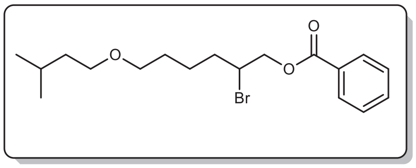

Yield: 90%
Physical state: colorless oil
R_f = 0.6 (1:20 EtOAc: hexane)
^{1}H NMR (500 MHz, $CDCl_3$) δ: 8.07 (d, J = 7.2 Hz, 2H), 7.58 (t, J = 7.4 Hz, 1H), 7.46 (t, J = 7.7 Hz, 2H), 4.72 – 4.46 (m, 2H), 4.26 (dq, J = 10.6, 6.0 Hz, 1H), 3.42 (t, J = 6.7 Hz, 4H), 2.05 –

1.82 (m, 2H), 1.74 – 1.63 (m, 3H), 1.58 – 1.50 (m, 2H), 1.45 (q, *J* = 6.9 Hz, 2H), 0.89 (d, *J* = 6.7 Hz, 6H).
^{13}C NMR (126 MHz, $CDCl_3$) δ: 166.14, 133.41, 129.88, 129.81, 128.61, 70.58, 69.55, 68.28, 51.62, 38.68, 35.28, 29.25, 25.25, 24.26, 22.79.
HRMS (ESI) calcd for $C_{18}H_{27}BrO_3$ $[M + Na]^+$: 393.1036, found: 393.1031.

*2-bromo-6-(isopentyloxy)hexyl 3-fluorobenzoate (**3dc**)*

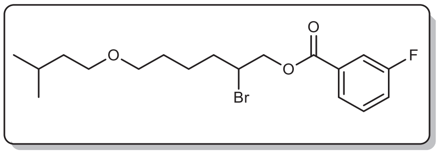

Yield: 75%
Physical state: colorless oil
R_f = 0.6 (1:20 EtOAc: hexane)
^{1}H NMR (500 MHz, $CDCl_3$) δ: 7.86 (d, *J* = 7.7 Hz, 1H), 7.73 (d, *J* = 9.2 Hz, 1H), 7.44 (td, *J* = 8.0, 5.6 Hz, 1H), 7.29 (dt, *J* = 8.2, 4.1 Hz, 1H), 4.55 (d, *J* = 5.9 Hz, 2H), 4.25 (dq, *J* = 10.6, 5.8 Hz, 1H), 3.42 (t, *J* = 6.6 Hz, 4H), 1.94 (dtt, *J* = 34.9, 9.6, 4.8 Hz, 2H), 1.75 – 1.64 (m, 3H), 1.60 – 1.50 (m, 2H), 1.45 (q, *J* = 6.9 Hz, 2H), 0.89 (d, *J* = 6.7 Hz, 6H).
^{13}C NMR (126 MHz, $CDCl_3$) δ: 165.04, 163.68, 161.71, 131.98, 130.32, 125.65, 120.60, 116.86, 70.56, 69.56, 68.59, 51.37, 38.67, 35.25, 29.23, 25.26, 24.27, 22.79.
HRMS (ESI) calcd for $C_{18}H_{26}BrFO_3$ $[M + Na]^+$: 411.0942, found: 411.0937.

*2-bromo-6-(3-phenylpropoxy)hexyl benzoate (**3e**)*

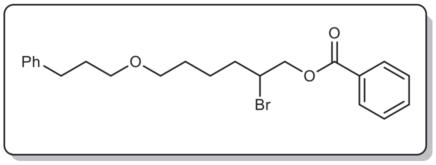

Yield: 73%
Physical state: colorless oil
R_f = 0.6 (1:30 EtOAc: hexane)
^{1}H NMR (500 MHz, $CDCl_3$) δ: 8.07 (d, *J* = 7.3 Hz, 2H), 7.58 (t, *J* = 7.4 Hz, 1H), 7.46 (t, *J* = 7.7 Hz, 2H), 7.37 – 7.25 (m, 3H), 7.17 (d, *J* = 9.5 Hz, 2H), 4.56 (m, 2H), 4.27 (m, 1H), 3.42 (q, *J* = 6.5 Hz, 4H), 2.75 – 2.62 (m, 2H), 2.01 (ddt, *J* = 14.5, 9.8, 5.0 Hz, 1H), 1.89 (m, 3H), 1.78 – 1.62 (m, 4H).
^{13}C NMR (126 MHz, $CDCl_3$) δ: 166.14, 142.12, 133.41, 129.88, 129.80, 128.61, 128.44, 128.43, 125.89, 70.59, 70.17, 68.28, 51.61, 35.28, 32.49, 31.41, 29.24, 24.28.
HRMS (ESI) calcd for $C_{22}H_{27}BrO_3$ $[M + Na]^+$: 441.1036, found: 441.1031.

*2-bromo-6-(isobutyryloxy)hexyl benzoate (**3g**)*

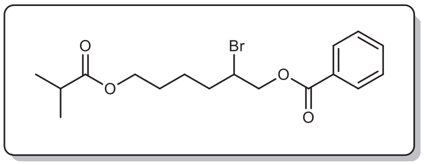

Yield: 58%
Physical state: colorless oil
R_f = 0.3 (1:20 EtOAc: hexane)
^{1}H NMR (500 MHz, $CDCl_3$) δ: 8.06 (d, *J* = 8.2 Hz, 2H), 7.59 (t, *J* = 7.5 Hz, 1H), 7.46 (t, *J* = 7.8 Hz, 2H), 4.61 – 4.50 (m, 2H), 4.25 (m, 1H), 4.13 – 4.04 (m, 2H), 2.53 (m, 1H), 2.05 – 1.83 (m, 2H), 1.71 – 1.60 (m, 4H), 1.16 (s, 3H), 1.15 (s, 3H).
^{13}C NMR (126 MHz, $CDCl_3$) δ: 177.32, 166.11, 133.45, 129.87, 129.74, 128.63, 128.58, 68.19, 63.92, 51.30, 34.97, 34.15, 28.15, 23.92, 19.14.
HRMS (ESI) calcd for $C_{17}H_{23}BrO_4$ $[M + H]^+$: 371.08525, found: 371.08502.

*2-bromo-6-((cyclohexanecarbonyl)oxy)hexyl benzoate (**3h**)*

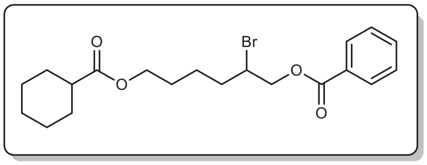

Yield: 68%
Physical state: colorless oil
R_f = 0.4 (1:20 EtOAc: hexane)
f^{1}H NMR (500 MHz, $CDCl_3$) δ: 8.06 (d, *J* = 8.8 Hz, 2H), 7.59 (t, *J* = 7.5 Hz, 1H), 7.46 (t, *J* z= 7.8 Hz, 2H), 4.61 – 4.49 (m, 2H), 4.31 – 4.21 (m, 1H), 4.08 (td, *J* = 5.8, 1.4 Hz, 2H), 2.34 – 2.20 (m, 1H), 2.06 – 1.81 (m, 3H), 1.76 – 1.59 (m, 7H), 1.46 – 1.15 (m, 6H).
^{13}C NMR (126 MHz, $CDCl_3$) δ: 176.31, 166.12, 133.45, 129.88, 129.75, 128.63, 128.58, 68.20, 63.77, 51.30, 43.36, 34.98, 29.17, 29.15, 28.19, 25.88, 25.57, 23.93.
HRMS (ESI) calcd for $C_{20}H_{27}BrO_4$ $[M + Na]^+$: 433.09849, found: 433.09841.

*2-bromo-6-(pivaloyloxy)hexyl benzoate (**3i**)*

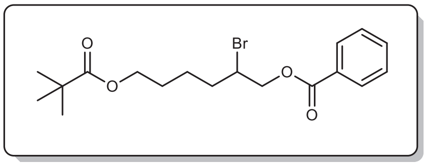

Yield: 30%
Physical state: colorless oil
R_f = 0.3 (1:20 EtOAc: hexane)
^{1}H NMR (500 MHz, $CDCl_3$) δ: 8.07 (d, *J* = 8.1 Hz, 2H), 7.59 (t, *J* = 7.4 Hz, 1H), 7.46 (t, *J* = 7.8 Hz, 2H), 4.61 – 4.50 (m, 2H), 4.25 (td, *J* = 10.0, 5.8 Hz, 1H), 4.08 (td, *J* = 6.2, 1.7 Hz, 2H), 2.04 – 1.85 (m, 2H), 1.72 – 1.61 (m, 4H), 1.19 (s, 9H).

^{13}C NMR (126 MHz, $CDCl_3$) δ: 172.82, 148.22, 133.47, 129.89, 128.64, 68.21, 63.98, 51.34, 34.97, 28.15, 27.35, 23.91.
HRMS (ESI) calcd for $C_{18}H_{25}BrO_4$ $[M + Na]^+$: 407.08284, found: 407.08265.

*2-bromo-6-((4-chlorobutanoyl)oxy)hexyl benzoate (**3j**)*

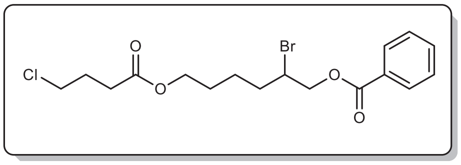

Yield: 71%
Physical state: colorless oil
R_f = 0.2 (1:20 EtOAc: hexane)
^{1}H NMR (500 MHz, $CDCl_3$) δ: 8.06 (d, J = 7.7 Hz, 2H), 7.58 (t, J = 7.5 Hz, 1H), 7.46 (t, J = 7.6 Hz, 2H), 4.61 – 4.50 (m, 2H), 4.29 – 4.22 (m, 1H), 4.11 (t, J = 5.9 Hz, 2H), 3.58 (qd, J = 6.5, 1.1 Hz, 2H), 2.49 (t, J = 7.3 Hz, 2H), 2.13 – 2.05 (m, 2H), 2.00 – 1.87 (m, 2H), 1.72 – 1.54 (m, 4H).
^{13}C NMR (126 MHz, $CDCl_3$) δ: 172.82, 166.09, 133.45, 129.86, 128.63, 128.59, 68.15, 64.28, 51.24, 44.22, 34.93, 31.30, 28.08, 27.74, 23.92.
HRMS (ESI) calcd for $C_{17}H_{22}BrClO_4$ $[M + Na]^+$: 427.02822, found: 427.02839.

*2-bromo-6-((4-bromobutanoyl)oxy)hexyl benzoate (**3k**)*

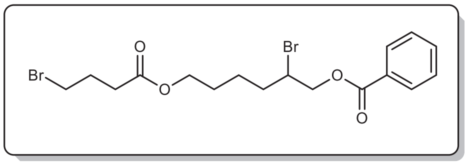

Yield: 69%
Physical state: colorless oil
R_f = 0.2 (1:20 EtOAc: hexane)
^{1}H NMR (500 MHz, $CDCl_3$) δ: 8.06 (d, J = 7.7 Hz, 2H), 7.58 (t, J = 7.5 Hz, 1H), 7.46 (t, J = 7.6 Hz, 2H), 4.61 – 4.50 (m, 2H), 4.25 (pd, J = 5.3, 3.4 Hz, 1H), 4.11 (t, J = 5.9 Hz, 2H), 3.45 (td, J = 6.5, 1.1 Hz, 2H), 2.49 (t, J = 7.3 Hz, 2H), 2.22 – 2.13 (m, 2H), 2.00 – 1.86 (m, 2H), 1.73 – 1.57 (m, 4H).
^{13}C NMR (126 MHz, $CDCl_3$) δ: 172.68, 166.08, 133.45, 129.86, 128.62, 128.59, 68.15, 64.29, 51.24, 34.93, 32.86, 32.54, 28.08, 27.84, 23.92.
HRMS (ESI) calcd for $C_{17}H_{22}Br_2O_4$ $[M + Na]^+$: 470.97771, found: 470.97808.

*2-bromo-8-oxononyl benzoate (**3l**)*

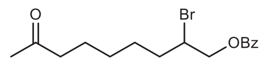

Yield: 64%
Physical state: colorless oil
R_f = 0.5 (1:20 EtOAc: hexane)
^{1}H NMR (500 MHz, $CDCl_3$) δ: 8.06 (d, *J* = 8.5 Hz, 2H), 7.63 – 7.53 (m, 1H), 7.46 (t, *J* = 7.7 Hz, 2H), 4.60 – 4.48 (m, 2H), 4.24 (dtd, *J* = 9.1, 6.1, 4.4 Hz, 1H), 2.43 (t, *J* = 7.4 Hz, 2H), 2.13 (s, 3H), 1.95 – 1.84 (m, 2H), 1.64 – 1.55 (m, 4H), 1.38 – 1.31 (m, 2H).
^{13}C NMR (126 MHz, $CDCl_3$) δ: 209.22, 166.15, 133.43, 129.87, 128.62, 128.57, 68.27, 51.59, 43.59, 35.17, 30.07, 28.54, 27.13, 23.58.
HRMS (ESI) calcd for $C_{16}H_{21}BrO_3$ $[M + Na]^+$: 363.05663, found: 363.05632.

*2-bromo-10-((triisopropylsilyl)oxy)decyl benzoate (**3m**)*

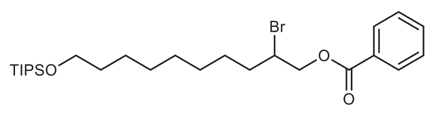

Yield: 99%
Physical state: pale-yellow oil
R_f = 0.7 (1:20 EtOAc: hexane)
^{1}H NMR (500 MHz, $CDCl_3$) δ: 8.07 (d, *J* = 6.9 Hz, 2H), 7.58 (t, *J* = 7.4 Hz, 1H), 7.46 (t, *J* = 7.8 Hz, 2H), 4.55 (dd, *J* = 6.2, 1.4 Hz, 2H), 4.29 – 4.22 (m, 1H), 3.66 (t, *J* = 6.6 Hz, 2H), 2.00 – 1.84 (m, 2H), 1.65 – 1.48 (m, 6H), 1.38 – 1.26 (m, 6H), 1.05 (d, *J* = 4.8 Hz, 21H).
^{13}C NMR (126 MHz, $CDCl_3$) δ: 166.18, 133.40, 129.89, 129.86, 128.61, 68.36, 63.61, 51.85, 35.45, 33.14, 29.54, 29.48, 29.05, 27.35, 25.91, 18.19, 12.16.
HRMS (ESI) calcd for $C_{26}H_{45}BrO_3Si$ $[M + H]^+$: 513.23941, found: 513.23959.

*2-bromo-10-(pivaloyloxy)decyl benzoate (**3n**)*

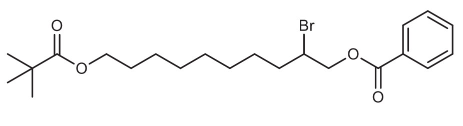

Yield: 45%
Physical state: colorless oil
R_f = 0.4 (1:20 EtOAc: hexane)
^{1}H NMR (500 MHz, $CDCl_3$) δ: 8.07 (d, *J* = 6.9 Hz, 2H), 7.58 (t, *J* = 7.5 Hz, 1H), 7.46 (t, *J* = 7.8 Hz, 2H), 4.55 (dd, *J* = 6.1, 1.5 Hz, 2H), 4.30 – 4.21 (m, 1H), 4.04 (t, *J* = 6.5 Hz, 2H), 2.01 – 1.79 (m, 2H), 1.66 – 1.42 (m, 6H), 1.31 (d, *J* = 8.7 Hz, 6H), 1.19 (s, 9H).
^{13}C NMR (126 MHz, $CDCl_3$) δ: 178.81, 166.15, 133.41, 129.88, 129.83, 128.61, 128.56, 68.32, 64.54, 51.79, 38.87, 35.40, 29.41, 29.23, 28.99, 28.71, 27.35, 27.31, 26.00.
HRMS (ESI) calcd for $C_{22}H_{33}BrO_4$ $[M + Na]^+$: 463.14544, found: 463.14510.

*2-bromo-6-((tert-butoxycarbonyl)amino)hexyl benzoate (**3o**)*

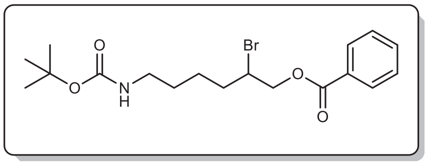

Yield: 53%
Physical state: colorless oil
R_f = 0.5 (1:20 EtOAc: hexane)
^{1}H NMR (500 MHz, $CDCl_3$) δ: 8.06 (d, *J* = 6.9 Hz, 2H), 7.58 (t, *J* = 7.4 Hz, 1H), 7.46 (t, *J* = 7.8 Hz, 2H), 4.60 – 4.49 (m, 2H), 4.24 (ddt, *J* = 8.5, 6.0, 3.1 Hz, 1H), 3.13 (s, 1H), 2.05 – 1.48 (m, 8H), 1.44 (s, 9H).
^{13}C NMR (126 MHz, $CDCl_3$) δ: 166.12, 133.43, 129.88, 129.75, 128.62, 128.58, 68.20, 51.44, 35.00, 29.63, 28.54, 28.52, 24.63.
HRMS (ESI) calcd for $C_{18}H_{26}BrNO_4$ $[M + Na]^+$: 422.09374, found: 422.09355.

*tert-butyl 2-(bromomethyl)piperidine-1-carboxylate (**4**)*

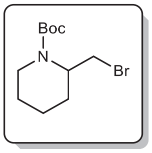

Yield: 70%
Physical state: colorless oil
R_f = 0.2 (1:20 EtOAc: hexane)
^{1}H NMR (400 MHz, $CDCl_3$) δ: 4.21 – 4.10 (m, 1H), 3.84 (dd, *J* = 10.3, 4.4 Hz, 1H), 3.61 (t, *J* = 10.1 Hz, 1H), 3.14 (t, *J* = 6.7 Hz, 2H), 2.22 – 2.09 (m, 1H), 1.85 – 1.75 (m, 1H), 1.64 – 1.51 (m, 4H), 1.44 (s, 9H).
^{13}C NMR (126 MHz, $CDCl_3$) δ: 156.11, 52.80, 40.42, 36.26, 35.78, 29.50, 28.56, 24.19.
HRMS (ESI) calcd. for $C_{11}H_{20}BrNO_2$ $[M + H]^+$: 278.06774, found: 278.06569.

*2-bromo-6-((dimethyl((2-methylbut-3-en-2-yl)oxy)silyl)oxy)hexyl benzoate (**11a**)*

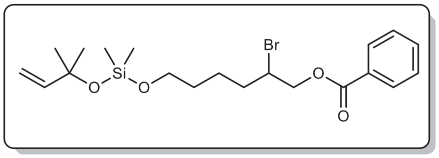

Yield: 63%
Physical state: pale-yellow oil
R_f = 0.4 (1:30 EtOAc: hexane)
^{1}H NMR (500 MHz, $CDCl_3$) δ: 8.07 (d, *J* = 7.2 Hz, 2H), 7.58 (t, *J* = 7.4 Hz, 1H), 7.46 (t, *J* = 7.7 Hz, 2H), 5.96 (dd, *J* = 17.3, 10.6 Hz, 1H), 5.24 – 4.85 (m, 2H), 4.64 – 4.49 (m, 2H), 4.26 (dd, *J* = 9.2, 4.5 Hz, 1H), 3.68 (t, *J* = 6.2 Hz, 2H), 1.95 (dddd, *J* = 42.3, 19.0, 9.8, 5.1 Hz, 2H), 1.78 – 1.65 (m, 1H), 1.65 – 1.46 (m, 3H), 1.35 (s, 6H), 0.12 (s, 6H).
^{13}C NMR (126 MHz, $CDCl_3$) δ: 166.14, 146.44, 133.41, 129.89, 129.82, 128.61, 110.78, 74.13, 68.31, 62.05, 51.68, 35.25, 31.93, 30.10, 23.93, -0.29.
HRMS (APCI) calcd for $C_{20}H_{31}BrO_4Si$ $[M + H]^+$: 467.10494, found: 467.10343.

*2-bromo-6-((dimethyl(pent-4-en-2-yloxy)silyl)oxy)hexyl benzoate (**11b**)*

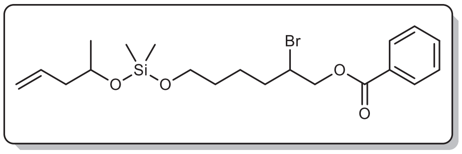

Yield: 68%
Physical state: pale-yellow oil
R_f = 0.4 (1:30 EtOAc: hexane)
^{1}H NMR (500 MHz, $CDCl_3$) δ: 8.07 (d, *J* = 7.1 Hz, 2H), 7.58 (t, *J* = 7.4 Hz, 1H), 7.46 (t, *J* = 7.7 Hz, 2H), 5.80 (td, *J* = 17.3, 7.2 Hz, 1H), 5.14 – 4.94 (m, 2H), 4.70 – 4.46 (m, 2H), 4.26 (dq, *J* = 10.6, 6.0 Hz, 1H), 3.96 (h, *J* = 6.1 Hz, 1H), 3.69 (t, *J* = 6.1 Hz, 2H), 2.34 – 2.12 (m, 2H), 2.02 – 1.84 (m, 2H), 1.74 – 1.64 (m, 1H), 1.64 – 1.48 (m, 4H), 1.17 (d, *J* = 6.1 Hz, 3H), 0.12 (s, 6H).
^{13}C NMR (126 MHz, $CDCl_3$) δ: 166.14, 135.42, 133.42, 129.88, 129.81, 128.62, 116.97, 68.34, 68.29, 62.19, 51.64, 44.12, 35.23, 31.97, 23.88, 23.44, -2.44.
HRMS (APCI) calcd for $C_{20}H_{31}BrO_4Si$ $[M + H]^+$: 467.10494, found: 467.10331.

8. Derivatization of anti-Markovnikov products

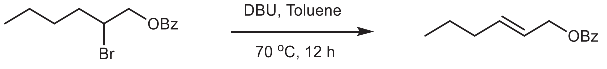

To a solution of 2-bromohexyl benzoate **3ba** (285 mg, 1.0 mmol) in toluene (1 mL) was added DBU (0.5 mL). The resultant mixture was heated at 70 ºC for 12 h. Then, the solution was cooled to 23 ºC and *n*-hexane (5 mL) was added. The mixture was washed with brine (3 mL), dried over anhydrous Na_2SO_4, filtered, and concentrated under reduced pressure. The residue was purified over silica gel column chromatography (EtOAc:hexanes, 1:40) to afford the (*E*)-hex-2-en-1-yl benzoate **5**.

*(E)-hex-2-en-1-yl benzoate (**5**)*

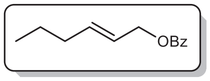

Yield: 72%
Physical state: colorless oil
R_f = 0.3 (1:40 EtOAc: hexane)
^{1}H NMR (500 MHz, $CDCl_3$) δ: 8.06 (d, *J* = 7.1 Hz, 1H), 7.61 – 7.50 (m, 1H), 7.44 (t, *J* = 7.7 Hz, 1H), 5.94 – 5.60 (m, 1H), 4.77 (d, *J* = 6.4 Hz, 1H), 2.07 (q, *J* = 6.9 Hz, 1H), 1.43 (h, *J* = 7.4 Hz, 1H), 0.92 (t, *J* = 7.4 Hz, 2H).
^{13}C NMR (126 MHz, $CDCl_3$) δ: 166.61, 136.59, 133.00, 130.56, 129.75, 128.46, 124.10, 65.91, 34.50, 29.85, 22.21.
HRMS (APCI) calcd for $C_{13}H_{16}O_2$ $[M + Na]^+$: 227.10425, found: 227.10419.

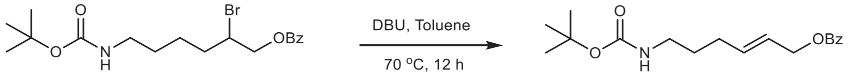

To a solution of *2-bromo-6-[(tert-butoxycarbonyl)amino]hexyl benzoate* **3o** (78.6 mg, 0.196 mmol) in toluene (0.5 mL) was added DBU (0.25 mL). The resultant mixture was heated at 70 ºC for 12 h. Then, the solution was cooled to 23 ºC and *n*-hexane (2.5 mL) was added. The mixture was washed with brine (3 mL), dried over anhydrous Na_2SO_4, filtered, and concentrated under reduced pressure. The residue was purified over silica gel column chromatography (EtOAc:hexanes, 1:4) to afford the *(E)-6-[(tert-butoxycarbonyl)amino]hex-2-en-1-yl benzoate* **6**.

*(E)-6-[(tert-butoxycarbonyl)amino]hex-2-en-1-yl benzoate (**6**)*

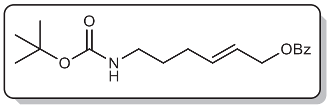

Yield: 53%
Physical state: colorless oil
R_f = 0.4 (1:4 EtOAc: hexane)

^{1}**H NMR** (500 MHz, $CDCl_3$) δ: 8.05 (d, J = 6.9 Hz, 2H), 7.56 (t, J = 7.4 Hz, 1H), 7.44 (t, J = 7.6 Hz, 2H), 5.84 (dd, J = 14.6, 7.3 Hz, 1H), 5.77 – 5.65 (m, 1H), 4.76 (dd, J = 6.2, 1.1 Hz, 2H), 3.14 (d, J = 7.1 Hz, 2H), 2.15 – 2.10 (m, 2H), 1.66 – 1.61 (m, 3H), 1.44 (s, 9H).
^{13}**C NMR** (100 MHz, $CDCl_3$) δ: 166.58, 156.11, 135.32, 133.06, 130.45, 129.77, 128.48, 124.80, 115.58, 65.65, 46.00, 40.22, 38.11, 29.68, 29.44, 28.56.
HRMS (ESI) calcd. for $C_{18}H_{25}NO_4$ $[M + H]^+$: 320.17836, found: 320.40100.

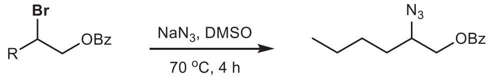

3 7-9

To a stirred solution of bromo-benzoate **3** (0.5 mmol, 1.0 equiv) in DMSO (5 mL) was added sodium azide (0.6 mmol, 1.2 equiv). The resultant mixture was heated at 80 °C for 6 h. Then, the solution was cooled to room temperature and brine (20 mL) was added. The mixture was extracted with ethyl acetate (10 mL × 3). The combined organic phase was washed with saturated aqueous $NaHCO_3$ (5 mL × 3) and brine (5 mL), dried over anhydrous Na_2SO_4, filtered, and concentrated under reduced pressure. The residue was purified over silica gel column chromatography (EtOAc:hexanes, 1:10) to afford the desired azide product.

*2-azidohexyl benzoate (**3ba**)*

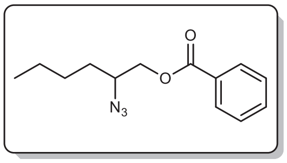

Yield: 85%
Physical state: pale-yellow oil
^{1}**H NMR** (400 MHz, $CDCl_3$) δ: 8.07 (d, J = 9.8 Hz, 2H), 7.59 (t, J = 7.4 Hz, 1H), 7.46 (t, J = 7.7 Hz, 2H), 4.55 (d, J = 5.8 Hz, 2H), 4.32 – 4.18 (m, 1H), 2.04 – 1.80 (m, 2H), 1.67 – 1.25 (m, 4H), 0.93 (t, J = 7.3 Hz, 3H).
^{13}**C NMR** (126 MHz, $CDCl_3$) δ: 166.42, 133.43, 129.93, 128.64, 67.37, 61.30, 30.65, 28.23, 22.56, 14.05.
HRMS (ESI) calcd for $C_{13}H_{17}N_3O_2$ $[M + Na]^+$: 270.12130, found: 270.12091.

*2-azido-6-[(cyclohexanecarbonyl)oxy]hexyl benzoate (**3ba**)*

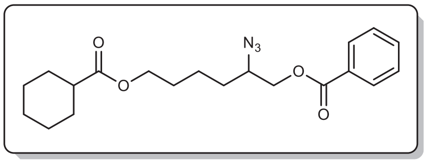

Yield: 88%
Physical state: pale-yellow oil
^{1}**H NMR** (500 MHz, $CDCl_3$) δ: 8.07 (d, J = 9.6 Hz, 2H), 7.62 – 7.55 (m, 1H), 7.46 (t, J = 7.7 Hz, 2H), 4.49 (dd, J = 11.6, 3.5 Hz, 1H), 4.29 (dd, J = 11.6, 7.7 Hz, 1H), 4.07 (dt, J = 15.2, 6.4 Hz, 2H), 3.71 (dp, J = 8.2, 3.9 Hz, 1H), 2.34 – 2.19 (m, 1H), 1.96 – 1.80 (m, 2H), 1.75 – 1.57 (m, 8H), 1.42 (dd, J = 13.5, 10.3 Hz, 2H), 1.31 – 1.21 (m, 4H).

^{13}**C NMR** (126 MHz, $CDCl_3$) δ: 176.31, 166.37, 133.48, 129.93, 128.66, 67.27, 63.76, 61.11, 43.36, 30.62, 29.18, 29.15, 28.53, 25.89, 25.58, 25.56, 22.69.
HRMS (ESI) calcd for $C_{20}H_{27}N_3O_4$ $[M + Na]^+$: 396.18938, found: 396.18934.

*2-azido-10-[(triisopropylsilyl)oxy]decyl benzoate (**3ba**)*

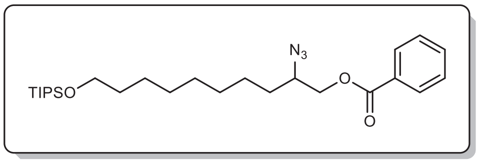

Yield: 85%
Physical state: pale-yellow oil
^{1}**H NMR** (500 MHz, $CDCl_3$) δ: 8.07 (d, *J* = 7.7 Hz, 2H), 7.58 (t, *J* = 7.4 Hz, 1H), 7.46 (t, *J* = 7.6 Hz, 2H), 4.48 (dd, *J* = 11.5, 3.4 Hz, 1H), 4.27 (dd, *J* = 11.5, 7.7 Hz, 1H), 3.71 (m, 1H), 3.67 (t, *J* = 6.7 Hz, 2H), 1.64 – 1.47 (m, 6H), 1.34 – 1.30 (m, 6H), 1.05 (d, *J* = 5.0 Hz, 21H).
^{13}**C NMR** (126 MHz, $CDCl_3$) δ: 166.42, 133.42, 129.94, 129.74, 128.64, 67.38, 63.60, 61.31, 33.14, 30.96, 29.56, 29.48, 29.40, 26.09, 25.92, 18.19, 12.16.
HRMS (ESI) calcd for $C_{26}H_{45}N_3O_3Si$ $[M + Na]^+$: 498.31224, found: 498.31227.

9. <u>Preparation of tosylate **14**</u>

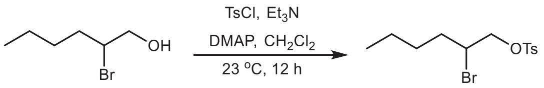

26 **14**

To a solution of 2-bromohexan-1-ol **26** (363 mg, 2 mmol), TsCl (420 mg, 2.2 mmol), DMAP (24.4 mg, 0.2 mmol) in CH_2Cl_2 (5 mL) was added Et_3N (306 μL, 2.2 mmol). The resultant mixture was stirred at 23 °C for 12 h. Then, water was added to the solution and the mixture was extracted by ethyl acetate (10 mL × 3). The combined organic phase was washed with saturated aqueous $NaHCO_3$ (5 mL × 3) and brine (5 mL), dried over anhydrous Na_2SO_4, filtered, and concentrated under reduced pressure. The residue was purified over silica gel column chromatography to afford the 2-bromohexyl 4-methylbenzenesulfonate **14** in 96% yield as a colorless oil.

*2-bromohexyl 4-methylbenzenesulfonate (**14**)*

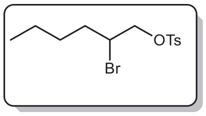

Yield: 96%
Physical state: colorless oil
R_f = 0.5 (1:20 EtOAc: hexane)
^{1}H NMR (500 MHz, $CDCl_3$) δ: 7.81 (d, J = 8.3 Hz, 2H), 7.36 (d, J = 8.0 Hz, 2H), 4.27 – 4.10 (m, 2H), 4.03 (dddd, J = 9.5, 7.2, 5.7, 4.0 Hz, 1H), 2.46 (s, 3H), 1.90 (ddt, J = 14.6, 9.6, 4.9 Hz, 1H), 1.75 – 1.63 (m, 1H), 1.53 – 1.38 (m, 1H), 1.38 – 1.20 (m, 3H), 0.89 (t, J = 7.2 Hz, 3H).
^{13}C NMR (126 MHz, $CDCl_3$) δ: 145.33, 132.75, 130.10, 128.15, 72.34, 49.94, 34.43, 29.04, 22.10, 21.83, 13.98.
HRMS (ESI) calcd for $C_{13}H_{19}BrO_3S$ $[M + H]^+$: 335.02238 found: 335.02210.

10. NMR Spectra

^{1}H NMR (500 MHz, $CDCl_3$)

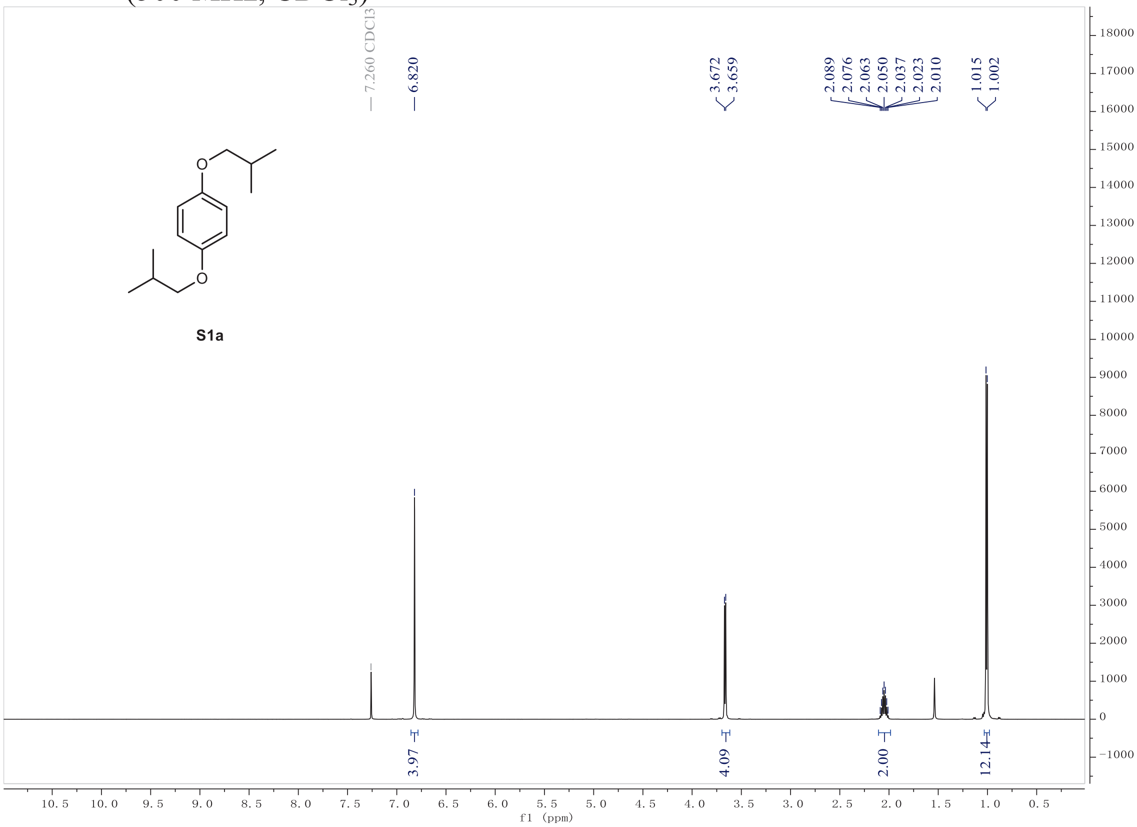

^{13}C NMR (126 MHz, $CDCl_3$)

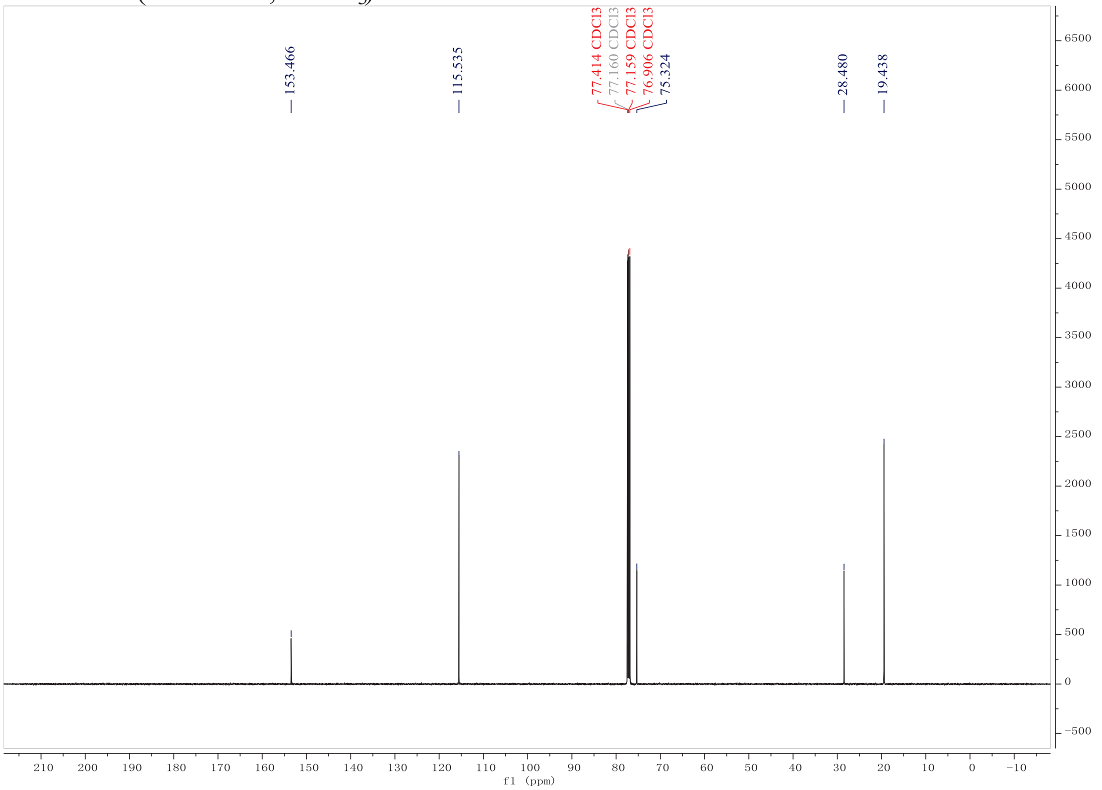

^{1}H NMR (500 MHz, $CDCl_3$)

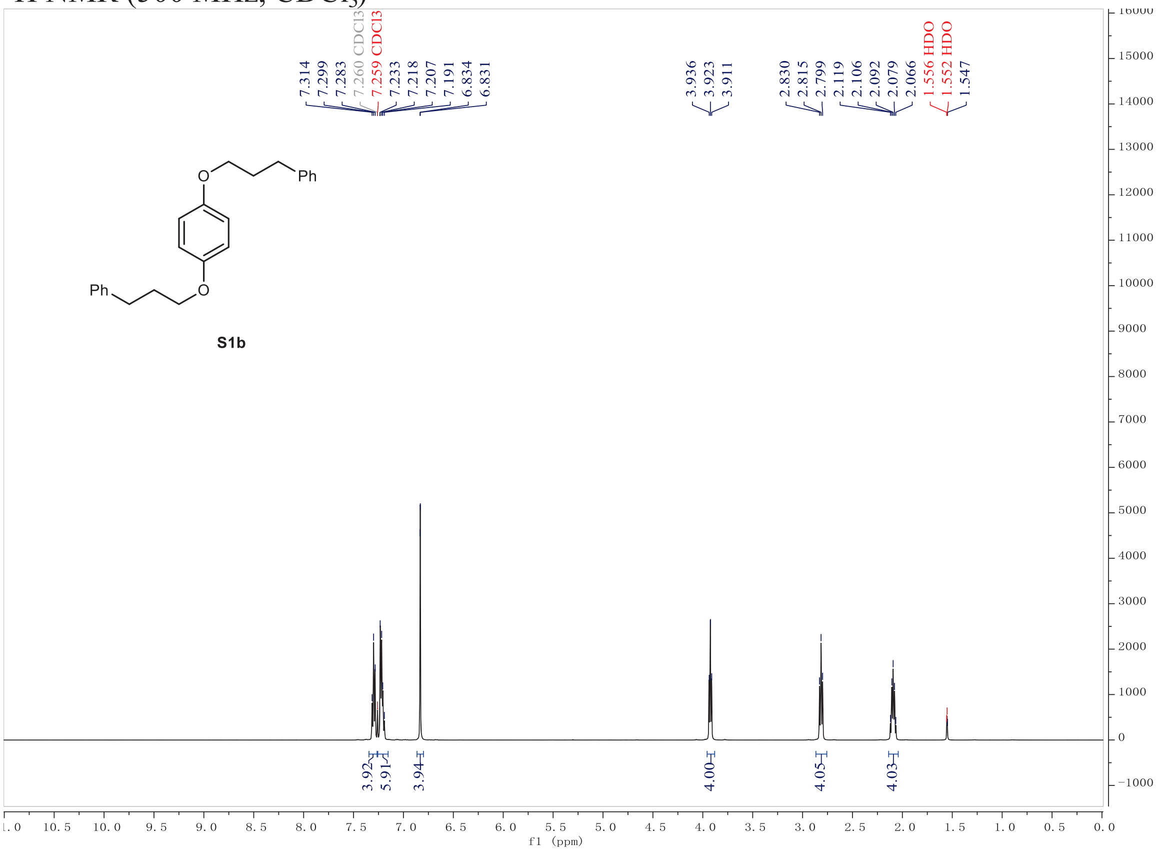

^{13}C NMR (126 MHz, $CDCl_3$)

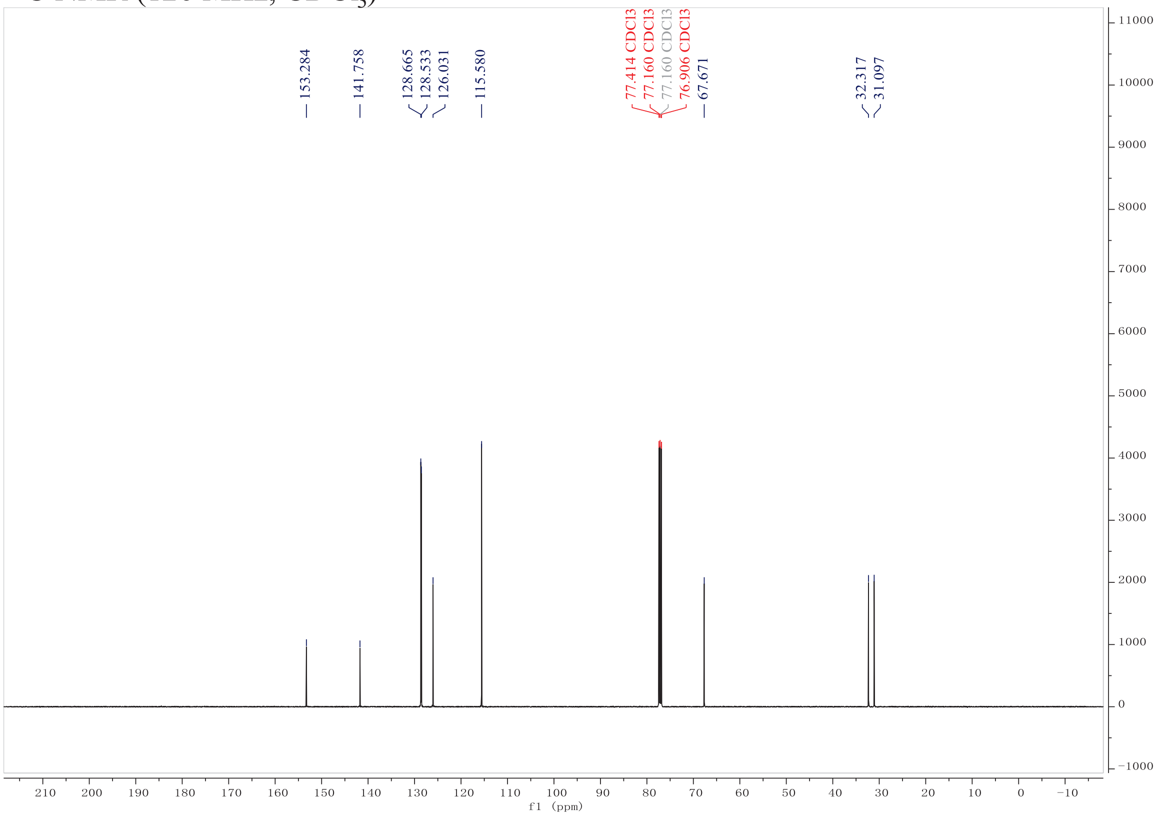

^{1}H NMR (500 MHz, $CDCl_3$)

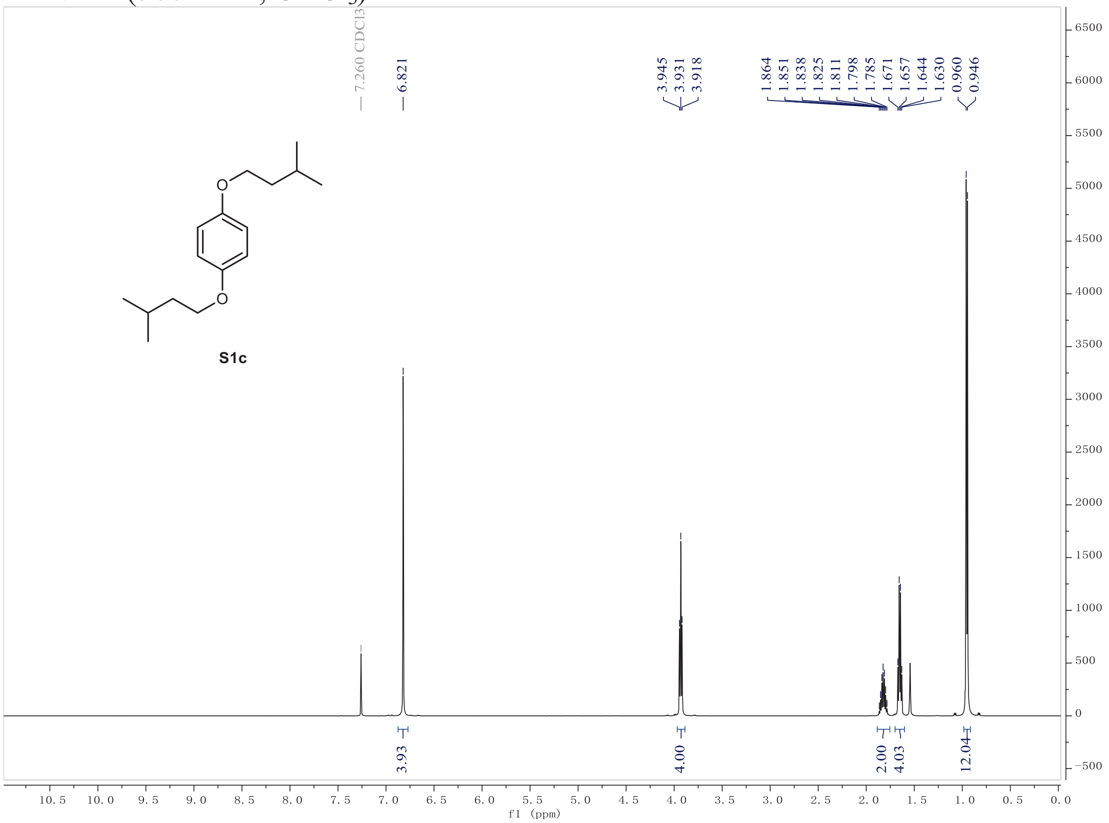

^{13}C NMR (126 MHz, $CDCl_3$)

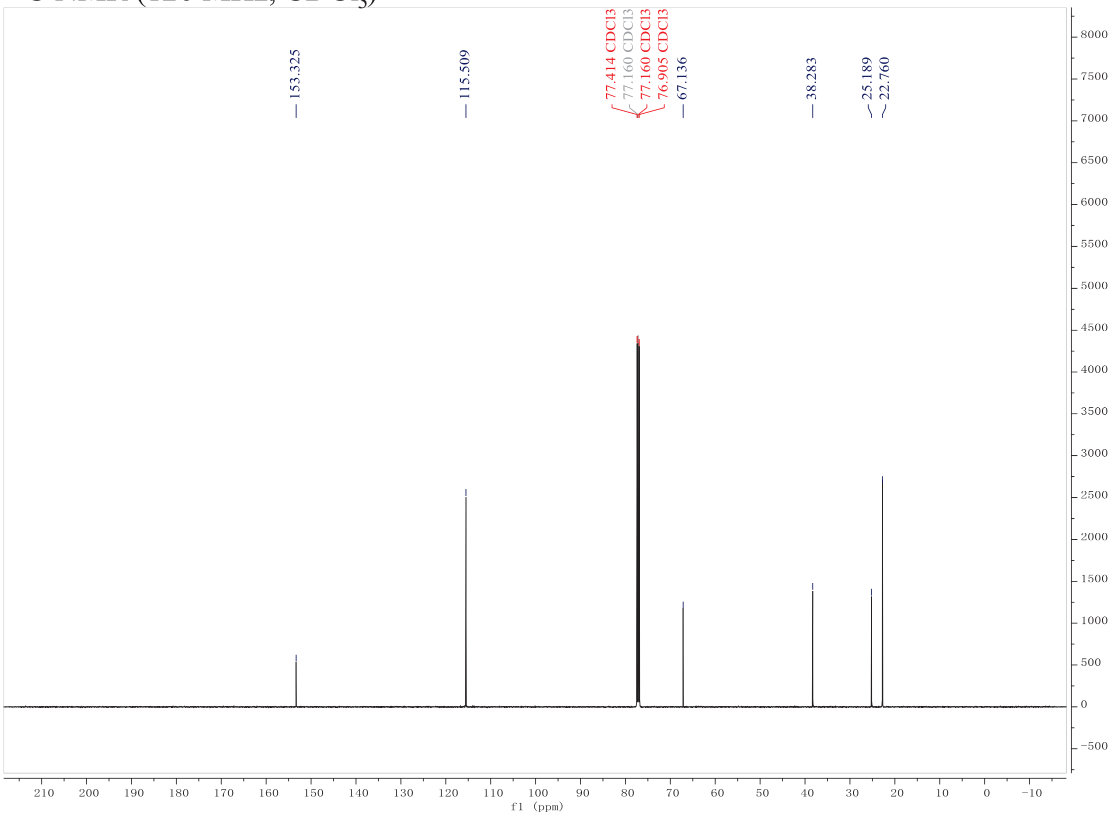

^{1}H NMR (500 MHz, $CDCl_3$)

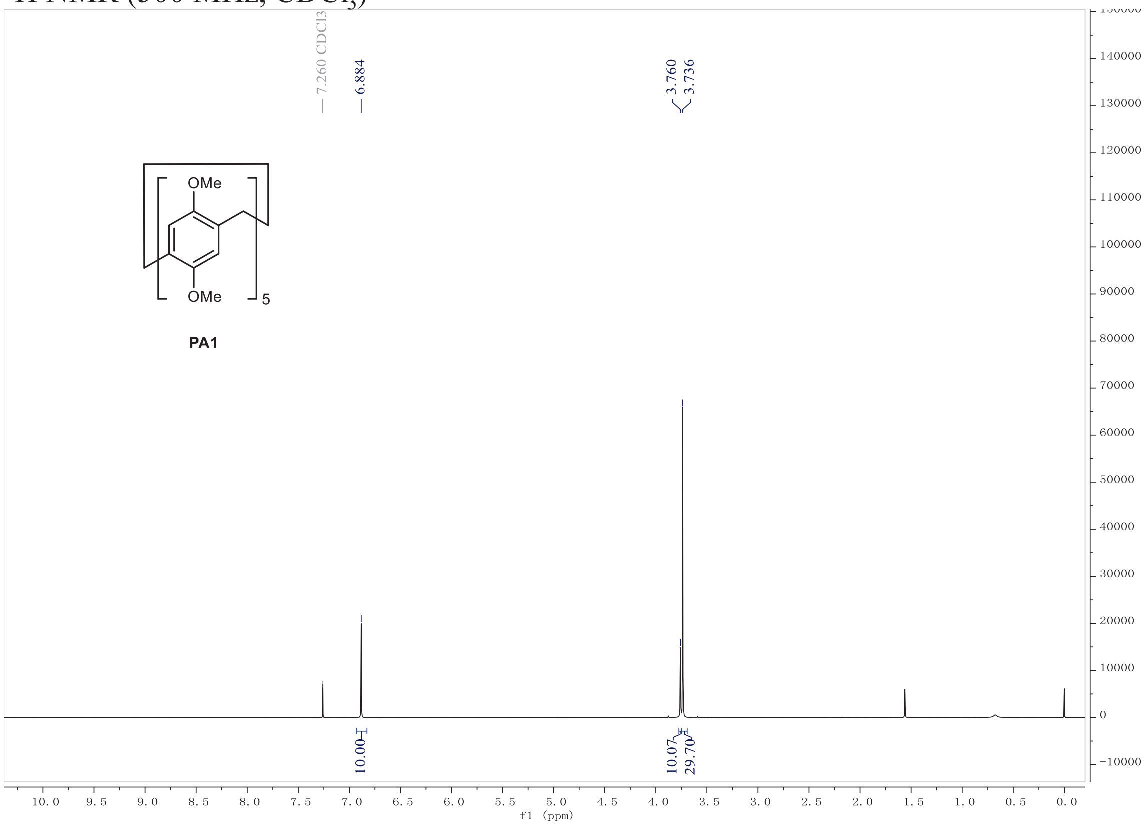

^{13}C NMR (126 MHz, $CDCl_3$)

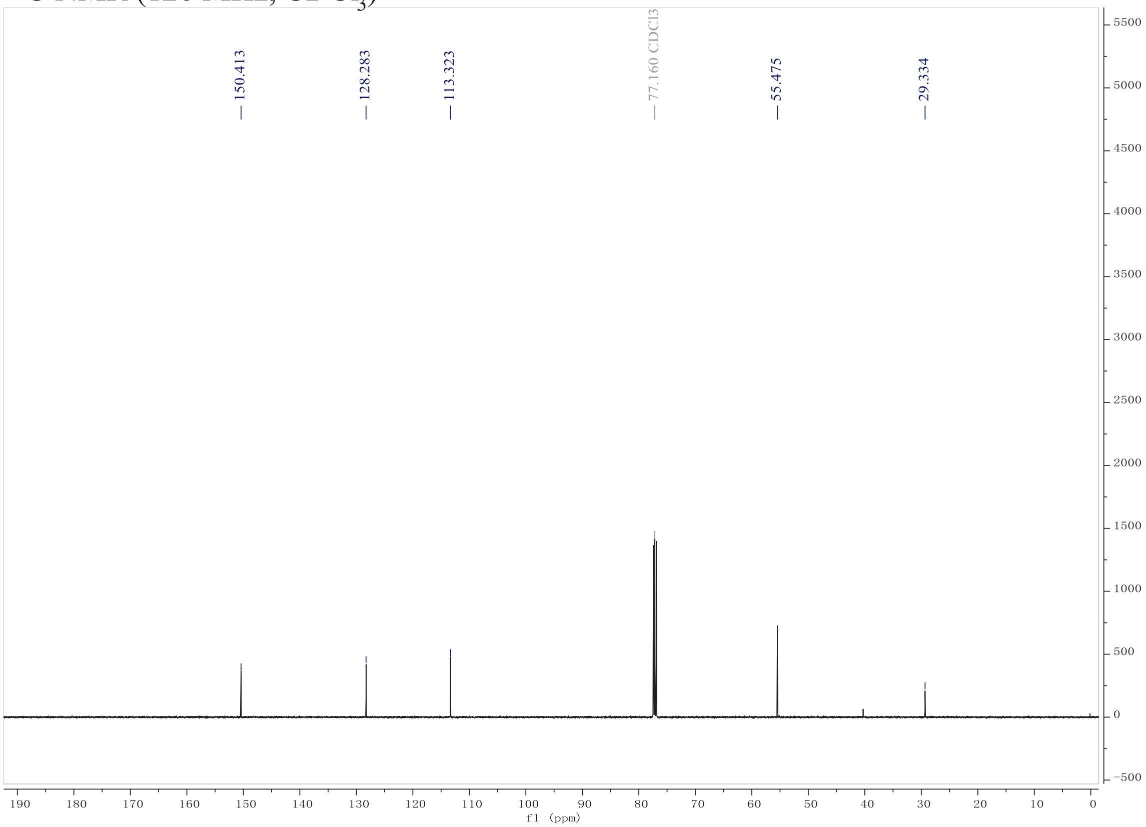

^{1}H NMR (500 MHz, $CDCl_3$)

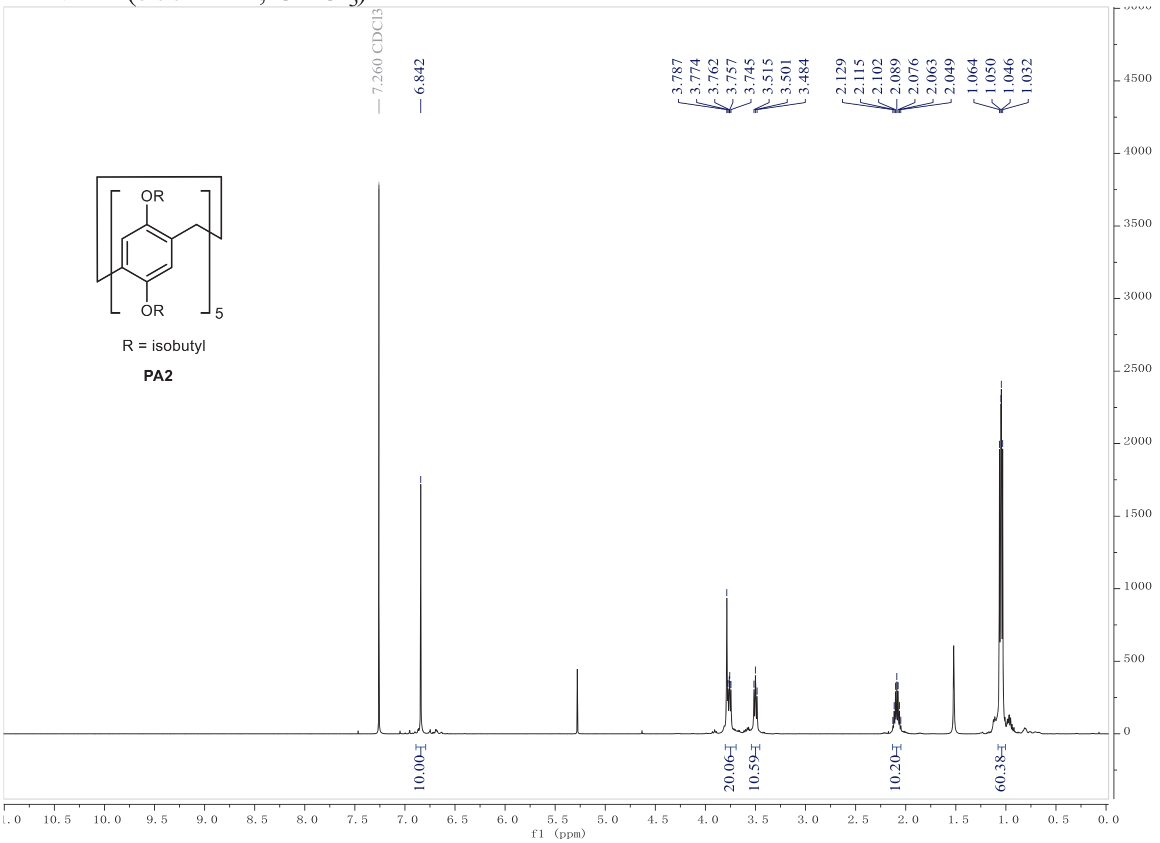

^{13}C NMR (126 MHz, $CDCl_3$)

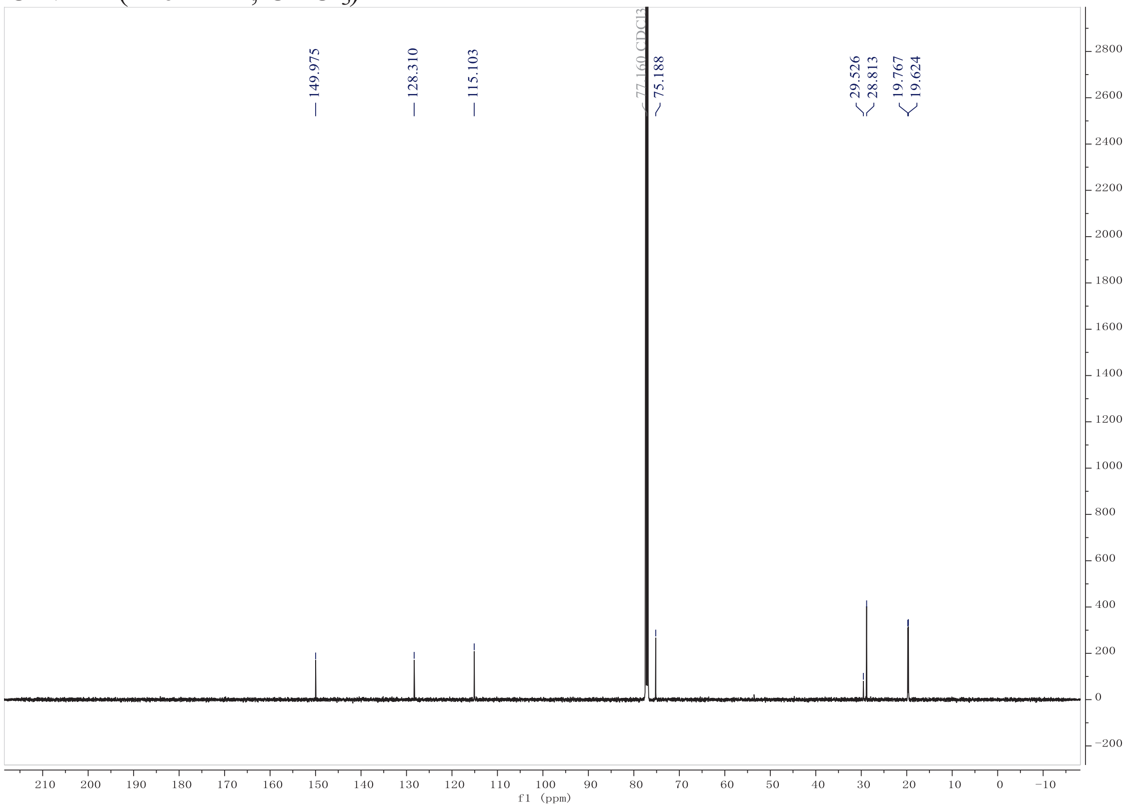

1

^{1}H NMR (500 MHz, $CDCl_3$)

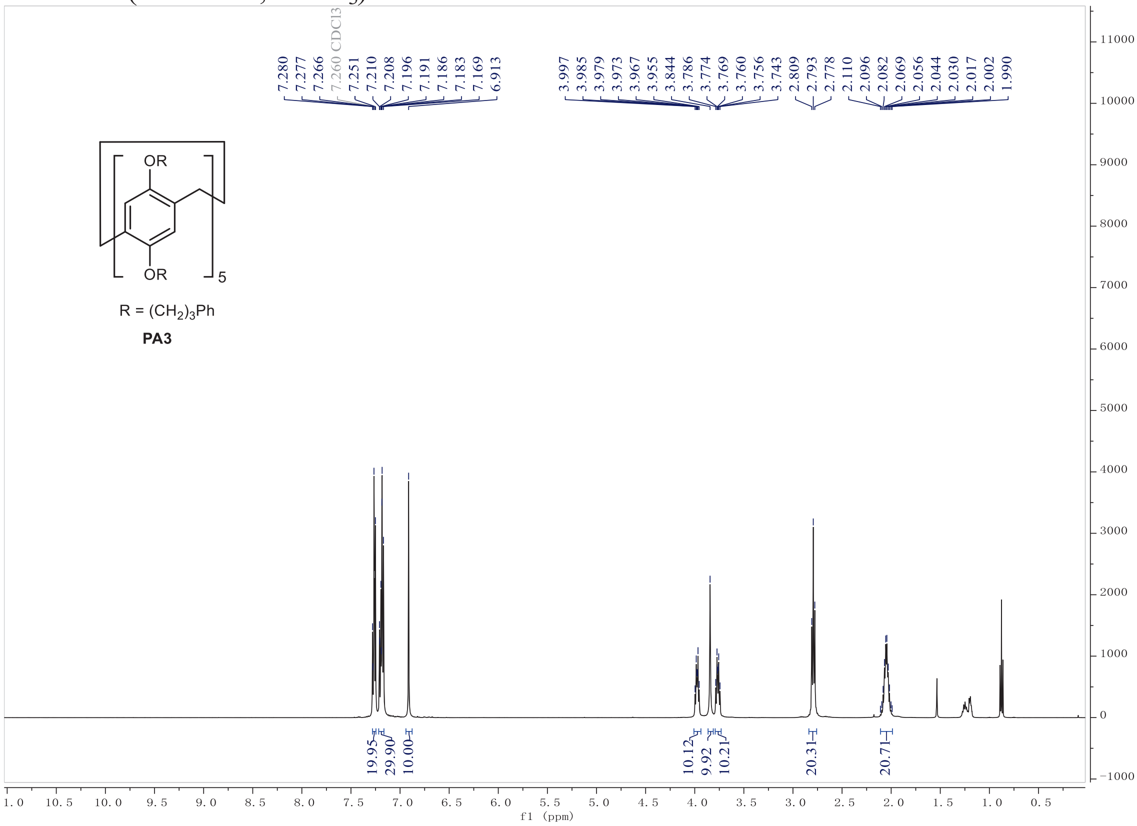

^{13}C NMR (126 MHz, $CDCl_3$)

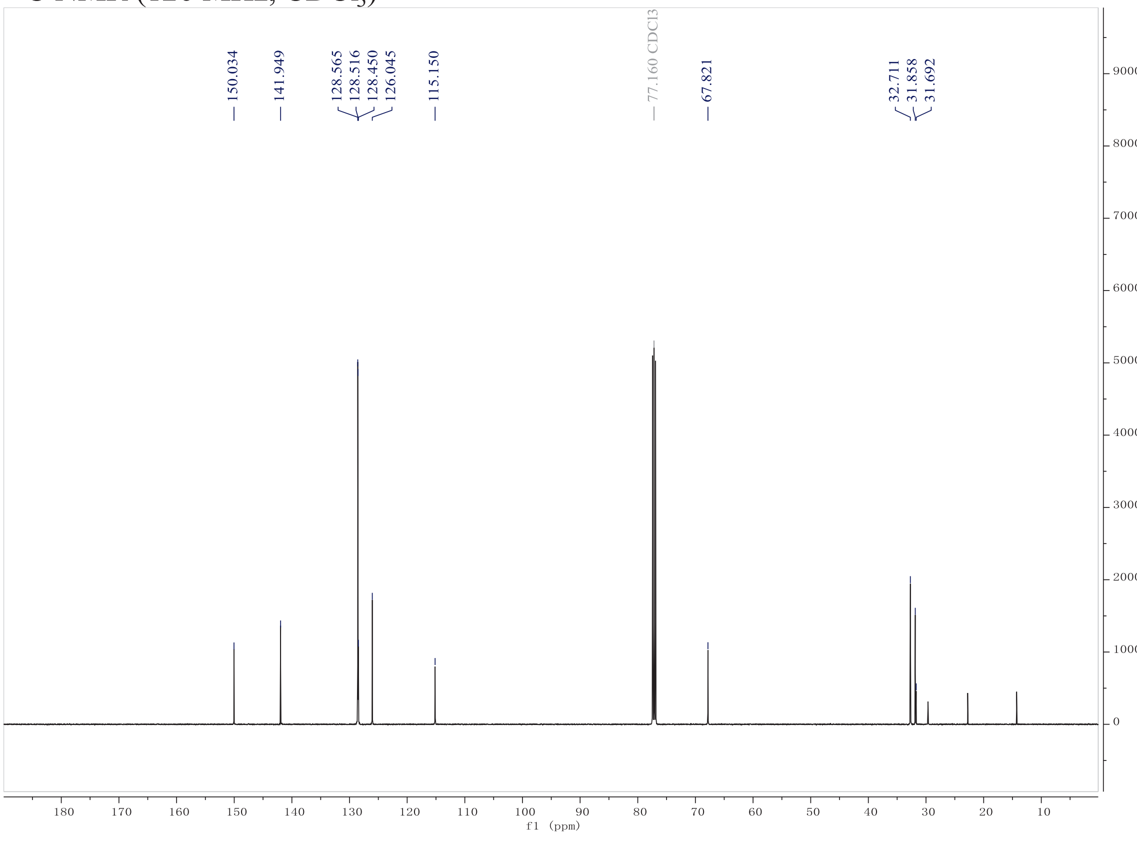

^{1}H NMR (500 MHz, $CDCl_3$)

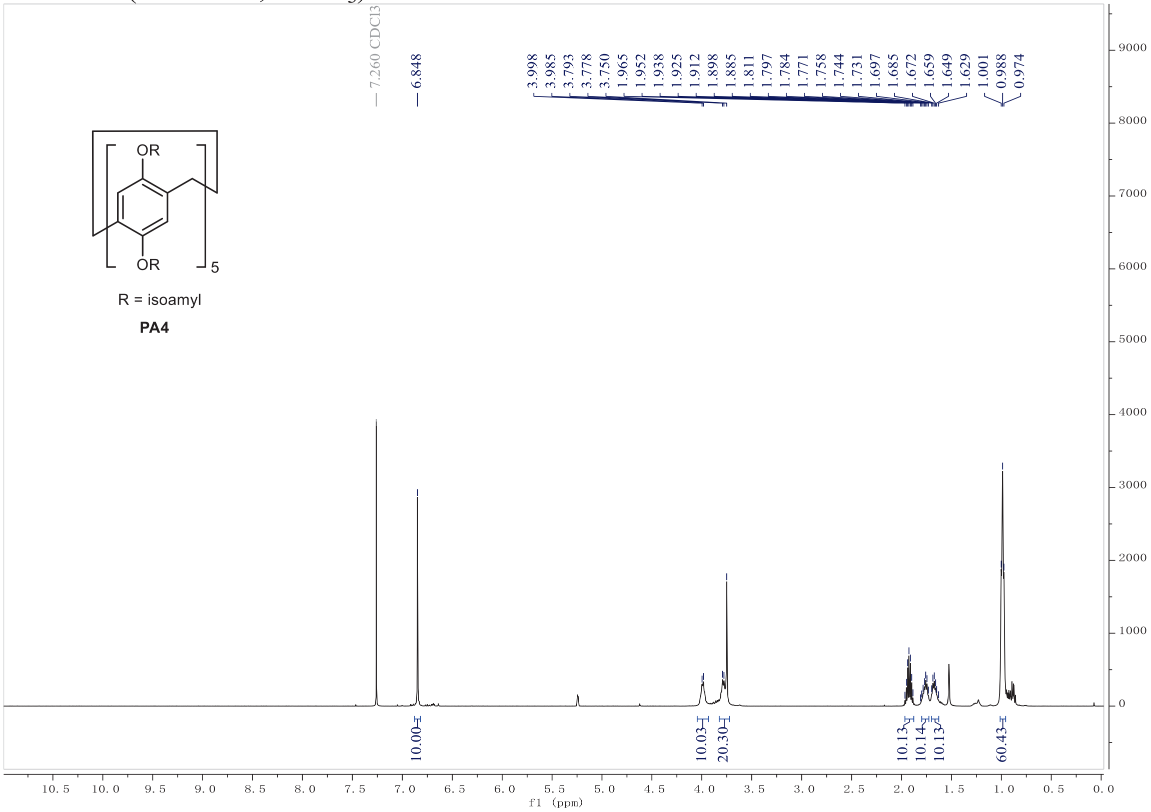

^{13}C NMR (126 MHz, $CDCl_3$)

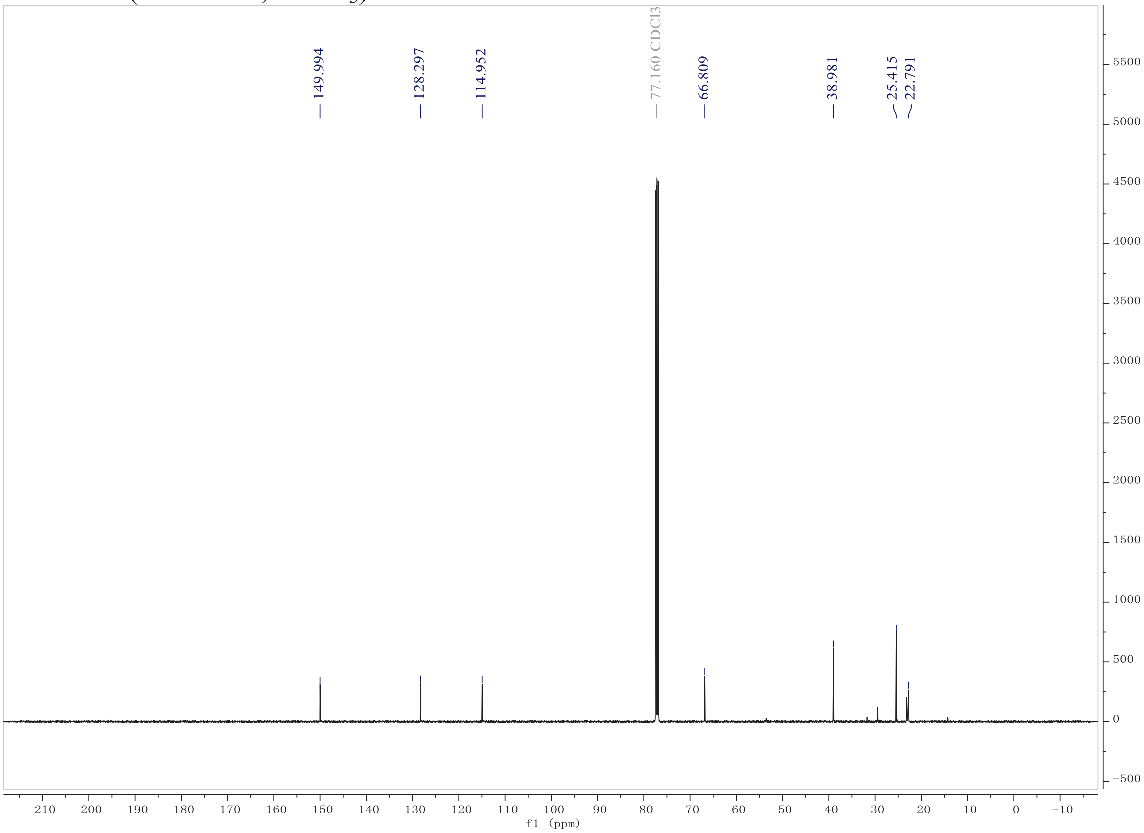

^{1}H NMR (500 MHz, $CDCl_3$)

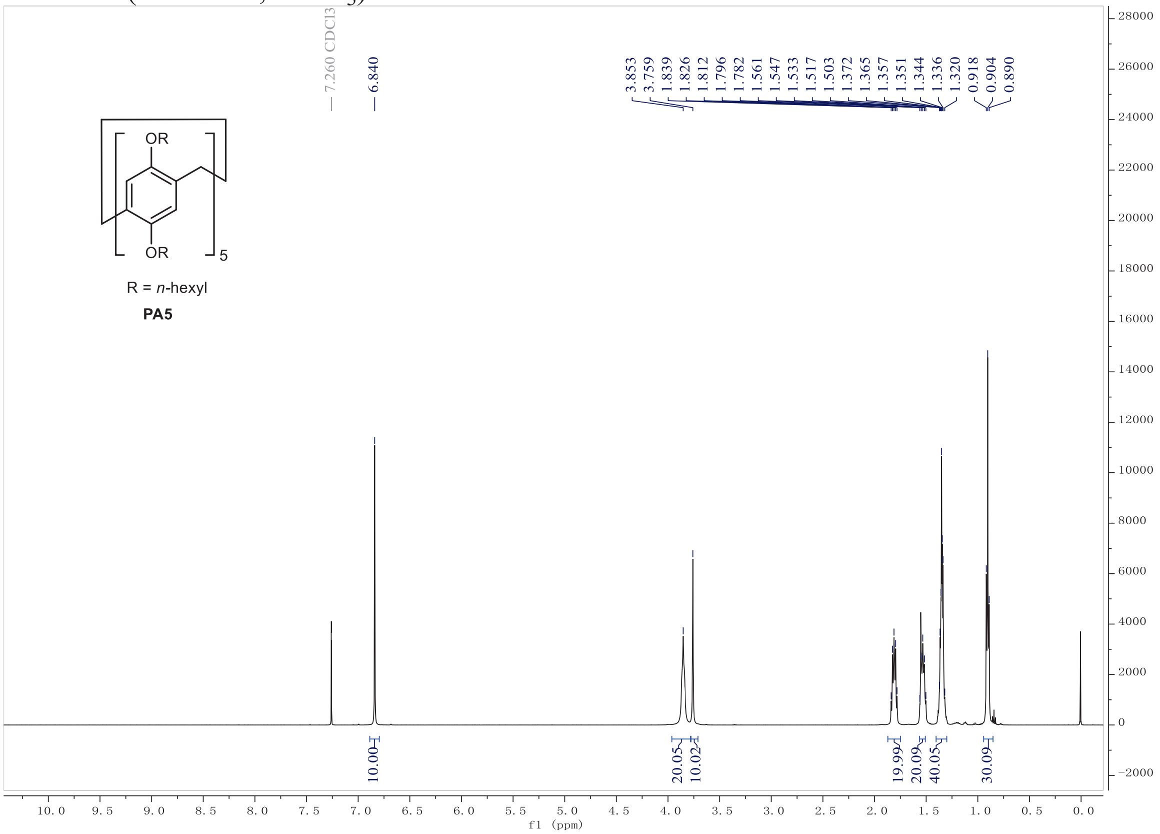

^{13}C NMR (126 MHz, $CDCl_3$)

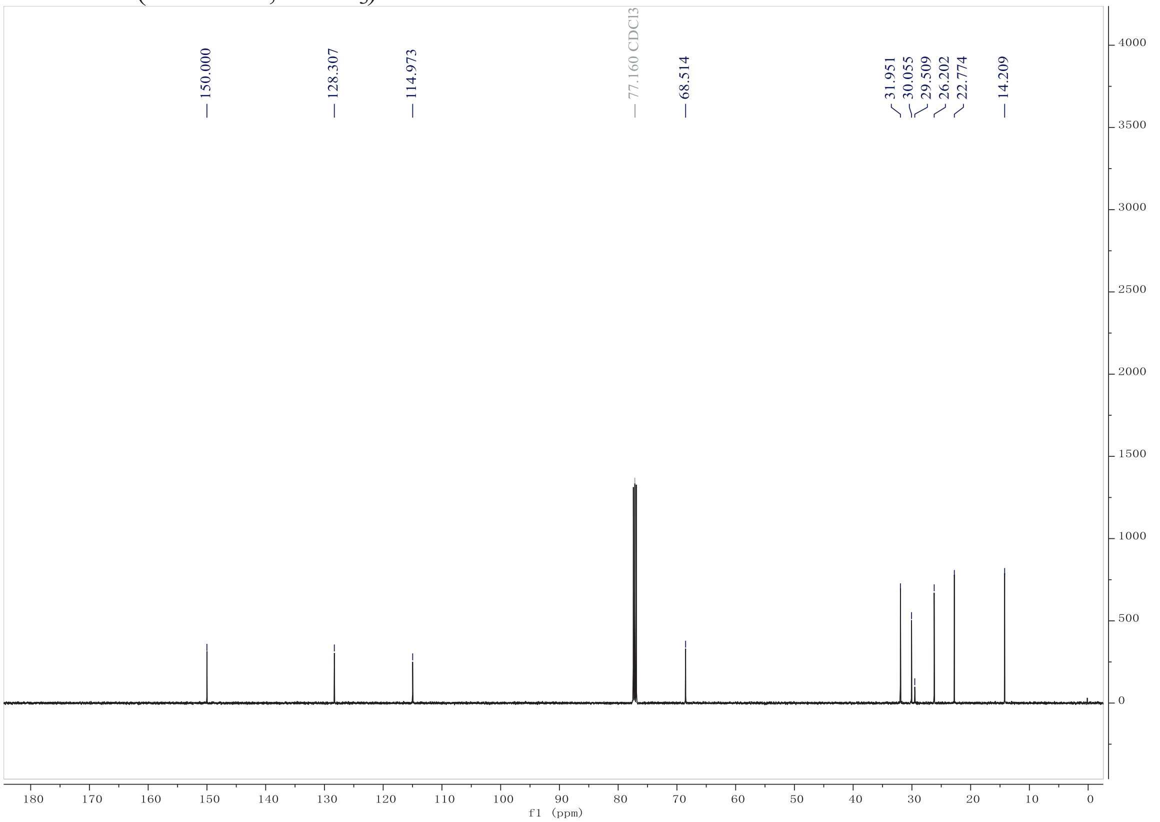

^{1}H NMR (500 MHz, $CDCl_3$)

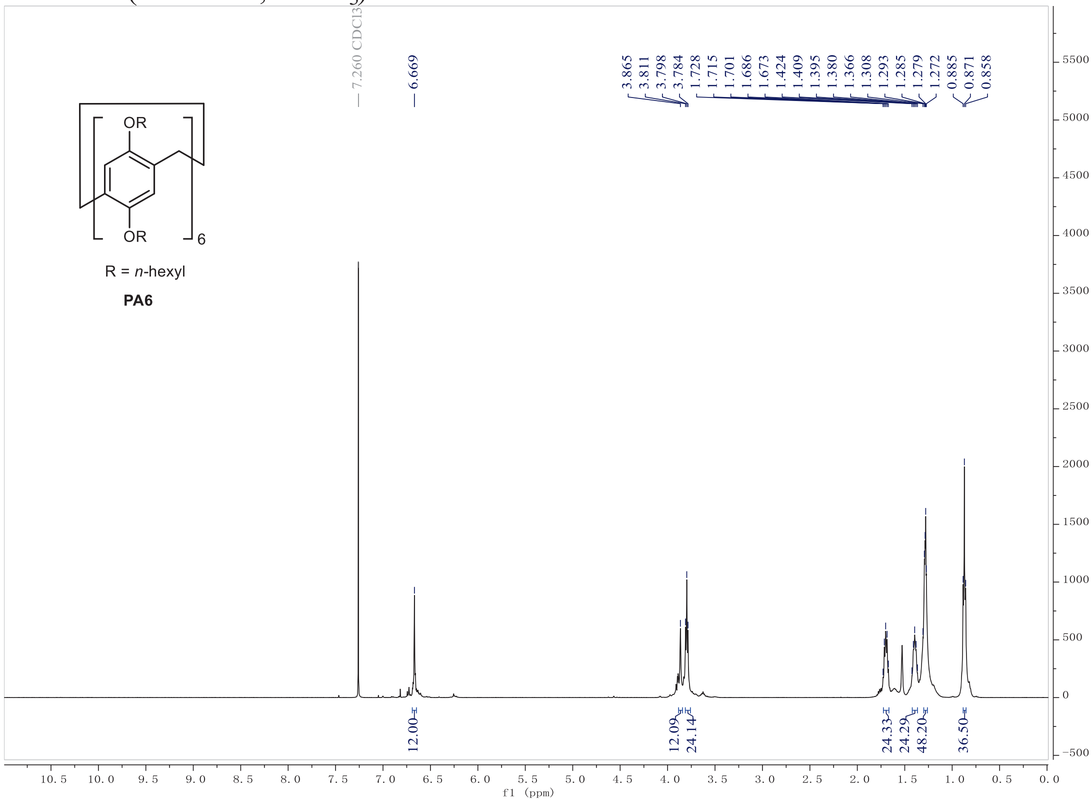

^{13}C NMR (126 MHz, $CDCl_3$)

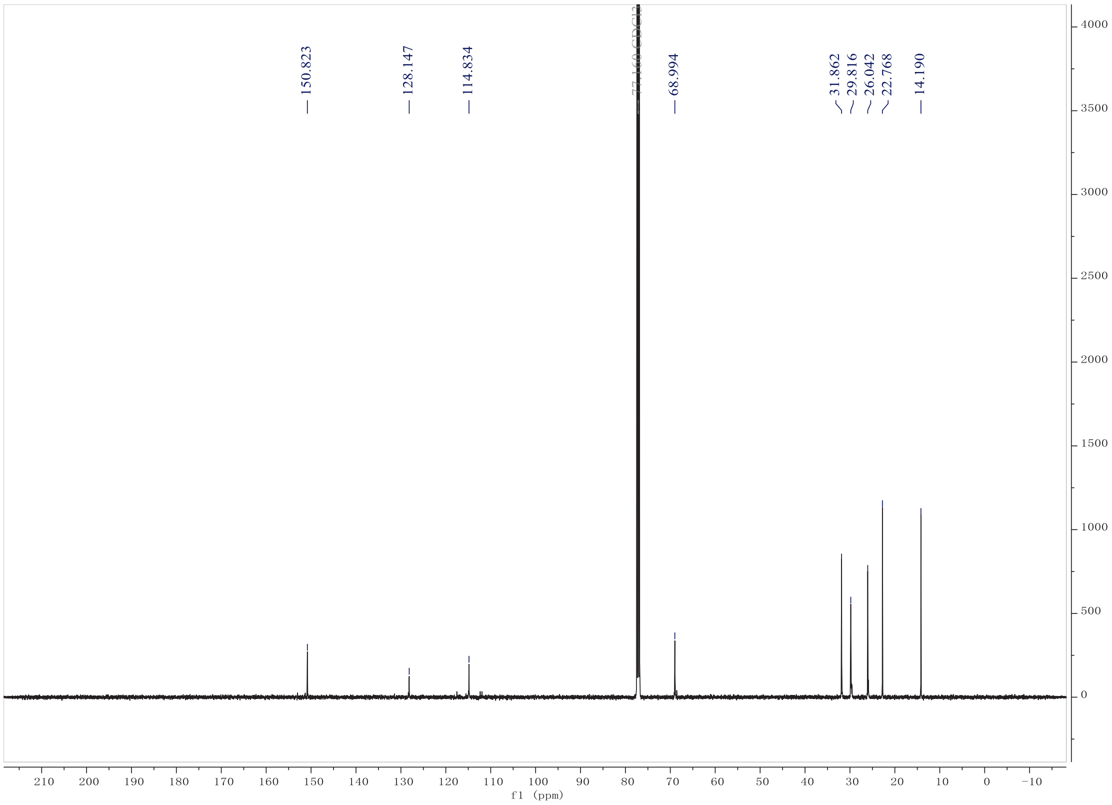

^{1}H NMR (500 MHz, $CDCl_3$)

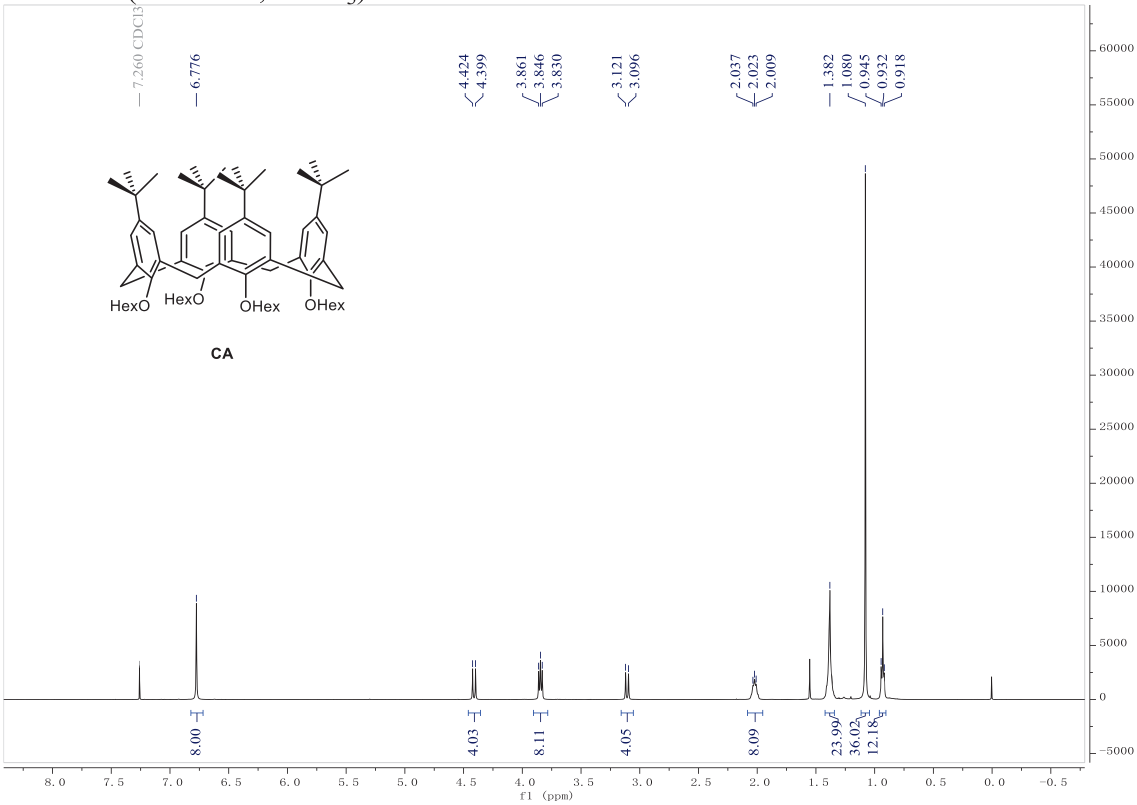

^{13}C NMR (126 MHz, $CDCl_3$)

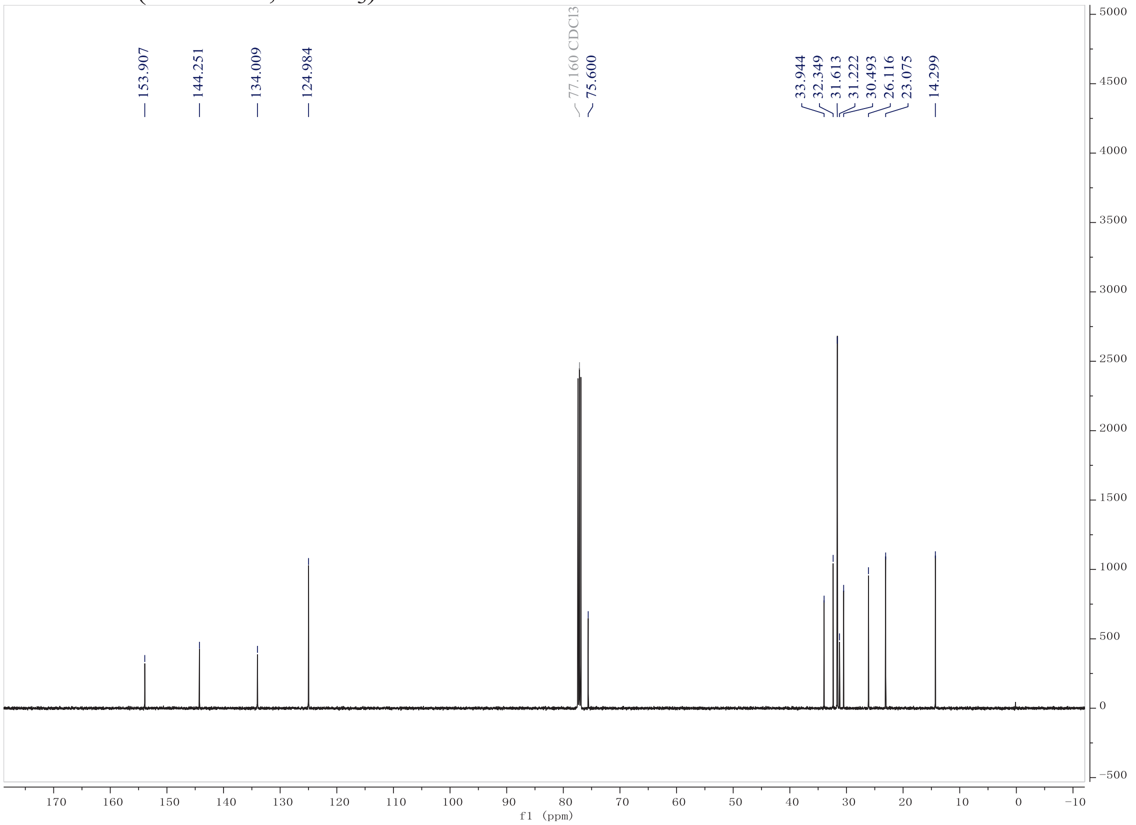

^{1}H NMR (500 MHz, $CDCl_3$)

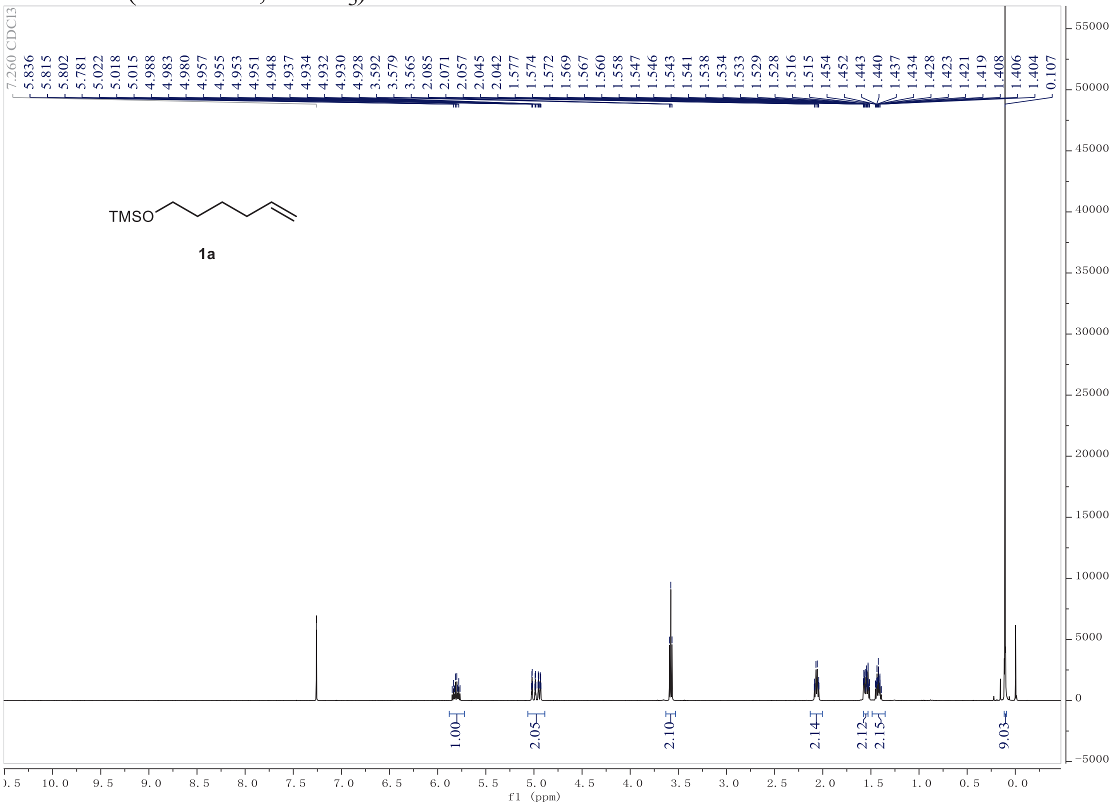

^{13}C NMR (126 MHz, $CDCl_3$)

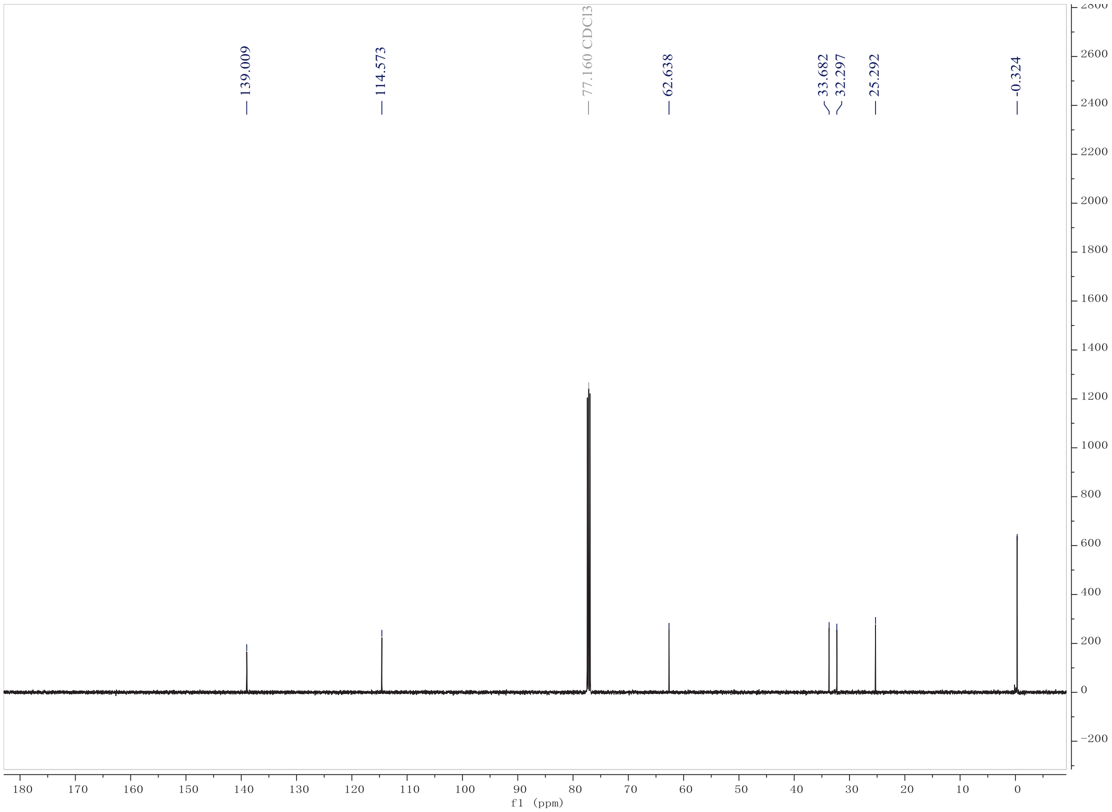

^{1}H NMR (500 MHz, $CDCl_3$)

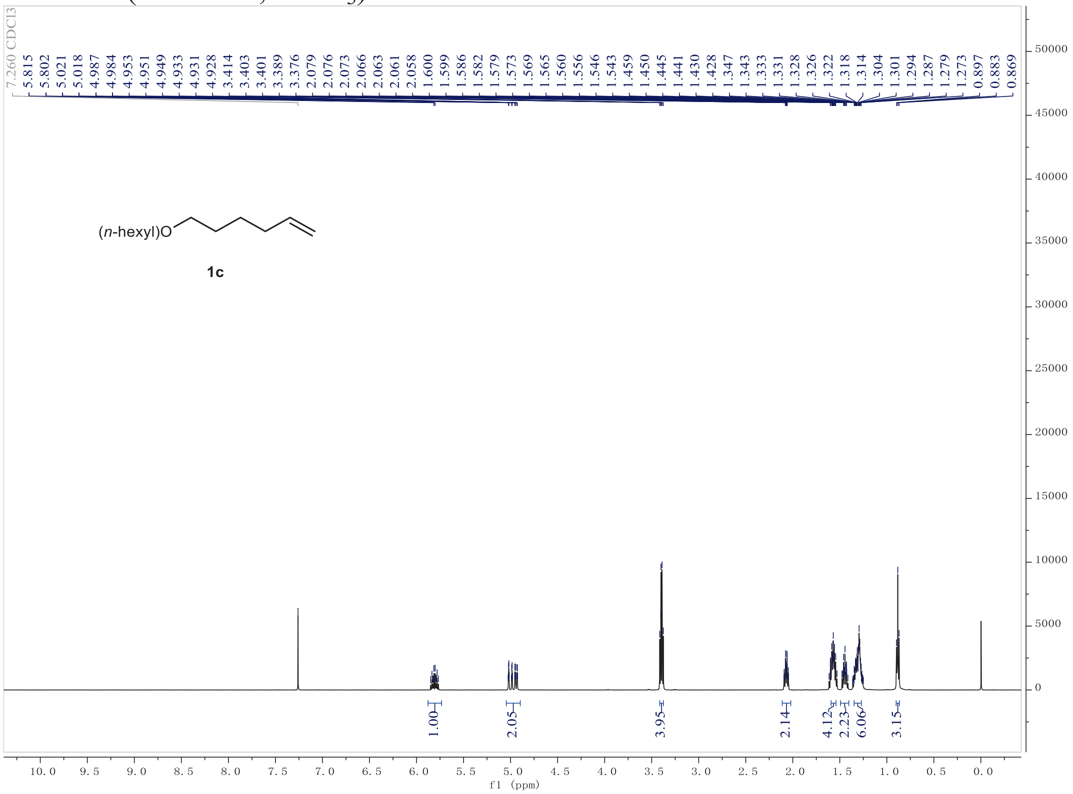

^{13}C NMR (126 MHz, $CDCl_3$)

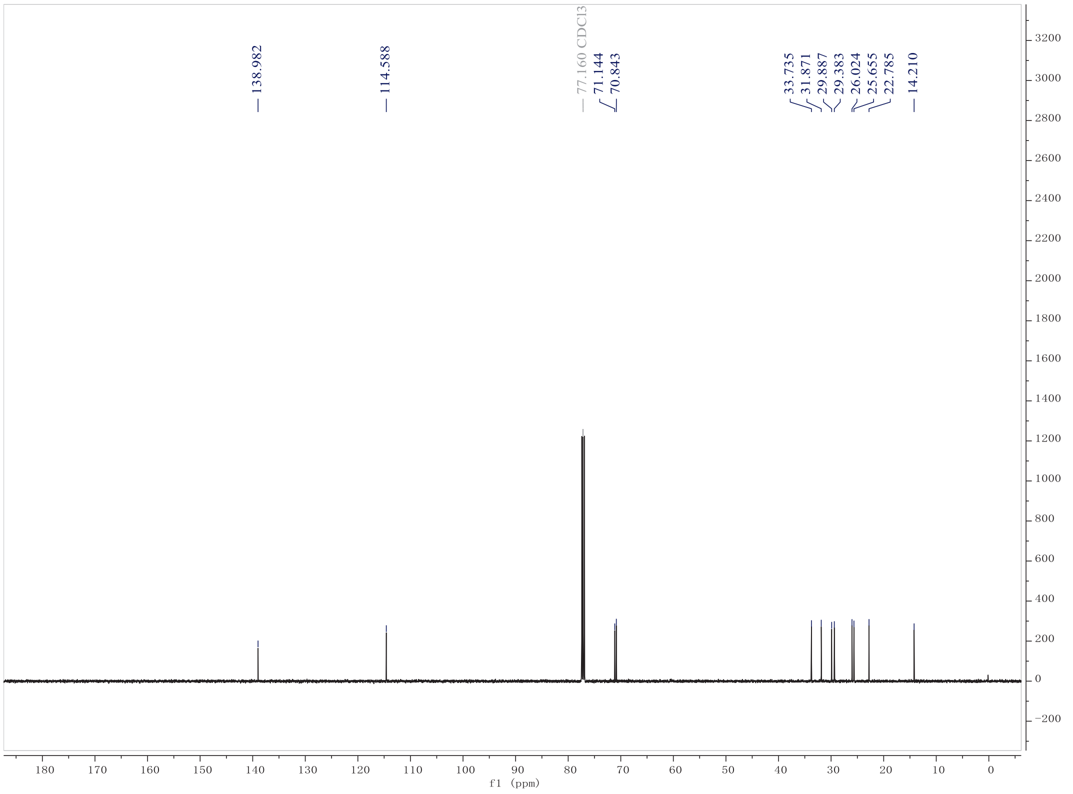

^{1}H NMR (500 MHz, $CDCl_3$)

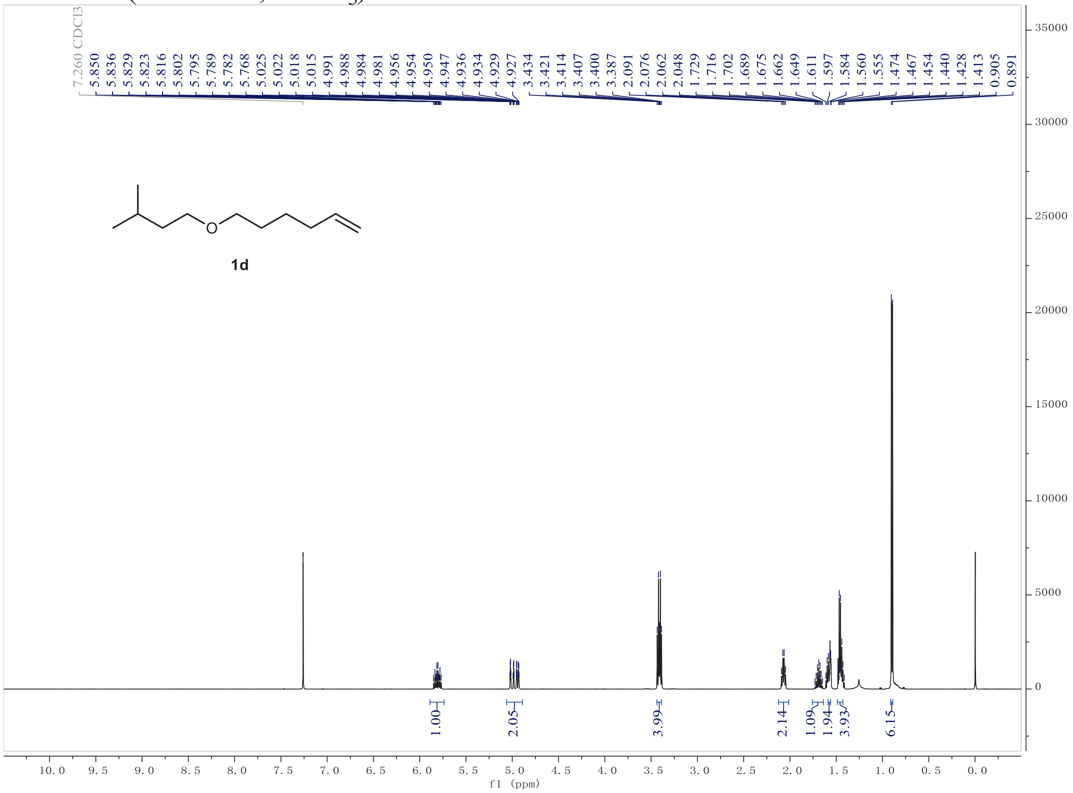

^{13}C NMR (126 MHz, $CDCl_3$)

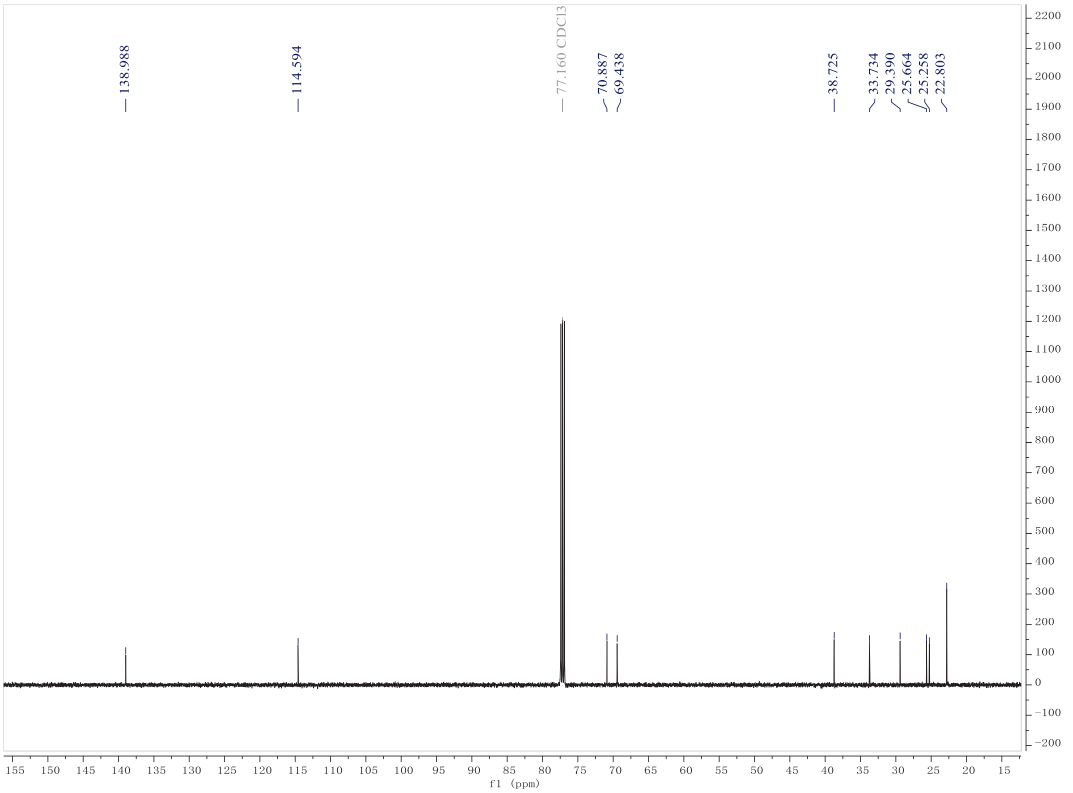

^{1}H NMR (500 MHz, $CDCl_3$)

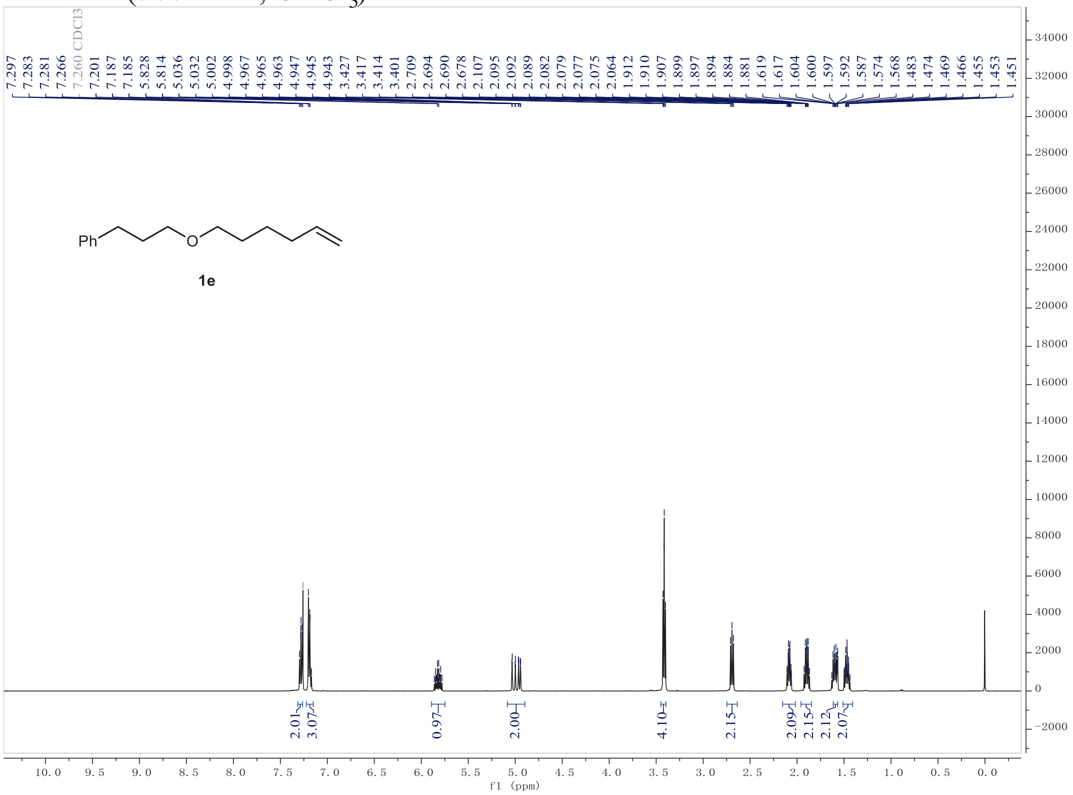

^{13}C NMR (126 MHz, $CDCl_3$)

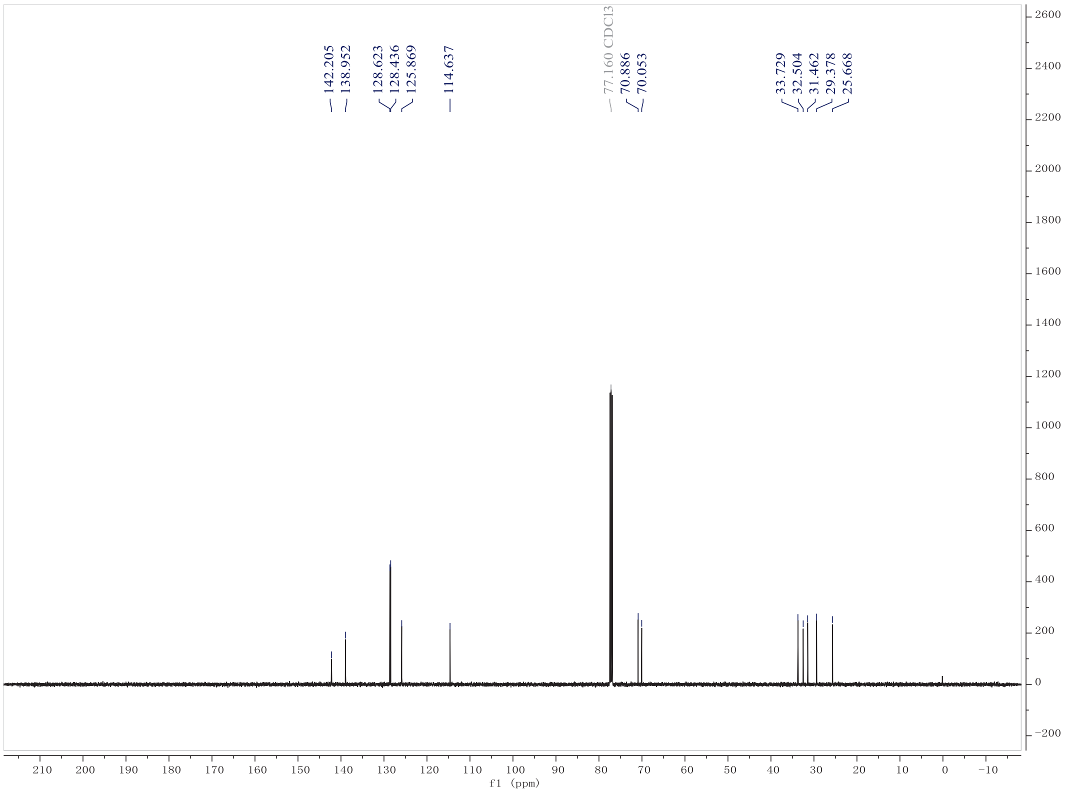

^{1}H NMR (400 MHz, $CDCl_3$)

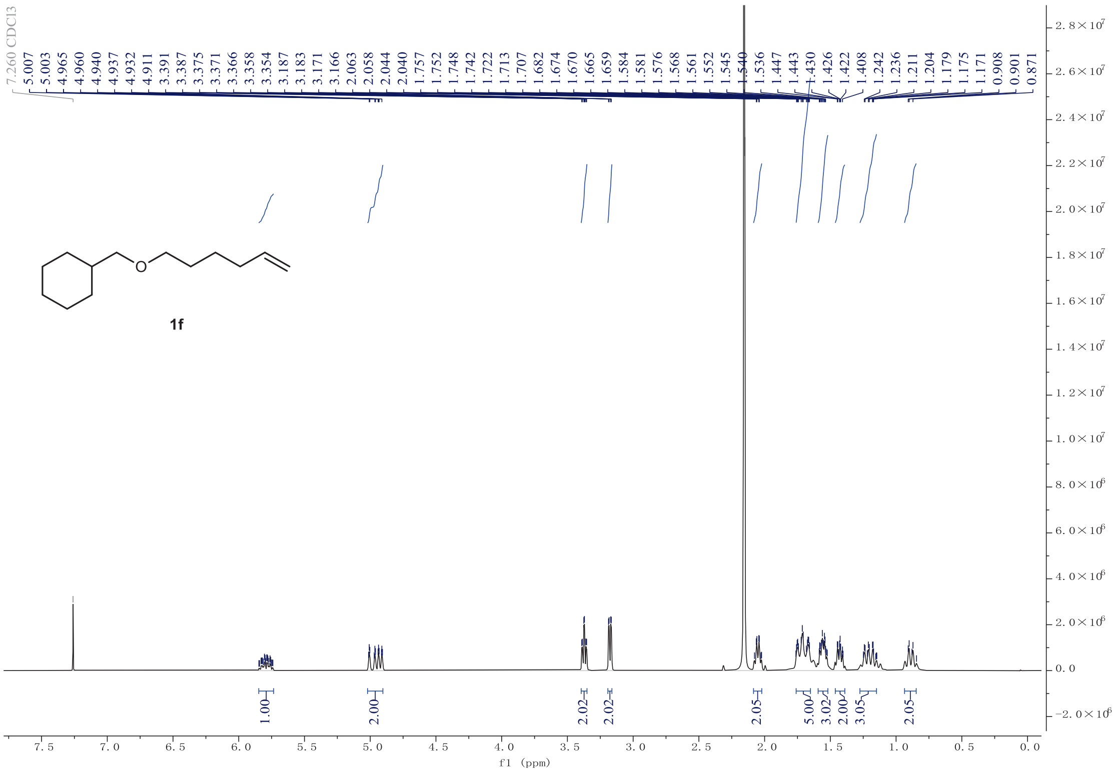

^{13}C NMR (100 MHz, $CDCl_3$)

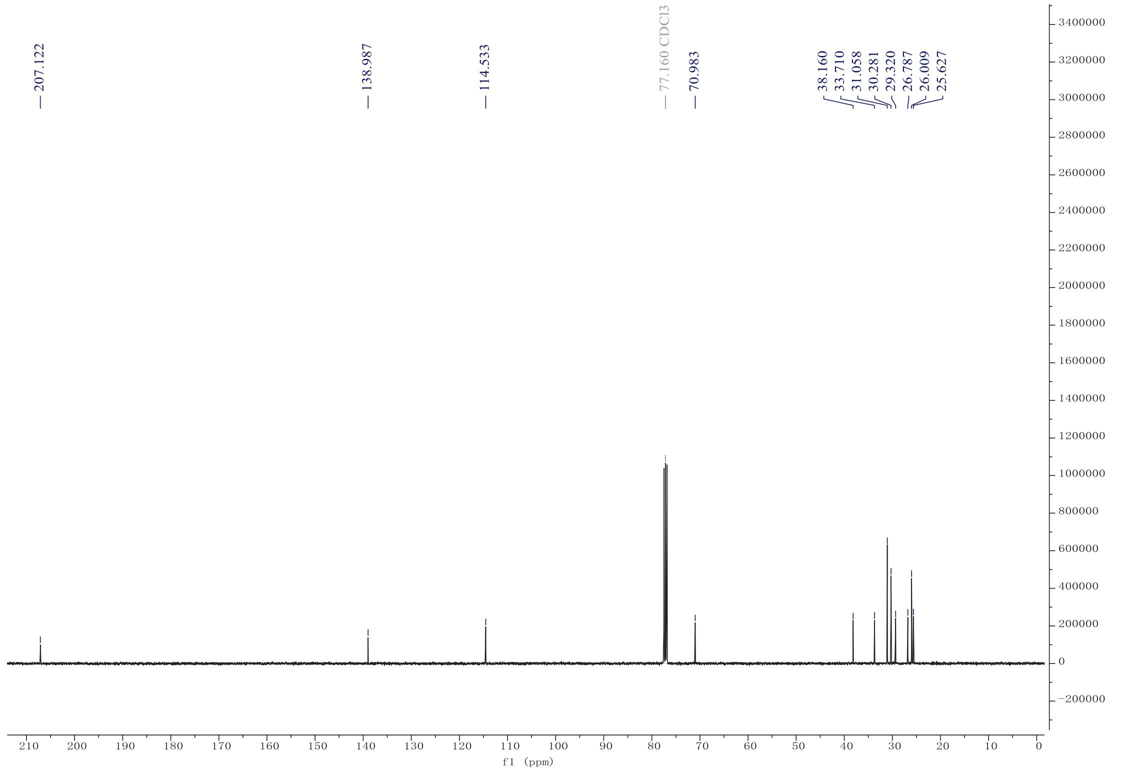

^{1}H NMR (500 MHz, $CDCl_3$)

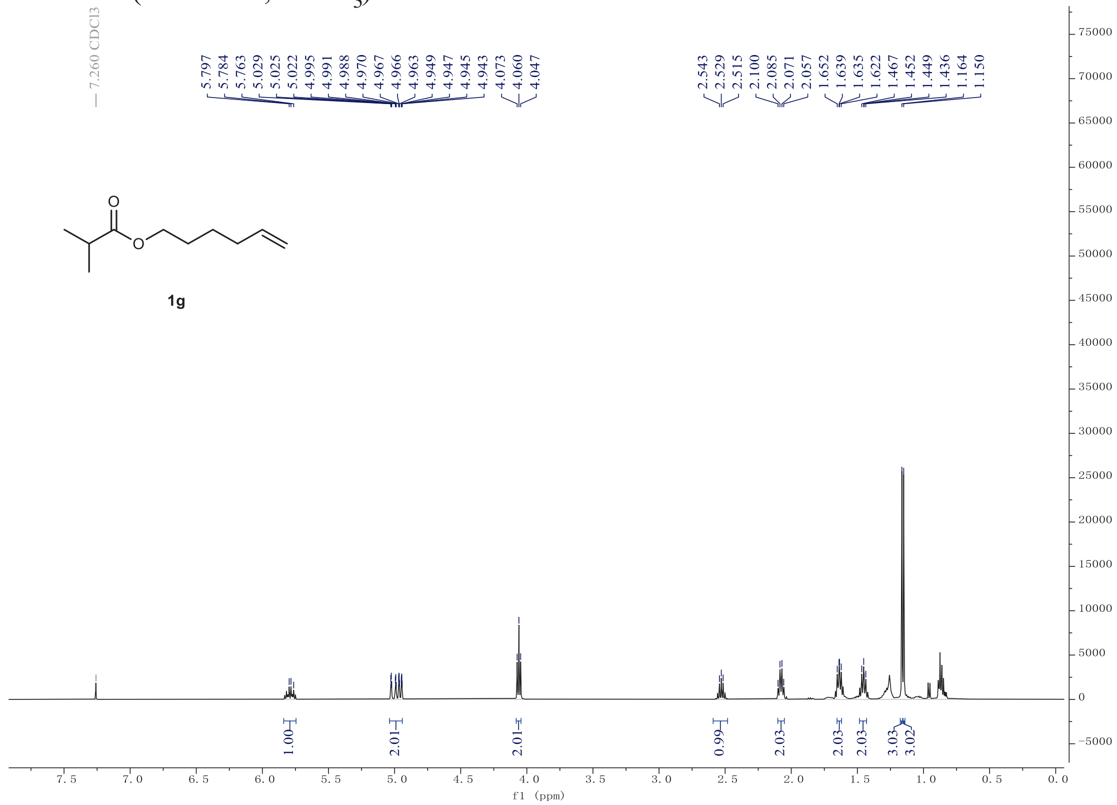

1g

^{13}C NMR (126 MHz, $CDCl_3$)

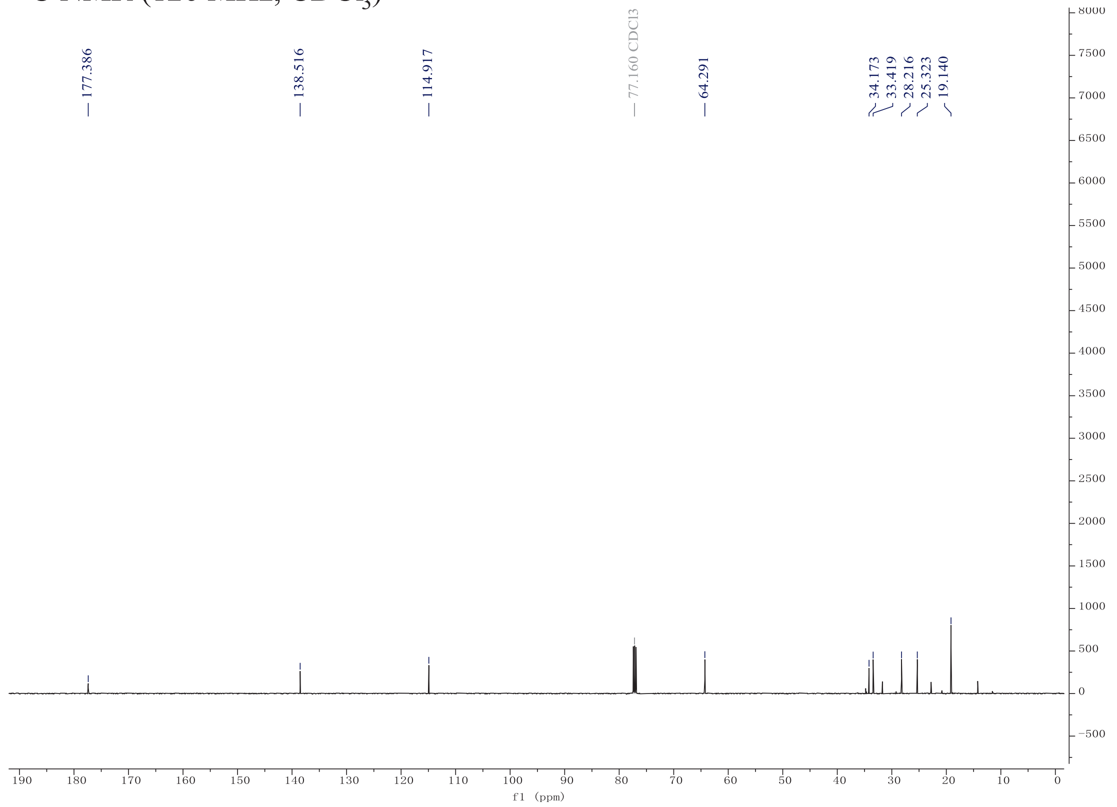

^{1}H NMR (400 MHz, $CDCl_3$)

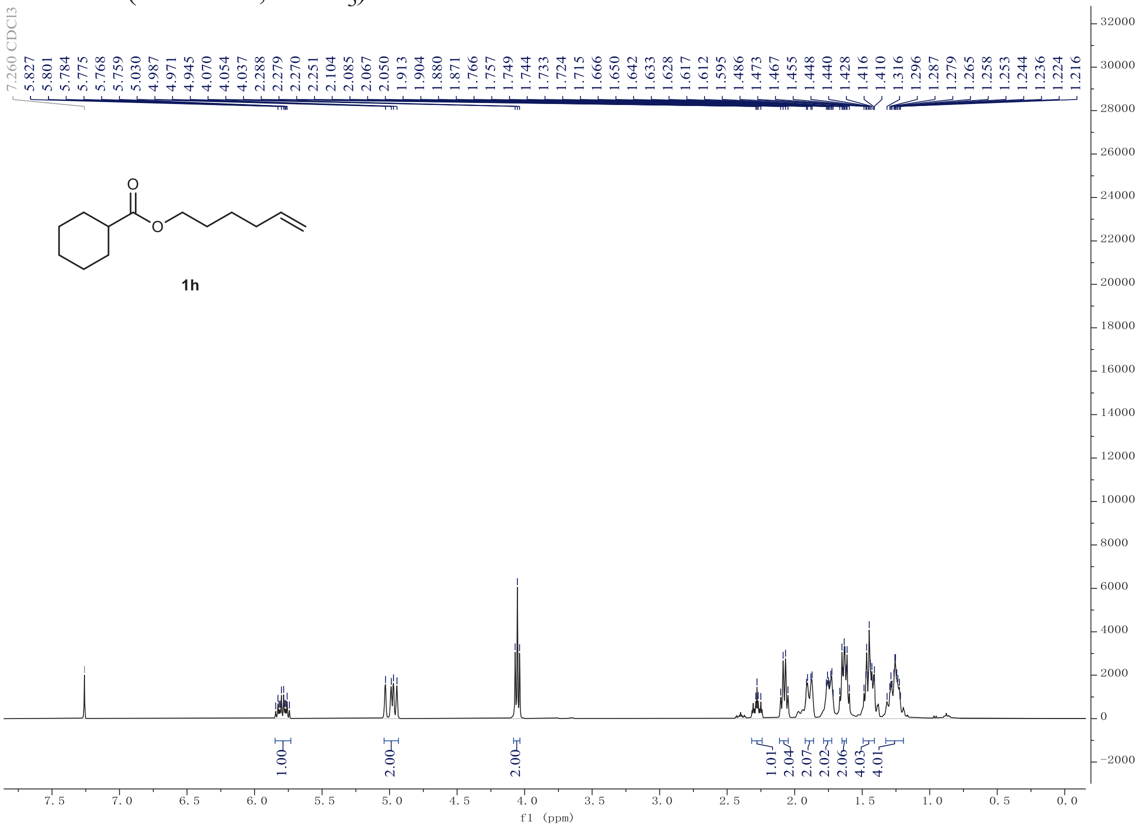

^{13}C NMR (100 MHz, $CDCl_3$)

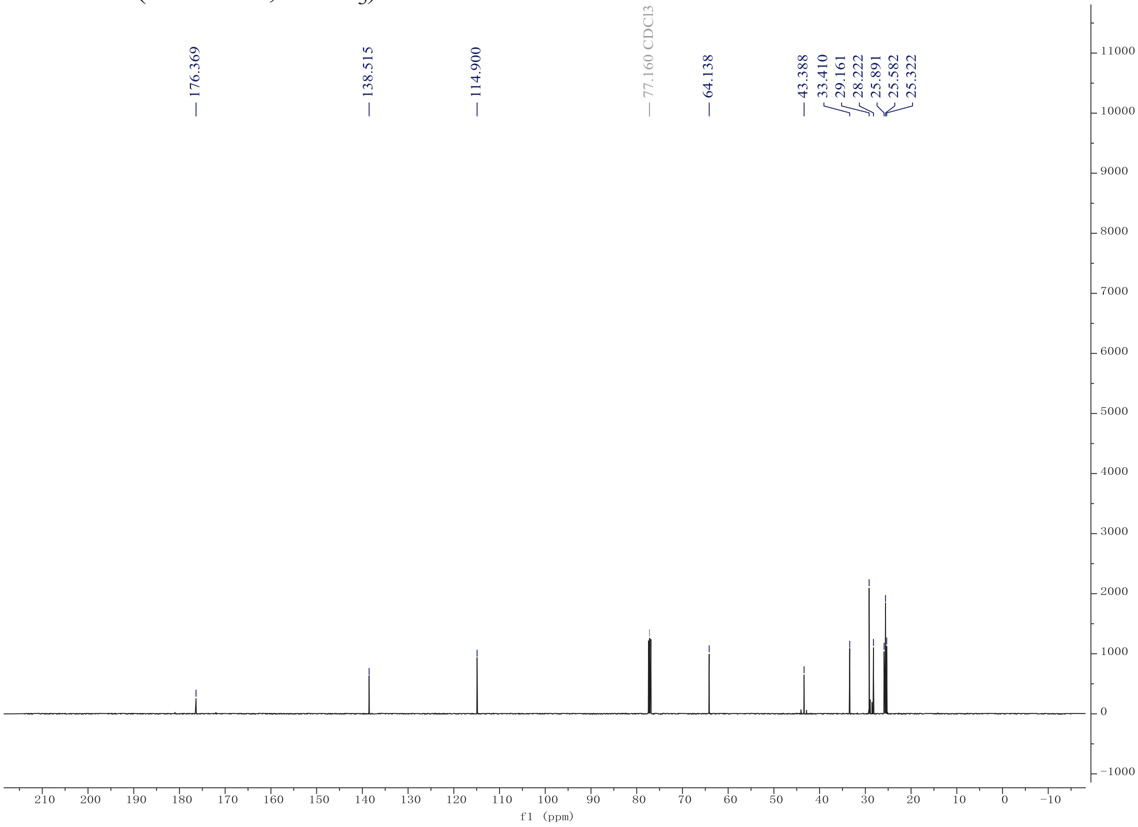

^{1}H NMR (500 MHz, $CDCl_3$)

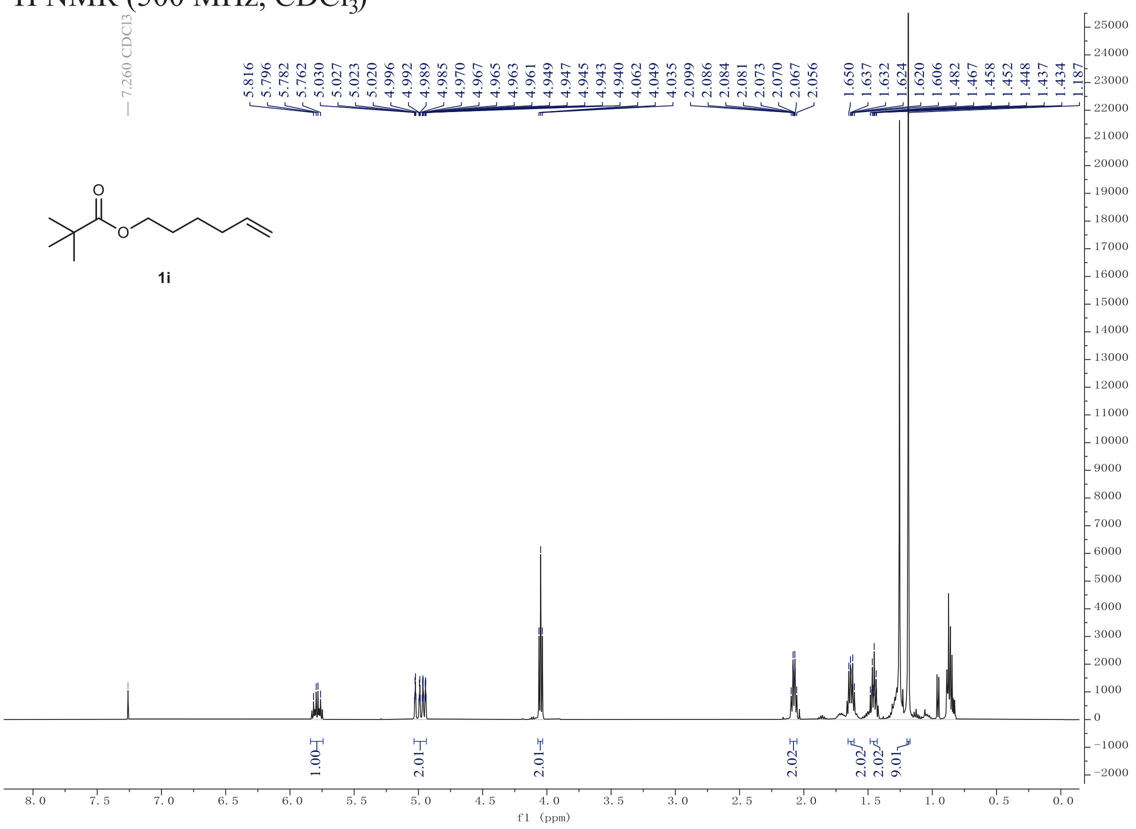

^{13}C NMR (126 MHz, $CDCl_3$)

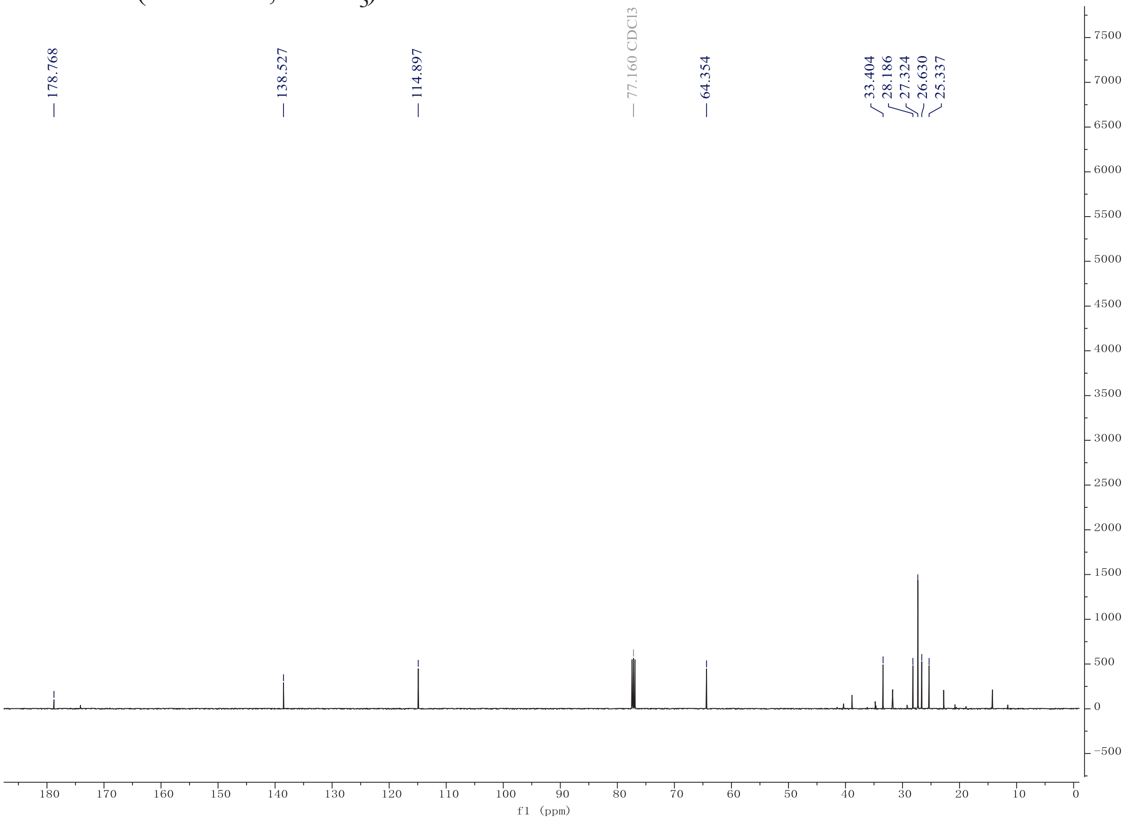

^{1}H NMR (400 MHz, $CDCl_3$)

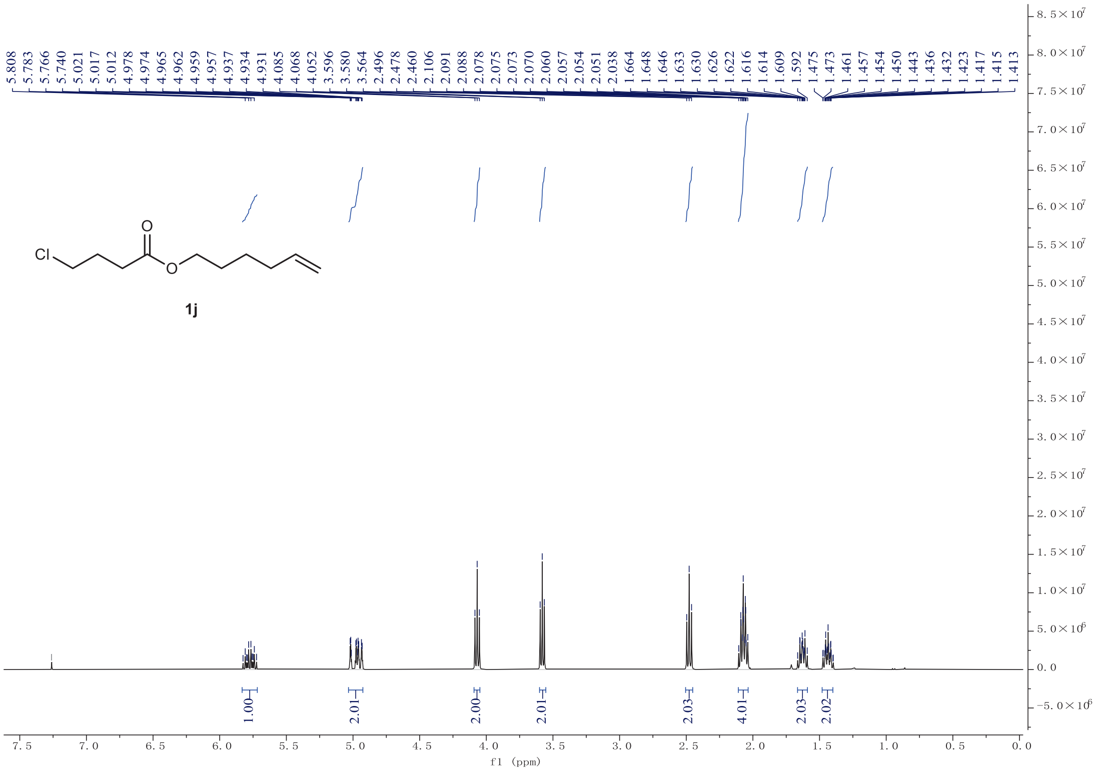

^{13}C NMR (100 MHz, $CDCl_3$)

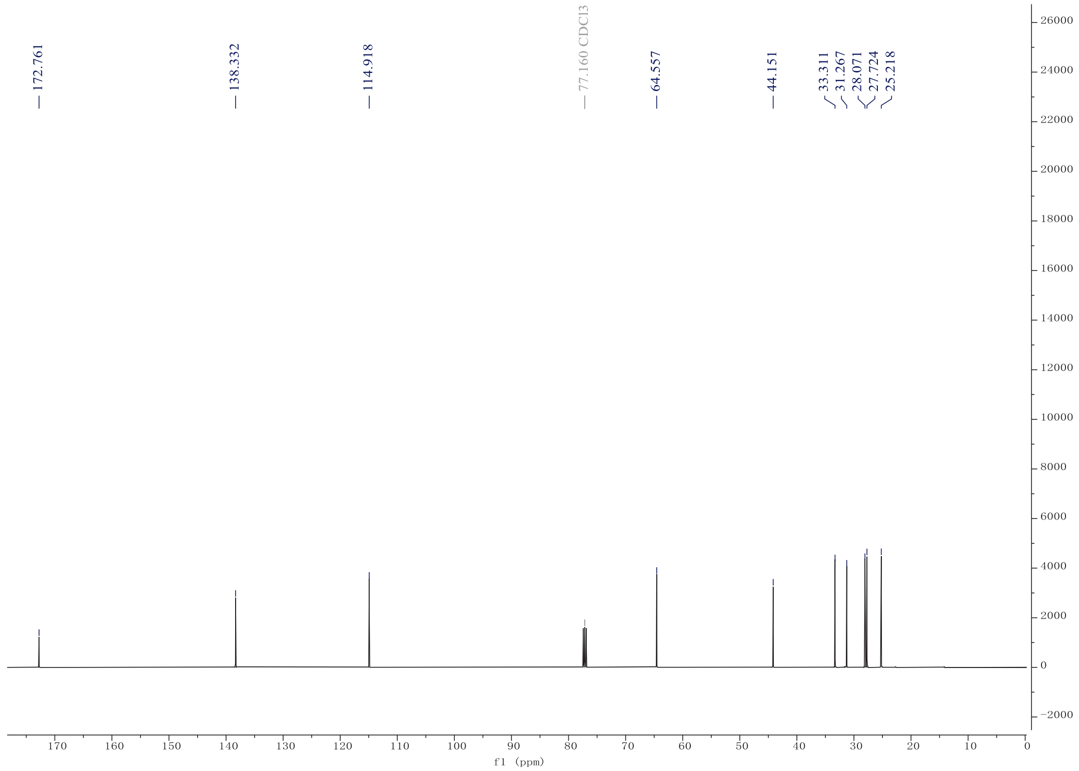

^1H NMR (500 MHz, $CDCl_3$)

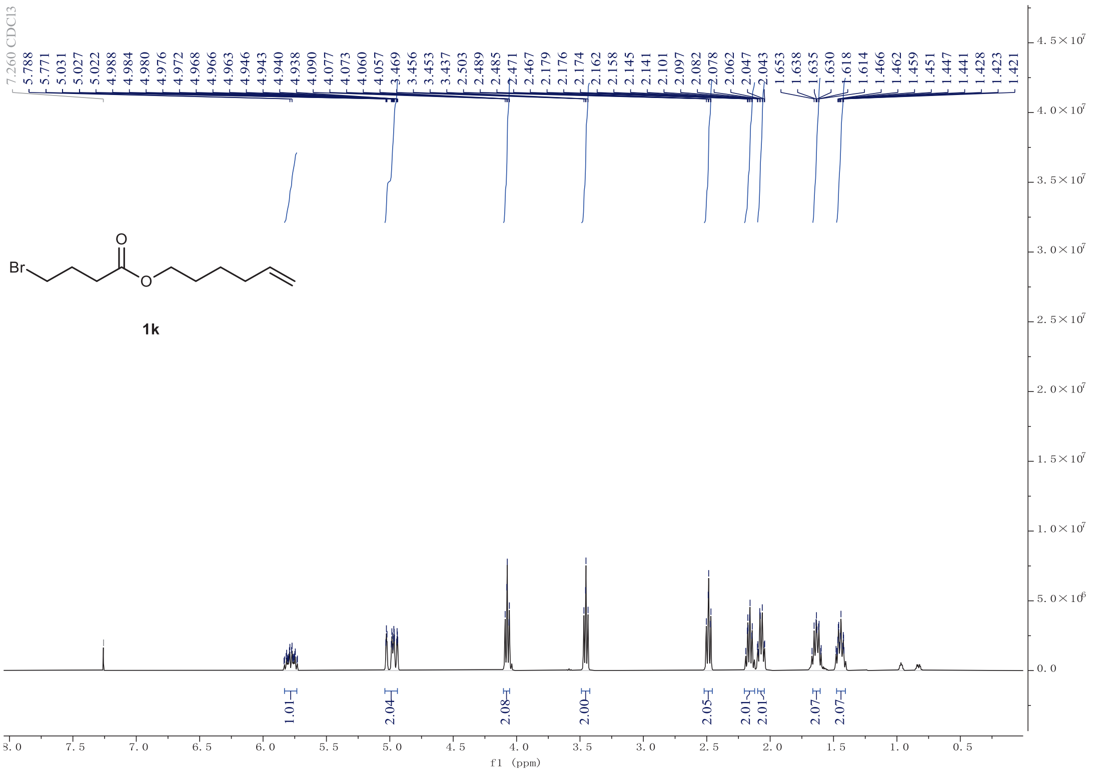

^{13}C NMR (126 MHz, $CDCl_3$)

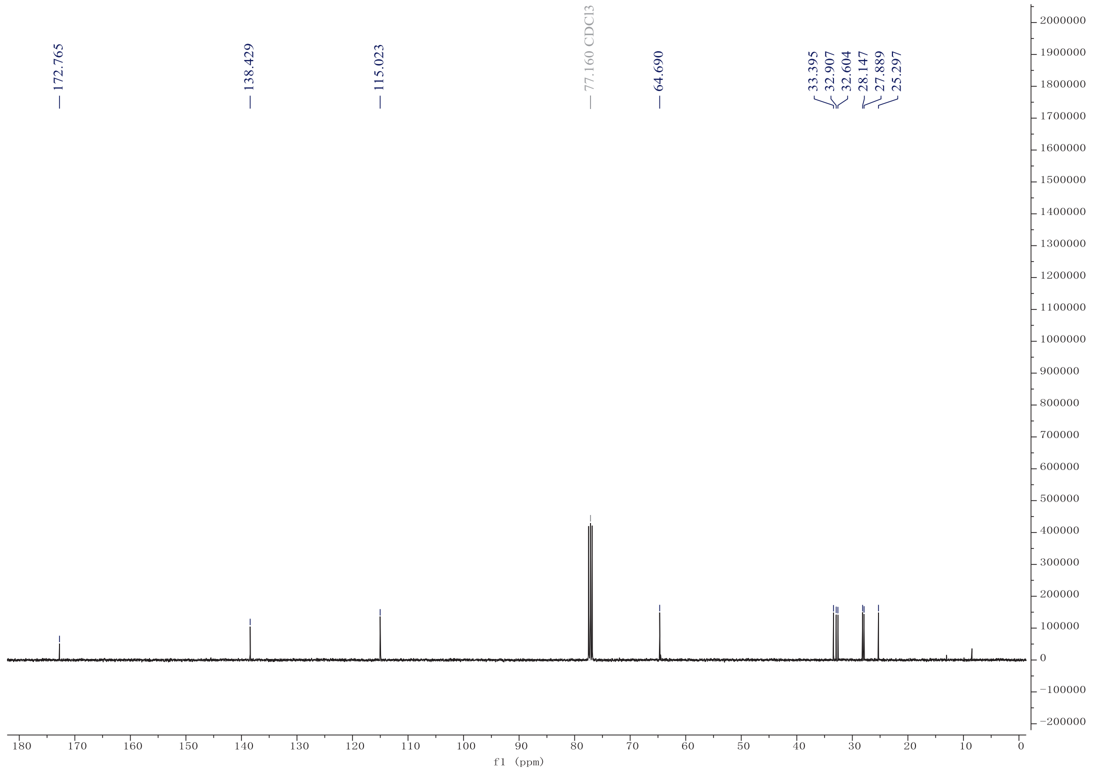

^{1}H NMR (500 MHz, $CDCl_3$)

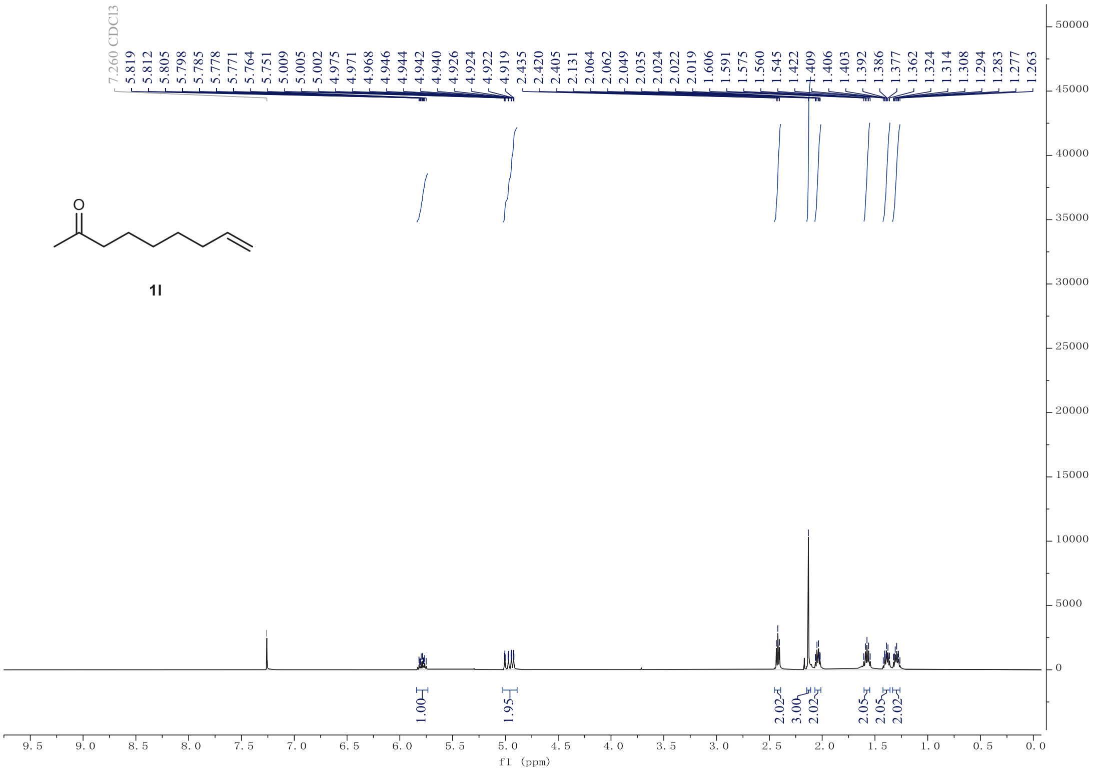

^{13}C NMR (126 MHz, $CDCl_3$)

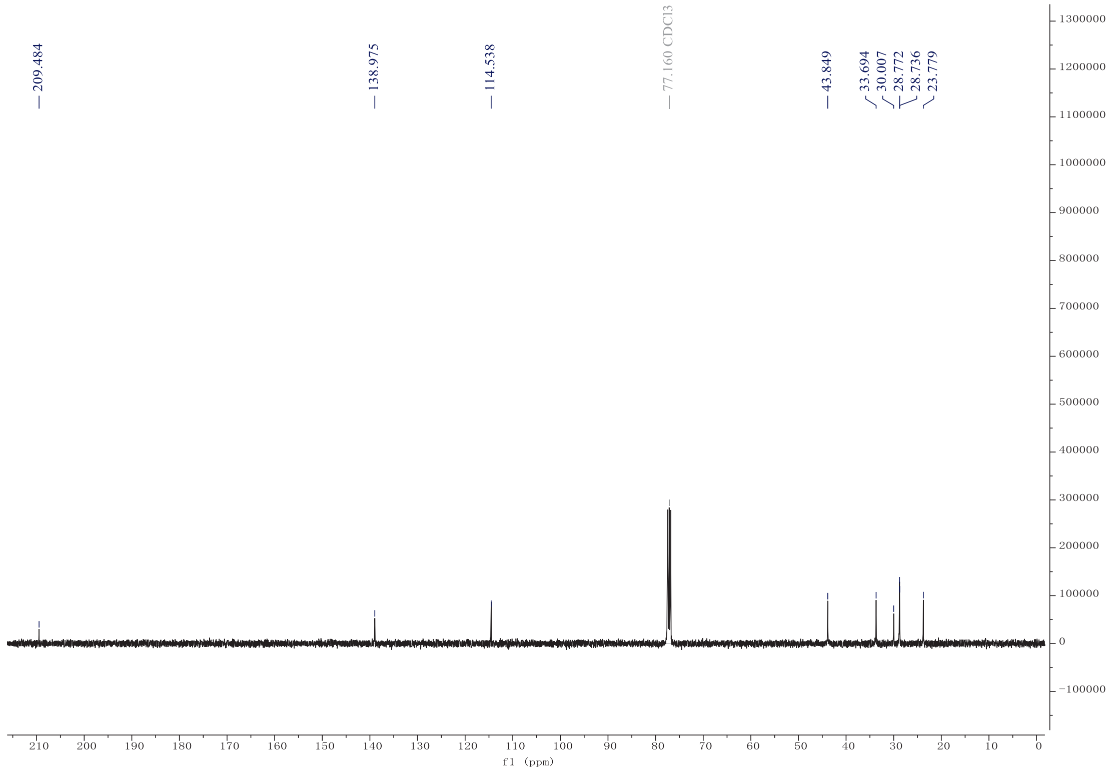

^{1}H NMR (500 MHz, $CDCl_3$)

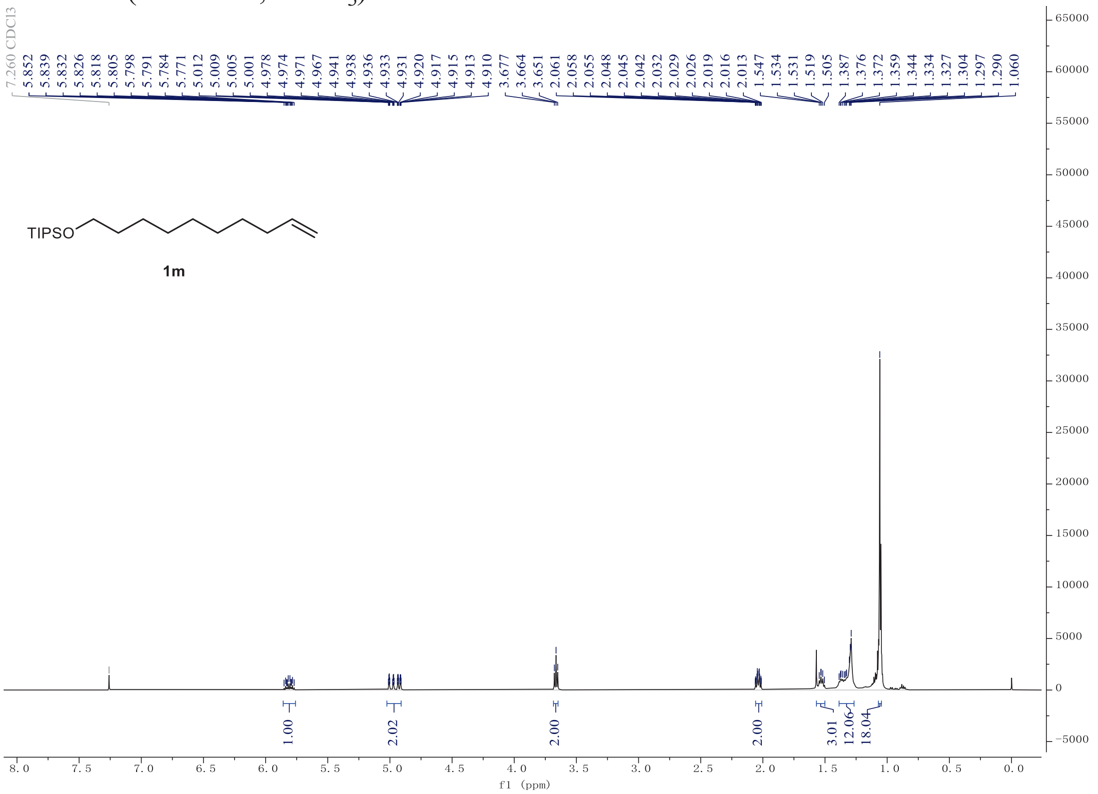

^{13}C NMR (126 MHz, $CDCl_3$)

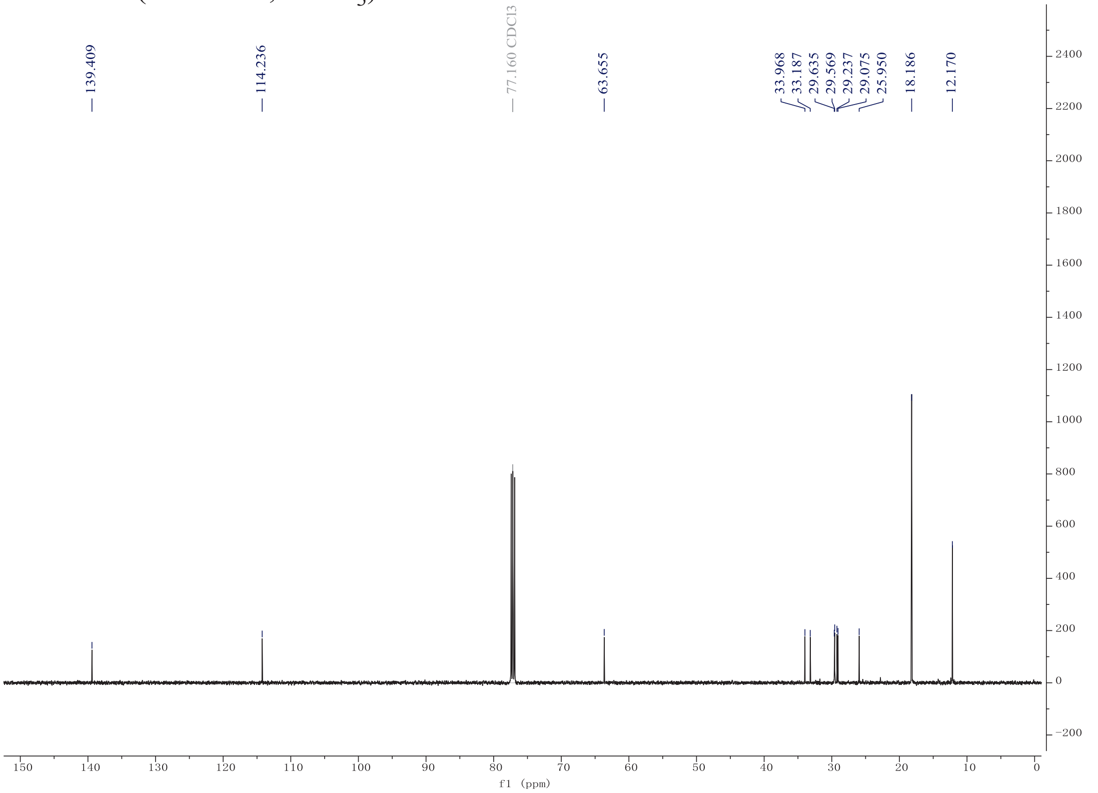

^{1}H NMR (500 MHz, $CDCl_3$)

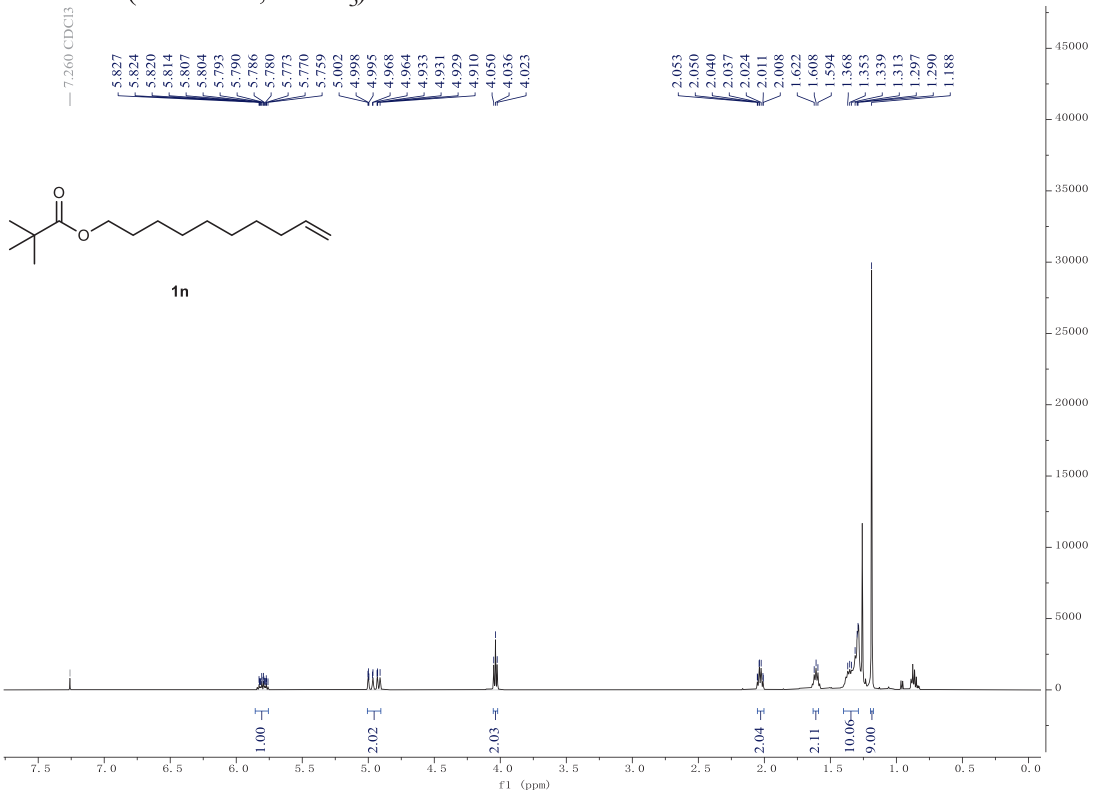

^{13}C NMR (126 MHz, $CDCl_3$)

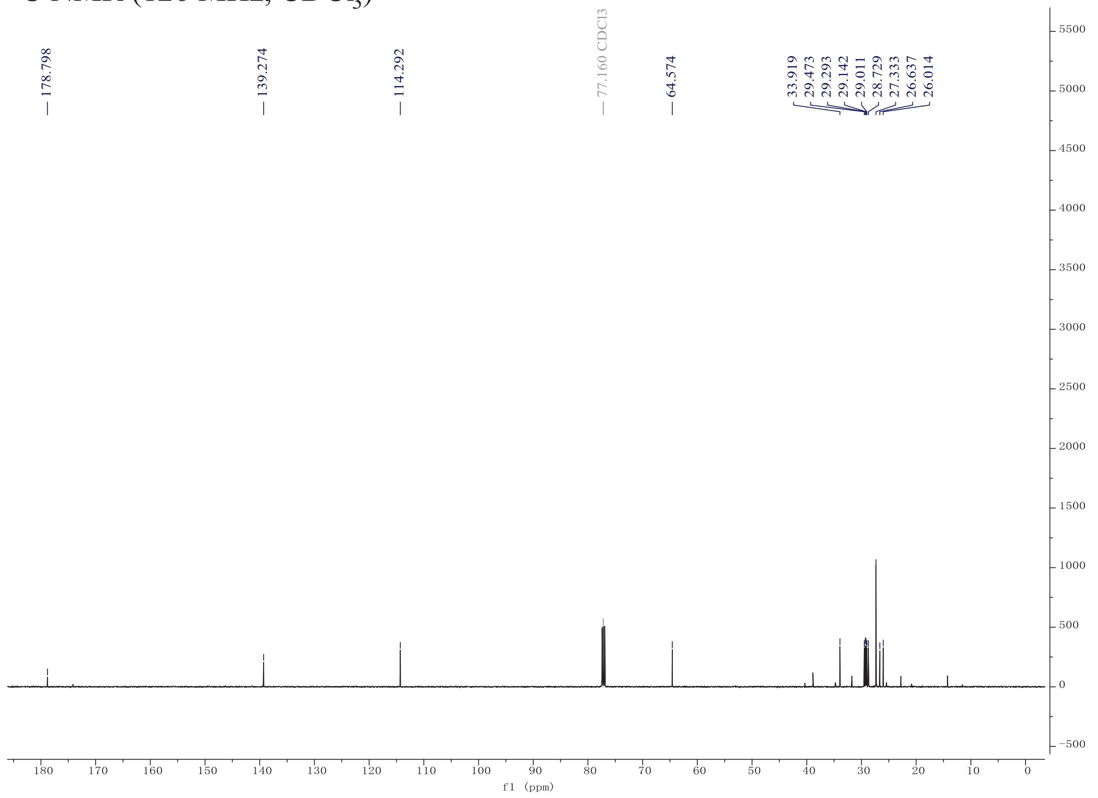

^{1}H NMR (500 MHz, $CDCl_3$)

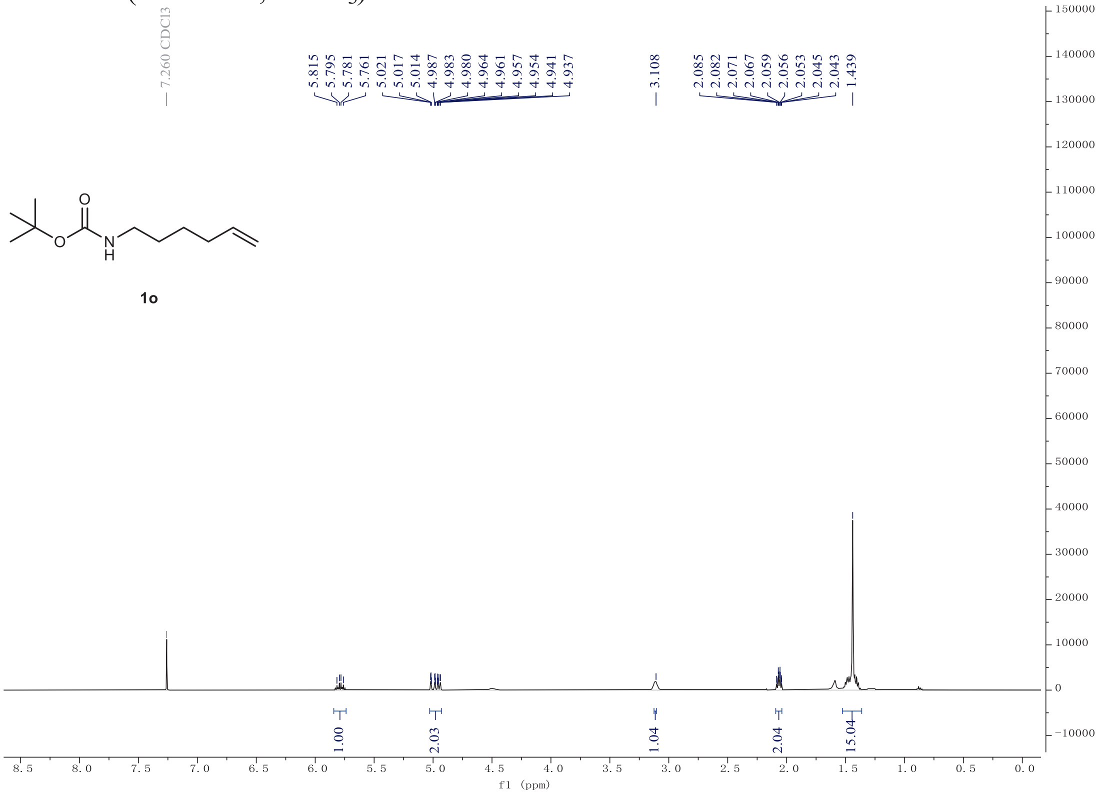

1o

^{13}C NMR (126 MHz, $CDCl_3$)

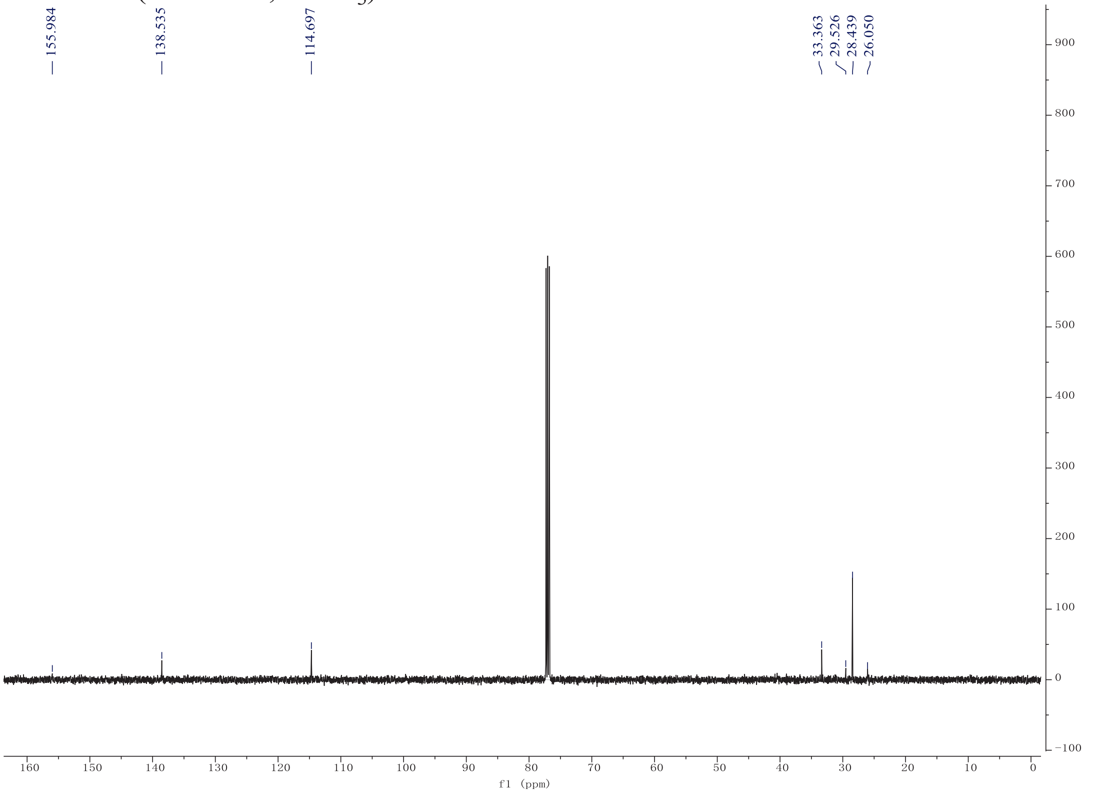

^{1}H NMR (500 MHz, $CDCl_3$)

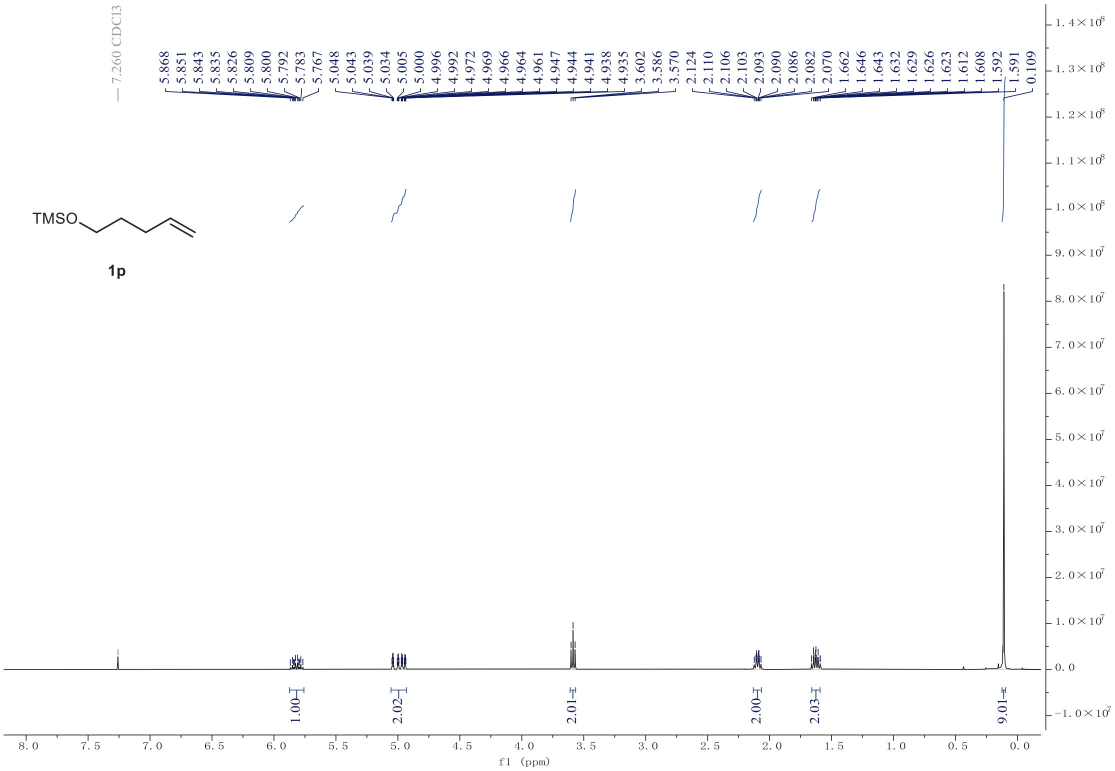

^{13}C NMR (126 MHz, $CDCl_3$)

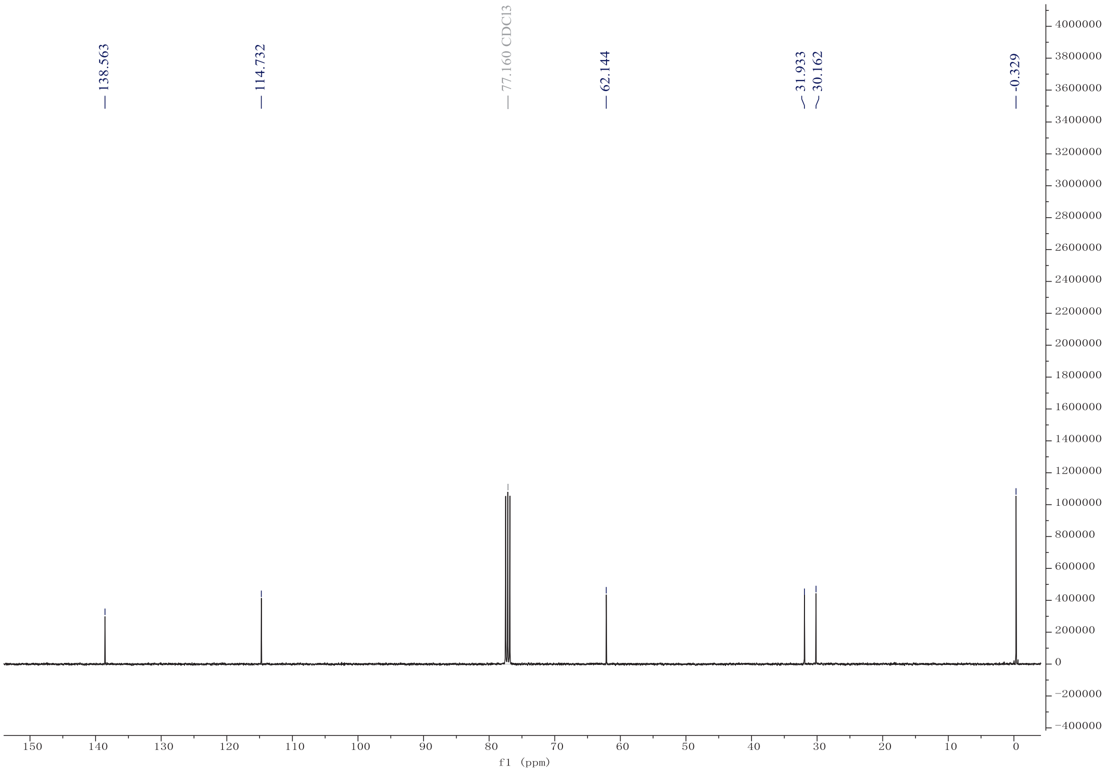

^{1}H NMR (500 MHz, $CDCl_3$)

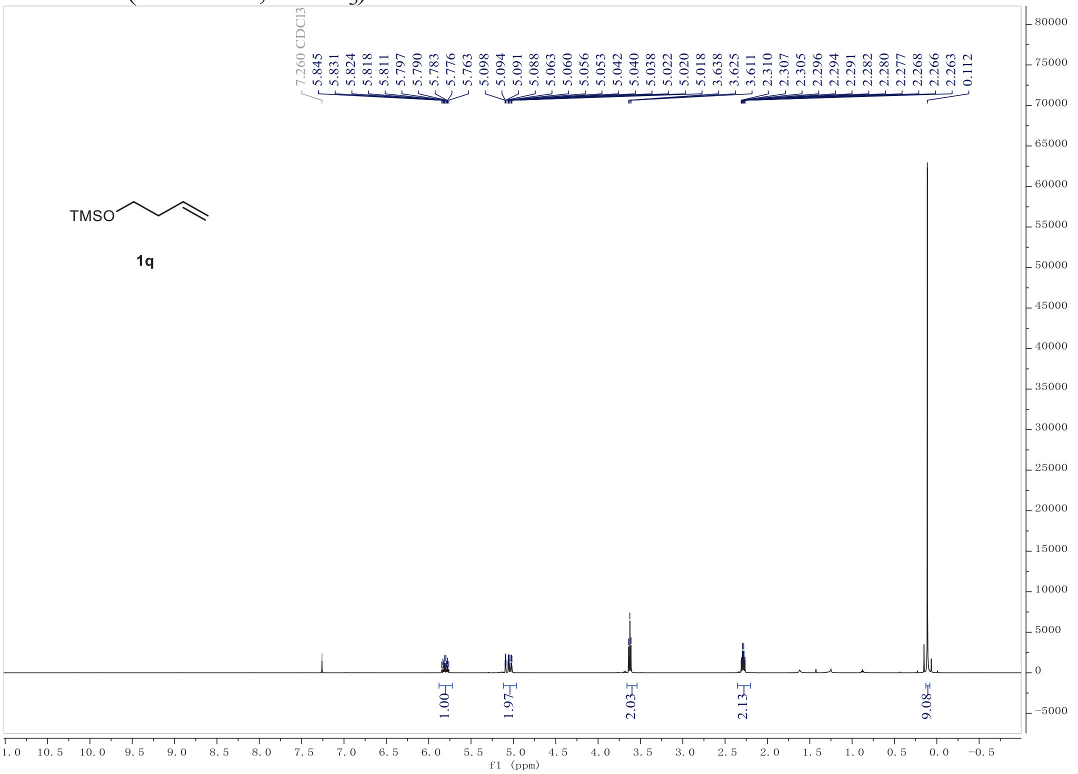

^{13}C NMR (126 MHz, $CDCl_3$)

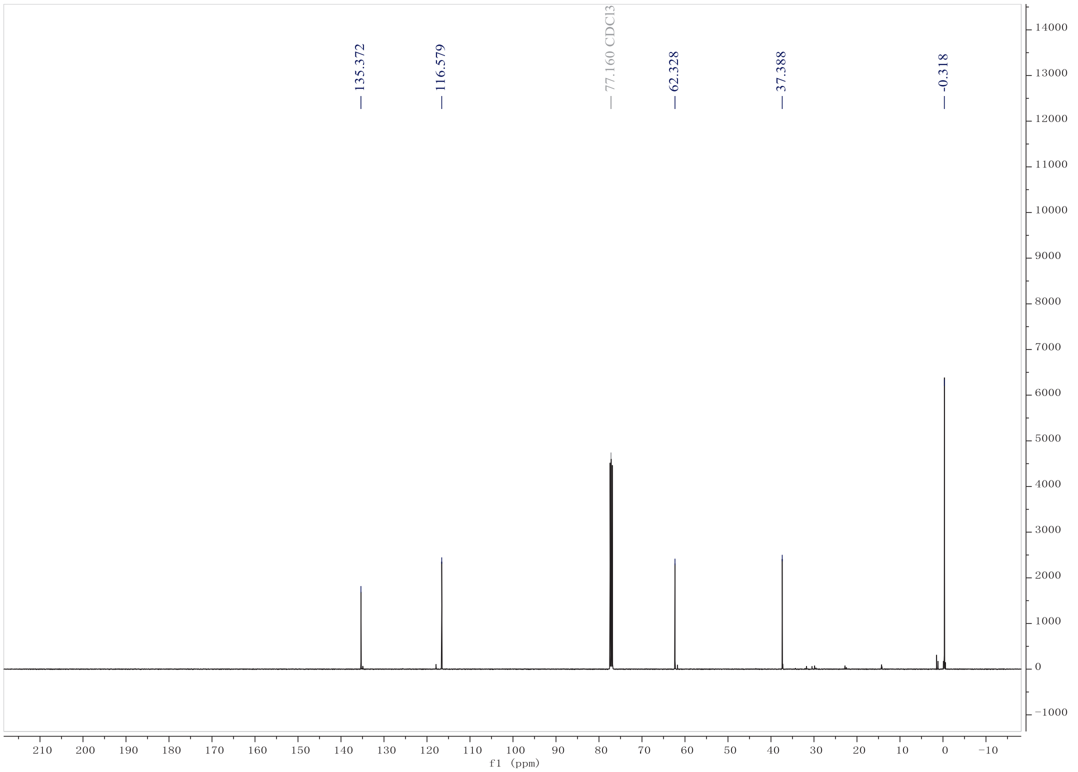

^{1}H NMR (500 MHz, $CDCl_3$)

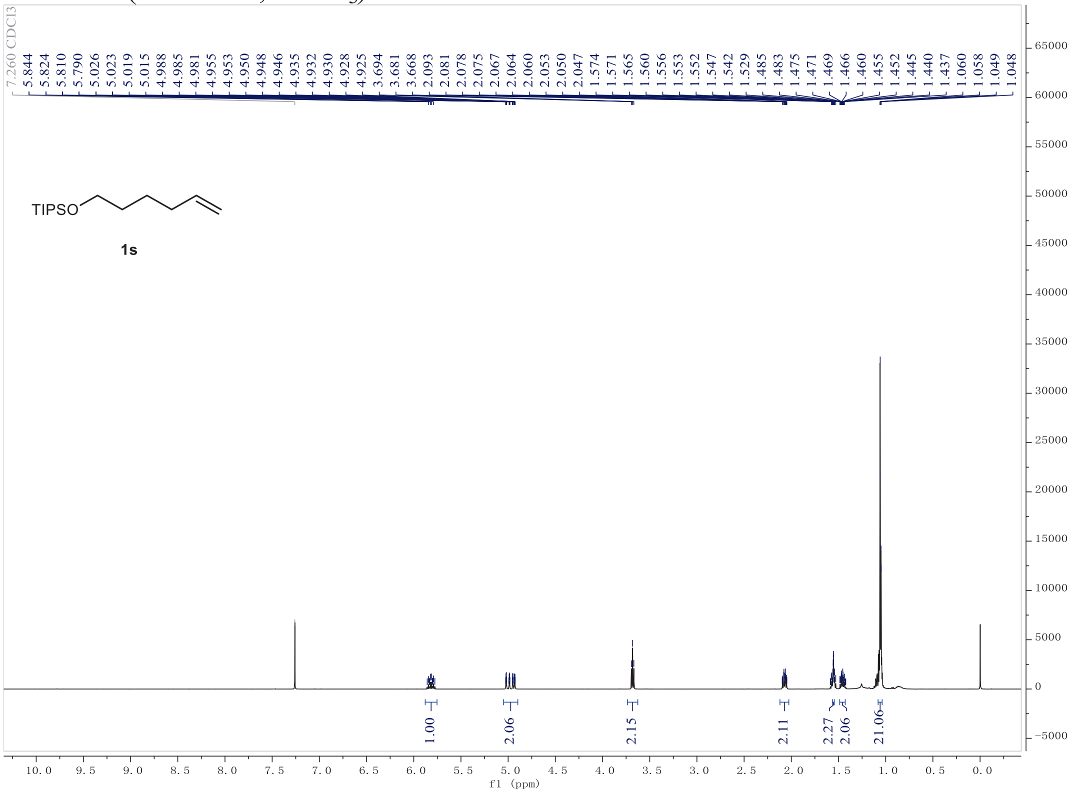

^{13}C NMR (126 MHz, $CDCl_3$)

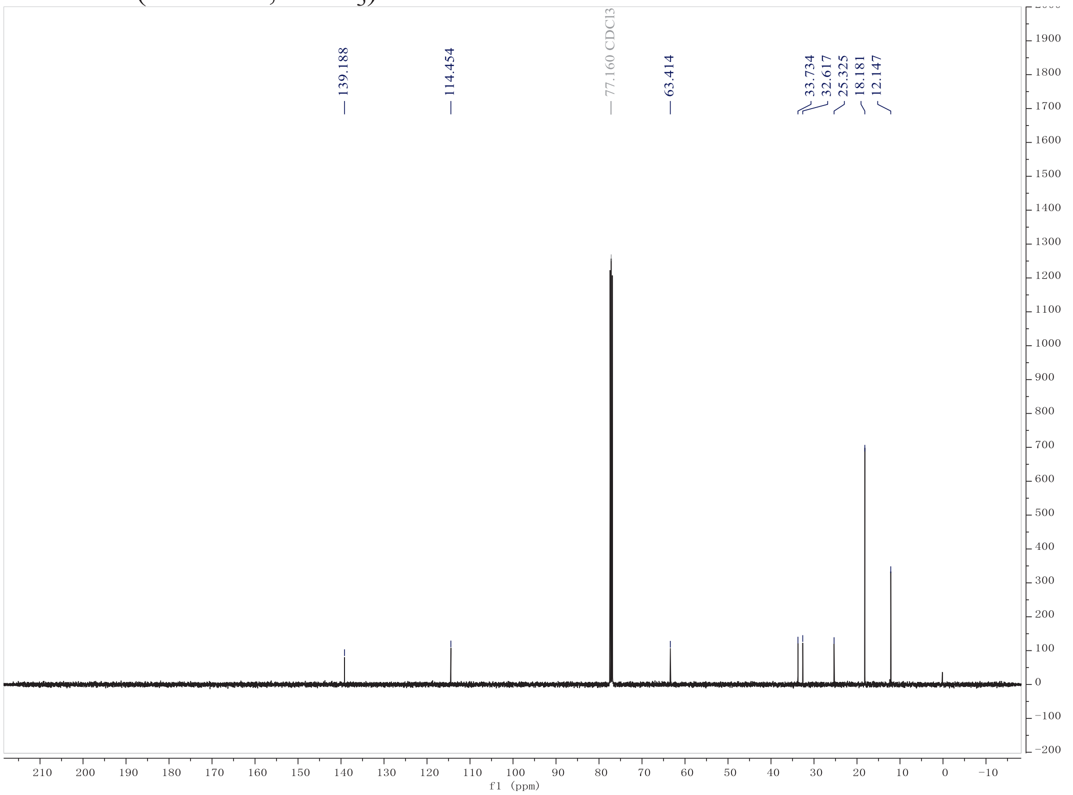

^{1}H NMR (500 MHz, $CDCl_3$)

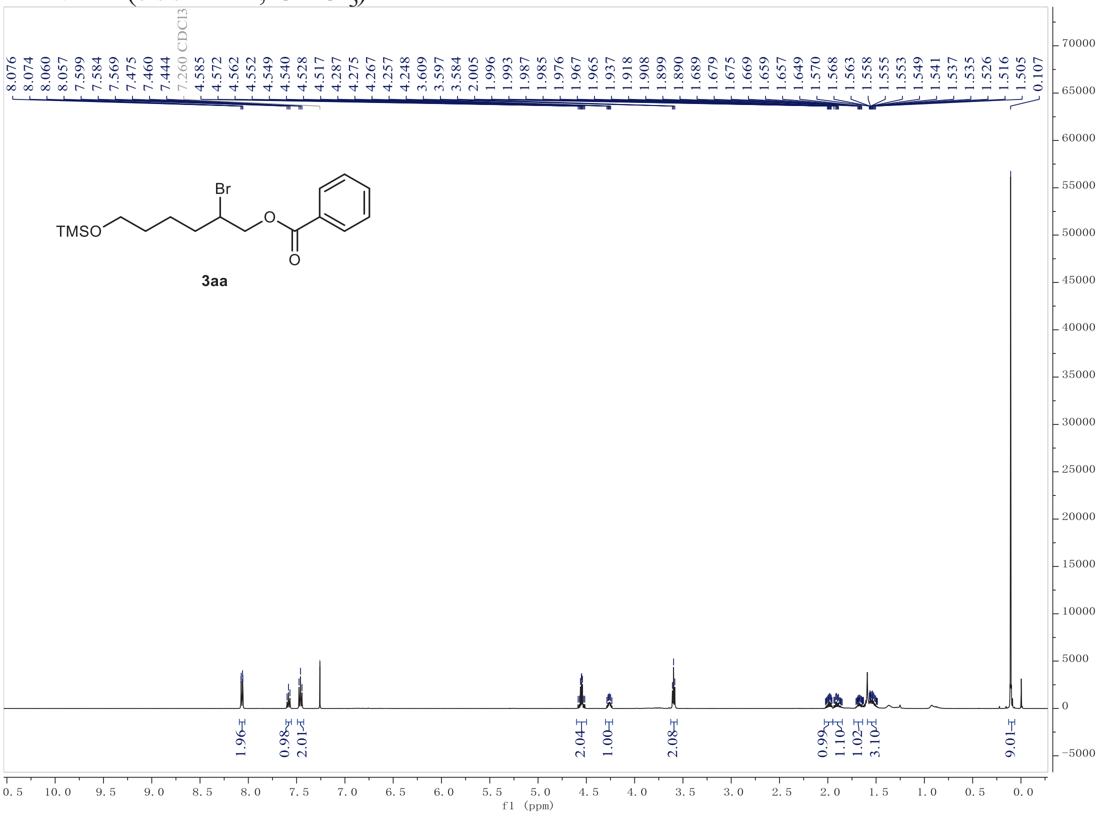

^{13}C NMR (126 MHz, $CDCl_3$)

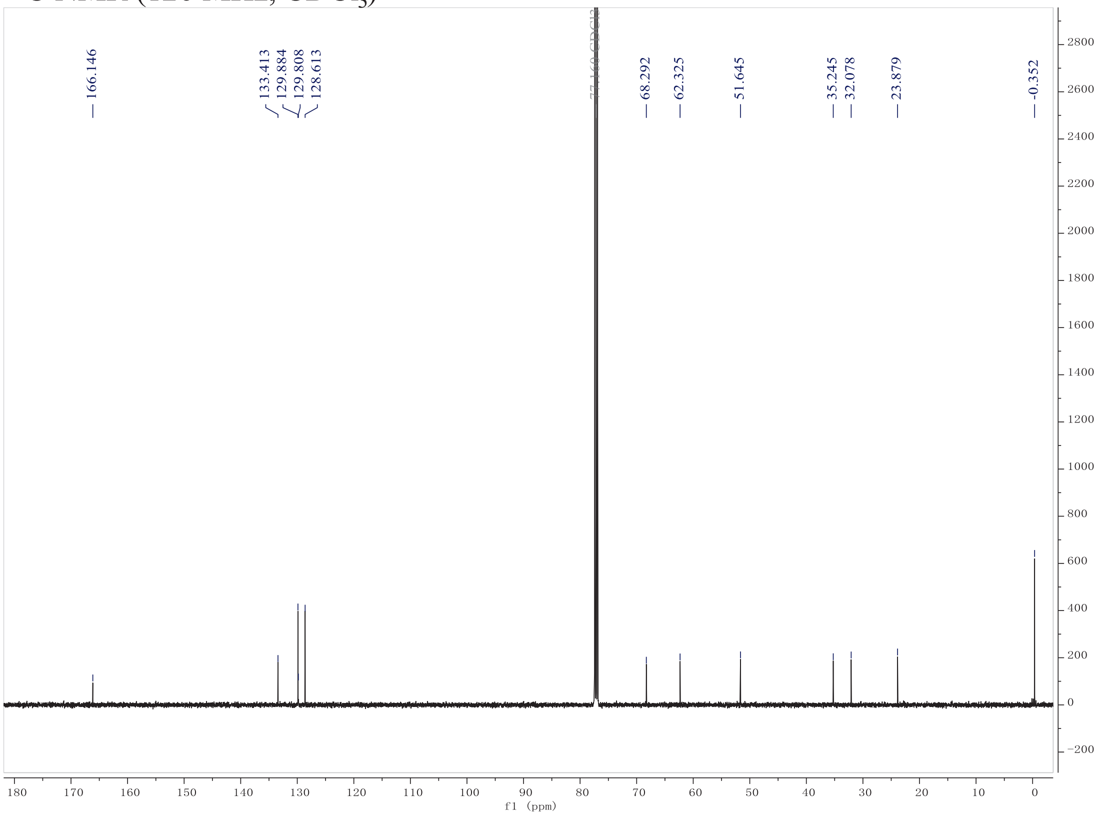

^{1}H NMR (500 MHz, $CDCl_3$)

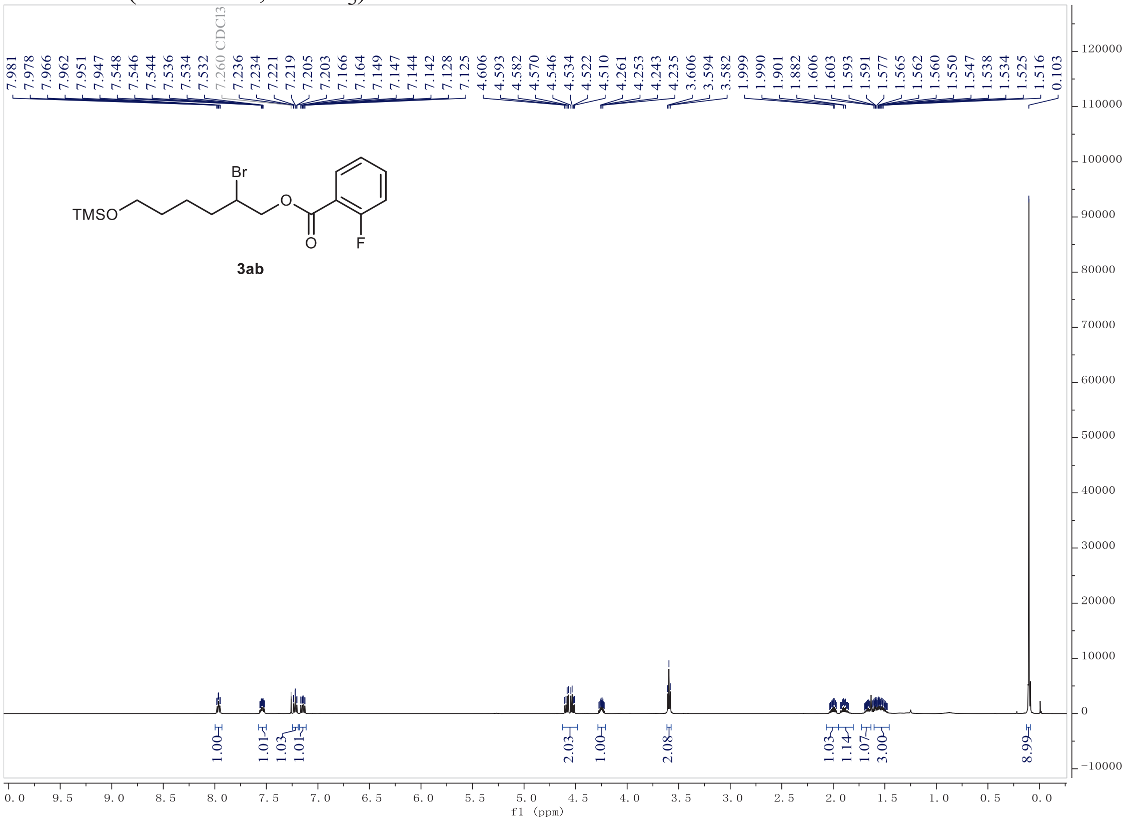

^{13}C NMR (126 MHz, $CDCl_3$)

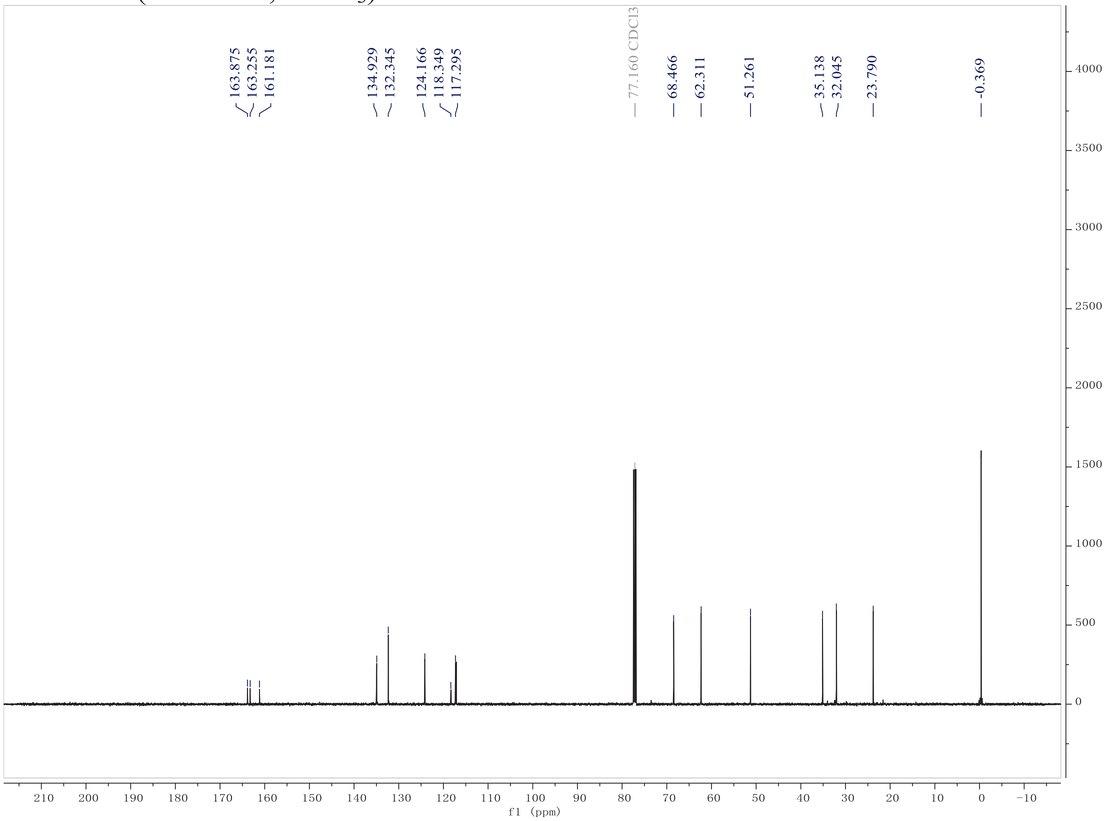

^{1}H NMR (500 MHz, $CDCl_3$)

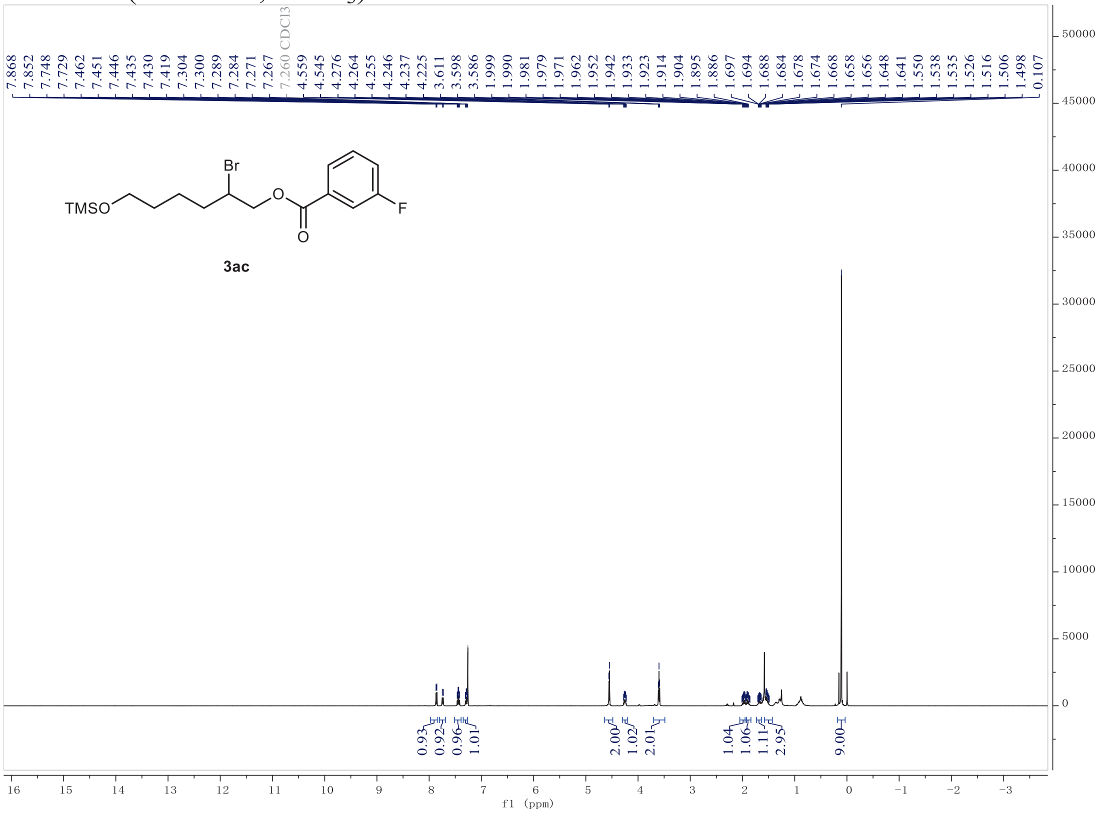

^{13}C NMR (126 MHz, $CDCl_3$)

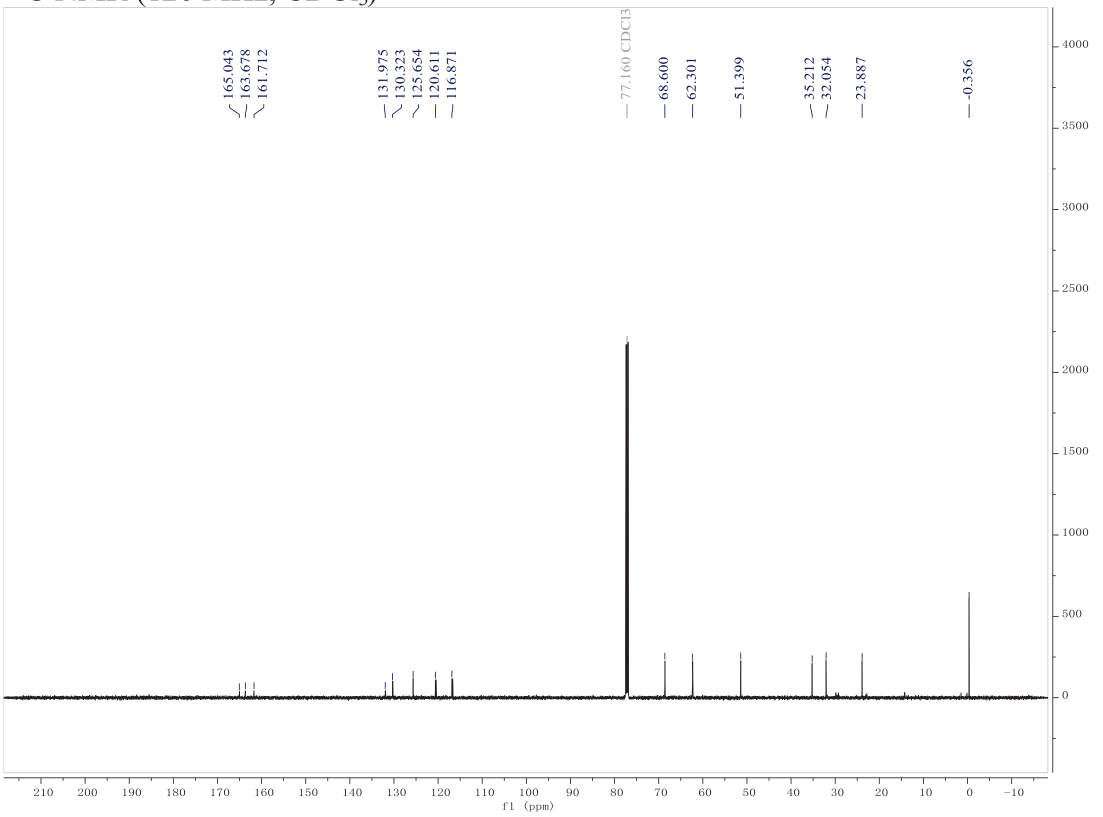

^{1}H NMR (500 MHz, $CDCl_3$)

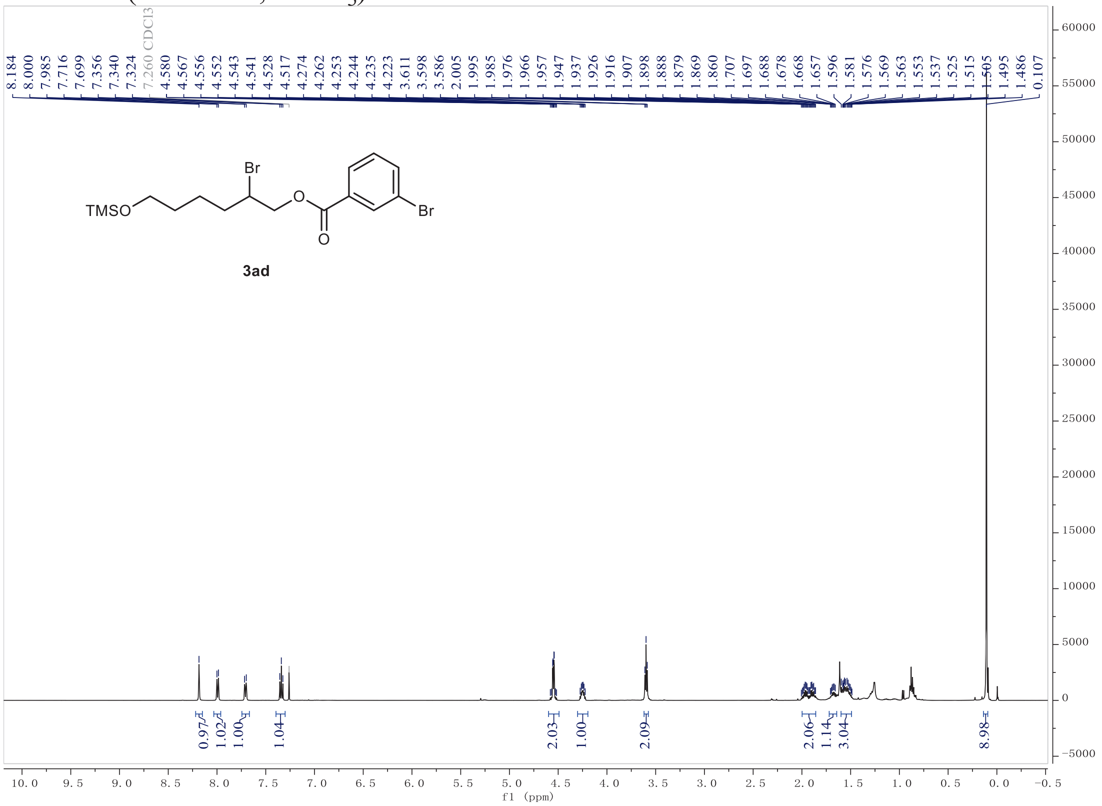

^{13}C NMR (126 MHz, $CDCl_3$)

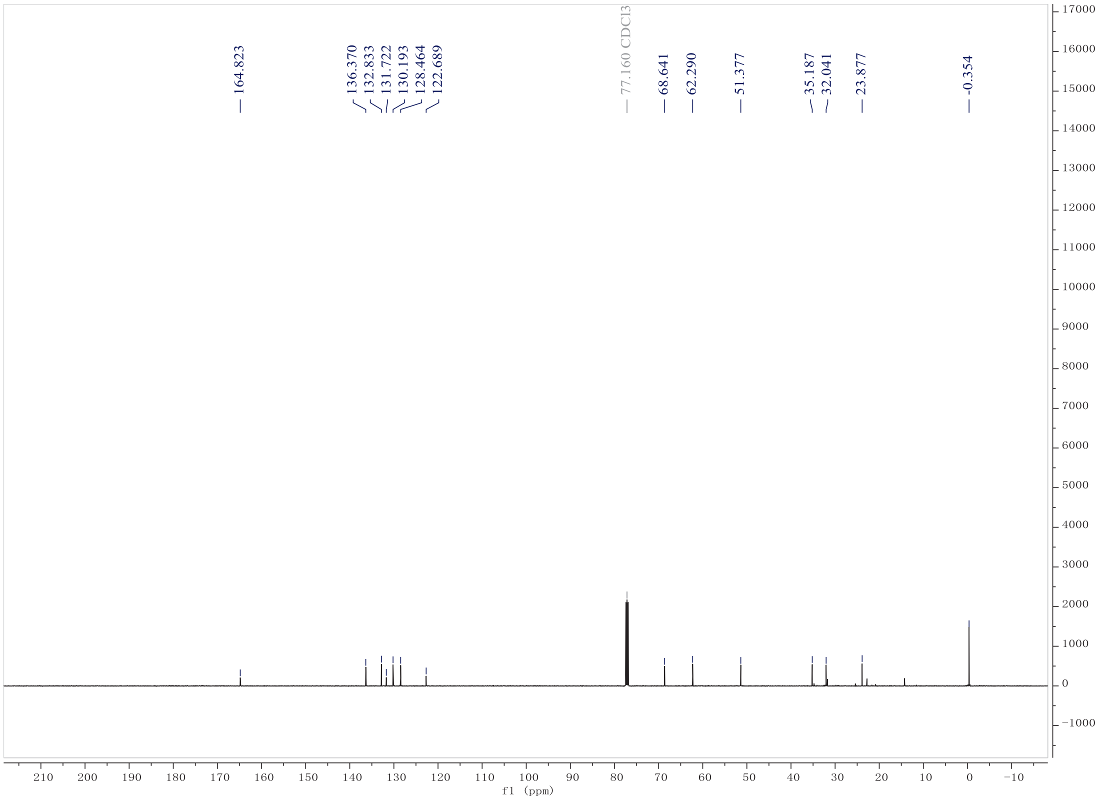

^{1}H NMR (500 MHz, $CDCl_3$)

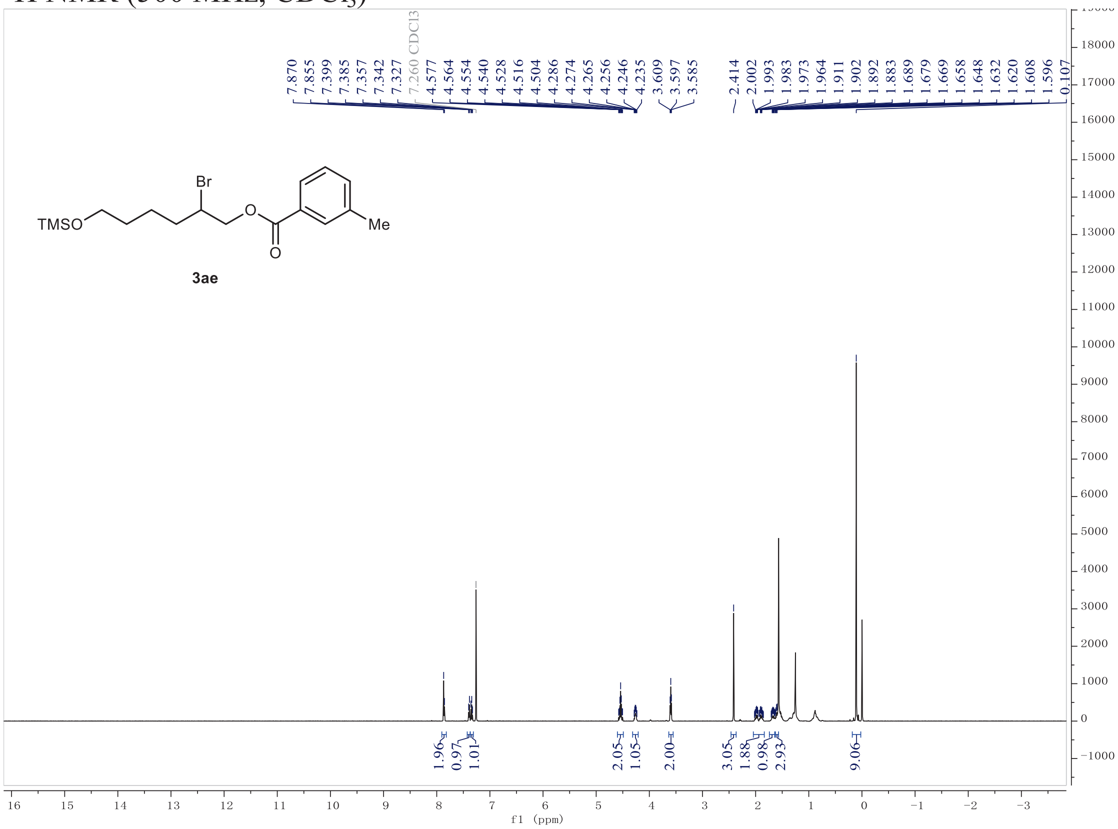

^{13}C NMR (126 MHz, $CDCl_3$)

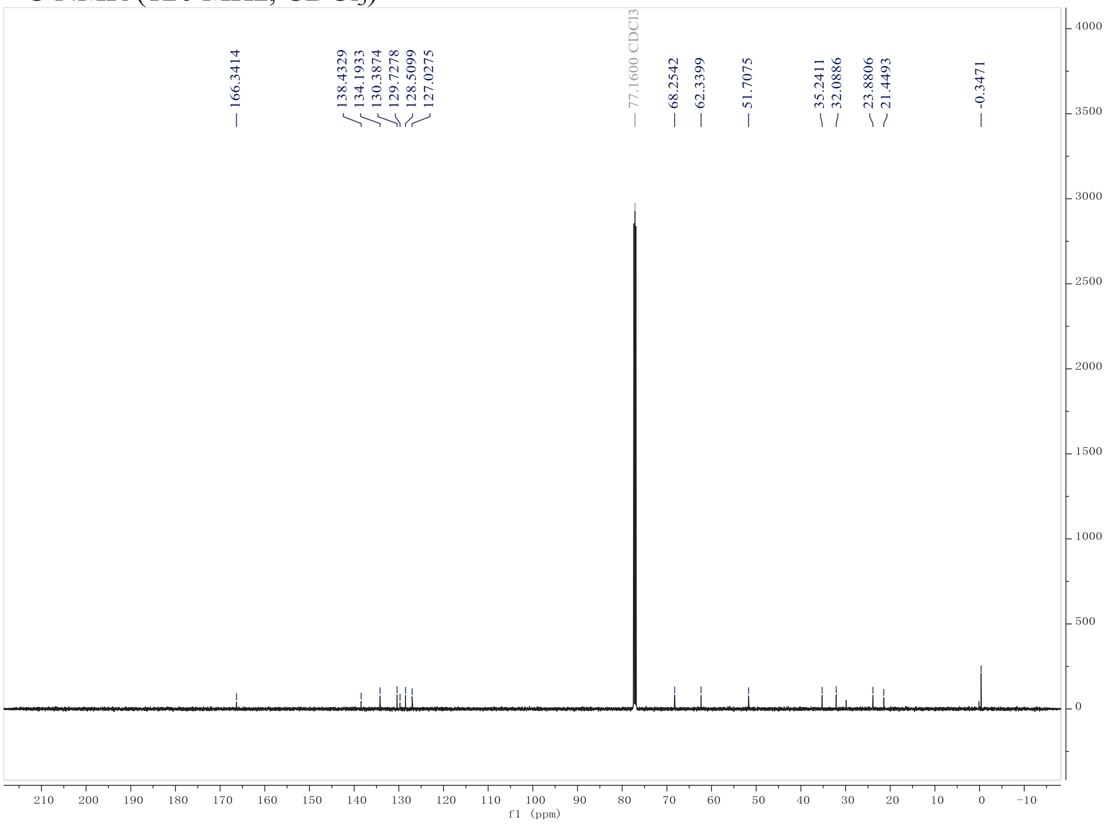

^{1}H NMR (400 MHz, $CDCl_3$)

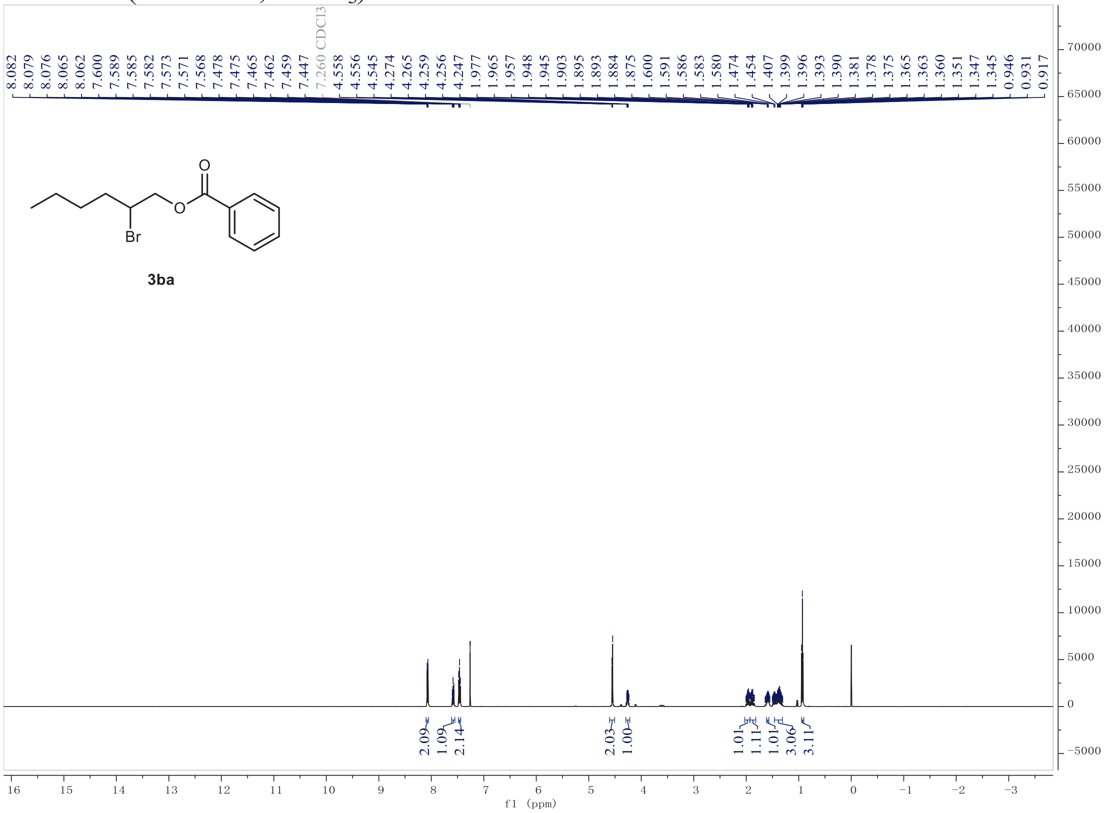

^{13}C NMR (126 MHz, $CDCl_3$)

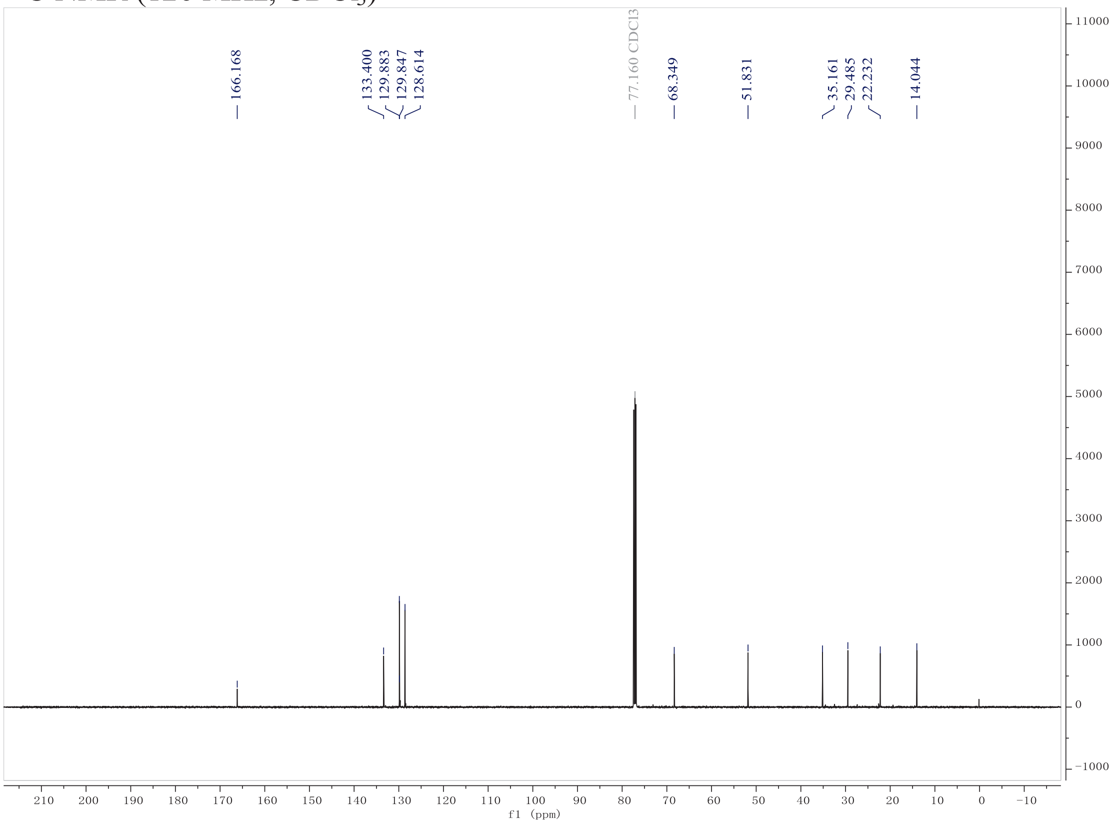

^{1}H NMR (500 MHz, $CDCl_3$)

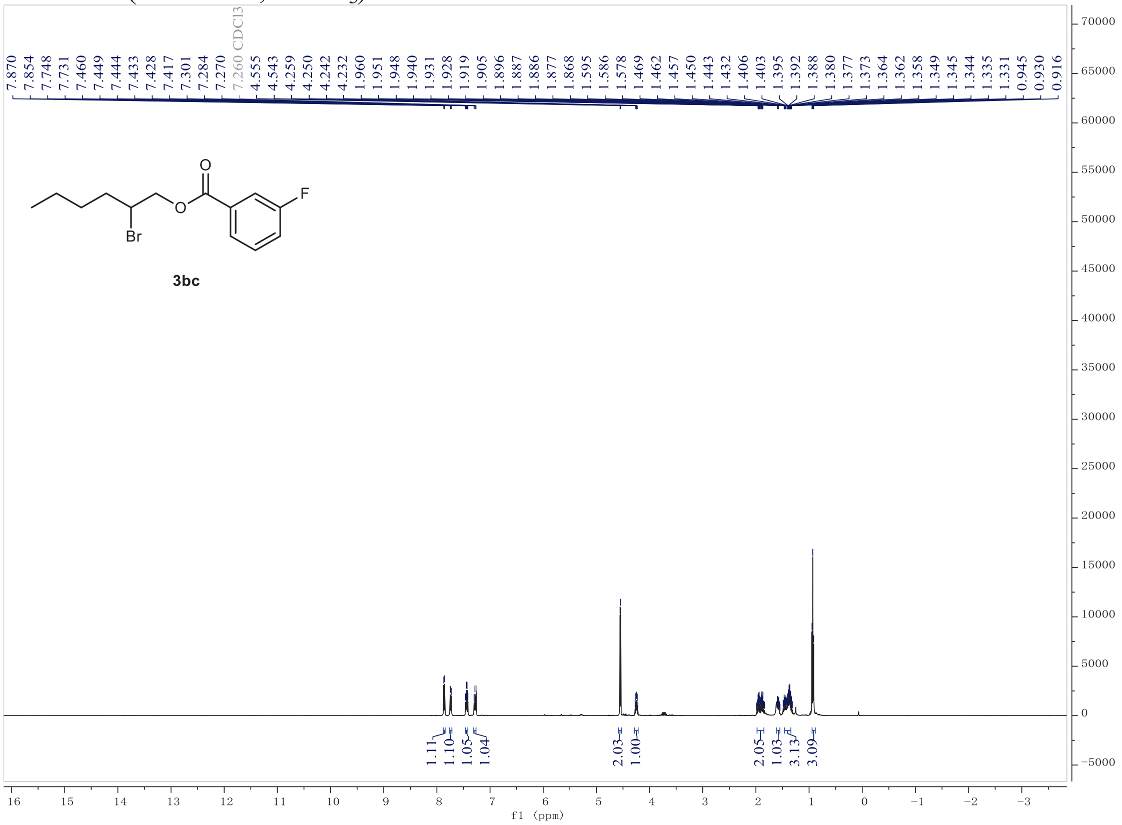

^{13}C NMR (126 MHz, $CDCl_3$)

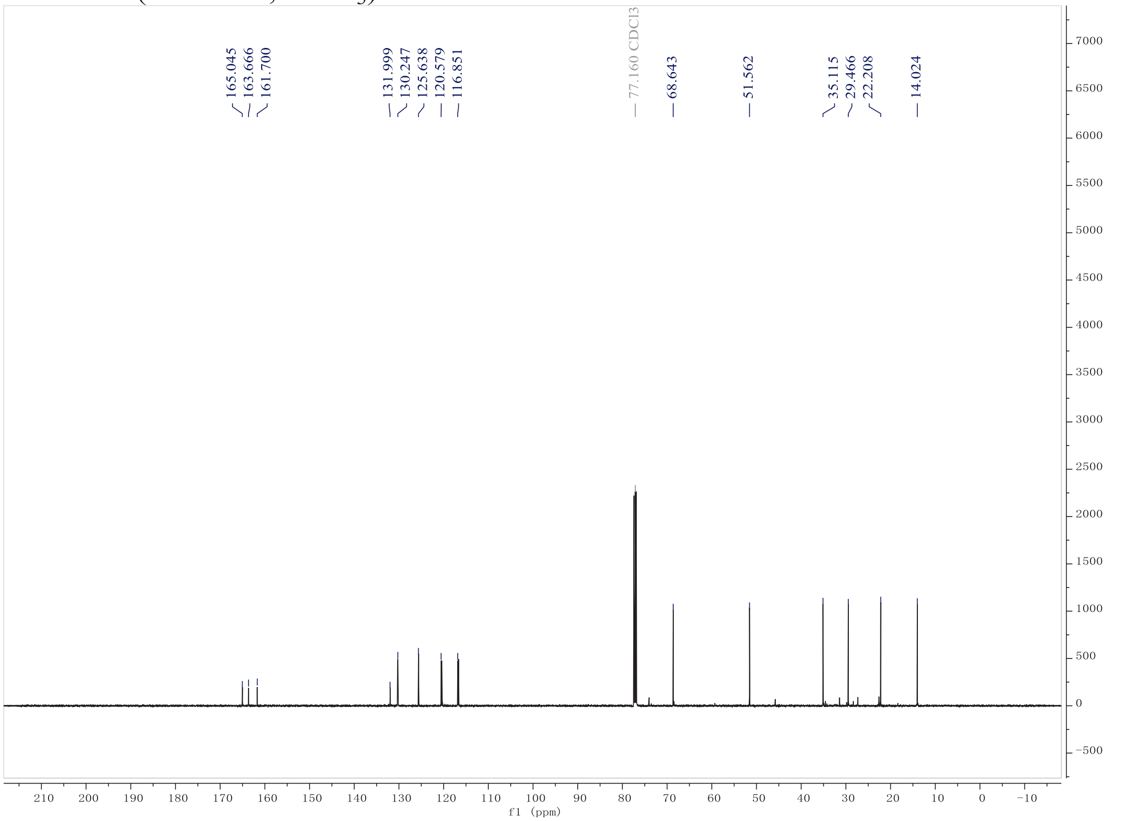

^{1}H NMR (500 MHz, $CDCl_3$)

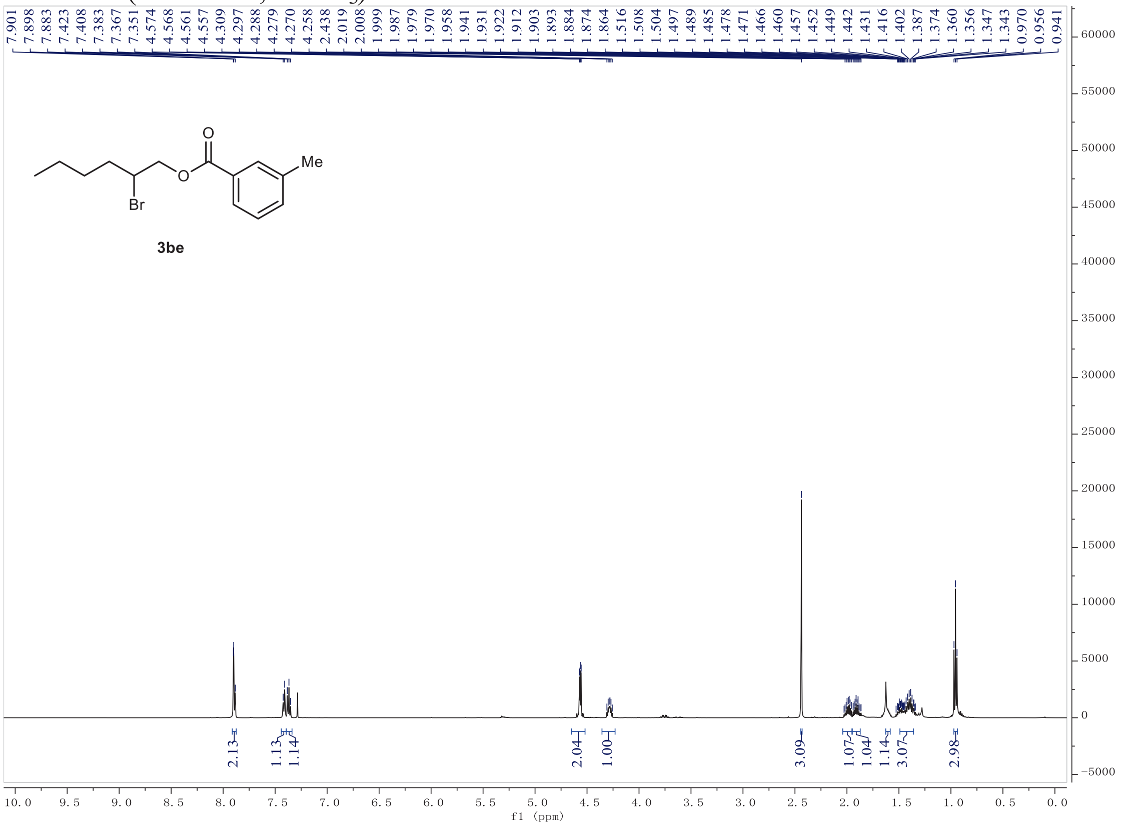

^{13}C NMR (126 MHz, $CDCl_3$)

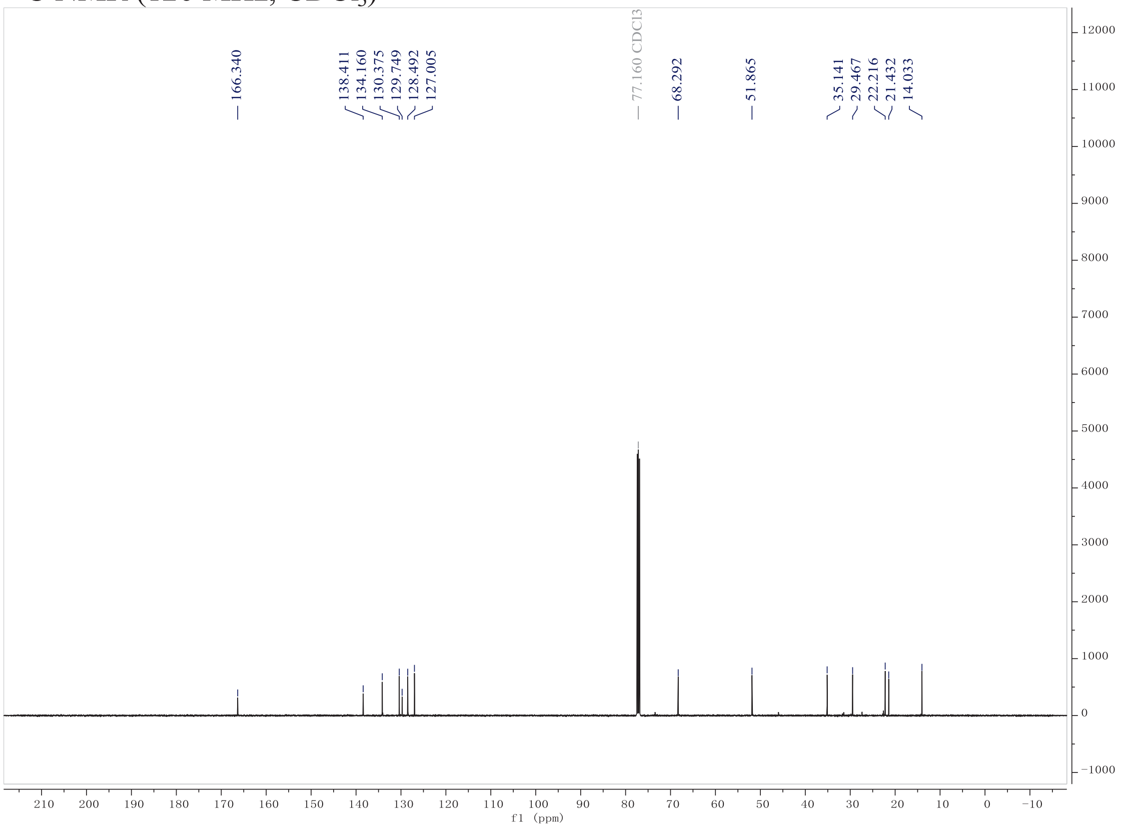

^{1}H NMR (500 MHz, $CDCl_3$)

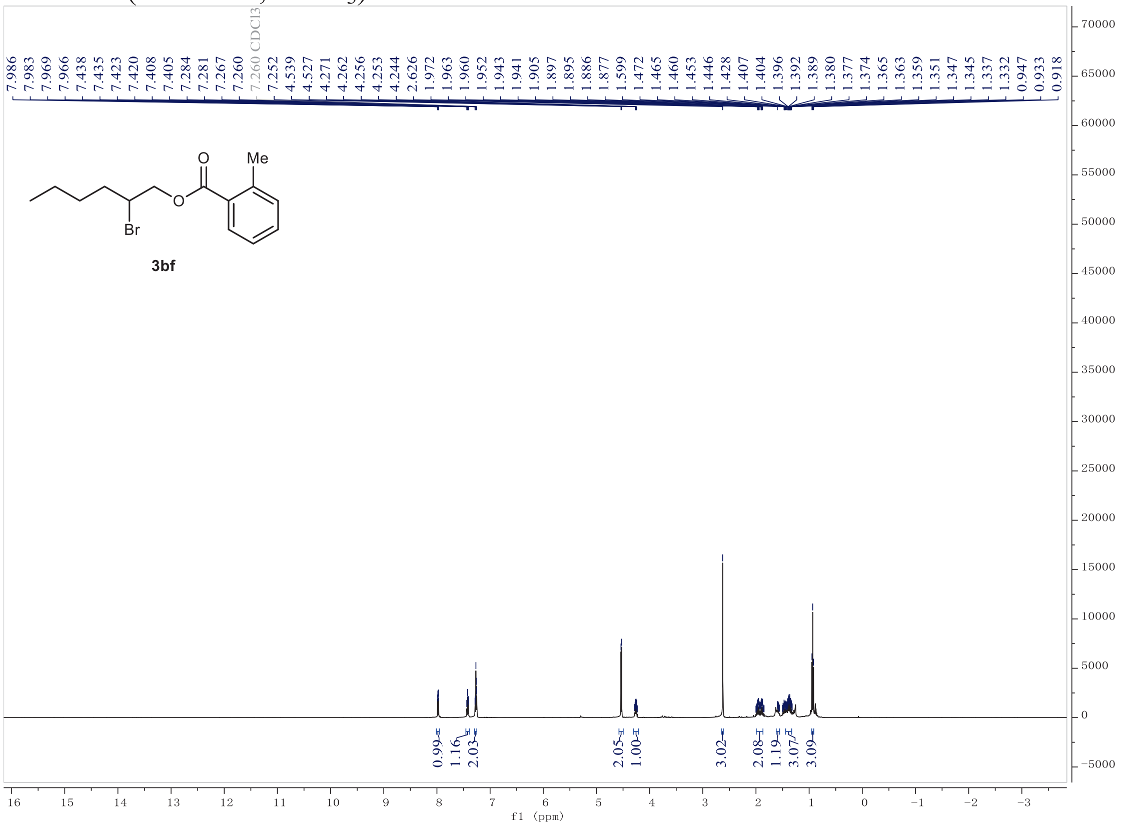

^{13}C NMR (126 MHz, $CDCl_3$)

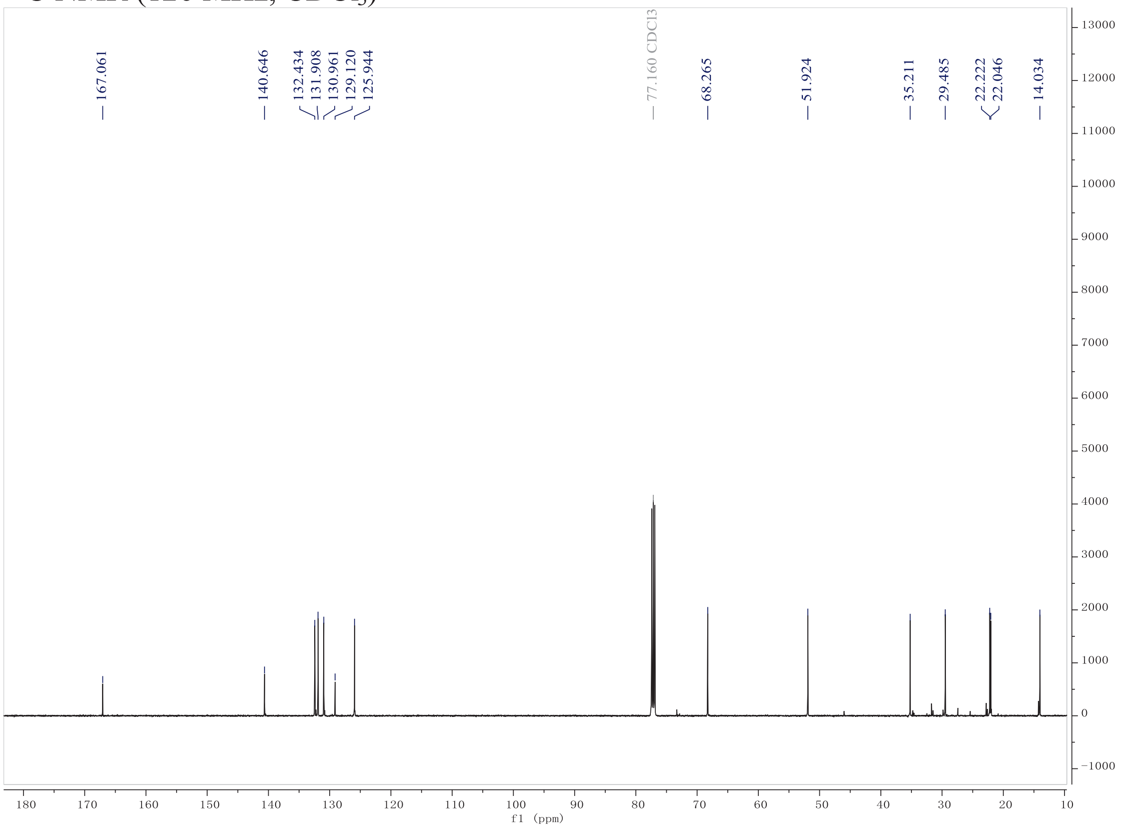

^{1}H NMR (500 MHz, $CDCl_3$)

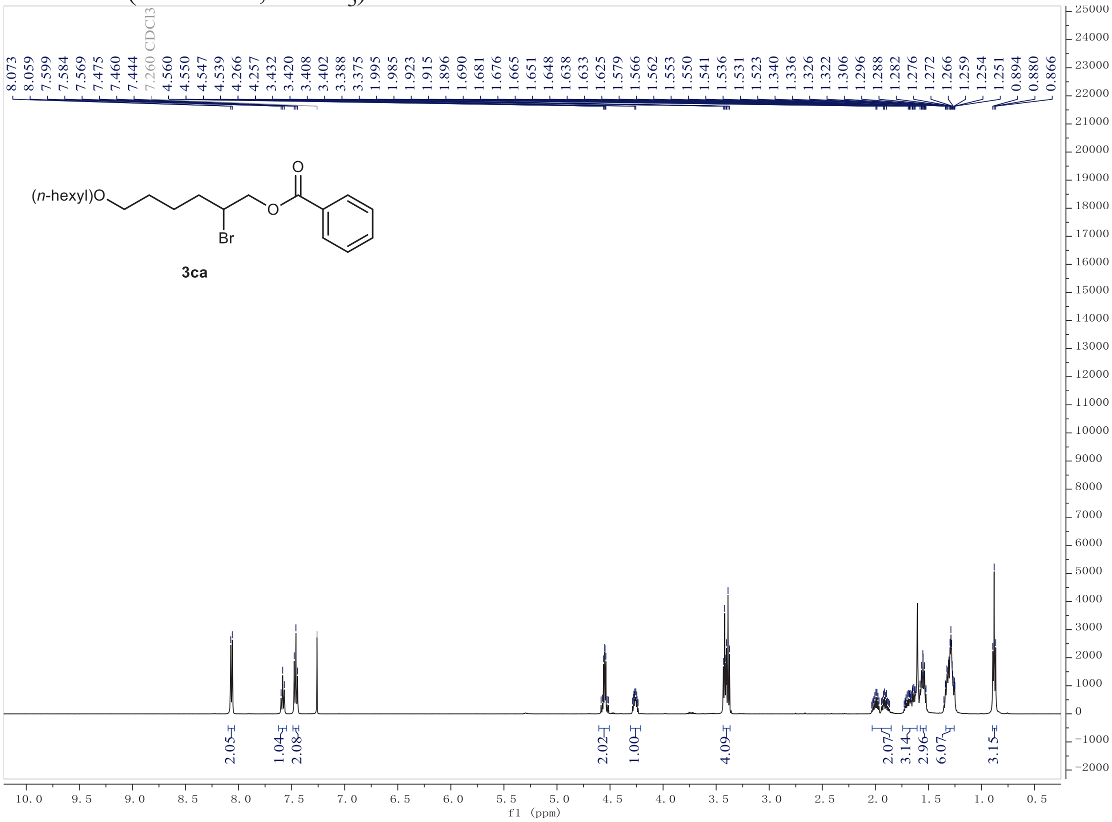

^{13}C NMR (126 MHz, $CDCl_3$)

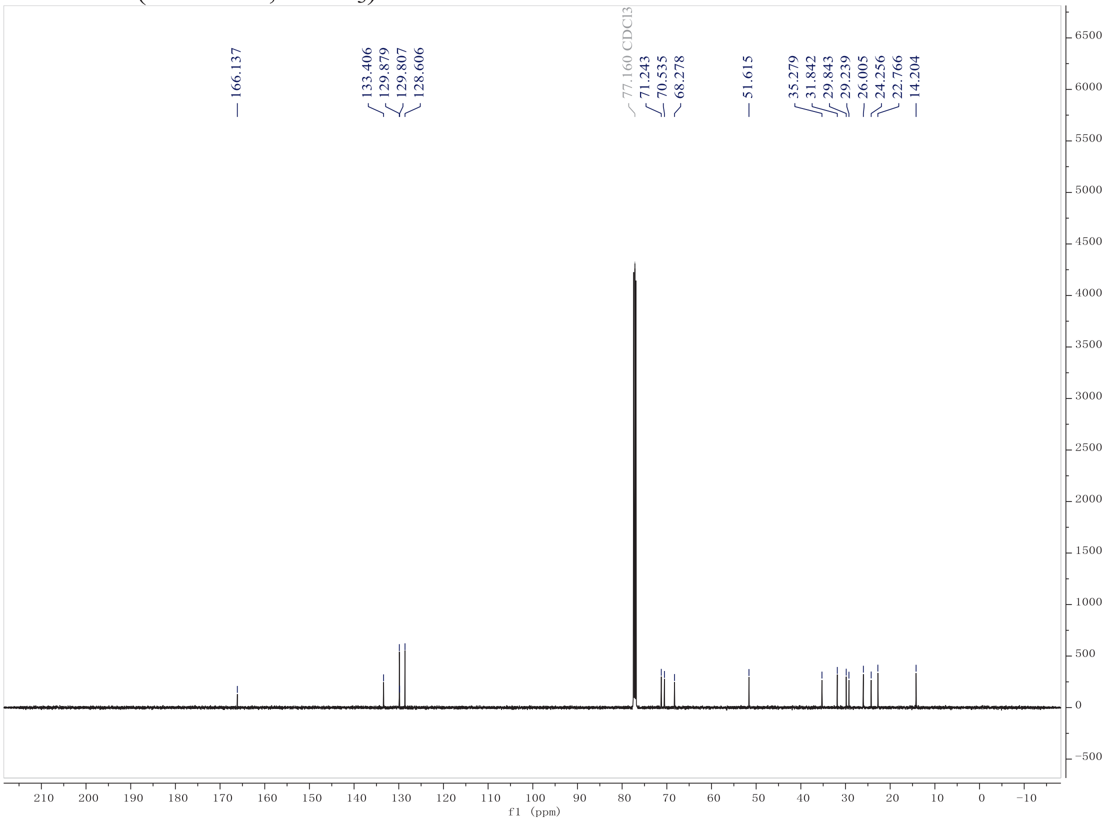

^{1}H NMR (500 MHz, $CDCl_3$)

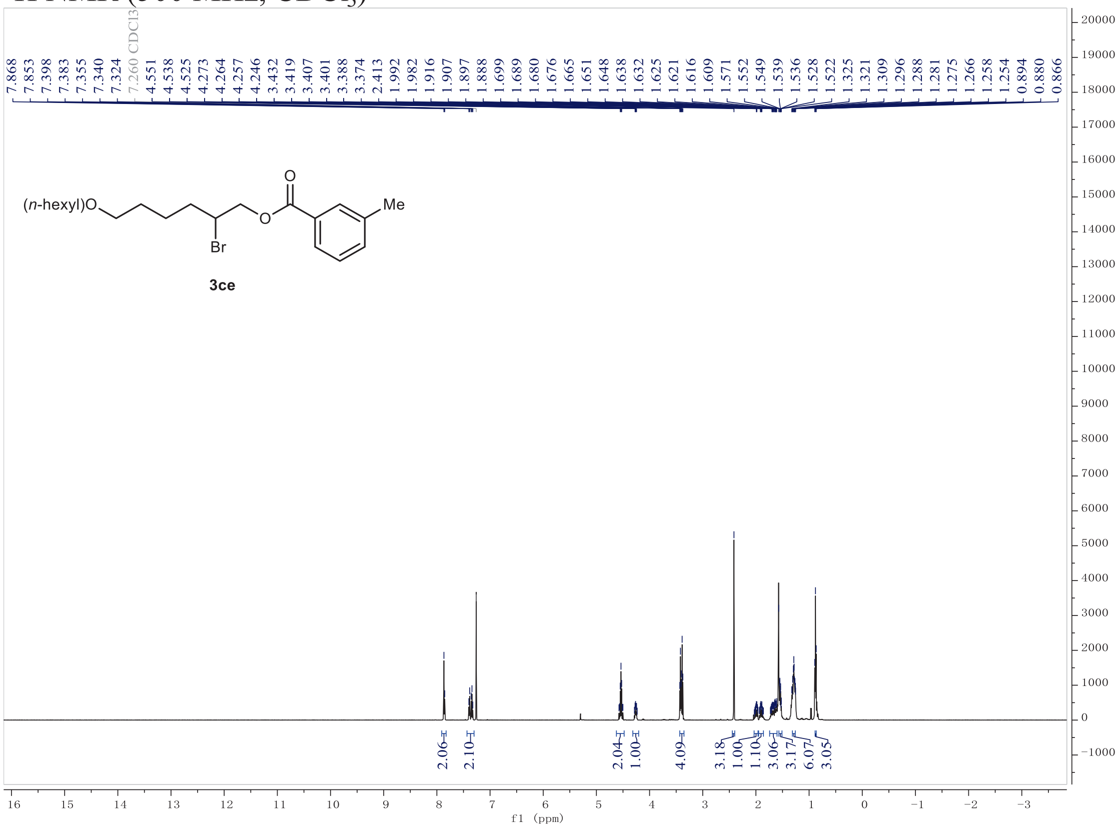

^{13}C NMR (126 MHz, $CDCl_3$)

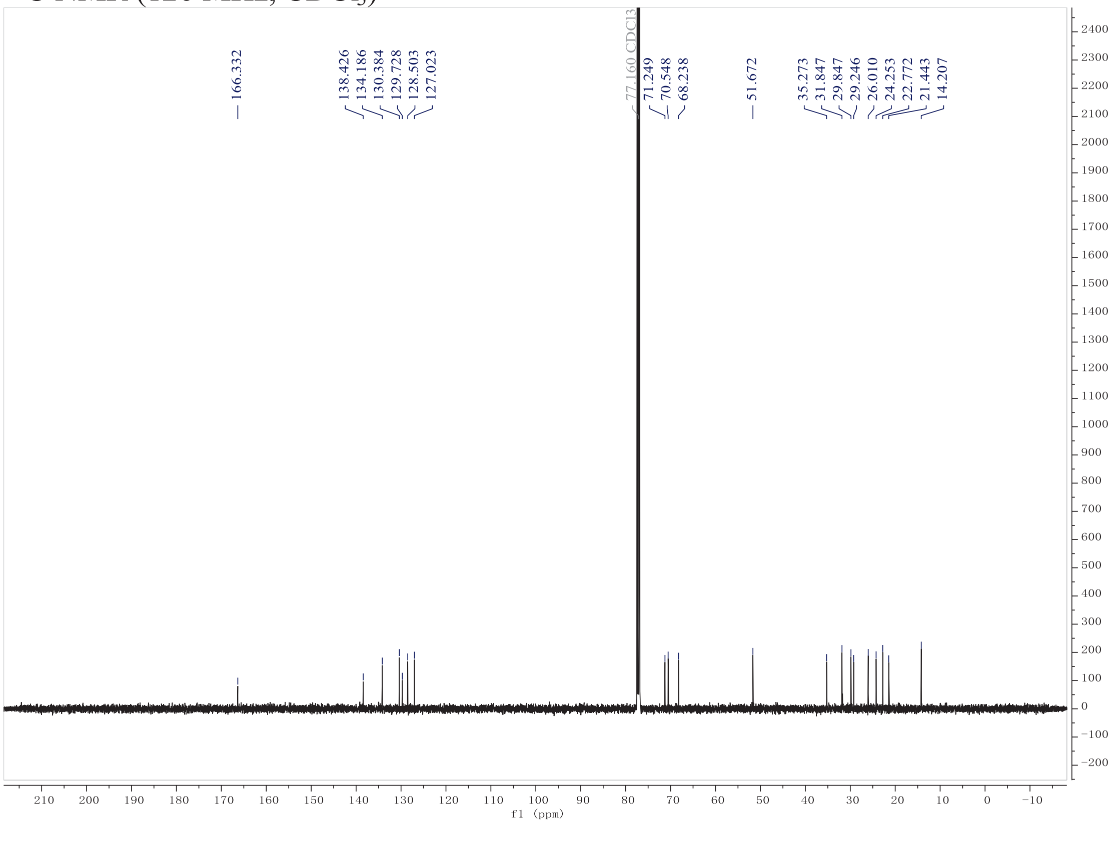

^{1}H NMR (500 MHz, $CDCl_3$)

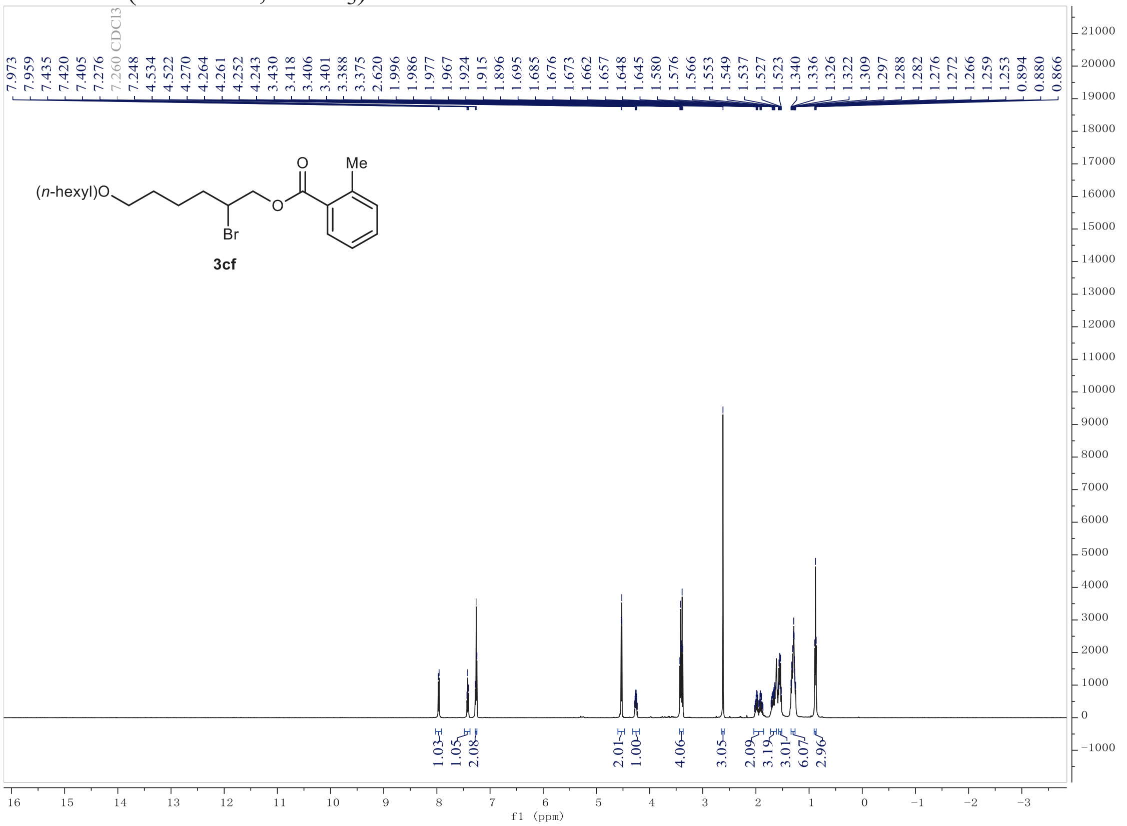

^{13}C NMR (126 MHz, $CDCl_3$)

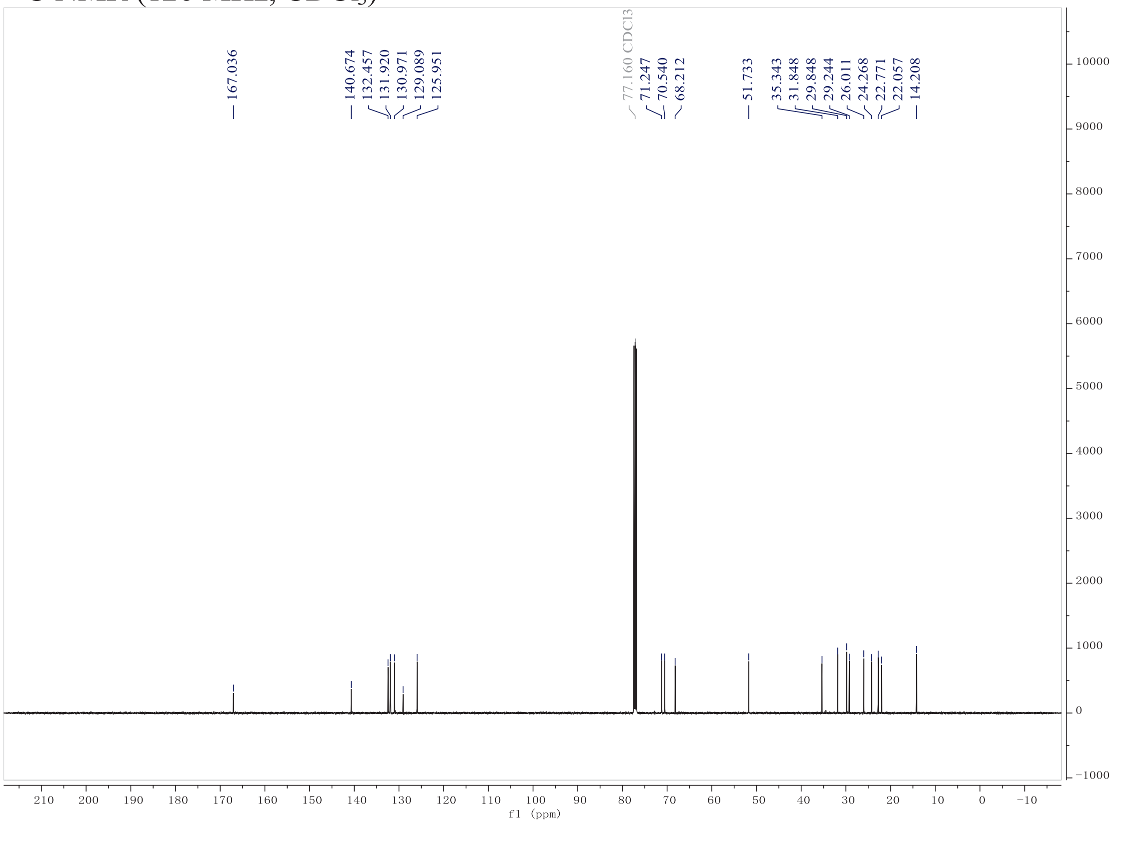

^{1}H NMR (500 MHz, $CDCl_3$)

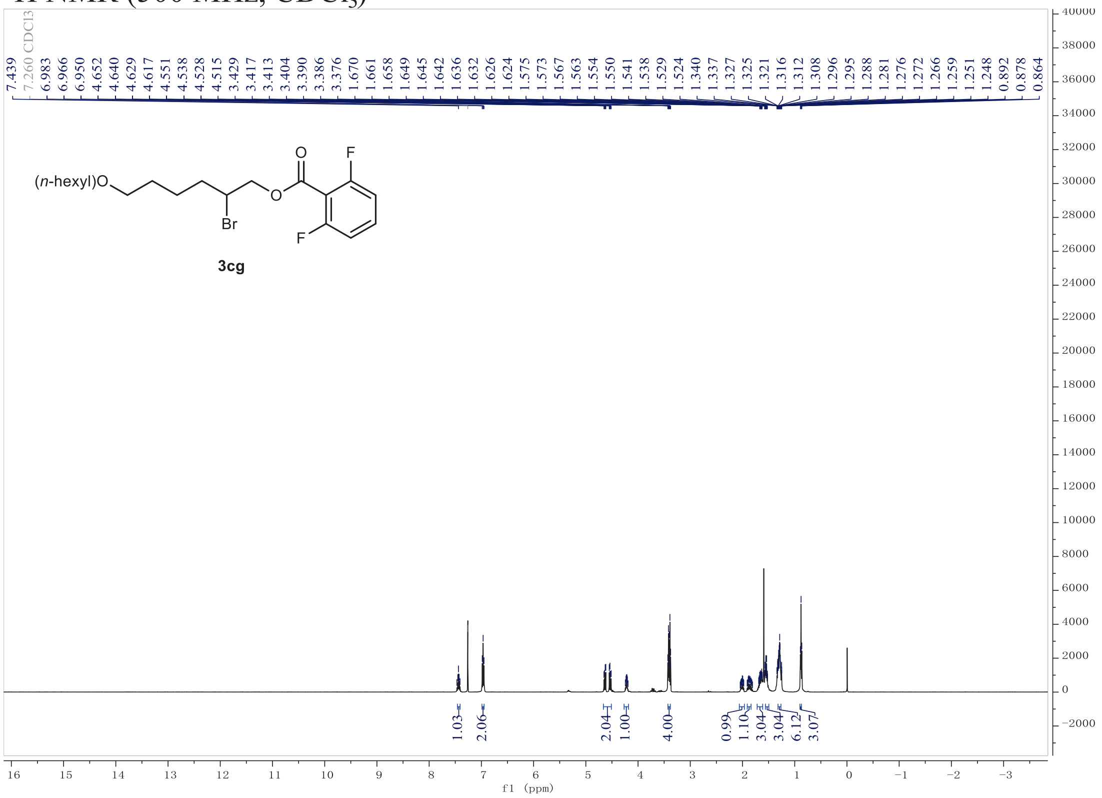

^{13}C NMR (126 MHz, $CDCl_3$)

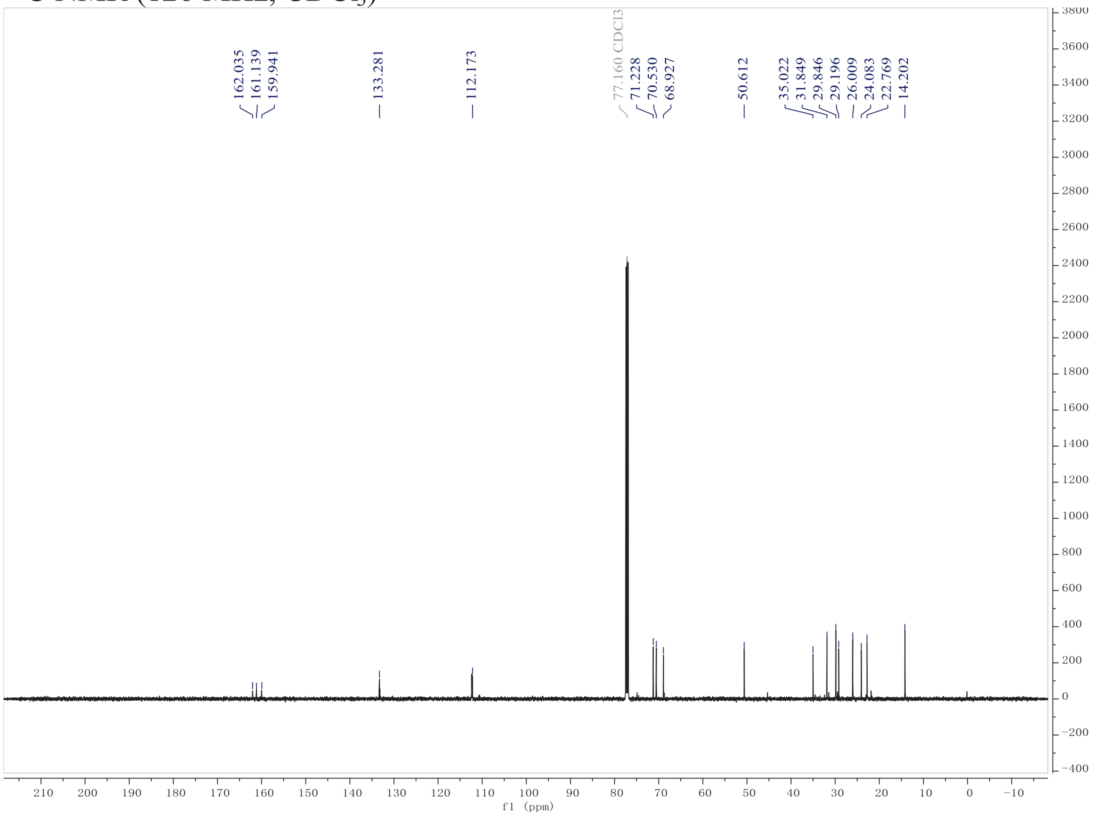

^{1}H NMR (500 MHz, $CDCl_3$)

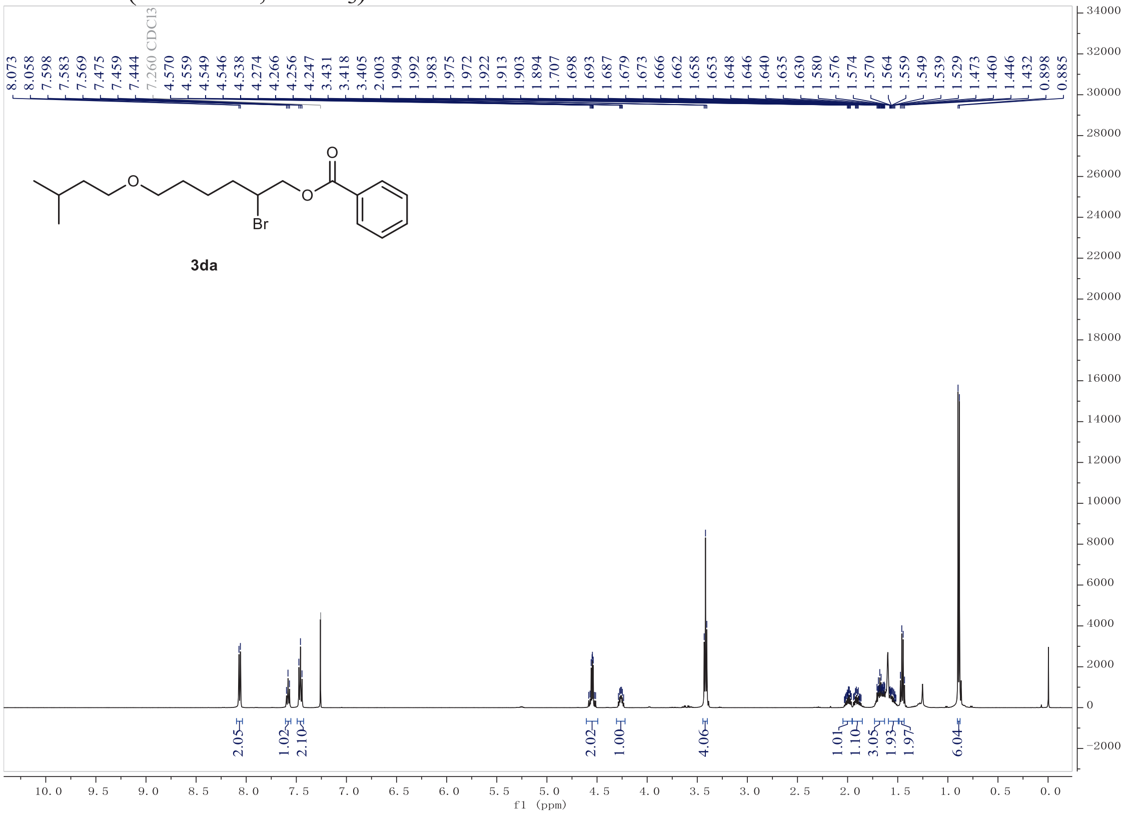

^{13}C NMR (126 MHz, $CDCl_3$)

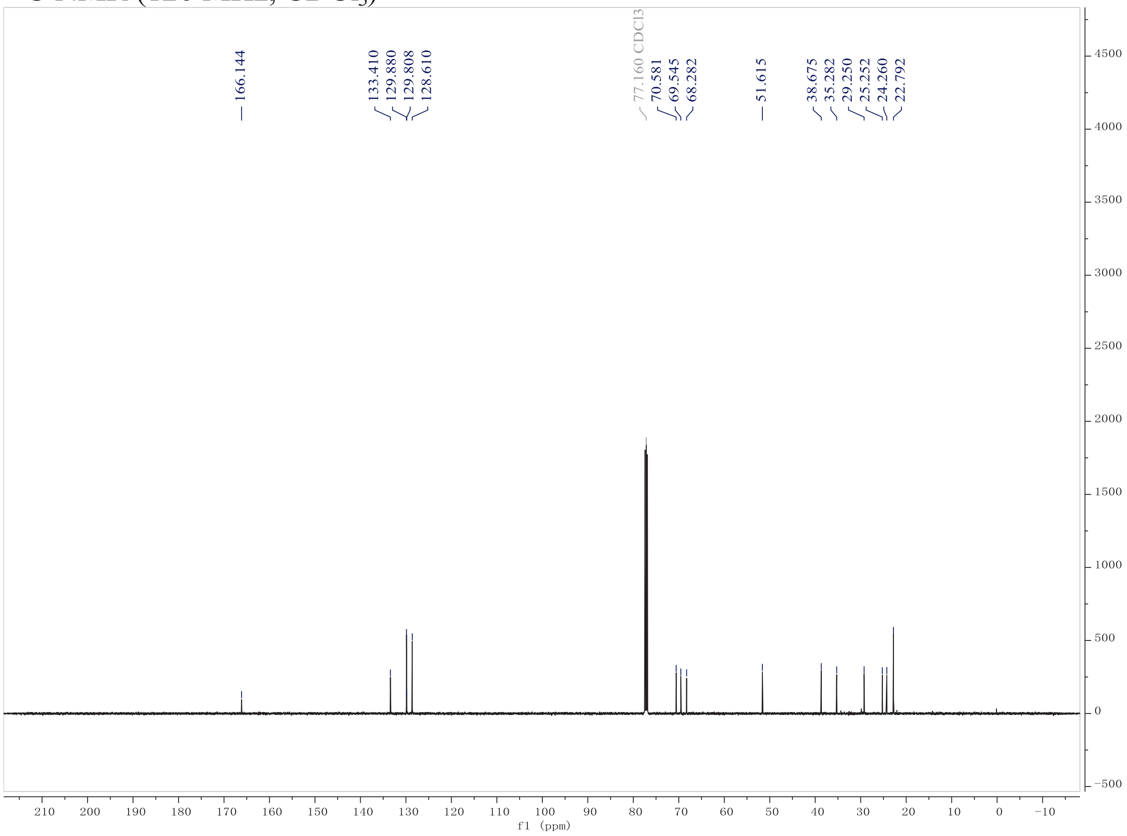

^{1}H NMR (500 MHz, $CDCl_3$)

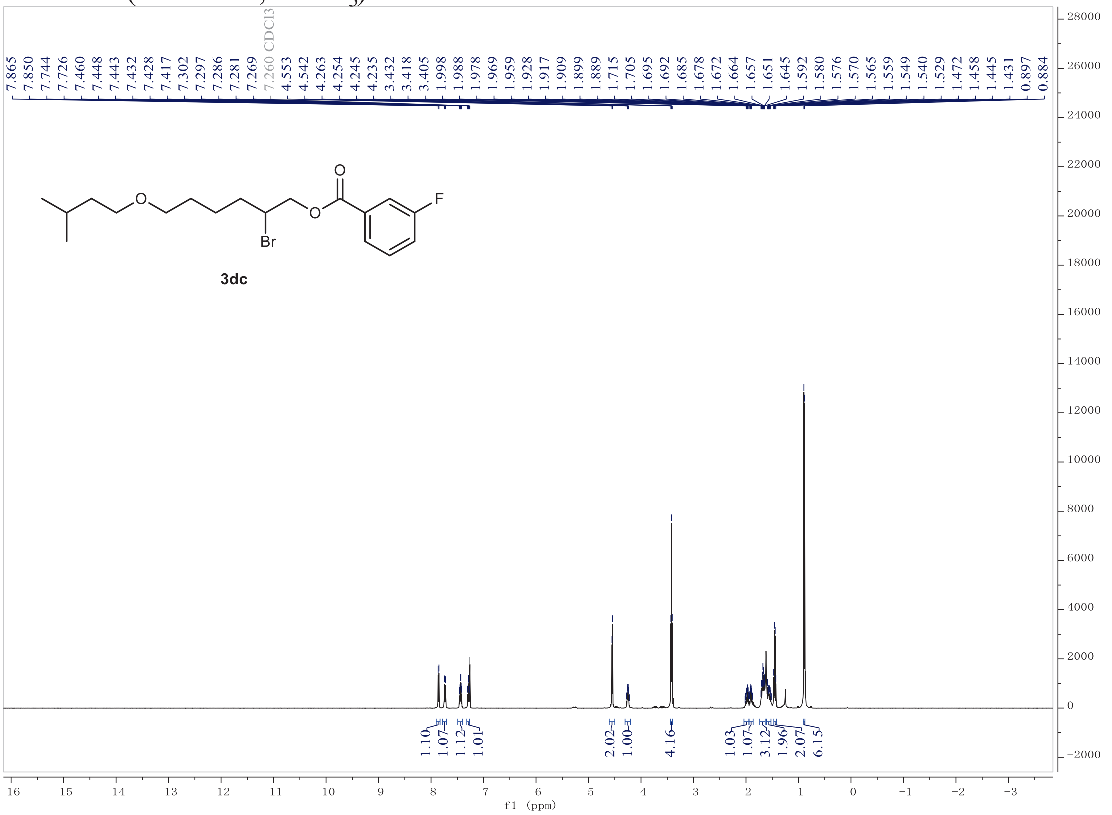

^{13}C NMR (126 MHz, $CDCl_3$)

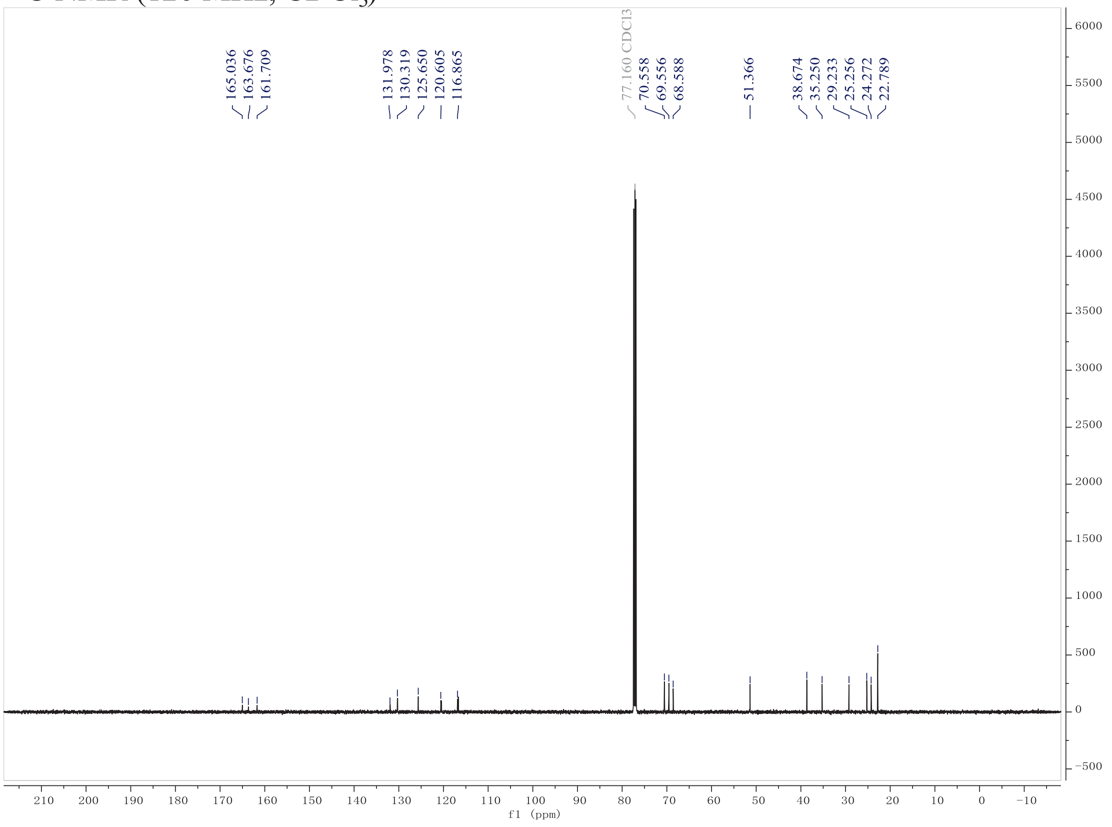

^{1}H NMR (500 MHz, $CDCl_3$)

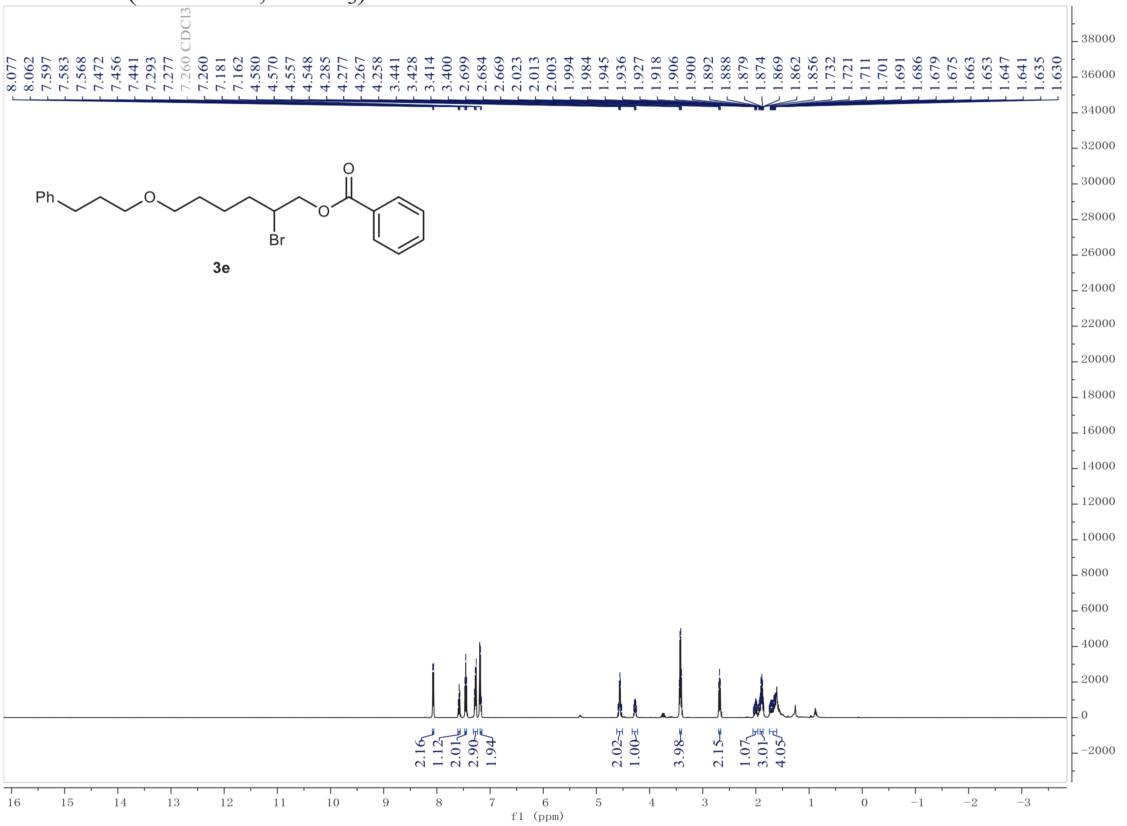

^{13}C NMR (126 MHz, $CDCl_3$)

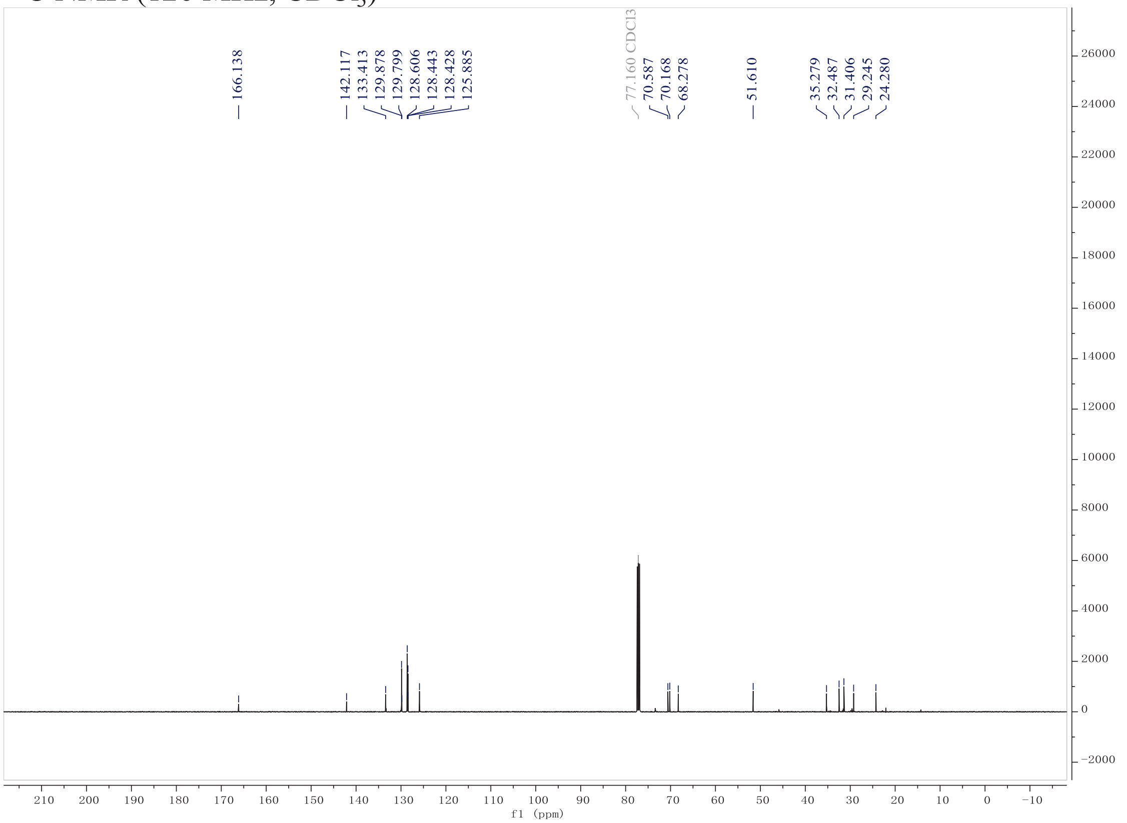

^{1}H NMR (500 MHz, $CDCl_3$)

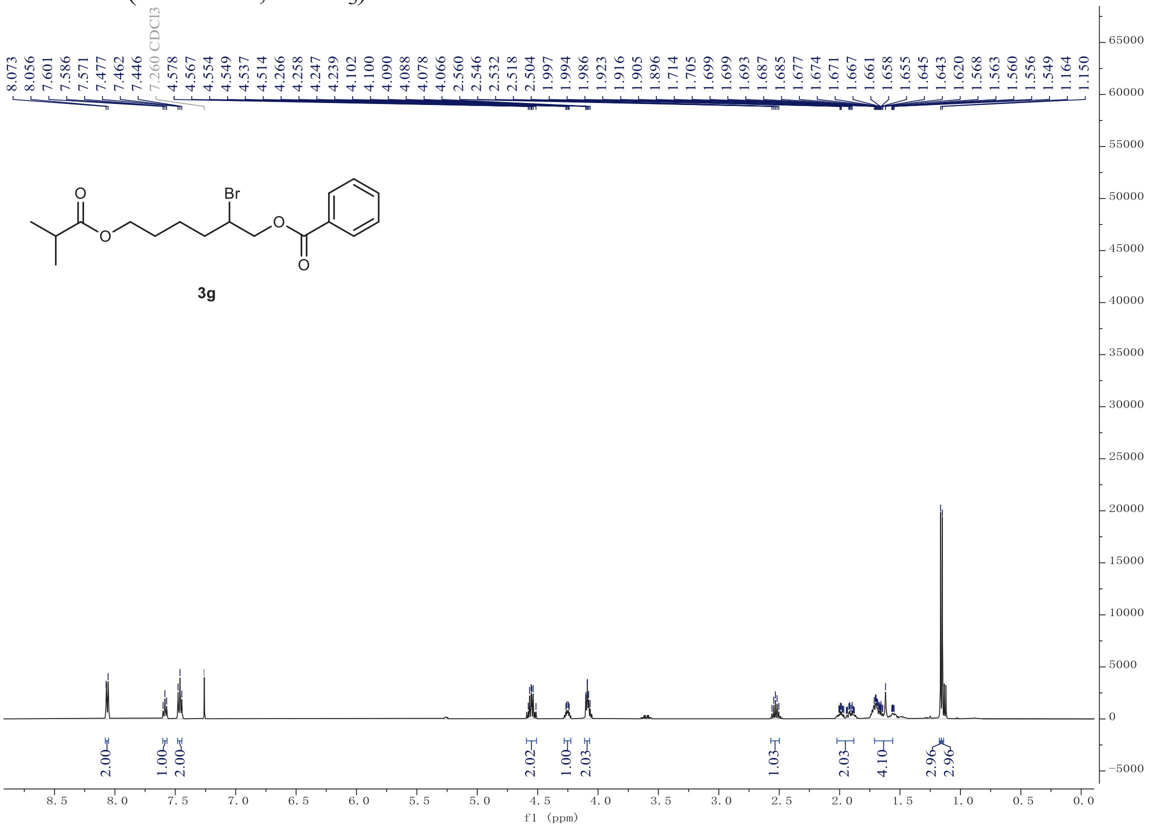

3g

^{13}C NMR (126 MHz, $CDCl_3$)

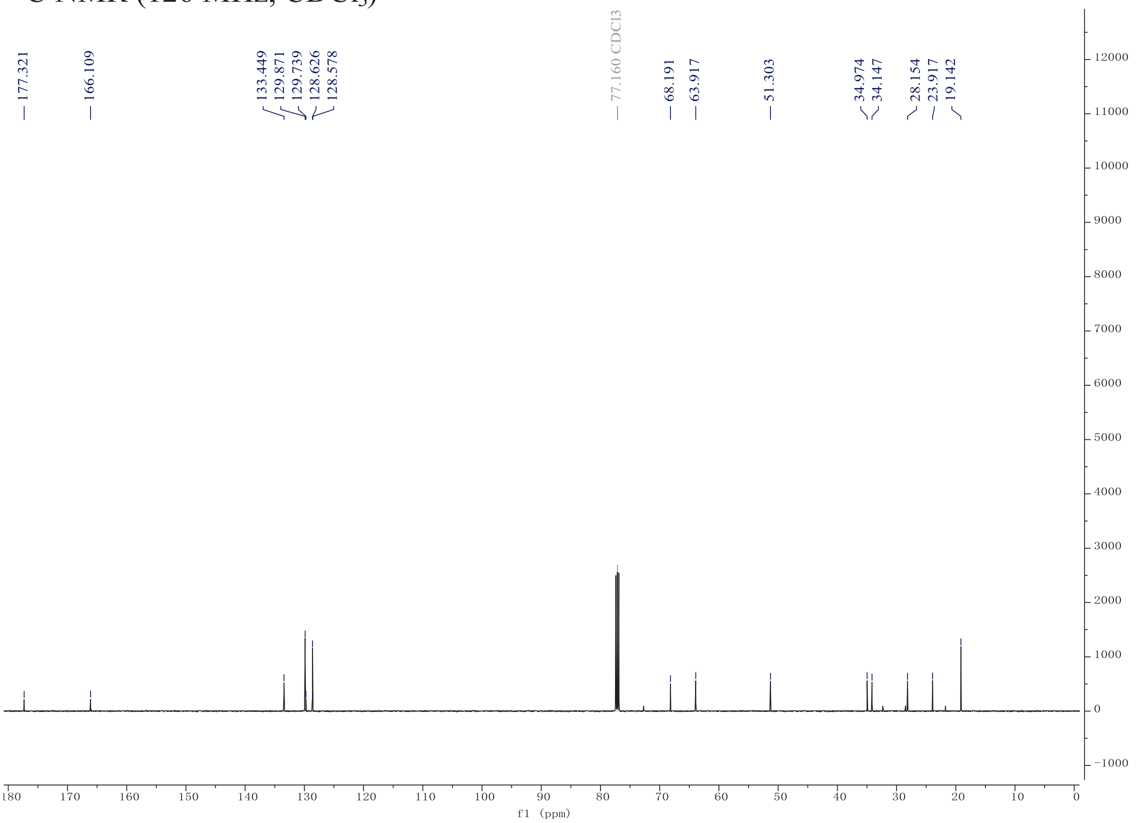

^{1}H NMR (500 MHz, $CDCl_3$)

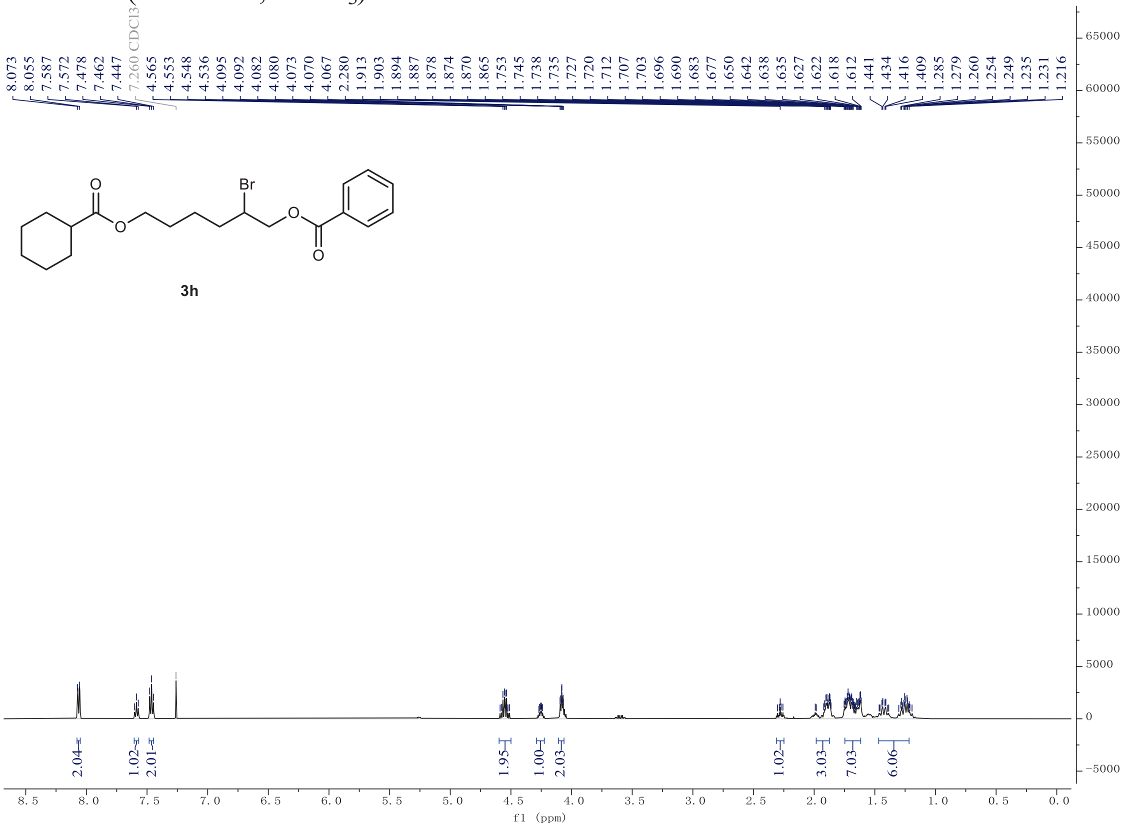

^{13}C NMR (126 MHz, $CDCl_3$)

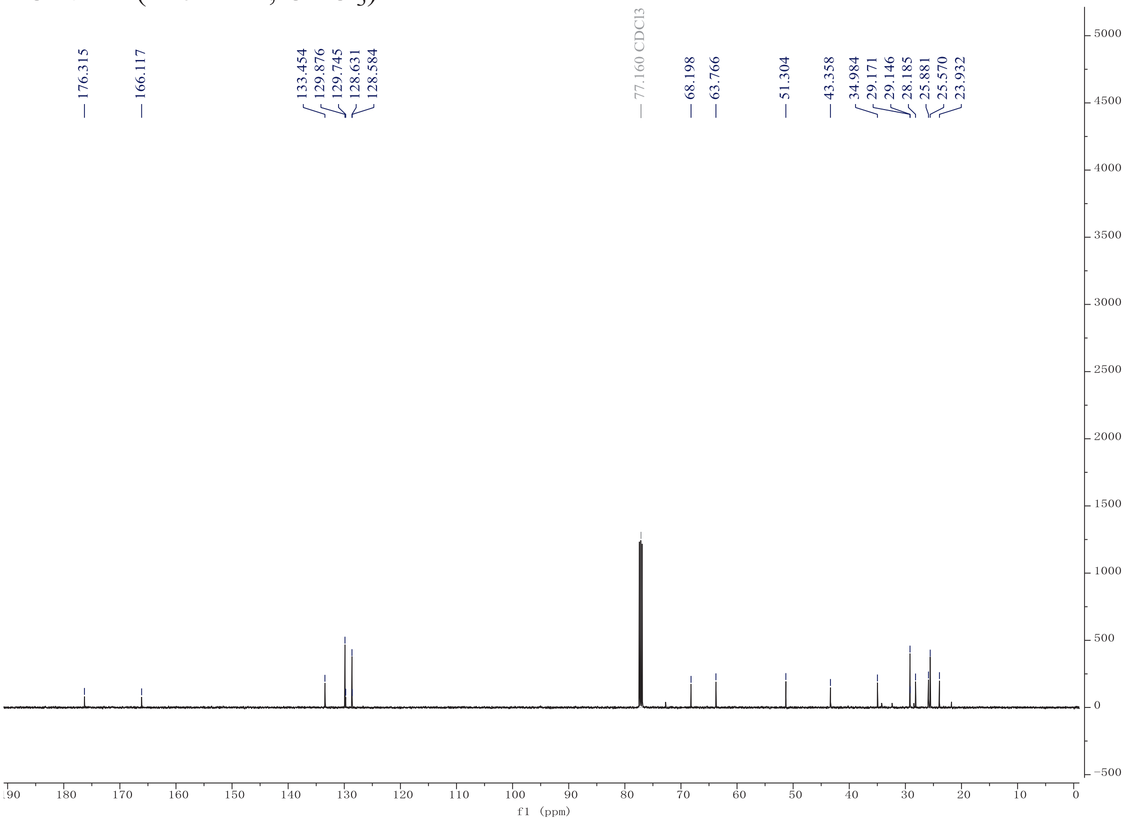

^1H NMR (500 MHz, $CDCl_3$)

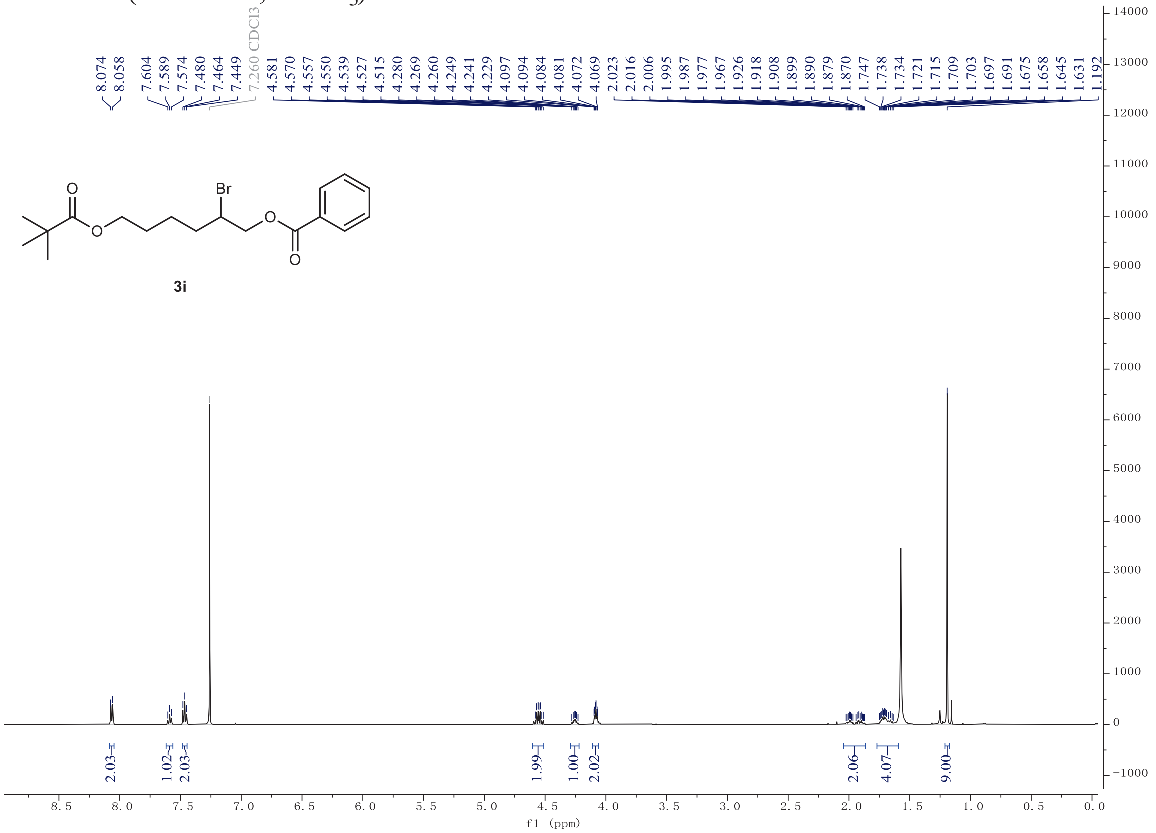

^{13}C NMR (126 MHz, $CDCl_3$)

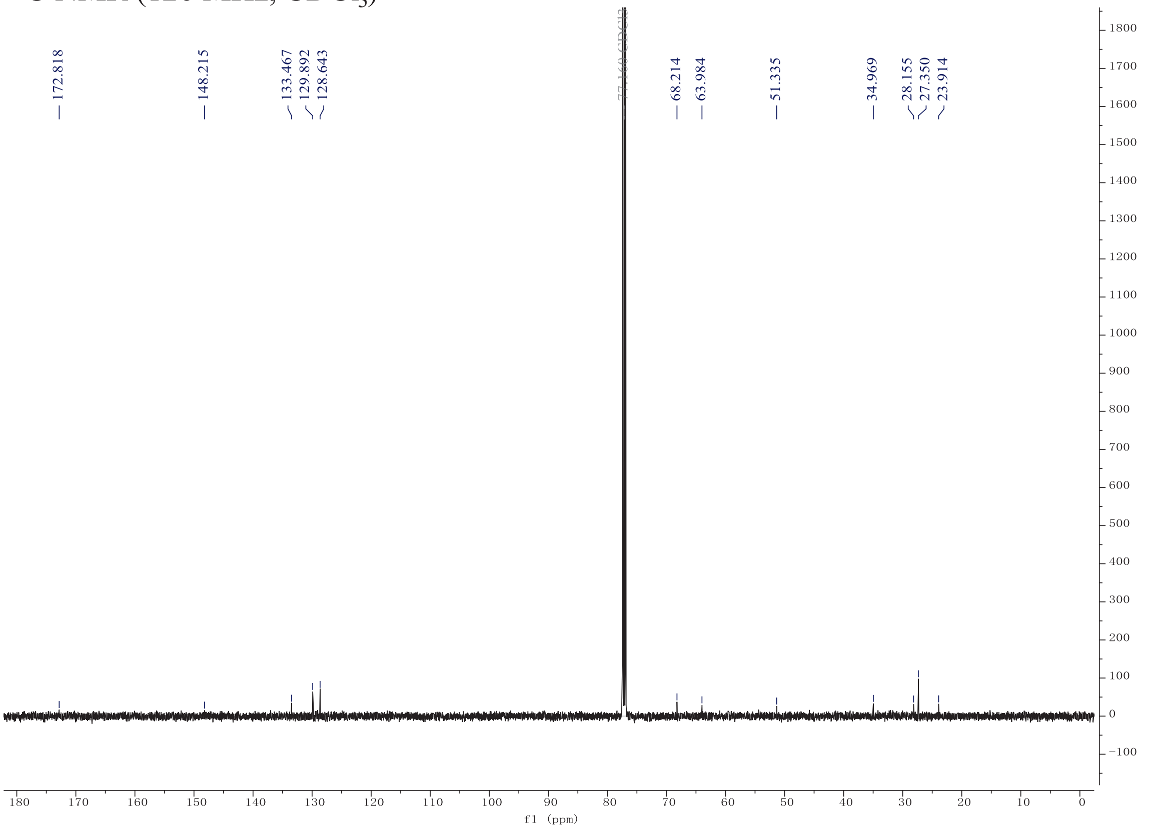

^{1}H NMR (500 MHz, $CDCl_3$)

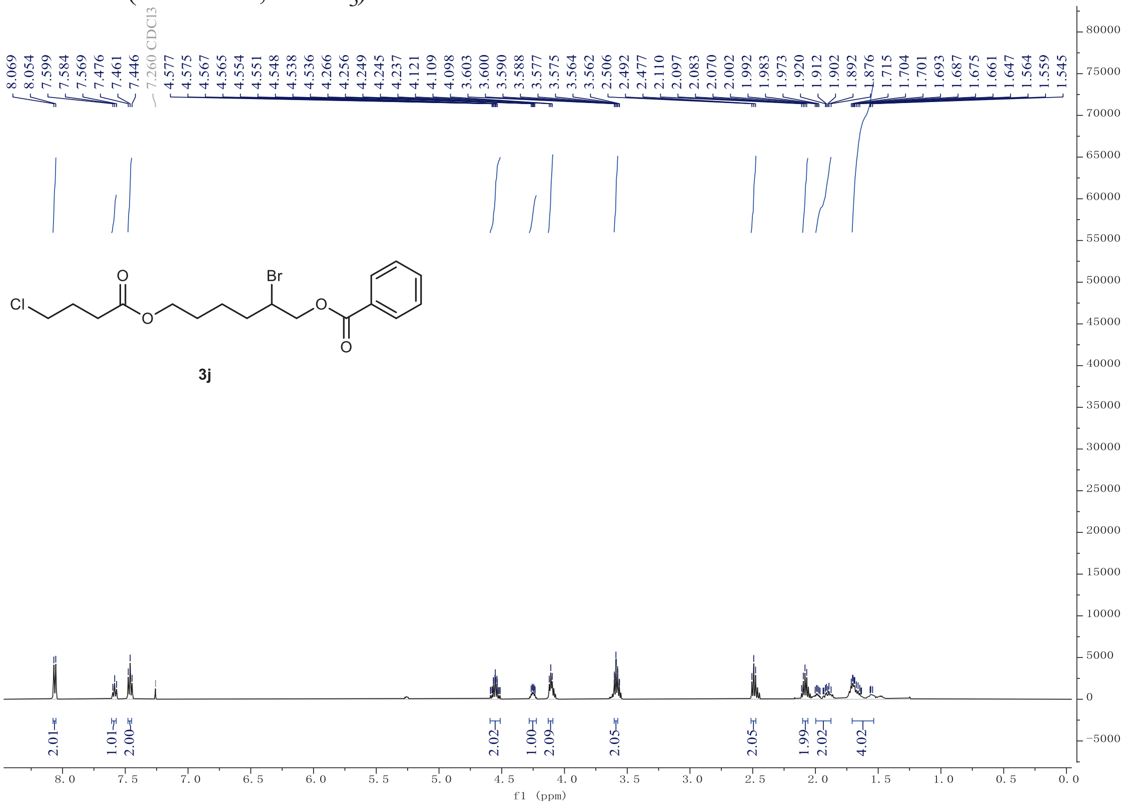

3j

^{13}C NMR (126 MHz, $CDCl_3$)

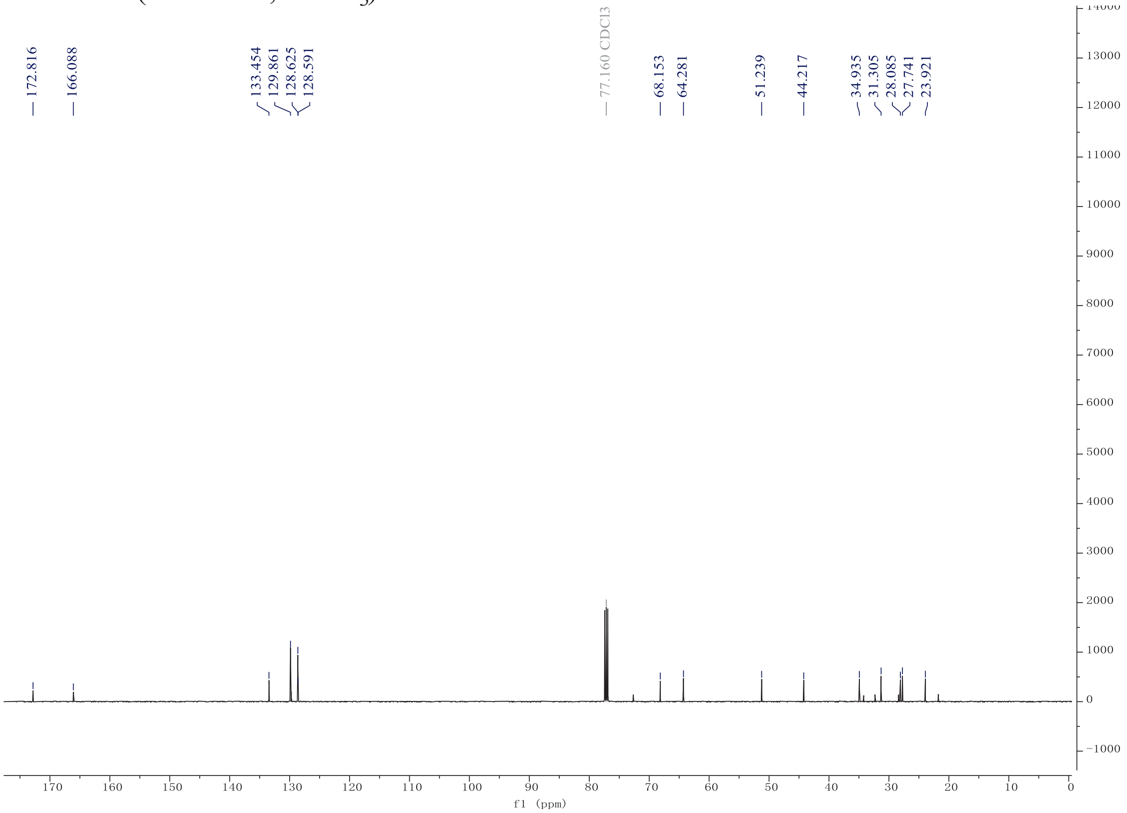

^{1}H NMR (500 MHz, $CDCl_3$)

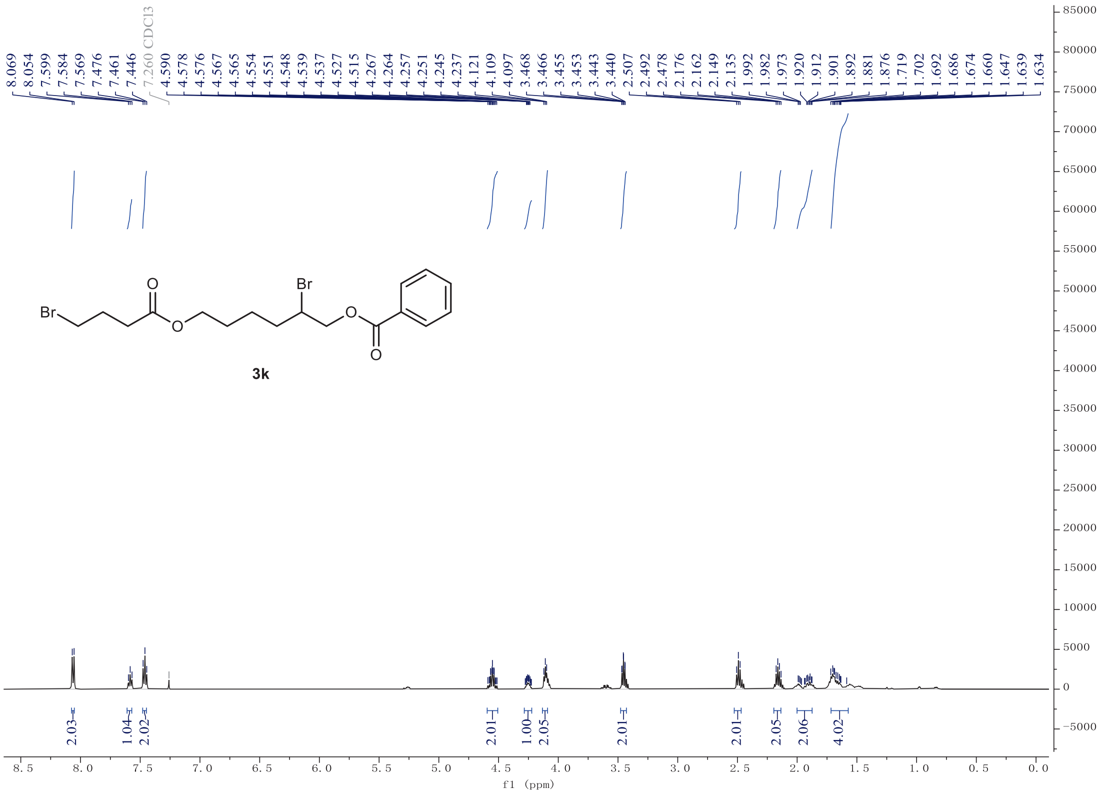

^{13}C NMR (126 MHz, $CDCl_3$)

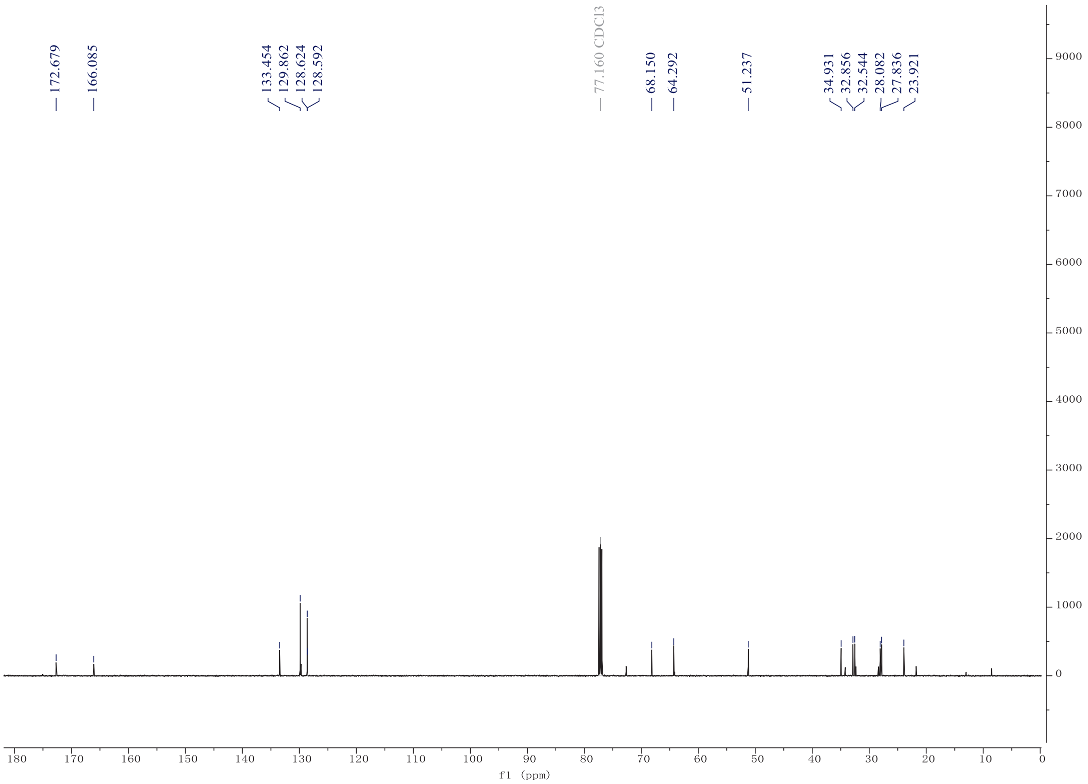

^{1}H NMR (500 MHz, $CDCl_3$)

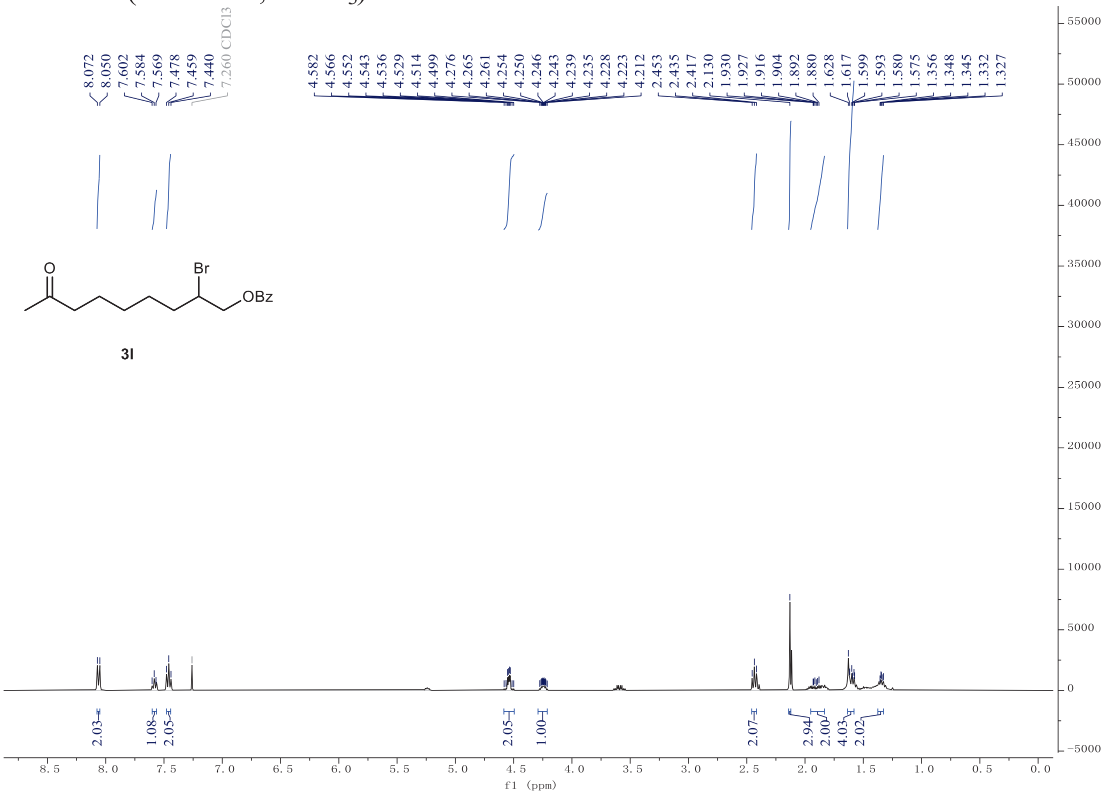

^{13}C NMR (126 MHz, $CDCl_3$)

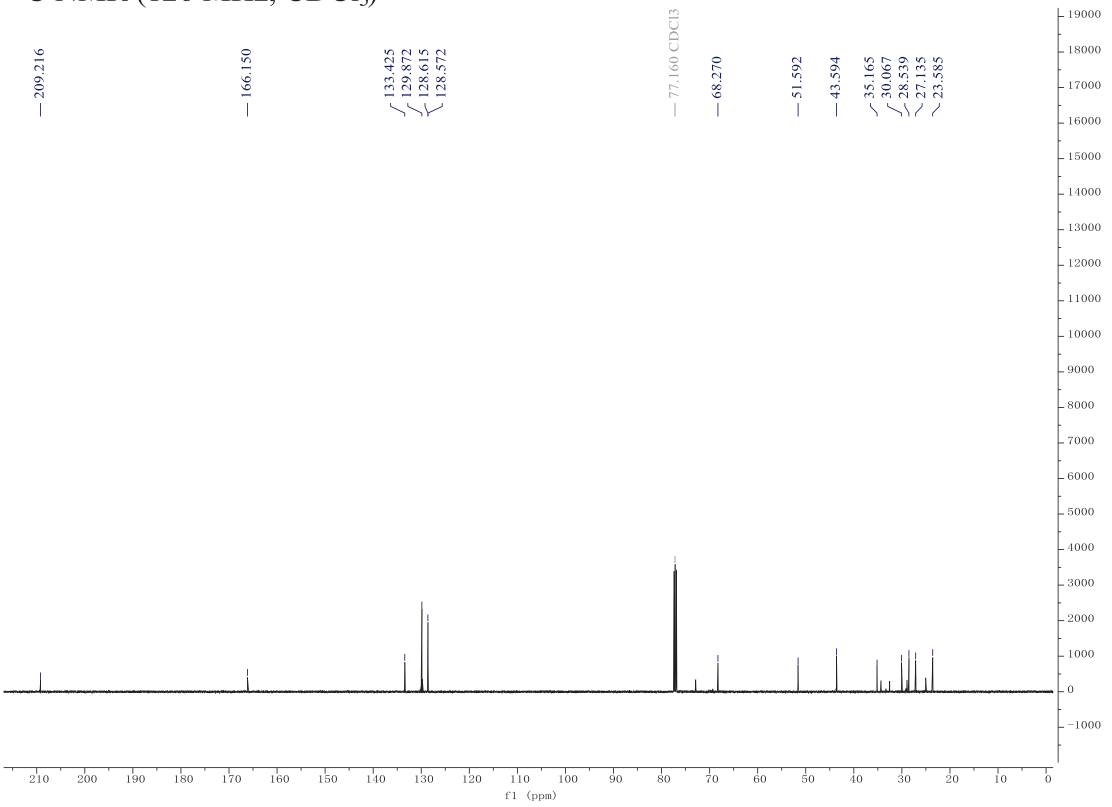

^{1}H NMR (500 MHz, $CDCl_3$)

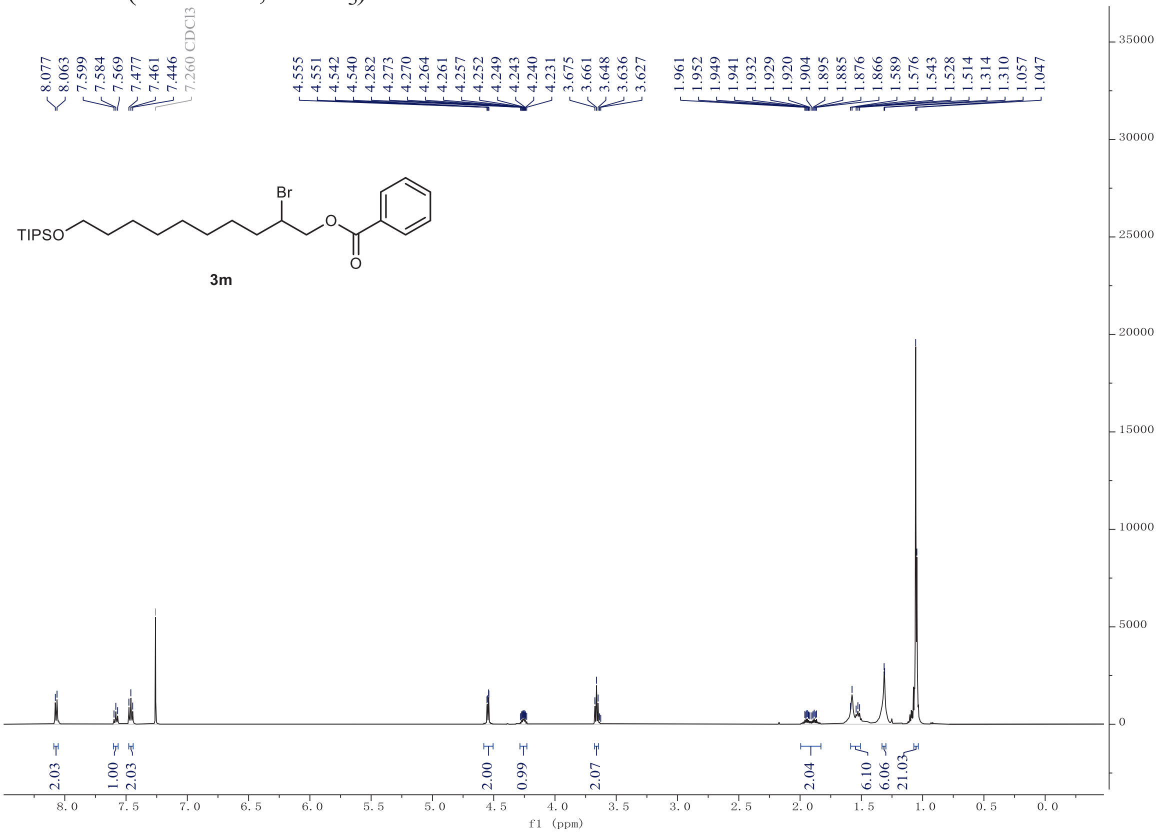

3m

^{13}C NMR (126 MHz, $CDCl_3$)

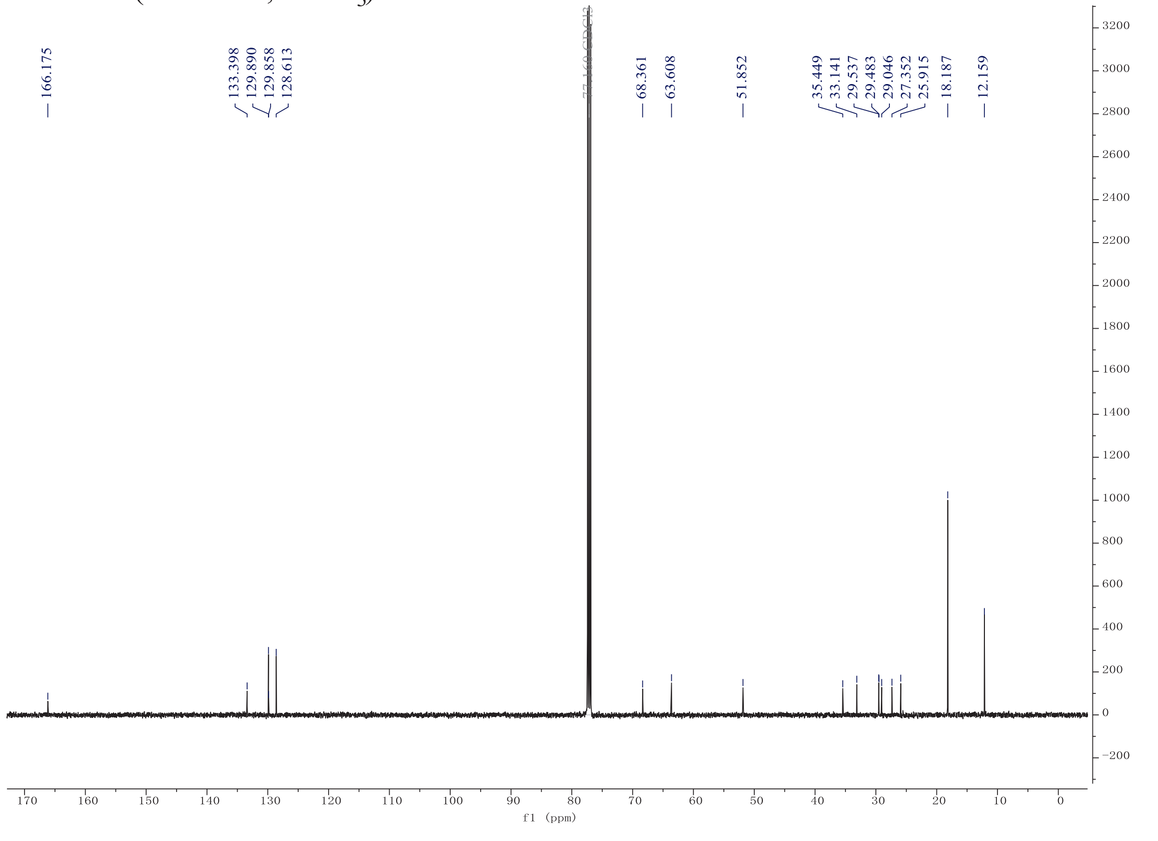

^1H NMR (500 MHz, $CDCl_3$)

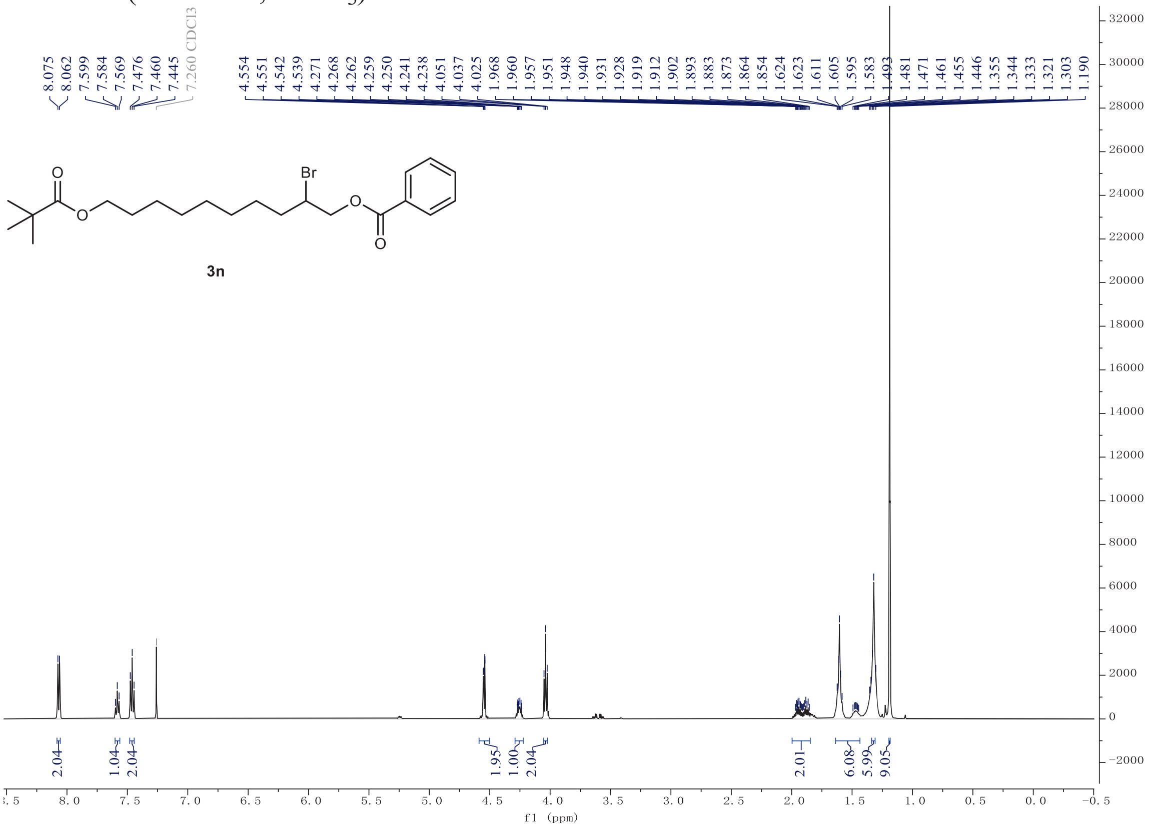

^{13}C NMR (126 MHz, $CDCl_3$)

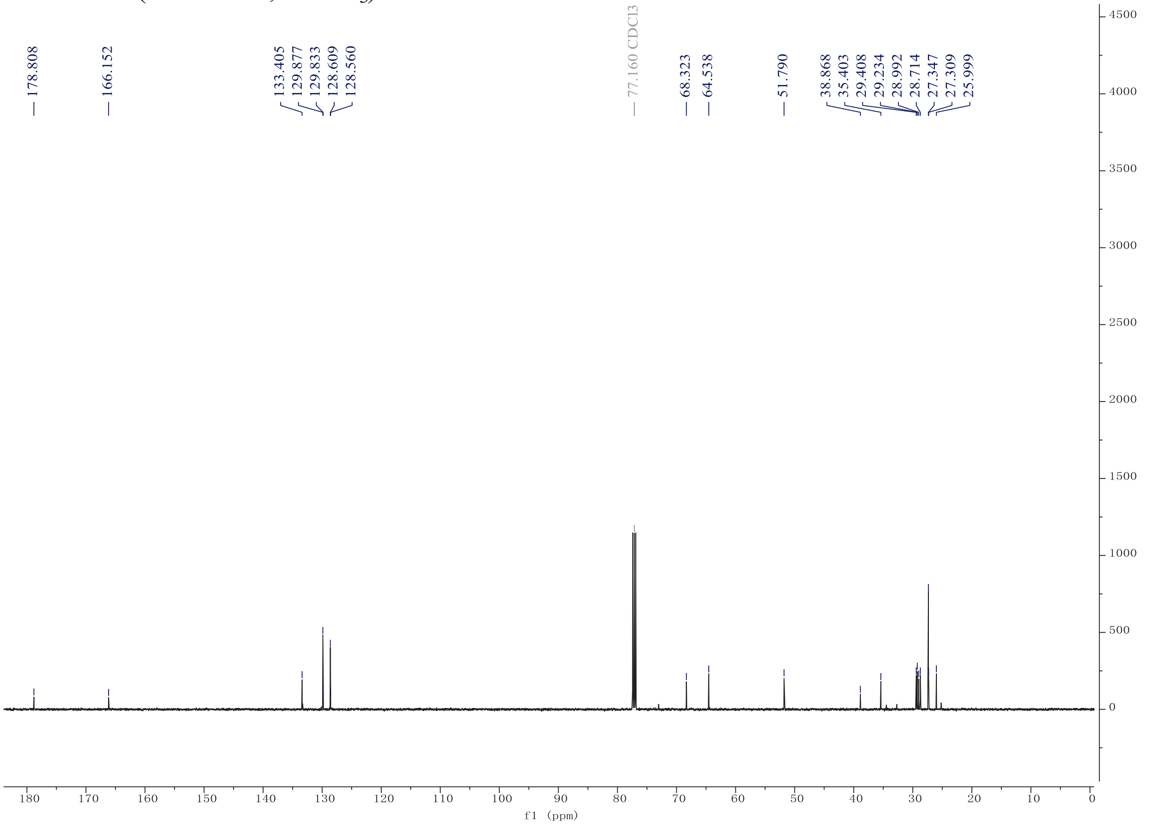

^{1}H NMR (500 MHz, $CDCl_3$)

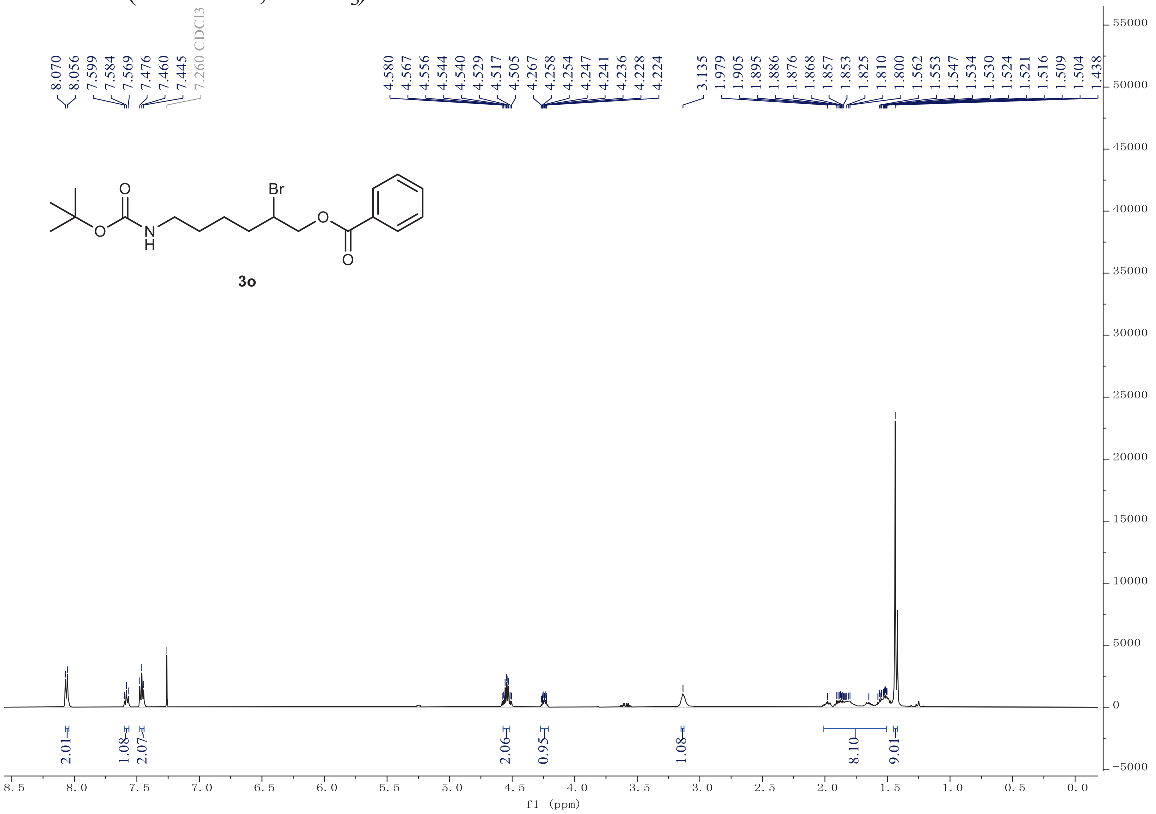

^{13}C NMR (126 MHz, $CDCl_3$)

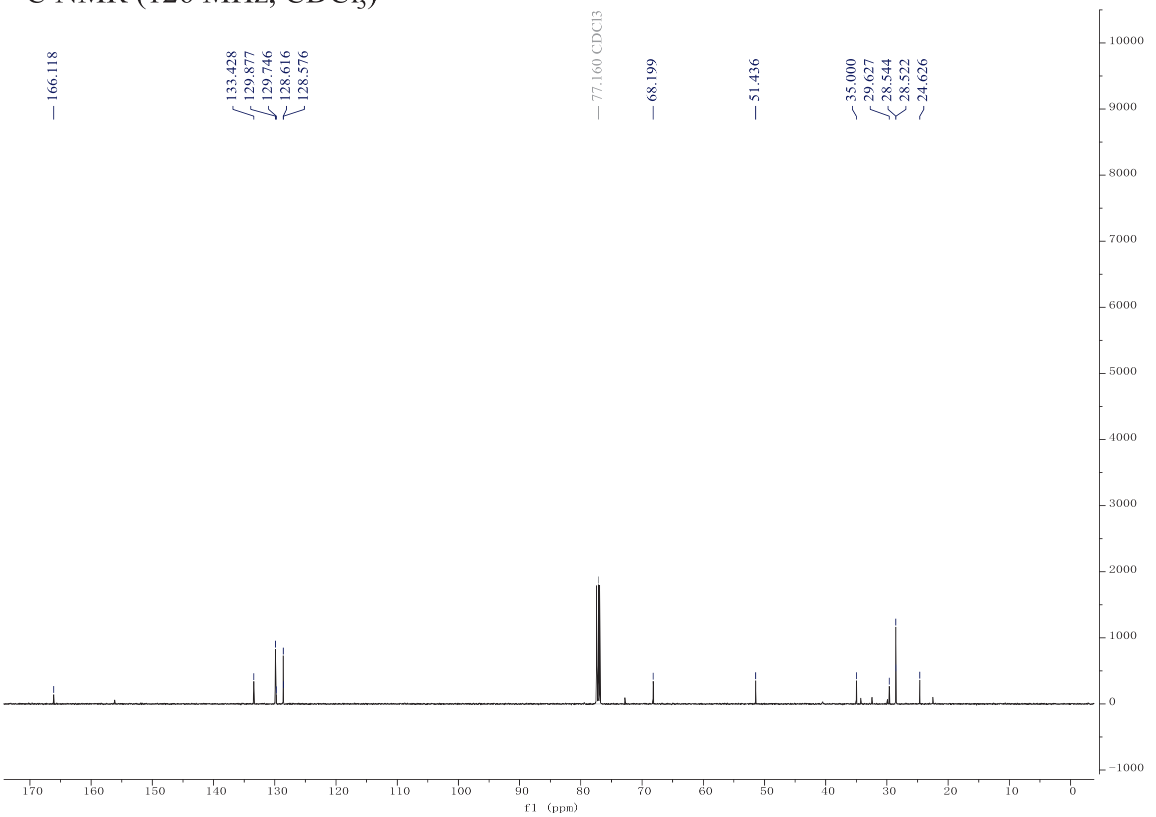

^{1}H NMR (400 MHz, $CDCl_3$)

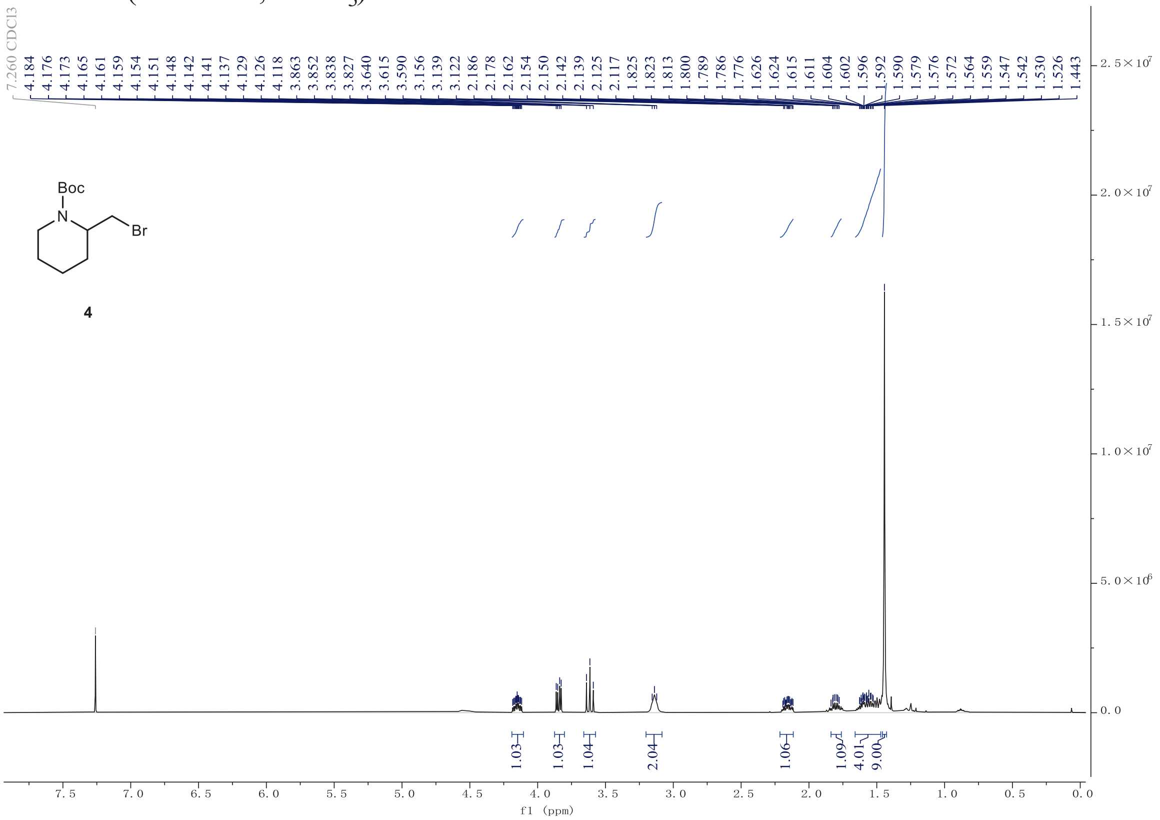

^{13}C NMR (126 MHz, $CDCl_3$)

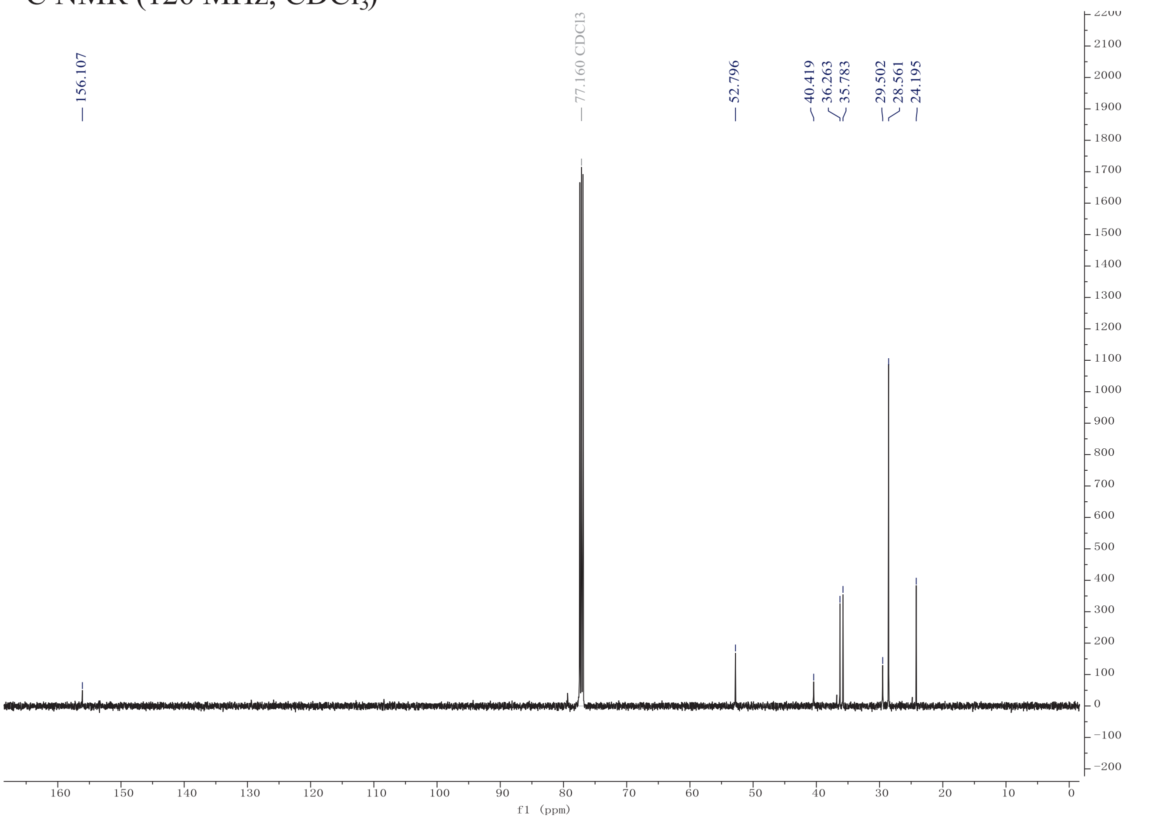

^{1}H NMR (500 MHz, $CDCl_3$)

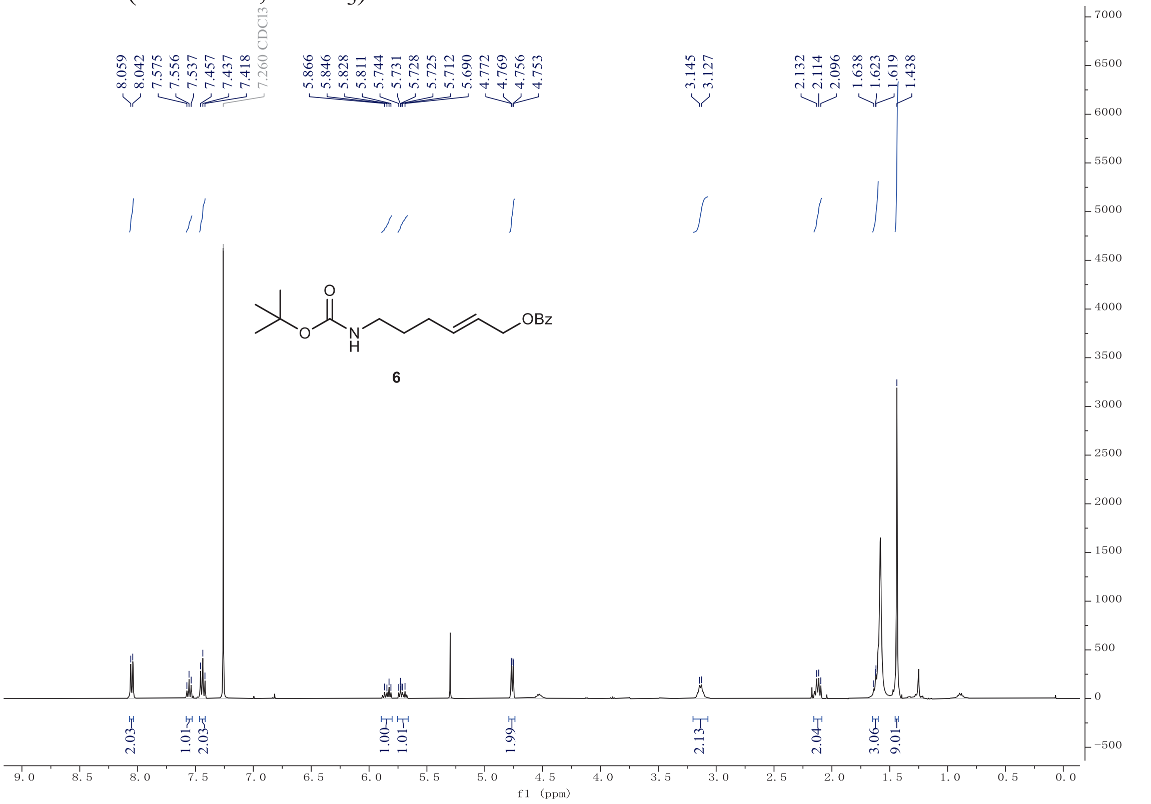

^{13}C NMR (126 MHz, $CDCl_3$)

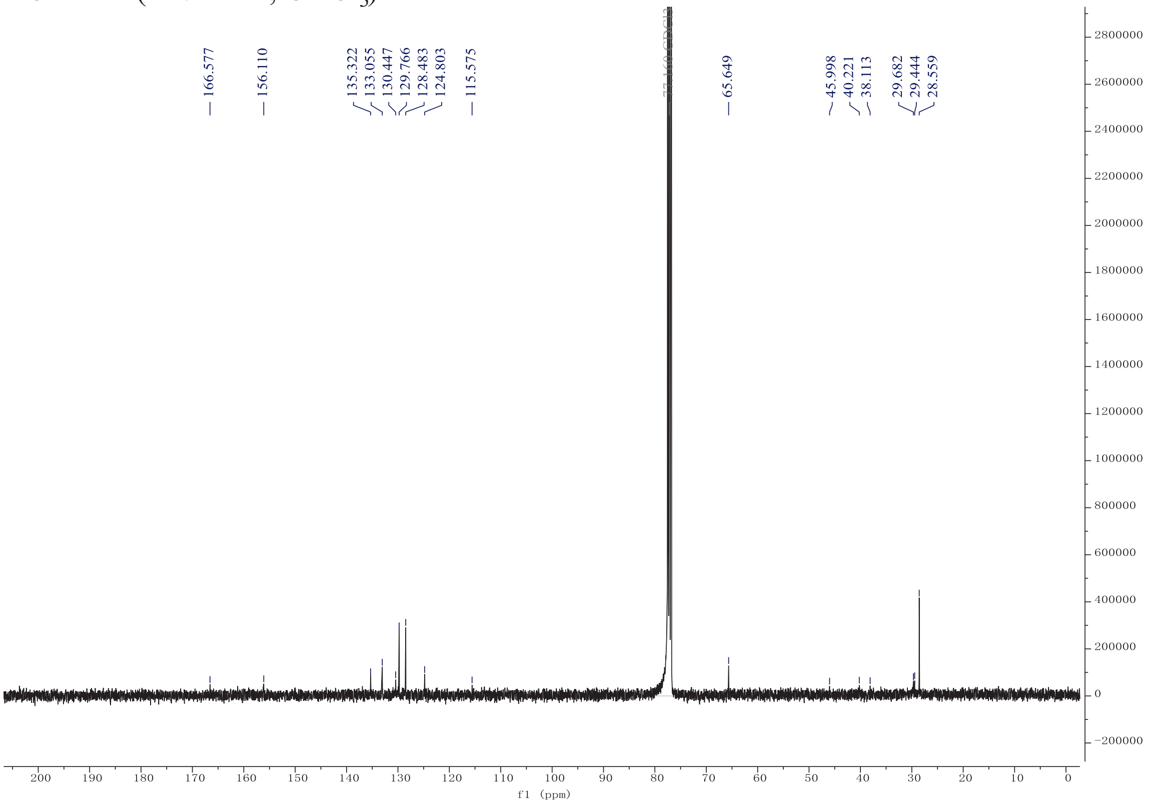

^{1}H NMR (500 MHz, $CDCl_3$)

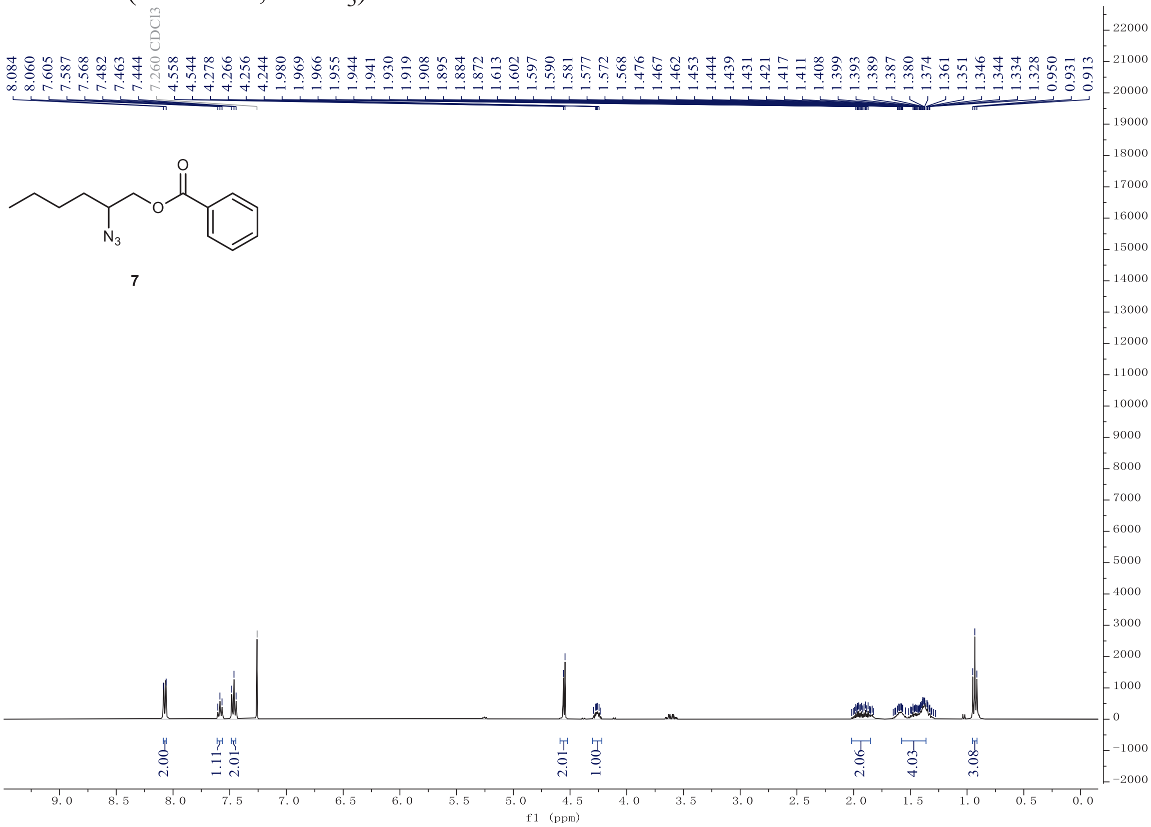

^{13}C NMR (126 MHz, $CDCl_3$)

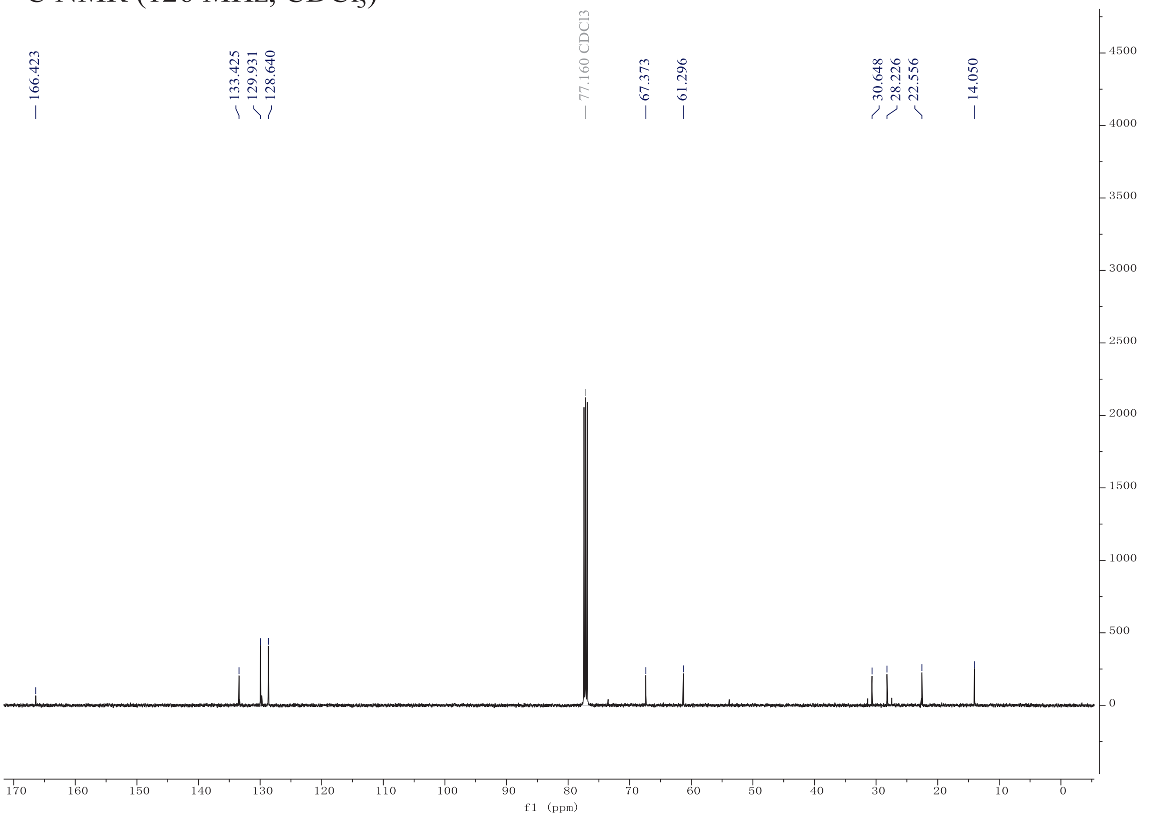

^{1}H NMR (500 MHz, $CDCl_3$)

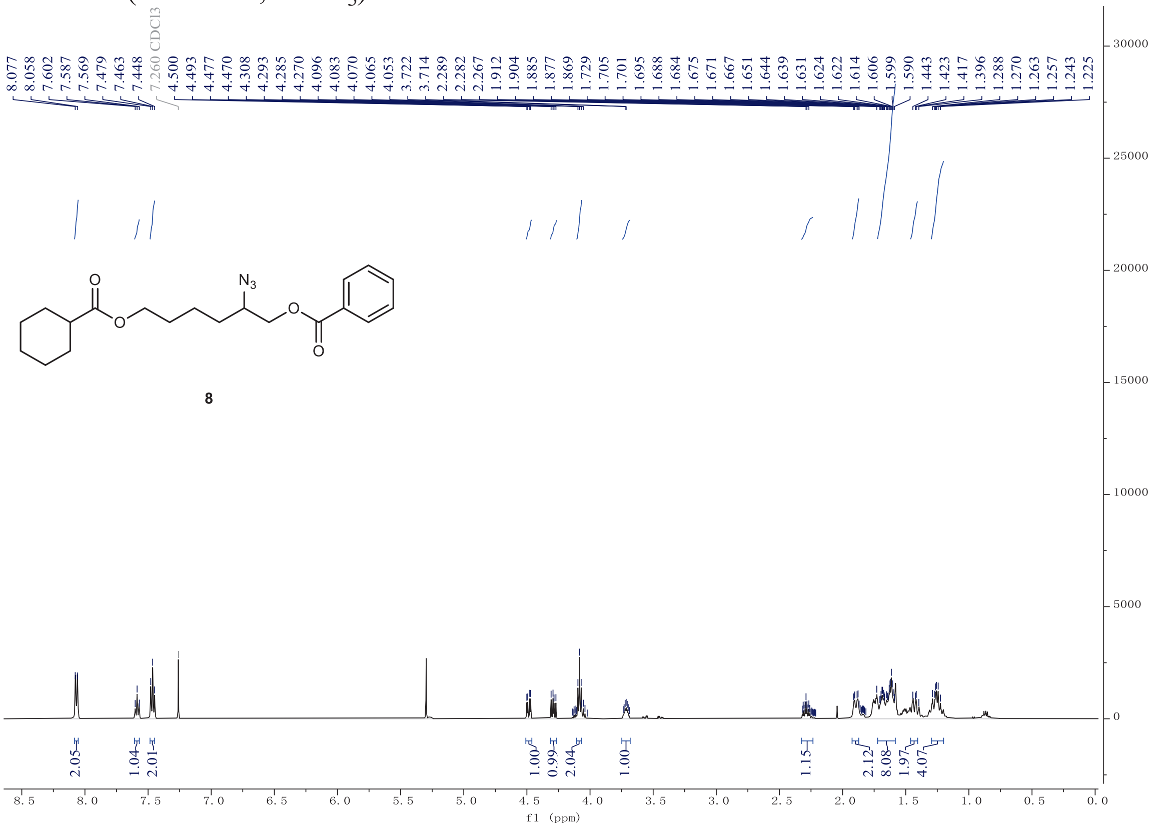

^{13}C NMR (126 MHz, $CDCl_3$)

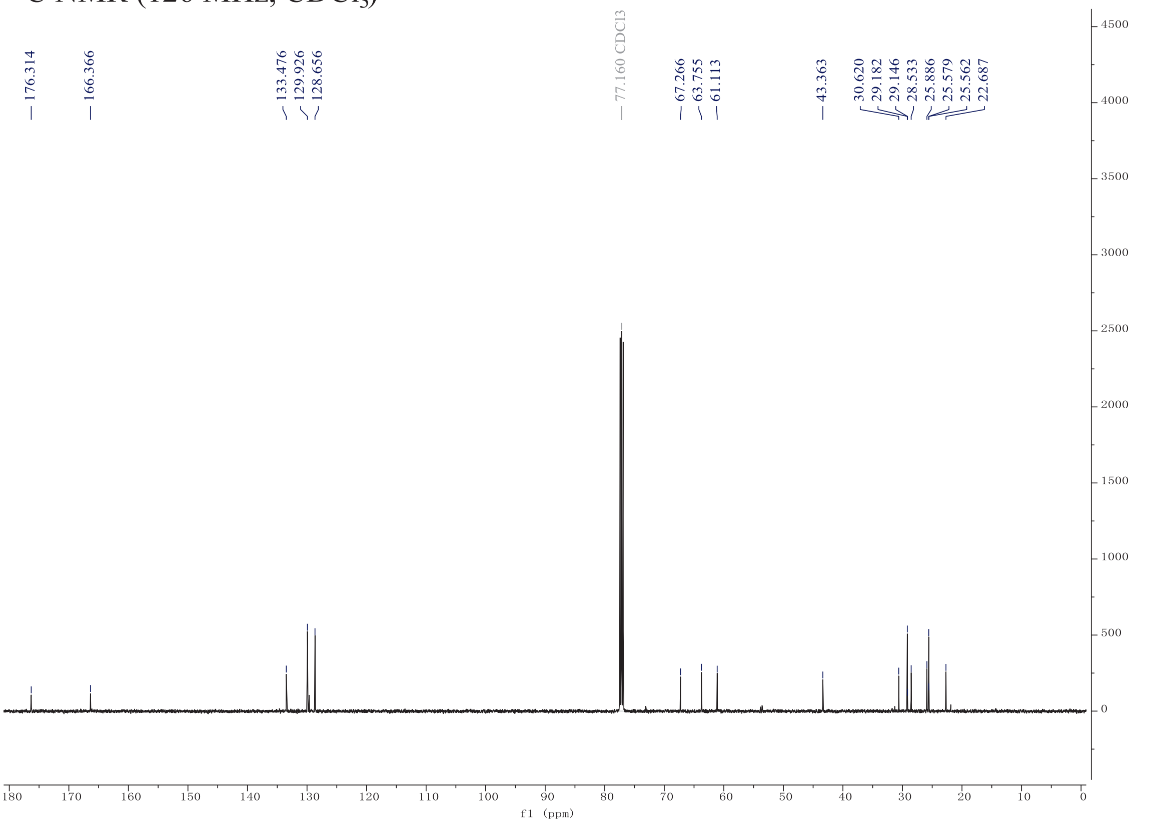

^{1}H NMR (500 MHz, $CDCl_3$)

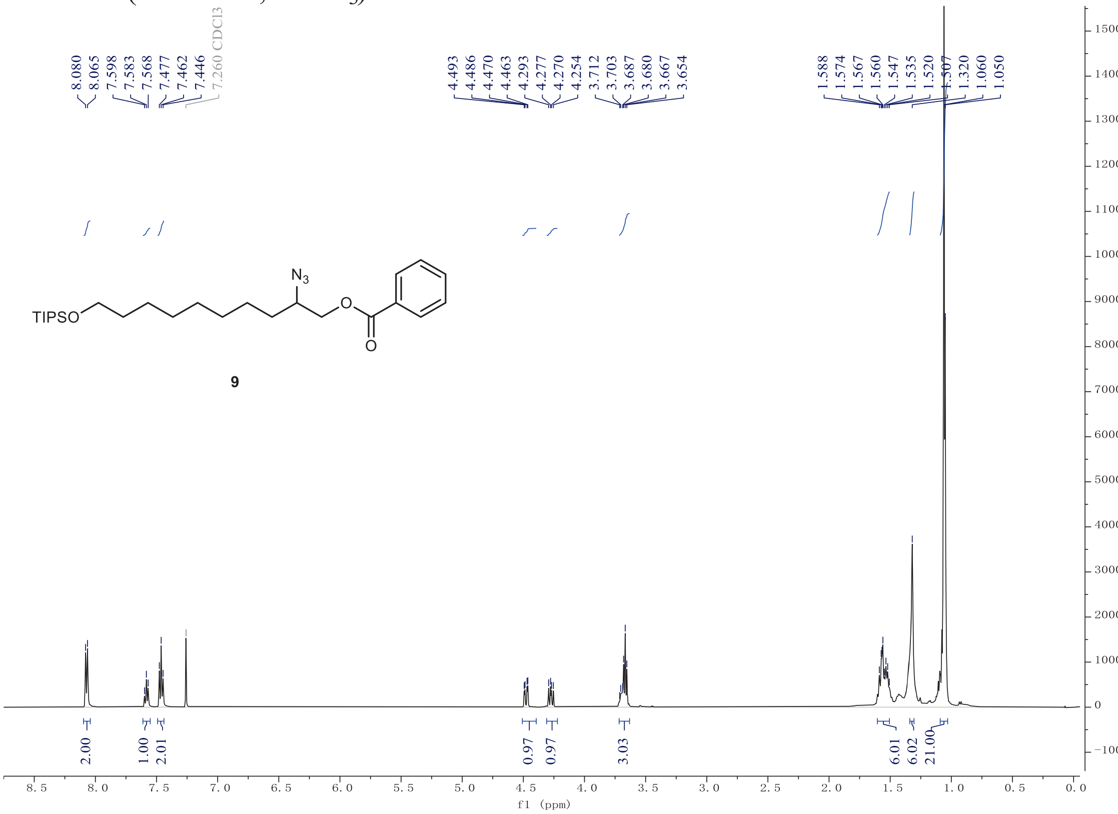

^{13}C NMR (126 MHz, $CDCl_3$)

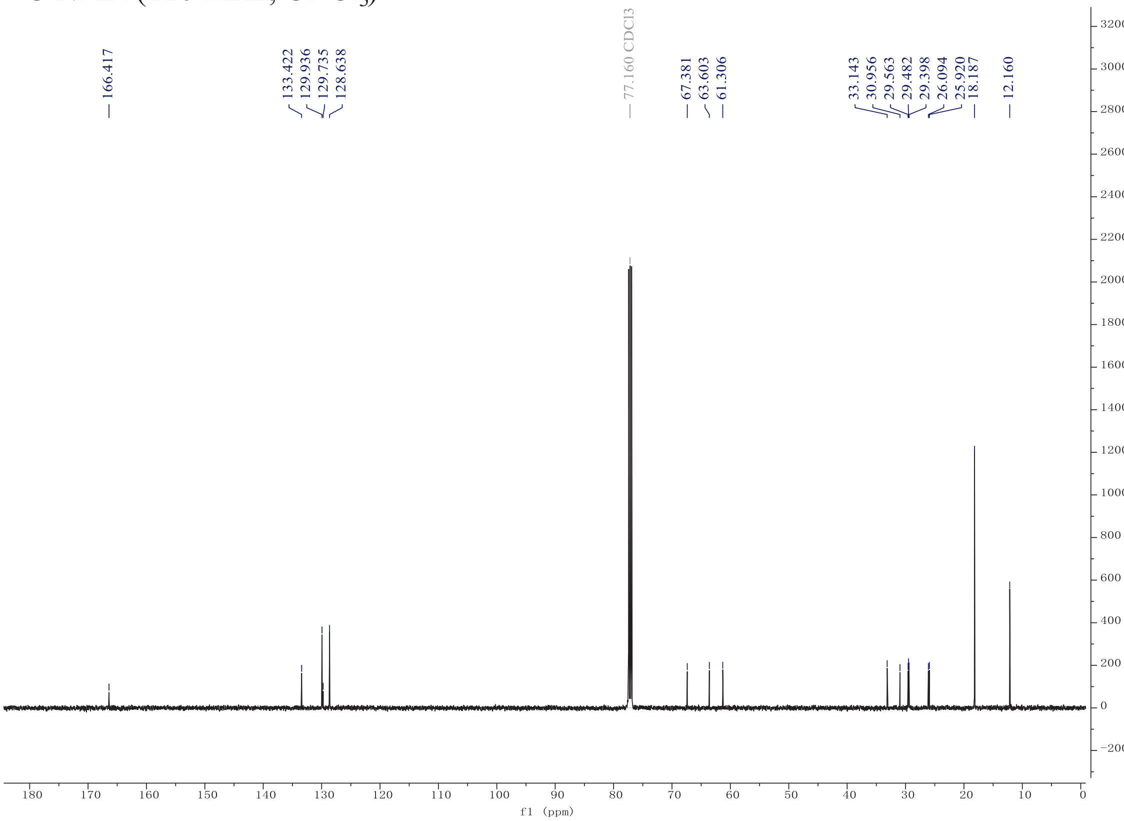

^{1}H NMR (500 MHz, $CDCl_3$)

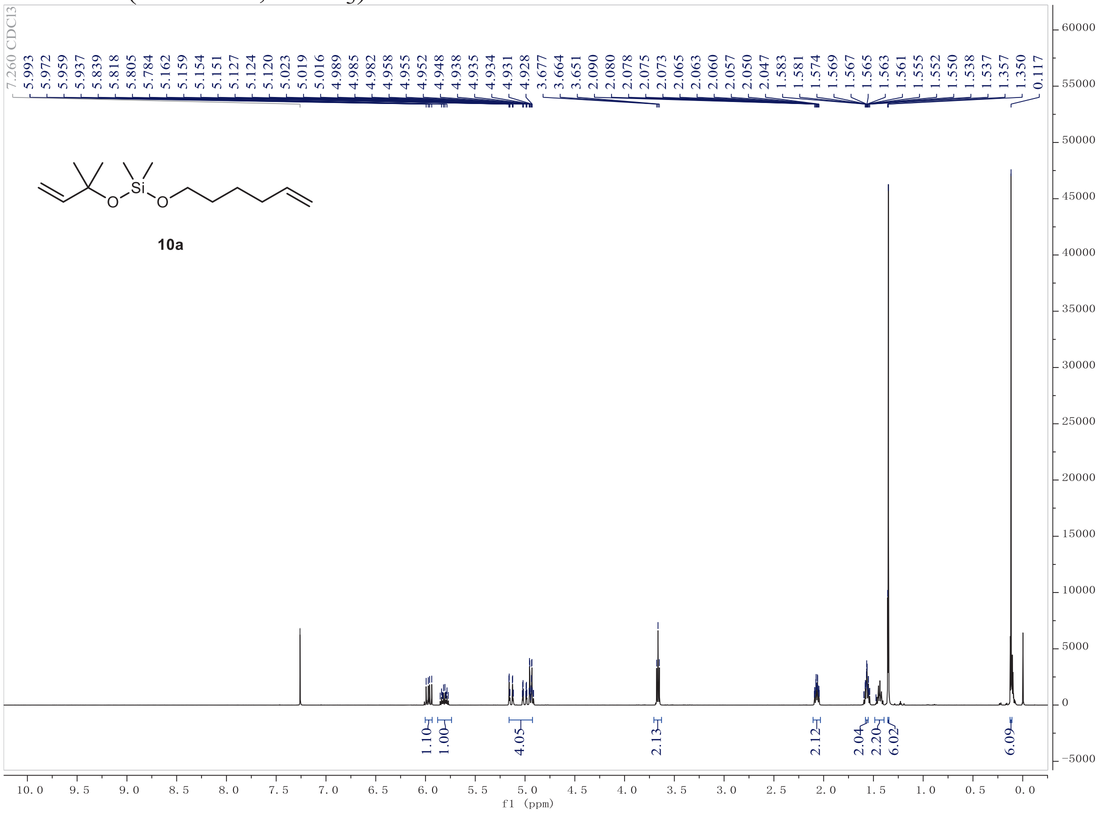

^{13}C NMR (126 MHz, $CDCl_3$)

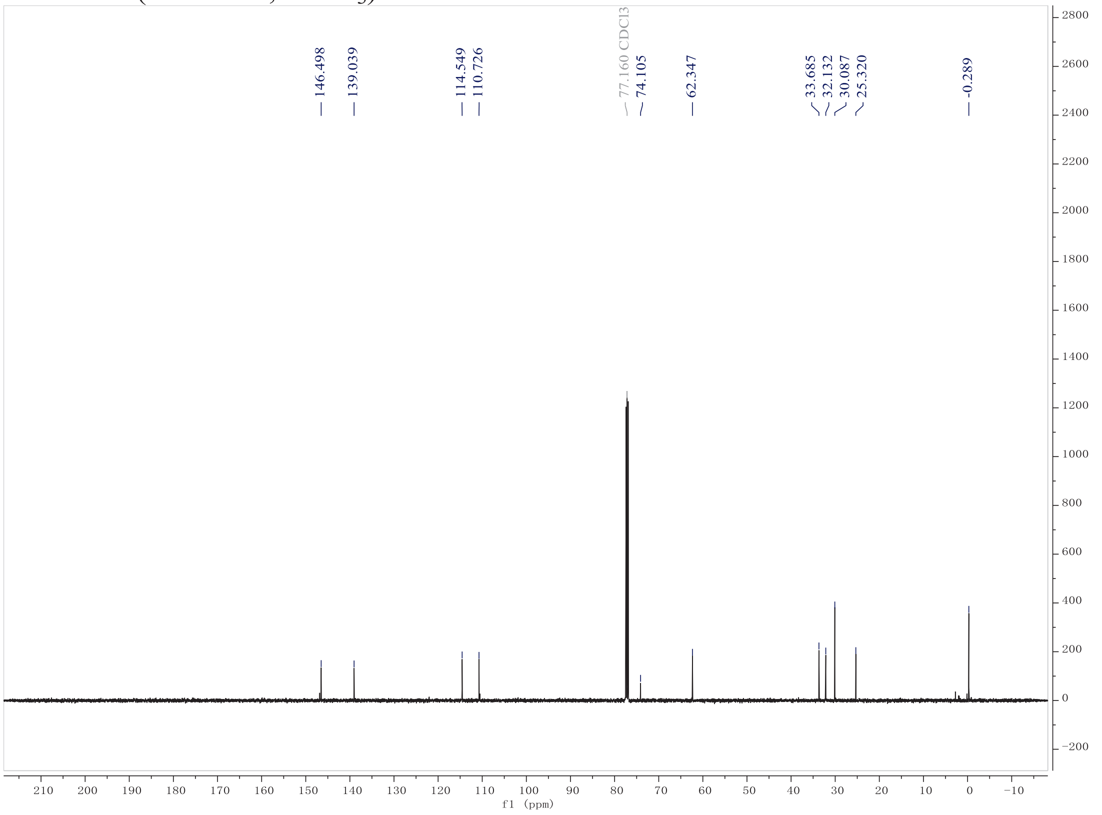

^{1}H NMR (500 MHz, $CDCl_3$)

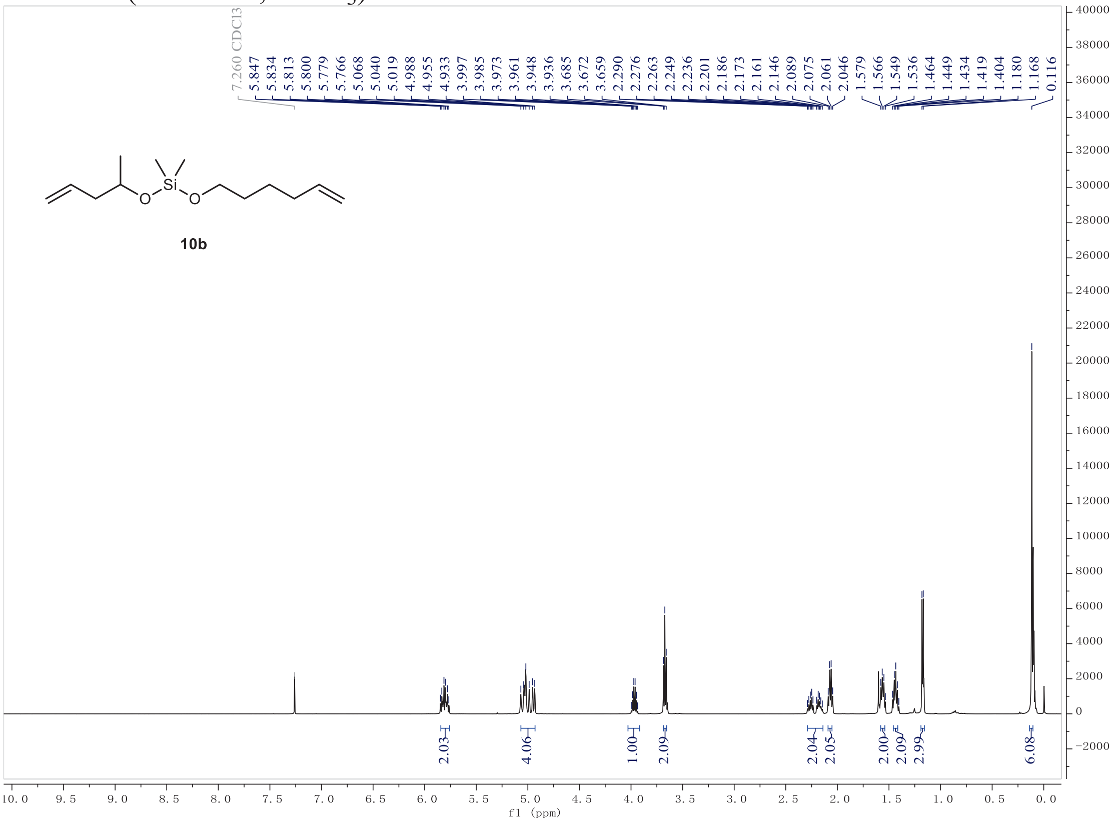

^{13}C NMR (126 MHz, $CDCl_3$)

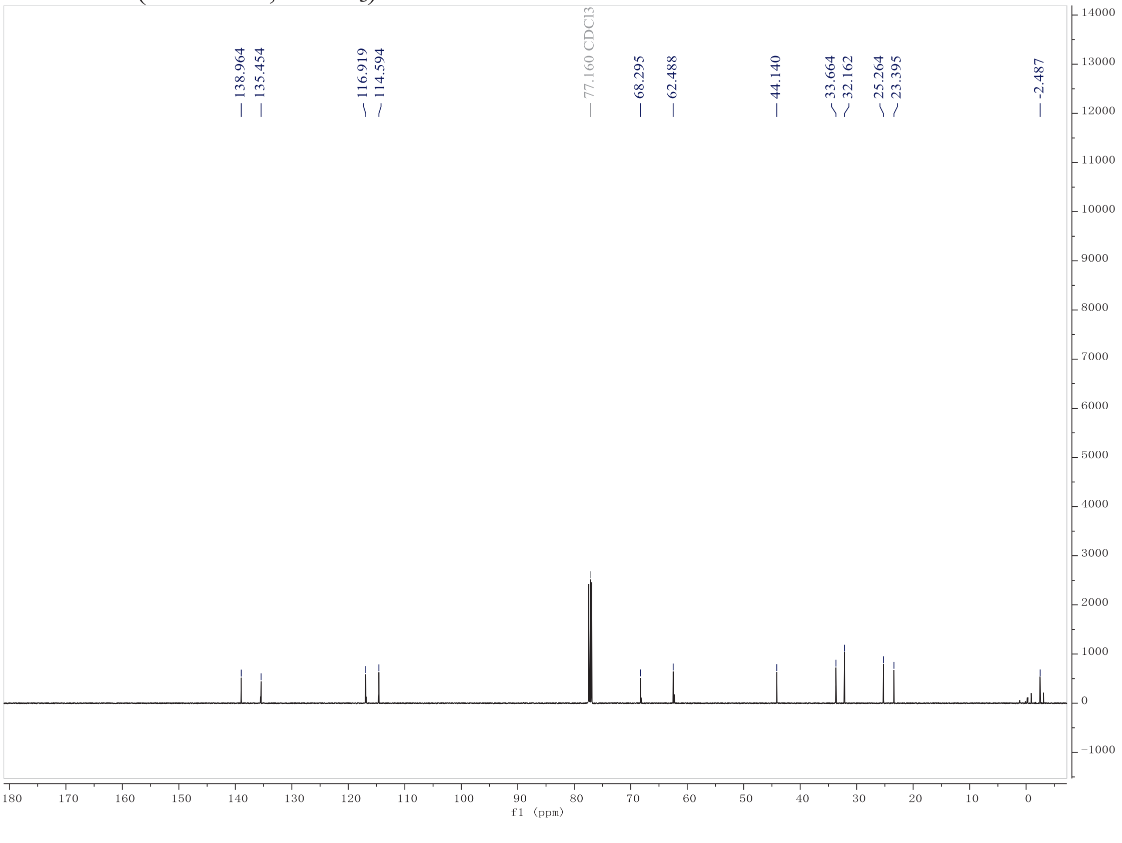

^{1}H NMR (500 MHz, $CDCl_3$)

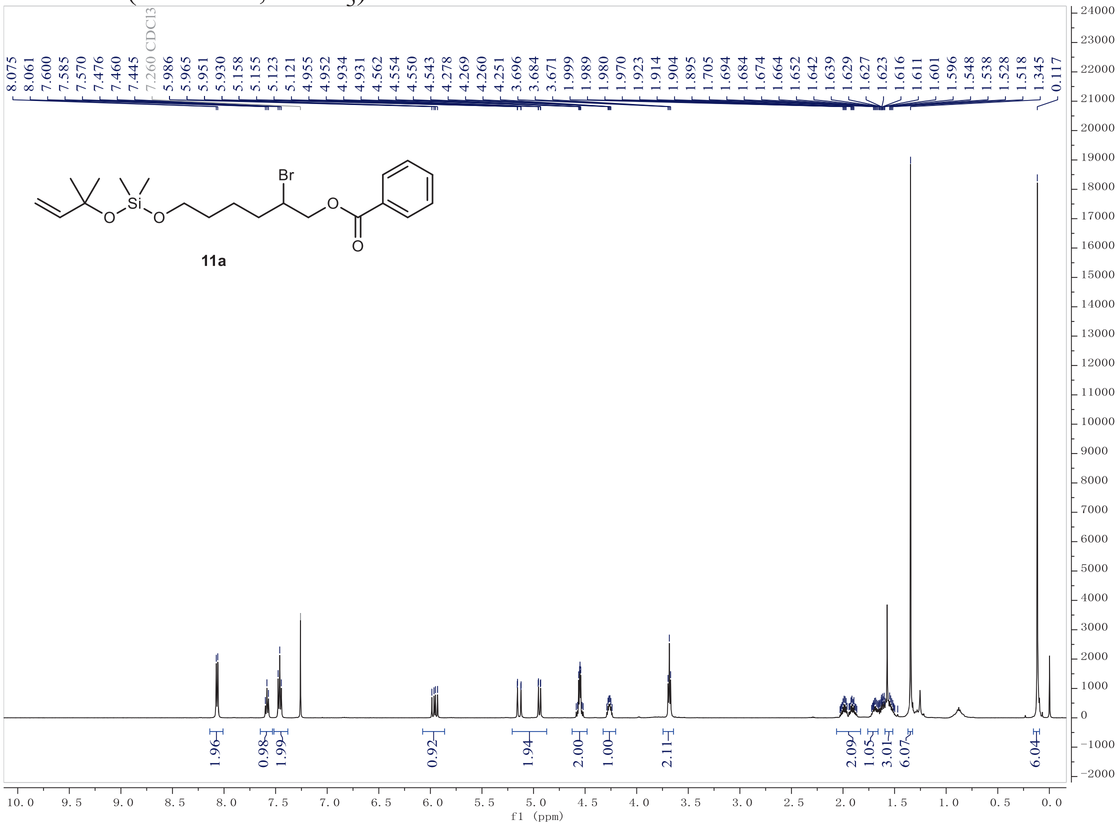

^{13}C NMR (126 MHz, $CDCl_3$)

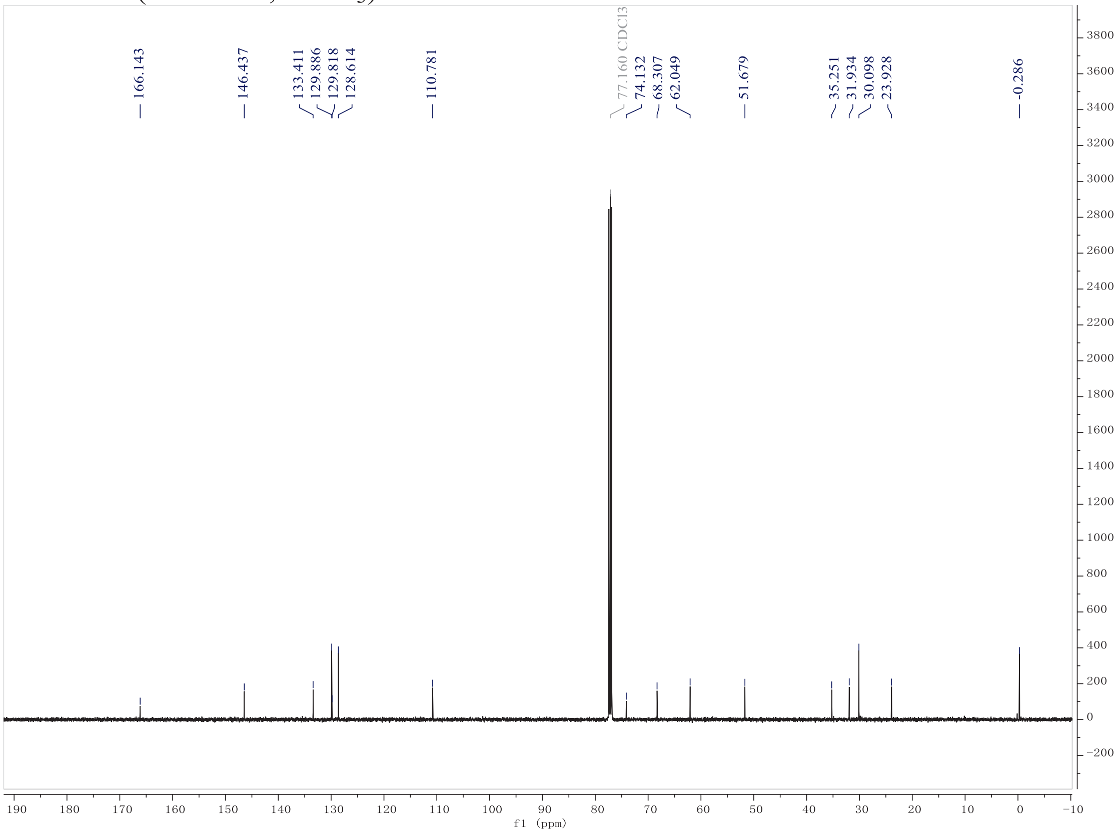

^{1}H NMR (500 MHz, $CDCl_3$)

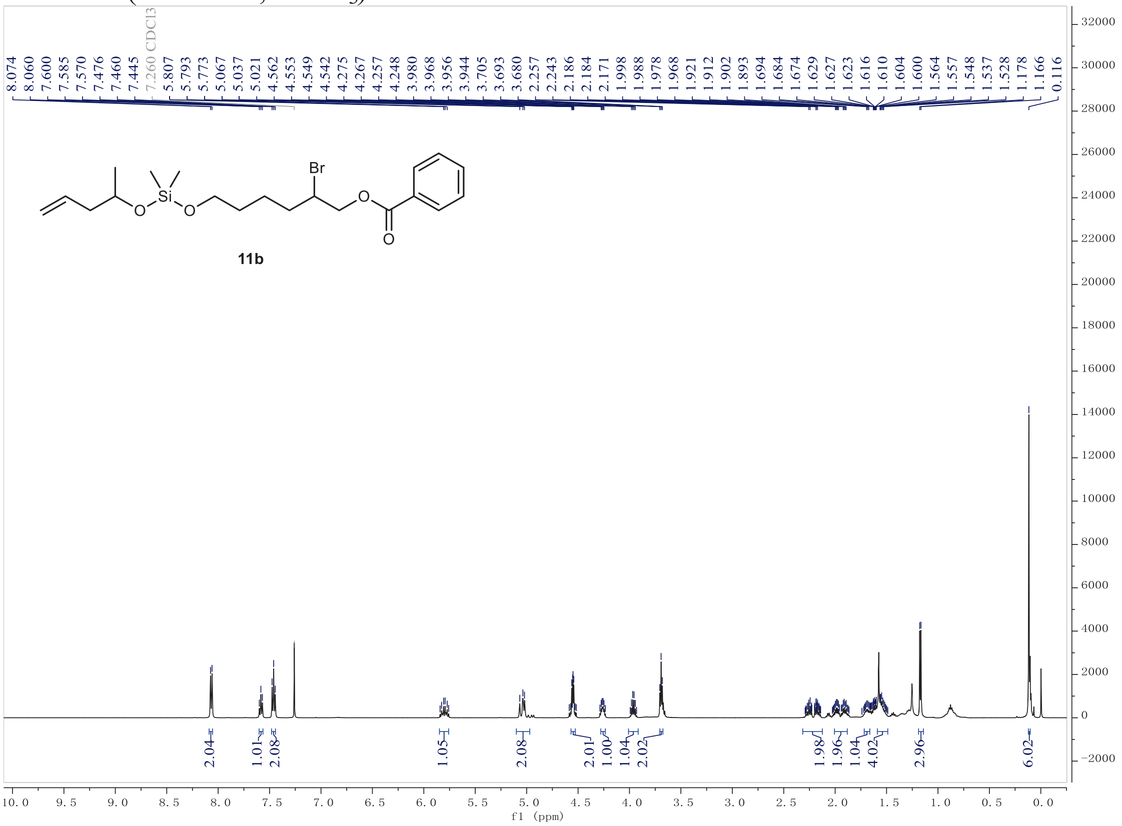

^{13}C NMR (126 MHz, $CDCl_3$)

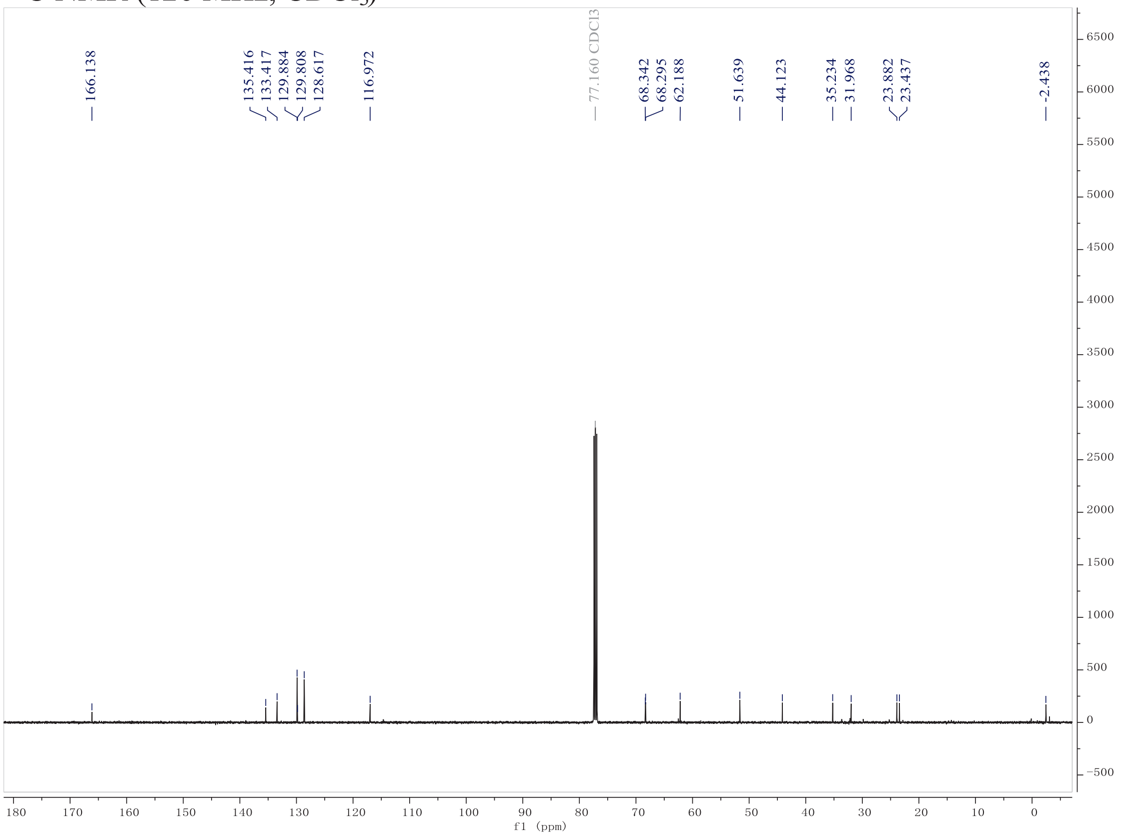

^{1}H NMR (500 MHz, $CDCl_3$)

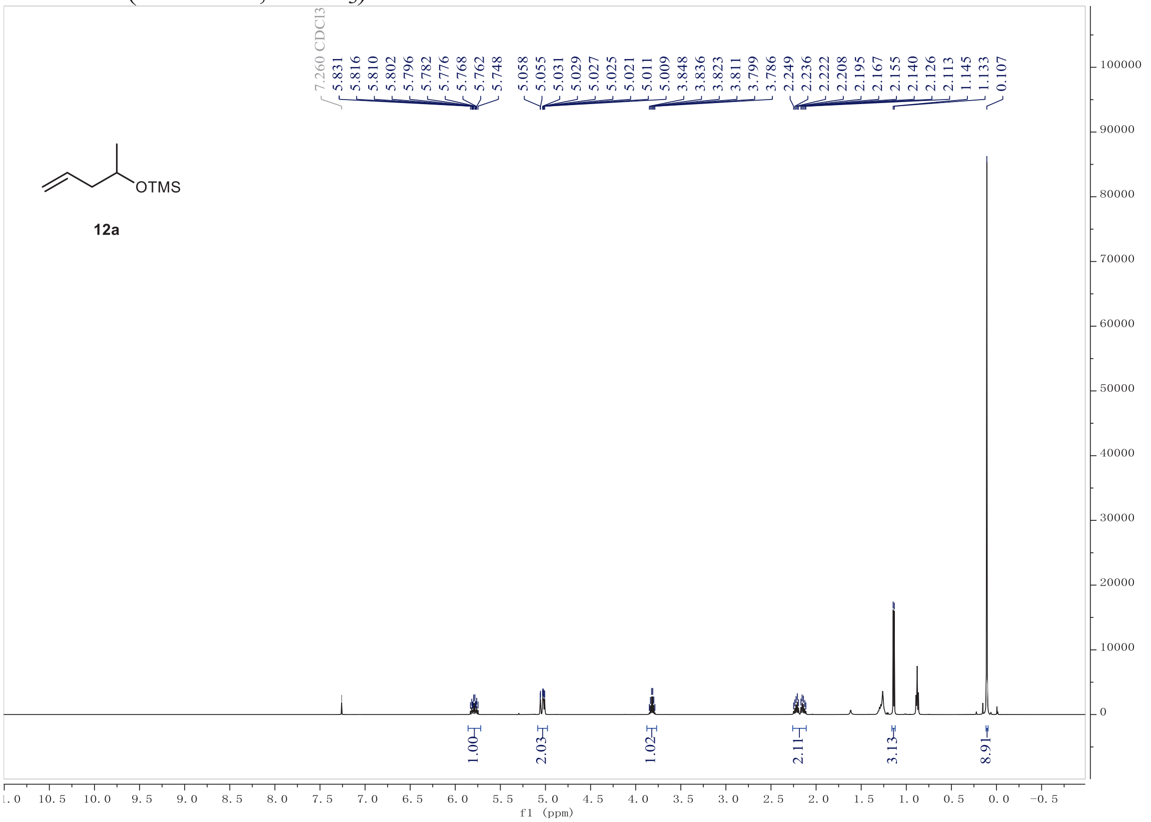

^{13}C NMR (126 MHz, $CDCl_3$)

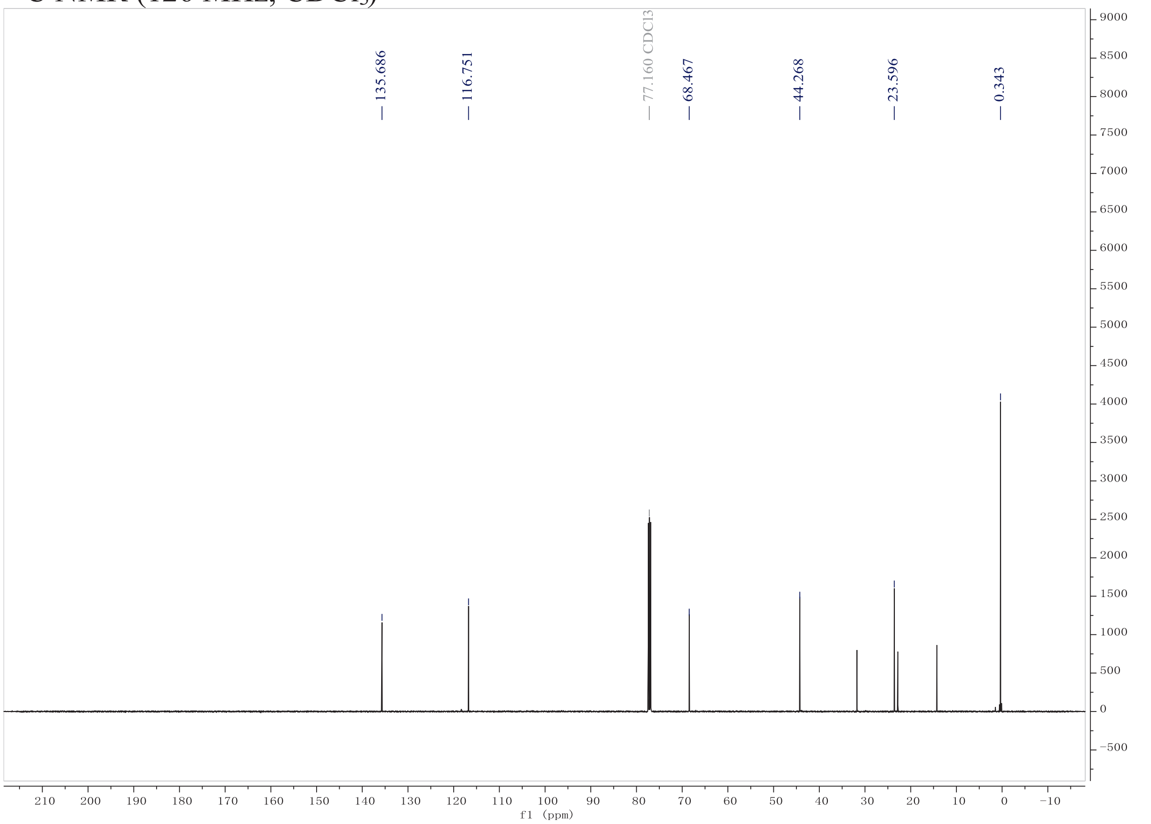

^{1}H NMR (500 MHz, $CDCl_3$)

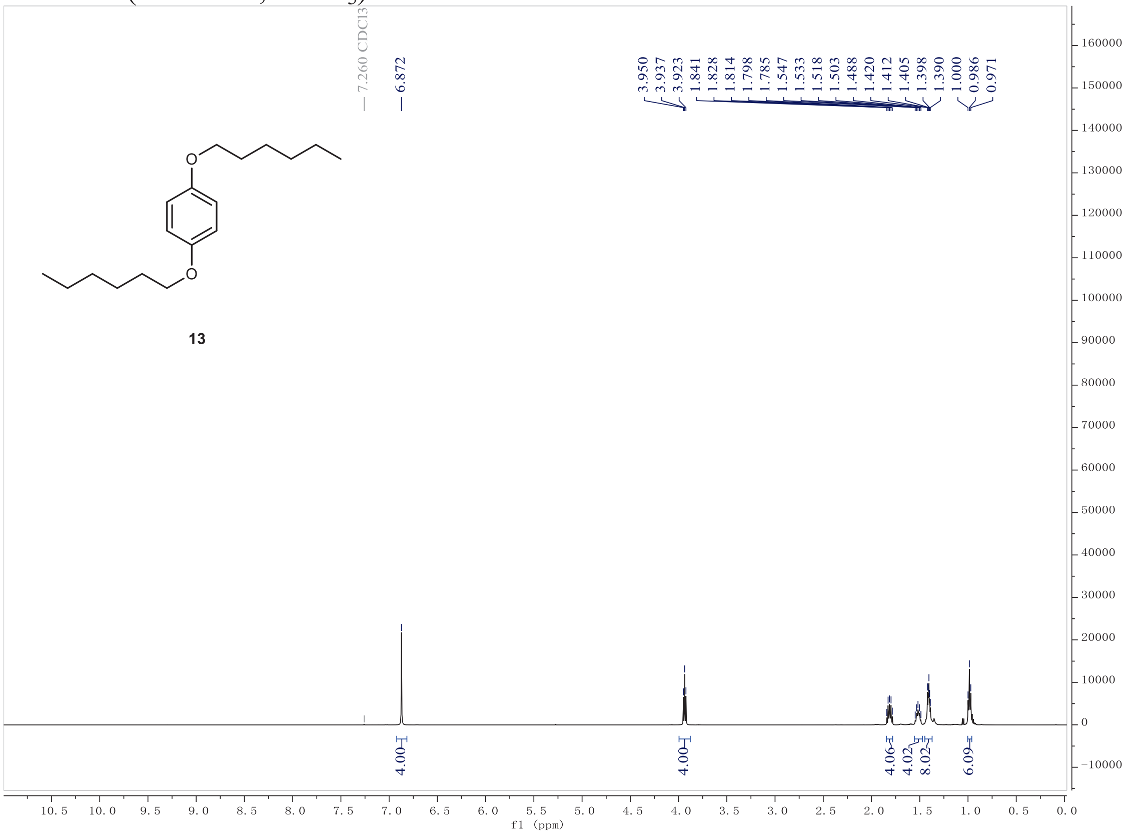

^{13}C NMR (126 MHz, $CDCl_3$)

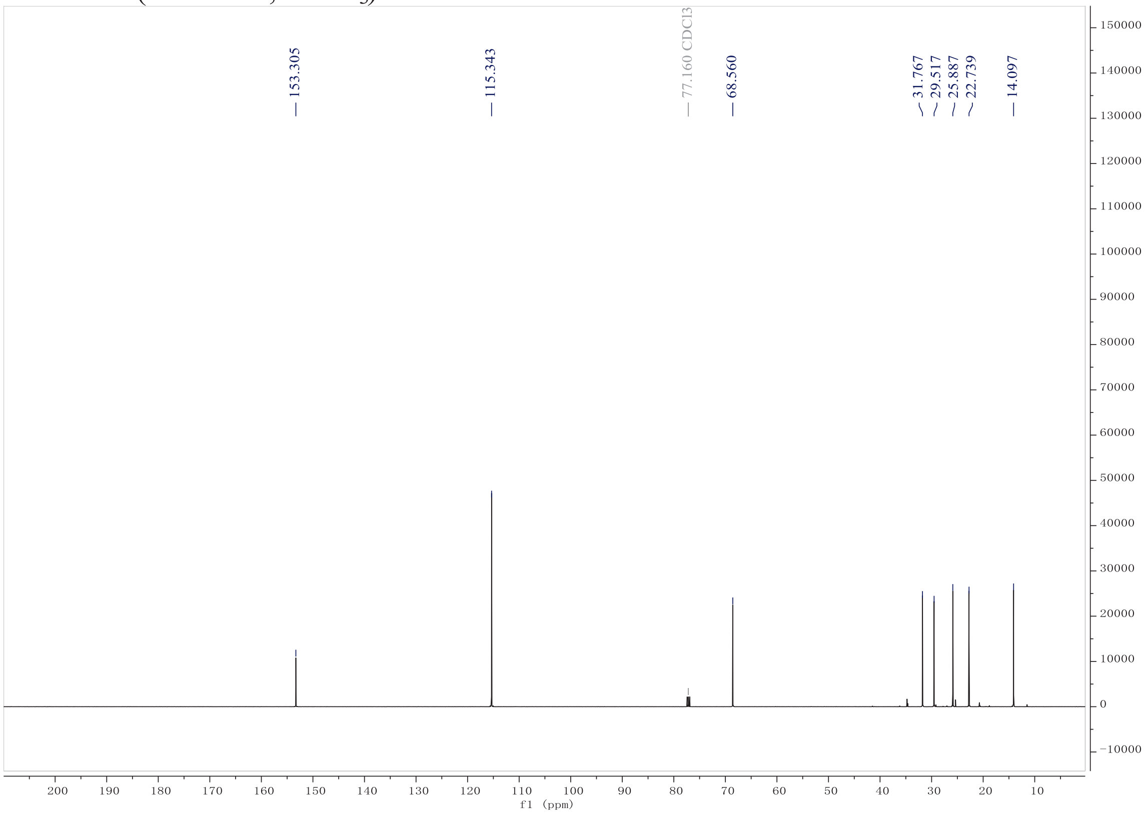

^{1}H NMR (500 MHz, $CDCl_3$)

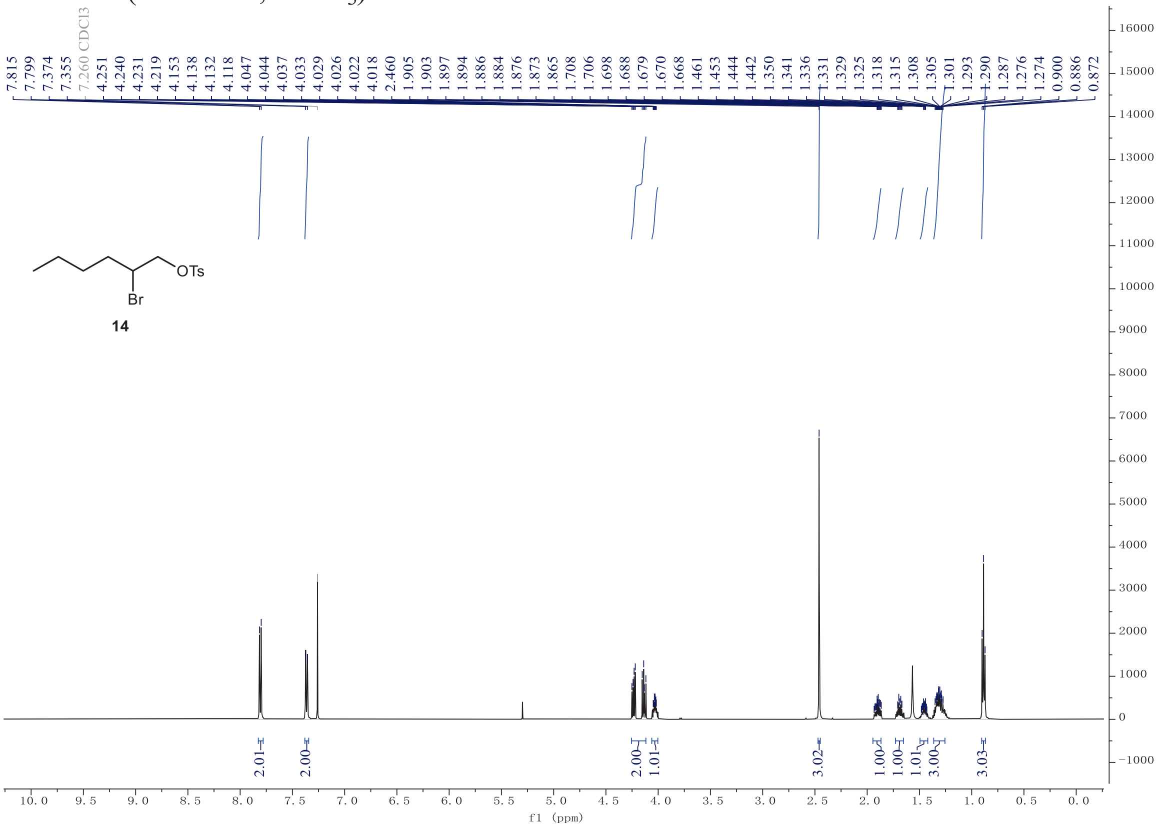

^{13}C NMR (126 MHz, $CDCl_3$)

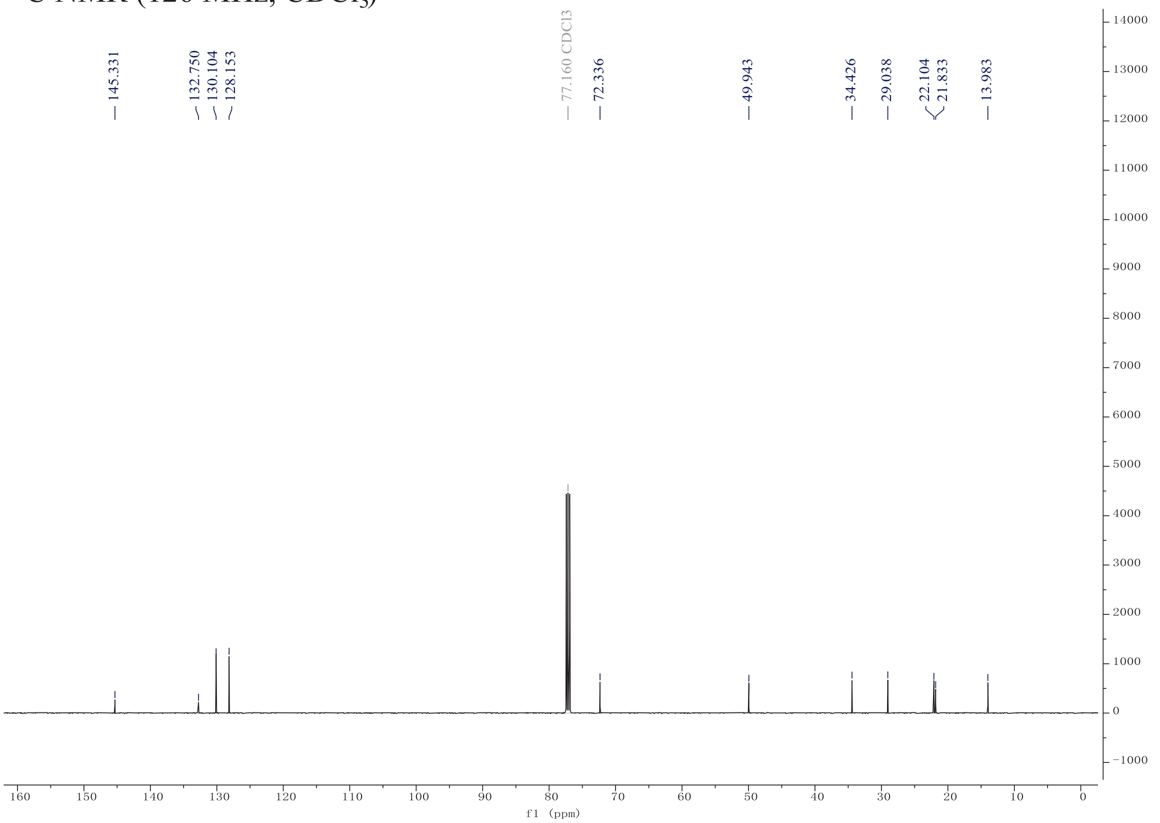

^{1}H NMR (500 MHz, $CDCl_3$)

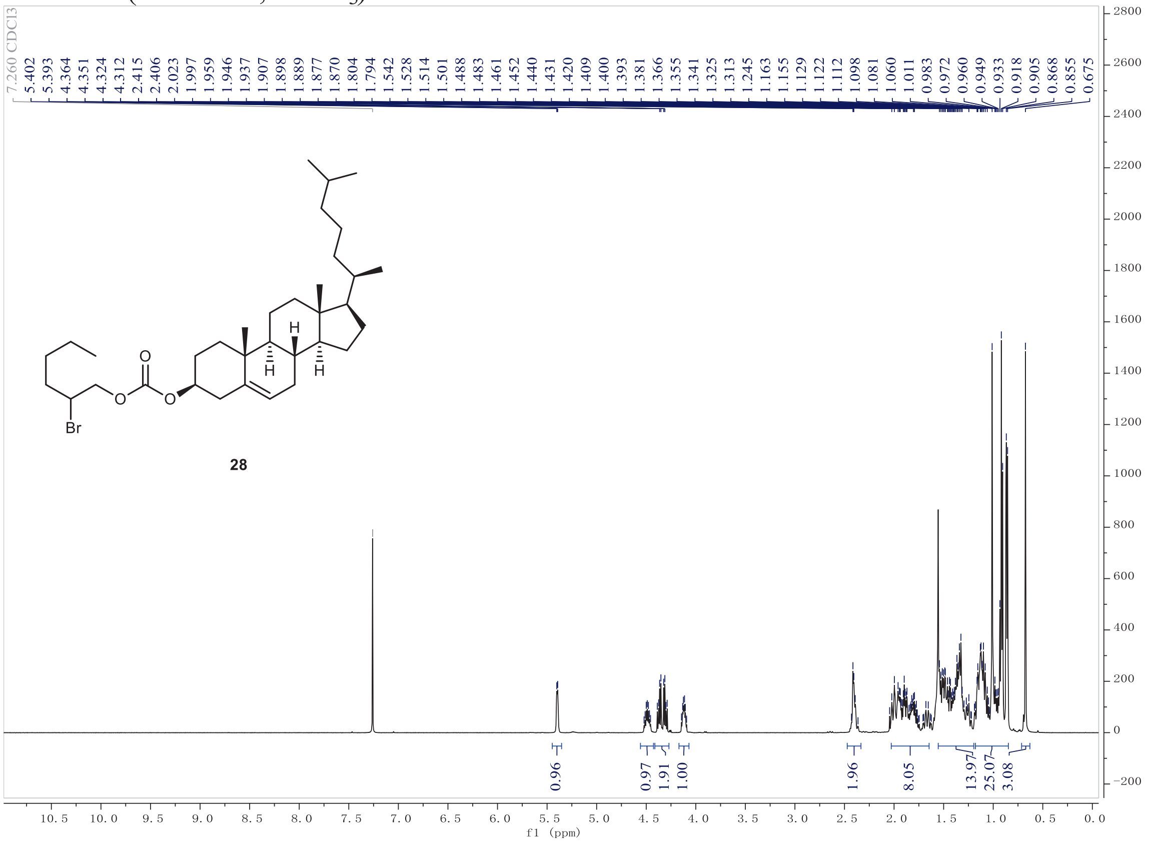

^{13}C NMR (126 MHz, $CDCl_3$)

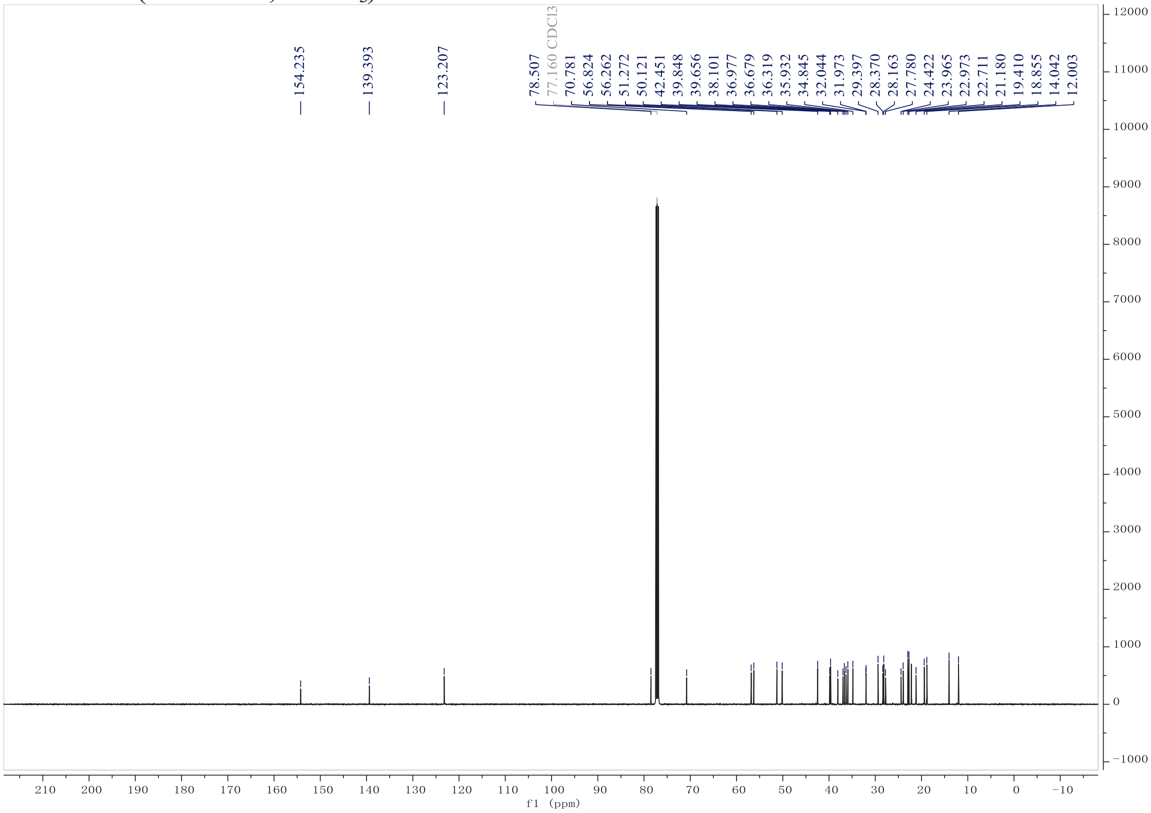

11. References

1. J.-D. Chai, M. Head-Gordon, Systematic optimization of long-range corrected hybrid density functionals, *J. Chem. Phys.* **128**, 084106 (2008).
2. M. Alipour, P. Fallahzadeh, First principles optimally tuned range-separated density functional theory for prediction of phosphorus–hydrogen spin–spin coupling constants. *Phy. Chem. Chem. Phy.* **18**, 18431-18440 (2016).
3. S. Grimme, J. Antony, S. Ehrlich, H. Krieg, A consistent and accurate ab initio parametrization of density functional dispersion correction (DFT-D) for the 94 elements H-Pu. *J. Chem. Phys.* **132**, 154014 (2010).
4. M. J. Frisch, G. W. Trucks, H. B. Schlegel, G. E. Scuseria, M. A. Robb, J. R. Cheeseman, G. Scalmani, V. Barone, G. A. Petersso, H. Nakatsuji, X. Li, M. Caricato, A. V. Marenich, J. Bloino, B. G. Janesko, R. Gomperts, B. Mennucci, H. P. Hratchian, J. V. Ortiz, A. F. Izmaylov, J. L. Sonnenberg, D. Williams-Young, F. Ding, F. Lipparini, F. Egidi, J. Goings, B. Peng, A. Petrone, T. Henderson, D. Ranasinghe, V. G. Zakrzewski, J. Gao, N. Rega, G. Zheng, W. Liang, M. Hada, M. Ehara, K. Toyota, R. Fukuda, J. Hasegawa, M. Ishida, T. Nakajima, Y. Honda, O. Kitao, H. Nakai, T. Vreven, K. Throssell, J. A. Montgomery, J. E. P. Jr., F. Ogliaro, M. J. Bearpark, J. J. Heyd, E. N. Brothers, K. N. Kudin, V. N. Staroverov, T. A. Keith, R. Kobayashi, J. Normand, K. Raghavachari, A. P. Rendell, J. C. Burant, S. S. Iyengar, J. Tomasi, M. Cossi, J. M. Millam, M. Klene, C. Adamo, R. Cammi, J. W. Ochterski, R. L. Martin, K. Morokuma, O. Farkas, J. B. Foresman, D. J. Fox, Gaussian 16, Revision C.02,Gaussian, Inc., Wallingford CT, 2016.
5. A.V. Marenich, C. J. Cramer, D. G. Truhlar, Universal Solvation Model Based on Solute Electron Density and on a Continuum Model of the Solvent Defined by the Bulk Dielectric Constant and Atomic Surface Tensions. *J. Phys. Chem. A.* **113**, 6378-6396 (2009).
6. T. Lu, F. Chen, Multiwfn: A multifunctional wavefunction analyzer, *J. Comput. Chem.* **33**, 580-592 (2012).
7. T. Lu, Q. Chen, Simple, Efficient, and Universal Energy Decomposition Analysis Method Based on Dispersion-Corrected Density Functional Theory. *J. Phys. Chem. A.* **127**, 7023-7035 (2023).
8. J. Klein, H. Khartabil, J.-C. Boisson, J. Contreras-García, J.-P. Piquemal, E. Hénon, New Way for Probing Bond Strength. *J. Phys. Chem. A.* **124**, 1850-1860 (2020).
9. Glendening, E. D.; Landis, C. R.; Weinhold, F., NBO 6.0: Natural bond orbital analysis program. *J. Comput. Chem.* **2013,** *34*, 1429-1437.
10. S. Mirzaei, D. Wang, S. V. Lindeman, C. M. Sem, R. Rathore, Highly Selective Synthesis of Pillar[n]Arene (n = 5, 6). *Org. Lett.* **20**, 6583–6586 (2018).
11. T. Ogoshi, T. Aoki, K. Kitajima, S. Fujinami, T. Yamagishi, Y. Nakamoto, Facile, Rapid, and High-Yield Synthesis of Pillar[5]Arene from Commercially Available Reagents and Its X-Ray Crystal Structure. *J. Org. Chem.* **76**, 328–331 (2011).
12. Thordarson, P. Determining association constants from titration experiments in supramolecular chemistry. *Chem. Soc. Rev.* **40**, 1305–1323 (2011).
13. Hibbert, D. B., Thordarson, P. The death of the Job plot, transparency, open science and online tools, uncertainty estimation methods and other developments in supramolecular chemistry data analysis. *Chem. Commun.* **52**, 12792–12805 (2016).
14. Wang, W., Li, X., Zhou, P.-P., Wang, Y. Catalysis with Supramolecular Carbon-Bonding Interactions. *Angew. Chem. In. Ed.* **60**, 22717–22721 (2021).

15. Heinen, F., Engelage, E., Cramer, C. J., Huber, S. M. Hypervalent Iodine(III) Compounds as Biaxial Halogen Bond Donors. *J. Am. Chem. Soc.* **142**, 8633–8640 (2020).
