# Supplementary material for: Pillar[5]arene-catalyzed anti-Markovnikov halogenations through cationic intermediates stabilization in confined spaces
Source: Nat Commun. 2026 Apr 1;17:4668. doi: 10.1038/s41467-026-71201-9 (PMC13201766; doi:10.1038/s41467-026-71201-9)
Supplement: Supplementary file 2 — Description of Additional Supplementary Files [file 41467_2026_71201_MOESM2_ESM.pdf]

Description of Additional Supplementary Files

File Name: Supplementary Data 1
Description: X-ray crystal data of compound **28** and Cartesian coordinates and energy parameters (A.U.) of all key stationary points.
