# Supplementary material for: Pillar[5]arene-catalyzed anti-Markovnikov halogenations through cationic intermediates stabilization in confined spaces
Source: Nat Commun. 2026 Apr 1;17:4668. doi: 10.1038/s41467-026-71201-9 (PMC13201766; doi:10.1038/s41467-026-71201-9)
Supplement: Supplementary file 3 — Supplementary data 1 [file 41467_2026_71201_MOESM3_ESM.pdf]

Supplementary Data

Pillar[5]arene-Catalyzed Anti-Markovnikov Halogenations Through Cationic Intermediates Stabilization in Confined Spaces

Tianyue Xu[1,†], Shengtian Lai[1,†], Manjaly J. Ajitha[2,*], Kuo-Wei Huang[2,*], Ying-Yeung Yeung[1,*]

[1]State Key Laboratory of Synthetic Chemistry, The Chinese University of Hong Kong, Shatin, NT, Hong Kong (China).

[2]Center of Excellence for Renewable Energy and Storage Technologies, KAUST Catalysis Platform, and Division of Physical Sciences and Engineering, King Abdullah University of Science and Technology, Thuwal 23955-6900, Saudi Arabia.

[†]These authors contributed equally.

*Corresponding authors. Email: ajitha.john@kaust.edu.sa; kuowei.huang@kaust.edu.sa; yyyeung@cuhk.edu.hk

Data S1. X-ray crystal data of compound **28**.

Crystal data and structure refinement for **28**.

Identification code	XTYHEXYLANTImea(LOW)	
Empirical formula	C34 H59 Br O4	
Formula weight	611.72	
Temperature	179(2) K	
Wavelength	1.54178 Å	
Crystal system	Monoclinic	
Space group	$P2_1$	
Unit cell dimensions	a = 12.9323(14) Å	α= 90°.
	b = 8.9478(9) Å	β= 90.206(4)°.
	c = 14.0574(15) Å	γ = 90°.
Volume	1626.7(3) $Å^3$	
Z	2	
Density (calculated)	1.249 Mg/m^3	
Absorption coefficient	1.981 mm^{-1}	
F(000)	660	
Crystal size	0.500 x 0.400 x 0.300 mm^3	
Theta range for data collection	3.144 to 68.537°.	
Index ranges	-15<=h<=14, -10<=k<=10, -16<=l<=16	
Reflections collected	15522	
Independent reflections	5555 [R(int) = 0.0639]	
Completeness to theta = 67.679°	99.1 %	
Absorption correction	multi-scan	
Max. and min. transmission	0.7531 and 0.3552	
Refinement method	Full-matrix least-squares on F^2	
Data / restraints / parameters	5555 / 442 / 495	
Goodness-of-fit on F^2	1.076	
Final R indices [I>2sigma(I)]	R1 = 0.0926, wR2 = 0.2570	
R indices (all data)	R1 = 0.0957, wR2 = 0.2621	
Absolute structure parameter	0.372(14)	
Extinction coefficient	n/a	
Largest diff. peak and hole	0.534 and -0.816 e.$Å^{-3}$	

Atomic coordinates (x 10^4) and equivalent isotropic displacement parameters ($Å^2$x 10^3) for **28**. U(eq) is defined as one third of the trace of the orthogonalized U^{ij} tensor.

	x	y	z	U(eq)
C(1)	-2976(15)	6500(30)	2538(16)	55(5)
C(2)	-2680(30)	4820(30)	1154(16)	61(6)
C(3)	-2106(9)	6112(17)	1835(8)	46(3)
C(1')	-2900(20)	6960(30)	2310(20)	63(7)
C(2')	-2520(30)	5230(40)	980(16)	77(10)
C(3')	-2405(10)	5430(20)	2157(11)	55(4)
C(4)	-1252(5)	5280(10)	2320(5)	52(2)
O(4W)	9059(5)	1817(11)	9032(5)	81(2)
C(5)	-765(5)	5992(8)	3198(5)	48(2)
C(6)	279(5)	5367(9)	3442(4)	46(2)
C(7)	802(4)	6097(7)	4318(4)	36(1)
C(8)	268(5)	5544(11)	5228(5)	55(2)
C(9)	1979(4)	5765(6)	4316(3)	31(1)
C(10)	2515(4)	6385(7)	3414(4)	36(1)
C(11)	3611(4)	6906(6)	3711(4)	36(1)
C(12)	3746(4)	6232(6)	4701(4)	29(1)
C(13)	2644(4)	6382(6)	5145(4)	28(1)
C(14)	2372(4)	8016(6)	5348(4)	36(1)
C(15)	2673(4)	5458(7)	6063(4)	38(1)
C(16)	3530(4)	5961(8)	6744(4)	40(1)
C(17)	4616(4)	5998(6)	6290(4)	30(1)
C(18)	4623(4)	6808(6)	5324(4)	30(1)
C(19)	5668(4)	6546(6)	4831(4)	34(1)
C(20)	6569(4)	6671(6)	5498(4)	36(1)
C(21)	6508(4)	6705(6)	6438(4)	37(1)
C(22)	5472(4)	6592(6)	6976(4)	34(1)
C(23)	5200(5)	8158(7)	7361(5)	44(1)
C(24)	5589(5)	5480(7)	7808(4)	41(1)
C(25)	6555(5)	5771(8)	8431(4)	47(2)
C(27)	7468(5)	6896(7)	7036(5)	44(1)
C(26)	7508(5)	5748(7)	7841(5)	46(1)

O(1)	8402(9)	6238(19)	8415(13)	58(4)
O(2)	8730(5)	3730(9)	8740(6)	59(2)
O(3)	9536(6)	5651(14)	9519(7)	78(3)
C(28)	8866(7)	5020(12)	8861(8)	54(2)
C(29)	10295(8)	4661(19)	9914(11)	78(4)
C(30)	11340(7)	5400(17)	9840(8)	51(3)
Br(1)	11560(1)	5600(3)	8465(1)	87(1)
C(31)	12173(9)	4466(15)	10291(10)	69(3)
C(32)	13273(8)	5014(16)	10176(13)	72(3)
C(33)	13500(9)	6493(18)	10653(11)	67(3)
C(34)	14607(18)	6900(30)	10550(20)	88(8)
O(1')	8337(19)	5980(40)	8560(20)	43(7)
O(2')	9576(17)	4910(30)	7524(14)	73(6)
O(3')	9999(12)	6150(30)	8896(13)	66(5)
C(28')	9323(15)	5670(30)	8181(14)	52(5)
C(29')	11048(17)	5990(40)	8782(16)	63(6)
C(30')	11616(17)	5710(30)	9660(20)	57(7)
Br(1')	11006(4)	4054(5)	10363(4)	97(2)
C(31')	12767(18)	5690(50)	9673(19)	72(6)
C(32')	13250(20)	5670(60)	10680(20)	74(7)
C(33')	14390(20)	5910(50)	10740(30)	87(9)
C(34')	14750(40)	7380(70)	10390(40)	68(10)

Bond lengths [Å] and angles [°] for **28**.

C(1)-C(3)	1.540(19)
C(1)-H(1A)	0.9800
C(1)-H(1B)	0.9800
C(1)-H(1C)	0.9800
C(2)-C(3)	1.68(2)
C(2)-H(2A)	0.9800
C(2)-H(2B)	0.9800
C(2)-H(2C)	0.9800
C(3)-C(4)	1.495(11)
C(3)-H(3)	1.0000
C(1')-C(3')	1.52(2)
C(1')-H(1'A)	0.9800
C(1')-H(1'B)	0.9800
C(1')-H(1'C)	0.9800
C(2')-C(3')	1.67(2)
C(2')-H(2'A)	0.9800
C(2')-H(2'B)	0.9800
C(2')-H(2'C)	0.9800
C(3')-C(4)	1.514(12)
C(3')-H(3')	1.0000
C(4)-C(5)	1.523(8)
C(4)-H(4A)	0.9900
C(4)-H(4B)	0.9900
O(4W)-O(2)	1.812(12)
O(4W)-H(1)	0.88(3)
O(4W)-H(2)	0.88(3)
C(5)-C(6)	1.501(8)
C(5)-H(5A)	0.9900
C(5)-H(5B)	0.9900
C(6)-C(7)	1.547(7)
C(6)-H(6A)	0.9900
C(6)-H(6B)	0.9900
C(7)-C(8)	1.538(9)
C(7)-C(9)	1.550(7)

C(7)-H(7)	1.0000
C(8)-H(8A)	0.9800
C(8)-H(8B)	0.9800
C(8)-H(8C)	0.9800
C(9)-C(13)	1.548(6)
C(9)-C(10)	1.550(7)
C(9)-H(9)	1.0000
C(10)-C(11)	1.549(7)
C(10)-H(10A)	0.9900
C(10)-H(10B)	0.9900
C(11)-C(12)	1.526(8)
C(11)-H(11A)	0.9900
C(11)-H(11B)	0.9900
C(12)-C(18)	1.521(6)
C(12)-C(13)	1.563(7)
C(12)-H(12)	1.0000
C(13)-C(14)	1.530(7)
C(13)-C(15)	1.533(7)
C(14)-H(14A)	0.9800
C(14)-H(14B)	0.9800
C(14)-H(14C)	0.9800
C(15)-C(16)	1.530(7)
C(15)-H(15A)	0.9900
C(15)-H(15B)	0.9900
C(16)-C(17)	1.545(7)
C(16)-H(16A)	0.9900
C(16)-H(16B)	0.9900
C(17)-C(18)	1.539(7)
C(17)-C(22)	1.558(6)
C(17)-H(17)	1.0000
C(18)-C(19)	1.538(7)
C(18)-H(18)	1.0000
C(19)-C(20)	1.498(7)
C(19)-H(19A)	0.9900
C(19)-H(19B)	0.9900
C(20)-C(21)	1.324(9)

C(20)-H(20)	0.9500
C(21)-C(27)	1.507(7)
C(21)-C(22)	1.545(8)
C(22)-C(24)	1.543(8)
C(22)-C(23)	1.543(8)
C(23)-H(23A)	0.9800
C(23)-H(23B)	0.9800
C(23)-H(23C)	0.9800
C(24)-C(25)	1.545(7)
C(24)-H(24A)	0.9900
C(24)-H(24B)	0.9900
C(25)-C(26)	1.488(10)
C(25)-H(25A)	0.9900
C(25)-H(25B)	0.9900
C(27)-C(26)	1.529(10)
C(27)-H(27A)	0.9900
C(27)-H(27B)	0.9900
C(26)-O(1)	1.474(9)
C(26)-O(1')	1.49(2)
C(26)-H(26)	1.0000
O(1)-C(28)	1.392(18)
O(2)-C(28)	1.180(13)
O(3)-C(28)	1.386(11)
O(3)-C(29)	1.432(15)
C(29)-C(30)	1.509(14)
C(29)-H(29A)	0.9900
C(29)-H(29B)	0.9900
C(30)-C(31)	1.502(15)
C(30)-Br(1)	1.963(11)
C(30)-H(30)	1.0000
C(31)-C(32)	1.514(16)
C(31)-H(31A)	0.9900
C(31)-H(31B)	0.9900
C(32)-C(33)	1.51(2)
C(32)-H(32A)	0.9900
C(32)-H(32B)	0.9900

C(33)-C(34)	1.49(2)
C(33)-H(33A)	0.9900
C(33)-H(33B)	0.9900
C(34)-H(34A)	0.9800
C(34)-H(34B)	0.9800
C(34)-H(34C)	0.9800
O(1')-C(28')	1.41(3)
O(2')-C(28')	1.20(2)
O(3')-C(29')	1.37(2)
O(3')-C(28')	1.40(2)
C(29')-C(30')	1.46(2)
C(29')-H(29C)	0.9900
C(29')-H(29D)	0.9900
C(30')-C(31')	1.49(2)
C(30')-Br(1')	1.95(2)
C(30')-H(30')	1.0000
C(31')-C(32')	1.55(3)
C(31')-H(31C)	0.9900
C(31')-H(31D)	0.9900
C(32')-C(33')	1.50(3)
C(32')-H(32C)	0.9900
C(32')-H(32D)	0.9900
C(33')-C(34')	1.48(3)
C(33')-H(33C)	0.9900
C(33')-H(33D)	0.9900
C(34')-H(34D)	0.9800
C(34')-H(34E)	0.9800
C(34')-H(34F)	0.9800

C(3)-C(1)-H(1A)	109.5
C(3)-C(1)-H(1B)	109.5
H(1A)-C(1)-H(1B)	109.5
C(3)-C(1)-H(1C)	109.5
H(1A)-C(1)-H(1C)	109.5
H(1B)-C(1)-H(1C)	109.5
C(3)-C(2)-H(2A)	109.5

C(3)-C(2)-H(2B)	109.5
H(2A)-C(2)-H(2B)	109.5
C(3)-C(2)-H(2C)	109.5
H(2A)-C(2)-H(2C)	109.5
H(2B)-C(2)-H(2C)	109.5
C(4)-C(3)-C(1)	111.1(12)
C(4)-C(3)-C(2)	104.0(14)
C(1)-C(3)-C(2)	101.4(15)
C(4)-C(3)-H(3)	113.1
C(1)-C(3)-H(3)	113.1
C(2)-C(3)-H(3)	113.1
C(3')-C(1')-H(1'A)	109.5
C(3')-C(1')-H(1'B)	109.5
H(1'A)-C(1')-H(1'B)	109.5
C(3')-C(1')-H(1'C)	109.5
H(1'A)-C(1')-H(1'C)	109.5
H(1'B)-C(1')-H(1'C)	109.5
C(3')-C(2')-H(2'A)	109.5
C(3')-C(2')-H(2'B)	109.5
H(2'A)-C(2')-H(2'B)	109.5
C(3')-C(2')-H(2'C)	109.5
H(2'A)-C(2')-H(2'C)	109.5
H(2'B)-C(2')-H(2'C)	109.5
C(4)-C(3')-C(1')	118.2(18)
C(4)-C(3')-C(2')	102.9(16)
C(1')-C(3')-C(2')	101.8(18)
C(4)-C(3')-H(3')	111.0
C(1')-C(3')-H(3')	111.0
C(2')-C(3')-H(3')	111.0
C(3)-C(4)-C(5)	117.6(8)
C(3')-C(4)-C(5)	119.3(8)
C(3)-C(4)-H(4A)	107.9
C(5)-C(4)-H(4A)	107.9
C(3)-C(4)-H(4B)	107.9
C(5)-C(4)-H(4B)	107.9
H(4A)-C(4)-H(4B)	107.2

O(2)-O(4W)-H(1)	113(10)
O(2)-O(4W)-H(2)	106(10)
H(1)-O(4W)-H(2)	103(6)
C(6)-C(5)-C(4)	113.5(6)
C(6)-C(5)-H(5A)	108.9
C(4)-C(5)-H(5A)	108.9
C(6)-C(5)-H(5B)	108.9
C(4)-C(5)-H(5B)	108.9
H(5A)-C(5)-H(5B)	107.7
C(5)-C(6)-C(7)	114.6(5)
C(5)-C(6)-H(6A)	108.6
C(7)-C(6)-H(6A)	108.6
C(5)-C(6)-H(6B)	108.6
C(7)-C(6)-H(6B)	108.6
H(6A)-C(6)-H(6B)	107.6
C(8)-C(7)-C(6)	109.2(5)
C(8)-C(7)-C(9)	112.6(5)
C(6)-C(7)-C(9)	110.1(4)
C(8)-C(7)-H(7)	108.3
C(6)-C(7)-H(7)	108.3
C(9)-C(7)-H(7)	108.3
C(7)-C(8)-H(8A)	109.5
C(7)-C(8)-H(8B)	109.5
H(8A)-C(8)-H(8B)	109.5
C(7)-C(8)-H(8C)	109.5
H(8A)-C(8)-H(8C)	109.5
H(8B)-C(8)-H(8C)	109.5
C(13)-C(9)-C(7)	118.3(4)
C(13)-C(9)-C(10)	103.8(4)
C(7)-C(9)-C(10)	112.0(4)
C(13)-C(9)-H(9)	107.4
C(7)-C(9)-H(9)	107.4
C(10)-C(9)-H(9)	107.4
C(11)-C(10)-C(9)	107.4(4)
C(11)-C(10)-H(10A)	110.2
C(9)-C(10)-H(10A)	110.2

C(11)-C(10)-H(10B)	110.2
C(9)-C(10)-H(10B)	110.2
H(10A)-C(10)-H(10B)	108.5
C(12)-C(11)-C(10)	103.2(4)
C(12)-C(11)-H(11A)	111.1
C(10)-C(11)-H(11A)	111.1
C(12)-C(11)-H(11B)	111.1
C(10)-C(11)-H(11B)	111.1
H(11A)-C(11)-H(11B)	109.1
C(18)-C(12)-C(11)	118.3(4)
C(18)-C(12)-C(13)	114.8(4)
C(11)-C(12)-C(13)	103.2(4)
C(18)-C(12)-H(12)	106.6
C(11)-C(12)-H(12)	106.6
C(13)-C(12)-H(12)	106.6
C(14)-C(13)-C(15)	111.3(5)
C(14)-C(13)-C(9)	110.7(4)
C(15)-C(13)-C(9)	116.9(4)
C(14)-C(13)-C(12)	111.5(4)
C(15)-C(13)-C(12)	105.7(4)
C(9)-C(13)-C(12)	100.0(4)
C(13)-C(14)-H(14A)	109.5
C(13)-C(14)-H(14B)	109.5
H(14A)-C(14)-H(14B)	109.5
C(13)-C(14)-H(14C)	109.5
H(14A)-C(14)-H(14C)	109.5
H(14B)-C(14)-H(14C)	109.5
C(16)-C(15)-C(13)	112.6(5)
C(16)-C(15)-H(15A)	109.1
C(13)-C(15)-H(15A)	109.1
C(16)-C(15)-H(15B)	109.1
C(13)-C(15)-H(15B)	109.1
H(15A)-C(15)-H(15B)	107.8
C(15)-C(16)-C(17)	113.9(5)
C(15)-C(16)-H(16A)	108.8
C(17)-C(16)-H(16A)	108.8

C(15)-C(16)-H(16B)	108.8
C(17)-C(16)-H(16B)	108.8
H(16A)-C(16)-H(16B)	107.7
C(18)-C(17)-C(16)	112.5(4)
C(18)-C(17)-C(22)	112.3(4)
C(16)-C(17)-C(22)	113.3(4)
C(18)-C(17)-H(17)	106.0
C(16)-C(17)-H(17)	106.0
C(22)-C(17)-H(17)	106.0
C(12)-C(18)-C(19)	110.1(4)
C(12)-C(18)-C(17)	110.0(4)
C(19)-C(18)-C(17)	109.5(4)
C(12)-C(18)-H(18)	109.1
C(19)-C(18)-H(18)	109.1
C(17)-C(18)-H(18)	109.1
C(20)-C(19)-C(18)	112.9(4)
C(20)-C(19)-H(19A)	109.0
C(18)-C(19)-H(19A)	109.0
C(20)-C(19)-H(19B)	109.0
C(18)-C(19)-H(19B)	109.0
H(19A)-C(19)-H(19B)	107.8
C(21)-C(20)-C(19)	125.3(5)
C(21)-C(20)-H(20)	117.4
C(19)-C(20)-H(20)	117.4
C(20)-C(21)-C(27)	120.5(5)
C(20)-C(21)-C(22)	122.8(5)
C(27)-C(21)-C(22)	116.7(5)
C(24)-C(22)-C(23)	110.0(5)
C(24)-C(22)-C(21)	109.3(4)
C(23)-C(22)-C(21)	108.1(5)
C(24)-C(22)-C(17)	108.5(4)
C(23)-C(22)-C(17)	111.4(4)
C(21)-C(22)-C(17)	109.5(4)
C(22)-C(23)-H(23A)	109.5
C(22)-C(23)-H(23B)	109.5
H(23A)-C(23)-H(23B)	109.5

C(22)-C(23)-H(23C)	109.5
H(23A)-C(23)-H(23C)	109.5
H(23B)-C(23)-H(23C)	109.5
C(22)-C(24)-C(25)	113.5(5)
C(22)-C(24)-H(24A)	108.9
C(25)-C(24)-H(24A)	108.9
C(22)-C(24)-H(24B)	108.9
C(25)-C(24)-H(24B)	108.9
H(24A)-C(24)-H(24B)	107.7
C(26)-C(25)-C(24)	110.5(5)
C(26)-C(25)-H(25A)	109.5
C(24)-C(25)-H(25A)	109.5
C(26)-C(25)-H(25B)	109.5
C(24)-C(25)-H(25B)	109.5
H(25A)-C(25)-H(25B)	108.1
C(21)-C(27)-C(26)	111.3(5)
C(21)-C(27)-H(27A)	109.4
C(26)-C(27)-H(27A)	109.4
C(21)-C(27)-H(27B)	109.4
C(26)-C(27)-H(27B)	109.4
H(27A)-C(27)-H(27B)	108.0
O(1)-C(26)-C(25)	109.9(9)
O(1')-C(26)-C(25)	102.3(14)
O(1)-C(26)-C(27)	103.3(9)
O(1')-C(26)-C(27)	115.8(19)
C(25)-C(26)-C(27)	112.2(5)
O(1)-C(26)-H(26)	110.4
C(25)-C(26)-H(26)	110.4
C(27)-C(26)-H(26)	110.4
C(28)-O(1)-C(26)	110.5(12)
C(28)-O(2)-O(4W)	149.0(8)
C(28)-O(3)-C(29)	115.6(12)
O(2)-C(28)-O(3)	126.0(10)
O(2)-C(28)-O(1)	129.6(9)
O(3)-C(28)-O(1)	104.4(9)
O(3)-C(29)-C(30)	108.4(11)

O(3)-C(29)-H(29A)	110.0
C(30)-C(29)-H(29A)	110.0
O(3)-C(29)-H(29B)	110.0
C(30)-C(29)-H(29B)	110.0
H(29A)-C(29)-H(29B)	108.4
C(31)-C(30)-C(29)	111.6(11)
C(31)-C(30)-Br(1)	111.1(8)
C(29)-C(30)-Br(1)	104.0(8)
C(31)-C(30)-H(30)	110.0
C(29)-C(30)-H(30)	110.0
Br(1)-C(30)-H(30)	110.0
C(30)-C(31)-C(32)	116.6(10)
C(30)-C(31)-H(31A)	108.2
C(32)-C(31)-H(31A)	108.2
C(30)-C(31)-H(31B)	108.2
C(32)-C(31)-H(31B)	108.2
H(31A)-C(31)-H(31B)	107.3
C(33)-C(32)-C(31)	114.7(12)
C(33)-C(32)-H(32A)	108.6
C(31)-C(32)-H(32A)	108.6
C(33)-C(32)-H(32B)	108.6
C(31)-C(32)-H(32B)	108.6
H(32A)-C(32)-H(32B)	107.6
C(34)-C(33)-C(32)	111.0(16)
C(34)-C(33)-H(33A)	109.4
C(32)-C(33)-H(33A)	109.4
C(34)-C(33)-H(33B)	109.4
C(32)-C(33)-H(33B)	109.4
H(33A)-C(33)-H(33B)	108.0
C(33)-C(34)-H(34A)	109.5
C(33)-C(34)-H(34B)	109.5
H(34A)-C(34)-H(34B)	109.5
C(33)-C(34)-H(34C)	109.5
H(34A)-C(34)-H(34C)	109.5
H(34B)-C(34)-H(34C)	109.5
C(28')-O(1')-C(26)	111(2)

C(29')-O(3')-C(28')	120.0(17)
O(2')-C(28')-O(3')	124.0(19)
O(2')-C(28')-O(1')	131(2)
O(3')-C(28')-O(1')	103.3(16)
O(3')-C(29')-C(30')	114(2)
O(3')-C(29')-H(29C)	108.7
C(30')-C(29')-H(29C)	108.7
O(3')-C(29')-H(29D)	108.7
C(30')-C(29')-H(29D)	108.7
H(29C)-C(29')-H(29D)	107.6
C(29')-C(30')-C(31')	121(2)
C(29')-C(30')-Br(1')	110.8(19)
C(31')-C(30')-Br(1')	113.2(19)
C(29')-C(30')-H(30')	103.3
C(31')-C(30')-H(30')	103.3
Br(1')-C(30')-H(30')	103.3
C(30')-C(31')-C(32')	114(2)
C(30')-C(31')-H(31C)	108.8
C(32')-C(31')-H(31C)	108.8
C(30')-C(31')-H(31D)	108.8
C(32')-C(31')-H(31D)	108.8
H(31C)-C(31')-H(31D)	107.7
C(33')-C(32')-C(31')	116(3)
C(33')-C(32')-H(32C)	108.2
C(31')-C(32')-H(32C)	108.2
C(33')-C(32')-H(32D)	108.2
C(31')-C(32')-H(32D)	108.2
H(32C)-C(32')-H(32D)	107.3
C(34')-C(33')-C(32')	114(3)
C(34')-C(33')-H(33C)	108.7
C(32')-C(33')-H(33C)	108.7
C(34')-C(33')-H(33D)	108.7
C(32')-C(33')-H(33D)	108.7
H(33C)-C(33')-H(33D)	107.6
C(33')-C(34')-H(34D)	109.5
C(33')-C(34')-H(34E)	109.5

H(34D)-C(34')-H(34E)	109.5
C(33')-C(34')-H(34F)	109.5
H(34D)-C(34')-H(34F)	109.5
H(34E)-C(34')-H(34F)	109.5

Symmetry transformations used to generate equivalent atoms:

Anisotropic displacement parameters ($Å^2$x 10^3) for **28**. The anisotropic displacement factor exponent takes the form: $-2\pi^2$[h^2 $a^{*2}U^{11}$ + ... + 2 h k a* b* U^{12}]

	U^{11}	U^{22}	U^{33}	U^{23}	U^{13}	U^{12}
C(1)	31(7)	78(13)	55(11)	-10(9)	-14(6)	10(9)
C(2)	60(12)	75(12)	47(9)	-9(7)	-24(8)	2(8)
C(3)	34(6)	72(8)	31(6)	9(5)	-12(4)	-8(6)
C(1')	68(13)	69(12)	51(12)	-7(9)	-13(9)	2(10)
C(2')	63(14)	120(20)	53(9)	-33(13)	-38(11)	20(15)
C(3')	48(8)	66(9)	50(7)	-6(7)	-26(6)	2(7)
C(4)	33(3)	80(5)	45(3)	-18(3)	-14(2)	-2(3)
O(4W)	57(4)	122(6)	65(4)	7(4)	-9(3)	-2(4)
C(5)	46(3)	51(4)	47(3)	-7(3)	-22(3)	0(3)
C(6)	30(3)	64(4)	45(3)	-18(3)	-11(2)	1(3)
C(7)	26(2)	46(3)	36(3)	-6(2)	-7(2)	-3(2)
C(8)	28(3)	90(5)	48(3)	1(4)	-7(2)	-16(3)
C(9)	27(2)	33(2)	32(2)	-5(2)	-5(2)	-2(2)
C(10)	31(3)	43(3)	33(3)	2(2)	-6(2)	-4(2)
C(11)	28(3)	39(3)	40(3)	4(2)	-5(2)	-4(2)
C(12)	19(2)	32(2)	36(3)	0(2)	-2(2)	-2(2)
C(13)	22(2)	32(2)	31(2)	-2(2)	-2(2)	-4(2)
C(14)	28(3)	33(3)	47(3)	-10(2)	-6(2)	1(2)
C(15)	29(2)	51(3)	34(2)	7(2)	-6(2)	-8(2)
C(16)	31(3)	58(3)	31(2)	3(2)	-6(2)	-4(2)
C(17)	25(2)	32(2)	33(2)	0(2)	-9(2)	-1(2)
C(18)	23(2)	30(2)	37(3)	-1(2)	-5(2)	-1(2)
C(19)	23(2)	37(3)	42(3)	1(2)	-2(2)	0(2)
C(20)	20(2)	37(3)	53(3)	-2(2)	-5(2)	0(2)
C(21)	26(3)	29(2)	56(3)	-2(2)	-9(2)	-2(2)
C(22)	25(2)	34(3)	42(3)	-8(2)	-9(2)	0(2)
C(23)	37(3)	36(3)	57(4)	-14(2)	-15(3)	5(2)
C(24)	37(3)	46(3)	41(3)	-1(2)	-13(2)	2(2)
C(25)	51(3)	44(3)	45(3)	-6(3)	-23(3)	3(3)
C(27)	29(3)	43(3)	62(4)	-6(3)	-17(3)	0(2)
C(26)	40(3)	39(3)	59(3)	-9(3)	-28(3)	2(2)

O(1)	42(5)	50(7)	82(8)	1(6)	-44(5)	-4(4)
O(2)	39(4)	59(4)	79(5)	16(3)	-16(3)	2(3)
O(3)	32(3)	118(7)	84(6)	-10(6)	-35(4)	0(4)
C(28)	31(4)	59(5)	70(6)	8(4)	-28(4)	-7(4)
C(29)	35(5)	110(9)	90(8)	37(8)	-21(5)	-8(6)
C(30)	22(4)	80(9)	53(5)	-11(5)	1(3)	2(4)
Br(1)	86(1)	116(1)	59(1)	17(1)	14(1)	3(1)
C(31)	46(5)	67(6)	92(8)	21(6)	-10(5)	1(5)
C(32)	36(5)	70(7)	111(11)	10(7)	-12(6)	6(5)
C(33)	35(5)	85(8)	82(8)	6(7)	-7(5)	-2(5)
C(34)	49(8)	93(16)	120(20)	-36(14)	-5(10)	-11(9)
O(1')	45(10)	31(13)	51(11)	4(10)	-15(8)	8(9)
O(2')	76(12)	91(15)	50(10)	-27(9)	-18(8)	26(11)
O(3')	34(7)	121(15)	44(8)	-30(9)	-13(6)	22(9)
C(28')	56(10)	52(11)	47(10)	-7(9)	-6(8)	8(10)
C(29')	60(10)	93(16)	35(10)	-15(9)	2(9)	30(13)
C(30')	54(11)	40(11)	78(16)	11(10)	-23(12)	7(11)
Br(1')	89(3)	87(3)	113(4)	31(2)	26(2)	3(2)
C(31')	56(10)	90(16)	69(12)	11(13)	-8(9)	13(11)
C(32')	70(13)	90(19)	60(13)	12(16)	-22(12)	-2(15)
C(33')	67(14)	90(20)	100(20)	-2(18)	-22(14)	11(16)
C(34')	50(20)	100(30)	45(15)	10(20)	-9(15)	16(15)

Hydrogen coordinates (x 10^4) and isotropic displacement parameters (Å^2 x 10^3) for **28**.

	x	y	z	U(eq)
H(1A)	-3588	6834	2184	82
H(1B)	-2743	7301	2963	82
H(1C)	-3151	5614	2914	82
H(2A)	-3127	5309	683	91
H(2B)	-3103	4159	1555	91
H(2C)	-2155	4223	827	91
H(3)	-1857	6995	1465	55
H(1'A)	-2809	7569	1738	94
H(1'B)	-2564	7453	2853	94
H(1'C)	-3636	6833	2441	94
H(2'A)	-2644	6204	685	116
H(2'B)	-3102	4560	838	116
H(2'C)	-1882	4796	725	116
H(3')	-2795	4636	2502	66
H(4A)	-697	5110	1849	63
H(4B)	-1522	4286	2505	63
H(1)	8520(80)	1270(140)	9170(100)	122
H(2)	9280(100)	1400(160)	8500(70)	122
H(5A)	-699	7081	3090	58
H(5B)	-1232	5845	3746	58
H(6A)	739	5492	2886	56
H(6B)	208	4282	3562	56
H(7)	705	7203	4274	43
H(8A)	-478	5725	5179	83
H(8B)	547	6083	5779	83
H(8C)	394	4471	5306	83
H(9)	2064	4654	4311	37
H(10A)	2560	5598	2921	43
H(10B)	2115	7235	3151	43
H(11A)	4141	6521	3267	43
H(11B)	3655	8010	3732	43

H(12)	3872	5139	4609	34
H(14A)	2331	8569	4748	54
H(14B)	2908	8459	5755	54
H(14C)	1704	8065	5673	54
H(15A)	1998	5545	6388	46
H(15B)	2780	4393	5900	46
H(16A)	3546	5275	7297	48
H(16B)	3362	6972	6984	48
H(17)	4800	4935	6153	36
H(18)	4527	7904	5432	36
H(19A)	5667	5538	4541	41
H(19B)	5749	7286	4313	41
H(20)	7239	6731	5225	44
H(23A)	5057	8833	6827	65
H(23B)	5783	8546	7733	65
H(23C)	4587	8090	7767	65
H(24A)	5629	4453	7548	49
H(24B)	4966	5538	8213	49
H(25A)	6490	6754	8748	56
H(25B)	6605	4994	8931	56
H(27A)	7480	7916	7309	53
H(27B)	8087	6780	6630	53
H(26)	7630	4724	7580	55
H(29A)	10295	3704	9561	94
H(29B)	10133	4450	10588	94
H(30)	11320	6410	10143	62
H(31A)	12128	3445	10022	82
H(31B)	12025	4389	10980	82
H(32A)	13748	4250	10439	87
H(32B)	13422	5110	9488	87
H(33A)	13062	7281	10366	81
H(33B)	13329	6425	11337	81
H(34A)	15040	6028	10676	133
H(34B)	14780	7702	10996	133
H(34C)	14730	7252	9895	133
H(29C)	11324	6912	8485	75

H(29D)	11177	5154	8339	75
H(30')	11444	6602	10065	69
H(31C)	13023	6585	9330	86
H(31D)	13008	4799	9323	86
H(32C)	12905	6462	11064	89
H(32D)	13081	4702	10981	89
H(33C)	14612	5795	11416	104
H(33D)	14741	5114	10373	104
H(34D)	15492	7482	10515	102
H(34E)	14375	8172	10726	102
H(34F)	14616	7450	9709	102

Torsion angles [°] for **28**.

C(1)-C(3)-C(4)-C(5)	-54.4(16)
C(2)-C(3)-C(4)-C(5)	-162.8(13)
C(1')-C(3')-C(4)-C(5)	44(2)
C(2')-C(3')-C(4)-C(5)	155.5(16)
C(3)-C(4)-C(5)-C(6)	-161.0(9)
C(3')-C(4)-C(5)-C(6)	161.5(11)
C(4)-C(5)-C(6)-C(7)	178.7(6)
C(5)-C(6)-C(7)-C(8)	74.2(8)
C(5)-C(6)-C(7)-C(9)	-161.7(6)
C(8)-C(7)-C(9)-C(13)	-57.0(7)
C(6)-C(7)-C(9)-C(13)	-179.2(5)
C(8)-C(7)-C(9)-C(10)	-177.7(5)
C(6)-C(7)-C(9)-C(10)	60.1(6)
C(13)-C(9)-C(10)-C(11)	16.6(6)
C(7)-C(9)-C(10)-C(11)	145.4(5)
C(9)-C(10)-C(11)-C(12)	12.8(6)
C(10)-C(11)-C(12)-C(18)	-165.4(4)
C(10)-C(11)-C(12)-C(13)	-37.3(5)
C(7)-C(9)-C(13)-C(14)	-45.6(6)
C(10)-C(9)-C(13)-C(14)	79.2(5)
C(7)-C(9)-C(13)-C(15)	83.3(6)
C(10)-C(9)-C(13)-C(15)	-151.9(5)
C(7)-C(9)-C(13)-C(12)	-163.3(5)
C(10)-C(9)-C(13)-C(12)	-38.5(5)
C(18)-C(12)-C(13)-C(14)	60.7(6)
C(11)-C(12)-C(13)-C(14)	-69.5(5)
C(18)-C(12)-C(13)-C(15)	-60.4(5)
C(11)-C(12)-C(13)-C(15)	169.4(4)
C(18)-C(12)-C(13)-C(9)	177.8(4)
C(11)-C(12)-C(13)-C(9)	47.6(5)
C(14)-C(13)-C(15)-C(16)	-64.5(6)
C(9)-C(13)-C(15)-C(16)	166.9(5)
C(12)-C(13)-C(15)-C(16)	56.7(6)
C(13)-C(15)-C(16)-C(17)	-54.0(7)

C(15)-C(16)-C(17)-C(18)	48.2(7)
C(15)-C(16)-C(17)-C(22)	177.0(5)
C(11)-C(12)-C(18)-C(19)	-59.8(6)
C(13)-C(12)-C(18)-C(19)	177.8(4)
C(11)-C(12)-C(18)-C(17)	179.5(5)
C(13)-C(12)-C(18)-C(17)	57.1(6)
C(16)-C(17)-C(18)-C(12)	-48.3(6)
C(22)-C(17)-C(18)-C(12)	-177.6(4)
C(16)-C(17)-C(18)-C(19)	-169.4(4)
C(22)-C(17)-C(18)-C(19)	61.3(6)
C(12)-C(18)-C(19)-C(20)	-163.6(4)
C(17)-C(18)-C(19)-C(20)	-42.5(6)
C(18)-C(19)-C(20)-C(21)	12.8(8)
C(19)-C(20)-C(21)-C(27)	-177.5(5)
C(19)-C(20)-C(21)-C(22)	1.0(9)
C(20)-C(21)-C(22)-C(24)	134.6(6)
C(27)-C(21)-C(22)-C(24)	-46.9(6)
C(20)-C(21)-C(22)-C(23)	-105.7(6)
C(27)-C(21)-C(22)-C(23)	72.9(6)
C(20)-C(21)-C(22)-C(17)	15.8(7)
C(27)-C(21)-C(22)-C(17)	-165.6(5)
C(18)-C(17)-C(22)-C(24)	-166.0(4)
C(16)-C(17)-C(22)-C(24)	65.2(6)
C(18)-C(17)-C(22)-C(23)	72.8(6)
C(16)-C(17)-C(22)-C(23)	-56.0(6)
C(18)-C(17)-C(22)-C(21)	-46.8(6)
C(16)-C(17)-C(22)-C(21)	-175.6(5)
C(23)-C(22)-C(24)-C(25)	-69.4(6)
C(21)-C(22)-C(24)-C(25)	49.2(6)
C(17)-C(22)-C(24)-C(25)	168.6(5)
C(22)-C(24)-C(25)-C(26)	-56.5(7)
C(20)-C(21)-C(27)-C(26)	-132.0(6)
C(22)-C(21)-C(27)-C(26)	49.4(7)
C(24)-C(25)-C(26)-O(1)	171.9(9)
C(24)-C(25)-C(26)-O(1')	-177.7(17)
C(24)-C(25)-C(26)-C(27)	57.6(7)

C(21)-C(27)-C(26)-O(1)	-172.3(8)
C(21)-C(27)-C(26)-O(1')	-171.0(12)
C(21)-C(27)-C(26)-C(25)	-54.0(7)
C(25)-C(26)-O(1)-C(28)	89.8(13)
C(27)-C(26)-O(1)-C(28)	-150.3(12)
O(4W)-O(2)-C(28)-O(3)	-3(3)
O(4W)-O(2)-C(28)-O(1)	178.1(14)
C(29)-O(3)-C(28)-O(2)	16.0(19)
C(29)-O(3)-C(28)-O(1)	-165.2(13)
C(26)-O(1)-C(28)-O(2)	11(2)
C(26)-O(1)-C(28)-O(3)	-167.8(12)
C(28)-O(3)-C(29)-C(30)	130.3(12)
O(3)-C(29)-C(30)-C(31)	176.7(12)
O(3)-C(29)-C(30)-Br(1)	-63.4(14)
C(29)-C(30)-C(31)-C(32)	174.1(14)
Br(1)-C(30)-C(31)-C(32)	58.5(16)
C(30)-C(31)-C(32)-C(33)	64.6(18)
C(31)-C(32)-C(33)-C(34)	177.1(18)
C(25)-C(26)-O(1')-C(28')	167(3)
C(27)-C(26)-O(1')-C(28')	-71(3)
C(29')-O(3')-C(28')-O(2')	12(5)
C(29')-O(3')-C(28')-O(1')	178(3)
C(26)-O(1')-C(28')-O(2')	-24(5)
C(26)-O(1')-C(28')-O(3')	171(3)
C(28')-O(3')-C(29')-C(30')	-147(3)
O(3')-C(29')-C(30')-C(31')	-173(3)
O(3')-C(29')-C(30')-Br(1')	52(3)
C(29')-C(30')-C(31')-C(32')	169(4)
Br(1')-C(30')-C(31')-C(32')	-56(4)
C(30')-C(31')-C(32')-C(33')	-170(4)
C(31')-C(32')-C(33')-C(34')	63(6)

Symmetry transformations used to generate equivalent atoms:

Data S2. Cartesian coordinates and energy parameters (A.U.) of all key stationary points. E_1 is the optimized energy and E_2 is the single point energy at ωB97XD/6-311+G(d,p) level.

1a

E_1 = -719.621570062 A.U.

Zero-point correction= 0.276066 (Hartree/Particle)

Thermal correction to Energy= 0.292583

Thermal correction to Enthalpy= 0.293527

Thermal correction to Gibbs Free Energy= 0.230248

E_2 = -719.772564996 A.U.

At No.	X	Y	Z
6	3.707252000	0.631966000	0.146893000
1	3.645925000	1.406749000	0.924597000
6	4.956148000	-0.178007000	0.345420000
1	5.036167000	-0.700153000	1.300125000
1	3.753334000	1.155435000	-0.816416000
6	5.931651000	-0.311529000	-0.548585000
1	5.890254000	0.189142000	-1.513780000
1	6.808988000	-0.920794000	-0.351478000
6	-0.097821000	-0.249217000	0.098998000
1	-0.063665000	-1.011520000	-0.695628000
1	-0.141292000	-0.783563000	1.061616000
6	1.161219000	0.601564000	0.044227000
1	1.176913000	1.137327000	-0.913012000
1	1.100779000	1.363187000	0.831700000
6	2.435786000	-0.224986000	0.206533000
1	2.407101000	-0.763662000	1.164368000
1	2.487589000	-0.992133000	-0.577463000
8	-1.229979000	0.579119000	-0.058704000
14	-2.791448000	-0.017458000	-0.043395000
6	-3.021972000	-1.265121000	-1.432064000
1	-2.372902000	-2.139619000	-1.308991000
1	-4.056603000	-1.626823000	-1.460482000
1	-2.797679000	-0.815524000	-2.405648000
6	-3.895783000	1.471497000	-0.298389000
1	-4.952863000	1.181668000	-0.301723000
1	-3.750598000	2.208297000	0.498604000
1	-3.677629000	1.962473000	-1.252592000
6	-3.154131000	-0.841865000	1.608051000
1	-2.505599000	-1.707438000	1.785134000
1	-3.011355000	-0.140490000	2.437483000
1	-4.190841000	-1.196919000	1.644239000

BzOH

E_1 = -420.682695907 A.U.

Zero-point correction= 0.117514 (Hartree/Particle)

Thermal correction to Energy= 0.124543

Thermal correction to Enthalpy= 0.125487

Thermal correction to Gibbs Free Energy= 0.085525

E_2 = -420.808286952 A.U.

At No.	X	Y	Z
6	-0.217573000	0.026937000	-0.000002000
6	0.509754000	1.218848000	0.000049000
6	0.448900000	-1.200536000	0.000003000
6	1.898204000	1.184174000	0.000007000
6	1.838813000	-1.230664000	-0.000010000
6	2.562795000	-0.040604000	-0.000016000
1	-0.030071000	2.159963000	0.000061000

1	-0.121443000	-2.122707000	0.000001000
1	2.463386000	2.111018000	-0.000019000
1	2.358259000	-2.183953000	-0.000014000
1	3.648536000	-0.067844000	-0.000032000
6	-1.701176000	0.119199000	-0.000013000
8	-2.330203000	1.153224000	-0.000039000
8	-2.306972000	-1.085452000	0.000018000
1	-3.259571000	-0.902778000	0.000065000

DBDMH

E_1 = -5597.38830549 A.U.

Zero-point correction= 0.117014 (Hartree/Particle)

Thermal correction to Energy= 0.128621

Thermal correction to Enthalpy= 0.129565

Thermal correction to Gibbs Free Energy= 0.077248

E_2 = -5602.41343084 A.U.

At No.	X	Y	Z
7	-1.179363000	0.011175000	-0.005329000
6	-0.101284000	-0.910527000	-0.064739000
6	-0.787278000	1.334346000	-0.016512000
6	0.751965000	1.319616000	-0.008694000
7	1.009922000	-0.111178000	-0.194573000
8	-0.183402000	-2.106672000	-0.028998000
8	-1.492505000	2.308692000	-0.009301000
35	2.691281000	-0.832588000	0.004772000
35	-2.927345000	-0.542173000	0.007929000
6	1.305581000	2.142269000	-1.167574000
1	2.399076000	2.098135000	-1.163194000
1	0.943607000	1.756291000	-2.124104000
1	0.991253000	3.183487000	-1.063067000
6	1.238406000	1.829022000	1.351175000
1	0.848750000	1.208040000	2.163443000
1	2.331405000	1.809357000	1.389001000
1	0.897150000	2.856851000	1.497150000

PA1

E_2 = -2496.29409764 A.U.

Zero-point correction= 0.887478 (Hartree/Particle)

Thermal correction to Energy= 0.940335

Thermal correction to Enthalpy= 0.941279

Thermal correction to Gibbs Free Energy= 0.796688

E_2 = -2496.93947776 A.U.

At No.	X	Y	Z
6	4.294561000	0.320874000	-0.008845000
6	4.101183000	-0.368663000	-1.209570000
6	4.113283000	-0.365225000	1.189688000
6	3.756813000	-1.718078000	-1.192315000
6	3.745658000	-1.708503000	1.206962000
6	3.573801000	-2.403747000	0.006162000
1	4.240717000	0.194109000	2.109093000
1	3.592388000	-2.267477000	-2.111789000
6	4.636848000	1.796055000	-0.002475000
1	5.242107000	2.021177000	0.879547000
1	5.234070000	2.036711000	-0.885840000
6	3.390171000	2.655797000	0.005225000
6	2.792399000	3.039782000	-1.192794000
6	2.784370000	3.035989000	1.206480000
6	1.615620000	3.784608000	-1.209298000
6	1.620744000	3.801219000	1.189868000
6	1.021993000	4.183862000	-0.008048000
1	3.262595000	2.711600000	-2.112365000

1	1.129803000	4.097069000	2.109506000
6	-0.275319000	4.965264000	-0.000015000
1	-0.320446000	5.608355000	-0.882814000
1	-0.301643000	5.609682000	0.882624000
6	-1.478288000	4.045294000	0.008573000
6	-2.032587000	3.599665000	-1.188975000
6	-2.022603000	3.582092000	1.210172000
6	-3.104599000	2.710630000	-1.204885000
6	-3.109667000	2.711562000	1.194375000
6	-3.663106000	2.264859000	-0.003190000
1	-1.578702000	3.948983000	-2.108888000
1	-3.539440000	2.332756000	2.114286000
6	-4.807333000	1.272781000	0.005386000
1	-5.434441000	1.430177000	-0.876028000
1	-5.426787000	1.445915000	0.889269000
6	-4.304536000	-0.155741000	0.010769000
6	-4.034694000	-0.820608000	1.210685000
6	-4.049888000	-0.816721000	-1.188502000
6	-3.542824000	-2.123529000	1.191501000
6	-3.535546000	-2.110955000	-1.207681000
6	-3.286225000	-2.783782000	-0.007698000
1	-4.240322000	-0.274228000	-2.107059000
1	-3.317241000	-2.652457000	2.110011000
6	-2.695340000	-4.178189000	-0.002666000
1	-3.052002000	-4.716947000	0.879092000
1	-3.037114000	-4.723247000	-0.886501000
6	-1.181439000	-4.140713000	0.005021000
6	-0.467917000	-4.091115000	1.206366000
6	-0.471902000	-4.101383000	-1.192868000
6	0.923255000	-4.026502000	1.190069000
6	0.917997000	-4.012676000	-1.209158000
6	1.632712000	-3.985373000	-0.007859000
1	-1.044758000	-4.113330000	-2.112667000
1	1.493935000	-3.976732000	2.109953000
6	3.141573000	-3.855159000	-0.000160000
1	3.541595000	-4.359455000	0.883391000
1	3.555768000	-4.350342000	-0.882347000
8	-3.671386000	2.228007000	-2.355361000
6	-3.082434000	2.593404000	-3.578211000
1	-2.030142000	2.282286000	-3.628604000
1	-3.141900000	3.676179000	-3.751750000
8	0.978304000	4.168731000	-2.359960000
6	1.503447000	3.713689000	-3.581798000
1	1.533791000	2.616498000	-3.624670000
1	2.513644000	4.104827000	-3.762127000
8	4.272472000	0.354383000	-2.360922000
6	4.006200000	-0.288305000	-3.582303000
1	4.690444000	-1.129073000	-3.758068000
1	2.972022000	-0.655659000	-3.628406000
8	1.659539000	-3.951098000	-2.359863000
6	0.967523000	-3.894040000	-3.581978000
1	0.299560000	-3.023164000	-3.627633000
1	1.730121000	-3.806557000	-4.358256000
8	-3.249951000	-2.795621000	-2.359868000
6	-3.412012000	-2.118358000	-3.580870000
1	-4.459598000	-1.839199000	-3.756054000
1	-2.790390000	-1.213887000	-3.626686000
8	-4.283552000	-0.121771000	2.362850000
6	-3.947207000	-0.731752000	3.583750000
1	-4.534263000	-1.643072000	3.759332000
1	-2.878726000	-0.982565000	3.629153000
8	-1.211421000	-4.113034000	2.357207000

6	-0.528982000	-3.982200000	3.579040000
1	0.156623000	-4.821882000	3.754827000
1	0.039135000	-3.043206000	3.625915000
8	3.535818000	-2.421722000	2.358140000
6	3.621714000	-1.732033000	3.579904000
1	4.632221000	-1.339980000	3.756489000
1	2.904597000	-0.901230000	3.625936000
8	3.399783000	2.617894000	2.357297000
6	2.772947000	2.915062000	3.579827000
1	2.713319000	3.997581000	3.754718000
1	1.761015000	2.490463000	3.628481000
8	-1.430596000	4.033991000	2.360366000
6	-1.899972000	3.522255000	3.582495000
1	-2.947741000	3.796674000	3.763758000
1	-1.806085000	2.428626000	3.625020000
1	-3.650226000	2.076972000	-4.354559000
1	0.833157000	4.087949000	-4.357985000
1	4.158766000	0.462842000	-4.359680000
1	0.378645000	-4.803758000	-3.759822000
1	-3.094674000	-2.815520000	-4.358744000
1	3.382124000	-2.460653000	4.356770000
1	3.393100000	2.463006000	4.356280000
1	-1.275710000	3.969780000	4.358292000
1	-4.181414000	-0.002464000	4.361638000
1	-1.295941000	-3.980570000	4.355952000

PTS^{PA1}

E_2 = -9234.11326689 A.U.

Zero-point correction= 1.407223 (Hartree/Particle)

Thermal correction to Energy= 1.497907

Thermal correction to Enthalpy= 1.498852

Thermal correction to Gibbs Free Energy= 1.275975

E_2 = -9240.01069055 A.U.

At No.	X	Y	Z
6	-1.170985000	2.751307000	-3.001556000
6	0.168682000	2.637488000	-2.612228000
6	-2.083572000	3.281732000	-2.093827000
6	0.564572000	3.029330000	-1.333662000
6	-1.687787000	3.682222000	-0.817077000
6	-0.351408000	3.551361000	-0.420778000
1	-3.121873000	3.345820000	-2.399829000
1	1.598169000	2.934233000	-1.017209000
6	-1.619138000	2.245705000	-4.359725000
1	-2.540451000	2.755652000	-4.650808000
1	-0.855904000	2.480403000	-5.106941000
6	-1.849681000	0.747089000	-4.335638000
6	-0.784852000	-0.135963000	-4.502526000
6	-3.110571000	0.216820000	-4.039711000
6	-0.945843000	-1.508194000	-4.338343000
6	-3.287399000	-1.163193000	-3.958081000
6	-2.211795000	-2.041261000	-4.085438000
1	0.192469000	0.286941000	-4.696286000
1	-4.257075000	-1.590448000	-3.729910000
6	-2.399434000	-3.529351000	-3.872070000
1	-1.661149000	-4.076925000	-4.462915000
1	-3.390929000	-3.820179000	-4.228933000
6	-2.257796000	-3.928685000	-2.415505000
6	-1.008377000	-4.238692000	-1.876708000
6	-3.370752000	-3.964115000	-1.571143000
6	-0.862928000	-4.590221000	-0.534946000

6	-3.233640000	-4.366368000	-0.245048000
6	-1.989819000	-4.686500000	0.290921000
1	-0.147816000	-4.172429000	-2.532958000
1	-4.089384000	-4.408912000	0.417577000
6	-1.861914000	-5.097722000	1.744877000
1	-1.069809000	-5.844828000	1.842915000
1	-2.798972000	-5.562989000	2.064072000
6	-1.548411000	-3.920979000	2.643968000
6	-2.564969000	-3.071761000	3.095465000
6	-0.232586000	-3.622823000	2.987960000
6	-2.251353000	-1.950481000	3.858096000
6	0.084921000	-2.483420000	3.724744000
6	-0.931036000	-1.633212000	4.171660000
1	0.547373000	-4.269979000	2.604137000
1	-3.021916000	-1.263271000	4.187092000
6	-0.611071000	-0.337503000	4.890304000
1	-1.425326000	-0.097197000	5.579383000
1	0.301633000	-0.457273000	5.479277000
6	-0.427561000	0.802341000	3.908253000
6	-1.517374000	1.557915000	3.462997000
6	0.830505000	1.079429000	3.378163000
6	-1.324601000	2.576250000	2.533040000
6	1.019569000	2.070175000	2.413112000
6	-0.066950000	2.847852000	1.994606000
1	1.658731000	0.467918000	3.717012000
1	-2.155284000	3.175553000	2.179899000
6	0.101156000	3.951841000	0.969101000
1	-0.469351000	4.827082000	1.293791000
1	1.151670000	4.243433000	0.929122000
8	0.337564000	-4.869812000	0.053724000
6	1.516025000	-4.580863000	-0.665905000
1	1.563584000	-3.520906000	-0.941523000
1	1.603014000	-5.201682000	-1.566999000
8	0.096788000	-2.396412000	-4.402006000
6	1.381892000	-1.912524000	-4.081048000
1	1.393333000	-1.405008000	-3.107156000
1	1.771088000	-1.230238000	-4.847617000
8	1.023681000	2.139755000	-3.550393000
6	2.319631000	1.784483000	-3.122278000
1	2.917551000	2.666927000	-2.874454000
1	2.284506000	1.126794000	-2.248920000
8	2.229606000	2.334669000	1.836248000
6	3.335503000	1.566857000	2.255196000
1	3.188216000	0.503247000	2.026968000
1	4.191198000	1.950360000	1.699606000
8	1.369049000	-2.104065000	4.008188000
6	2.416174000	-2.722964000	3.288632000
1	2.510277000	-3.787731000	3.534405000
1	2.288057000	-2.610719000	2.205425000
8	-3.844603000	-3.407140000	2.735388000
6	-4.898522000	-2.661440000	3.291522000
1	-4.885410000	-2.699299000	4.388742000
1	-4.870422000	-1.610450000	2.975086000
8	-2.738908000	1.244287000	3.995205000
6	-3.831722000	2.069530000	3.660789000
1	-3.652040000	3.111354000	3.958405000
1	-4.066025000	2.037503000	2.589627000
8	-2.544406000	4.215448000	0.106535000
6	-3.894957000	4.390951000	-0.260093000
1	-3.989026000	5.057328000	-1.128050000
1	-4.386352000	3.433497000	-0.464035000
8	-4.113767000	1.123211000	-3.817951000

6	-5.364099000	0.630055000	-3.403857000
1	-5.838369000	0.018107000	-4.182590000
1	-5.278911000	0.035771000	-2.484174000
8	-4.566935000	-3.575745000	-2.119073000
6	-5.657917000	-3.410963000	-1.247192000
1	-5.993181000	-4.367868000	-0.825740000
1	-5.412086000	-2.726008000	-0.423670000
6	-1.351875000	-0.618841000	-1.059828000
1	-1.329869000	0.114148000	-1.868956000
6	-0.430857000	-0.215639000	0.047502000
1	-0.254953000	0.844426000	0.218933000
1	-1.036986000	-1.583790000	-1.467915000
6	0.072831000	-1.115782000	0.905721000
1	-0.134433000	-2.177246000	0.788745000
1	0.603724000	-0.805410000	1.796562000
6	-4.795959000	0.345286000	0.453703000
1	-5.401485000	-0.321161000	-0.184150000
1	-4.663423000	-0.164844000	1.419605000
6	-3.441899000	0.567539000	-0.186733000
1	-3.563514000	1.163192000	-1.099357000
1	-2.813887000	1.154926000	0.495595000
6	-2.786807000	-0.765318000	-0.532710000
1	-2.773311000	-1.426148000	0.345014000
1	-3.384651000	-1.269994000	-1.299839000
8	-5.462656000	1.578677000	0.652347000
14	-7.086609000	1.626982000	1.048176000
6	-8.111542000	1.139029000	-0.451087000
1	-7.846144000	0.137295000	-0.809391000
1	-9.182378000	1.130777000	-0.217089000
1	-7.954302000	1.842042000	-1.276567000
6	-7.410777000	3.400752000	1.548196000
1	-6.793816000	3.680105000	2.409354000
1	-7.170536000	4.086112000	0.728005000
1	-8.460534000	3.557860000	1.820006000
6	-7.426426000	0.434133000	2.462744000
1	-6.794307000	0.657452000	3.329594000
1	-8.471699000	0.492040000	2.787108000
1	-7.230649000	-0.602903000	2.165608000
7	6.324014000	-1.897714000	-0.228082000
6	4.994995000	-2.152591000	0.039426000
6	6.556985000	-0.516659000	-0.670470000
6	5.122527000	-0.110416000	-1.029066000
7	4.291975000	-1.086102000	-0.564250000
8	4.510646000	-3.086011000	0.625967000
35	2.410844000	-0.908371000	-0.289798000
35	7.621836000	-2.835033000	0.688202000
1	5.018314000	2.735780000	-1.622101000
8	5.439379000	3.396611000	0.537168000
6	4.680169000	3.955221000	-0.233202000
8	4.579823000	3.605385000	-1.517928000
6	3.769118000	5.064686000	0.149414000
6	2.918514000	5.668680000	-0.779647000
6	3.742613000	5.465098000	1.486589000
6	2.051167000	6.674894000	-0.370201000
1	2.927092000	5.339581000	-1.812468000
1	4.400257000	4.970894000	2.193440000
1	1.382080000	7.135241000	-1.089998000
1	1.343715000	7.854591000	1.283050000
6	2.028835000	7.074836000	0.963556000
6	2.873080000	6.468772000	1.892302000
1	2.846183000	6.773867000	2.933658000
8	4.801931000	0.908115000	-1.607284000

6	7.030934000	0.391915000	0.471054000
1	8.064120000	0.149945000	0.735836000
1	6.966166000	1.440722000	0.171447000
1	6.402376000	0.257452000	1.357108000
6	7.476047000	-0.461458000	-1.883469000
1	7.101027000	-1.101966000	-2.686048000
1	7.543161000	0.566155000	-2.248940000
1	8.477703000	-0.801108000	-1.603095000
1	2.346673000	-4.798073000	0.006534000
1	2.030410000	-2.790320000	-4.034918000
1	2.784475000	1.255173000	-3.956689000
1	3.521564000	1.682889000	3.330963000
1	3.331626000	-2.211400000	3.590782000
1	-4.378781000	4.854181000	0.602088000
1	-5.985077000	1.505303000	-3.205648000
1	-6.464694000	-2.985058000	-1.847330000
1	-5.820325000	-3.118743000	2.926118000
1	-4.682351000	1.680735000	4.225287000

TS1^{PA1} (Imag. Freq → -72.0591 cm^{-1})
E_2 = -9234.10003411 A.U.
Zero-point correction= 1.407604 (Hartree/Particle)
Thermal correction to Energy= 1.497041
Thermal correction to Enthalpy= 1.497985
Thermal correction to Gibbs Free Energy= 1.277710
E_2 = -9239.99763891 A.U.

At No.	X	Y	Z
6	-0.904392000	1.222835000	-4.090481000
6	0.312919000	1.588205000	-3.500038000
6	-2.047453000	1.952837000	-3.777421000
6	0.363748000	2.677315000	-2.626912000
6	-2.000659000	3.027723000	-2.891280000
6	-0.783939000	3.408233000	-2.315105000
1	-2.984791000	1.635654000	-4.219295000
1	1.300773000	2.970964000	-2.162673000
6	-0.982085000	0.004605000	-4.988928000
1	-1.801191000	0.129453000	-5.701453000
1	-0.053411000	-0.091835000	-5.556430000
6	-1.202597000	-1.250956000	-4.169775000
6	-0.122777000	-1.971125000	-3.666991000
6	-2.496781000	-1.655614000	-3.821892000
6	-0.308379000	-3.057782000	-2.812745000
6	-2.686138000	-2.769573000	-3.008054000
6	-1.602115000	-3.479370000	-2.489396000
1	0.873029000	-1.619693000	-3.909087000
1	-3.681888000	-3.090407000	-2.720570000
6	-1.833837000	-4.635561000	-1.537370000
1	-1.014707000	-5.353072000	-1.631726000
1	-2.758009000	-5.149015000	-1.813123000
6	-1.923878000	-4.179772000	-0.094119000
6	-0.760070000	-3.933452000	0.633423000
6	-3.156646000	-3.941662000	0.522739000
6	-0.810437000	-3.477000000	1.948345000
6	-3.206405000	-3.538869000	1.856611000
6	-2.043723000	-3.310672000	2.588624000
1	0.186449000	-4.091564000	0.129349000
1	-4.153179000	-3.366576000	2.354281000
6	-2.104982000	-2.871475000	4.037959000
1	-1.289123000	-3.343656000	4.592301000
1	-3.047607000	-3.213952000	4.473863000
6	-1.999026000	-1.369200000	4.180229000

6	-3.128485000	-0.561006000	4.012682000
6	-0.771920000	-0.755643000	4.422892000
6	-3.008727000	0.823559000	4.065235000
6	-0.646632000	0.633246000	4.448316000
6	-1.773971000	1.439733000	4.259604000
1	0.098573000	-1.392237000	4.528378000
1	-3.866336000	1.465209000	3.908484000
6	-1.661849000	2.948775000	4.180071000
1	-2.584543000	3.397355000	4.559495000
1	-0.839295000	3.292455000	4.812017000
6	-1.424764000	3.410083000	2.755664000
6	-2.487535000	3.552859000	1.855024000
6	-0.131836000	3.659854000	2.299607000
6	-2.240070000	3.941882000	0.541173000
6	0.119959000	4.032528000	0.977266000
6	-0.946033000	4.184494000	0.082998000
1	0.681962000	3.534013000	3.005416000
1	-3.047829000	4.049687000	-0.173164000
6	-0.711045000	4.583373000	-1.360331000
1	-1.461298000	5.324989000	-1.648588000
1	0.270376000	5.053669000	-1.447629000
8	0.296717000	-3.170583000	2.687899000
6	1.562491000	-3.421893000	2.120403000
1	1.711447000	-2.844760000	1.199222000
1	1.696521000	-4.490558000	1.907941000
8	0.726831000	-3.747782000	-2.243588000
6	1.988140000	-3.106835000	-2.196163000
1	1.921988000	-2.126781000	-1.709321000
1	2.430097000	-2.977948000	-3.189887000
8	1.390314000	0.831665000	-3.834965000
6	2.585660000	1.021074000	-3.098372000
1	3.014939000	2.013468000	-3.274532000
1	2.417373000	0.894201000	-2.026087000
8	1.368207000	4.268537000	0.481536000
6	2.476944000	3.928512000	1.284216000
1	2.514304000	2.845029000	1.460114000
1	3.356566000	4.224517000	0.715300000
8	0.550111000	1.282200000	4.587293000
6	1.733600000	0.508724000	4.659656000
1	1.713942000	-0.181473000	5.511208000
1	1.916475000	-0.055367000	3.736347000
8	-4.315052000	-1.207897000	3.791823000
6	-5.487842000	-0.431476000	3.793849000
1	-5.612153000	0.108252000	4.741666000
1	-5.500206000	0.292542000	2.968917000
8	-3.740821000	3.304982000	2.342361000
6	-4.834817000	3.469284000	1.467525000
1	-4.901899000	4.501182000	1.098464000
1	-4.789392000	2.781471000	0.613749000
8	-3.099325000	3.764512000	-2.538402000
6	-4.361637000	3.329588000	-2.992958000
1	-4.431162000	3.370220000	-4.088160000
1	-4.588906000	2.316869000	-2.639342000
8	-3.518563000	-0.892956000	-4.319961000
6	-4.819351000	-1.147254000	-3.853208000
1	-5.175862000	-2.142407000	-4.150827000
1	-4.879838000	-1.053561000	-2.762294000
8	-4.271816000	-4.105787000	-0.258095000
6	-5.527382000	-3.896286000	0.339303000
1	-5.704282000	-4.592191000	1.169738000
1	-5.633254000	-2.866498000	0.710043000
6	-0.694750000	-0.482292000	-0.636716000

1	-0.649077000	-0.254367000	-1.702564000
6	-0.031775000	0.592984000	0.158267000
1	0.120863000	1.574468000	-0.286502000
1	-0.168038000	-1.428990000	-0.474418000
6	0.226835000	0.421085000	1.507169000
1	-0.072711000	-0.499488000	2.000951000
1	0.463239000	1.268727000	2.139180000
6	-4.533062000	0.035998000	-0.222127000
1	-4.758025000	-1.008407000	-0.496067000
1	-4.601814000	0.105445000	0.874438000
6	-3.124814000	0.374617000	-0.677013000
1	-3.105842000	0.412957000	-1.772778000
1	-2.850231000	1.374925000	-0.318044000
6	-2.151130000	-0.690359000	-0.192377000
1	-2.194541000	-0.787193000	0.900903000
1	-2.464033000	-1.653554000	-0.599355000
8	-5.459485000	0.919045000	-0.828953000
14	-7.116457000	0.707649000	-0.778463000
6	-7.619547000	-0.837859000	-1.725858000
1	-8.680560000	-1.061152000	-1.563602000
1	-7.466638000	-0.718614000	-2.803564000
1	-7.046922000	-1.714432000	-1.400429000
6	-7.808011000	2.251256000	-1.578530000
1	-7.501276000	3.146735000	-1.026868000
1	-7.445594000	2.351745000	-2.607522000
1	-8.903107000	2.232461000	-1.609069000
6	-7.701683000	0.534483000	0.999720000
1	-8.796649000	0.511394000	1.046162000
1	-7.334322000	-0.394437000	1.450030000
1	-7.358477000	1.369214000	1.620812000
7	5.583964000	-2.550345000	-0.255181000
6	4.672020000	-1.578029000	-0.639543000
6	5.547534000	-2.821171000	1.181269000
6	4.619455000	-1.671255000	1.627810000
7	4.221407000	-0.954223000	0.528959000
8	4.356246000	-1.306980000	-1.775895000
35	2.173538000	-0.101872000	0.640558000
35	6.086279000	-3.840293000	-1.475971000
1	5.038937000	0.699074000	0.578447000
8	4.149315000	2.267614000	-0.847420000
6	5.202904000	2.382969000	-0.252446000
8	5.676498000	1.453966000	0.575051000
6	6.071110000	3.590028000	-0.353422000
6	7.272258000	3.697591000	0.350444000
6	5.645096000	4.637553000	-1.172547000
6	8.041136000	4.849938000	0.232535000
1	7.595562000	2.879346000	0.984189000
1	4.709071000	4.530459000	-1.711263000
1	8.975637000	4.934156000	0.778832000
1	8.215752000	6.793945000	-0.672755000
6	7.613196000	5.894655000	-0.583825000
6	6.414636000	5.788609000	-1.286318000
1	6.081574000	6.602532000	-1.922878000
8	4.297424000	-1.467772000	2.782951000
6	4.894387000	-4.161252000	1.521630000
1	5.546660000	-4.991548000	1.235857000
1	4.710593000	-4.208640000	2.598095000
1	3.942473000	-4.275358000	0.992838000
6	6.931104000	-2.681408000	1.809656000
1	7.366329000	-1.709077000	1.562613000
1	6.855235000	-2.770218000	2.896691000
1	7.593621000	-3.467005000	1.431836000

1	2.644689000	-3.759583000	-1.616203000
1	2.297162000	-3.091996000	2.855669000
1	2.546576000	1.222375000	4.797709000
1	2.468743000	4.465224000	2.240897000
1	3.280093000	0.252642000	-3.432592000
1	-5.087532000	4.024441000	-2.565799000
1	-5.724771000	3.247537000	2.060895000
1	-6.316242000	-1.130836000	3.665677000
1	-6.268230000	-4.075923000	-0.442059000
1	-5.457319000	-0.388006000	-4.310049000

IM1^{PA1}

E_2 = -9234.10297064 A.U.

Zero-point correction= 1.408027 (Hartree/Particle)

Thermal correction to Energy= 1.497826

Thermal correction to Enthalpy= 1.498770

Thermal correction to Gibbs Free Energy= 1.277949

E_2 = -9240.00116763 A.U.

At No.	X	Y	Z
6	-0.472047000	1.451639000	-3.888247000
6	0.724150000	1.736361000	-3.216699000
6	-1.611431000	2.182046000	-3.568482000
6	0.749448000	2.705198000	-2.211422000
6	-1.580347000	3.177483000	-2.592851000
6	-0.396679000	3.438141000	-1.890940000
1	-2.532055000	1.937416000	-4.085743000
1	1.664197000	2.907148000	-1.660077000
6	-0.525868000	0.311156000	-4.883146000
1	-1.350224000	0.470698000	-5.581941000
1	0.403545000	0.278933000	-5.456994000
6	-0.716001000	-1.006304000	-4.158621000
6	0.377985000	-1.702386000	-3.649751000
6	-2.000354000	-1.499597000	-3.902082000
6	0.216692000	-2.859071000	-2.888447000
6	-2.161885000	-2.689625000	-3.197666000
6	-1.064443000	-3.382845000	-2.685551000
1	1.361170000	-1.283470000	-3.820399000
1	-3.148203000	-3.094758000	-2.998367000
6	-1.279146000	-4.640353000	-1.869397000
1	-0.382528000	-5.262847000	-1.918220000
1	-2.105530000	-5.212707000	-2.297440000
6	-1.581400000	-4.321187000	-0.420581000
6	-0.538468000	-4.030688000	0.457480000
6	-2.894788000	-4.245106000	0.054355000
6	-0.786508000	-3.684494000	1.783111000
6	-3.137759000	-3.958216000	1.397049000
6	-2.095408000	-3.684467000	2.278440000
1	0.471077000	-4.060100000	0.061830000
1	-4.148080000	-3.917195000	1.786337000
6	-2.367080000	-3.369510000	3.735276000
1	-1.607986000	-3.852205000	4.357734000
1	-3.340031000	-3.782909000	4.014349000
6	-2.351801000	-1.880722000	3.997178000
6	-3.481869000	-1.100284000	3.730382000
6	-1.193921000	-1.248592000	4.443903000
6	-3.427559000	0.281529000	3.891306000
6	-1.130243000	0.136548000	4.576007000
6	-2.255541000	0.918283000	4.295431000
1	-0.323375000	-1.865151000	4.630754000
1	-4.282721000	0.905814000	3.663128000
6	-2.185008000	2.429012000	4.348106000

1	-3.176205000	2.827939000	4.581071000
1	-1.502686000	2.740605000	5.142910000
6	-1.711585000	2.999153000	3.027946000
6	-2.606470000	3.205683000	1.972663000
6	-0.368200000	3.309638000	2.826357000
6	-2.144812000	3.702560000	0.758508000
6	0.098787000	3.816870000	1.609258000
6	-0.798767000	4.003745000	0.548088000
1	0.312197000	3.147574000	3.654436000
1	-2.822702000	3.860234000	-0.071068000
6	-0.342248000	4.503921000	-0.810160000
1	-0.974797000	5.346644000	-1.107001000
1	0.681460000	4.873164000	-0.729593000
8	0.191687000	-3.319403000	2.666321000
6	1.533362000	-3.388576000	2.232224000
1	1.715523000	-2.729547000	1.374459000
1	1.809249000	-4.416020000	1.967690000
8	1.259199000	-3.526369000	-2.306541000
6	2.483490000	-2.828408000	-2.157264000
1	2.352954000	-1.927555000	-1.542830000
1	2.929005000	-2.545722000	-3.115622000
8	1.807080000	1.022872000	-3.621063000
6	2.981967000	1.082819000	-2.835507000
1	3.433611000	2.080977000	-2.866266000
1	2.764147000	0.831477000	-1.794885000
8	1.393792000	4.171479000	1.396876000
6	2.350922000	3.783936000	2.357492000
1	2.406633000	2.690095000	2.432666000
1	3.306691000	4.164693000	2.001026000
8	0.011359000	0.813247000	4.914241000
6	1.196900000	0.071909000	5.126284000
1	1.078628000	-0.653461000	5.939970000
1	1.524720000	-0.450038000	4.217138000
8	-4.596123000	-1.770503000	3.305641000
6	-5.799771000	-1.047063000	3.218255000
1	-6.067527000	-0.592954000	4.181042000
1	-5.749387000	-0.258698000	2.456762000
8	-3.920178000	2.904594000	2.208157000
6	-4.871344000	3.348500000	1.263710000
1	-4.782928000	4.428462000	1.089002000
1	-4.786353000	2.816649000	0.308343000
8	-2.668705000	3.944431000	-2.269297000
6	-3.903330000	3.638055000	-2.880344000
1	-3.856059000	3.771287000	-3.969083000
1	-4.229995000	2.618821000	-2.641194000
8	-3.042265000	-0.742896000	-4.368331000
6	-4.348779000	-1.168936000	-4.074835000
1	-4.572845000	-2.142284000	-4.531010000
1	-4.521100000	-1.231241000	-2.992252000
8	-3.888454000	-4.452129000	-0.865770000
6	-5.218355000	-4.265613000	-0.448761000
1	-5.515757000	-5.000859000	0.310296000
1	-5.376368000	-3.255227000	-0.044674000
6	-0.718223000	-0.575236000	-0.522866000
1	-0.572380000	-0.281256000	-1.564599000
6	-0.104520000	0.435197000	0.390299000
1	-0.175868000	1.493057000	0.142032000
1	-0.234647000	-1.544541000	-0.372159000
6	0.186016000	0.087780000	1.755835000
1	-0.035148000	-0.917634000	2.108241000
1	0.254914000	0.875426000	2.499279000
6	-4.547447000	0.138001000	-0.457167000

1	-4.818709000	-0.911510000	-0.662579000
1	-4.692905000	0.308526000	0.620387000
6	-3.090396000	0.360958000	-0.817298000
1	-2.985555000	0.346050000	-1.908319000
1	-2.783217000	1.360422000	-0.484409000
6	-2.215759000	-0.728649000	-0.209356000
1	-2.369581000	-0.794777000	0.877455000
1	-2.518854000	-1.696368000	-0.619377000
8	-5.365935000	1.018572000	-1.202664000
14	-7.027409000	0.850966000	-1.307124000
6	-7.469092000	-0.657457000	-2.339574000
1	-8.548273000	-0.847585000	-2.305989000
1	-7.187481000	-0.523871000	-3.389088000
1	-6.966784000	-1.559167000	-1.968886000
6	-7.604559000	2.433753000	-2.119755000
1	-7.356004000	3.302403000	-1.500108000
1	-7.121307000	2.568508000	-3.093704000
1	-8.688343000	2.433238000	-2.280301000
6	-7.769894000	0.637950000	0.407685000
1	-8.864033000	0.688130000	0.363605000
1	-7.504452000	-0.334867000	0.836668000
1	-7.428201000	1.416024000	1.098979000
7	5.849285000	-2.448029000	-0.209223000
6	5.110999000	-1.390053000	-0.751433000
6	5.491834000	-2.698293000	1.183764000
6	4.604939000	-1.454622000	1.425502000
7	4.475324000	-0.725101000	0.288031000
8	5.071829000	-1.117094000	-1.934763000
35	1.925510000	0.059578000	0.724727000
35	6.390171000	-3.818651000	-1.327580000
1	4.994225000	0.920608000	0.270878000
8	3.411423000	2.569422000	-0.179151000
6	4.612814000	2.756935000	-0.065877000
8	5.477000000	1.802505000	0.217311000
6	5.223801000	4.109196000	-0.218732000
6	6.606753000	4.298000000	-0.194179000
6	4.371021000	5.202085000	-0.389562000
6	7.131894000	5.576714000	-0.343778000
1	7.258783000	3.441619000	-0.062371000
1	3.298527000	5.031797000	-0.395556000
1	8.207519000	5.724269000	-0.329243000
1	6.693584000	7.664181000	-0.626918000
6	6.280010000	6.666360000	-0.511886000
6	4.899414000	6.479262000	-0.532332000
1	4.235572000	7.328785000	-0.660877000
8	4.069737000	-1.214729000	2.504014000
6	4.669516000	-3.970224000	1.380118000
1	5.274363000	-4.859997000	1.182307000
1	4.314618000	-4.008982000	2.413076000
1	3.807383000	-3.983498000	0.705082000
6	6.723153000	-2.681641000	2.086105000
1	7.297179000	-1.764141000	1.930277000
1	6.416370000	-2.732855000	3.134582000
1	7.367333000	-3.537842000	1.858796000
1	3.172609000	-3.514123000	-1.659840000
1	2.149122000	-3.037281000	3.060485000
1	1.958916000	0.801095000	5.403060000
1	2.137287000	4.220283000	3.341257000
1	3.672984000	0.345196000	-3.238066000
1	-4.623012000	4.347956000	-2.467442000
1	-5.848304000	3.136422000	1.703466000
1	-6.567951000	-1.768609000	2.933465000

1	-5.836529000	-4.401899000	-1.337918000
1	-5.012652000	-0.411851000	-4.495226000

IM2^{PA1}
E_2 = -9234.11934822 A.U.
Zero-point correction= 1.406568 (Hartree/Particle)
Thermal correction to Energy= 1.496307
Thermal correction to Enthalpy= 1.497251
Thermal correction to Gibbs Free Energy= 1.277195
E_2 = -9240.01081949 A.U.

At No.	X	Y	Z
6	0.832363000	1.749839000	-3.907162000
6	-0.274776000	0.892918000	-3.984896000
6	2.108602000	1.195213000	-3.859688000
6	-0.076645000	-0.488508000	-4.006283000
6	2.303014000	-0.184197000	-3.895447000
6	1.201849000	-1.044269000	-3.955952000
1	2.948451000	1.876371000	-3.789409000
1	-0.922242000	-1.163773000	-4.057448000
6	0.640117000	3.251132000	-3.879084000
1	1.472342000	3.728844000	-4.403432000
1	-0.280643000	3.502186000	-4.410157000
6	0.556958000	3.794581000	-2.468877000
6	-0.664565000	3.905655000	-1.813407000
6	1.725055000	4.151674000	-1.770973000
6	-0.733048000	4.378694000	-0.502458000
6	1.653668000	4.617973000	-0.462242000
6	0.422219000	4.738617000	0.186973000
1	-1.579334000	3.574230000	-2.298537000
1	2.547522000	4.866354000	0.097428000
6	0.356014000	5.138553000	1.644128000
1	-0.627363000	5.557654000	1.867559000
1	1.108333000	5.901687000	1.856694000
6	0.591961000	3.925711000	2.519118000
6	-0.462826000	3.071084000	2.845041000
6	1.885393000	3.594050000	2.933071000
6	-0.243939000	1.906213000	3.582795000
6	2.096602000	2.455630000	3.705992000
6	1.044578000	1.610999000	4.048148000
1	-1.453827000	3.320425000	2.477968000
1	3.087913000	2.190106000	4.052258000
6	1.290419000	0.388942000	4.904356000
1	0.447584000	0.252133000	5.587358000
1	2.187635000	0.552223000	5.508551000
6	1.462836000	-0.863335000	4.077575000
6	2.687984000	-1.145576000	3.459628000
6	0.404765000	-1.747265000	3.900771000
6	2.824386000	-2.288113000	2.675971000
6	0.540555000	-2.892202000	3.115549000
6	1.759503000	-3.172106000	2.495196000
1	-0.541066000	-1.503488000	4.371927000
1	3.764444000	-2.521823000	2.186069000
6	1.916761000	-4.372122000	1.588855000
1	2.893873000	-4.832052000	1.752183000
1	1.149756000	-5.112100000	1.828968000
6	1.790170000	-3.959400000	0.139639000
6	2.915164000	-3.793505000	-0.668024000
6	0.532482000	-3.693122000	-0.402975000
6	2.775807000	-3.347894000	-1.982378000
6	0.393949000	-3.271330000	-1.721055000
6	1.525794000	-3.076752000	-2.526379000

1	-0.332633000	-3.831121000	0.236816000
1	3.648783000	-3.185986000	-2.605494000
6	1.387802000	-2.547279000	-3.938053000
1	2.282655000	-2.811608000	-4.508205000
1	0.529033000	-3.020083000	-4.423092000
8	-1.212290000	1.005649000	3.902310000
6	-2.563630000	1.329025000	3.604659000
1	-2.723327000	1.516511000	2.538232000
1	-2.885903000	2.200861000	4.188915000
8	-1.947150000	4.460786000	0.140499000
6	-2.686976000	5.621876000	-0.205193000
1	-2.926873000	5.631179000	-1.274627000
1	-2.120543000	6.529514000	0.046432000
8	-1.491465000	1.484777000	-4.047013000
6	-2.636060000	0.666710000	-4.191909000
1	-2.597249000	0.110189000	-5.137994000
1	-2.756629000	-0.025700000	-3.353681000
8	-0.811686000	-3.014307000	-2.310075000
6	-1.984663000	-3.372501000	-1.613361000
1	-2.087711000	-2.795792000	-0.686075000
1	-2.816473000	-3.130438000	-2.274043000
8	-0.485946000	-3.770662000	2.893067000
6	-1.591367000	-3.711137000	3.774219000
1	-1.262018000	-3.671135000	4.819468000
1	-2.241223000	-2.854357000	3.558266000
8	3.695056000	-0.254591000	3.696278000
6	4.976734000	-0.546874000	3.191572000
1	5.331221000	-1.527539000	3.534894000
1	5.001271000	-0.514615000	2.096960000
8	4.135671000	-4.072564000	-0.103045000
6	5.169871000	-4.467610000	-0.977631000
1	4.814897000	-5.214964000	-1.697641000
1	5.590246000	-3.613655000	-1.521137000
8	3.543747000	-0.770051000	-3.885557000
6	4.658518000	0.073649000	-4.067307000
1	4.540727000	0.704720000	-4.957093000
1	4.834954000	0.710410000	-3.189340000
8	2.903246000	3.990033000	-2.446235000
6	4.101813000	4.270978000	-1.761272000
1	4.156558000	5.324539000	-1.458750000
1	4.219161000	3.633750000	-0.874782000
8	2.891342000	4.439690000	2.543905000
6	4.216953000	4.025291000	2.769709000
1	4.453593000	3.977612000	3.840749000
1	4.414806000	3.044665000	2.315278000
6	0.923823000	1.185301000	0.108141000
1	1.053489000	1.997859000	-0.624987000
6	-0.532568000	0.932567000	0.042113000
1	-1.218248000	1.700012000	0.401353000
1	1.159131000	1.627593000	1.081227000
6	-1.164421000	-0.089969000	-0.770770000
1	-0.555681000	-0.750501000	-1.381843000
1	-2.192412000	0.123444000	-1.068375000
6	4.344037000	-0.498151000	-0.507800000
1	4.226612000	-1.368971000	0.152961000
1	4.201821000	-0.852828000	-1.536963000
6	3.305750000	0.554694000	-0.168976000
1	3.516179000	0.944351000	0.836609000
1	3.417238000	1.392765000	-0.870266000
6	1.871818000	0.039213000	-0.221375000
1	1.662734000	-0.363130000	-1.219498000
1	1.734363000	-0.777562000	0.498566000

7	-4.904484000	-3.016942000	-0.378740000
6	-4.644799000	-1.688726000	-0.675672000
6	-5.038693000	-3.272168000	1.052511000
6	-4.482819000	-1.941508000	1.598386000
7	-4.368316000	-1.080794000	0.547717000
8	-4.601394000	-1.182049000	-1.771167000
35	-1.124362000	-0.765173000	1.131001000
35	-5.640432000	-4.121274000	-1.651815000
1	-4.034431000	-0.079468000	0.602429000
8	-3.218377000	1.912509000	-1.598893000
6	-3.745860000	2.075681000	-0.487917000
8	-3.449265000	1.410270000	0.566054000
6	-4.818504000	3.129997000	-0.342494000
6	-5.343170000	3.442187000	0.911185000
6	-5.267138000	3.823929000	-1.465761000
6	-6.292248000	4.451368000	1.044191000
1	-4.986316000	2.888971000	1.773589000
1	-4.843963000	3.566026000	-2.431241000
1	-6.692110000	4.695794000	2.024585000
1	-7.469918000	5.937674000	0.022144000
6	-6.729501000	5.149033000	-0.080551000
6	-6.219374000	4.829341000	-1.337623000
1	-6.563291000	5.367866000	-2.216480000
8	-4.218859000	-1.729435000	2.764553000
6	-6.498784000	-3.420388000	1.490822000
1	-6.923502000	-4.343128000	1.083757000
1	-6.549948000	-3.457730000	2.582091000
1	-7.097142000	-2.574559000	1.138953000
6	-4.186113000	-4.457470000	1.486847000
1	-3.135079000	-4.301995000	1.223607000
1	-4.262717000	-4.586726000	2.568809000
1	-4.539326000	-5.370050000	0.996103000
1	-3.614873000	5.587890000	0.367018000
1	-3.146758000	0.452030000	3.888374000
1	-2.153103000	-4.631851000	3.611840000
1	-1.999419000	-4.442790000	-1.371460000
1	-3.486979000	1.344245000	-4.181060000
1	5.517144000	-0.584550000	-4.203747000
1	5.952577000	-4.901060000	-0.352403000
1	5.638168000	0.229881000	3.580325000
1	4.853294000	4.777607000	2.300069000
1	4.907702000	4.056823000	-2.465145000
8	5.628627000	0.086658000	-0.349549000
14	7.064978000	-0.759012000	-0.409546000
6	7.051246000	-2.166874000	0.841351000
1	6.088677000	-2.690229000	0.863572000
1	7.826311000	-2.906818000	0.609701000
1	7.249381000	-1.788924000	1.850673000
6	7.368746000	-1.421211000	-2.145871000
1	6.498768000	-1.953849000	-2.545848000
1	7.594139000	-0.597710000	-2.832803000
1	8.219146000	-2.112715000	-2.163224000
6	8.390897000	0.487527000	0.030432000
1	9.385612000	0.027298000	0.011662000
1	8.394544000	1.324903000	-0.675394000
1	8.228209000	0.894750000	1.034142000

TS2^{PA1} (Imag. Freq → -243.8520 cm^{-1})
E_2 = -9234.10571804 A.U.
Zero-point correction= 1.405590 (Hartree/Particle)
Thermal correction to Energy= 1.494531

Thermal correction to Enthalpy= 1.495476
Thermal correction to Gibbs Free Energy= 1.280120
E_2 = -9240.00030242 A.U.

At No.	X	Y	Z
6	0.868348000	-0.072335000	-3.972238000
6	-0.369308000	-0.521801000	-3.496900000
6	1.943502000	-0.959543000	-3.966601000
6	-0.516123000	-1.842534000	-3.069038000
6	1.801651000	-2.271345000	-3.524532000
6	0.552257000	-2.735379000	-3.096857000
1	2.901443000	-0.591153000	-4.313021000
1	-1.475523000	-2.206434000	-2.714753000
6	1.038120000	1.328013000	-4.528482000
1	1.886144000	1.323491000	-5.220761000
1	0.145368000	1.587317000	-5.105108000
6	1.259467000	2.397270000	-3.480165000
6	0.230619000	3.260298000	-3.121693000
6	2.497908000	2.539524000	-2.841243000
6	0.398168000	4.232232000	-2.133105000
6	2.662402000	3.491257000	-1.841314000
6	1.616770000	4.332187000	-1.455766000
1	-0.728192000	3.130440000	-3.609359000
1	3.601817000	3.589286000	-1.310065000
6	1.789278000	5.242131000	-0.254186000
1	0.970338000	5.963915000	-0.222812000
1	2.725221000	5.800058000	-0.340778000
6	1.798918000	4.422390000	1.023491000
6	0.598751000	4.043163000	1.624994000
6	2.994105000	3.937317000	1.564573000
6	0.575292000	3.186870000	2.724844000
6	2.972282000	3.105252000	2.682236000
6	1.774089000	2.709189000	3.267520000
1	-0.326542000	4.395330000	1.182937000
1	3.889487000	2.709358000	3.101999000
6	1.766103000	1.766695000	4.452075000
1	0.943696000	2.038464000	5.119402000
1	2.699239000	1.892344000	5.008125000
6	1.617164000	0.306954000	4.069919000
6	2.737066000	-0.478162000	3.776016000
6	0.357877000	-0.291853000	4.028153000
6	2.586684000	-1.835497000	3.493541000
6	0.206229000	-1.645946000	3.732774000
6	1.330752000	-2.437977000	3.471441000
1	-0.503497000	0.331276000	4.239268000
1	3.445569000	-2.456337000	3.266878000
6	1.170255000	-3.910255000	3.139737000
1	2.066820000	-4.449215000	3.458173000
1	0.319982000	-4.314503000	3.694447000
6	0.953733000	-4.124216000	1.656633000
6	2.047208000	-4.148191000	0.785646000
6	-0.330081000	-4.238884000	1.117695000
6	1.845628000	-4.208547000	-0.590169000
6	-0.533505000	-4.310512000	-0.264664000
6	0.564791000	-4.262996000	-1.131283000
1	-1.170397000	-4.229118000	1.803432000
1	2.679931000	-4.159013000	-1.280533000
6	0.374731000	-4.166271000	-2.629088000
1	1.102990000	-4.806179000	-3.133699000
1	-0.623468000	-4.515552000	-2.898418000
8	-0.570587000	2.736567000	3.317875000
6	-1.807312000	3.267486000	2.876412000

1	-1.994094000	3.029830000	1.824374000
1	-1.843507000	4.355300000	3.016179000
8	-0.587631000	5.107895000	-1.774153000
6	-1.690989000	5.227706000	-2.646369000
1	-2.320069000	4.330105000	-2.626096000
1	-1.360091000	5.418577000	-3.675021000
8	-1.389894000	0.391190000	-3.469735000
6	-2.718794000	-0.113862000	-3.495893000
1	-2.865943000	-0.794356000	-4.341710000
1	-2.988518000	-0.637362000	-2.575244000
8	-1.760064000	-4.388495000	-0.854929000
6	-2.904377000	-4.125297000	-0.073486000
1	-2.847043000	-3.123258000	0.370981000
1	-3.760302000	-4.162937000	-0.746759000
8	-0.996195000	-2.284506000	3.694330000
6	-2.166986000	-1.513054000	3.854913000
1	-2.177812000	-0.986928000	4.817381000
1	-2.297843000	-0.794502000	3.037845000
8	3.949776000	0.162809000	3.787010000
6	5.113142000	-0.627207000	3.747968000
1	5.124071000	-1.370646000	4.555457000
1	5.222499000	-1.144764000	2.786077000
8	3.288296000	-4.096087000	1.359075000
6	4.394779000	-4.399002000	0.536448000
1	4.256247000	-5.360980000	0.026602000
1	4.575696000	-3.612148000	-0.205809000
8	2.829046000	-3.173618000	-3.496988000
6	4.130791000	-2.693228000	-3.750863000
1	4.237357000	-2.341097000	-4.785427000
1	4.401627000	-1.894244000	-3.050816000
8	3.491410000	1.691313000	-3.248231000
6	4.725570000	1.761123000	-2.578483000
1	5.214714000	2.732617000	-2.730913000
1	4.609779000	1.580097000	-1.501801000
8	4.149520000	4.302325000	0.923510000
6	5.332484000	3.627559000	1.277390000
1	5.656512000	3.875723000	2.296446000
1	5.210508000	2.538533000	1.197221000
6	0.761128000	1.115625000	-0.306473000
1	0.630181000	0.737677000	-1.324594000
6	-0.405982000	0.717920000	0.533135000
1	-0.380526000	1.006197000	1.583867000
1	0.702496000	2.206771000	-0.368438000
6	-1.715784000	0.520713000	-0.043185000
1	-1.806641000	0.414795000	-1.116132000
1	-2.584102000	0.439717000	0.602197000
6	4.109992000	-0.823256000	0.169496000
1	4.519098000	0.058829000	0.688521000
1	3.939813000	-1.597874000	0.928546000
6	2.790750000	-0.441911000	-0.495000000
1	2.990712000	-0.109624000	-1.518810000
1	2.131195000	-1.314049000	-0.572676000
6	2.110639000	0.684306000	0.283767000
1	1.983365000	0.385973000	1.331750000
1	2.777447000	1.552187000	0.298141000
8	5.044711000	-1.293901000	-0.793049000
14	6.700939000	-1.184476000	-0.610310000
6	7.191398000	0.517500000	0.029751000
1	8.283598000	0.613382000	0.028758000
1	6.787302000	1.325453000	-0.589553000
1	6.852204000	0.679403000	1.059469000
6	7.406357000	-1.492886000	-2.317859000

1	7.096514000	-2.473817000	-2.694503000
1	7.070038000	-0.738483000	-3.037141000
1	8.501997000	-1.474236000	-2.300068000
6	7.356019000	-2.472366000	0.594309000
1	8.418689000	-2.297325000	0.800306000
1	6.825297000	-2.442793000	1.553019000
1	7.258600000	-3.485100000	0.189233000
7	-5.675692000	-2.355756000	-0.159500000
6	-5.165457000	-1.460442000	-1.119669000
6	-5.760936000	-1.716121000	1.150047000
6	-4.755879000	-0.576348000	0.888948000
7	-4.533001000	-0.435557000	-0.437512000
8	-5.225747000	-1.625330000	-2.319617000
35	-0.635906000	-1.298950000	0.538774000
35	-6.928334000	-3.610828000	-0.687442000
1	-4.394257000	1.035175000	-0.848630000
8	-2.446261000	2.571271000	-0.338146000
6	-3.640148000	2.868142000	-0.483983000
8	-4.573254000	2.074031000	-0.912390000
6	-4.113374000	4.245503000	-0.151911000
6	-5.475272000	4.553571000	-0.154976000
6	-3.180368000	5.220343000	0.206457000
6	-5.897576000	5.829405000	0.201618000
1	-6.191002000	3.787461000	-0.430705000
1	-2.124472000	4.977176000	0.163816000
1	-6.956923000	6.066964000	0.201428000
1	-5.297533000	7.794538000	0.840805000
6	-4.964486000	6.799214000	0.561313000
6	-3.604542000	6.495008000	0.560532000
1	-2.874384000	7.251404000	0.832031000
8	-4.258495000	0.113370000	1.779365000
6	-7.136162000	-1.095354000	1.427692000
1	-7.889412000	-1.878986000	1.556095000
1	-7.088853000	-0.495520000	2.340925000
1	-7.442594000	-0.449145000	0.599159000
6	-5.333290000	-2.649287000	2.272231000
1	-4.336149000	-3.054571000	2.081873000
1	-5.316545000	-2.102596000	3.218867000
1	-6.037059000	-3.483870000	2.358314000
1	-2.275173000	6.074316000	-2.284555000
1	-2.573600000	2.793336000	3.490551000
1	-2.994129000	-2.221431000	3.831242000
1	-3.040187000	-4.870728000	0.720891000
1	-3.371375000	0.753812000	-3.597415000
1	4.796581000	-3.545334000	-3.598490000
1	5.254006000	-4.465107000	1.205909000
1	5.951538000	0.060010000	3.878273000
1	6.095565000	3.959425000	0.570994000
1	5.344710000	0.968684000	-3.001657000

TS3^{PA1} (Imag. Freq → -159.1944 cm^{-1})
E_2 = -9234.10967803 A.U.
Zero-point correction= 1.405487 (Hartree/Particle)
Thermal correction to Energy= 1.494192
Thermal correction to Enthalpy= 1.495136
Thermal correction to Gibbs Free Energy= 1.281502
E_2 = -9240.00017271 A.U.

At No.	X	Y	Z
6	-0.782132000	3.183276000	2.938401000
6	0.350188000	2.524819000	3.435178000
6	-2.017585000	2.546000000	3.044798000

6	0.219197000	1.251080000	3.998609000
6	-2.146669000	1.283645000	3.614127000
6	-1.016859000	0.614959000	4.098375000
1	-2.880403000	3.072897000	2.656867000
1	1.085008000	0.728371000	4.390423000
6	-0.704848000	4.554590000	2.290539000
1	-1.514559000	5.177324000	2.682374000
1	0.238969000	5.031635000	2.563549000
6	-0.804318000	4.484587000	0.778418000
6	0.341706000	4.366576000	0.006691000
6	-2.044488000	4.453744000	0.117072000
6	0.285575000	4.195791000	-1.374194000
6	-2.102938000	4.298810000	-1.263687000
6	-0.940674000	4.159856000	-2.029620000
1	1.310090000	4.323033000	0.488156000
1	-3.055330000	4.237385000	-1.776671000
6	-1.028549000	3.857364000	-3.510265000
1	-0.108894000	4.178316000	-4.005683000
1	-1.862628000	4.406590000	-3.953011000
6	-1.217348000	2.369390000	-3.725293000
6	-0.110998000	1.524846000	-3.787773000
6	-2.495202000	1.807074000	-3.772612000
6	-0.261574000	0.143456000	-3.899454000
6	-2.647136000	0.425156000	-3.898345000
6	-1.542339000	-0.420497000	-3.974910000
1	0.872760000	1.976365000	-3.718563000
1	-3.634796000	-0.021779000	-3.948037000
6	-1.710498000	-1.911742000	-4.193213000
1	-0.914317000	-2.255879000	-4.859878000
1	-2.662991000	-2.086632000	-4.699778000
6	-1.670732000	-2.742816000	-2.928214000
6	-2.851591000	-3.095609000	-2.255356000
6	-0.467824000	-3.205164000	-2.405268000
6	-2.808116000	-3.846004000	-1.084419000
6	-0.430491000	-3.981004000	-1.250222000
6	-1.595806000	-4.295640000	-0.558414000
1	0.460476000	-2.947748000	-2.906868000
1	-3.714914000	-4.070472000	-0.535344000
6	-1.551396000	-5.009120000	0.777685000
1	-2.454966000	-5.611722000	0.901744000
1	-0.686830000	-5.676340000	0.817886000
6	-1.454733000	-3.986456000	1.889553000
6	-2.598836000	-3.356320000	2.381098000
6	-0.212148000	-3.585088000	2.378229000
6	-2.487337000	-2.317877000	3.299563000
6	-0.098475000	-2.544899000	3.298985000
6	-1.247559000	-1.887600000	3.763342000
1	0.671655000	-4.083338000	1.996549000
1	-3.374240000	-1.806701000	3.656923000
6	-1.145356000	-0.745651000	4.752670000
1	-2.038776000	-0.748859000	5.384741000
1	-0.280621000	-0.910270000	5.400564000
8	0.785432000	-0.725660000	-3.956748000
6	2.092021000	-0.197442000	-3.859035000
1	2.247184000	0.333318000	-2.912821000
1	2.315001000	0.486243000	-4.686713000
8	1.450546000	3.971328000	-2.077117000
6	2.241843000	5.133185000	-2.268315000
1	2.489451000	5.612169000	-1.313503000
1	1.721586000	5.857551000	-2.909968000
8	1.535120000	3.195637000	3.372143000
6	2.687004000	2.557100000	3.877636000

1	2.585908000	2.345163000	4.949669000
1	2.906470000	1.632136000	3.333641000
8	1.094427000	-2.097212000	3.789402000
6	2.244448000	-2.907800000	3.608049000
1	2.548891000	-2.961340000	2.559312000
1	3.044703000	-2.423603000	4.167231000
8	0.790961000	-4.383022000	-0.738614000
6	1.391141000	-5.475999000	-1.413282000
1	0.774539000	-6.378677000	-1.315200000
1	1.538025000	-5.254811000	-2.477365000
8	-4.027948000	-2.666273000	-2.809349000
6	-5.199794000	-3.366203000	-2.446417000
1	-5.048373000	-4.450049000	-2.519079000
1	-5.527701000	-3.114001000	-1.430115000
8	-3.821376000	-3.780436000	1.906044000
6	-4.825619000	-3.981426000	2.881158000
1	-4.452333000	-4.602711000	3.704295000
1	-5.203452000	-3.036147000	3.287327000
8	-3.346020000	0.645448000	3.762045000
6	-4.501222000	1.318509000	3.314206000
1	-4.639719000	2.272946000	3.839176000
1	-4.469046000	1.493257000	2.231920000
8	-3.154870000	4.563418000	0.905125000
6	-4.403768000	4.229212000	0.334820000
1	-4.700175000	4.950267000	-0.438109000
1	-4.390484000	3.214745000	-0.082607000
8	-3.544443000	2.683337000	-3.706146000
6	-4.790794000	2.168764000	-3.299090000
1	-5.239806000	1.521960000	-4.064633000
1	-4.692834000	1.613568000	-2.359272000
6	1.667972000	0.398168000	0.058522000
1	2.492553000	0.440246000	-0.642569000
6	1.918101000	-0.303114000	1.298566000
1	1.152357000	-0.236499000	2.067825000
1	2.949983000	-0.367906000	1.644114000
7	5.153585000	-2.729486000	-0.207216000
6	4.823143000	-1.522081000	0.430063000
6	5.143656000	-2.576310000	-1.662571000
6	4.948450000	-1.042623000	-1.747076000
7	4.760143000	-0.520545000	-0.503895000
8	4.636240000	-1.425225000	1.634467000
35	1.585909000	-1.913558000	0.197328000
35	4.553071000	-4.290457000	0.598885000
1	4.736629000	0.980235000	-0.282892000
8	2.862701000	2.295449000	0.769041000
6	3.941475000	2.726508000	0.345344000
8	4.874377000	2.029647000	-0.223776000
6	4.272528000	4.182269000	0.459525000
6	5.278149000	4.742855000	-0.330611000
6	3.546684000	4.990171000	1.336630000
6	5.531443000	6.108467000	-0.266067000
1	5.844137000	4.102885000	-0.998396000
1	2.774878000	4.542148000	1.954892000
1	6.304255000	6.544462000	-0.891983000
1	4.997207000	7.981822000	0.650051000
6	4.797110000	6.915373000	0.600686000
6	3.811185000	6.353402000	1.408708000
1	3.244283000	6.977580000	2.092900000
8	4.952359000	-0.438618000	-2.807163000
6	6.485500000	-2.993434000	-2.259495000
1	6.624991000	-4.074797000	-2.152687000
1	6.516241000	-2.729999000	-3.320355000

1	7.306430000	-2.485128000	-1.746559000
6	3.983857000	-3.270194000	-2.375092000
1	3.024960000	-2.994819000	-1.928156000
1	3.988260000	-2.961719000	-3.423946000
1	4.095112000	-4.357833000	-2.333392000
1	3.164223000	4.808024000	-2.752785000
1	2.776557000	-1.040919000	-3.899764000
1	2.360416000	-5.627123000	-0.935281000
1	2.073231000	-3.918867000	3.997948000
1	3.511429000	3.254533000	3.723912000
1	-5.340901000	0.661094000	3.549508000
1	-5.646997000	-4.493410000	2.375565000
1	-5.971669000	-3.054868000	-3.151795000
1	-5.441736000	3.033026000	-3.147187000
1	-5.125752000	4.273611000	1.153004000
6	0.352984000	1.003797000	-0.254138000
1	0.285795000	1.853447000	0.443039000
1	0.361528000	1.414576000	-1.264652000
6	-3.385225000	-0.004582000	-0.105901000
1	-3.409231000	-0.687433000	-0.967922000
1	-3.330727000	-0.616213000	0.807091000
6	-2.171362000	0.901213000	-0.194578000
1	-2.184909000	1.435938000	-1.149907000
1	-2.232064000	1.652696000	0.599829000
6	-0.880326000	0.103672000	-0.054478000
1	-0.856127000	-0.375066000	0.933228000
1	-0.854624000	-0.698041000	-0.801789000
8	-4.563741000	0.795649000	-0.076569000
14	-6.099098000	0.130841000	-0.083969000
6	-6.133510000	-1.375757000	1.036111000
1	-7.102534000	-1.886229000	0.988745000
1	-5.352435000	-2.100635000	0.777751000
1	-5.961193000	-1.081413000	2.077539000
6	-6.595499000	-0.336637000	-1.837928000
1	-6.972112000	0.533571000	-2.386669000
1	-5.735575000	-0.733912000	-2.388448000
1	-7.383459000	-1.098538000	-1.843212000
6	-7.216280000	1.492742000	0.557730000
1	-6.968584000	1.763919000	1.589647000
1	-7.115871000	2.393530000	-0.058683000
1	-8.269082000	1.189085000	0.532366000

P^{PA1}

E_2 = -9234.20291199 A.U.

Zero-point correction= 1.412509 (Hartree/Particle)
Thermal correction to Energy= 1.501095
Thermal correction to Enthalpy= 1.502039
Thermal correction to Gibbs Free Energy= 1.287527

E_2 = -9240.09170703 A.U.

At No.	X	Y	Z
6	1.362560000	-0.097832000	-4.380359000
6	0.171335000	-0.778780000	-4.105071000
6	2.570716000	-0.757084000	-4.164354000
6	0.211809000	-2.097342000	-3.655131000
6	2.611523000	-2.053955000	-3.660942000
6	1.421617000	-2.742159000	-3.406759000
1	3.484647000	-0.209139000	-4.356076000
1	-0.699017000	-2.639998000	-3.435316000
6	1.354566000	1.339132000	-4.862085000
1	2.293106000	1.538663000	-5.389151000
1	0.537909000	1.482248000	-5.575715000

6	1.193853000	2.329558000	-3.728403000
6	-0.020020000	2.973435000	-3.511084000
6	2.251535000	2.602984000	-2.850307000
6	-0.210484000	3.843680000	-2.434146000
6	2.056885000	3.449442000	-1.766536000
6	0.830954000	4.074182000	-1.532079000
1	-0.833448000	2.754786000	-4.193886000
1	2.861713000	3.660836000	-1.074677000
6	0.668386000	4.985768000	-0.328650000
1	-0.312790000	5.463085000	-0.369054000
1	1.417811000	5.781864000	-0.384426000
6	0.817813000	4.251998000	0.988901000
6	-0.300747000	3.732945000	1.640516000
6	2.073586000	4.051725000	1.569633000
6	-0.191098000	3.008311000	2.827755000
6	2.189226000	3.322345000	2.750187000
6	1.073304000	2.792008000	3.390122000
1	-1.273215000	3.882409000	1.187042000
1	3.158072000	3.134200000	3.197269000
6	1.243758000	1.958567000	4.641412000
1	0.388044000	2.112365000	5.304166000
1	2.142857000	2.283479000	5.172114000
6	1.352748000	0.488932000	4.299665000
6	2.579919000	-0.093448000	3.961920000
6	0.210402000	-0.300167000	4.261444000
6	2.635257000	-1.433106000	3.588127000
6	0.262325000	-1.636240000	3.873572000
6	1.482466000	-2.217017000	3.524138000
1	-0.733921000	0.174376000	4.498501000
1	3.574983000	-1.903366000	3.318769000
6	1.557613000	-3.650039000	3.046701000
1	2.472621000	-4.110214000	3.427953000
1	0.710269000	-4.207995000	3.452899000
6	1.539989000	-3.760682000	1.534863000
6	2.727935000	-3.894164000	0.810336000
6	0.332010000	-3.729725000	0.837865000
6	2.692324000	-4.038154000	-0.576292000
6	0.299313000	-3.865060000	-0.548068000
6	1.486716000	-4.028783000	-1.270734000
1	-0.580170000	-3.591809000	1.408280000
1	3.604638000	-4.122694000	-1.154547000
6	1.453566000	-4.122333000	-2.783255000
1	2.336690000	-4.663397000	-3.131910000
1	0.569279000	-4.683583000	-3.096111000
8	-1.256328000	2.497238000	3.503404000
6	-2.550269000	2.709080000	2.972123000
1	-2.665266000	2.248926000	1.986353000
1	-2.788254000	3.778974000	2.911898000
8	-1.393189000	4.490906000	-2.197866000
6	-2.374662000	4.467887000	-3.210421000
1	-2.777367000	3.459903000	-3.371464000
1	-1.977090000	4.852244000	-4.158278000
8	-0.990845000	-0.072630000	-4.268757000
6	-2.210038000	-0.751866000	-4.050858000
1	-2.363937000	-1.550698000	-4.786246000
1	-2.261756000	-1.191223000	-3.046998000
8	-0.844602000	-3.871240000	-1.289447000
6	-2.065288000	-3.583824000	-0.648690000
1	-2.036751000	-2.589959000	-0.184924000
1	-2.819921000	-3.611470000	-1.434832000
8	-0.848768000	-2.435067000	3.802806000
6	-2.011230000	-1.979479000	4.454376000

1	-1.807103000	-1.716529000	5.500285000
1	-2.447268000	-1.110466000	3.944553000
8	3.679435000	0.724707000	4.022853000
6	4.946792000	0.119103000	3.964669000
1	5.055499000	-0.664636000	4.725821000
1	5.144305000	-0.315572000	2.977108000
8	3.891805000	-3.871964000	1.536095000
6	5.100187000	-4.020392000	0.827826000
1	5.137997000	-4.977819000	0.291627000
1	5.251126000	-3.197219000	0.116773000
8	3.770268000	-2.721385000	-3.378608000
6	4.977854000	-1.997146000	-3.447520000
1	5.201987000	-1.682641000	-4.475799000
1	4.959054000	-1.127498000	-2.781199000
8	3.446956000	1.999991000	-3.125147000
6	4.541048000	2.296409000	-2.290582000
1	4.794031000	3.364641000	-2.324667000
1	4.350707000	2.001591000	-1.250809000
8	3.147018000	4.609214000	0.923170000
6	4.432642000	4.265846000	1.378885000
1	4.629785000	4.663457000	2.383019000
1	4.573699000	3.177399000	1.392358000
6	0.406031000	0.031412000	-0.752563000
1	0.121612000	-0.962733000	-1.116815000
6	-0.782885000	0.761676000	-0.162812000
1	-0.453820000	1.680443000	0.324065000
1	0.714000000	0.611603000	-1.632270000
6	-1.876654000	1.086036000	-1.153040000
1	-1.443912000	1.434964000	-2.094729000
1	-2.511318000	0.223058000	-1.352572000
6	4.087143000	-0.452695000	0.366124000
1	4.140344000	0.504500000	0.911269000
1	4.021496000	-1.255886000	1.111391000
6	2.859702000	-0.467547000	-0.525931000
1	3.028488000	0.215727000	-1.366108000
1	2.744800000	-1.465931000	-0.958555000
6	1.586988000	-0.061406000	0.212427000
1	1.360413000	-0.768862000	1.018394000
1	1.735549000	0.921446000	0.685733000
8	5.251546000	-0.633814000	-0.431466000
14	6.806446000	-0.239510000	0.012356000
6	6.872580000	1.497259000	0.734802000
1	7.909743000	1.778198000	0.952394000
1	6.467545000	2.237544000	0.035894000
1	6.309118000	1.573691000	1.671400000
6	7.805289000	-0.331011000	-1.570523000
1	7.745380000	-1.332278000	-2.010865000
1	7.441318000	0.383505000	-2.316713000
1	8.862761000	-0.111633000	-1.383714000
6	7.505673000	-1.451831000	1.271125000
1	8.453443000	-1.080319000	1.678590000
1	6.824146000	-1.615645000	2.113350000
1	7.701049000	-2.425836000	0.809898000
7	-5.164466000	-2.830761000	-0.168516000
6	-4.978110000	-2.114166000	-1.331537000
6	-5.467161000	-2.001002000	0.998416000
6	-5.165101000	-0.607852000	0.413766000
7	-4.957284000	-0.778670000	-0.930524000
8	-4.823740000	-2.533202000	-2.454230000
35	-1.582933000	-0.289683000	1.309156000
35	-5.523989000	-4.630141000	-0.220362000
1	-4.762329000	-0.005433000	-1.568323000

8	-2.653678000	2.149026000	-0.580902000
6	-3.858464000	2.390810000	-1.078079000
8	-4.366835000	1.737505000	-1.976528000
6	-4.521062000	3.545506000	-0.422477000
6	-5.909613000	3.528277000	-0.293376000
6	-3.782168000	4.632938000	0.046482000
6	-6.556168000	4.588498000	0.329566000
1	-6.464337000	2.673817000	-0.664861000
1	-2.709465000	4.653690000	-0.110822000
1	-7.635242000	4.570339000	0.446279000
1	-6.328438000	6.502032000	1.286659000
6	-5.820165000	5.672645000	0.803717000
6	-4.435430000	5.699290000	0.653723000
1	-3.863152000	6.551294000	1.007508000
8	-5.167115000	0.435046000	1.025554000
6	-6.949261000	-2.060876000	1.381193000
1	-7.201134000	-3.058308000	1.753810000
1	-7.152710000	-1.327126000	2.165369000
1	-7.584491000	-1.840308000	0.517790000
6	-4.558567000	-2.329677000	2.174932000
1	-3.508737000	-2.296918000	1.874825000
1	-4.718765000	-1.598105000	2.971106000
1	-4.787086000	-3.329666000	2.557594000
1	-3.180864000	5.115007000	-2.861109000
1	-3.238678000	2.224600000	3.665835000
1	-2.722711000	-2.806664000	4.419318000
1	-2.316584000	-4.333792000	0.113701000
1	-2.997387000	-0.003023000	-4.148030000
1	5.756429000	-2.684416000	-3.110049000
1	5.892182000	-3.999669000	1.578262000
1	5.673934000	0.911321000	4.155075000
1	5.135568000	4.713486000	0.673676000
1	5.377364000	1.708044000	-2.671165000

PTS

E_1 = -6737.72659131A.U.

Zero-point correction= 0.513334 (Hartree/Particle)

Thermal correction to Energy= 0.552196

Thermal correction to Enthalpy= 0.553140

Thermal correction to Gibbs Free Energy= 0.429563

E_2 = -6743.01545102 A.U.

At No.	X	Y	Z
6	3.665537000	1.668728000	0.550258000
1	3.327125000	0.665253000	0.842513000
6	2.716555000	2.689778000	1.108003000
1	2.626285000	2.712457000	2.195062000
1	3.640954000	1.701337000	-0.546161000
6	2.013693000	3.566628000	0.384099000
1	2.088401000	3.594445000	-0.701055000
1	1.356022000	4.294920000	0.849696000
6	5.102087000	1.877821000	1.045445000
1	5.113103000	1.861819000	2.144272000
1	5.442790000	2.878345000	0.746618000
8	8.347602000	0.004560000	0.535694000
14	9.405667000	0.217397000	-0.739860000
6	10.737142000	1.460145000	-0.270244000
1	11.306001000	1.116284000	0.600414000
1	11.442595000	1.607488000	-1.096604000
1	10.311039000	2.439801000	-0.024691000
6	8.485206000	0.848058000	-2.255528000
1	9.174056000	0.996597000	-3.095530000
1	7.714138000	0.138435000	-2.575163000

1	7.995818000	1.809612000	-2.059747000
6	10.149748000	-1.468475000	-1.061707000
1	9.375207000	-2.192872000	-1.334450000
1	10.878016000	-1.427710000	-1.879860000
1	10.663418000	-1.846144000	-0.171405000
7	-0.851293000	-0.355682000	0.240309000
6	-2.200854000	-0.275901000	0.054080000
6	-0.355214000	-1.688066000	0.245164000
7	-1.458962000	-2.453145000	-0.027800000
6	-2.729119000	-1.714702000	-0.028511000
8	-2.851859000	0.746358000	-0.014197000
8	0.784324000	-2.017829000	0.431190000
35	0.284267000	1.099305000	0.430803000
1	-4.546719000	1.236419000	-0.193521000
8	-6.209906000	-0.381752000	-0.046855000
6	-6.394452000	0.812947000	-0.205340000
8	-5.410547000	1.700190000	-0.289743000
6	-7.750415000	1.418213000	-0.325990000
6	-7.928998000	2.791845000	-0.503891000
6	-8.859217000	0.572941000	-0.256792000
6	-9.213222000	3.313473000	-0.611839000
1	-7.062430000	3.441385000	-0.556163000
1	-8.696225000	-0.490629000	-0.117928000
1	-9.352677000	4.381280000	-0.750084000
1	-11.320284000	2.878546000	-0.627386000
6	-10.318237000	2.468058000	-0.542688000
6	-10.141011000	1.097434000	-0.365053000
1	-11.002554000	0.438957000	-0.311162000
6	6.063180000	0.818266000	0.508781000
1	5.732089000	-0.182170000	0.812573000
1	6.058888000	0.832211000	-0.588818000
6	7.490712000	1.025044000	1.001213000
1	7.512144000	1.006569000	2.098416000
1	7.845319000	2.023104000	0.694334000
35	-1.388769000	-4.282017000	0.134411000
6	-3.574122000	-2.009807000	1.213472000
1	-4.468303000	-1.382698000	1.194792000
1	-3.885503000	-3.058434000	1.201100000
1	-3.003767000	-1.824556000	2.129042000
6	-3.506703000	-1.942778000	-1.321115000
1	-2.905140000	-1.670856000	-2.192988000
1	-3.773655000	-3.001471000	-1.397270000
1	-4.426813000	-1.354661000	-1.300501000

IM1

E_1 = -6737.70297451 A.U.

Zero-point correction= 0.514122 (Hartree/Particle)

Thermal correction to Energy= 0.551401

Thermal correction to Enthalpy= 0.552345

Thermal correction to Gibbs Free Energy= 0.436325

E_2 = -6742.98716461 A.U.

At No.	X	Y	Z
6	-1.626210000	1.093961000	-1.433341000
1	-1.697183000	2.132294000	-1.087914000
6	-0.221382000	0.596743000	-1.357918000
1	0.001661000	-0.454908000	-1.607240000
1	-1.858915000	1.097236000	-2.509914000
6	0.940047000	1.475836000	-1.330838000
1	0.809906000	2.556003000	-1.334674000
1	1.895684000	1.028224000	-1.628233000
6	-2.626444000	0.196209000	-0.702086000

1	-2.387091000	0.210647000	0.369286000
1	-2.491806000	-0.839675000	-1.037872000
8	-6.365623000	0.249809000	-0.411499000
14	-7.729095000	-0.471938000	0.244570000
6	-7.636675000	-0.423748000	2.121791000
1	-7.539303000	0.604933000	2.485966000
1	-8.544945000	-0.849975000	2.563738000
1	-6.786649000	-0.998860000	2.506430000
6	-7.855198000	-2.254172000	-0.338645000
1	-8.769942000	-2.721380000	0.044691000
1	-7.884817000	-2.310325000	-1.432323000
1	-7.010325000	-2.861160000	0.006106000
6	-9.170273000	0.542480000	-0.381248000
1	-9.212209000	0.531747000	-1.475417000
1	-10.120787000	0.148306000	-0.003909000
1	-9.086519000	1.585490000	-0.058443000
7	2.787700000	-2.985924000	-0.435566000
6	2.018527000	-1.975222000	-1.020739000
6	3.993783000	-2.402124000	0.151171000
6	3.968657000	-1.060808000	-0.598070000
7	2.791523000	-0.853312000	-1.207188000
8	0.836269000	-2.114866000	-1.324878000
35	0.625844000	0.787053000	0.518463000
35	1.924359000	-4.395261000	0.404853000
1	4.535303000	1.205932000	-0.615256000
8	3.380637000	1.659665000	1.406999000
6	3.485785000	2.415151000	0.454750000
8	4.172086000	2.162279000	-0.640306000
6	2.740369000	3.717017000	0.391732000
6	2.840167000	4.569432000	-0.710462000
6	1.868339000	4.032011000	1.435353000
6	2.068950000	5.726518000	-0.765746000
1	3.520649000	4.315898000	-1.516110000
1	1.812208000	3.358902000	2.284970000
1	2.150883000	6.390305000	-1.621246000
1	0.593930000	6.937388000	0.229804000
6	1.195485000	6.034201000	0.275427000
6	1.097456000	5.186941000	1.377648000
1	0.422355000	5.430125000	2.192685000
6	-4.069473000	0.642783000	-0.921344000
1	-4.201460000	1.681023000	-0.592540000
1	-4.319785000	0.612060000	-1.988755000
6	-5.064365000	-0.228891000	-0.168833000
1	-4.834357000	-0.204982000	0.908498000
1	-4.963380000	-1.274385000	-0.500637000
8	4.937909000	-0.288193000	-0.542322000
6	5.233292000	-3.233803000	-0.141014000
1	6.124948000	-2.687280000	0.176705000
1	5.186577000	-4.186007000	0.398415000
1	5.310355000	-3.443019000	-1.211413000
6	3.841067000	-2.082655000	1.643598000
1	2.937508000	-1.487781000	1.814579000
1	3.781849000	-3.001622000	2.234673000
1	4.699659000	-1.492795000	1.973527000

PA5

E_1 = -4461.57954061 A.U.

Zero-point correction= 2.336339 (Hartree/Particle)

Thermal correction to Energy= 2.452579

Thermal correction to Enthalpy= 2.453523

Thermal correction to Gibbs Free Energy= 2.180718

E_2 = -4462.74093533 A.U.

At No.	X	Y	Z
6	-0.326386000	-3.062828000	-2.882264000
6	0.918860000	-2.562632000	-3.269226000
6	-1.453861000	-2.272378000	-3.088551000
6	1.002884000	-1.319395000	-3.892498000
6	-1.366054000	-1.012300000	-3.676341000
6	-0.125082000	-0.533221000	-4.110306000
1	-2.401487000	-2.668926000	-2.744588000
1	1.957426000	-0.915030000	-4.207142000
6	-0.464639000	-4.397160000	-2.180817000
1	-1.411436000	-4.860860000	-2.468629000
1	0.342464000	-5.064174000	-2.494606000
6	-0.413093000	-4.223758000	-0.678150000
6	0.817804000	-4.153420000	-0.037285000
6	-1.572075000	-4.048738000	0.084115000
6	0.919170000	-3.914224000	1.331206000
6	-1.475912000	-3.866087000	1.461568000
6	-0.238482000	-3.792984000	2.102453000
1	1.701410000	-4.255243000	-0.655482000
1	-2.362475000	-3.735327000	2.070748000
6	-0.160390000	-3.504531000	3.587015000
1	0.741740000	-3.960219000	4.002955000
1	-1.021757000	-3.953154000	4.088741000
6	-0.135017000	-2.014054000	3.852243000
6	1.077319000	-1.334028000	3.933335000
6	-1.322270000	-1.284945000	3.969342000
6	1.121062000	0.043898000	4.129019000
6	-1.277988000	0.096535000	4.147764000
6	-0.065305000	0.777199000	4.223406000
1	1.984542000	-1.915740000	3.819933000
1	-2.189003000	0.680305000	4.227282000
6	-0.032882000	2.282013000	4.373014000
1	0.794050000	2.565303000	5.030557000
1	-0.961377000	2.613162000	4.844837000
6	0.126672000	2.995111000	3.045580000
6	-0.983611000	3.394328000	2.306280000
6	1.396712000	3.260594000	2.521701000
6	-0.819219000	4.116970000	1.128434000
6	1.554355000	3.894550000	1.293191000
6	0.435086000	4.368491000	0.594355000
1	2.255378000	2.921569000	3.088333000
1	-1.692372000	4.462345000	0.585360000
6	0.566505000	5.029741000	-0.760411000
1	-0.207956000	5.793328000	-0.868671000
1	1.538328000	5.521562000	-0.846188000
6	0.423866000	3.992118000	-1.853800000
6	-0.829134000	3.647396000	-2.366037000
6	1.540515000	3.290219000	-2.298858000
6	-0.937148000	2.641464000	-3.322417000
6	1.431855000	2.266737000	-3.238103000
6	0.179285000	1.939604000	-3.769196000
1	2.495296000	3.550150000	-1.857866000
1	-1.902442000	2.356414000	-3.724802000
6	0.014540000	0.820256000	-4.774210000
1	-0.870667000	1.019177000	-5.385137000
1	0.879040000	0.798576000	-5.443909000
8	2.282171000	0.756511000	4.260400000
6	3.498733000	0.120687000	3.943870000
1	3.492769000	-0.193561000	2.887806000
1	3.637707000	-0.780495000	4.559270000
8	2.130742000	-3.779240000	1.967588000
6	3.188365000	-3.329151000	1.147047000

1	2.857039000	-2.441657000	0.586025000
1	3.456700000	-4.100214000	0.411845000
8	2.013904000	-3.347533000	-3.011028000
6	3.282135000	-2.739712000	-3.122851000
1	3.487236000	-2.465270000	-4.168971000
1	3.303860000	-1.814928000	-2.525694000
8	2.507309000	1.545546000	-3.689630000
6	3.728427000	1.713162000	-3.002686000
1	4.056209000	2.762143000	-3.057262000
1	3.595428000	1.463318000	-1.937903000
8	2.769670000	4.104535000	0.699495000
6	3.885592000	3.393299000	1.189716000
1	4.139565000	3.732883000	2.205280000
1	3.653532000	2.317990000	1.246162000
8	-2.256148000	3.084928000	2.742931000
6	-2.962669000	2.225869000	1.855892000
1	-2.877723000	2.594120000	0.824278000
1	-2.513355000	1.221767000	1.878020000
8	-1.906295000	4.338304000	-1.877471000
6	-3.189658000	3.794370000	-2.096381000
1	-3.465478000	3.868814000	-3.159546000
1	-3.192219000	2.726864000	-1.826476000
8	-2.451069000	-0.196900000	-3.866369000
6	-3.680111000	-0.636921000	-3.330817000
1	-3.975073000	-1.589351000	-3.797626000
1	-3.575632000	-0.817013000	-2.249547000
8	-2.759254000	-4.066911000	-0.598774000
6	-3.925151000	-3.643522000	0.073453000
1	-4.174021000	-4.343082000	0.886360000
1	-3.763528000	-2.652027000	0.525736000
8	-2.483074000	-2.007083000	3.911997000
6	-3.701744000	-1.315771000	3.753279000
1	-3.882680000	-0.642531000	4.604371000
1	-3.666848000	-0.698347000	2.842296000
6	5.058222000	3.651705000	0.258811000
1	4.775019000	3.373474000	-0.763937000
1	5.260941000	4.729604000	0.248735000
6	6.313219000	2.885905000	0.680043000
1	6.472534000	3.008972000	1.760969000
1	6.160639000	1.809439000	0.514128000
6	7.571700000	3.345540000	-0.056443000
1	7.428370000	3.230374000	-1.139768000
1	7.716133000	4.420727000	0.119878000
6	8.829089000	2.591349000	0.371890000
1	8.941590000	2.673382000	1.460812000
1	8.700371000	1.523255000	0.154778000
6	10.099649000	3.100081000	-0.304587000
1	10.027733000	3.023172000	-1.395494000
1	10.280785000	4.152725000	-0.058826000
1	10.975515000	2.525326000	0.013798000
6	4.631514000	1.093307000	4.216650000
1	4.443359000	2.029857000	3.678870000
1	4.635184000	1.337223000	5.285882000
6	5.980942000	0.515992000	3.797146000
1	6.095178000	-0.493352000	4.218408000
1	5.999159000	0.395819000	2.704719000
6	7.171049000	1.369911000	4.228332000
1	7.044435000	2.394930000	3.851245000
1	7.186135000	1.448887000	5.324241000
6	8.503847000	0.811592000	3.734033000
1	8.625171000	-0.215922000	4.103981000
1	8.473702000	0.741180000	2.639802000

6	9.704546000	1.654428000	4.155696000
1	9.613980000	2.681736000	3.782920000
1	9.787412000	1.705913000	5.247283000
1	10.639514000	1.239042000	3.765761000
6	4.431638000	-3.004608000	1.957959000
1	4.161888000	-2.451412000	2.863824000
1	4.916217000	-3.932891000	2.283163000
6	5.375384000	-2.174130000	1.080928000
1	5.295487000	-2.522682000	0.040710000
1	5.024587000	-1.131592000	1.067390000
6	6.849718000	-2.214006000	1.470738000
1	6.976322000	-1.953286000	2.529772000
1	7.224645000	-3.241309000	1.356938000
6	7.676609000	-1.267155000	0.602756000
1	7.409926000	-1.436361000	-0.449040000
1	7.379822000	-0.232288000	0.822814000
6	9.183953000	-1.421083000	0.777024000
1	9.479161000	-1.263906000	1.820345000
1	9.510734000	-2.425068000	0.484545000
1	9.733963000	-0.700013000	0.161645000
6	4.340605000	-3.708209000	-2.630853000
1	4.135873000	-3.959762000	-1.583567000
1	4.268421000	-4.642020000	-3.200805000
6	5.743829000	-3.117197000	-2.763048000
1	5.960392000	-2.932579000	-3.824839000
1	5.777112000	-2.133539000	-2.270439000
6	6.833817000	-4.008963000	-2.171087000
1	6.676209000	-4.103400000	-1.086526000
1	6.739288000	-5.023603000	-2.582468000
6	8.246593000	-3.488785000	-2.433216000
1	8.411715000	-3.424939000	-3.517402000
1	8.334861000	-2.463212000	-2.052178000
6	9.327375000	-4.360743000	-1.799332000
1	9.177328000	-4.443066000	-0.716244000
1	9.308164000	-5.375648000	-2.212463000
1	10.326163000	-3.945878000	-1.969442000
6	4.794829000	0.833162000	-3.630333000
1	4.450337000	-0.206793000	-3.659079000
1	4.947162000	1.147414000	-4.669978000
6	6.105519000	0.938377000	-2.848173000
1	6.328874000	2.000118000	-2.668743000
1	5.978634000	0.484643000	-1.853544000
6	7.306163000	0.302902000	-3.547449000
1	7.120686000	-0.766995000	-3.711425000
1	7.423554000	0.751280000	-4.543865000
6	8.600876000	0.483138000	-2.756285000
1	8.762897000	1.554854000	-2.580718000
1	8.481746000	0.030146000	-1.763826000
6	9.825946000	-0.111334000	-3.445654000
1	9.702698000	-1.187705000	-3.609780000
1	9.993304000	0.356866000	-4.422383000
1	10.729037000	0.035287000	-2.843837000
6	-4.756577000	0.402480000	-3.583949000
1	-4.501188000	1.334328000	-3.064865000
1	-4.791934000	0.630507000	-4.655971000
6	-6.113743000	-0.117439000	-3.109350000
1	-6.344658000	-1.047947000	-3.648056000
1	-6.050637000	-0.390579000	-2.044797000
6	-7.270080000	0.861879000	-3.298765000
1	-7.105526000	1.752275000	-2.676515000
1	-7.290499000	1.217856000	-4.338034000
6	-8.618653000	0.232020000	-2.948799000

1	-8.834382000	-0.567845000	-3.670027000
1	-8.542428000	-0.257580000	-1.969007000
6	-9.770442000	1.232650000	-2.926249000
1	-9.602957000	2.004175000	-2.165866000
1	-9.876218000	1.736436000	-3.893886000
1	-10.720955000	0.739032000	-2.697817000
6	-5.060602000	-3.599284000	-0.934738000
1	-4.801447000	-2.898527000	-1.738734000
1	-5.155020000	-4.589881000	-1.395895000
6	-6.386814000	-3.191794000	-0.294583000
1	-6.597280000	-3.850451000	0.560119000
1	-6.297739000	-2.176318000	0.117794000
6	-7.565611000	-3.248082000	-1.266316000
1	-7.374112000	-2.578172000	-2.115524000
1	-7.637348000	-4.261903000	-1.684682000
6	-8.898310000	-2.875207000	-0.618841000
1	-9.066136000	-3.515307000	0.257577000
1	-8.839200000	-1.847941000	-0.237298000
6	-10.084839000	-2.991998000	-1.572210000
1	-9.954780000	-2.339619000	-2.442841000
1	-10.194944000	-4.018798000	-1.939380000
1	-11.020264000	-2.709959000	-1.077973000
6	-4.813773000	-2.345816000	3.668830000
1	-4.571291000	-3.070033000	2.881513000
1	-4.847624000	-2.904562000	4.612005000
6	-6.170024000	-1.707786000	3.379654000
1	-6.352181000	-0.883973000	4.085046000
1	-6.150488000	-1.254610000	2.379123000
6	-7.329134000	-2.698959000	3.457418000
1	-7.120432000	-3.556103000	2.801635000
1	-7.395917000	-3.103186000	4.477112000
6	-8.666339000	-2.077651000	3.060608000
1	-8.860110000	-1.199340000	3.691818000
1	-8.591198000	-1.705632000	2.031685000
6	-9.836536000	-3.052183000	3.163676000
1	-9.666124000	-3.937226000	2.539363000
1	-9.973603000	-3.397272000	4.194857000
1	-10.772296000	-2.587594000	2.836296000
6	-4.423466000	2.193313000	2.264139000
1	-4.521664000	1.750845000	3.264406000
1	-4.778337000	3.229136000	2.341408000
6	-5.282903000	1.428167000	1.258588000
1	-5.105479000	1.834049000	0.252625000
1	-4.965778000	0.375594000	1.216163000
6	-6.777591000	1.501147000	1.565460000
1	-6.971511000	1.091926000	2.566608000
1	-7.087328000	2.555142000	1.599847000
6	-7.632940000	0.762078000	0.539943000
1	-7.383267000	1.129255000	-0.464126000
1	-7.362911000	-0.302660000	0.544653000
6	-9.131623000	0.913375000	0.784463000
1	-9.407113000	0.537997000	1.776217000
1	-9.433090000	1.965749000	0.732197000
1	-9.718506000	0.361919000	0.041606000
6	-4.185753000	4.556423000	-1.241807000
1	-3.942884000	4.401555000	-0.182267000
1	-4.077295000	5.629748000	-1.437101000
6	-5.621673000	4.112264000	-1.511594000
1	-5.901369000	4.367625000	-2.543166000
1	-5.684177000	3.016494000	-1.444090000
6	-6.626917000	4.723345000	-0.538531000
1	-6.342589000	4.448418000	0.487479000

1	-6.568149000	5.819519000	-0.587800000
6	-8.062181000	4.273977000	-0.804501000
1	-8.360518000	4.586282000	-1.814178000
1	-8.095931000	3.177273000	-0.803627000
6	-9.059051000	4.811069000	0.219056000
1	-8.801782000	4.473796000	1.230372000
1	-9.065741000	5.906940000	0.227208000
1	-10.077039000	4.470723000	0.001459000

PTS^{PA5}

E_1 = -11199.4100843 A.U.

Zero-point correction= 2.854835 (Hartree/Particle)
Thermal correction to Energy= 3.010272
Thermal correction to Enthalpy= 3.011217
Thermal correction to Gibbs Free Energy= 2.650231

E_2 = -11205.8086998 A.U.

At No.	X	Y	Z
6	0.940918000	-0.153173000	4.131292000
6	-0.393646000	0.257482000	4.055197000
6	1.918686000	0.666253000	3.578767000
6	-0.743509000	1.424176000	3.377861000
6	1.578078000	1.855220000	2.935701000
6	0.238037000	2.246723000	2.820315000
1	2.954010000	0.348088000	3.640669000
1	-1.782119000	1.727491000	3.287952000
6	1.281487000	-1.512421000	4.708582000
1	2.285835000	-1.493575000	5.135291000
1	0.578879000	-1.759422000	5.507795000
6	1.214542000	-2.572671000	3.623035000
6	-0.013114000	-2.997273000	3.109933000
6	2.383799000	-3.066644000	3.040161000
6	-0.087619000	-3.875511000	2.032165000
6	2.310238000	-3.980262000	1.989042000
6	1.086735000	-4.390230000	1.468356000
1	-0.910927000	-2.586722000	3.553902000
1	3.207058000	-4.357549000	1.512271000
6	1.037930000	-5.318689000	0.272353000
1	0.148208000	-5.950581000	0.338058000
1	1.913500000	-5.973280000	0.292724000
6	1.007438000	-4.559332000	-1.038835000
6	-0.209259000	-4.214564000	-1.624044000
6	2.188301000	-4.151969000	-1.666068000
6	-0.264636000	-3.490386000	-2.814202000
6	2.134193000	-3.418591000	-2.850382000
6	0.919051000	-3.080746000	-3.439850000
1	-1.113280000	-4.509728000	-1.105882000
1	3.039743000	-3.076555000	-3.337660000
6	0.875569000	-2.286434000	-4.728906000
1	0.084923000	-2.688278000	-5.369726000
1	1.825792000	-2.412155000	-5.255324000
6	0.616032000	-0.812176000	-4.500623000
6	1.665094000	0.067082000	-4.209979000
6	-0.682314000	-0.312099000	-4.521093000
6	1.407543000	1.420204000	-4.009526000
6	-0.946701000	1.026946000	-4.241259000
6	0.105212000	1.918817000	-4.022815000
1	-1.488770000	-1.012571000	-4.699888000
1	2.208617000	2.114731000	-3.787016000
6	-0.173287000	3.365788000	-3.666663000
1	0.596627000	4.006361000	-4.102639000
1	-1.138344000	3.666792000	-4.079975000
6	-0.187324000	3.529462000	-2.161419000

6	0.994333000	3.795460000	-1.466012000
6	-1.351962000	3.314382000	-1.432555000
6	1.004187000	3.803300000	-0.076390000
6	-1.352859000	3.337101000	-0.036624000
6	-0.158413000	3.563514000	0.658260000
1	-2.253247000	3.084884000	-1.989167000
1	1.922748000	3.978344000	0.470801000
6	-0.132252000	3.569789000	2.173848000
1	0.585856000	4.327926000	2.504105000
1	-1.118265000	3.869158000	2.536233000
8	-1.429920000	-3.154113000	-3.440754000
6	-2.639299000	-3.590444000	-2.849896000
1	-2.770388000	-3.099135000	-1.876014000
1	-2.600481000	-4.678283000	-2.688963000
8	-1.259780000	-4.279872000	1.454755000
6	-2.479680000	-3.729797000	1.911434000
1	-2.435985000	-2.632784000	1.876022000
1	-2.663350000	-4.031408000	2.954582000
8	-1.280032000	-0.546635000	4.711729000
6	-2.645677000	-0.487522000	4.365405000
1	-3.087052000	0.469311000	4.676231000
1	-2.748614000	-0.556771000	3.275747000
8	-2.471644000	3.139803000	0.721138000
6	-3.728440000	3.069082000	0.073882000
1	-3.707415000	2.293669000	-0.706056000
1	-4.412451000	2.734085000	0.854153000
8	-2.212609000	1.534079000	-4.128414000
6	-3.278905000	0.613462000	-4.001555000
1	-3.422280000	0.067381000	-4.946369000
1	-3.052036000	-0.122160000	-3.215764000
8	2.913732000	-0.490079000	-4.140058000
6	4.017665000	0.333560000	-3.835938000
1	4.117259000	1.133354000	-4.586081000
1	3.881590000	0.812335000	-2.852764000
8	2.092490000	4.058515000	-2.238328000
6	3.382608000	3.883483000	-1.698251000
1	3.579074000	4.609844000	-0.895556000
1	3.477619000	2.873990000	-1.265275000
8	2.506599000	2.681946000	2.363249000
6	3.838633000	2.661742000	2.846229000
1	3.838846000	2.805310000	3.936882000
1	4.329036000	1.705713000	2.623073000
8	3.563114000	-2.587103000	3.542640000
6	4.764881000	-3.033390000	2.958193000
1	4.810682000	-4.132566000	2.966883000
1	4.818420000	-2.703017000	1.908620000
8	3.359677000	-4.500725000	-1.044892000
6	4.571337000	-4.080969000	-1.631216000
1	4.678902000	-4.515214000	-2.636629000
1	4.577226000	-2.986026000	-1.738264000
6	-4.539199000	1.385782000	-3.652542000
1	-4.498978000	1.686117000	-2.597393000
1	-4.564918000	2.309516000	-4.243270000
6	-5.795831000	0.559765000	-3.918426000
1	-5.897913000	0.404882000	-5.001620000
1	-5.681471000	-0.435440000	-3.471760000
6	-7.067257000	1.203303000	-3.370601000
1	-7.051563000	1.145554000	-2.273630000
1	-7.085334000	2.274170000	-3.620226000
6	-8.342825000	0.543553000	-3.892343000
1	-8.416833000	0.716736000	-4.973853000
1	-8.263406000	-0.544575000	-3.767973000

6	-9.604137000	1.054163000	-3.199404000
1	-9.588497000	0.801387000	-2.132634000
1	-9.684829000	2.144335000	-3.281109000
1	-10.508023000	0.615720000	-3.635125000
6	-3.807509000	-3.248334000	-3.756728000
1	-4.002006000	-2.172614000	-3.707459000
1	-3.545580000	-3.490112000	-4.793568000
6	-5.050881000	-4.030250000	-3.321007000
1	-4.978491000	-5.060758000	-3.696443000
1	-5.061636000	-4.102760000	-2.225854000
6	-6.371162000	-3.404684000	-3.763414000
1	-6.494107000	-2.455878000	-3.228498000
1	-6.343015000	-3.167044000	-4.836365000
6	-7.570690000	-4.303551000	-3.464303000
1	-7.496180000	-5.222344000	-4.061093000
1	-7.524381000	-4.619803000	-2.413685000
6	-8.914703000	-3.629584000	-3.731260000
1	-9.054370000	-2.762703000	-3.073018000
1	-8.980959000	-3.267771000	-4.763752000
1	-9.751862000	-4.313432000	-3.556631000
6	-3.587301000	-4.245057000	1.003591000
1	-3.479977000	-3.788973000	0.012435000
1	-3.452790000	-5.326178000	0.876156000
6	-4.986362000	-3.966481000	1.552751000
1	-5.094656000	-4.457180000	2.531046000
1	-5.104681000	-2.890625000	1.739380000
6	-6.109454000	-4.433677000	0.624308000
1	-6.101894000	-3.829822000	-0.293053000
1	-5.916567000	-5.470217000	0.313282000
6	-7.493390000	-4.351915000	1.268693000
1	-7.491441000	-4.933519000	2.200590000
1	-7.696056000	-3.310723000	1.553931000
6	-8.616052000	-4.849267000	0.361377000
1	-8.709229000	-4.211140000	-0.523252000
1	-8.424745000	-5.872794000	0.019157000
1	-9.579871000	-4.842242000	0.881054000
6	-3.320868000	-1.650394000	5.070443000
1	-2.770552000	-2.568259000	4.827099000
1	-3.226532000	-1.512934000	6.154620000
6	-4.784497000	-1.823588000	4.682946000
1	-5.357407000	-0.927554000	4.957862000
1	-4.851588000	-1.907449000	3.590707000
6	-5.412988000	-3.063434000	5.316780000
1	-4.830998000	-3.948334000	5.019857000
1	-5.330080000	-2.998146000	6.410708000
6	-6.874918000	-3.273453000	4.928138000
1	-7.465466000	-2.402562000	5.243585000
1	-6.950675000	-3.319329000	3.834893000
6	-7.473933000	-4.547562000	5.518626000
1	-6.921704000	-5.431384000	5.177994000
1	-7.433820000	-4.533110000	6.613767000
1	-8.520500000	-4.671687000	5.221767000
6	-4.220077000	4.403514000	-0.474391000
1	-4.137004000	5.151218000	0.322593000
1	-3.580155000	4.742997000	-1.296142000
6	-5.669703000	4.303210000	-0.945058000
1	-5.777519000	3.452422000	-1.635210000
1	-6.308080000	4.093871000	-0.077840000
6	-6.171808000	5.563575000	-1.645232000
1	-6.068922000	6.422579000	-0.966896000
1	-5.533722000	5.779358000	-2.513830000
6	-7.626542000	5.446048000	-2.100860000

1	-7.724721000	4.578779000	-2.768695000
1	-8.260639000	5.233644000	-1.229684000
6	-8.133340000	6.699066000	-2.811916000
1	-8.073948000	7.574077000	-2.154351000
1	-7.533573000	6.913773000	-3.703915000
1	-9.175882000	6.587419000	-3.127611000
6	4.572937000	3.785597000	2.138017000
1	4.565329000	3.571274000	1.060734000
1	4.016492000	4.720487000	2.282582000
6	6.013677000	3.946621000	2.612260000
1	6.033698000	4.155472000	3.690709000
1	6.541305000	2.994298000	2.475435000
6	6.756140000	5.053960000	1.864741000
1	6.626737000	4.910156000	0.781713000
1	6.294439000	6.024660000	2.092766000
6	8.249369000	5.108029000	2.183771000
1	8.386608000	5.316050000	3.253051000
1	8.689186000	4.117839000	2.003733000
6	8.993870000	6.149009000	1.350708000
1	8.907101000	5.923760000	0.280901000
1	8.582996000	7.152035000	1.513181000
1	10.059019000	6.177800000	1.602080000
6	5.921580000	-2.462926000	3.754153000
1	5.835792000	-1.369842000	3.774230000
1	5.846913000	-2.817860000	4.789357000
6	7.258735000	-2.869104000	3.141124000
1	7.257086000	-3.946881000	2.921058000
1	7.362561000	-2.360146000	2.174799000
6	8.463864000	-2.545748000	4.021337000
1	8.413848000	-1.498396000	4.345124000
1	8.417919000	-3.150746000	4.937408000
6	9.795235000	-2.791381000	3.313099000
1	9.849818000	-3.843295000	3.001158000
1	9.823560000	-2.197219000	2.388937000
6	11.006496000	-2.445071000	4.175540000
1	10.990244000	-1.387748000	4.465379000
1	11.017507000	-3.040316000	5.095647000
1	11.944202000	-2.633259000	3.642521000
6	5.717122000	-4.518444000	-0.737965000
1	5.585825000	-4.070735000	0.255797000
1	5.675954000	-5.606415000	-0.606438000
6	7.064847000	-4.101631000	-1.322822000
1	7.176610000	-4.530743000	-2.328672000
1	7.076749000	-3.010389000	-1.453313000
6	8.261241000	-4.511794000	-0.466690000
1	8.170734000	-4.057680000	0.530454000
1	8.244043000	-5.599054000	-0.307253000
6	9.596828000	-4.109615000	-1.092145000
1	9.705621000	-4.611898000	-2.062649000
1	9.579538000	-3.032675000	-1.303358000
6	10.796890000	-4.431444000	-0.205178000
1	10.738825000	-3.884487000	0.742550000
1	10.838863000	-5.501277000	0.030027000
1	11.738263000	-4.158113000	-0.692923000
6	5.250007000	-0.555579000	-3.846412000
1	5.137166000	-1.324379000	-3.071033000
1	5.278741000	-1.080469000	-4.809210000
6	6.556889000	0.205712000	-3.633149000
1	6.611698000	1.056232000	-4.327610000
1	6.572429000	0.636236000	-2.622202000
6	7.789641000	-0.678348000	-3.823117000
1	7.730792000	-1.539674000	-3.141783000

1	7.778952000	-1.099532000	-4.837982000
6	9.109403000	0.058309000	-3.597124000
1	9.186289000	0.888744000	-4.311738000
1	9.105784000	0.513456000	-2.597541000
6	10.327287000	-0.852227000	-3.733320000
1	10.291235000	-1.666465000	-3.000453000
1	10.370304000	-1.305895000	-4.730093000
1	11.258864000	-0.299251000	-3.575716000
6	4.351922000	4.092637000	-2.848920000
1	4.058780000	3.429802000	-3.673059000
1	4.231880000	5.117838000	-3.219858000
6	5.807327000	3.828594000	-2.484400000
1	6.108187000	4.460400000	-1.636350000
1	5.909076000	2.790002000	-2.143968000
6	6.746121000	4.076093000	-3.665287000
1	6.421610000	3.458076000	-4.515460000
1	6.643406000	5.119716000	-3.994582000
6	8.213675000	3.781358000	-3.364096000
1	8.540855000	4.388482000	-2.509211000
1	8.313020000	2.736194000	-3.050684000
6	9.127408000	4.030822000	-4.561349000
1	8.834993000	3.403948000	-5.412408000
1	9.077648000	5.075899000	-4.887901000
1	10.171033000	3.802683000	-4.321127000
6	1.360619000	-1.167368000	0.285237000
1	1.328931000	-1.251225000	1.375515000
6	0.591143000	0.034831000	-0.156743000
1	0.759221000	0.976196000	0.363628000
1	0.883287000	-2.060577000	-0.126772000
6	-0.242060000	0.007029000	-1.206383000
1	-0.393667000	-0.906755000	-1.776707000
1	-0.713385000	0.908463000	-1.576174000
6	5.171151000	-0.245210000	0.156368000
1	5.477555000	-1.280760000	-0.070166000
1	5.252630000	0.327649000	-0.782169000
6	3.731232000	-0.234685000	0.650007000
1	3.730829000	-0.585577000	1.688978000
1	3.349092000	0.794378000	0.661424000
6	2.829322000	-1.152503000	-0.172293000
1	2.870853000	-0.901427000	-1.242133000
1	3.206820000	-2.177280000	-0.073988000
8	6.027725000	0.292753000	1.149202000
14	7.659989000	0.585937000	0.920040000
6	8.468775000	-0.827864000	-0.017264000
1	8.007565000	-0.947902000	-1.003899000
1	9.531121000	-0.608518000	-0.182716000
1	8.405849000	-1.790032000	0.497723000
6	8.375404000	0.796052000	2.643937000
1	8.596220000	1.843803000	2.873420000
1	7.662339000	0.435159000	3.392051000
1	9.303970000	0.225884000	2.761827000
6	7.929950000	2.126032000	-0.114658000
1	7.292929000	2.956452000	0.201247000
1	8.973737000	2.457746000	-0.051039000
1	7.717880000	1.930760000	-1.168869000
7	-6.467754000	-0.941335000	0.190557000
6	-5.200782000	-1.047836000	-0.331044000
6	-6.559179000	0.060111000	1.258546000
6	-5.068378000	0.222751000	1.583257000
7	-4.361338000	-0.407932000	0.601007000
8	-4.848294000	-1.599691000	-1.343354000
35	-2.501502000	-0.267486000	0.253630000

35	-7.908553000	-1.364313000	-0.875291000
1	-4.427064000	2.331895000	3.463006000
8	-5.674959000	4.028979000	2.538347000
6	-4.641529000	4.175275000	3.166342000
8	-4.005607000	3.165175000	3.758493000
6	-3.955046000	5.483125000	3.332929000
6	-2.751604000	5.594469000	4.032322000
6	-4.526808000	6.609543000	2.738682000
6	-2.121227000	6.829759000	4.127963000
1	-2.313399000	4.714092000	4.489558000
1	-5.462345000	6.497436000	2.199960000
1	-1.182203000	6.915661000	4.665783000
1	-2.193094000	8.914493000	3.602437000
6	-2.690266000	7.951615000	3.529863000
6	-3.894127000	7.842125000	2.836434000
1	-4.336213000	8.717205000	2.370244000
8	-4.618941000	0.832094000	2.529890000
6	-7.076517000	1.410720000	0.753906000
1	-8.142868000	1.338592000	0.519873000
1	-6.922336000	2.176908000	1.519665000
1	-6.543048000	1.717038000	-0.150154000
6	-7.354907000	-0.448970000	2.450013000
1	-6.969460000	-1.407904000	2.799070000
1	-7.298272000	0.275447000	3.266122000
1	-8.403233000	-0.575927000	2.162298000

IM1^{PA5}

E_1 = -11199.4125307 A.U.

Zero-point correction= 2.855453 (Hartree/Particle)

Thermal correction to Energy= 3.010270

Thermal correction to Enthalpy= 3.011214

Thermal correction to Gibbs Free Energy= 2.651482

E_2 = -11205.8033667 A.U.

At No.	X	Y	Z
6	0.797064000	1.388110000	3.735175000
6	-0.513937000	1.760424000	3.404310000
6	1.856298000	1.987967000	3.058900000
6	-0.743069000	2.748377000	2.446110000
6	1.623893000	2.938284000	2.068257000
6	0.319471000	3.343931000	1.767057000
1	2.866575000	1.679270000	3.308185000
1	-1.751621000	3.071094000	2.208206000
6	1.057867000	0.299454000	4.754971000
1	2.023818000	0.472067000	5.234560000
1	0.285258000	0.332188000	5.527848000
6	1.049837000	-1.064350000	4.096804000
6	-0.164637000	-1.672360000	3.782373000
6	2.235816000	-1.699205000	3.715866000
6	-0.215226000	-2.884927000	3.101760000
6	2.185603000	-2.925622000	3.053392000
6	0.971712000	-3.536518000	2.751253000
1	-1.069809000	-1.146047000	4.055845000
1	3.089515000	-3.424989000	2.726471000
6	0.930505000	-4.840372000	1.986034000
1	0.044033000	-5.407853000	2.279703000
1	1.811609000	-5.437522000	2.233605000
6	0.889983000	-4.583806000	0.496451000
6	-0.327056000	-4.424495000	-0.161892000
6	2.074386000	-4.427597000	-0.230335000
6	-0.372343000	-4.128403000	-1.522219000
6	2.030340000	-4.150086000	-1.594678000

6	0.813462000	-4.003784000	-2.256979000
1	-1.236342000	-4.510731000	0.421660000
1	2.940444000	-4.026720000	-2.171100000
6	0.760674000	-3.704275000	-3.739216000
1	-0.060587000	-4.268935000	-4.189991000
1	1.691461000	-4.035840000	-4.206500000
6	0.552984000	-2.231239000	-4.013387000
6	1.644196000	-1.357713000	-4.077328000
6	-0.729959000	-1.708475000	-4.152372000
6	1.441086000	-0.003203000	-4.324572000
6	-0.936706000	-0.342612000	-4.336672000
6	0.155686000	0.521072000	-4.458725000
1	-1.565255000	-2.390855000	-4.058756000
1	2.273686000	0.686955000	-4.389795000
6	-0.054466000	2.011019000	-4.620507000
1	0.728615000	2.423993000	-5.260214000
1	-1.017165000	2.197387000	-5.101272000
6	-0.022047000	2.685040000	-3.266391000
6	1.185121000	3.132888000	-2.724708000
6	-1.184114000	2.804534000	-2.507088000
6	1.215900000	3.688045000	-1.448217000
6	-1.157919000	3.351917000	-1.223741000
6	0.054440000	3.807709000	-0.685467000
1	-2.110965000	2.453580000	-2.945740000
1	2.146965000	4.032696000	-1.013654000
6	0.085733000	4.397912000	0.707128000
1	0.880424000	5.146380000	0.762925000
1	-0.860277000	4.902857000	0.904172000
8	-1.531854000	-3.936139000	-2.212315000
6	-2.761914000	-4.302424000	-1.599203000
1	-2.980136000	-3.635486000	-0.756500000
1	-2.690904000	-5.334892000	-1.227646000
8	-1.376761000	-3.496177000	2.735264000
6	-2.590334000	-2.808192000	2.987738000
1	-2.580559000	-1.842851000	2.462228000
1	-2.687057000	-2.610765000	4.065283000
8	-1.502619000	1.096862000	4.066598000
6	-2.856688000	1.499894000	3.910041000
1	-2.989414000	2.514331000	4.307014000
1	-3.142136000	1.519661000	2.850811000
8	-2.247826000	3.479642000	-0.427601000
6	-3.494658000	2.940674000	-0.843096000
1	-3.355890000	1.926800000	-1.244047000
1	-4.059038000	2.833428000	0.081936000
8	-2.174685000	0.237720000	-4.352910000
6	-3.303018000	-0.564430000	-4.041317000
1	-3.396210000	-1.383380000	-4.768742000
1	-3.188986000	-1.010974000	-3.041940000
8	2.873322000	-1.921536000	-3.878284000
6	4.019984000	-1.099520000	-3.891485000
1	4.147636000	-0.633059000	-4.880287000
1	3.920340000	-0.289349000	-3.152553000
8	2.283847000	3.017127000	-3.531863000
6	3.572709000	3.034656000	-2.957999000
1	3.792803000	4.011899000	-2.504381000
1	3.640411000	2.276193000	-2.160608000
8	2.608529000	3.507947000	1.312468000
6	3.949651000	3.509244000	1.764282000
1	4.015701000	4.029540000	2.731399000
1	4.330012000	2.487919000	1.887204000
8	3.405843000	-1.054505000	4.021996000
6	4.619827000	-1.716436000	3.741089000

1	4.571330000	-2.763095000	4.072043000
1	4.803350000	-1.715927000	2.653269000
8	3.232927000	-4.543335000	0.489182000
6	4.472422000	-4.459377000	-0.176991000
1	4.548771000	-5.243760000	-0.944817000
1	4.568867000	-3.485577000	-0.682703000
6	-4.535365000	0.317759000	-4.090055000
1	-4.444044000	1.112217000	-3.337783000
1	-4.573453000	0.809553000	-5.069999000
6	-5.813817000	-0.486938000	-3.857270000
1	-5.825841000	-1.352878000	-4.535748000
1	-5.820974000	-0.899579000	-2.839273000
6	-7.081576000	0.335115000	-4.085866000
1	-7.083620000	1.196554000	-3.405963000
1	-7.067906000	0.755045000	-5.102000000
6	-8.363564000	-0.473420000	-3.887790000
1	-8.379198000	-1.300701000	-4.611011000
1	-8.343191000	-0.936222000	-2.893662000
6	-9.629164000	0.367000000	-4.038535000
1	-9.668774000	1.156287000	-3.279232000
1	-9.667429000	0.852480000	-5.020752000
1	-10.530348000	-0.245676000	-3.931007000
6	-3.851909000	-4.178965000	-2.646035000
1	-3.983246000	-3.119249000	-2.893901000
1	-3.531099000	-4.695942000	-3.559432000
6	-5.178045000	-4.745856000	-2.148070000
1	-5.082264000	-5.827899000	-1.981885000
1	-5.408121000	-4.290281000	-1.178321000
6	-6.330729000	-4.468522000	-3.111315000
1	-6.431069000	-3.381368000	-3.234409000
1	-6.089387000	-4.866626000	-4.107228000
6	-7.668155000	-5.048064000	-2.647080000
1	-7.609327000	-6.144199000	-2.644146000
1	-7.850623000	-4.758188000	-1.602686000
6	-8.840747000	-4.589537000	-3.511822000
1	-8.953575000	-3.499629000	-3.465971000
1	-8.686085000	-4.862037000	-4.562286000
1	-9.783459000	-5.038553000	-3.182742000
6	-3.743378000	-3.668977000	2.511162000
1	-3.783842000	-3.656954000	1.415887000
1	-3.561462000	-4.703294000	2.828178000
6	-5.074281000	-3.180483000	3.079346000
1	-5.053950000	-3.261685000	4.176021000
1	-5.203948000	-2.113701000	2.848428000
6	-6.268794000	-3.948474000	2.522221000
1	-6.335567000	-3.749762000	1.447172000
1	-6.103374000	-5.029706000	2.633336000
6	-7.591377000	-3.562967000	3.183668000
1	-7.597516000	-3.912606000	4.224985000
1	-7.658262000	-2.470103000	3.227837000
6	-8.808573000	-4.103357000	2.435978000
1	-8.844802000	-3.680802000	1.425592000
1	-8.765874000	-5.195031000	2.342921000
1	-9.742396000	-3.845098000	2.946746000
6	-3.709710000	0.487732000	4.654404000
1	-3.737721000	-0.441576000	4.071843000
1	-3.222822000	0.253780000	5.609744000
6	-5.134874000	0.978530000	4.904394000
1	-5.109249000	1.822906000	5.607348000
1	-5.564398000	1.368873000	3.974220000
6	-6.037893000	-0.122557000	5.458360000
1	-6.095307000	-0.941352000	4.726447000

1	-5.582890000	-0.559971000	6.359086000
6	-7.449732000	0.360695000	5.788995000
1	-7.398994000	1.095329000	6.603422000
1	-7.860845000	0.895123000	4.922991000
6	-8.387420000	-0.780847000	6.176904000
1	-8.490007000	-1.497931000	5.353647000
1	-8.003126000	-1.328593000	7.045469000
1	-9.387487000	-0.412214000	6.427952000
6	-4.268337000	3.820184000	-1.813589000
1	-4.164281000	4.862551000	-1.497187000
1	-3.869435000	3.739662000	-2.833076000
6	-5.740966000	3.419163000	-1.779936000
1	-5.814065000	2.323740000	-1.837821000
1	-6.147620000	3.703908000	-0.802017000
6	-6.577846000	4.023490000	-2.903752000
1	-6.370356000	5.099395000	-2.984317000
1	-6.272287000	3.577865000	-3.862339000
6	-8.078935000	3.813934000	-2.697513000
1	-8.265369000	2.764506000	-2.434806000
1	-8.405488000	4.403131000	-1.830953000
6	-8.910519000	4.178068000	-3.924380000
1	-8.749819000	5.222928000	-4.214498000
1	-8.635485000	3.548197000	-4.778910000
1	-9.981261000	4.040695000	-3.739688000
6	4.746568000	4.220825000	0.685380000
1	4.690625000	3.612062000	-0.226640000
1	4.262334000	5.180987000	0.465936000
6	6.208516000	4.438387000	1.054402000
1	6.282344000	5.066840000	1.952626000
1	6.653783000	3.471719000	1.315861000
6	7.009888000	5.070016000	-0.082307000
1	6.868667000	4.469438000	-0.993284000
1	6.603380000	6.064047000	-0.314759000
6	8.504438000	5.186451000	0.216094000
1	8.653970000	5.868036000	1.063455000
1	8.885917000	4.208125000	0.539737000
6	9.309444000	5.668157000	-0.988877000
1	9.213374000	4.965336000	-1.825576000
1	8.955245000	6.646036000	-1.334490000
1	10.373739000	5.761448000	-0.750379000
6	5.740348000	-1.008499000	4.478446000
1	5.734555000	0.053585000	4.205786000
1	5.539139000	-1.061385000	5.555204000
6	7.095277000	-1.636611000	4.157524000
1	7.040976000	-2.725224000	4.304464000
1	7.318028000	-1.484226000	3.093982000
6	8.251126000	-1.079738000	4.985114000
1	8.264812000	0.015821000	4.906911000
1	8.089038000	-1.310321000	6.046897000
6	9.604167000	-1.634965000	4.540632000
1	9.590874000	-2.730226000	4.623821000
1	9.746973000	-1.414276000	3.472522000
6	10.777219000	-1.070931000	5.338588000
1	10.830341000	0.019365000	5.238955000
1	10.673792000	-1.301971000	6.404786000
1	11.730323000	-1.486746000	4.996253000
6	5.561575000	-4.613834000	0.870244000
1	5.498930000	-3.765369000	1.565779000
1	5.358963000	-5.517079000	1.457785000
6	6.958654000	-4.682512000	0.257560000
1	7.052259000	-5.593785000	-0.348493000
1	7.094651000	-3.840052000	-0.433345000

6	8.070362000	-4.646725000	1.305775000
1	7.924396000	-3.776259000	1.961079000
1	7.994380000	-5.531548000	1.953018000
6	9.468191000	-4.576315000	0.693139000
1	9.633716000	-5.455094000	0.055860000
1	9.519004000	-3.701961000	0.029901000
6	10.572681000	-4.482018000	1.743375000
1	10.447091000	-3.586067000	2.362606000
1	10.556125000	-5.350762000	2.411419000
1	11.563300000	-4.434267000	1.279874000
6	5.200799000	-1.995153000	-3.561925000
1	5.016672000	-2.463035000	-2.586212000
1	5.228647000	-2.806943000	-4.299112000
6	6.544456000	-1.270293000	-3.535452000
1	6.678912000	-0.687926000	-4.457752000
1	6.553114000	-0.543820000	-2.710530000
6	7.714303000	-2.242469000	-3.376743000
1	7.547958000	-2.869677000	-2.488455000
1	7.723479000	-2.933501000	-4.230938000
6	9.077581000	-1.562450000	-3.263573000
1	9.255911000	-0.955690000	-4.161380000
1	9.067358000	-0.863452000	-2.416559000
6	10.218930000	-2.560956000	-3.084054000
1	10.073303000	-3.164428000	-2.180525000
1	10.277162000	-3.248906000	-3.935148000
1	11.184399000	-2.052706000	-2.996694000
6	4.544504000	2.744406000	-4.088561000
1	4.221306000	1.826500000	-4.595942000
1	4.463524000	3.549698000	-4.828654000
6	5.993103000	2.584320000	-3.640298000
1	6.323056000	3.476738000	-3.089962000
1	6.063820000	1.745667000	-2.934434000
6	6.925359000	2.341057000	-4.828092000
1	6.552680000	1.481805000	-5.405167000
1	6.873347000	3.203916000	-5.506753000
6	8.380273000	2.088276000	-4.440641000
1	8.753548000	2.932334000	-3.845185000
1	8.431333000	1.209213000	-3.788288000
6	9.284894000	1.862263000	-5.649504000
1	8.946926000	0.997679000	-6.233531000
1	9.282584000	2.732703000	-6.315355000
1	10.319514000	1.675705000	-5.343792000
6	0.978022000	-0.816261000	0.491705000
1	1.061690000	-0.478174000	1.528011000
6	0.081864000	0.117125000	-0.262636000
1	0.413988000	1.142644000	-0.427091000
1	0.528497000	-1.813369000	0.505544000
6	-0.954257000	-0.387430000	-1.152179000
1	-1.105368000	-1.459667000	-1.254474000
1	-1.309416000	0.246885000	-1.956842000
6	4.733119000	0.069647000	-0.194365000
1	5.023212000	-0.993517000	-0.178725000
1	4.755944000	0.393139000	-1.246579000
6	3.327397000	0.210232000	0.376022000
1	3.400256000	0.125684000	1.467222000
1	2.937232000	1.214054000	0.162084000
6	2.387591000	-0.885529000	-0.130557000
1	2.326335000	-0.895031000	-1.228007000
1	2.800576000	-1.858123000	0.159309000
8	5.645552000	0.840768000	0.564143000
14	7.303822000	0.590706000	0.578091000
6	7.636906000	-1.245273000	0.363012000

1	7.484520000	-1.555131000	-0.676105000
1	8.671307000	-1.490839000	0.631206000
1	6.979546000	-1.857683000	0.988789000
6	7.952115000	1.219547000	2.222999000
1	8.263344000	2.267656000	2.169540000
1	7.196328000	1.135049000	3.008983000
1	8.825105000	0.633957000	2.534817000
6	8.149293000	1.511564000	-0.816428000
1	7.870036000	2.568920000	-0.835915000
1	9.239699000	1.453756000	-0.709395000
1	7.893557000	1.085122000	-1.789004000
7	-6.346369000	-1.053195000	0.508458000
6	-4.969456000	-0.820862000	0.318933000
6	-7.012863000	0.197164000	0.877815000
6	-5.759056000	1.015616000	1.238784000
7	-4.627889000	0.364113000	0.929585000
8	-4.216096000	-1.588521000	-0.266743000
35	-1.816340000	0.257092000	0.529454000
35	-7.204398000	-2.237890000	-0.633238000
1	-4.811952000	3.329530000	2.163126000
8	-4.940712000	5.319744000	0.884055000
6	-4.114132000	5.075544000	1.739933000
8	-4.101889000	3.964028000	2.468923000
6	-2.974803000	5.987034000	2.059785000
6	-2.154327000	5.783309000	3.170534000
6	-2.706358000	7.037131000	1.180734000
6	-1.062425000	6.615653000	3.389969000
1	-2.362552000	4.962968000	3.847656000
1	-3.358346000	7.177558000	0.325077000
1	-0.420003000	6.446774000	4.248644000
1	0.072376000	8.295226000	2.668193000
6	-0.786912000	7.651944000	2.501369000
6	-1.611684000	7.864636000	1.398218000
1	-1.397076000	8.672506000	0.705167000
8	-5.880993000	2.138571000	1.747305000
6	-7.762884000	0.856284000	-0.280078000
1	-8.707303000	0.338847000	-0.476612000
1	-7.974234000	1.897502000	-0.022957000
1	-7.164358000	0.837772000	-1.194325000
6	-7.918321000	0.022666000	2.089876000
1	-7.353601000	-0.363009000	2.942071000
1	-8.347944000	0.988430000	2.368850000
1	-8.728516000	-0.677624000	1.859229000

D

E_1 = -5181.26403275A.U.

Zero-point correction= 2.616441 (Hartree/Particle)
Thermal correction to Energy= 2.750121
Thermal correction to Enthalpy= 2.751065
Thermal correction to Gibbs Free Energy= 2.438797

E_2 = -5182.56189888 A.U.

At No.	X	Y	Z
6	-0.042886000	1.904542000	3.566432000
6	1.267041000	1.450281000	3.766570000
6	-1.086150000	0.984739000	3.675449000
6	1.506386000	0.100673000	4.022687000
6	-0.847142000	-0.353077000	3.984309000
6	0.464875000	-0.812808000	4.139803000
1	-2.098359000	1.342417000	3.514220000
1	2.514999000	-0.279473000	4.134354000
6	-0.344558000	3.350604000	3.220164000

1	-1.352440000	3.600139000	3.558909000
1	0.353209000	4.002739000	3.755472000
6	-0.227939000	3.617461000	1.731813000
6	1.033692000	3.587623000	1.142235000
6	-1.335620000	3.869080000	0.918020000
6	1.214346000	3.779932000	-0.221674000
6	-1.156084000	4.049862000	-0.455165000
6	0.106731000	4.022862000	-1.038754000
1	1.873114000	3.402050000	1.797098000
1	-2.001263000	4.197764000	-1.116031000
6	0.269031000	4.199973000	-2.531362000
1	1.207870000	4.721133000	-2.738138000
1	-0.548393000	4.814433000	-2.917087000
6	0.276970000	2.857751000	-3.224527000
6	1.475599000	2.187302000	-3.433869000
6	-0.918080000	2.237053000	-3.597598000
6	1.503097000	0.918083000	-4.004271000
6	-0.895389000	0.959616000	-4.152804000
6	0.308148000	0.284001000	-4.357181000
1	2.382687000	2.677913000	-3.105528000
1	-1.813466000	0.445693000	-4.414763000
6	0.319875000	-1.126283000	-4.902681000
1	1.175148000	-1.251458000	-5.573043000
1	-0.588338000	-1.293473000	-5.488038000
6	0.404454000	-2.160986000	-3.800282000
6	-0.751552000	-2.636897000	-3.172024000
6	1.639110000	-2.643764000	-3.376104000
6	-0.656888000	-3.623339000	-2.193194000
6	1.737772000	-3.595373000	-2.363649000
6	0.579964000	-4.122226000	-1.786637000
1	2.524394000	-2.236231000	-3.848268000
1	-1.540684000	-4.010134000	-1.698997000
6	0.667458000	-5.153453000	-0.682411000
1	-0.193221000	-5.824159000	-0.742276000
1	1.571646000	-5.753487000	-0.810656000
6	0.696484000	-4.476684000	0.669155000
6	-0.489384000	-4.196126000	1.351052000
6	1.898101000	-4.021849000	1.204762000
6	-0.445364000	-3.543545000	2.578703000
6	1.932743000	-3.284873000	2.387381000
6	0.754319000	-3.073430000	3.108212000
1	2.799860000	-4.214470000	0.636400000
1	-1.350944000	-3.337094000	3.133416000
6	0.751873000	-2.276613000	4.395675000
1	-0.007830000	-2.684796000	5.068630000
1	1.721476000	-2.372567000	4.890266000
8	2.665897000	0.228665000	-4.238030000
6	3.856742000	0.796184000	-3.735905000
1	3.770575000	0.921863000	-2.647095000
1	4.017849000	1.793885000	-4.170129000
8	2.442875000	3.750298000	-0.823256000
6	3.547927000	3.506515000	0.019315000
1	3.387149000	2.579997000	0.586524000
1	3.651644000	4.331885000	0.741015000
8	2.269793000	2.385261000	3.713179000
6	3.600060000	1.926272000	3.825746000
1	3.737342000	1.389075000	4.775530000
1	3.822136000	1.221881000	3.009657000
8	3.078278000	-2.725559000	2.889549000
6	4.198653000	-2.696574000	2.029262000
1	4.608483000	-3.711373000	1.906796000
1	3.895108000	-2.332249000	1.038011000

8	2.932562000	-4.071924000	-1.892583000
6	4.096388000	-3.343610000	-2.218947000
1	4.273692000	-3.374891000	-3.304908000
1	3.970171000	-2.290281000	-1.924884000
8	-1.933382000	-2.094208000	-3.591690000
6	-3.121379000	-2.480665000	-2.933611000
1	-3.275975000	-3.566288000	-3.026894000
1	-3.061894000	-2.239969000	-1.862254000
8	-1.651362000	-4.583710000	0.742452000
6	-2.869846000	-4.194586000	1.341021000
1	-2.988067000	-4.703152000	2.310992000
1	-2.878465000	-3.109974000	1.519896000
8	-1.827210000	-1.285352000	4.163303000
6	-3.181094000	-0.905181000	4.036192000
1	-3.403369000	-0.059853000	4.705468000
1	-3.387771000	-0.591859000	3.003279000
8	-2.561869000	3.949652000	1.529951000
6	-3.659278000	4.349104000	0.735255000
1	-3.383283000	5.199000000	0.096918000
1	-3.950627000	3.519056000	0.071770000
8	-2.056817000	2.961023000	-3.378317000
6	-3.297737000	2.414438000	-3.758577000
1	-3.295533000	2.158660000	-4.828875000
1	-3.494070000	1.489354000	-3.194135000
6	5.280527000	-3.964161000	-1.500960000
1	5.124072000	-3.905256000	-0.417172000
1	5.331423000	-5.028668000	-1.760162000
6	6.583981000	-3.267162000	-1.885760000
1	6.679346000	-3.267921000	-2.981284000
1	6.540629000	-2.210498000	-1.582483000
6	7.831682000	-3.910404000	-1.284409000
1	7.775861000	-3.870692000	-0.187578000
1	7.859512000	-4.976445000	-1.549503000
6	9.119286000	-3.235522000	-1.753607000
1	9.197321000	-3.337027000	-2.844628000
1	9.053100000	-2.158674000	-1.551668000
6	10.375316000	-3.798787000	-1.094313000
1	10.340312000	-3.667935000	-0.006086000
1	10.480389000	-4.871020000	-1.295426000
1	11.276292000	-3.296940000	-1.462110000
6	5.040030000	-0.092295000	-4.073363000
1	4.823425000	-1.125394000	-3.780325000
1	5.196170000	-0.092533000	-5.158888000
6	6.291230000	0.409202000	-3.349169000
1	6.346724000	1.504473000	-3.436514000
1	6.189393000	0.198589000	-2.273600000
6	7.602176000	-0.184130000	-3.860977000
1	7.567623000	-1.279697000	-3.796216000
1	7.717437000	0.059591000	-4.926321000
6	8.813475000	0.333165000	-3.086221000
1	8.810223000	1.431508000	-3.101822000
1	8.707214000	0.046821000	-2.030267000
6	10.147833000	-0.177231000	-3.623150000
1	10.190177000	-1.271578000	-3.602497000
1	10.300651000	0.142476000	-4.660220000
1	10.985424000	0.202362000	-3.028269000
6	4.814556000	3.377240000	-0.802639000
1	4.700586000	2.554946000	-1.519063000
1	4.977400000	4.292904000	-1.383866000
6	6.001109000	3.114766000	0.123571000
1	6.074467000	3.940017000	0.846229000
1	5.802506000	2.209799000	0.717738000

6	7.344357000	2.957309000	-0.583660000
1	7.326807000	2.056789000	-1.210649000
1	7.507999000	3.805075000	-1.263855000
6	8.502404000	2.868611000	0.408694000
1	8.529788000	3.788781000	1.007732000
1	8.297777000	2.054054000	1.116102000
6	9.861336000	2.646861000	-0.248641000
1	9.891024000	1.689085000	-0.779813000
1	10.079917000	3.437768000	-0.975550000
1	10.665037000	2.640920000	0.494772000
6	4.550581000	3.107245000	3.763085000
1	4.406599000	3.642406000	2.816707000
1	4.305488000	3.809173000	4.569066000
6	6.000595000	2.638684000	3.887225000
1	6.117736000	2.059710000	4.814433000
1	6.226943000	1.945782000	3.064314000
6	7.024390000	3.771661000	3.873546000
1	6.957273000	4.313019000	2.919691000
1	6.778149000	4.501036000	4.657839000
6	8.455815000	3.273682000	4.074987000
1	8.533983000	2.790788000	5.058024000
1	8.672691000	2.493294000	3.333888000
6	9.497387000	4.384308000	3.963119000
1	9.466671000	4.853172000	2.972508000
1	9.317839000	5.169470000	4.706551000
1	10.510260000	3.999172000	4.119235000
6	5.262581000	-1.781300000	2.607156000
1	4.814322000	-0.807339000	2.839931000
1	5.633304000	-2.195981000	3.552575000
6	6.409423000	-1.611078000	1.610051000
1	6.714360000	-2.602741000	1.246818000
1	6.043465000	-1.066161000	0.727673000
6	7.639523000	-0.906624000	2.177384000
1	7.368194000	0.097618000	2.533131000
1	7.994694000	-1.456646000	3.060120000
6	8.775269000	-0.801342000	1.159810000
1	8.972402000	-1.798108000	0.744260000
1	8.447864000	-0.178042000	0.315119000
6	10.066286000	-0.240755000	1.750181000
1	9.908522000	0.754646000	2.181320000
1	10.442814000	-0.890264000	2.548556000
1	10.847845000	-0.154035000	0.988543000
6	-4.026690000	-2.112259000	4.407887000
1	-3.846633000	-2.908118000	3.675240000
1	-3.690264000	-2.492187000	5.379949000
6	-5.517170000	-1.784046000	4.454770000
1	-5.714376000	-1.072115000	5.268564000
1	-5.800161000	-1.270538000	3.528862000
6	-6.405605000	-3.015483000	4.623779000
1	-6.185101000	-3.731922000	3.820570000
1	-6.155548000	-3.523774000	5.565502000
6	-7.898143000	-2.685618000	4.600757000
1	-8.126355000	-1.972967000	5.404559000
1	-8.138863000	-2.171753000	3.659594000
6	-8.780375000	-3.923884000	4.743189000
1	-8.591197000	-4.632842000	3.929094000
1	-8.581415000	-4.441637000	5.688549000
1	-9.843440000	-3.662995000	4.720397000
6	-4.819806000	4.743125000	1.633295000
1	-4.891740000	4.032122000	2.462015000
1	-4.622423000	5.725753000	2.077958000
6	-6.130934000	4.759922000	0.848579000

1	-6.057758000	5.470636000	0.012691000
1	-6.281059000	3.771140000	0.391570000
6	-7.351619000	5.099943000	1.700089000
1	-7.393095000	4.418811000	2.562523000
1	-7.243575000	6.111852000	2.114403000
6	-8.661251000	5.002699000	0.917891000
1	-8.609782000	5.664239000	0.042530000
1	-8.764883000	3.981658000	0.524069000
6	-9.889395000	5.353299000	1.754818000
1	-9.978918000	4.685859000	2.619628000
1	-9.826306000	6.379921000	2.133189000
1	-10.809762000	5.267991000	1.168102000
6	-4.370207000	3.445519000	-3.454537000
1	-4.348965000	3.659380000	-2.377504000
1	-4.121136000	4.383814000	-3.963717000
6	-5.760159000	2.959711000	-3.864208000
1	-5.845975000	2.960152000	-4.959086000
1	-5.881788000	1.915216000	-3.550739000
6	-6.896293000	3.782015000	-3.257690000
1	-6.736078000	3.875715000	-2.173921000
1	-6.873337000	4.804104000	-3.660838000
6	-8.269333000	3.157587000	-3.503758000
1	-8.418078000	3.014925000	-4.582602000
1	-8.281817000	2.153399000	-3.056868000
6	-9.417249000	3.985406000	-2.930853000
1	-9.303835000	4.116348000	-1.848795000
1	-9.446641000	4.983159000	-3.383672000
1	-10.384958000	3.506253000	-3.111664000
6	-4.272074000	-1.732199000	-3.578310000
1	-4.076420000	-0.654243000	-3.505189000
1	-4.302908000	-1.977300000	-4.647191000
6	-5.610102000	-2.056063000	-2.918682000
1	-5.770874000	-3.143518000	-2.915498000
1	-5.567149000	-1.753270000	-1.863838000
6	-6.791185000	-1.379138000	-3.614885000
1	-6.575757000	-0.309822000	-3.757652000
1	-6.900276000	-1.798552000	-4.624664000
6	-8.108603000	-1.524809000	-2.855082000
1	-8.284077000	-2.587121000	-2.636611000
1	-8.018774000	-1.020930000	-1.883555000
6	-9.302420000	-0.953657000	-3.617140000
1	-9.152945000	0.108704000	-3.842563000
1	-9.446480000	-1.476415000	-4.569561000
1	-10.227605000	-1.047383000	-3.039339000
6	-4.008129000	-4.568520000	0.410514000
1	-3.963863000	-3.933277000	-0.482875000
1	-3.880304000	-5.606791000	0.081261000
6	-5.354175000	-4.393887000	1.108179000
1	-5.420103000	-5.101342000	1.947518000
1	-5.397674000	-3.388073000	1.547004000
6	-6.557910000	-4.588115000	0.190079000
1	-6.540418000	-3.819321000	-0.595232000
1	-6.476636000	-5.553202000	-0.329735000
6	-7.888864000	-4.527965000	0.940701000
1	-7.954156000	-5.388404000	1.620262000
1	-7.906549000	-3.634147000	1.578000000
6	-9.100253000	-4.508090000	0.012132000
1	-9.090330000	-3.611039000	-0.618222000
1	-9.105330000	-5.381289000	-0.650601000
1	-10.038030000	-4.509360000	0.577227000
6	0.905977000	-0.782541000	0.116099000
1	0.771085000	-1.844708000	-0.127422000

6	2.154140000	-0.314118000	-0.565165000
1	2.124616000	-0.318720000	-1.655076000
1	1.022804000	-0.720875000	1.205093000
6	3.270168000	0.056613000	0.062384000
1	3.320918000	0.065749000	1.149968000
1	4.167278000	0.360953000	-0.473986000
6	-2.874957000	0.045865000	-0.089902000
1	-2.818095000	1.058819000	0.340451000
1	-2.958900000	0.160172000	-1.182012000
6	-1.606231000	-0.710546000	0.252439000
1	-1.522604000	-0.797446000	1.342984000
1	-1.674608000	-1.734683000	-0.138825000
6	-0.357707000	-0.031502000	-0.314325000
1	-0.417154000	-0.006049000	-1.411533000
1	-0.303007000	1.011618000	0.025573000
8	-4.024130000	-0.649860000	0.398455000
14	-5.455030000	0.192728000	0.614597000
6	-5.820043000	1.192123000	-0.930836000
1	-5.848502000	0.535489000	-1.806007000
1	-6.790675000	1.698379000	-0.863849000
1	-5.058690000	1.956508000	-1.116590000
6	-5.309003000	1.343943000	2.090881000
1	-6.130344000	2.070352000	2.093025000
1	-5.342814000	0.806720000	3.043175000
1	-4.366690000	1.903280000	2.058437000
6	-6.847321000	-1.034115000	0.862859000
1	-6.929183000	-1.727048000	0.020392000
1	-6.731373000	-1.630782000	1.770596000
1	-7.798969000	-0.494064000	0.943154000

D’

E_1 = -4882.29725960 A.U.
Zero-point correction= 2.455287 (Hartree/Particle)
Thermal correction to Energy= 2.580326
Thermal correction to Enthalpy= 2.581270
Thermal correction to Gibbs Free Energy= 2.286978
E_2 = -4883.57324569 A.U.

At No.	X	Y	Z
6	-0.006656000	-4.444366000	-0.968998000
6	-1.267904000	-4.363199000	-0.366307000
6	1.120703000	-4.315396000	-0.164443000
6	-1.375952000	-4.139545000	1.000653000
6	1.013359000	-4.081056000	1.205938000
6	-0.245940000	-3.988132000	1.800907000
1	2.089259000	-4.355081000	-0.652011000
1	-2.343538000	-4.045584000	1.479792000
6	0.129686000	-4.548717000	-2.474321000
1	1.079882000	-5.025226000	-2.725839000
1	-0.675245000	-5.168304000	-2.879321000
6	0.062022000	-3.167771000	-3.095490000
6	-1.163911000	-2.556618000	-3.307889000
6	1.220573000	-2.434353000	-3.389349000
6	-1.262709000	-1.247095000	-3.765689000
6	1.123525000	-1.149823000	-3.919708000
6	-0.118164000	-0.536868000	-4.115893000
1	-2.065903000	-3.114167000	-3.083247000
1	2.010667000	-0.579467000	-4.169396000
6	-0.214810000	0.842228000	-4.735446000
1	-1.101029000	0.877044000	-5.375242000
1	0.658663000	0.999242000	-5.375228000
6	-0.299186000	1.973655000	-3.735509000

6	-1.535351000	2.490700000	-3.349711000
6	0.861151000	2.535064000	-3.191834000
6	-1.630313000	3.537869000	-2.435703000
6	0.764768000	3.601847000	-2.299813000
6	-0.470123000	4.102232000	-1.898125000
1	-2.425131000	2.043741000	-3.781096000
1	1.647936000	4.040429000	-1.851909000
6	-0.540226000	5.145746000	-0.802741000
1	-1.441038000	5.753039000	-0.917254000
1	0.325762000	5.808414000	-0.867571000
6	-0.559016000	4.450851000	0.543880000
6	0.622033000	4.201634000	1.249261000
6	-1.758783000	3.949899000	1.050188000
6	0.579668000	3.462334000	2.432491000
6	-1.798226000	3.208233000	2.226580000
6	-0.614867000	2.952568000	2.930718000
1	-2.656743000	4.134927000	0.472969000
1	1.486100000	3.233319000	2.980351000
6	-0.617289000	2.112663000	4.189004000
1	0.238336000	2.399036000	4.806828000
1	-1.524207000	2.323379000	4.763348000
6	-0.552987000	0.625150000	3.907896000
6	0.669853000	-0.043950000	3.800567000
6	-1.726359000	-0.107276000	3.742732000
6	0.688596000	-1.426448000	3.630095000
6	-1.703329000	-1.478898000	3.498962000
6	-0.484237000	-2.160488000	3.487609000
1	-2.661469000	0.436639000	3.794406000
1	1.621097000	-1.972845000	3.568212000
6	-0.408835000	-3.656574000	3.266796000
1	0.438310000	-4.057092000	3.830199000
1	-1.317981000	-4.130849000	3.645940000
8	-2.813714000	4.085588000	-2.019660000
6	-4.009003000	3.365553000	-2.224730000
1	-3.918756000	2.356385000	-1.793950000
1	-4.218568000	3.251828000	-3.298738000
8	-2.507441000	-0.665800000	-3.904432000
6	-3.226810000	-0.543457000	-2.683856000
1	-2.801188000	0.276467000	-2.085834000
1	-3.125626000	-1.461134000	-2.089211000
8	-2.352092000	-4.507243000	-1.189317000
6	-3.625577000	-4.203584000	-0.664728000
1	-3.897813000	-4.922338000	0.123453000
1	-3.615343000	-3.200229000	-0.210710000
8	-2.834967000	-2.222802000	3.277140000
6	-4.036966000	-1.513456000	3.079794000
1	-4.324245000	-0.987943000	4.004069000
1	-3.898788000	-0.752425000	2.296139000
8	-2.949802000	2.698328000	2.760671000
6	-4.151909000	2.858707000	2.039863000
1	-4.352655000	3.926793000	1.865575000
1	-4.073161000	2.370193000	1.055002000
8	1.779263000	4.687463000	0.703978000
6	2.996953000	4.326238000	1.321948000
1	3.068853000	4.786771000	2.318798000
1	3.036019000	3.237102000	1.450584000
8	1.807404000	0.714418000	3.885873000
6	3.034166000	0.033068000	3.720983000
1	3.181122000	-0.681563000	4.545494000
1	3.020216000	-0.541632000	2.784416000
8	2.099973000	-3.864656000	2.021393000
6	3.330695000	-4.475547000	1.697438000

1	3.239437000	-5.569868000	1.761720000
1	3.630954000	-4.234522000	0.668231000
8	2.407296000	-3.065683000	-3.143974000
6	3.599282000	-2.341661000	-3.353926000
1	3.686647000	-2.038322000	-4.408070000
1	3.592143000	-1.428577000	-2.740284000
8	2.050415000	1.983367000	-3.578743000
6	3.230764000	2.575263000	-3.077055000
1	3.328578000	3.600911000	-3.466035000
1	3.179432000	2.634233000	-1.983627000
6	-5.279561000	2.251188000	2.855678000
1	-5.057587000	1.194531000	3.048682000
1	-5.312608000	2.751857000	3.830938000
6	-6.630510000	2.379168000	2.153178000
1	-6.805878000	3.430438000	1.882783000
1	-6.601908000	1.821948000	1.206091000
6	-7.799489000	1.881883000	3.003388000
1	-7.631609000	0.832256000	3.279292000
1	-7.823849000	2.447233000	3.945609000
6	-9.151987000	2.008864000	2.304080000
1	-9.297838000	3.049175000	1.984031000
1	-9.142218000	1.407009000	1.386124000
6	-10.324225000	1.573299000	3.179594000
1	-10.216420000	0.527220000	3.487302000
1	-10.384482000	2.183074000	4.088338000
1	-11.275568000	1.670090000	2.646364000
6	-5.128008000	4.146324000	-1.558322000
1	-4.870096000	4.308917000	-0.504065000
1	-5.185753000	5.137705000	-2.023961000
6	-6.476044000	3.438060000	-1.653759000
1	-6.682719000	3.171158000	-2.700259000
1	-6.429998000	2.490924000	-1.099409000
6	-7.631053000	4.277932000	-1.112497000
1	-7.404629000	4.587457000	-0.082201000
1	-7.715381000	5.203642000	-1.698729000
6	-8.963550000	3.533045000	-1.131087000
1	-9.175511000	3.194692000	-2.154771000
1	-8.869192000	2.626484000	-0.521302000
6	-10.129963000	4.372237000	-0.616504000
1	-9.941706000	4.721043000	0.405718000
1	-10.286676000	5.257368000	-1.243574000
1	-11.061201000	3.796570000	-0.606800000
6	-4.690414000	-0.303518000	-3.005142000
1	-4.802290000	0.646349000	-3.544815000
1	-5.020801000	-1.094467000	-3.690890000
6	-5.568438000	-0.308185000	-1.754727000
1	-5.384200000	-1.232207000	-1.188527000
1	-5.275318000	0.514758000	-1.085864000
6	-7.059764000	-0.205169000	-2.072189000
1	-7.255773000	0.723473000	-2.625687000
1	-7.343543000	-1.027112000	-2.744170000
6	-7.942254000	-0.250146000	-0.828078000
1	-7.713982000	-1.162264000	-0.261311000
1	-7.677790000	0.586674000	-0.167857000
6	-9.434623000	-0.199985000	-1.143459000
1	-9.687959000	0.711075000	-1.696435000
1	-9.736427000	-1.055325000	-1.758399000
1	-10.038946000	-0.217136000	-0.229673000
6	-4.633331000	-4.260815000	-1.797799000
1	-4.404413000	-3.470616000	-2.525368000
1	-4.522450000	-5.216822000	-2.322972000
6	-6.064907000	-4.100661000	-1.292198000

1	-6.312566000	-4.929872000	-0.614706000
1	-6.140548000	-3.184203000	-0.689270000
6	-7.092587000	-4.035219000	-2.419018000
1	-6.834323000	-3.204487000	-3.091197000
1	-7.032452000	-4.948572000	-3.026968000
6	-8.520109000	-3.850760000	-1.908333000
1	-8.798385000	-4.711933000	-1.286325000
1	-8.547998000	-2.976707000	-1.245650000
6	-9.540770000	-3.669093000	-3.028566000
1	-9.301105000	-2.788286000	-3.636400000
1	-9.554452000	-4.538168000	-3.696077000
1	-10.551953000	-3.535842000	-2.629625000
6	-5.139547000	-2.478837000	2.683484000
1	-4.894487000	-2.942560000	1.719691000
1	-5.192970000	-3.286102000	3.423725000
6	-6.480915000	-1.751257000	2.599746000
1	-6.705914000	-1.310913000	3.581941000
1	-6.398275000	-0.906143000	1.899255000
6	-7.653637000	-2.631550000	2.173289000
1	-7.495654000	-2.983609000	1.144368000
1	-7.689300000	-3.533387000	2.799934000
6	-8.989867000	-1.893951000	2.265977000
1	-9.201259000	-1.674926000	3.321345000
1	-8.897991000	-0.919627000	1.768068000
6	-10.154928000	-2.667405000	1.655275000
1	-9.990431000	-2.841742000	0.585674000
1	-10.276558000	-3.644632000	2.136490000
1	-11.096651000	-2.119091000	1.763826000
6	4.385362000	-3.935475000	2.649516000
1	4.373461000	-2.841379000	2.567900000
1	4.109884000	-4.176316000	3.682406000
6	5.784205000	-4.468637000	2.317932000
1	5.920488000	-5.467466000	2.752339000
1	5.875412000	-4.608797000	1.229704000
6	6.913027000	-3.543329000	2.774634000
1	6.744549000	-2.541682000	2.354626000
1	6.879710000	-3.422196000	3.866303000
6	8.294412000	-4.041905000	2.349788000
1	8.497051000	-5.008339000	2.829820000
1	8.285156000	-4.236917000	1.267773000
6	9.416811000	-3.058564000	2.675022000
1	9.273258000	-2.109785000	2.144964000
1	9.447894000	-2.834225000	3.747496000
1	10.394230000	-3.458592000	2.386185000
6	4.767576000	-3.223237000	-2.955001000
1	4.607690000	-3.552360000	-1.920863000
1	4.778319000	-4.126269000	-3.576538000
6	6.091601000	-2.468345000	-3.064102000
1	6.334238000	-2.288673000	-4.120347000
1	5.969106000	-1.478022000	-2.606145000
6	7.261923000	-3.173556000	-2.380757000
1	6.990361000	-3.393863000	-1.337741000
1	7.448474000	-4.146134000	-2.856411000
6	8.539534000	-2.335210000	-2.392726000
1	8.868494000	-2.182395000	-3.428886000
1	8.304868000	-1.335905000	-2.002295000
6	9.672081000	-2.946315000	-1.571180000
1	9.384008000	-3.043202000	-0.516955000
1	9.930832000	-3.946726000	-1.936289000
1	10.575565000	-2.329129000	-1.614205000
6	4.430908000	1.736940000	-3.475221000
1	4.347129000	0.750945000	-3.000859000

1	4.432490000	1.579491000	-4.560405000
6	5.721906000	2.422525000	-3.028291000
1	5.839423000	3.371337000	-3.570577000
1	5.630381000	2.692962000	-1.966570000
6	6.981874000	1.579161000	-3.207134000
1	6.854161000	0.620584000	-2.686309000
1	7.121344000	1.337241000	-4.269788000
6	8.224870000	2.286777000	-2.668460000
1	8.327135000	3.261714000	-3.164665000
1	8.072087000	2.506000000	-1.602607000
6	9.515262000	1.489595000	-2.839717000
1	9.468288000	0.538846000	-2.297589000
1	9.699509000	1.259523000	-3.895256000
1	10.377995000	2.047594000	-2.460965000
6	4.145199000	4.787675000	0.442514000
1	4.023260000	4.349965000	-0.557905000
1	4.097513000	5.876111000	0.317754000
6	5.493123000	4.374150000	1.032857000
1	5.588962000	4.776962000	2.050778000
1	5.517075000	3.281438000	1.144216000
6	6.697611000	4.817684000	0.204091000
1	6.565519000	4.505676000	-0.841817000
1	6.749288000	5.915095000	0.191432000
6	8.012164000	4.246956000	0.736189000
1	8.099200000	4.469602000	1.808222000
1	7.978089000	3.150327000	0.660932000
6	9.243456000	4.771783000	0.001921000
1	9.184369000	4.557998000	-1.071185000
1	9.334813000	5.857496000	0.119263000
1	10.161056000	4.314050000	0.386055000
6	4.177352000	1.027966000	3.686892000
1	4.034524000	1.710245000	2.839219000
1	4.169051000	1.635936000	4.599836000
6	5.505104000	0.285969000	3.539449000
1	5.648786000	-0.380481000	4.402032000
1	5.447948000	-0.365020000	2.657464000
6	6.724367000	1.194225000	3.402807000
1	6.570813000	1.893008000	2.568791000
1	6.831582000	1.812155000	4.305483000
6	8.008093000	0.400291000	3.164258000
1	8.126148000	-0.346551000	3.960970000
1	7.896954000	-0.170342000	2.232523000
6	9.261748000	1.267730000	3.086868000
1	9.188333000	1.998258000	2.273328000
1	9.411933000	1.825214000	4.018496000
1	10.155753000	0.659503000	2.912385000
1	0.832919000	1.878071000	-0.073620000
8	1.066094000	-0.424050000	0.159356000
6	1.968401000	0.367873000	0.046692000
8	1.787331000	1.691116000	-0.087244000
6	3.416792000	0.001463000	0.041024000
6	4.428535000	0.960162000	-0.029513000
6	3.747598000	-1.350766000	0.119200000
6	5.764513000	0.573802000	-0.027771000
1	4.164383000	2.008551000	-0.090767000
1	2.942727000	-2.076644000	0.182255000
1	6.546405000	1.326174000	-0.083422000
1	7.133701000	-1.088947000	0.047948000
6	6.093046000	-0.776280000	0.046407000
6	5.083199000	-1.732003000	0.122886000
1	5.345645000	-2.781836000	0.181727000

D’’

E_1 = -10059.0012905 A.U.

Zero-point correction= 2.455738 (Hartree/Particle)

Thermal correction to Energy= 2.585392

Thermal correction to Enthalpy= 2.586336

Thermal correction to Gibbs Free Energy= 2.282545

E_2 = -10065.1581483 A.U.

At No.	X	Y	Z
6	-1.171193000	-3.234339000	2.773265000
6	-2.419101000	-2.659039000	3.025303000
6	-0.029229000	-2.591570000	3.247939000
6	-2.505288000	-1.495107000	3.787730000
6	-0.114113000	-1.402326000	3.962000000
6	-1.365411000	-0.851664000	4.260215000
1	0.927513000	-3.038925000	3.010338000
1	-3.462827000	-1.033994000	4.000252000
6	-1.035356000	-4.479316000	1.924040000
1	-0.157859000	-5.045550000	2.246351000
1	-1.915582000	-5.113558000	2.057060000
6	-0.903324000	-4.104078000	0.464419000
6	-2.039537000	-3.983544000	-0.322943000
6	0.339447000	-3.783363000	-0.090837000
6	-1.965179000	-3.548666000	-1.642987000
6	0.420805000	-3.350147000	-1.413324000
6	-0.724339000	-3.231311000	-2.206490000
1	-2.990304000	-4.203810000	0.144763000
1	1.376589000	-3.075108000	-1.850406000
6	-0.620386000	-2.728564000	-3.633169000
1	-1.407805000	-3.190334000	-4.236406000
1	0.340079000	-3.033221000	-4.053873000
6	-0.750143000	-1.220513000	-3.715850000
6	-1.998411000	-0.623214000	-3.546818000
6	0.361590000	-0.399321000	-3.918271000
6	-2.155541000	0.759083000	-3.567235000
6	0.201955000	0.986284000	-3.937096000
6	-1.043723000	1.582441000	-3.772390000
1	-2.839858000	-1.283779000	-3.379718000
1	1.051289000	1.645258000	-4.063195000
6	-1.176924000	3.089752000	-3.797443000
1	-2.111071000	3.364297000	-4.296281000
1	-0.353688000	3.509479000	-4.383063000
6	-1.167382000	3.688419000	-2.408756000
6	0.032962000	3.911037000	-1.726260000
6	-2.363137000	3.992818000	-1.768205000
6	0.013740000	4.426786000	-0.433273000
6	-2.385207000	4.506557000	-0.473447000
6	-1.185976000	4.730504000	0.208152000
1	-3.280867000	3.785936000	-2.304829000
1	0.930457000	4.576296000	0.125938000
6	-1.176235000	5.191426000	1.650274000
1	-0.271625000	5.772588000	1.843848000
1	-2.037614000	5.836899000	1.841323000
6	-1.230590000	3.992114000	2.572529000
6	-0.069823000	3.391740000	3.065161000
6	-2.455120000	3.395503000	2.853800000
6	-0.158791000	2.268227000	3.885191000
6	-2.536962000	2.216978000	3.588658000
6	-1.386748000	1.669266000	4.163708000
1	-3.341939000	3.859276000	2.439482000
1	0.730875000	1.806791000	4.298766000
6	-1.471396000	0.438662000	5.042938000

1	-0.668287000	0.479070000	5.784373000
1	-2.421038000	0.448883000	5.584999000
8	-3.365122000	1.382961000	-3.399660000
6	-4.489382000	0.556470000	-3.201555000
1	-4.351134000	-0.065607000	-2.303564000
1	-4.609016000	-0.124017000	-4.058836000
8	-3.080905000	-3.408041000	-2.433581000
6	-4.330298000	-3.483699000	-1.778787000
1	-4.360125000	-2.759370000	-0.950120000
1	-4.472003000	-4.484421000	-1.344384000
8	-3.500419000	-3.262860000	2.433275000
6	-4.779527000	-3.164134000	3.025459000
1	-4.812688000	-3.768533000	3.944161000
1	-5.018903000	-2.130663000	3.301072000
8	-3.714598000	1.541822000	3.775479000
6	-4.775315000	1.860882000	2.898541000
1	-5.291400000	2.768060000	3.249496000
1	-4.382817000	2.069358000	1.893638000
8	-3.546662000	4.808298000	0.191810000
6	-4.753576000	4.365364000	-0.389020000
1	-4.909795000	4.847284000	-1.365650000
1	-4.703449000	3.278807000	-0.560137000
8	1.179695000	3.600753000	-2.404507000
6	2.412349000	3.744441000	-1.740021000
1	2.533681000	4.776114000	-1.375482000
1	2.453317000	3.075664000	-0.864512000
8	1.117046000	3.975522000	2.703426000
6	2.306279000	3.241992000	2.885119000
1	2.515503000	3.100567000	3.956760000
1	2.209382000	2.242183000	2.431474000
8	0.980872000	-0.714775000	4.414283000
6	2.253335000	-1.202556000	4.055359000
1	2.405543000	-2.209263000	4.475083000
1	2.336396000	-1.283303000	2.960252000
8	1.405685000	-3.942684000	0.747482000
6	2.691293000	-3.495495000	0.372191000
1	3.016388000	-3.968927000	-0.563690000
1	2.690481000	-2.407947000	0.207007000
8	1.564706000	-1.021332000	-4.118594000
6	2.731378000	-0.231214000	-4.049582000
1	2.655260000	0.640202000	-4.714786000
1	2.860572000	0.136439000	-3.019451000
6	-5.910541000	4.698866000	0.533811000
1	-5.759778000	4.203422000	1.500792000
1	-5.920244000	5.778361000	0.725787000
6	-7.236823000	4.253689000	-0.080528000
1	-7.381600000	4.758550000	-1.046141000
1	-7.185841000	3.178848000	-0.305195000
6	-8.450780000	4.509159000	0.809176000
1	-8.320878000	3.976854000	1.762175000
1	-8.509481000	5.577103000	1.061233000
6	-9.756958000	4.061991000	0.153822000
1	-9.924702000	4.648528000	-0.759086000
1	-9.649493000	3.019395000	-0.170472000
6	-10.967074000	4.181912000	1.076315000
1	-10.838007000	3.564426000	1.973386000
1	-11.112585000	5.217105000	1.405671000
1	-11.884572000	3.856533000	0.575100000
6	-5.732833000	1.414900000	-3.055880000
1	-5.635197000	2.057609000	-2.172232000
1	-5.815068000	2.076856000	-3.926380000
6	-6.979630000	0.538339000	-2.940036000

1	-7.046785000	-0.103194000	-3.830344000
1	-6.870555000	-0.143575000	-2.083133000
6	-8.285351000	1.316146000	-2.791094000
1	-8.256961000	1.909313000	-1.866607000
1	-8.381693000	2.040021000	-3.612074000
6	-9.506856000	0.396872000	-2.776958000
1	-9.571971000	-0.119510000	-3.744064000
1	-9.358860000	-0.389783000	-2.024697000
6	-10.817519000	1.125449000	-2.494218000
1	-10.803116000	1.588336000	-1.500563000
1	-10.995206000	1.920602000	-3.227242000
1	-11.668705000	0.437280000	-2.530246000
6	-5.450576000	-3.209981000	-2.764735000
1	-5.314599000	-2.221894000	-3.220908000
1	-5.402245000	-3.946379000	-3.575928000
6	-6.805878000	-3.286044000	-2.059698000
1	-6.867248000	-4.227830000	-1.495034000
1	-6.872934000	-2.483125000	-1.309825000
6	-8.007548000	-3.196845000	-2.997824000
1	-7.997758000	-2.231846000	-3.522335000
1	-7.924562000	-3.971407000	-3.772902000
6	-9.332217000	-3.361354000	-2.255282000
1	-9.336371000	-4.329827000	-1.737583000
1	-9.394587000	-2.599120000	-1.467572000
6	-10.558449000	-3.255310000	-3.157078000
1	-10.602090000	-2.276384000	-3.647631000
1	-10.538080000	-4.020480000	-3.941501000
1	-11.483391000	-3.385394000	-2.585260000
6	-5.794138000	-3.652203000	2.004356000
1	-5.758941000	-2.981277000	1.135080000
1	-5.493460000	-4.645514000	1.647870000
6	-7.212709000	-3.702466000	2.568384000
1	-7.253706000	-4.430219000	3.390668000
1	-7.467748000	-2.728031000	3.008153000
6	-8.263177000	-4.064358000	1.518940000
1	-8.242042000	-3.318650000	0.711381000
1	-7.999288000	-5.023670000	1.050673000
6	-9.675284000	-4.151886000	2.097231000
1	-9.686769000	-4.899049000	2.901971000
1	-9.929659000	-3.192179000	2.566445000
6	-10.734777000	-4.507869000	1.056444000
1	-10.795515000	-3.743011000	0.274354000
1	-10.501651000	-5.461657000	0.568560000
1	-11.726018000	-4.598223000	1.512514000
6	-5.747020000	0.697678000	2.820902000
1	-5.217642000	-0.172747000	2.414545000
1	-6.090450000	0.433277000	3.828997000
6	-6.935157000	1.051953000	1.928737000
1	-7.427544000	1.951133000	2.325157000
1	-6.566785000	1.322643000	0.928244000
6	-7.973644000	-0.058333000	1.790504000
1	-7.510505000	-0.941831000	1.326987000
1	-8.311447000	-0.373916000	2.788029000
6	-9.178714000	0.380812000	0.959978000
1	-9.623866000	1.274422000	1.418091000
1	-8.828374000	0.689724000	-0.033559000
6	-10.243200000	-0.700955000	0.813547000
1	-9.813714000	-1.613560000	0.386722000
1	-10.675303000	-0.965379000	1.785421000
1	-11.058634000	-0.376172000	0.158855000
6	3.309488000	-0.253523000	4.592264000
1	3.249847000	0.701321000	4.054064000

1	3.093639000	-0.041601000	5.646172000
6	4.707828000	-0.851973000	4.453763000
1	4.774657000	-1.757619000	5.073079000
1	4.859285000	-1.182753000	3.415943000
6	5.827423000	0.110635000	4.843147000
1	5.846534000	0.950468000	4.133975000
1	5.608187000	0.552117000	5.825267000
6	7.203273000	-0.556531000	4.888338000
1	7.225200000	-1.277616000	5.715967000
1	7.357075000	-1.146196000	3.975198000
6	8.342896000	0.447233000	5.046469000
1	8.358756000	1.156779000	4.209740000
1	8.228643000	1.030076000	5.967575000
1	9.316138000	-0.052320000	5.082716000
6	3.616239000	-3.890276000	1.515610000
1	3.244538000	-3.437950000	2.444227000
1	3.546277000	-4.976720000	1.649138000
6	5.070899000	-3.488456000	1.297636000
1	5.425273000	-3.898834000	0.343917000
1	5.119181000	-2.394206000	1.203631000
6	5.998019000	-3.950599000	2.421926000
1	5.703188000	-3.467919000	3.364706000
1	5.858096000	-5.029484000	2.577139000
6	7.479428000	-3.680153000	2.153006000
1	7.772653000	-4.155461000	1.208218000
1	7.645354000	-2.605590000	2.007231000
6	8.383045000	-4.165456000	3.283701000
1	8.130869000	-3.664934000	4.226223000
1	8.272424000	-5.244483000	3.440529000
1	9.436243000	-3.962190000	3.066021000
6	3.926818000	-1.065576000	-4.463658000
1	3.965029000	-1.967502000	-3.846791000
1	3.807030000	-1.379587000	-5.507609000
6	5.215585000	-0.263753000	-4.288531000
1	5.203312000	0.616355000	-4.947859000
1	5.247224000	0.133154000	-3.263147000
6	6.484172000	-1.073693000	-4.547107000
1	6.461168000	-1.988347000	-3.940087000
1	6.505556000	-1.406202000	-5.593900000
6	7.757204000	-0.288880000	-4.232411000
1	7.801302000	0.605208000	-4.868814000
1	7.694020000	0.080785000	-3.199189000
6	9.035966000	-1.107767000	-4.389073000
1	9.031611000	-1.964316000	-3.706508000
1	9.134078000	-1.492578000	-5.410473000
1	9.924417000	-0.507963000	-4.166787000
6	3.521715000	3.408682000	-2.720769000
1	3.467110000	2.345877000	-2.985285000
1	3.347773000	3.977274000	-3.642289000
6	4.901159000	3.743953000	-2.159012000
1	4.934561000	4.810535000	-1.894310000
1	5.052734000	3.186719000	-1.223952000
6	6.040364000	3.426356000	-3.128333000
1	6.159829000	2.336850000	-3.204039000
1	5.769555000	3.767433000	-4.137305000
6	7.367683000	4.073332000	-2.729502000
1	7.271131000	5.163599000	-2.813279000
1	7.572169000	3.872855000	-1.669534000
6	8.543600000	3.591905000	-3.576260000
1	8.702201000	2.513665000	-3.455158000
1	8.362423000	3.779044000	-4.640969000
1	9.472625000	4.100158000	-3.298979000

6	3.435336000	4.026376000	2.238389000
1	3.247828000	4.107027000	1.159574000
1	3.418898000	5.046885000	2.639316000
6	4.802518000	3.392667000	2.486353000
1	4.968445000	3.293933000	3.568566000
1	4.802470000	2.372862000	2.076234000
6	5.950281000	4.192266000	1.867323000
1	5.883237000	4.136017000	0.770853000
1	5.829163000	5.254917000	2.118945000
6	7.335094000	3.733152000	2.326204000
1	7.423731000	3.889987000	3.409249000
1	7.438012000	2.652403000	2.170462000
6	8.469200000	4.455786000	1.603475000
1	8.425003000	4.273308000	0.522930000
1	8.406633000	5.538996000	1.756580000
1	9.448780000	4.121790000	1.960243000
7	7.019295000	-1.976684000	-1.119809000
6	5.676424000	-1.552632000	-1.196930000
6	7.851657000	-1.084192000	-0.473030000
6	6.949507000	0.077179000	-0.014664000
7	5.685695000	-0.281015000	-0.673555000
8	4.757189000	-2.184885000	-1.644244000
8	9.031819000	-1.196531000	-0.276680000
35	4.069909000	0.481136000	-0.206690000
35	7.544875000	-3.606125000	-1.761520000
6	7.484818000	1.413977000	-0.504363000
1	6.824158000	2.218233000	-0.168710000
1	8.482230000	1.586498000	-0.091946000
1	7.541196000	1.440907000	-1.593884000
6	6.848629000	0.036441000	1.507482000
1	7.845501000	0.154806000	1.939984000
1	6.213366000	0.848526000	1.865290000
1	6.426881000	-0.908328000	1.851531000

Methylcyclohexane
E_1 = -275.118975315 A.U.
Zero-point correction= 0.201296 (Hartree/Particle)
Thermal correction to Energy= 0.208286
Thermal correction to Enthalpy= 0.209230
Thermal correction to Gibbs Free Energy= 0.170847
E_2 = -275.193404375 A.U.

At No.	X	Y	Z
6	0.292782000	1.258652000	0.169279000
1	0.788972000	2.154305000	-0.225506000
1	0.385174000	1.305860000	1.265138000
6	-1.190255000	1.262676000	-0.212236000
1	-1.681047000	2.157876000	0.187367000
1	-1.280720000	1.315283000	-1.306644000
6	-1.896668000	0.000010000	0.289204000
1	-1.896943000	0.000166000	1.388669000
1	-2.946321000	-0.000150000	-0.027114000
6	-1.190171000	-1.262790000	-0.212064000
1	-1.280885000	-1.315559000	-1.306447000
1	-1.680777000	-2.157984000	0.187798000
6	0.292917000	-1.258622000	0.169117000
1	0.385505000	-1.305684000	1.264960000
1	0.789012000	-2.154315000	-0.225677000
6	1.014316000	0.000041000	-0.331135000
1	0.959397000	0.000220000	-1.431154000
6	2.487147000	0.000029000	0.074364000
1	3.005294000	0.886364000	-0.309013000

1	2.587933000	0.000314000	1.167001000
1	3.004998000	-0.886669000	-0.308555000

D’’’

E_1 = -4736.74091139 A.U.

Zero-point correction= 2.541010 (Hartree/Particle)

Thermal correction to Energy= 2.665380

Thermal correction to Enthalpy= 2.666324

Thermal correction to Gibbs Free Energy= 2.376986

E_2 = -4737.96841734 A.U.

At No.	X	Y	Z
6	-0.041819000	-4.230620000	-1.619317000
6	1.212832000	-3.815658000	-2.073385000
6	-1.175191000	-3.647094000	-2.177848000
6	1.308737000	-2.846206000	-3.066334000
6	-1.081165000	-2.654776000	-3.154718000
6	0.176168000	-2.249202000	-3.614740000
1	-2.135895000	-3.979069000	-1.802525000
1	2.273560000	-2.509237000	-3.425307000
6	-0.177138000	-5.213364000	-0.474736000
1	-1.111473000	-5.769707000	-0.574504000
1	0.647174000	-5.931139000	-0.501193000
6	-0.152860000	-4.465670000	0.842284000
6	1.054182000	-4.016067000	1.355907000
6	-1.328468000	-4.117317000	1.520363000
6	1.114503000	-3.199541000	2.478492000
6	-1.263882000	-3.377235000	2.699299000
6	-0.046030000	-2.891408000	3.185122000
1	1.966081000	-4.289296000	0.837559000
1	-2.163700000	-3.121245000	3.246593000
6	0.013724000	-2.051781000	4.444328000
1	0.901198000	-2.332629000	5.018424000
1	-0.862382000	-2.273126000	5.060480000
6	0.065387000	-0.564578000	4.167513000
6	1.282793000	0.110478000	4.131556000
6	-1.110188000	0.163599000	3.951803000
6	1.342441000	1.479772000	3.884359000
6	-1.050486000	1.531994000	3.690886000
6	0.170045000	2.204824000	3.655612000
1	2.185213000	-0.465687000	4.305377000
1	-1.949774000	2.107370000	3.498459000
6	0.234707000	3.682210000	3.329496000
1	1.091678000	4.129472000	3.839392000
1	-0.667924000	4.175581000	3.695916000
6	0.364356000	3.908115000	1.837477000
6	-0.761281000	4.172246000	1.054190000
6	1.604554000	3.796022000	1.211535000
6	-0.628409000	4.329468000	-0.324758000
6	1.732276000	3.924092000	-0.167453000
6	0.604060000	4.183614000	-0.954653000
1	2.461485000	3.577257000	1.837152000
1	-1.489249000	4.528138000	-0.953086000
6	0.696298000	4.227931000	-2.465646000
1	-0.103667000	4.859105000	-2.859846000
1	1.650177000	4.671830000	-2.764668000
6	0.587002000	2.838406000	-3.063730000
6	-0.629399000	2.330687000	-3.526521000
6	1.712175000	2.019640000	-3.123682000
6	-0.689249000	1.036076000	-4.045056000
6	1.650868000	0.723853000	-3.629303000

6	0.435903000	0.218229000	-4.104989000
1	2.636632000	2.429637000	-2.739001000
1	-1.621275000	0.622326000	-4.411179000
6	0.329680000	-1.180219000	-4.676426000
1	-0.525179000	-1.219480000	-5.356966000
1	1.227537000	-1.396838000	-5.263695000
8	2.507765000	2.200267000	3.878522000
6	3.730276000	1.513784000	3.733056000
1	3.707379000	0.898398000	2.820761000
1	3.907442000	0.840416000	4.584781000
8	2.332004000	-2.727592000	2.920511000
6	3.058792000	-1.962302000	1.967361000
1	2.625735000	-0.953077000	1.901117000
1	2.978522000	-2.413439000	0.970008000
8	2.302924000	-4.413421000	-1.498694000
6	3.571046000	-3.842860000	-1.732314000
1	3.860520000	-3.965691000	-2.787361000
1	3.539720000	-2.762723000	-1.519992000
8	2.744962000	-0.097779000	-3.710642000
6	3.960126000	0.403903000	-3.199237000
1	4.224567000	1.341160000	-3.712146000
1	3.851194000	0.632463000	-2.127360000
8	2.926801000	3.816563000	-0.830145000
6	4.084282000	3.495231000	-0.091612000
1	4.257621000	4.247934000	0.692566000
1	3.963003000	2.518870000	0.405386000
8	-1.953122000	4.221490000	1.724340000
6	-3.138440000	4.533696000	1.024507000
1	-3.189971000	5.615408000	0.830482000
1	-3.162497000	4.018456000	0.058415000
8	-1.716799000	3.158466000	-3.431651000
6	-2.953276000	2.657531000	-3.891787000
1	-2.930545000	2.537013000	-4.985757000
1	-3.138221000	1.668478000	-3.453892000
8	-2.173754000	-2.033221000	-3.702037000
6	-3.438740000	-2.409705000	-3.200470000
1	-3.688896000	-3.431325000	-3.525732000
1	-3.414381000	-2.406676000	-2.103379000
8	-2.494206000	-4.529337000	0.935309000
6	-3.727390000	-4.178604000	1.527740000
1	-3.893014000	-4.772235000	2.439403000
1	-3.723258000	-3.120604000	1.813427000
8	-2.274716000	-0.548438000	4.027701000
6	-3.499317000	0.140021000	3.889361000
1	-3.592755000	0.915419000	4.664343000
1	-3.536734000	0.638851000	2.912048000
6	5.258938000	3.471236000	-1.054531000
1	5.067401000	2.727406000	-1.838099000
1	5.318473000	4.446590000	-1.552431000
6	6.580539000	3.158156000	-0.355453000
1	6.737280000	3.866765000	0.470393000
1	6.521617000	2.161889000	0.105098000
6	7.782370000	3.215335000	-1.298648000
1	7.635521000	2.502887000	-2.121591000
1	7.828905000	4.211781000	-1.760135000
6	9.111700000	2.918912000	-0.606319000
1	9.240209000	3.606600000	0.239973000
1	9.078528000	1.909900000	-0.175382000
6	10.313396000	3.028384000	-1.541508000
1	10.224266000	2.327421000	-2.378954000
1	10.395609000	4.038024000	-1.960009000
1	11.247441000	2.805889000	-1.015383000

6	4.836047000	2.551728000	3.659874000
1	4.597458000	3.272447000	2.867779000
1	4.853317000	3.113925000	4.601435000
6	6.200815000	1.924884000	3.389693000
1	6.387052000	1.114203000	4.109037000
1	6.194063000	1.456408000	2.396037000
6	7.348465000	2.929649000	3.458261000
1	7.135742000	3.772084000	2.784724000
1	7.403976000	3.353726000	4.470501000
6	8.693720000	2.313336000	3.082040000
1	8.893594000	1.452669000	3.735251000
1	8.625766000	1.915389000	2.062182000
6	9.853899000	3.301422000	3.165881000
1	9.674852000	4.172054000	2.523989000
1	9.986863000	3.668544000	4.189951000
1	10.794460000	2.839774000	2.848096000
6	4.514942000	-1.926840000	2.393145000
1	4.604831000	-1.426832000	3.366643000
1	4.847948000	-2.962216000	2.541635000
6	5.412484000	-1.242897000	1.363366000
1	5.252222000	-1.706711000	0.379804000
1	5.119314000	-0.188880000	1.247585000
6	6.895632000	-1.328087000	1.722677000
1	7.065561000	-0.852874000	2.698618000
1	7.176860000	-2.383642000	1.843791000
6	7.806637000	-0.686607000	0.680335000
1	7.607127000	-1.143972000	-0.297340000
1	7.543785000	0.374192000	0.574270000
6	9.290251000	-0.813678000	1.014759000
1	9.518782000	-0.332736000	1.972203000
1	9.587815000	-1.865551000	1.092606000
1	9.916579000	-0.346895000	0.246456000
6	4.574996000	-4.533621000	-0.827669000
1	4.333726000	-4.305826000	0.219390000
1	4.470510000	-5.618382000	-0.947628000
6	6.008233000	-4.106487000	-1.134084000
1	6.268566000	-4.396898000	-2.161601000
1	6.079102000	-3.009925000	-1.102189000
6	7.027107000	-4.694174000	-0.160978000
1	6.759281000	-4.393729000	0.862067000
1	6.966713000	-5.791163000	-0.181718000
6	8.458268000	-4.253090000	-0.461348000
1	8.746183000	-4.606564000	-1.460431000
1	8.489157000	-3.157295000	-0.506650000
6	9.467325000	-4.744422000	0.573154000
1	9.216257000	-4.368904000	1.572514000
1	9.480110000	-5.839058000	0.624681000
1	10.481278000	-4.407273000	0.333372000
6	5.068893000	-0.610594000	-3.409988000
1	4.848881000	-1.526045000	-2.848148000
1	5.106423000	-0.885437000	-4.470901000
6	6.409966000	-0.025595000	-2.967262000
1	6.606962000	0.888344000	-3.546212000
1	6.342321000	0.290054000	-1.915015000
6	7.596924000	-0.973593000	-3.122681000
1	7.456100000	-1.850805000	-2.476466000
1	7.633940000	-1.357886000	-4.151362000
6	8.923828000	-0.293834000	-2.783688000
1	9.118541000	0.494005000	-3.523891000
1	8.828330000	0.217310000	-1.816732000
6	10.105314000	-1.258159000	-2.731560000
1	9.956647000	-2.016546000	-1.954132000

1	10.231998000	-1.781201000	-3.686374000
1	11.039130000	-0.730619000	-2.510213000
6	-4.492426000	-1.415330000	-3.653572000
1	-4.177849000	-0.423282000	-3.307645000
1	-4.534718000	-1.371748000	-4.747159000
6	-5.857964000	-1.779169000	-3.044892000
1	-6.403034000	-2.459752000	-3.710543000
1	-5.687143000	-2.350978000	-2.121994000
6	-6.737513000	-0.576026000	-2.690009000
1	-6.108233000	0.225845000	-2.283002000
1	-7.204493000	-0.166722000	-3.596247000
6	-7.806738000	-0.910048000	-1.649955000
1	-8.442697000	-1.726531000	-2.016951000
1	-7.304625000	-1.294440000	-0.750134000
6	-8.671300000	0.289336000	-1.270811000
1	-8.050622000	1.116899000	-0.904052000
1	-9.238920000	0.660928000	-2.131524000
1	-9.386905000	0.033812000	-0.481978000
6	-4.815603000	-4.428629000	0.497822000
1	-4.574692000	-3.841993000	-0.399079000
1	-4.793432000	-5.484575000	0.201707000
6	-6.207598000	-4.049356000	1.000203000
1	-6.428755000	-4.591319000	1.930899000
1	-6.225932000	-2.980705000	1.256261000
6	-7.308533000	-4.330441000	-0.021687000
1	-7.066376000	-3.824861000	-0.967088000
1	-7.332390000	-5.405879000	-0.246734000
6	-8.689166000	-3.877065000	0.449321000
1	-8.920201000	-4.362794000	1.407005000
1	-8.664624000	-2.797206000	0.651358000
6	-9.796286000	-4.184219000	-0.556050000
1	-9.597592000	-3.701397000	-1.519863000
1	-9.874971000	-5.262347000	-0.736950000
1	-10.768923000	-3.831171000	-0.198551000
6	-4.616702000	-0.882667000	4.005561000
1	-4.381951000	-1.720153000	3.336981000
1	-4.630271000	-1.288224000	5.024804000
6	-5.984264000	-0.310193000	3.636978000
1	-6.187291000	0.596168000	4.224802000
1	-5.969278000	0.006123000	2.586782000
6	-7.126568000	-1.307292000	3.830400000
1	-6.845967000	-2.276062000	3.393319000
1	-7.280695000	-1.489166000	4.903022000
6	-8.432268000	-0.833365000	3.192782000
1	-8.667012000	0.177547000	3.552127000
1	-8.281663000	-0.743124000	2.106658000
6	-9.610290000	-1.762877000	3.472424000
1	-9.396041000	-2.781014000	3.129506000
1	-9.824459000	-1.811621000	4.546156000
1	-10.517973000	-1.421972000	2.963613000
6	-4.304050000	4.050744000	1.869709000
1	-4.153270000	2.978064000	2.046010000
1	-4.276505000	4.538123000	2.851496000
6	-5.655624000	4.275371000	1.192105000
1	-5.913485000	5.342817000	1.211017000
1	-5.581249000	4.002947000	0.129693000
6	-6.784181000	3.457208000	1.819468000
1	-6.487868000	2.399166000	1.843475000
1	-6.929684000	3.759418000	2.866131000
6	-8.098979000	3.584922000	1.052861000
1	-8.429545000	4.632010000	1.058218000
1	-7.913148000	3.331311000	0.000611000

6	-9.204688000	2.686799000	1.600297000
1	-8.902423000	1.634224000	1.564870000
1	-9.433877000	2.932139000	2.643956000
1	-10.127530000	2.789534000	1.019541000
6	-4.064758000	3.598000000	-3.467911000
1	-4.019141000	3.711463000	-2.378651000
1	-3.901636000	4.593126000	-3.897382000
6	-5.430364000	3.044750000	-3.885802000
1	-5.577263000	3.208348000	-4.962066000
1	-5.443044000	1.954846000	-3.742472000
6	-6.604498000	3.642520000	-3.111599000
1	-6.517299000	3.353620000	-2.053459000
1	-6.550170000	4.740032000	-3.136842000
6	-7.957966000	3.185078000	-3.656239000
1	-8.048013000	3.507566000	-4.702151000
1	-7.983522000	2.087469000	-3.671409000
6	-9.147470000	3.710045000	-2.855996000
1	-9.132562000	3.328827000	-1.829062000
1	-9.136538000	4.804895000	-2.804620000
1	-10.096704000	3.404849000	-3.308852000
6	-2.479522000	1.360536000	-0.471268000
1	-1.937791000	2.003406000	-1.172404000
1	-2.256836000	1.742484000	0.537403000
6	-3.984901000	1.441524000	-0.725498000
1	-4.340883000	2.474319000	-0.675629000
1	-4.199523000	1.092534000	-1.743649000
6	-4.756702000	0.585075000	0.266560000
1	-4.606674000	1.002987000	1.268187000
1	-5.837141000	0.630407000	0.072070000
6	-4.272278000	-0.861330000	0.231340000
1	-4.589417000	-1.301725000	-0.720658000
1	-4.776761000	-1.436374000	1.016202000
6	-2.750720000	-0.993567000	0.370326000
1	-2.451242000	-0.765302000	1.403371000
1	-2.451780000	-2.033559000	0.192320000
6	-1.968692000	-0.079487000	-0.582252000
1	-2.151336000	-0.414921000	-1.615223000
6	-0.465865000	-0.170480000	-0.319795000
1	0.090296000	0.461705000	-1.021531000
1	-0.232816000	0.163371000	0.700345000
1	-0.111943000	-1.202839000	-0.430584000

D_{1a}

E_1 = -5181.25294473 A.U.

At No.	X	Y	Z
6	0.147351000	0.179345000	4.224273000
6	1.378074000	-0.449509000	4.065964000
6	-1.005078000	-0.580647000	4.011357000
6	1.438788000	-1.800378000	3.723135000
6	-0.941192000	-1.916936000	3.628575000
6	0.301091000	-2.551703000	3.464636000
1	-1.955641000	-0.078200000	4.131200000
1	2.410801000	-2.271575000	3.622640000
6	0.018989000	1.638549000	4.620476000
1	-0.887044000	1.756691000	5.223643000
1	0.868756000	1.922863000	5.247853000
6	-0.040935000	2.580447000	3.436364000
6	1.106827000	3.253990000	3.026855000
6	-1.227188000	2.788347000	2.722182000
6	1.119234000	4.067204000	1.892883000
6	-1.223112000	3.624960000	1.610227000
6	-0.059193000	4.247434000	1.164707000

1	2.009623000	3.091613000	3.604427000
1	-2.121191000	3.791819000	1.028338000
6	-0.081107000	5.031224000	-0.131270000
1	0.783837000	5.696395000	-0.173106000
1	-0.982018000	5.649826000	-0.166661000
6	-0.062126000	4.105189000	-1.331485000
6	1.147679000	3.650575000	-1.850872000
6	-1.247883000	3.656761000	-1.919920000
6	1.194959000	2.774098000	-2.936015000
6	-1.202921000	2.797815000	-3.016386000
6	0.006983000	2.344606000	-3.540001000
1	2.056142000	4.003215000	-1.375516000
1	-2.114117000	2.440743000	-3.480978000
6	0.024792000	1.405666000	-4.732786000
1	0.897241000	1.628202000	-5.353361000
1	-0.868387000	1.591796000	-5.337234000
6	0.062693000	-0.054536000	-4.330811000
6	-1.100816000	-0.715324000	-3.922601000
6	1.258707000	-0.767526000	-4.335783000
6	-1.035530000	-2.033525000	-3.483820000
6	1.329587000	-2.085762000	-3.880116000
6	0.171766000	-2.728275000	-3.432802000
1	2.145639000	-0.246832000	-4.676936000
1	-1.919145000	-2.552897000	-3.137413000
6	0.210015000	-4.138246000	-2.873536000
1	-0.682877000	-4.675519000	-3.208337000
1	1.081279000	-4.662926000	-3.271998000
6	0.263801000	-4.161425000	-1.358126000
6	-0.900854000	-4.044881000	-0.593318000
6	1.477098000	-4.295863000	-0.686258000
6	-0.828932000	-4.003956000	0.794996000
6	1.545480000	-4.288715000	0.708363000
6	0.385032000	-4.102430000	1.467861000
1	2.374311000	-4.408845000	-1.285590000
1	-1.717789000	-3.865299000	1.396940000
6	0.412221000	-3.988069000	2.983662000
1	-0.415068000	-4.573473000	3.397186000
1	1.342080000	-4.418294000	3.362520000
8	2.362575000	2.308634000	-3.477606000
6	3.567430000	2.679915000	-2.837281000
1	3.547828000	2.349082000	-1.790385000
1	3.677649000	3.774523000	-2.842357000
8	2.235322000	4.682750000	1.395261000
6	3.471403000	4.549218000	2.069902000
1	3.585877000	3.539184000	2.473373000
1	3.514433000	5.257011000	2.910851000
8	2.554400000	0.267797000	4.209061000
6	3.204552000	0.095102000	5.461130000
1	2.614034000	0.583341000	6.251302000
1	3.260560000	-0.975828000	5.704734000
8	2.710726000	-4.457950000	1.406337000
6	3.933566000	-4.504746000	0.696909000
1	4.000377000	-5.432277000	0.109978000
1	3.993109000	-3.660133000	-0.004520000
8	2.489632000	-2.810124000	-3.802063000
6	3.710702000	-2.209093000	-4.188676000
1	3.819599000	-2.250681000	-5.282071000
1	3.728172000	-1.157594000	-3.889501000
8	-2.270870000	-0.004033000	-3.991868000
6	-3.465172000	-0.708461000	-3.732497000
1	-3.556562000	-1.559438000	-4.424649000
1	-3.445357000	-1.113289000	-2.711284000

8	-2.086454000	-3.970993000	-1.279486000
6	-3.273553000	-3.922940000	-0.521629000
1	-3.341679000	-4.805498000	0.132987000
1	-3.271325000	-3.033278000	0.124580000
8	-2.059864000	-2.675234000	3.407944000
6	-3.303771000	-2.012172000	3.486740000
1	-3.469217000	-1.634675000	4.506865000
1	-3.295158000	-1.141776000	2.817156000
8	-2.354446000	2.143951000	3.167114000
6	-3.590019000	2.605980000	2.662127000
1	-3.713544000	3.672778000	2.904524000
1	-3.611973000	2.514915000	1.568834000
8	-2.412473000	4.116247000	-1.362982000
6	-3.636716000	3.706677000	-1.932030000
1	-3.700876000	4.033763000	-2.980585000
1	-3.707204000	2.610283000	-1.920003000
6	4.852540000	-2.923824000	-3.480820000
1	4.559342000	-3.073567000	-2.434178000
1	5.013375000	-3.918263000	-3.914298000
6	6.132659000	-2.091103000	-3.530906000
1	6.402032000	-1.881268000	-4.576271000
1	5.931165000	-1.114519000	-3.066732000
6	7.325208000	-2.731767000	-2.823417000
1	7.047514000	-2.985059000	-1.790239000
1	7.582040000	-3.680205000	-3.315679000
6	8.545797000	-1.811785000	-2.809864000
1	8.774364000	-1.508169000	-3.840895000
1	8.288863000	-0.889386000	-2.271080000
6	9.784024000	-2.441178000	-2.178111000
1	9.593105000	-2.722651000	-1.137306000
1	10.085436000	-3.345950000	-2.718421000
1	10.630369000	-1.746126000	-2.187655000
6	4.740578000	2.041517000	-3.556079000
1	4.544669000	0.970715000	-3.686764000
1	4.840175000	2.475799000	-4.558278000
6	6.023628000	2.244902000	-2.750125000
1	6.112294000	3.304473000	-2.467199000
1	5.945288000	1.686352000	-1.805564000
6	7.297040000	1.824395000	-3.480208000
1	7.226562000	0.768220000	-3.774427000
1	7.389069000	2.398330000	-4.412972000
6	8.545781000	2.039519000	-2.626425000
1	8.583357000	3.091277000	-2.312634000
1	8.453686000	1.451165000	-1.702230000
6	9.845771000	1.675516000	-3.338739000
1	9.852083000	0.620772000	-3.634718000
1	9.977457000	2.276317000	-4.245989000
1	10.713332000	1.848591000	-2.693210000
6	4.597632000	4.788311000	1.077397000
1	4.437852000	4.119253000	0.222340000
1	4.558073000	5.815060000	0.694554000
6	5.955062000	4.495494000	1.716775000
1	6.166870000	5.243332000	2.493924000
1	5.901203000	3.525869000	2.231619000
6	7.119191000	4.444666000	0.728680000
1	6.938594000	3.640463000	0.000285000
1	7.165931000	5.378174000	0.150445000
6	8.458546000	4.211067000	1.430669000
1	8.671003000	5.065656000	2.086642000
1	8.369989000	3.336980000	2.091259000
6	9.623025000	3.997843000	0.466603000
1	9.480540000	3.084595000	-0.121179000

1	9.713615000	4.834923000	-0.235689000
1	10.573180000	3.905751000	1.002844000
6	4.601448000	0.691684000	5.390635000
1	4.514901000	1.739970000	5.075898000
1	4.993100000	0.709606000	6.414538000
6	5.571334000	-0.067435000	4.460277000
1	6.502032000	-0.292811000	4.996587000
1	5.134223000	-1.040372000	4.195239000
6	5.917728000	0.677573000	3.169739000
1	4.989969000	0.929721000	2.642355000
1	6.402501000	1.632562000	3.419483000
6	6.831648000	-0.130581000	2.246567000
1	7.816374000	-0.253173000	2.718382000
1	6.418508000	-1.140753000	2.138401000
6	6.995008000	0.487592000	0.859463000
1	6.026642000	0.550112000	0.348553000
1	7.406531000	1.502146000	0.917211000
1	7.665714000	-0.109940000	0.230448000
6	5.052672000	-4.404423000	1.721665000
1	4.763126000	-3.638519000	2.450874000
1	5.142216000	-5.349212000	2.271293000
6	6.391572000	-4.011152000	1.102165000
1	6.705844000	-4.753409000	0.354782000
1	6.263758000	-3.063095000	0.558104000
6	7.490601000	-3.833140000	2.149155000
1	7.124098000	-3.166248000	2.942591000
1	7.700812000	-4.796912000	2.633292000
6	8.780713000	-3.255006000	1.571862000
1	9.175950000	-3.940499000	0.810180000
1	8.547006000	-2.315589000	1.051526000
6	9.846280000	-2.993213000	2.633265000
1	9.482348000	-2.279024000	3.381348000
1	10.114980000	-3.916589000	3.159110000
1	10.758729000	-2.580638000	2.190645000
6	-4.429609000	-2.954004000	3.101198000
1	-4.187151000	-3.451644000	2.154921000
1	-4.527003000	-3.740347000	3.859399000
6	-5.737341000	-2.171172000	2.962583000
1	-5.850389000	-1.494698000	3.822724000
1	-5.675573000	-1.519914000	2.078199000
6	-6.989100000	-3.039903000	2.859608000
1	-6.890546000	-3.738808000	2.017939000
1	-7.080791000	-3.656608000	3.764622000
6	-8.253229000	-2.200446000	2.681256000
1	-8.309865000	-1.459093000	3.489414000
1	-8.163519000	-1.621312000	1.751666000
6	-9.540247000	-3.019586000	2.654895000
1	-9.530810000	-3.748410000	1.837207000
1	-9.672221000	-3.572534000	3.591954000
1	-10.415631000	-2.375275000	2.519888000
6	-4.732738000	1.787399000	3.233274000
1	-4.649881000	0.755794000	2.869427000
1	-4.660383000	1.752610000	4.326675000
6	-6.071626000	2.385957000	2.793762000
1	-6.247888000	3.327615000	3.331595000
1	-6.007189000	2.653430000	1.729546000
6	-7.267957000	1.455121000	2.970312000
1	-7.062481000	0.513758000	2.445709000
1	-7.397996000	1.201340000	4.032052000
6	-8.555454000	2.066815000	2.418596000
1	-8.768762000	3.005376000	2.948386000
1	-8.387835000	2.342400000	1.368352000

6	-9.766172000	1.140799000	2.503972000
1	-9.610296000	0.235868000	1.906665000
1	-9.954008000	0.827349000	3.537552000
1	-10.669827000	1.635705000	2.132848000
6	-4.764160000	4.310315000	-1.113781000
1	-4.635385000	4.011321000	-0.063959000
1	-4.685238000	5.403723000	-1.137323000
6	-6.133946000	3.857652000	-1.617590000
1	-6.269510000	4.177298000	-2.660101000
1	-6.165321000	2.760376000	-1.632248000
6	-7.295808000	4.378794000	-0.772738000
1	-7.109407000	4.153825000	0.287355000
1	-7.343354000	5.474012000	-0.847726000
6	-8.640194000	3.777071000	-1.181751000
1	-8.794497000	3.926759000	-2.258873000
1	-8.602680000	2.688669000	-1.030744000
6	-9.821347000	4.357612000	-0.407542000
1	-9.696261000	4.209367000	0.671131000
1	-9.914646000	5.434914000	-0.585746000
1	-10.763230000	3.884967000	-0.704611000
6	-4.647450000	0.228470000	-3.880888000
1	-4.550000000	1.046162000	-3.155867000
1	-4.638851000	0.677036000	-4.881605000
6	-5.954014000	-0.519984000	-3.630092000
1	-6.063001000	-1.331781000	-4.363522000
1	-5.893651000	-0.999434000	-2.644533000
6	-7.196620000	0.365871000	-3.665670000
1	-7.058257000	1.213466000	-2.981419000
1	-7.318208000	0.795013000	-4.670096000
6	-8.463239000	-0.393531000	-3.273121000
1	-8.583384000	-1.261241000	-3.936530000
1	-8.334020000	-0.800138000	-2.260181000
6	-9.724715000	0.465001000	-3.320149000
1	-9.640710000	1.324400000	-2.645331000
1	-9.898625000	0.853413000	-4.330162000
1	-10.608869000	-0.109261000	-3.024013000
6	-4.458166000	-3.881767000	-1.468421000
1	-4.356547000	-3.020043000	-2.139996000
1	-4.444877000	-4.777787000	-2.100428000
6	-5.776181000	-3.789807000	-0.702570000
1	-5.846934000	-4.630248000	0.002827000
1	-5.773649000	-2.879663000	-0.084357000
6	-7.009874000	-3.791659000	-1.604081000
1	-6.932148000	-2.982368000	-2.342537000
1	-7.036092000	-4.728277000	-2.178188000
6	-8.311209000	-3.639367000	-0.818731000
1	-8.351052000	-4.415448000	-0.042455000
1	-8.300445000	-2.678256000	-0.285966000
6	-9.562611000	-3.725813000	-1.688638000
1	-9.562942000	-2.945661000	-2.457640000
1	-9.620421000	-4.694812000	-2.197438000
1	-10.470790000	-3.606470000	-1.088613000
6	-3.867782000	-0.196219000	-0.008017000
1	-3.783460000	-0.490205000	1.047184000
6	-5.085025000	0.673476000	-0.137974000
1	-4.911737000	1.744686000	-0.046930000
1	-4.021399000	-1.130204000	-0.562810000
6	-6.338412000	0.245609000	-0.280683000
1	-6.579006000	-0.812142000	-0.370823000
1	-7.176818000	0.936811000	-0.311853000
6	-0.006917000	0.276237000	-0.196664000
1	0.176157000	0.307435000	-1.281845000

1	-0.006580000	1.313497000	0.171734000
6	-1.352220000	-0.370965000	0.084933000
1	-1.365530000	-1.368857000	-0.370497000
1	-1.426999000	-0.510347000	1.171263000
6	-2.539034000	0.453285000	-0.409328000
1	-2.479005000	1.462398000	0.022086000
1	-2.482536000	0.584564000	-1.497306000
8	0.995317000	-0.476387000	0.464529000
14	2.614577000	-0.160748000	0.265678000
6	3.132697000	-0.493674000	-1.494305000
1	2.614510000	0.139786000	-2.219661000
1	4.214556000	-0.339784000	-1.606830000
1	2.903166000	-1.536328000	-1.734378000
6	3.587821000	-1.296374000	1.375296000
1	4.582551000	-1.467810000	0.947935000
1	3.703329000	-0.859901000	2.369275000
1	3.083489000	-2.255745000	1.482000000
6	3.017538000	1.591788000	0.759973000
1	2.399501000	2.341552000	0.260360000
1	2.841197000	1.670151000	1.836662000
1	4.072505000	1.824527000	0.565879000

D_{1b}

E_1 = -5181.23758279 A.U.

At No.	X	Y	Z
6	0.383105000	-1.495454000	3.762546000
6	-0.599532000	-0.703981000	4.365534000
6	1.530554000	-0.860921000	3.291419000
6	-0.417594000	0.678859000	4.462802000
6	1.711968000	0.514893000	3.385001000
6	0.722539000	1.313028000	3.975144000
1	2.292979000	-1.490351000	2.856047000
1	-1.178880000	1.298913000	4.923387000
6	0.249634000	-3.007939000	3.645928000
1	1.012401000	-3.474275000	4.279863000
1	-0.722526000	-3.305739000	4.044596000
6	0.388666000	-3.541072000	2.230728000
6	-0.748403000	-3.804819000	1.464897000
6	1.637473000	-3.790968000	1.650014000
6	-0.665462000	-4.244014000	0.141663000
6	1.721579000	-4.212383000	0.325011000
6	0.587763000	-4.424760000	-0.452549000
1	-1.714793000	-3.652483000	1.931549000
1	2.680347000	-4.356199000	-0.154864000
6	0.726669000	-4.765048000	-1.924358000
1	-0.115165000	-5.384470000	-2.243634000
1	1.644170000	-5.340375000	-2.077337000
6	0.765312000	-3.496546000	-2.754362000
6	-0.398476000	-2.996854000	-3.331026000
6	1.945790000	-2.753378000	-2.878660000
6	-0.421435000	-1.757208000	-3.971018000
6	1.929883000	-1.529253000	-3.539098000
6	0.755095000	-1.012360000	-4.085107000
1	-1.300688000	-3.585504000	-3.216705000
1	2.825287000	-0.925602000	-3.628401000
6	0.764680000	0.335748000	-4.776985000
1	-0.079444000	0.387781000	-5.468431000
1	1.680065000	0.418287000	-5.369910000
6	0.690651000	1.514136000	-3.825765000
6	1.853987000	2.101909000	-3.321923000
6	-0.541088000	2.046543000	-3.440797000
6	1.772012000	3.233809000	-2.516206000

6	-0.623256000	3.153255000	-2.593114000
6	0.547440000	3.786652000	-2.158350000
1	-1.437148000	1.567250000	-3.821305000
1	2.663534000	3.716606000	-2.134397000
6	0.509987000	5.021139000	-1.277932000
1	1.337875000	5.680752000	-1.551166000
1	-0.423390000	5.563318000	-1.447872000
6	0.614734000	4.632632000	0.179391000
6	1.854167000	4.464619000	0.802116000
6	-0.537115000	4.316739000	0.889894000
6	1.912850000	3.928802000	2.085747000
6	-0.478656000	3.775487000	2.172909000
6	0.762963000	3.539324000	2.769967000
1	-1.481848000	4.439288000	0.375630000
1	2.864719000	3.746890000	2.568808000
6	0.878236000	2.817930000	4.097027000
1	1.857860000	3.042513000	4.533476000
1	0.118178000	3.191783000	4.789944000
8	-1.554500000	-1.169813000	-4.471722000
6	-2.793538000	-1.839717000	-4.366512000
1	-2.852785000	-2.394476000	-3.426406000
1	-2.898838000	-2.563313000	-5.188178000
8	-1.753472000	-4.508640000	-0.643712000
6	-3.032945000	-4.266803000	-0.098091000
1	-3.084209000	-3.240290000	0.277768000
1	-3.219996000	-4.944007000	0.749223000
8	-1.705071000	-1.350059000	4.847765000
6	-2.862089000	-0.586588000	5.132900000
1	-2.767763000	-0.101800000	6.115216000
1	-2.972323000	0.206918000	4.383063000
8	-1.593060000	3.398973000	2.873568000
6	-2.835338000	3.586193000	2.219095000
1	-2.990691000	4.659108000	2.028849000
1	-2.836982000	3.065234000	1.252366000
8	-1.796562000	3.689841000	-2.145682000
6	-3.016164000	3.185740000	-2.648694000
1	-3.084503000	3.383850000	-3.729822000
1	-3.074310000	2.099421000	-2.493433000
8	3.052164000	1.535802000	-3.683647000
6	4.163843000	1.831374000	-2.867008000
1	4.415491000	2.900075000	-2.927287000
1	3.922627000	1.608443000	-1.815312000
8	2.961231000	4.819233000	0.073598000
6	4.223934000	4.607238000	0.663765000
1	4.350231000	5.265713000	1.537065000
1	4.301147000	3.572632000	1.018714000
8	2.847159000	1.137413000	2.936010000
6	3.898155000	0.296287000	2.511753000
1	4.166461000	-0.386900000	3.331574000
1	3.579101000	-0.320690000	1.658402000
8	2.744687000	-3.600049000	2.439121000
6	4.005852000	-3.673358000	1.810045000
1	4.147232000	-4.665980000	1.357098000
1	4.060434000	-2.928316000	1.000207000
8	3.073249000	-3.308565000	-2.332335000
6	4.267086000	-2.559187000	-2.346436000
1	4.514064000	-2.254048000	-3.374021000
1	4.147971000	-1.642072000	-1.746498000
6	-4.146807000	3.859449000	-1.889192000
1	-4.153507000	3.452750000	-0.870532000
1	-3.936211000	4.932019000	-1.809245000
6	-5.504902000	3.635609000	-2.563644000

1	-5.625812000	4.339871000	-3.397453000
1	-5.533736000	2.634335000	-3.019183000
6	-6.684153000	3.765088000	-1.599967000
1	-6.548512000	3.051540000	-0.774219000
1	-6.680326000	4.761422000	-1.136456000
6	-8.034791000	3.509547000	-2.267745000
1	-8.218797000	4.282348000	-3.025436000
1	-7.987909000	2.557131000	-2.814189000
6	-9.196906000	3.471074000	-1.277382000
1	-9.066485000	2.661680000	-0.548654000
1	-9.267994000	4.408415000	-0.713718000
1	-10.152797000	3.310675000	-1.787147000
6	-3.911356000	-0.809835000	-4.387180000
1	-3.696997000	-0.055951000	-3.618094000
1	-3.924402000	-0.289582000	-5.352564000
6	-5.265092000	-1.462496000	-4.108516000
1	-5.439019000	-2.271533000	-4.832820000
1	-5.232196000	-1.942887000	-3.118367000
6	-6.445846000	-0.493496000	-4.148337000
1	-6.320366000	0.266645000	-3.363761000
1	-6.446658000	0.048826000	-5.104111000
6	-7.788767000	-1.200074000	-3.962827000
1	-7.923545000	-1.931535000	-4.771347000
1	-7.766493000	-1.779544000	-3.031478000
6	-8.979287000	-0.246557000	-3.929246000
1	-8.880680000	0.469948000	-3.106370000
1	-9.057411000	0.324060000	-4.861892000
1	-9.917858000	-0.790067000	-3.780658000
6	-4.081532000	-4.469158000	-1.176764000
1	-3.784528000	-3.907880000	-2.071749000
1	-4.114087000	-5.526935000	-1.464448000
6	-5.459621000	-4.001630000	-0.702702000
1	-5.673181000	-4.434275000	0.285650000
1	-5.444174000	-2.909626000	-0.559806000
6	-6.589872000	-4.373395000	-1.661527000
1	-6.404573000	-3.916126000	-2.643868000
1	-6.580392000	-5.460202000	-1.824798000
6	-7.970638000	-3.952636000	-1.159322000
1	-8.153378000	-4.410804000	-0.177270000
1	-7.988913000	-2.867824000	-0.994671000
6	-9.093736000	-4.334166000	-2.120289000
1	-8.939583000	-3.875866000	-3.103883000
1	-9.139248000	-5.420157000	-2.262088000
1	-10.066352000	-3.999121000	-1.746660000
6	-4.066071000	-1.517252000	5.074084000
1	-3.905578000	-2.196181000	4.229289000
1	-4.115420000	-2.138705000	5.976013000
6	-5.377756000	-0.762172000	4.858178000
1	-5.621528000	-0.147656000	5.735469000
1	-5.244760000	-0.058586000	4.024199000
6	-6.551370000	-1.683256000	4.524206000
1	-6.244040000	-2.374856000	3.726112000
1	-6.799463000	-2.307587000	5.393686000
6	-7.789108000	-0.917630000	4.059001000
1	-8.088347000	-0.195001000	4.830052000
1	-7.517528000	-0.324560000	3.175533000
6	-8.968741000	-1.823376000	3.714164000
1	-8.683179000	-2.568288000	2.961047000
1	-9.322092000	-2.367062000	4.597622000
1	-9.809592000	-1.249143000	3.310124000
6	-3.965026000	3.043141000	3.071187000
1	-3.872359000	1.951411000	3.139798000

1	-3.893947000	3.443261000	4.089958000
6	-5.303955000	3.417143000	2.434495000
1	-5.402037000	4.511689000	2.415366000
1	-5.290734000	3.096242000	1.383552000
6	-6.525541000	2.820172000	3.128445000
1	-6.427509000	1.726726000	3.169158000
1	-6.563137000	3.161618000	4.172418000
6	-7.830759000	3.182671000	2.419532000
1	-7.907327000	4.275143000	2.336777000
1	-7.789286000	2.807996000	1.387586000
6	-9.075482000	2.634774000	3.113481000
1	-9.035285000	1.542838000	3.198065000
1	-9.169363000	3.039010000	4.127938000
1	-9.985227000	2.894498000	2.561912000
6	5.109548000	1.124837000	2.122172000
1	4.917059000	1.641347000	1.174494000
1	5.275766000	1.894015000	2.886534000
6	6.350762000	0.241793000	1.994181000
1	6.487948000	-0.310588000	2.934576000
1	6.184277000	-0.519817000	1.217369000
6	7.636710000	1.006845000	1.685138000
1	7.575631000	1.448338000	0.680846000
1	7.739192000	1.846668000	2.387097000
6	8.874438000	0.116104000	1.779159000
1	8.894225000	-0.360494000	2.768377000
1	8.778897000	-0.702862000	1.054012000
6	10.189258000	0.856299000	1.551021000
1	10.222711000	1.312961000	0.556336000
1	10.320158000	1.657324000	2.287721000
1	11.044104000	0.177270000	1.636824000
6	5.101371000	-3.425144000	2.829290000
1	4.979126000	-2.427474000	3.267069000
1	4.999813000	-4.147719000	3.647585000
6	6.477517000	-3.552662000	2.176525000
1	6.572855000	-4.553271000	1.730222000
1	6.553911000	-2.839679000	1.342487000
6	7.645882000	-3.321033000	3.131655000
1	7.589290000	-2.301488000	3.537675000
1	7.559890000	-3.999535000	3.991674000
6	9.000299000	-3.524987000	2.453898000
1	9.067162000	-4.560224000	2.092037000
1	9.053495000	-2.887235000	1.562530000
6	10.187288000	-3.222034000	3.364252000
1	10.166126000	-2.179779000	3.703422000
1	10.175955000	-3.861078000	4.254580000
1	11.137404000	-3.385164000	2.845061000
6	5.385400000	-3.431168000	-1.800836000
1	5.109850000	-3.805557000	-0.807886000
1	5.485513000	-4.309060000	-2.450569000
6	6.715600000	-2.681729000	-1.727681000
1	6.904522000	-2.182679000	-2.688905000
1	6.649753000	-1.879032000	-0.977315000
6	7.901105000	-3.589348000	-1.400737000
1	7.736218000	-4.078344000	-0.430910000
1	7.952156000	-4.395200000	-2.146227000
6	9.234354000	-2.844645000	-1.374916000
1	9.378212000	-2.333583000	-2.336342000
1	9.192484000	-2.052297000	-0.615970000
6	10.427931000	-3.753114000	-1.092210000
1	10.320718000	-4.251187000	-0.121921000
1	10.519148000	-4.531988000	-1.857833000
1	11.363854000	-3.185247000	-1.076831000

6	5.363072000	1.026533000	-3.331932000
1	5.080492000	-0.024875000	-3.455510000
1	5.673685000	1.388234000	-4.319591000
6	6.513640000	1.149414000	-2.330899000
1	6.553370000	2.177831000	-1.942147000
1	6.309831000	0.509145000	-1.460101000
6	7.879077000	0.802284000	-2.918594000
1	7.849608000	-0.199886000	-3.368683000
1	8.103695000	1.499741000	-3.737925000
6	8.990918000	0.862046000	-1.876221000
1	8.955282000	1.839524000	-1.377110000
1	8.786607000	0.116359000	-1.098088000
6	10.385620000	0.636008000	-2.450947000
1	10.456356000	-0.339255000	-2.945836000
1	10.634074000	1.403518000	-3.193156000
1	11.149053000	0.668676000	-1.666423000
6	5.308571000	4.869541000	-0.365100000
1	5.134007000	4.218556000	-1.231421000
1	5.238596000	5.903389000	-0.723428000
6	6.692983000	4.592304000	0.221045000
1	6.872262000	5.264404000	1.071993000
1	6.710030000	3.572877000	0.631093000
6	7.834953000	4.737271000	-0.783261000
1	7.661133000	4.063393000	-1.634874000
1	7.839382000	5.754967000	-1.197593000
6	9.199933000	4.426664000	-0.168369000
1	9.400361000	5.136183000	0.645324000
1	9.163820000	3.433424000	0.299400000
6	10.340045000	4.472645000	-1.183073000
1	10.173055000	3.755466000	-1.995703000
1	10.426613000	5.467934000	-1.633712000
1	11.300173000	4.229896000	-0.716140000
6	-8.504923000	-0.276171000	0.428519000
1	-8.610191000	0.554686000	1.141336000
6	-9.801714000	-0.465494000	-0.302268000
1	-10.124227000	0.371461000	-0.923180000
1	-8.279669000	-1.171445000	1.020926000
6	-10.558621000	-1.557938000	-0.241794000
1	-10.271652000	-2.412706000	0.367701000
1	-11.492126000	-1.638616000	-0.791192000
6	-4.883753000	0.555175000	-0.730253000
1	-4.704834000	-0.366343000	-1.307561000
1	-5.188650000	1.314510000	-1.457539000
6	-6.032560000	0.317710000	0.242808000
1	-5.794038000	-0.515815000	0.915384000
1	-6.151522000	1.205009000	0.874028000
6	-7.330642000	0.029647000	-0.503912000
1	-7.582015000	0.889458000	-1.139774000
1	-7.181044000	-0.817231000	-1.185897000
8	-3.702516000	1.011302000	-0.111705000
14	-2.510870000	-0.092956000	0.352977000
6	-2.099204000	-1.150677000	-1.126705000
1	-2.940393000	-1.758567000	-1.472935000
1	-1.778541000	-0.506800000	-1.953546000
1	-1.269742000	-1.829399000	-0.905722000
6	-3.224921000	-1.065754000	1.793447000
1	-2.442309000	-1.560044000	2.376437000
1	-3.727727000	-0.349962000	2.451450000
1	-3.972512000	-1.812736000	1.504683000
6	-0.993008000	0.822427000	0.888639000
1	-0.646551000	1.533878000	0.131828000
1	-1.147940000	1.355721000	1.828025000

1	-0.191570000	0.094020000	1.064464000

D$_{1c}$

E_1 = -5181.22146746 A.U.

At No.	X	Y	Z
6	-0.684187000	0.010401000	3.761833000
6	0.384104000	-0.885688000	3.862894000
6	-1.920501000	-0.485125000	3.346036000
6	0.201423000	-2.229256000	3.525402000
6	-2.105883000	-1.827828000	3.033524000
6	-1.031692000	-2.721468000	3.113080000
1	-2.737587000	0.221723000	3.281046000
1	1.025228000	-2.931246000	3.568518000
6	-0.537881000	1.480336000	4.120680000
1	-1.335279000	1.747907000	4.821952000
1	0.412353000	1.624205000	4.639756000
6	-0.598506000	2.421851000	2.930752000
6	0.575528000	2.863026000	2.313559000
6	-1.823419000	2.875869000	2.424215000
6	0.539850000	3.714690000	1.209430000
6	-1.854038000	3.719288000	1.316581000
6	-0.686391000	4.144350000	0.695372000
1	1.522868000	2.511573000	2.712445000
1	-2.791346000	4.050720000	0.887624000
6	-0.752771000	4.964369000	-0.576245000
1	0.145561000	5.578158000	-0.672223000
1	-1.617881000	5.630292000	-0.541347000
6	-0.865506000	4.027457000	-1.760381000
6	0.278361000	3.485767000	-2.339273000
6	-2.115762000	3.599226000	-2.213016000
6	0.196860000	2.510884000	-3.331415000
6	-2.200091000	2.643471000	-3.222345000
6	-1.055179000	2.082979000	-3.787190000
1	1.234513000	3.822663000	-1.958047000
1	-3.162905000	2.306700000	-3.593243000
6	-1.176593000	1.037349000	-4.872568000
1	-0.358224000	1.166026000	-5.587235000
1	-2.113590000	1.199733000	-5.411732000
6	-1.151868000	-0.389117000	-4.362666000
6	-2.327084000	-1.072537000	-4.063212000
6	0.060793000	-1.067630000	-4.205310000
6	-2.288333000	-2.421841000	-3.723381000
6	0.099500000	-2.389580000	-3.771503000
6	-1.090768000	-3.101970000	-3.563275000
1	0.972786000	-0.516355000	-4.402307000
1	-3.216911000	-2.952393000	-3.542781000
6	-1.081456000	-4.536115000	-3.076079000
1	-1.948520000	-5.061493000	-3.485345000
1	-0.182184000	-5.046100000	-3.429981000
6	-1.122645000	-4.571323000	-1.562798000
6	-2.334278000	-4.479870000	-0.873399000
6	0.059617000	-4.599712000	-0.828402000
6	-2.340241000	-4.396168000	0.515633000
6	0.055793000	-4.516427000	0.563747000
6	-1.157040000	-4.392784000	1.249379000
1	0.987122000	-4.649170000	-1.385701000
1	-3.268559000	-4.287360000	1.062429000
6	-1.194538000	-4.183144000	2.748109000
1	-2.149733000	-4.549963000	3.136493000
1	-0.398132000	-4.761229000	3.224629000
8	1.295638000	1.924955000	-3.904297000
6	2.566761000	2.391144000	-3.506468000

1	2.664469000	2.341256000	-2.412586000
1	2.688521000	3.445020000	-3.801944000
8	1.639473000	4.176846000	0.541088000
6	2.932924000	3.930831000	1.038462000
1	3.116795000	2.848910000	1.109229000
1	3.046480000	4.353588000	2.048431000
8	1.581863000	-0.389108000	4.309972000
6	2.588730000	-1.331499000	4.619834000
1	2.208654000	-2.068731000	5.341562000
1	2.870000000	-1.878912000	3.710365000
8	1.195405000	-4.544175000	1.322944000
6	2.422730000	-4.491907000	0.629679000
1	2.516396000	-5.351138000	-0.051654000
1	2.457158000	-3.578137000	0.017380000
8	1.261381000	-3.073554000	-3.543165000
6	2.455762000	-2.327779000	-3.453648000
1	2.681345000	-1.844313000	-4.416037000
1	2.343845000	-1.531117000	-2.701199000
8	-3.545274000	-0.421777000	-4.107188000
6	-4.159743000	-0.316699000	-2.828309000
1	-4.133626000	-1.289092000	-2.317263000
1	-3.593733000	0.390461000	-2.203217000
8	-3.475426000	-4.465131000	-1.631823000
6	-4.675318000	-4.078823000	-1.000433000
1	-4.973833000	-4.825129000	-0.248073000
1	-4.527846000	-3.121236000	-0.477439000
8	-3.314547000	-2.351759000	2.659147000
6	-4.438273000	-1.503223000	2.713092000
1	-4.520585000	-1.057057000	3.716141000
1	-4.331763000	-0.678442000	1.990819000
8	-2.963115000	2.462420000	3.067838000
6	-4.200980000	2.779359000	2.466469000
1	-4.312637000	3.870581000	2.380858000
1	-4.241960000	2.361271000	1.447577000
8	-3.199899000	4.182163000	-1.615086000
6	-4.477126000	3.616104000	-1.803065000
1	-4.773327000	3.662316000	-2.861818000
1	-4.467646000	2.556283000	-1.505290000
6	3.588387000	-3.269222000	-3.086437000
1	3.349488000	-3.785503000	-2.148420000
1	3.668379000	-4.039932000	-3.862670000
6	4.910067000	-2.515187000	-2.954847000
1	5.043521000	-1.864513000	-3.831869000
1	4.866617000	-1.847347000	-2.081282000
6	6.133975000	-3.419648000	-2.826254000
1	6.047826000	-4.032833000	-1.918971000
1	6.163782000	-4.118840000	-3.673477000
6	7.429637000	-2.613054000	-2.781361000
1	7.513009000	-2.019172000	-3.701785000
1	7.359073000	-1.890602000	-1.959238000
6	8.683833000	-3.464765000	-2.607417000
1	8.642392000	-4.041065000	-1.676609000
1	8.795498000	-4.175292000	-3.434448000
1	9.584426000	-2.841643000	-2.577876000
6	3.652164000	1.531578000	-4.134773000
1	3.609840000	0.528900000	-3.689198000
1	3.457765000	1.414013000	-5.206830000
6	5.027129000	2.165927000	-3.891982000
1	5.173239000	3.000104000	-4.591051000
1	5.033566000	2.612446000	-2.888892000
6	6.210653000	1.202377000	-3.990122000
1	6.022456000	0.323050000	-3.358998000

1	6.306639000	0.825368000	-5.017899000
6	7.522465000	1.854443000	-3.550445000
1	7.717696000	2.735684000	-4.176457000
1	7.401469000	2.232831000	-2.525068000
6	8.725011000	0.915028000	-3.596987000
1	8.582739000	0.059395000	-2.927067000
1	8.881165000	0.521096000	-4.607874000
1	9.641545000	1.430837000	-3.291137000
6	3.906146000	4.567607000	0.056532000
1	3.737279000	4.111756000	-0.928770000
1	3.661386000	5.631262000	-0.045663000
6	5.366645000	4.381626000	0.460600000
1	5.581836000	4.955658000	1.372047000
1	5.525158000	3.331370000	0.720510000
6	6.364420000	4.758501000	-0.635475000
1	6.042008000	4.316552000	-1.589787000
1	6.364817000	5.846220000	-0.788371000
6	7.781717000	4.273324000	-0.327018000
1	8.088028000	4.638846000	0.663067000
1	7.766146000	3.176268000	-0.253253000
6	8.811172000	4.693479000	-1.372810000
1	8.512173000	4.358306000	-2.372361000
1	8.914557000	5.784101000	-1.406358000
1	9.796044000	4.266306000	-1.156537000
6	3.806287000	-0.602403000	5.166346000
1	3.878215000	0.364118000	4.656219000
1	3.681983000	-0.389042000	6.234453000
6	5.079563000	-1.407603000	4.906199000
1	4.994015000	-2.410198000	5.346994000
1	5.156145000	-1.568107000	3.821829000
6	6.371277000	-0.739169000	5.387206000
1	6.289126000	0.351176000	5.273710000
1	6.517016000	-0.917589000	6.460639000
6	7.585170000	-1.230008000	4.596503000
1	7.686211000	-2.316718000	4.719356000
1	7.387719000	-1.065605000	3.527942000
6	8.890067000	-0.539030000	4.981163000
1	8.823106000	0.542697000	4.813042000
1	9.129658000	-0.697171000	6.038869000
1	9.729092000	-0.919137000	4.387722000
6	3.578460000	-4.496541000	1.612435000
1	3.429834000	-3.691406000	2.341629000
1	3.583183000	-5.436073000	2.177377000
6	4.899561000	-4.310065000	0.862987000
1	5.082897000	-5.193259000	0.233836000
1	4.805902000	-3.459391000	0.172575000
6	6.116832000	-4.058538000	1.749928000
1	5.973085000	-3.123924000	2.307932000
1	6.218046000	-4.860730000	2.494502000
6	7.396386000	-3.946204000	0.922486000
1	7.593475000	-4.908430000	0.429285000
1	7.221502000	-3.214590000	0.124322000
6	8.616682000	-3.519786000	1.732769000
1	8.462707000	-2.522534000	2.158739000
1	8.809041000	-4.211623000	2.561330000
1	9.516747000	-3.486332000	1.108361000
6	-5.686429000	-2.311340000	2.408027000
1	-5.634672000	-2.695940000	1.382429000
1	-5.716042000	-3.181705000	3.074466000
6	-6.945101000	-1.465671000	2.591315000
1	-6.991726000	-1.116109000	3.632677000
1	-6.876158000	-0.560934000	1.968322000

6	-8.240375000	-2.200528000	2.253507000
1	-8.235628000	-2.481462000	1.190989000
1	-8.289425000	-3.144013000	2.814427000
6	-9.481933000	-1.361576000	2.555788000
1	-9.559092000	-1.216383000	3.641797000
1	-9.351729000	-0.360225000	2.125448000
6	-10.771745000	-1.977251000	2.020933000
1	-10.734121000	-2.072290000	0.929276000
1	-10.934988000	-2.978200000	2.436525000
1	-11.641501000	-1.361581000	2.273345000
6	-5.330534000	2.225209000	3.315420000
1	-5.202304000	1.143753000	3.441300000
1	-5.274923000	2.671492000	4.315540000
6	-6.688513000	2.521412000	2.677812000
1	-6.754017000	3.594322000	2.445341000
1	-6.758833000	1.997593000	1.713046000
6	-7.878542000	2.133433000	3.553927000
1	-7.838833000	1.058705000	3.775877000
1	-7.800078000	2.651158000	4.520147000
6	-9.222515000	2.468001000	2.908775000
1	-9.246278000	3.537516000	2.660041000
1	-9.306166000	1.933227000	1.953789000
6	-10.419925000	2.119203000	3.788552000
1	-10.437167000	1.048458000	4.020879000
1	-10.383455000	2.665454000	4.738022000
1	-11.363028000	2.370520000	3.292318000
6	-5.451496000	4.416536000	-0.955869000
1	-5.115168000	4.396620000	0.088800000
1	-5.408893000	5.463570000	-1.279886000
6	-6.882877000	3.895304000	-1.046707000
1	-7.173583000	3.792465000	-2.102198000
1	-6.932157000	2.886904000	-0.615079000
6	-7.891984000	4.795560000	-0.336051000
1	-7.591942000	4.923020000	0.713919000
1	-7.864317000	5.797935000	-0.785900000
6	-9.317338000	4.250063000	-0.388246000
1	-9.604715000	4.086875000	-1.436125000
1	-9.338010000	3.265157000	0.092958000
6	-10.336057000	5.165546000	0.285854000
1	-10.077524000	5.334384000	1.337847000
1	-10.373511000	6.144262000	-0.206073000
1	-11.341738000	4.734022000	0.254383000
6	-5.600089000	0.125999000	-3.005143000
1	-5.630301000	1.130310000	-3.448253000
1	-6.079721000	-0.550482000	-3.724259000
6	-6.369295000	0.105316000	-1.684695000
1	-6.259051000	-0.884807000	-1.220241000
1	-5.919832000	0.818175000	-0.977335000
6	-7.856710000	0.415191000	-1.845207000
1	-7.981866000	1.403842000	-2.307795000
1	-8.304006000	-0.307807000	-2.541468000
6	-8.616648000	0.372925000	-0.522321000
1	-8.431883000	-0.593656000	-0.036674000
1	-8.202774000	1.132000000	0.155776000
6	-10.119182000	0.588417000	-0.678671000
1	-10.330082000	1.542698000	-1.173599000
1	-10.568616000	-0.205790000	-1.285387000
1	-10.626758000	0.594083000	0.292483000
6	-5.757251000	-3.943657000	-2.055970000
1	-5.492894000	-3.129790000	-2.743993000
1	-5.791454000	-4.863210000	-2.651965000
6	-7.122726000	-3.669223000	-1.429015000

1	-7.429566000	-4.530707000	-0.819672000
1	-7.042115000	-2.821273000	-0.733544000
6	-8.204292000	-3.357223000	-2.459918000
1	-7.887059000	-2.492212000	-3.059820000
1	-8.298082000	-4.196993000	-3.162368000
6	-9.562797000	-3.064555000	-1.825584000
1	-9.902227000	-3.947980000	-1.268600000
1	-9.442885000	-2.265344000	-1.082953000
6	-10.624190000	-2.655057000	-2.843324000
1	-10.321514000	-1.748574000	-3.381272000
1	-10.783348000	-3.443816000	-3.587345000
1	-11.585080000	-2.451734000	-2.358756000
6	10.613553000	2.089312000	1.175157000
1	10.867346000	1.601070000	2.127573000
6	11.873253000	2.549358000	0.500058000
1	12.549866000	1.759838000	0.169516000
1	9.988283000	2.956934000	1.422919000
6	12.205676000	3.819257000	0.283295000
1	11.561180000	4.637010000	0.599740000
1	13.134080000	4.091576000	-0.210054000
6	7.731034000	-0.332783000	0.168927000
1	7.510740000	0.144755000	-0.799136000
1	8.332054000	-1.225122000	-0.044343000
6	8.541975000	0.625444000	1.031521000
1	7.928214000	1.497240000	1.289474000
1	8.796208000	0.130805000	1.978015000
6	9.807395000	1.102971000	0.322230000
1	10.436425000	0.239694000	0.063994000
1	9.539360000	1.584855000	-0.628458000
8	6.537206000	-0.775053000	0.782593000
14	5.168621000	0.184888000	0.932561000
6	4.929987000	1.133830000	-0.665916000
1	5.769292000	1.790976000	-0.913505000
1	4.804500000	0.426212000	-1.492930000
1	4.024813000	1.748685000	-0.624354000
6	5.346198000	1.302305000	2.433815000
1	4.390577000	1.763702000	2.708219000
1	5.676590000	0.696262000	3.280813000
1	6.080434000	2.102404000	2.307687000
6	3.710771000	-0.955420000	1.171626000
1	3.385537000	-1.390965000	0.220373000
1	3.978698000	-1.777359000	1.838616000
1	2.855840000	-0.430931000	1.614285000

D_{1d}

E_1 = -5181.21776666 A.U.

At No.	X	Y	Z
6	-1.918949000	2.080738000	4.079156000
6	-0.726694000	1.376261000	4.272361000
6	-3.122195000	1.378770000	4.101037000
6	-0.752662000	-0.007390000	4.437409000
6	-3.148490000	-0.001335000	4.286283000
6	-1.953551000	-0.713021000	4.434242000
1	-4.037126000	1.941363000	3.950734000
1	0.165989000	-0.570122000	4.568177000
6	-1.897810000	3.566999000	3.794247000
1	-2.863437000	3.999387000	4.069340000
1	-1.129318000	4.050619000	4.402959000
6	-1.620058000	3.842362000	2.330915000
6	-0.329309000	4.094245000	1.872070000
6	-2.651178000	3.824635000	1.386217000
6	-0.060440000	4.292782000	0.520340000
6	-2.390919000	4.099602000	0.050919000

6	-1.096239000	4.310584000	-0.415006000
1	0.487300000	4.123436000	2.585349000
1	-3.204097000	4.134222000	-0.663730000
6	-0.840360000	4.508234000	-1.894931000
1	0.164597000	4.913287000	-2.033908000
1	-1.553211000	5.237098000	-2.291505000
6	-0.969444000	3.205243000	-2.657594000
6	0.147683000	2.397030000	-2.864598000
6	-2.209225000	2.754130000	-3.120259000
6	0.047808000	1.162857000	-3.504214000
6	-2.308296000	1.526753000	-3.769841000
6	-1.195448000	0.716074000	-3.966497000
1	1.106483000	2.757109000	-2.506669000
1	-3.264774000	1.157128000	-4.120503000
6	-1.353286000	-0.635018000	-4.630430000
1	-0.462362000	-0.859710000	-5.223254000
1	-2.205298000	-0.597755000	-5.314929000
6	-1.564662000	-1.742107000	-3.619092000
6	-2.840636000	-2.068460000	-3.147446000
6	-0.470092000	-2.433132000	-3.105407000
6	-2.995204000	-3.103709000	-2.227213000
6	-0.622229000	-3.431989000	-2.146809000
6	-1.901357000	-3.799393000	-1.720328000
1	0.512210000	-2.146802000	-3.462183000
1	-3.972603000	-3.379801000	-1.849979000
6	-2.109709000	-4.863193000	-0.662329000
1	-3.058746000	-5.373945000	-0.841779000
1	-1.311106000	-5.607155000	-0.723206000
6	-2.106520000	-4.236707000	0.714903000
6	-3.288293000	-3.813164000	1.340661000
6	-0.904052000	-3.967416000	1.347056000
6	-3.240829000	-3.204925000	2.591836000
6	-0.848598000	-3.266478000	2.547621000
6	-2.019644000	-2.914058000	3.212020000
1	0.016361000	-4.275952000	0.862341000
1	-4.149105000	-2.906259000	3.101481000
6	-1.968622000	-2.220847000	4.559131000
1	-2.833859000	-2.529384000	5.152441000
1	-1.069103000	-2.543149000	5.089542000
8	1.113239000	0.336593000	-3.735250000
6	2.356882000	0.634236000	-3.136341000
1	2.252011000	0.655385000	-2.039870000
1	2.717313000	1.623705000	-3.456391000
8	1.227992000	4.521437000	0.092342000
6	2.102854000	3.407334000	0.168749000
1	1.607643000	2.510879000	-0.232994000
1	2.368545000	3.194796000	1.215094000
8	0.417550000	2.125211000	4.309922000
6	1.651368000	1.495896000	4.036128000
1	1.890282000	0.743878000	4.802480000
1	1.595668000	0.979069000	3.065574000
8	0.379538000	-2.920572000	3.072643000
6	1.112605000	-2.045659000	2.223675000
1	1.076852000	-2.408984000	1.187918000
1	0.645614000	-1.049602000	2.230609000
8	0.433465000	-4.094649000	-1.578252000
6	1.727401000	-3.572775000	-1.788971000
1	2.028209000	-3.706951000	-2.839863000
1	1.736195000	-2.492308000	-1.576004000
8	-3.891799000	-1.335690000	-3.634171000
6	-5.160397000	-1.587016000	-3.069713000
1	-5.458878000	-2.629139000	-3.261700000

1	-5.119360000	-1.450519000	-1.977810000
8	-4.445798000	-4.045179000	0.649680000
6	-5.641243000	-3.446409000	1.100817000
1	-5.943586000	-3.875605000	2.068517000
1	-5.493003000	-2.364732000	1.247698000
8	-4.298224000	-0.739230000	4.355510000
6	-5.511059000	-0.146972000	3.948753000
1	-5.734613000	0.744041000	4.553696000
1	-5.436173000	0.173267000	2.898463000
8	-3.939107000	3.580225000	1.807219000
6	-4.610859000	2.524196000	1.134001000
1	-4.444819000	2.586749000	0.051058000
1	-4.203375000	1.559077000	1.469612000
8	-3.287571000	3.571342000	-2.904370000
6	-4.576064000	3.014500000	-3.058627000
1	-4.781710000	2.806447000	-4.119687000
1	-4.635903000	2.058700000	-2.515020000
6	2.696823000	-4.293439000	-0.870228000
1	2.445952000	-4.058850000	0.172910000
1	2.567062000	-5.375308000	-0.990265000
6	4.145861000	-3.903382000	-1.160943000
1	4.427158000	-4.269293000	-2.158500000
1	4.233457000	-2.807179000	-1.203148000
6	5.126896000	-4.441908000	-0.122412000
1	4.882224000	-4.007999000	0.857212000
1	4.987389000	-5.526950000	-0.017498000
6	6.590016000	-4.153371000	-0.456404000
1	6.843992000	-4.636765000	-1.409292000
1	6.724098000	-3.075300000	-0.616956000
6	7.549986000	-4.628556000	0.631151000
1	7.327954000	-4.141312000	1.588345000
1	7.466153000	-5.710490000	0.784901000
1	8.588926000	-4.402101000	0.372289000
6	3.339674000	-0.439495000	-3.572823000
1	2.949585000	-1.420637000	-3.275387000
1	3.384331000	-0.438645000	-4.668724000
6	4.739924000	-0.244670000	-2.992978000
1	5.108090000	0.760850000	-3.242624000
1	4.693709000	-0.290625000	-1.895835000
6	5.731561000	-1.289672000	-3.504969000
1	5.365907000	-2.292112000	-3.244309000
1	5.757781000	-1.251712000	-4.603069000
6	7.153857000	-1.124580000	-2.968876000
1	7.536322000	-0.133106000	-3.248050000
1	7.135987000	-1.146221000	-1.869962000
6	8.095584000	-2.208802000	-3.488448000
1	7.753119000	-3.202218000	-3.177481000
1	8.137827000	-2.197137000	-4.583587000
1	9.114162000	-2.075145000	-3.112473000
6	3.343720000	3.757438000	-0.631179000
1	3.063432000	3.880769000	-1.686130000
1	3.700178000	4.737093000	-0.289509000
6	4.465113000	2.730863000	-0.501638000
1	4.763838000	2.651886000	0.551932000
1	4.103075000	1.735298000	-0.794629000
6	5.681816000	3.101587000	-1.348106000
1	5.395545000	3.091630000	-2.410123000
1	5.972552000	4.137988000	-1.124176000
6	6.891711000	2.192305000	-1.143499000
1	7.217776000	2.251591000	-0.096255000
1	6.600437000	1.149116000	-1.314110000
6	8.050809000	2.557988000	-2.066498000

1	7.758286000	2.461109000	-3.119036000
1	8.370661000	3.593876000	-1.904976000
1	8.918304000	1.912799000	-1.900988000
6	2.725083000	2.571278000	4.010293000
1	2.396812000	3.373856000	3.339695000
1	2.814813000	3.017305000	5.007806000
6	4.074669000	2.034379000	3.539805000
1	4.454937000	1.296118000	4.259817000
1	3.937915000	1.494398000	2.591750000
6	5.119550000	3.128283000	3.323062000
1	4.743508000	3.833331000	2.566829000
1	5.250133000	3.711898000	4.244463000
6	6.468215000	2.569383000	2.869377000
1	6.903162000	1.968537000	3.679292000
1	6.302066000	1.876093000	2.035540000
6	7.457041000	3.645890000	2.430306000
1	7.057262000	4.217724000	1.583813000
1	7.662737000	4.353194000	3.241708000
1	8.409806000	3.205602000	2.116394000
6	2.555782000	-1.989050000	2.683780000
1	2.627326000	-1.481502000	3.654688000
1	2.907021000	-3.017475000	2.836052000
6	3.435488000	-1.294061000	1.647631000
1	3.322406000	-1.811514000	0.683941000
1	3.078861000	-0.267212000	1.477643000
6	4.915375000	-1.266406000	2.017480000
1	5.062091000	-0.678927000	2.933801000
1	5.252882000	-2.286083000	2.250495000
6	5.776245000	-0.693711000	0.897011000
1	5.583777000	-1.262874000	-0.022579000
1	5.455488000	0.334250000	0.685326000
6	7.268127000	-0.718540000	1.200239000
1	7.504480000	-0.134305000	2.096609000
1	7.620155000	-1.742875000	1.365546000
1	7.843714000	-0.300188000	0.369522000
6	-6.614557000	-1.174919000	4.122203000
1	-6.319201000	-2.103707000	3.619389000
1	-6.714211000	-1.411639000	5.188406000
6	-7.946021000	-0.688726000	3.556260000
1	-8.182967000	0.306219000	3.960352000
1	-7.850451000	-0.561882000	2.469084000
6	-9.105670000	-1.638512000	3.846684000
1	-8.837053000	-2.651702000	3.515581000
1	-9.263091000	-1.703309000	4.932257000
6	-10.401992000	-1.213862000	3.160627000
1	-10.656003000	-0.189489000	3.466404000
1	-10.232248000	-1.177644000	2.077464000
6	-11.573676000	-2.145000000	3.459826000
1	-11.340141000	-3.175220000	3.166042000
1	-11.809233000	-2.152590000	4.530135000
1	-12.474570000	-1.839018000	2.918093000
6	-6.095329000	2.639191000	1.425061000
1	-6.273327000	2.513849000	2.501337000
1	-6.412733000	3.658860000	1.171476000
6	-6.922259000	1.630308000	0.630568000
1	-6.682603000	1.729349000	-0.437618000
1	-6.634237000	0.605227000	0.907471000
6	-8.427106000	1.810055000	0.822116000
1	-8.680869000	1.697151000	1.885157000
1	-8.705252000	2.837446000	0.548230000
6	-9.254855000	0.831058000	-0.005093000
1	-8.955716000	0.913022000	-1.057921000

1	-9.008349000	-0.195558000	0.297048000
6	-10.758566000	1.056887000	0.121310000
1	-11.084605000	0.951406000	1.162024000
1	-11.033005000	2.063927000	-0.213357000
1	-11.325432000	0.339730000	-0.482504000
6	-5.595200000	3.995330000	-2.508353000
1	-5.422335000	4.128930000	-1.432267000
1	-5.440775000	4.974457000	-2.976419000
6	-7.025719000	3.514250000	-2.743945000
1	-7.237390000	3.492819000	-3.821979000
1	-7.123774000	2.475594000	-2.396332000
6	-8.068544000	4.371284000	-2.030398000
1	-7.846615000	4.380057000	-0.953497000
1	-7.983221000	5.413541000	-2.367720000
6	-9.496756000	3.875776000	-2.248878000
1	-9.732938000	3.905577000	-3.320919000
1	-9.554319000	2.820282000	-1.954611000
6	-10.536184000	4.674928000	-1.467317000
1	-10.341914000	4.621037000	-0.389328000
1	-10.519989000	5.732454000	-1.754312000
1	-11.547708000	4.294347000	-1.644564000
6	-6.188236000	-0.647993000	-3.673796000
1	-5.936649000	0.389165000	-3.419550000
1	-6.151557000	-0.729185000	-4.766707000
6	-7.586738000	-0.997497000	-3.164804000
1	-7.816922000	-2.035474000	-3.445676000
1	-7.594107000	-0.971740000	-2.064323000
6	-8.696729000	-0.087309000	-3.685650000
1	-8.539874000	0.934879000	-3.314330000
1	-8.643033000	-0.022904000	-4.781147000
6	-10.084305000	-0.578642000	-3.271172000
1	-10.288263000	-1.532377000	-3.776269000
1	-10.080630000	-0.801479000	-2.195958000
6	-11.198556000	0.417833000	-3.577821000
1	-11.046629000	1.354656000	-3.029313000
1	-11.230464000	0.659410000	-4.646430000
1	-12.177874000	0.018899000	-3.293331000
6	-6.710056000	-3.711277000	0.053637000
1	-6.393024000	-3.266910000	-0.898773000
1	-6.773735000	-4.794043000	-0.108947000
6	-8.079135000	-3.162715000	0.451593000
1	-8.370182000	-3.573194000	1.428882000
1	-8.013277000	-2.073495000	0.585465000
6	-9.167836000	-3.488901000	-0.571477000
1	-8.888129000	-3.072007000	-1.548579000
1	-9.217059000	-4.578674000	-0.705907000
6	-10.549340000	-2.967963000	-0.178453000
1	-10.808991000	-3.345184000	0.819478000
1	-10.512029000	-1.874643000	-0.089940000
6	-11.641353000	-3.357126000	-1.171922000
1	-11.417976000	-2.970492000	-2.172481000
1	-11.732685000	-4.446597000	-1.248999000
1	-12.615037000	-2.959251000	-0.867928000
6	17.567275000	-0.310735000	0.438866000
1	17.736441000	-1.170673000	-0.225039000
6	18.738945000	0.624803000	0.353844000
1	18.943071000	1.040974000	-0.633878000
1	17.484439000	-0.710517000	1.457396000
6	19.507620000	0.982991000	1.378257000
1	19.336885000	0.594061000	2.380024000
1	20.337343000	1.673381000	1.257100000
6	13.751067000	0.075660000	-0.297720000

1	13.556141000	0.925102000	0.375937000
1	13.833073000	0.481356000	-1.318890000
6	15.058875000	-0.594880000	0.091736000
1	14.949002000	-1.009279000	1.101568000
1	15.228726000	-1.444267000	-0.581710000
6	16.247406000	0.363755000	0.041987000
1	16.344055000	0.780114000	-0.970800000
1	16.069350000	1.216496000	0.710537000
8	12.701383000	-0.865489000	-0.221294000
14	11.124468000	-0.498482000	-0.638267000
6	11.049180000	0.018352000	-2.445588000
1	11.522436000	-0.736652000	-3.083685000
1	10.012127000	0.132635000	-2.781669000
1	11.557374000	0.973268000	-2.622110000
6	10.499685000	0.895280000	0.462026000
1	9.477661000	1.194534000	0.206503000
1	10.502535000	0.588341000	1.514007000
1	11.131229000	1.786758000	0.369824000
6	10.179476000	-2.089996000	-0.361203000
1	10.643858000	-2.910752000	-0.918856000
1	10.176309000	-2.362106000	0.699784000
1	9.137851000	-2.007295000	-0.688972000

D_{1a}’

E_1 = -5181.25897126 A.U.

At No.	X	Y	Z
6	-0.267746000	2.877360000	-3.169271000
6	-1.506698000	2.310936000	-3.479560000
6	0.886673000	2.123454000	-3.380017000
6	-1.564395000	1.035303000	-4.034974000
6	0.824263000	0.835076000	-3.906614000
6	-0.414360000	0.287884000	-4.260695000
1	1.835292000	2.565999000	-3.094181000
1	-2.511736000	0.577374000	-4.292190000
6	-0.169204000	4.243202000	-2.525179000
1	0.745943000	4.740363000	-2.854396000
1	-1.017615000	4.859530000	-2.834663000
6	-0.165273000	4.105245000	-1.019120000
6	-1.353957000	3.807386000	-0.359243000
6	1.013946000	4.176168000	-0.276544000
6	-1.388364000	3.564081000	1.010218000
6	0.970484000	3.989003000	1.102721000
6	-0.215212000	3.681059000	1.761875000
1	-2.246300000	3.742290000	-0.967520000
1	1.872066000	4.039324000	1.698212000
6	-0.210781000	3.449413000	3.255682000
1	-1.113259000	3.882695000	3.697551000
1	0.648709000	3.971361000	3.688969000
6	-0.145784000	1.981828000	3.613837000
6	-1.302814000	1.296730000	3.984147000
6	1.050098000	1.271214000	3.541304000
6	-1.268412000	-0.060386000	4.289540000
6	1.088366000	-0.085385000	3.852388000
6	-0.058105000	-0.768947000	4.239885000
1	-2.232788000	1.851758000	4.003138000
1	2.021461000	-0.634522000	3.753783000
6	-0.024238000	-2.258188000	4.503167000
1	-0.814811000	-2.524705000	5.209237000
1	0.933496000	-2.526891000	4.954032000
6	-0.214165000	-3.030590000	3.215554000
6	0.882542000	-3.520657000	2.502834000
6	-1.488380000	-3.204091000	2.682765000
6	0.682755000	-4.195094000	1.300442000

6	-1.687727000	-3.858020000	1.468823000
6	-0.593216000	-4.378547000	0.773065000
1	-2.320001000	-2.787936000	3.238004000
1	1.517411000	-4.563408000	0.717755000
6	-0.769256000	-5.064348000	-0.564186000
1	0.016469000	-5.814934000	-0.689053000
1	-1.731587000	-5.581923000	-0.589216000
6	-0.706217000	-4.069929000	-1.702116000
6	0.522537000	-3.658898000	-2.229064000
6	-1.874598000	-3.503586000	-2.206706000
6	0.554279000	-2.717507000	-3.253879000
6	-1.839216000	-2.547640000	-3.220126000
6	-0.612511000	-2.156444000	-3.763958000
1	-2.812093000	-3.813779000	-1.760559000
1	1.492693000	-2.367093000	-3.664454000
6	-0.524668000	-1.102791000	-4.844894000
1	0.347940000	-1.306265000	-5.471831000
1	-1.412005000	-1.153911000	-5.481529000
8	-2.379079000	-0.773823000	4.644124000
6	-3.633435000	-0.144763000	4.482430000
1	-3.717112000	0.248214000	3.459612000
1	-3.725825000	0.705166000	5.175044000
8	-2.527928000	3.208415000	1.682594000
6	-3.721657000	3.166588000	0.927016000
1	-3.566541000	2.578482000	0.015670000
1	-4.003617000	4.187956000	0.625877000
8	-2.624393000	3.062230000	-3.216917000
6	-3.871366000	2.411132000	-3.334773000
1	-4.064504000	2.148675000	-4.386361000
1	-3.856461000	1.478822000	-2.754426000
8	-2.957223000	-1.934552000	-3.725944000
6	-4.207469000	-2.267405000	-3.161709000
1	-4.431878000	-3.331698000	-3.330045000
1	-4.184855000	-2.099469000	-2.075981000
8	-2.924336000	-4.023003000	0.903595000
6	-3.942758000	-3.153910000	1.355200000
1	-4.176766000	-3.357058000	2.410542000
1	-3.599219000	-2.112512000	1.275407000
8	2.115851000	-3.307927000	3.056767000
6	3.245687000	-3.671507000	2.295000000
1	3.182634000	-4.727231000	1.993371000
1	3.289956000	-3.061022000	1.379120000
8	1.648271000	-4.232161000	-1.704648000
6	2.894281000	-3.730450000	-2.146734000
1	2.973671000	-3.831479000	-3.239388000
1	2.978391000	-2.663381000	-1.895844000
8	1.918174000	0.043057000	-4.116650000
6	3.199936000	0.567253000	-3.831975000
1	3.374686000	1.473371000	-4.431823000
1	3.275916000	0.839750000	-2.770911000
8	2.171245000	4.426284000	-0.965604000
6	3.329594000	4.690497000	-0.204972000
1	3.154336000	5.548811000	0.462285000
1	3.570129000	3.824657000	0.430078000
8	2.175723000	1.914582000	3.070787000
6	3.206073000	2.162764000	4.012693000
1	2.800926000	2.698026000	4.884010000
1	3.630915000	1.214153000	4.374404000
6	-5.200837000	-3.363920000	0.533551000
1	-4.950111000	-3.369204000	-0.532847000
1	-5.625505000	-4.348740000	0.764105000
6	-6.216759000	-2.258517000	0.832357000

1	-6.203250000	-2.033028000	1.909228000
1	-5.900196000	-1.332334000	0.330931000
6	-7.646922000	-2.609509000	0.430391000
1	-7.683881000	-2.866157000	-0.637193000
1	-7.959071000	-3.512075000	0.975016000
6	-8.633528000	-1.481374000	0.720141000
1	-8.525620000	-1.180023000	1.770599000
1	-8.360162000	-0.599024000	0.124299000
6	-10.086089000	-1.862827000	0.448244000
1	-10.224492000	-2.171931000	-0.593482000
1	-10.395873000	-2.698493000	1.086468000
1	-10.763743000	-1.024690000	0.641500000
6	-4.738752000	-1.150716000	4.741348000
1	-4.642416000	-1.978070000	4.027851000
1	-4.620612000	-1.577314000	5.744341000
6	-6.109621000	-0.491202000	4.594723000
1	-6.224054000	0.288553000	5.360867000
1	-6.156874000	0.026707000	3.626472000
6	-7.283533000	-1.464061000	4.684492000
1	-7.207594000	-2.200859000	3.871688000
1	-7.226069000	-2.036792000	5.620366000
6	-8.634538000	-0.753152000	4.597337000
1	-8.769006000	-0.121132000	5.485025000
1	-8.622152000	-0.067514000	3.739990000
6	-9.812633000	-1.714455000	4.462566000
1	-9.713857000	-2.328752000	3.559410000
1	-9.868836000	-2.394000000	5.320637000
1	-10.763096000	-1.174885000	4.397054000
6	-4.828741000	2.527322000	1.743919000
1	-4.572812000	1.476875000	1.932515000
1	-4.905701000	3.023941000	2.719129000
6	-6.159382000	2.617644000	0.996557000
1	-6.375195000	3.675466000	0.788181000
1	-6.061590000	2.129916000	0.015055000
6	-7.347698000	2.010034000	1.740078000
1	-7.202130000	0.926307000	1.846956000
1	-7.390734000	2.419379000	2.759466000
6	-8.674089000	2.278036000	1.028726000
1	-8.765543000	3.357899000	0.848199000
1	-8.652642000	1.805795000	0.037231000
6	-9.898180000	1.793418000	1.800910000
1	-9.861722000	0.711973000	1.966595000
1	-9.960161000	2.276902000	2.782733000
1	-10.822007000	2.016528000	1.256714000
6	-4.977094000	3.315079000	-2.822923000
1	-4.784003000	3.574532000	-1.774433000
1	-4.976978000	4.252885000	-3.391111000
6	-6.329423000	2.614242000	-2.954205000
1	-6.478771000	2.320766000	-4.003554000
1	-6.311118000	1.675311000	-2.379117000
6	-7.522941000	3.452316000	-2.501722000
1	-7.422233000	3.692896000	-1.434820000
1	-7.522192000	4.411958000	-3.036966000
6	-8.851804000	2.737567000	-2.742658000
1	-8.950772000	2.525917000	-3.816141000
1	-8.829334000	1.758904000	-2.243667000
6	-10.067479000	3.525879000	-2.263661000
1	-10.013212000	3.715426000	-1.185847000
1	-10.131893000	4.495991000	-2.769549000
1	-10.996250000	2.980739000	-2.461985000
6	-5.275820000	-1.393840000	-3.794889000
1	-4.955182000	-0.345572000	-3.746898000

1	-5.371976000	-1.648781000	-4.857301000
6	-6.615873000	-1.561815000	-3.078708000
1	-6.825809000	-2.633406000	-2.945337000
1	-6.538251000	-1.140203000	-2.065463000
6	-7.794362000	-0.919441000	-3.808247000
1	-7.594513000	0.148647000	-3.969287000
1	-7.890232000	-1.369412000	-4.806276000
6	-9.109915000	-1.082072000	-3.048530000
1	-9.264761000	-2.147045000	-2.826427000
1	-9.026203000	-0.579060000	-2.075772000
6	-10.321826000	-0.539370000	-3.801333000
1	-10.205351000	0.527795000	-4.019384000
1	-10.457122000	-1.061014000	-4.755663000
1	-11.239550000	-0.663884000	-3.217053000
6	4.226808000	-0.499333000	-4.165976000
1	4.068218000	-1.362104000	-3.507863000
1	4.061661000	-0.839392000	-5.195886000
6	5.654364000	0.018733000	-4.005588000
1	5.797464000	0.904189000	-4.641453000
1	5.798109000	0.356335000	-2.970678000
6	6.718861000	-1.021632000	-4.351952000
1	6.591813000	-1.905365000	-3.710832000
1	6.563633000	-1.370588000	-5.382571000
6	8.145351000	-0.489582000	-4.209305000
1	8.265186000	0.393851000	-4.850684000
1	8.302312000	-0.140956000	-3.179270000
6	9.207530000	-1.528216000	-4.564289000
1	9.132853000	-2.405576000	-3.912267000
1	9.088797000	-1.872533000	-5.598155000
1	10.217588000	-1.118262000	-4.461455000
6	4.483402000	4.972394000	-1.147218000
1	4.672984000	4.073946000	-1.747624000
1	4.197896000	5.768546000	-1.844429000
6	5.742050000	5.363064000	-0.370716000
1	5.609057000	6.362519000	0.066095000
1	5.875042000	4.678092000	0.477982000
6	7.012983000	5.336504000	-1.216391000
1	7.107281000	4.348559000	-1.688603000
1	6.930890000	6.064396000	-2.035716000
6	8.270712000	5.618359000	-0.395957000
1	8.179382000	6.600932000	0.086716000
1	8.332211000	4.883910000	0.418122000
6	9.552055000	5.570091000	-1.224960000
1	9.679320000	4.586655000	-1.692274000
1	9.531135000	6.317514000	-2.026376000
1	10.435001000	5.764891000	-0.607405000
6	4.269497000	2.983900000	3.302835000
1	4.498464000	2.483155000	2.354274000
1	3.855197000	3.967397000	3.045054000
6	5.551750000	3.151205000	4.113914000
1	5.347192000	3.733185000	5.023040000
1	5.899042000	2.164648000	4.451959000
6	6.667171000	3.824280000	3.313755000
1	6.875199000	3.224133000	2.415192000
1	6.321876000	4.802843000	2.950520000
6	7.958132000	4.005026000	4.111825000
1	7.754328000	4.634219000	4.988292000
1	8.279763000	3.030253000	4.502609000
6	9.087063000	4.621952000	3.288771000
1	9.351707000	3.978432000	2.441433000
1	8.792796000	5.596989000	2.883626000
1	9.989234000	4.766533000	3.891919000

6	4.490190000	-3.465179000	3.136248000
1	4.526534000	-2.424451000	3.479413000
1	4.428832000	-4.096567000	4.030819000
6	5.740196000	-3.802768000	2.327113000
1	5.669892000	-4.836761000	1.960086000
1	5.761320000	-3.166962000	1.431291000
6	7.050507000	-3.634713000	3.092772000
1	7.117604000	-2.613405000	3.494034000
1	7.054416000	-4.303972000	3.964246000
6	8.275051000	-3.917909000	2.222751000
1	8.204896000	-4.940606000	1.827928000
1	8.256127000	-3.251851000	1.349036000
6	9.597084000	-3.744902000	2.966475000
1	9.705574000	-2.720385000	3.340844000
1	9.655982000	-4.420965000	3.827080000
1	10.451327000	-3.956103000	2.314809000
6	4.018974000	-4.510316000	-1.492593000
1	3.988992000	-4.361583000	-0.406448000
1	3.873212000	-5.581539000	-1.677774000
6	5.365368000	-4.049361000	-2.050359000
1	5.377276000	-4.208320000	-3.138459000
1	5.457326000	-2.965539000	-1.899967000
6	6.573771000	-4.752073000	-1.434817000
1	6.604211000	-4.557018000	-0.353857000
1	6.460472000	-5.839862000	-1.545452000
6	7.894501000	-4.312539000	-2.067508000
1	7.852534000	-4.498567000	-3.149540000
1	8.004751000	-3.226824000	-1.948196000
6	9.113676000	-5.014823000	-1.474392000
1	9.205033000	-4.805162000	-0.402822000
1	9.040064000	-6.101758000	-1.595712000
1	10.038068000	-4.684956000	-1.960227000
6	-0.741456000	-0.616821000	-0.143889000
1	-0.579549000	-1.459625000	0.543136000
6	-2.059129000	0.014461000	0.187893000
1	-2.144907000	0.466091000	1.179037000
1	-0.779124000	-1.036092000	-1.155701000
6	-3.099966000	0.034484000	-0.645116000
1	-3.010543000	-0.377687000	-1.647122000
1	-4.061254000	0.465514000	-0.370188000
6	2.988175000	0.422381000	-0.047615000
1	2.981033000	1.363474000	-0.620223000
1	2.981244000	0.695409000	1.017312000
6	1.751461000	-0.395053000	-0.367818000
1	1.756182000	-0.661559000	-1.433226000
1	1.803274000	-1.337844000	0.195091000
6	0.451709000	0.334590000	-0.026357000
1	0.502208000	0.734924000	0.993828000
1	0.311550000	1.192110000	-0.696125000
8	4.158803000	-0.331490000	-0.374669000
14	5.647919000	0.097644000	0.245588000
6	5.656372000	-0.035559000	2.125407000
1	6.008106000	-1.023751000	2.435135000
1	6.321544000	0.710106000	2.576278000
1	4.655891000	0.108315000	2.548162000
6	6.064930000	1.856483000	-0.264228000
1	7.046943000	2.163323000	0.116126000
1	6.077052000	1.968718000	-1.354143000
1	5.325578000	2.559183000	0.131461000
6	6.922208000	-1.077578000	-0.459921000
1	6.731061000	-2.111332000	-0.156614000
1	6.943583000	-1.049396000	-1.553098000

1	7.921419000	-0.806041000	-0.097260000

D_{1b}'

E_1 = -5181.24828659 A.U.

At No.	X	Y	Z
6	-0.843404000	4.386489000	-0.797401000
6	-2.076563000	4.061228000	-1.367389000
6	0.318384000	3.967793000	-1.440647000
6	-2.119634000	3.357078000	-2.568290000
6	0.275041000	3.228593000	-2.621474000
6	-0.959081000	2.933886000	-3.209618000
1	1.259050000	4.216672000	-0.965008000
1	-3.063526000	3.090310000	-3.027726000
6	-0.746621000	5.100178000	0.533398000
1	0.161119000	5.708559000	0.554115000
1	-1.601154000	5.770465000	0.658211000
6	-0.723692000	4.103659000	1.673019000
6	-1.920649000	3.620929000	2.187418000
6	0.474713000	3.587093000	2.176638000
6	-1.949764000	2.650216000	3.185425000
6	0.450107000	2.660078000	3.215753000
6	-0.754439000	2.181174000	3.733306000
1	-2.835406000	4.007278000	1.755123000
1	1.368259000	2.246106000	3.615629000
6	-0.760693000	1.128081000	4.820425000
1	-1.658677000	1.241715000	5.433025000
1	0.106132000	1.277703000	5.469708000
6	-0.723501000	-0.272931000	4.249915000
6	-1.904323000	-0.960734000	3.983041000
6	0.495477000	-0.892526000	3.957661000
6	-1.884934000	-2.245121000	3.446400000
6	0.515286000	-2.176644000	3.416595000
6	-0.666626000	-2.862783000	3.147097000
1	-2.837755000	-0.455066000	4.201229000
1	1.451250000	-2.665595000	3.169474000
6	-0.636630000	-4.220170000	2.478349000
1	-1.456907000	-4.835955000	2.856943000
1	0.300881000	-4.725471000	2.723131000
6	-0.763556000	-4.088328000	0.974411000
6	0.369699000	-3.993815000	0.162451000
6	-2.019246000	-4.004861000	0.375238000
6	0.229755000	-3.862349000	-1.217136000
6	-2.158544000	-3.837042000	-0.999840000
6	-1.024753000	-3.788757000	-1.815261000
1	-2.885475000	-4.051989000	1.023479000
1	1.092677000	-3.778879000	-1.866492000
6	-1.140553000	-3.588041000	-3.310764000
1	-0.313725000	-4.100961000	-3.808741000
1	-2.072693000	-4.030739000	-3.670940000
6	-1.115052000	-2.117046000	-3.663660000
6	0.088567000	-1.454221000	-3.914020000
6	-2.296966000	-1.381728000	-3.675662000
6	0.083414000	-0.090673000	-4.197184000
6	-2.301525000	-0.013911000	-3.939062000
6	-1.099159000	0.643661000	-4.218203000
1	-3.211494000	-1.913598000	-3.443260000
1	1.006092000	0.446662000	-4.381217000
6	-1.055836000	2.131848000	-4.488978000
1	-0.195406000	2.353812000	-5.126393000
1	-1.956274000	2.433374000	-5.031578000
8	-3.013412000	-2.981570000	3.199941000
6	-4.261439000	-2.330270000	3.247367000

1	-4.284165000	-1.509068000	2.512876000
1	-4.432806000	-1.892369000	4.242076000
8	-3.127016000	2.119009000	3.660484000
6	-4.191686000	2.114393000	2.732770000
1	-3.837349000	1.693410000	1.779096000
1	-4.528852000	3.140385000	2.532083000
8	-3.200990000	4.469292000	-0.696515000
6	-4.435552000	3.939410000	-1.126346000
1	-4.658024000	4.266665000	-2.153479000
1	-4.386413000	2.839307000	-1.130913000
8	-3.444468000	0.745269000	-3.948717000
6	-4.619421000	0.143841000	-3.451066000
1	-4.880278000	-0.739331000	-4.053519000
1	-4.455001000	-0.198476000	-2.416834000
8	-3.370643000	-3.720345000	-1.629262000
6	-4.515118000	-3.503672000	-0.834508000
1	-4.700891000	-4.372889000	-0.185375000
1	-4.364373000	-2.628241000	-0.182524000
8	1.586159000	-4.039875000	0.794845000
6	2.735927000	-3.966570000	-0.017079000
1	2.727985000	-4.776663000	-0.761803000
1	2.746492000	-3.013066000	-0.565824000
8	1.230236000	-2.209883000	-3.868891000
6	2.460782000	-1.520502000	-3.869675000
1	2.597609000	-0.977871000	-4.817234000
1	2.466268000	-0.777021000	-3.058777000
8	1.396288000	2.761559000	-3.255443000
6	2.631041000	3.005780000	-2.619837000
1	2.836473000	4.087568000	-2.597947000
1	2.587329000	2.660690000	-1.576478000
8	1.623832000	4.041576000	1.588664000
6	2.849663000	3.422252000	1.922356000
1	3.077123000	3.571741000	2.988993000
1	2.785117000	2.338994000	1.740170000
8	1.615507000	-0.163185000	4.234428000
6	2.882607000	-0.747282000	4.034661000
1	3.014087000	-1.609213000	4.707011000
1	2.982619000	-1.104208000	3.001147000
6	-5.704669000	-3.298598000	-1.756800000
1	-5.485916000	-2.480102000	-2.453382000
1	-5.833173000	-4.203893000	-2.362635000
6	-6.988669000	-2.997755000	-0.982408000
1	-7.085486000	-3.704137000	-0.145235000
1	-6.919724000	-1.997148000	-0.531267000
6	-8.247117000	-3.085629000	-1.845855000
1	-8.165410000	-2.387109000	-2.689931000
1	-8.309104000	-4.090835000	-2.285990000
6	-9.530026000	-2.795731000	-1.069073000
1	-9.581754000	-3.467435000	-0.202234000
1	-9.485245000	-1.775756000	-0.666338000
6	-10.795431000	-2.957060000	-1.907700000
1	-10.785150000	-2.287727000	-2.775503000
1	-10.890346000	-3.982704000	-2.282316000
1	-11.691384000	-2.729542000	-1.320883000
6	-5.344245000	-3.353117000	2.955073000
1	-5.131000000	-3.845023000	1.998531000
1	-5.307622000	-4.131765000	3.726549000
6	-6.728981000	-2.711944000	2.912394000
1	-6.877280000	-2.100203000	3.814091000
1	-6.782219000	-2.019800000	2.060228000
6	-7.867075000	-3.724860000	2.809202000
1	-7.711831000	-4.364909000	1.928754000

1	-7.840022000	-4.394100000	3.680396000
6	-9.238991000	-3.060526000	2.714060000
1	-9.388087000	-2.411576000	3.588116000
1	-9.253096000	-2.401471000	1.837505000
6	-10.387776000	-4.060830000	2.615507000
1	-10.270435000	-4.709408000	1.739126000
1	-10.426652000	-4.706131000	3.500492000
1	-11.352943000	-3.551561000	2.526902000
6	-5.383940000	1.324950000	3.246239000
1	-5.051693000	0.386986000	3.703520000
1	-5.898946000	1.896325000	4.027677000
6	-6.321337000	1.046251000	2.065825000
1	-6.293496000	1.903730000	1.377483000
1	-5.926641000	0.196358000	1.489582000
6	-7.781608000	0.789011000	2.424588000
1	-7.860345000	-0.006225000	3.177551000
1	-8.204299000	1.694202000	2.883755000
6	-8.595587000	0.412677000	1.187943000
1	-8.370369000	1.132699000	0.389817000
1	-8.248185000	-0.561559000	0.817233000
6	-10.101704000	0.369313000	1.424105000
1	-10.356353000	-0.334838000	2.223894000
1	-10.480297000	1.355633000	1.714062000
1	-10.639894000	0.058943000	0.521206000
6	-5.531445000	4.413928000	-0.191568000
1	-5.309050000	4.070700000	0.825476000
1	-5.531373000	5.509948000	-0.162946000
6	-6.901558000	3.898406000	-0.630782000
1	-7.140126000	4.305312000	-1.623703000
1	-6.862325000	2.805219000	-0.751754000
6	-8.021889000	4.253631000	0.345069000
1	-7.836240000	3.755082000	1.307906000
1	-7.998017000	5.332941000	0.550621000
6	-9.410316000	3.868874000	-0.164185000
1	-9.599222000	4.381006000	-1.117538000
1	-9.432881000	2.794406000	-0.387374000
6	-10.520806000	4.205403000	0.827741000
1	-10.346655000	3.712825000	1.791762000
1	-10.569744000	5.284352000	1.013664000
1	-11.498967000	3.882002000	0.457231000
6	-5.767487000	1.135974000	-3.510376000
1	-5.495732000	2.052518000	-2.974172000
1	-5.939392000	1.417615000	-4.556298000
6	-7.036111000	0.525623000	-2.911732000
1	-7.187971000	-0.473883000	-3.344218000
1	-6.895515000	0.368724000	-1.831446000
6	-8.300472000	1.351122000	-3.144941000
1	-8.186242000	2.346003000	-2.694236000
1	-8.432004000	1.515007000	-4.223602000
6	-9.548413000	0.673574000	-2.580463000
1	-9.640370000	-0.326832000	-3.023727000
1	-9.414509000	0.514361000	-1.502927000
6	-10.833833000	1.459271000	-2.825325000
1	-10.780537000	2.454605000	-2.370172000
1	-11.014255000	1.593494000	-3.898013000
1	-11.700945000	0.942117000	-2.401115000
6	3.748548000	2.277302000	-3.342307000
1	3.586488000	1.194874000	-3.263220000
1	3.727195000	2.531381000	-4.408627000
6	5.097239000	2.654677000	-2.727990000
1	5.277601000	3.727712000	-2.885495000
1	5.042046000	2.518611000	-1.637632000

6	6.284929000	1.860575000	-3.266502000
1	6.137300000	0.792469000	-3.060878000
1	6.328126000	1.961214000	-4.360319000
6	7.613970000	2.308882000	-2.658527000
1	7.758391000	3.379161000	-2.861292000
1	7.567148000	2.199305000	-1.567343000
6	8.807020000	1.514521000	-3.179903000
1	8.705386000	0.459407000	-2.908913000
1	8.884840000	1.581150000	-4.271579000
1	9.747182000	1.882531000	-2.754184000
6	3.928083000	4.063505000	1.065757000
1	3.683614000	3.910531000	0.006930000
1	3.906572000	5.146445000	1.239149000
6	5.324925000	3.519490000	1.360288000
1	5.526164000	3.592228000	2.438846000
1	5.366924000	2.450117000	1.111366000
6	6.422308000	4.259379000	0.596740000
1	6.241734000	4.173685000	-0.483471000
1	6.362472000	5.331161000	0.832618000
6	7.829394000	3.756500000	0.914708000
1	7.997839000	3.824993000	1.998313000
1	7.905865000	2.690864000	0.655694000
6	8.912921000	4.540359000	0.177958000
1	8.804951000	4.429343000	-0.906922000
1	8.848729000	5.609254000	0.412126000
1	9.914601000	4.196143000	0.452376000
6	3.919363000	0.325271000	4.310203000
1	3.798045000	1.120617000	3.562932000
1	3.712060000	0.778166000	5.287168000
6	5.347757000	-0.212438000	4.275788000
1	5.510218000	-0.879208000	5.133658000
1	5.493945000	-0.828906000	3.377204000
6	6.389902000	0.903345000	4.283967000
1	6.269832000	1.507631000	3.373063000
1	6.197574000	1.583425000	5.125770000
6	7.823936000	0.385589000	4.363715000
1	7.975750000	-0.118164000	5.327161000
1	7.969863000	-0.381084000	3.592294000
6	8.859297000	1.492933000	4.186469000
1	8.758834000	1.960526000	3.199903000
1	8.729457000	2.279234000	4.939008000
1	9.882534000	1.112633000	4.275705000
6	3.971477000	-4.082157000	0.854126000
1	3.932812000	-3.320889000	1.644301000
1	3.970234000	-5.058102000	1.354514000
6	5.234132000	-3.917813000	0.009891000
1	5.181933000	-4.604828000	-0.847122000
1	5.259310000	-2.906136000	-0.418712000
6	6.533375000	-4.187151000	0.764673000
1	6.590075000	-3.545653000	1.656630000
1	6.521828000	-5.222119000	1.134339000
6	7.772678000	-3.970870000	-0.106811000
1	7.600922000	-4.434827000	-1.087320000
1	7.901971000	-2.896981000	-0.299801000
6	9.042930000	-4.560537000	0.502806000
1	9.212649000	-4.186729000	1.518150000
1	8.972349000	-5.652490000	0.563058000
1	9.927585000	-4.314069000	-0.094033000
6	3.588005000	-2.520674000	-3.690768000
1	3.432735000	-3.084005000	-2.761689000
1	3.554321000	-3.246792000	-4.512149000
6	4.944571000	-1.818769000	-3.655355000

1	5.038743000	-1.157245000	-4.528618000
1	4.984732000	-1.160829000	-2.777243000
6	6.134439000	-2.776086000	-3.631752000
1	6.031496000	-3.475292000	-2.790859000
1	6.115368000	-3.388928000	-4.544106000
6	7.478912000	-2.053508000	-3.533024000
1	7.507235000	-1.248155000	-4.279867000
1	7.564230000	-1.564852000	-2.552347000
6	8.671645000	-2.981449000	-3.750925000
1	8.709211000	-3.766430000	-2.986644000
1	8.611879000	-3.473274000	-4.728863000
1	9.616785000	-2.431395000	-3.709094000
6	3.324358000	-0.158705000	-0.229982000
1	3.434915000	-0.878773000	-1.054799000
6	2.025551000	-0.410766000	0.482688000
1	1.906913000	-1.396805000	0.935225000
1	3.310113000	0.838095000	-0.689797000
6	1.028262000	0.463947000	0.590369000
1	1.075407000	1.456103000	0.142941000
1	0.117210000	0.218595000	1.128218000
6	7.119802000	-0.238447000	0.722733000
1	7.188069000	0.590121000	1.444692000
1	7.088549000	-1.174630000	1.302724000
6	5.857731000	-0.109239000	-0.111213000
1	5.870129000	0.865637000	-0.611846000
1	5.915165000	-0.863002000	-0.905768000
6	4.559637000	-0.282292000	0.670181000
1	4.556299000	-1.265817000	1.158882000
1	4.495552000	0.459249000	1.476990000
8	8.225675000	-0.238238000	-0.152741000
14	9.840409000	-0.378006000	0.272077000
6	10.028029000	-1.355564000	1.871374000
1	9.189493000	-2.041988000	2.029256000
1	10.941301000	-1.960765000	1.842520000
1	10.091422000	-0.699038000	2.744518000
6	10.595554000	1.326420000	0.479850000
1	11.677507000	1.256090000	0.644490000
1	10.427849000	1.938635000	-0.412821000
1	10.163144000	1.856353000	1.335504000
6	10.653426000	-1.278922000	-1.152806000
1	10.185467000	-2.256193000	-1.311735000
1	10.562153000	-0.710026000	-2.083820000
1	11.720412000	-1.439954000	-0.959686000

D_{1c}'

E_1 = -5181.24083718 A.U.

At No.	X	Y	Z
6	0.854530000	-4.189130000	1.301935000
6	2.074881000	-4.186479000	0.620183000
6	-0.323141000	-4.097250000	0.562598000
6	2.092708000	-4.106612000	-0.769441000
6	-0.303298000	-3.993322000	-0.826948000
6	0.917962000	-4.010338000	-1.508344000
1	-1.256723000	-4.077884000	1.113267000
1	3.025639000	-4.087515000	-1.319623000
6	0.801046000	-4.207021000	2.814349000
1	-0.117604000	-4.701580000	3.139636000
1	1.645644000	-4.779648000	3.206978000
6	0.849943000	-2.800565000	3.373571000
6	2.074512000	-2.174179000	3.571424000
6	-0.316807000	-2.076816000	3.636259000
6	2.161026000	-0.859217000	4.023717000

6	-0.233333000	-0.781162000	4.142393000
6	0.997591000	-0.154977000	4.339921000
1	2.963846000	-2.742148000	3.328948000
1	-1.125833000	-0.207366000	4.361759000
6	1.057348000	1.272559000	4.841318000
1	1.959327000	1.409159000	5.443941000
1	0.196077000	1.457558000	5.489764000
6	1.060931000	2.284305000	3.714692000
6	2.264017000	2.764159000	3.200986000
6	-0.136078000	2.746263000	3.155846000
6	2.290257000	3.691564000	2.161842000
6	-0.107360000	3.678428000	2.120570000
6	1.095013000	4.159916000	1.612622000
1	3.181581000	2.379753000	3.631900000
1	-1.019322000	4.036065000	1.658271000
6	1.096646000	5.095704000	0.423684000
1	1.973601000	5.746521000	0.462957000
1	0.206464000	5.728775000	0.454999000
6	1.114874000	4.298758000	-0.863113000
6	-0.072483000	3.921418000	-1.495760000
6	2.324576000	3.850920000	-1.390525000
6	-0.025110000	3.149426000	-2.654437000
6	2.368870000	3.046593000	-2.525901000
6	1.182869000	2.702438000	-3.181088000
1	3.229641000	4.127400000	-0.863197000
1	-0.930684000	2.840361000	-3.161811000
6	1.189948000	1.814833000	-4.405674000
1	0.332373000	2.068616000	-5.035149000
1	2.095576000	2.001153000	-4.989316000
6	1.129913000	0.346219000	-4.044319000
6	-0.092306000	-0.310252000	-3.869574000
6	2.305361000	-0.373689000	-3.847516000
6	-0.110649000	-1.672709000	-3.583225000
6	2.286302000	-1.724196000	-3.504619000
6	1.065085000	-2.394936000	-3.399503000
1	3.239178000	0.165852000	-3.950409000
1	-1.043584000	-2.207623000	-3.456618000
6	0.990815000	-3.855654000	-3.011453000
1	0.108041000	-4.306515000	-3.472921000
1	1.870650000	-4.385218000	-3.386325000
8	3.442459000	4.207771000	1.629517000
6	4.656577000	3.541949000	1.887095000
1	4.581114000	2.488785000	1.572041000
1	4.885401000	3.552739000	2.963483000
8	3.364531000	-0.210924000	4.175600000
6	4.406254000	-0.690779000	3.352389000
1	4.044648000	-0.754413000	2.314401000
1	4.700212000	-1.704113000	3.658812000
8	3.214048000	-4.267688000	1.379434000
6	4.443794000	-4.051672000	0.722915000
1	4.608923000	-4.821941000	-0.045802000
1	4.429183000	-3.073875000	0.216365000
8	3.422971000	-2.456160000	-3.268033000
6	4.623467000	-1.736962000	-3.090313000
1	4.889788000	-1.203299000	-4.015295000
1	4.492628000	-0.981739000	-2.299047000
8	3.531756000	2.561107000	-3.065193000
6	4.717311000	2.689508000	-2.311988000
1	4.953938000	3.751632000	-2.147305000
1	4.591034000	2.220370000	-1.322889000
8	-1.243655000	4.340591000	-0.918817000
6	-2.443157000	3.848948000	-1.475191000

1	-2.567400000	4.223100000	-2.503036000
1	-2.405746000	2.751027000	-1.526869000
8	-1.227412000	0.444566000	-3.997973000
6	-2.461713000	-0.209799000	-3.798285000
1	-2.609167000	-0.973341000	-4.578037000
1	-2.465030000	-0.721429000	-2.824300000
8	-1.431433000	-3.864341000	-1.592771000
6	-2.682991000	-3.918157000	-0.945094000
1	-2.812419000	-4.895054000	-0.454286000
1	-2.743584000	-3.145460000	-0.164622000
8	-1.498582000	-2.711526000	3.364787000
6	-2.692313000	-1.983312000	3.547801000
1	-2.841713000	-1.758595000	4.615214000
1	-2.637339000	-1.028405000	3.007521000
8	-1.297879000	2.243898000	3.677389000
6	-2.505127000	2.685670000	3.095928000
1	-2.605740000	3.775155000	3.216629000
1	-2.503926000	2.467392000	2.018952000
6	5.850058000	2.036447000	-3.085154000
1	5.577266000	1.001527000	-3.323800000
1	5.965003000	2.562064000	-4.041007000
6	7.166937000	2.064461000	-2.307442000
1	7.316204000	3.064442000	-1.874813000
1	7.105586000	1.370088000	-1.456860000
6	8.381908000	1.716423000	-3.167435000
1	8.244572000	0.723124000	-3.616448000
1	8.440869000	2.424724000	-4.005797000
6	9.696628000	1.744842000	-2.390303000
1	9.805611000	2.725605000	-1.909399000
1	9.652682000	1.006692000	-1.579047000
6	10.920349000	1.472226000	-3.261426000
1	10.852066000	0.492086000	-3.747230000
1	11.016366000	2.226391000	-4.050922000
1	11.840538000	1.487782000	-2.668279000
6	5.758116000	4.261042000	1.129272000
1	5.499887000	4.298241000	0.063814000
1	5.802369000	5.299142000	1.480389000
6	7.112180000	3.581255000	1.313838000
1	7.297729000	3.421981000	2.385892000
1	7.084022000	2.582569000	0.855458000
6	8.276577000	4.373553000	0.723400000
1	8.091623000	4.560197000	-0.344313000
1	8.324237000	5.361987000	1.201284000
6	9.616831000	3.659739000	0.886302000
1	9.793020000	3.460719000	1.952458000
1	9.557820000	2.679595000	0.397746000
6	10.792421000	4.444606000	0.310111000
1	10.647509000	4.642508000	-0.758667000
1	10.905285000	5.412228000	0.812206000
1	11.732270000	3.894422000	0.423123000
6	5.638699000	0.195092000	3.426110000
1	5.349124000	1.250684000	3.407559000
1	6.164100000	0.024144000	4.373225000
6	6.541349000	-0.137179000	2.232381000
1	6.458684000	-1.211264000	2.010317000
1	6.154056000	0.375988000	1.339707000
6	8.022394000	0.184280000	2.406851000
1	8.158119000	1.229191000	2.714961000
1	8.432199000	-0.434278000	3.218221000
6	8.797497000	-0.080335000	1.117419000
1	8.517321000	-1.073101000	0.740615000
1	8.465838000	0.633763000	0.351056000

6	10.312690000	-0.004599000	1.273233000
1	10.622285000	0.973192000	1.658359000
1	10.672664000	-0.768230000	1.971447000
1	10.821438000	-0.163241000	0.315493000
6	5.565663000	-4.100138000	1.742156000
1	5.401633000	-3.320226000	2.494832000
1	5.532023000	-5.061007000	2.269115000
6	6.929676000	-3.910621000	1.079134000
1	7.105110000	-4.733281000	0.371380000
1	6.922271000	-2.988823000	0.477906000
6	8.083554000	-3.847970000	2.078271000
1	7.963405000	-2.960528000	2.717045000
1	8.031891000	-4.716543000	2.749593000
6	9.457893000	-3.808704000	1.411322000
1	9.579356000	-4.701828000	0.783251000
1	9.509130000	-2.949556000	0.730158000
6	10.603668000	-3.729344000	2.416953000
1	10.496103000	-2.850817000	3.064119000
1	10.622513000	-4.613979000	3.063613000
1	11.573168000	-3.658666000	1.913245000
6	5.744632000	-2.697403000	-2.734291000
1	5.464813000	-3.283442000	-1.850941000
1	5.878079000	-3.408142000	-3.558848000
6	7.046158000	-1.935851000	-2.477456000
1	7.210378000	-1.225379000	-3.300356000
1	6.943344000	-1.325001000	-1.567642000
6	8.280483000	-2.827964000	-2.354483000
1	8.155562000	-3.526182000	-1.515966000
1	8.371622000	-3.445802000	-3.258749000
6	9.563830000	-2.021130000	-2.162100000
1	9.664577000	-1.310499000	-2.993118000
1	9.472231000	-1.411530000	-1.254191000
6	10.820020000	-2.883635000	-2.074701000
1	10.757481000	-3.588237000	-1.237833000
1	10.960137000	-3.467927000	-2.991337000
1	11.713893000	-2.267922000	-1.929106000
6	-3.774853000	-3.686108000	-1.973964000
1	-3.710790000	-2.650743000	-2.333477000
1	-3.596185000	-4.335329000	-2.839145000
6	-5.162035000	-3.957142000	-1.391668000
1	-5.260928000	-5.030009000	-1.176171000
1	-5.262915000	-3.445233000	-0.423596000
6	-6.301978000	-3.510573000	-2.304299000
1	-6.256968000	-2.419661000	-2.424130000
1	-6.159209000	-3.933715000	-3.308623000
6	-7.681512000	-3.907501000	-1.778747000
1	-7.755901000	-5.003138000	-1.766746000
1	-7.781091000	-3.584206000	-0.733890000
6	-8.825815000	-3.314303000	-2.596902000
1	-8.848901000	-2.224096000	-2.497183000
1	-8.723961000	-3.567267000	-3.659131000
1	-9.793250000	-3.693856000	-2.251585000
6	-3.848760000	-2.804598000	3.010385000
1	-3.675360000	-3.007272000	1.944824000
1	-3.878824000	-3.775530000	3.519191000
6	-5.167527000	-2.059313000	3.193098000
1	-5.332540000	-1.868719000	4.263293000
1	-5.081831000	-1.072116000	2.718280000
6	-6.383308000	-2.783690000	2.620116000
1	-6.258442000	-2.906141000	1.535026000
1	-6.442529000	-3.798809000	3.036291000
6	-7.685091000	-2.036244000	2.906392000

1	-7.834845000	-1.987647000	3.993505000
1	-7.573125000	-0.996772000	2.571710000
6	-8.913047000	-2.655375000	2.242885000
1	-8.851102000	-2.596898000	1.150813000
1	-9.022248000	-3.709481000	2.522907000
1	-9.828593000	-2.134218000	2.544605000
6	-3.671830000	1.978632000	3.755298000
1	-3.559766000	0.896400000	3.618331000
1	-3.655557000	2.174475000	4.834338000
6	-4.991806000	2.445955000	3.145114000
1	-5.067006000	3.539976000	3.226261000
1	-4.985232000	2.220364000	2.069720000
6	-6.225398000	1.815147000	3.785913000
1	-6.157132000	0.720042000	3.723040000
1	-6.243373000	2.057378000	4.857680000
6	-7.528125000	2.281015000	3.137751000
1	-7.572714000	3.378290000	3.164109000
1	-7.515191000	2.004619000	2.074491000
6	-8.772338000	1.702387000	3.806403000
1	-8.783304000	0.607958000	3.742849000
1	-8.805664000	1.971914000	4.868233000
1	-9.689195000	2.073035000	3.337907000
6	-3.616895000	4.292377000	-0.619998000
1	-3.524286000	3.854809000	0.383456000
1	-3.584892000	5.381179000	-0.495170000
6	-4.937400000	3.868454000	-1.263599000
1	-5.038961000	4.363231000	-2.239644000
1	-4.904392000	2.790937000	-1.480428000
6	-6.175586000	4.163850000	-0.418900000
1	-6.109115000	3.619472000	0.534064000
1	-6.201768000	5.231267000	-0.159389000
6	-7.469031000	3.777610000	-1.137251000
1	-7.553583000	4.356363000	-2.066577000
1	-7.403824000	2.726757000	-1.448323000
6	-8.722614000	3.982155000	-0.290202000
1	-8.675877000	3.394169000	0.633959000
1	-8.840297000	5.033931000	-0.005806000
1	-9.623625000	3.679749000	-0.835615000
6	-3.587153000	0.809167000	-3.841666000
1	-3.607570000	1.365971000	-2.896060000
1	-3.388175000	1.536761000	-4.636981000
6	-4.931020000	0.119650000	-4.084782000
1	-4.981047000	-0.205720000	-5.132768000
1	-4.988676000	-0.802320000	-3.487245000
6	-6.142700000	0.987489000	-3.757425000
1	-6.158165000	1.185722000	-2.676144000
1	-6.043471000	1.969420000	-4.241348000
6	-7.459888000	0.338617000	-4.181952000
1	-7.471843000	0.246090000	-5.275941000
1	-7.503682000	-0.687369000	-3.791183000
6	-8.691715000	1.107731000	-3.713950000
1	-8.777024000	1.082652000	-2.622682000
1	-8.647839000	2.157803000	-4.026635000
1	-9.609371000	0.672296000	-4.121405000
6	-4.553307000	-0.371362000	-0.250757000
1	-4.563664000	-0.461779000	-1.347044000
6	-3.352797000	0.435731000	0.158327000
1	-3.465031000	1.516730000	0.059098000
1	-4.468571000	-1.393609000	0.140127000
6	-2.187852000	-0.048592000	0.585910000
1	-2.008970000	-1.116962000	0.697530000
1	-1.354221000	0.603709000	0.832140000

6	-8.420117000	0.127916000	-0.000783000
1	-8.523086000	0.170535000	1.093166000
1	-8.461055000	1.165536000	-0.366036000
6	-7.089578000	-0.497171000	-0.380217000
1	-7.082448000	-1.537594000	-0.035695000
1	-7.029955000	-0.532721000	-1.474483000
6	-5.885358000	0.247540000	0.184401000
1	-5.917443000	1.296817000	-0.141066000
1	-5.941811000	0.268674000	1.280121000
8	-9.460414000	-0.641721000	-0.559940000
14	-11.085831000	-0.301674000	-0.339539000
6	-11.704012000	0.816652000	-1.715732000
1	-11.619447000	0.327342000	-2.691615000
1	-12.756829000	1.081063000	-1.560590000
1	-11.129529000	1.749036000	-1.761088000
6	-11.329220000	0.570279000	1.307748000
1	-12.395729000	0.724802000	1.507887000
1	-10.910637000	-0.001062000	2.143402000
1	-10.848486000	1.555667000	1.303749000
6	-11.956344000	-1.957451000	-0.386055000
1	-11.774541000	-2.462605000	-1.340772000
1	-11.589362000	-2.610602000	0.413015000
1	-13.039862000	-1.845335000	-0.265565000

D_{1d}’

E_1 = -5181.23051708 A.U.

At No.	X	Y	Z
6	1.207396000	4.483425000	-0.491634000
6	2.436755000	4.311612000	0.149675000
6	0.040116000	4.270116000	0.237402000
6	2.472748000	3.961459000	1.497355000
6	0.076196000	3.897893000	1.579452000
6	1.306830000	3.751870000	2.227579000
1	-0.899375000	4.373823000	-0.292572000
1	3.413601000	3.813321000	2.013354000
6	1.121481000	4.807904000	-1.967786000
1	0.231687000	5.413893000	-2.155483000
1	1.993851000	5.393389000	-2.269757000
6	1.062067000	3.537575000	-2.788848000
6	2.239288000	2.874507000	-3.115344000
6	-0.152978000	2.956014000	-3.159458000
6	2.233701000	1.657305000	-3.792796000
6	-0.160081000	1.764309000	-3.881638000
6	1.021355000	1.096930000	-4.201323000
1	3.165250000	3.328919000	-2.785874000
1	-1.090530000	1.297015000	-4.180890000
6	0.970699000	-0.228869000	-4.929940000
1	1.841536000	-0.318944000	-5.585162000
1	0.078739000	-0.250969000	-5.563156000
6	0.942440000	-1.414536000	-3.987838000
6	2.127951000	-2.056056000	-3.632671000
6	-0.260507000	-1.887239000	-3.449183000
6	2.130901000	-3.152926000	-2.774435000
6	-0.255577000	-2.997646000	-2.607788000
6	0.928335000	-3.639278000	-2.259526000
1	3.052521000	-1.662714000	-4.040134000
1	-1.170372000	-3.375873000	-2.168981000
6	0.912168000	-4.776882000	-1.262568000
1	1.749910000	-5.451581000	-1.454153000
1	-0.012529000	-5.348312000	-1.374512000
6	1.012132000	-4.236569000	0.147792000
6	-0.135267000	-3.915577000	0.879319000

6	2.258113000	-3.976345000	0.711390000
6	-0.018606000	-3.395184000	2.165806000
6	2.376547000	-3.417475000	1.980970000
6	1.229625000	-3.139179000	2.731013000
1	3.132706000	-4.201448000	0.112696000
1	-0.895794000	-3.142461000	2.750934000
6	1.332386000	-2.508741000	4.103132000
1	0.485450000	-2.833591000	4.713088000
1	2.247083000	-2.848999000	4.595673000
6	1.344951000	-0.997219000	4.018664000
6	0.155693000	-0.264609000	4.048125000
6	2.545453000	-0.310951000	3.856800000
6	0.192062000	1.124823000	3.959692000
6	2.581028000	1.076990000	3.738387000
6	1.393786000	1.812264000	3.809977000
1	3.450660000	-0.903243000	3.802731000
1	-0.718421000	1.711866000	3.974292000
6	1.389385000	3.320092000	3.675509000
1	0.537731000	3.725565000	4.228677000
1	2.299773000	3.731153000	4.120461000
8	3.264002000	-3.825891000	-2.398493000
6	4.508431000	-3.187601000	-2.563636000
1	4.498031000	-2.208986000	-2.057531000
1	4.716990000	-3.009966000	-3.629524000
8	3.388496000	0.968319000	-4.079885000
6	4.470404000	1.231483000	-3.211422000
1	4.132212000	1.116053000	-2.170023000
1	4.813606000	2.268186000	-3.330755000
8	3.566384000	4.502251000	-0.605266000
6	4.791916000	4.082626000	-0.047192000
1	5.028929000	4.675060000	0.849790000
1	4.718715000	3.027815000	0.260614000
8	3.741349000	1.787014000	3.560640000
6	4.904969000	1.053790000	3.247959000
1	5.155372000	0.366242000	4.070012000
1	4.732042000	0.443490000	2.347179000
8	3.581023000	-3.121606000	2.565090000
6	4.736029000	-3.149757000	1.755317000
1	4.911988000	-4.166595000	1.372900000
1	4.605337000	-2.485818000	0.885691000
8	-1.331122000	-4.138066000	0.251022000
6	-2.529159000	-3.849851000	0.936178000
1	-2.623636000	-4.494734000	1.823651000
1	-2.529941000	-2.805217000	1.283376000
8	-1.003363000	-0.982690000	4.173110000
6	-2.220435000	-0.281769000	4.050208000
1	-2.312442000	0.468982000	4.850063000
1	-2.254970000	0.249043000	3.086294000
8	-1.047636000	3.663070000	2.326199000
6	-2.287169000	3.700100000	1.654107000
1	-2.445666000	4.693508000	1.207257000
1	-2.292968000	2.961780000	0.837347000
8	-1.293368000	3.609050000	-2.770047000
6	-2.513792000	2.924407000	-2.939262000
1	-2.742992000	2.809250000	-4.009847000
1	-2.432520000	1.913836000	-2.512730000
8	-1.405726000	-1.217428000	-3.791004000
6	-2.615226000	-1.700327000	-3.243722000
1	-2.792866000	-2.732653000	-3.582782000
1	-2.554834000	-1.716059000	-2.146065000
6	5.923908000	-2.717684000	2.597749000
1	5.714844000	-1.738821000	3.045764000

1	6.037716000	-3.427291000	3.426354000
6	7.214411000	-2.653113000	1.779136000
1	7.301914000	-3.558162000	1.160586000
1	7.162060000	-1.807738000	1.077652000
6	8.469720000	-2.526302000	2.642227000
1	8.396816000	-1.628181000	3.270854000
1	8.516132000	-3.379710000	3.333289000
6	9.758324000	-2.470071000	1.823962000
1	9.803614000	-3.349875000	1.169069000
1	9.728333000	-1.594608000	1.162508000
6	11.018897000	-2.416742000	2.683454000
1	11.014373000	-1.540603000	3.341947000
1	11.099345000	-3.306727000	3.318079000
1	11.919615000	-2.363936000	2.063178000
6	5.580358000	-4.092195000	-1.982591000
1	5.338697000	-4.312269000	-0.935403000
1	5.558189000	-5.048194000	-2.519717000
6	6.968325000	-3.464505000	-2.076331000
1	7.144415000	-3.119829000	-3.105527000
1	7.006676000	-2.567958000	-1.441504000
6	8.094034000	-4.414876000	-1.674248000
1	7.916131000	-4.786481000	-0.654760000
1	8.075762000	-5.298340000	-2.327562000
6	9.470790000	-3.757241000	-1.738916000
1	9.640389000	-3.373244000	-2.754377000
1	9.477403000	-2.883287000	-1.076347000
6	10.607969000	-4.698253000	-1.349978000
1	10.469449000	-5.083680000	-0.332751000
1	10.655848000	-5.560071000	-2.025328000
1	11.576296000	-4.188404000	-1.384652000
6	5.651752000	0.316346000	-3.486211000
1	5.307129000	-0.708749000	-3.655216000
1	6.164976000	0.636186000	-4.400901000
6	6.596533000	0.376133000	-2.280329000
1	6.579244000	1.393890000	-1.863369000
1	6.199854000	-0.272098000	-1.484914000
6	8.052892000	0.015832000	-2.557042000
1	8.122191000	-0.960071000	-3.055085000
1	8.479777000	0.751008000	-3.254280000
6	8.870305000	-0.003274000	-1.266684000
1	8.658617000	0.917150000	-0.706181000
1	8.513705000	-0.826920000	-0.632819000
6	10.374332000	-0.132207000	-1.484130000
1	10.615372000	-1.037360000	-2.052296000
1	10.763765000	0.726365000	-2.042138000
1	10.913601000	-0.181946000	-0.531246000
6	5.891705000	4.255228000	-1.077139000
1	5.660328000	3.642659000	-1.956557000
1	5.909601000	5.298511000	-1.413737000
6	7.254396000	3.862462000	-0.507194000
1	7.501231000	4.529570000	0.330933000
1	7.197731000	2.848843000	-0.082219000
6	8.378674000	3.910131000	-1.540239000
1	8.186944000	3.159008000	-2.320641000
1	8.367778000	4.885729000	-2.046080000
6	9.762516000	3.671658000	-0.937424000
1	9.955233000	4.430360000	-0.166654000
1	9.774104000	2.703723000	-0.419893000
6	10.877703000	3.704861000	-1.979382000
1	10.699622000	2.963237000	-2.767111000
1	10.938349000	4.687660000	-2.460565000
1	11.851918000	3.488926000	-1.528898000

6	6.065373000	2.009477000	3.033102000
1	5.802494000	2.745449000	2.264451000
1	6.241516000	2.566996000	3.961003000
6	7.327117000	1.245471000	2.628144000
1	7.468994000	0.401329000	3.318285000
1	7.185197000	0.800175000	1.631688000
6	8.599092000	2.092052000	2.630425000
1	8.494363000	2.926466000	1.924153000
1	8.731029000	2.543959000	3.623439000
6	9.841480000	1.275627000	2.277315000
1	9.922455000	0.432174000	2.975731000
1	9.708622000	0.831530000	1.282621000
6	11.133808000	2.087151000	2.306407000
1	11.090649000	2.922917000	1.599047000
1	11.313432000	2.504702000	3.303712000
1	11.996843000	1.467299000	2.041445000
6	-3.405827000	3.399948000	2.634312000
1	-3.282267000	2.384445000	3.031318000
1	-3.337289000	4.091061000	3.482961000
6	-4.762102000	3.532642000	1.943248000
1	-4.866063000	4.549972000	1.539039000
1	-4.783180000	2.858369000	1.076095000
6	-5.960655000	3.232218000	2.841339000
1	-5.861254000	2.222910000	3.266272000
1	-5.961657000	3.920804000	3.697609000
6	-7.289528000	3.342352000	2.093173000
1	-7.392319000	4.360757000	1.693911000
1	-7.264643000	2.677440000	1.219678000
6	-8.504925000	3.002476000	2.951802000
1	-8.424688000	1.991004000	3.366427000
1	-8.600911000	3.697657000	3.793587000
1	-9.429479000	3.047465000	2.366829000
6	-3.624359000	3.699141000	-2.253071000
1	-3.406601000	3.774349000	-1.179343000
1	-3.647360000	4.722201000	-2.647087000
6	-4.972280000	3.013663000	-2.471453000
1	-5.155687000	2.907276000	-3.550137000
1	-4.921702000	1.988469000	-2.077770000
6	-6.158057000	3.735681000	-1.834410000
1	-5.999030000	3.826888000	-0.750873000
1	-6.215885000	4.761178000	-2.224850000
6	-7.478221000	3.011723000	-2.096220000
1	-7.615584000	2.889086000	-3.179100000
1	-7.406654000	1.995172000	-1.688207000
6	-8.696377000	3.711894000	-1.500660000
1	-8.609365000	3.800009000	-0.412229000
1	-8.807185000	4.722762000	-1.909422000
1	-9.615563000	3.158613000	-1.719420000
6	-3.765449000	-0.809121000	-3.671906000
1	-3.659992000	0.174712000	-3.198920000
1	-3.723029000	-0.653292000	-4.756645000
6	-5.102576000	-1.439460000	-3.281218000
1	-5.237300000	-2.372962000	-3.845494000
1	-5.069982000	-1.722976000	-2.219281000
6	-6.304864000	-0.525434000	-3.511711000
1	-6.288235000	0.284063000	-2.769863000
1	-6.221841000	-0.038403000	-4.493511000
6	-7.641315000	-1.263979000	-3.430591000
1	-7.706417000	-1.984096000	-4.256458000
1	-7.672638000	-1.861973000	-2.509320000
6	-8.841458000	-0.322184000	-3.466268000
1	-8.819800000	0.365522000	-2.612685000

1	-8.840694000	0.286215000	-4.378007000
1	-9.788148000	-0.872768000	-3.431677000
6	-3.676666000	-4.088766000	-0.032582000
1	-3.674853000	-3.294289000	-0.790197000
1	-3.488982000	-5.032001000	-0.559289000
6	-5.038301000	-4.149787000	0.656870000
1	-5.059821000	-5.005419000	1.345835000
1	-5.184792000	-3.256278000	1.279831000
6	-6.196352000	-4.260981000	-0.333730000
1	-6.207314000	-3.369732000	-0.976702000
1	-6.023780000	-5.115924000	-1.002485000
6	-7.557811000	-4.413529000	0.342302000
1	-7.563662000	-5.330718000	0.945712000
1	-7.701770000	-3.586187000	1.050053000
6	-8.716399000	-4.444334000	-0.651822000
1	-8.734260000	-3.534438000	-1.263045000
1	-8.626834000	-5.297844000	-1.333277000
1	-9.682203000	-4.519484000	-0.141306000
6	-3.365883000	-1.271732000	4.139875000
1	-3.307093000	-1.968187000	3.292836000
1	-3.257672000	-1.870696000	5.051754000
6	-4.708822000	-0.545376000	4.131318000
1	-4.801294000	0.064836000	5.040507000
1	-4.723936000	0.160752000	3.289628000
6	-5.912971000	-1.476049000	4.018070000
1	-5.819485000	-2.077243000	3.101581000
1	-5.912005000	-2.192062000	4.851422000
6	-7.238380000	-0.717868000	3.983779000
1	-7.366832000	-0.160788000	4.921366000
1	-7.184027000	0.037802000	3.190469000
6	-8.446872000	-1.618118000	3.743738000
1	-8.303585000	-2.225221000	2.842064000
1	-8.601010000	-2.307423000	4.581392000
1	-9.363752000	-1.035979000	3.605143000
6	-5.744719000	0.331607000	0.534544000
1	-5.911796000	0.808477000	1.509389000
6	-4.391888000	-0.319957000	0.555101000
1	-4.317792000	-1.229013000	1.153999000
1	-5.762476000	1.140043000	-0.207106000
6	-3.297536000	0.139907000	-0.048999000
1	-3.320556000	1.049389000	-0.646204000
1	-2.337110000	-0.361211000	0.037148000
6	-9.413503000	-0.966848000	0.204318000
1	-9.438443000	-1.231629000	-0.863503000
1	-9.241182000	-1.899486000	0.757154000
6	-8.272623000	0.005195000	0.461672000
1	-8.385173000	0.872385000	-0.201646000
1	-8.380461000	0.385215000	1.483801000
6	-6.899015000	-0.640307000	0.285467000
1	-6.809611000	-1.481900000	0.985855000
1	-6.805683000	-1.067150000	-0.720098000
8	-10.636959000	-0.413530000	0.637162000
14	-12.008802000	-0.305525000	-0.310388000
6	-12.259157000	-1.909012000	-1.262520000
1	-12.341163000	-2.761848000	-0.579359000
1	-13.179337000	-1.866332000	-1.856892000
1	-11.433018000	-2.115268000	-1.952758000
6	-11.863000000	1.135614000	-1.507885000
1	-12.769955000	1.236871000	-2.115829000
1	-11.718801000	2.074427000	-0.961371000
1	-11.017735000	1.012432000	-2.193479000
6	-13.418488000	-0.009353000	0.884499000

1	-13.525783000	-0.847089000	1.581478000
1	-13.244945000	0.896988000	1.474479000
1	-14.369003000	0.112954000	0.352604000

D_{1e}'

E_1 = -5181.21682755 A.U.

At No.	X	Y	Z
6	-0.603018000	-4.041581000	-1.076589000
6	-1.800976000	-4.159168000	-0.366823000
6	0.550373000	-3.678186000	-0.382636000
6	-1.818510000	-3.939533000	1.008095000
6	0.526342000	-3.432067000	0.987816000
6	-0.668578000	-3.577230000	1.701331000
1	1.463430000	-3.564080000	-0.955972000
1	-2.737210000	-4.016830000	1.576925000
6	-0.564929000	-4.201954000	-2.581754000
1	0.402359000	-4.609975000	-2.882508000
1	-1.339427000	-4.904949000	-2.898933000
6	-0.792027000	-2.863081000	-3.251886000
6	-2.086562000	-2.383197000	-3.416789000
6	0.273534000	-2.044128000	-3.627467000
6	-2.334144000	-1.116793000	-3.938564000
6	0.030173000	-0.786433000	-4.175793000
6	-1.268002000	-0.304311000	-4.339445000
1	-2.894863000	-3.023610000	-3.087609000
1	0.853432000	-0.142483000	-4.466553000
6	-1.519123000	1.068500000	-4.927441000
1	-2.419134000	1.034189000	-5.548035000
1	-0.681305000	1.329407000	-5.580861000
6	-1.689157000	2.154003000	-3.884901000
6	-2.957561000	2.542340000	-3.458545000
6	-0.575244000	2.795757000	-3.331695000
6	-3.126649000	3.551164000	-2.511899000
6	-0.747446000	3.824129000	-2.409464000
6	-2.015299000	4.210989000	-1.986054000
1	-3.811402000	2.023103000	-3.879234000
1	0.099968000	4.331160000	-1.964782000
6	-2.167263000	5.244449000	-0.890999000
1	-3.132689000	5.747151000	-0.982071000
1	-1.383515000	5.999737000	-0.986404000
6	-2.069761000	4.567782000	0.459718000
6	-0.837267000	4.417748000	1.100138000
6	-3.197796000	3.989709000	1.038054000
6	-0.760106000	3.729557000	2.308236000
6	-3.116449000	3.278300000	2.232306000
6	-1.887664000	3.156193000	2.889120000
1	-4.136831000	4.086768000	0.505683000
1	0.187126000	3.591270000	2.816355000
6	-1.759724000	2.373769000	4.177829000
1	-0.948091000	2.798775000	4.774578000
1	-2.681645000	2.469428000	4.757741000
6	-1.485117000	0.906572000	3.926160000
6	-0.179933000	0.421463000	3.801567000
6	-2.543616000	0.014210000	3.776413000
6	0.036230000	-0.939225000	3.596952000
6	-2.328043000	-1.339273000	3.525989000
6	-1.022161000	-1.833375000	3.460558000
1	-3.545686000	0.420830000	3.840271000
1	1.038044000	-1.340827000	3.506706000
6	-0.742680000	-3.295288000	3.186122000
1	0.201596000	-3.574716000	3.661479000
1	-1.531586000	-3.909928000	3.628024000

8	-4.349457000	3.969712000	-2.054765000
6	-5.445527000	3.094825000	-2.184911000
1	-5.194399000	2.113113000	-1.752473000
1	-5.696507000	2.938598000	-3.245062000
8	-3.605441000	-0.615604000	-4.081993000
6	-4.574423000	-1.186214000	-3.228382000
1	-4.210688000	-1.140115000	-2.190180000
1	-4.725940000	-2.245852000	-3.475357000
8	-2.921384000	-4.491702000	-1.084792000
6	-4.166674000	-4.361281000	-0.434666000
1	-4.236060000	-5.066423000	0.407743000
1	-4.268620000	-3.343699000	-0.026266000
8	-3.344242000	-2.243128000	3.347164000
6	-4.643223000	-1.725234000	3.161126000
1	-4.967221000	-1.175149000	4.057598000
1	-4.645841000	-1.015620000	2.318478000
8	-4.192833000	2.675384000	2.829069000
6	-5.391070000	2.566262000	2.092367000
1	-5.782394000	3.564615000	1.844797000
1	-5.206406000	2.037129000	1.143533000
8	0.250852000	4.972185000	0.477706000
6	1.528876000	4.599891000	0.943164000
1	1.676411000	4.942093000	1.978672000
1	1.621920000	3.502483000	0.940515000
8	0.834937000	1.337224000	3.896534000
6	2.143543000	0.880055000	3.631826000
1	2.446873000	0.140452000	4.389020000
1	2.174777000	0.381204000	2.651305000
8	1.620945000	-3.043707000	1.711861000
6	2.862880000	-2.905557000	1.056548000
1	3.160801000	-3.863249000	0.601519000
1	2.792853000	-2.160724000	0.248548000
8	1.530192000	-2.560383000	-3.446557000
6	2.597297000	-1.672139000	-3.190772000
1	2.851993000	-1.090791000	-4.089572000
1	2.310713000	-0.960808000	-2.400557000
8	0.654784000	2.362409000	-3.747323000
6	1.783016000	2.928528000	-3.119193000
1	1.843816000	4.004065000	-3.348794000
1	1.697996000	2.827676000	-2.026520000
6	-6.406003000	1.823468000	2.944477000
1	-5.980083000	0.863336000	3.259859000
1	-6.582937000	2.405770000	3.857024000
6	-7.724638000	1.591204000	2.205307000
1	-8.035660000	2.520473000	1.706251000
1	-7.571637000	0.851577000	1.406064000
6	-8.853298000	1.126075000	3.125690000
1	-8.552207000	0.203922000	3.641572000
1	-9.006254000	1.879004000	3.911640000
6	-10.171849000	0.888954000	2.391952000
1	-10.446449000	1.801115000	1.846684000
1	-10.028415000	0.107910000	1.633999000
6	-11.317117000	0.494390000	3.321201000
1	-11.082133000	-0.421675000	3.875162000
1	-11.515726000	1.282961000	4.056040000
1	-12.240495000	0.317911000	2.759961000
6	-6.631529000	3.718217000	-1.471278000
1	-6.362454000	3.913527000	-0.425767000
1	-6.842390000	4.691701000	-1.930376000
6	-7.867468000	2.825300000	-1.533494000
1	-8.053221000	2.532002000	-2.576755000
1	-7.673582000	1.893855000	-0.982430000

6	-9.124650000	3.488461000	-0.975331000
1	-8.943096000	3.808748000	0.060778000
1	-9.336535000	4.404519000	-1.544131000
6	-10.341973000	2.567355000	-1.013714000
1	-10.513413000	2.238633000	-2.048074000
1	-10.118824000	1.660920000	-0.437815000
6	-11.609239000	3.218613000	-0.466229000
1	-11.466917000	3.546415000	0.570477000
1	-11.886303000	4.100215000	-1.055394000
1	-12.454129000	2.522407000	-0.483222000
6	-5.916353000	-0.483660000	-3.348491000
1	-5.773375000	0.599855000	-3.402138000
1	-6.413214000	-0.787457000	-4.277551000
6	-6.765879000	-0.855402000	-2.127667000
1	-6.535980000	-1.890383000	-1.834340000
1	-6.454213000	-0.233882000	-1.275047000
6	-8.276958000	-0.753214000	-2.311645000
1	-8.554084000	0.237836000	-2.693723000
1	-8.597460000	-1.479691000	-3.072146000
6	-9.010433000	-1.025050000	-0.999588000
1	-8.599592000	-1.941008000	-0.554170000
1	-8.778772000	-0.218708000	-0.290108000
6	-10.521952000	-1.164287000	-1.146910000
1	-10.959571000	-0.265966000	-1.595914000
1	-10.775990000	-2.016132000	-1.787230000
1	-11.005323000	-1.322149000	-0.175979000
6	-5.276467000	-4.642435000	-1.429406000
1	-5.217690000	-3.916028000	-2.248689000
1	-5.120155000	-5.632807000	-1.872928000
6	-6.651390000	-4.574488000	-0.765280000
1	-6.721213000	-5.358508000	0.002052000
1	-6.757087000	-3.617350000	-0.232431000
6	-7.806748000	-4.727398000	-1.752873000
1	-7.802663000	-3.876886000	-2.450451000
1	-7.646157000	-5.625557000	-2.365509000
6	-9.172953000	-4.819216000	-1.074438000
1	-9.177947000	-5.678184000	-0.389591000
1	-9.331180000	-3.931313000	-0.448763000
6	-10.322299000	-4.951074000	-2.070472000
1	-10.329789000	-4.109274000	-2.772999000
1	-10.229652000	-5.870491000	-2.659702000
1	-11.291446000	-4.972477000	-1.561634000
6	-5.611188000	-2.866737000	2.904223000
1	-5.263624000	-3.462407000	2.052049000
1	-5.614714000	-3.531052000	3.776892000
6	-7.020472000	-2.333915000	2.640706000
1	-7.278568000	-1.607432000	3.424579000
1	-7.032394000	-1.773482000	1.693573000
6	-8.100452000	-3.414274000	2.606313000
1	-7.881821000	-4.136139000	1.808072000
1	-8.076013000	-3.980183000	3.548057000
6	-9.498937000	-2.833552000	2.402000000
1	-9.698013000	-2.099558000	3.194004000
1	-9.521115000	-2.272724000	1.459045000
6	-10.602111000	-3.888332000	2.395427000
1	-10.442414000	-4.620821000	1.596245000
1	-10.630144000	-4.433851000	3.345624000
1	-11.585586000	-3.432099000	2.241080000
6	3.880903000	-2.474563000	2.100306000
1	3.641514000	-1.458886000	2.440831000
1	3.771089000	-3.131812000	2.971289000
6	5.323133000	-2.535455000	1.598189000

1	5.561359000	-3.570953000	1.315881000
1	5.432040000	-1.937538000	0.681681000
6	6.328204000	-2.052702000	2.646388000
1	6.239149000	-0.962764000	2.764545000
1	6.066576000	-2.482663000	3.623438000
6	7.777931000	-2.414195000	2.323355000
1	7.877259000	-3.507089000	2.278176000
1	8.032204000	-2.049768000	1.318986000
6	8.763315000	-1.851728000	3.345335000
1	8.698626000	-0.758335000	3.396758000
1	8.550772000	-2.242479000	4.346886000
1	9.795265000	-2.114225000	3.093209000
6	3.781882000	-2.523068000	-2.768673000
1	3.488366000	-3.128999000	-1.902065000
1	4.001173000	-3.229105000	-3.578998000
6	5.035370000	-1.721463000	-2.427669000
1	5.313756000	-1.080568000	-3.275781000
1	4.831226000	-1.044386000	-1.584321000
6	6.204887000	-2.640934000	-2.078931000
1	5.905993000	-3.307242000	-1.257239000
1	6.413068000	-3.294234000	-2.937844000
6	7.484552000	-1.908811000	-1.684389000
1	7.784913000	-1.231964000	-2.496052000
1	7.286897000	-1.266806000	-0.815417000
6	8.622067000	-2.877107000	-1.369166000
1	8.344688000	-3.555975000	-0.554119000
1	8.868461000	-3.492196000	-2.242466000
1	9.530977000	-2.348453000	-1.065510000
6	3.032838000	2.220980000	-3.606571000
1	3.014095000	1.179144000	-3.264849000
1	3.030552000	2.200621000	-4.703061000
6	4.291075000	2.922578000	-3.098252000
1	4.333009000	3.936408000	-3.521416000
1	4.222713000	3.053976000	-2.007346000
6	5.583985000	2.181019000	-3.429824000
1	5.614015000	1.235487000	-2.870975000
1	5.593538000	1.912964000	-4.495794000
6	6.831531000	2.997599000	-3.099533000
1	6.842803000	3.908649000	-3.713007000
1	6.766184000	3.336284000	-2.057251000
6	8.125474000	2.215589000	-3.305946000
1	8.130520000	1.312063000	-2.685982000
1	8.236246000	1.907153000	-4.351876000
1	9.003629000	2.812631000	-3.036783000
6	2.578996000	5.230732000	0.046804000
1	2.438070000	4.884095000	-0.984980000
1	2.430742000	6.317257000	0.040177000
6	3.988565000	4.894646000	0.529647000
1	4.088571000	5.185554000	1.585333000
1	4.134211000	3.804056000	0.504298000
6	5.097742000	5.567852000	-0.276973000
1	5.059450000	5.219690000	-1.318807000
1	4.922567000	6.652196000	-0.308249000
6	6.482664000	5.289346000	0.305030000
1	6.528808000	5.683430000	1.329260000
1	6.614648000	4.204562000	0.397257000
6	7.622637000	5.877630000	-0.522446000
1	7.618462000	5.475715000	-1.542391000
1	7.535147000	6.967612000	-0.596302000
1	8.596473000	5.650136000	-0.075331000
6	3.101238000	2.057485000	3.650452000
1	2.868911000	2.732535000	2.817072000

1	2.949616000	2.626640000	4.575457000
6	4.551899000	1.582780000	3.561579000
1	4.779660000	0.965366000	4.442335000
1	4.671797000	0.923257000	2.689285000
6	5.569555000	2.717999000	3.462445000
1	5.479076000	3.203255000	2.481609000
1	5.340015000	3.487569000	4.213188000
6	7.006424000	2.235703000	3.656710000
1	7.121706000	1.846024000	4.676967000
1	7.191962000	1.390714000	2.981516000
6	8.039119000	3.329667000	3.399922000
1	7.991920000	3.670297000	2.358965000
1	7.859805000	4.199656000	4.042405000
1	9.057314000	2.975711000	3.595115000
6	8.754536000	0.908439000	0.776659000
1	9.195670000	1.483399000	1.602810000
6	7.665761000	1.711293000	0.135663000
1	7.982887000	2.630865000	-0.356890000
1	8.321504000	0.005934000	1.220231000
6	6.377393000	1.373987000	0.119952000
1	6.024396000	0.462132000	0.599385000
1	5.627951000	1.993776000	-0.365486000
6	11.998981000	-0.835330000	-0.502980000
1	11.549466000	-1.283680000	-1.403897000
1	12.563798000	0.043448000	-0.838187000
6	10.898696000	-0.402519000	0.459358000
1	10.388831000	-1.294793000	0.844054000
1	11.357953000	0.089485000	1.325697000
6	9.870850000	0.516672000	-0.196845000
1	10.360323000	1.422325000	-0.580757000
1	9.430058000	0.013356000	-1.066807000
8	12.914496000	-1.721624000	0.100387000
14	12.764196000	-3.386532000	0.118182000
6	12.418169000	-4.027706000	-1.615965000
1	13.189296000	-3.701114000	-2.322266000
1	12.406484000	-5.124146000	-1.616381000
1	11.447256000	-3.692921000	-1.997487000
6	11.385448000	-3.951094000	1.269436000
1	11.366422000	-5.046008000	1.331918000
1	11.538361000	-3.562936000	2.282607000
1	10.396386000	-3.625427000	0.927896000
6	14.410795000	-4.014883000	0.747756000
1	15.226401000	-3.716267000	0.081022000
1	14.627657000	-3.612425000	1.742961000
1	14.414104000	-5.108652000	0.819053000

E

E_1 = -5417.07200342 A.U.

At No.	X	Y	Z
6	-0.461235000	2.525671000	-3.473135000
6	-1.712434000	1.960403000	-3.733643000
6	0.677461000	1.734316000	-3.633273000
6	-1.801143000	0.644157000	-4.178402000
6	0.584083000	0.406556000	-4.047866000
6	-0.668606000	-0.144247000	-4.343571000
1	1.640535000	2.178038000	-3.400554000
1	-2.759896000	0.187497000	-4.391600000
6	-0.341412000	3.936517000	-2.935419000
1	0.586614000	4.389611000	-3.290425000
1	-1.173816000	4.542004000	-3.304684000
6	-0.355503000	3.921927000	-1.421515000
6	-1.538881000	3.627268000	-0.748430000

6	0.807596000	4.111850000	-0.673940000
6	-1.581534000	3.499877000	0.636316000
6	0.759725000	4.002194000	0.713619000
6	-0.420821000	3.708267000	1.387147000
1	-2.421995000	3.474252000	-1.354503000
1	1.659093000	4.113371000	1.301904000
6	-0.433300000	3.605480000	2.896028000
1	-1.347545000	4.065463000	3.283666000
1	0.409993000	4.180516000	3.292483000
6	-0.360946000	2.175712000	3.384616000
6	-1.513945000	1.518960000	3.809180000
6	0.837928000	1.466564000	3.377190000
6	-1.478800000	0.186982000	4.213230000
6	0.873783000	0.129580000	3.764562000
6	-0.274003000	-0.534434000	4.187596000
1	-2.443855000	2.073254000	3.789707000
1	1.814479000	-0.412042000	3.709351000
6	-0.233519000	-2.004332000	4.555276000
1	-0.979576000	-2.205354000	5.329899000
1	0.747234000	-2.245598000	4.970117000
6	-0.501607000	-2.891564000	3.354557000
6	0.513239000	-3.648062000	2.762408000
6	-1.778324000	-2.932418000	2.792846000
6	0.239395000	-4.388626000	1.611330000
6	-2.052601000	-3.686761000	1.656646000
6	-1.029934000	-4.419530000	1.046341000
1	-2.549196000	-2.339272000	3.268013000
1	1.018466000	-4.940631000	1.102144000
6	-1.276838000	-5.182622000	-0.235922000
1	-0.553056000	-5.999023000	-0.313949000
1	-2.277161000	-5.623791000	-0.216363000
6	-1.154524000	-4.279112000	-1.442922000
6	0.097131000	-3.976690000	-1.989719000
6	-2.286582000	-3.695570000	-2.002622000
6	0.187689000	-3.118357000	-3.082643000
6	-2.195498000	-2.827467000	-3.088783000
6	-0.946904000	-2.537396000	-3.646017000
1	-3.241030000	-3.923214000	-1.544797000
1	1.147341000	-2.855485000	-3.511417000
6	-0.808084000	-1.577917000	-4.807323000
1	0.068346000	-1.856540000	-5.398444000
1	-1.686203000	-1.658920000	-5.454006000
8	-2.590182000	-0.483097000	4.644855000
6	-3.837840000	0.159909000	4.478318000
1	-3.950040000	0.475878000	3.431649000
1	-3.886436000	1.062355000	5.105625000
8	-2.715875000	3.172321000	1.329364000
6	-3.916689000	3.091975000	0.589773000
1	-3.787984000	2.420697000	-0.266874000
1	-4.177598000	4.089731000	0.202683000
8	-2.813549000	2.755632000	-3.534719000
6	-4.073733000	2.119740000	-3.556050000
1	-4.296360000	1.750478000	-4.568991000
1	-4.061970000	1.254094000	-2.879209000
8	-3.284937000	-2.212110000	-3.654504000
6	-4.544966000	-2.455885000	-3.066344000
1	-4.806141000	-3.521471000	-3.153231000
1	-4.507883000	-2.208854000	-1.996907000
8	-3.288944000	-3.762593000	1.078455000
6	-4.287162000	-2.890499000	1.560843000
1	-4.484529000	-3.092702000	2.624209000
1	-3.948483000	-1.849512000	1.466636000

8	1.743764000	-3.630358000	3.364389000
6	2.794170000	-4.288379000	2.688666000
1	2.531485000	-5.337296000	2.490607000
1	2.957699000	-3.798460000	1.716306000
8	1.178078000	-4.573418000	-1.404738000
6	2.447772000	-4.351624000	-1.980382000
1	2.436938000	-4.653022000	-3.039074000
1	2.701702000	-3.281720000	-1.937827000
8	1.652836000	-0.428391000	-4.199739000
6	2.960309000	0.067706000	-3.981897000
1	3.163747000	0.906154000	-4.665059000
1	3.073174000	0.426162000	-2.951535000
8	1.954733000	4.406564000	-1.361335000
6	3.068968000	4.844244000	-0.610197000
1	2.754325000	5.590441000	0.134168000
1	3.508442000	3.995629000	-0.064422000
8	1.977551000	2.086756000	2.907116000
6	2.804632000	2.713717000	3.880107000
1	2.186212000	3.224364000	4.631298000
1	3.405240000	1.953342000	4.402254000
6	-5.561118000	-3.110068000	0.766289000
1	-5.323008000	-3.131243000	-0.302595000
1	-5.978122000	-4.093327000	1.016791000
6	-6.582556000	-2.008303000	1.052824000
1	-6.571025000	-1.766955000	2.125895000
1	-6.276664000	-1.086756000	0.535643000
6	-8.008712000	-2.380890000	0.653957000
1	-8.037255000	-2.666839000	-0.406442000
1	-8.315923000	-3.270962000	1.221349000
6	-9.004464000	-1.252671000	0.906987000
1	-8.896086000	-0.914103000	1.945801000
1	-8.739247000	-0.390649000	0.279349000
6	-10.455585000	-1.650810000	0.653337000
1	-10.597556000	-1.990791000	-0.378377000
1	-10.757782000	-2.468815000	1.317563000
1	-11.136041000	-0.810270000	0.825837000
6	-4.958906000	-0.791401000	4.850537000
1	-4.900167000	-1.682444000	4.214207000
1	-4.829943000	-1.128422000	5.885688000
6	-6.314159000	-0.106926000	4.670258000
1	-6.389278000	0.742991000	5.363170000
1	-6.367318000	0.321787000	3.659298000
6	-7.515257000	-1.028761000	4.870461000
1	-7.477279000	-1.841289000	4.130537000
1	-7.458543000	-1.511885000	5.855572000
6	-8.844587000	-0.284530000	4.736389000
1	-8.945014000	0.428995000	5.564822000
1	-8.823809000	0.320681000	3.820478000
6	-10.055111000	-1.214002000	4.704014000
1	-9.993394000	-1.907014000	3.856147000
1	-10.117248000	-1.814656000	5.618659000
1	-10.988724000	-0.650276000	4.607000000
6	-5.024328000	2.559853000	1.479971000
1	-4.805377000	1.516120000	1.740735000
1	-5.046923000	3.130983000	2.416534000
6	-6.376094000	2.654359000	0.773329000
1	-6.542624000	3.698838000	0.472971000
1	-6.345307000	2.071671000	-0.159544000
6	-7.563176000	2.191956000	1.616636000
1	-7.476801000	1.115766000	1.819773000
1	-7.535219000	2.694383000	2.594084000
6	-8.902649000	2.479851000	0.938385000

1	-8.945467000	3.547681000	0.683424000
1	-8.947917000	1.938980000	-0.016658000
6	-10.116127000	2.117761000	1.790720000
1	-10.132142000	1.048846000	2.026941000
1	-10.107297000	2.666532000	2.739598000
1	-11.049558000	2.358627000	1.271220000
6	-5.148976000	3.098450000	-3.122161000
1	-4.932861000	3.455633000	-2.107552000
1	-5.132299000	3.974965000	-3.780749000
6	-6.522084000	2.428483000	-3.165386000
1	-6.704309000	2.052831000	-4.182760000
1	-6.516421000	1.540887000	-2.513644000
6	-7.679485000	3.338139000	-2.759409000
1	-7.542027000	3.669606000	-1.721346000
1	-7.667429000	4.245912000	-3.378470000
6	-9.034043000	2.645542000	-2.899201000
1	-9.167572000	2.334866000	-3.944435000
1	-9.026138000	1.719462000	-2.308001000
6	-10.213154000	3.514291000	-2.470541000
1	-10.123382000	3.807389000	-1.418609000
1	-10.264189000	4.431243000	-3.068657000
1	-11.162182000	2.981222000	-2.590259000
6	-5.598012000	-1.600177000	-3.746857000
1	-5.246107000	-0.562259000	-3.792862000
1	-5.736561000	-1.937403000	-4.781191000
6	-6.916069000	-1.672372000	-2.973810000
1	-7.138228000	-2.723094000	-2.734678000
1	-6.792455000	-1.163265000	-2.005927000
6	-8.113964000	-1.077620000	-3.711163000
1	-7.909789000	-0.030159000	-3.970648000
1	-8.254967000	-1.610017000	-4.662113000
6	-9.398473000	-1.157793000	-2.887384000
1	-9.558524000	-2.199577000	-2.576503000
1	-9.265396000	-0.583157000	-1.960715000
6	-10.634577000	-0.654194000	-3.627759000
1	-10.514384000	0.391411000	-3.931259000
1	-10.817731000	-1.244410000	-4.532880000
1	-11.527634000	-0.719533000	-2.997366000
6	3.921980000	-1.079167000	-4.234220000
1	3.661502000	-1.903244000	-3.556959000
1	3.776928000	-1.449261000	-5.256983000
6	5.378511000	-0.678985000	-4.013734000
1	5.627123000	0.183921000	-4.648205000
1	5.498211000	-0.343972000	-2.976334000
6	6.354792000	-1.817364000	-4.308096000
1	6.077987000	-2.699533000	-3.713672000
1	6.254404000	-2.116165000	-5.361269000
6	7.812447000	-1.459190000	-4.020345000
1	8.072177000	-0.536142000	-4.556156000
1	7.924168000	-1.236064000	-2.951152000
6	8.786470000	-2.568623000	-4.411877000
1	8.559173000	-3.498824000	-3.878523000
1	8.727600000	-2.781514000	-5.485492000
1	9.820737000	-2.294124000	-4.179993000
6	4.083766000	5.458921000	-1.554995000
1	4.335831000	4.729241000	-2.331933000
1	3.623124000	6.315925000	-2.061198000
6	5.346120000	5.898624000	-0.813049000
1	5.087450000	6.665050000	-0.068200000
1	5.753162000	5.050788000	-0.244919000
6	6.432212000	6.441212000	-1.739669000
1	6.680800000	5.679127000	-2.492338000

1	6.042676000	7.304475000	-2.297365000
6	7.703816000	6.845662000	-0.995008000
1	7.457600000	7.613891000	-0.249640000
1	8.078630000	5.982329000	-0.428498000
6	8.800317000	7.364184000	-1.922710000
1	9.087689000	6.600869000	-2.654971000
1	8.460656000	8.245763000	-2.478477000
1	9.697618000	7.645995000	-1.362311000
6	3.698684000	3.691091000	3.132790000
1	4.082814000	3.170058000	2.247363000
1	3.089376000	4.523022000	2.758143000
6	4.876085000	4.242214000	3.933325000
1	4.521317000	4.934203000	4.708786000
1	5.384517000	3.422604000	4.457947000
6	5.876704000	4.950547000	3.018879000
1	6.206010000	4.245356000	2.240899000
1	5.371256000	5.768459000	2.485250000
6	7.106142000	5.495618000	3.741760000
1	6.793021000	6.234409000	4.491229000
1	7.589122000	4.679608000	4.295975000
6	8.111785000	6.126318000	2.780685000
1	8.466680000	5.389369000	2.050425000
1	7.656286000	6.950963000	2.220463000
1	8.984301000	6.520764000	3.311343000
6	4.060511000	-4.226384000	3.523834000
1	4.177969000	-3.209266000	3.907488000
1	3.969122000	-4.886140000	4.394539000
6	5.272956000	-4.607633000	2.673284000
1	5.227899000	-5.672904000	2.406863000
1	5.215056000	-4.060709000	1.722089000
6	6.622824000	-4.293133000	3.312925000
1	6.646600000	-3.231300000	3.599387000
1	6.745241000	-4.863489000	4.243934000
6	7.787515000	-4.584369000	2.366485000
1	7.802497000	-5.656595000	2.129589000
1	7.607240000	-4.064634000	1.415100000
6	9.142297000	-4.155547000	2.922914000
1	9.162802000	-3.074956000	3.107718000
1	9.359035000	-4.660184000	3.871443000
1	9.952384000	-4.390813000	2.224513000
6	3.480668000	-5.162977000	-1.217414000
1	3.622952000	-4.724485000	-0.221910000
1	3.097362000	-6.179566000	-1.068993000
6	4.810241000	-5.200282000	-1.970632000
1	4.692726000	-5.798107000	-2.885034000
1	5.062894000	-4.184970000	-2.303035000
6	5.972663000	-5.749957000	-1.146887000
1	6.082110000	-5.153382000	-0.231214000
1	5.745222000	-6.774644000	-0.820226000
6	7.293139000	-5.737388000	-1.916602000
1	7.191504000	-6.351495000	-2.821355000
1	7.489708000	-4.713691000	-2.264590000
6	8.480149000	-6.227327000	-1.090572000
1	8.643149000	-5.583546000	-0.219179000
1	8.310743000	-7.246332000	-0.723522000
1	9.402113000	-6.232453000	-1.681264000
6	-0.887855000	-0.694808000	-0.159368000
1	-0.641884000	-1.451457000	0.598857000
6	-2.243719000	-0.132678000	0.143281000
1	-2.366352000	0.326395000	1.127192000
1	-0.907832000	-1.206084000	-1.128744000
6	-3.265982000	-0.152655000	-0.711743000

1	-3.144504000	-0.570413000	-1.707746000
1	-4.246711000	0.247259000	-0.457181000
6	2.690801000	0.811729000	-0.334786000
1	2.535127000	1.602400000	-1.083942000
1	2.658370000	1.296045000	0.650285000
6	1.583068000	-0.224962000	-0.422257000
1	1.604146000	-0.700904000	-1.412503000
1	1.786119000	-1.016433000	0.313002000
6	0.204884000	0.377113000	-0.163019000
1	0.206599000	0.901886000	0.800522000
1	-0.025173000	1.127231000	-0.928471000
8	3.950069000	0.188473000	-0.572284000
14	5.344912000	0.480810000	0.299590000
6	4.912063000	0.385747000	2.139128000
1	4.040904000	1.041935000	2.251933000
6	6.009951000	2.194860000	-0.181706000
1	5.393395000	2.932175000	0.355206000
6	6.567153000	-0.845782000	-0.283308000
1	7.026103000	-0.419995000	-1.188722000
6	7.705812000	-1.132587000	0.708018000
1	7.326979000	-1.599817000	1.625017000
1	8.432224000	-1.829948000	0.269855000
1	8.250913000	-0.227967000	0.998359000
6	7.477395000	2.447559000	0.193691000
1	7.679967000	2.299705000	1.259470000
1	8.144075000	1.779946000	-0.365941000
1	7.768347000	3.477030000	-0.058024000
6	5.998476000	0.890713000	3.096696000
1	5.649777000	0.842351000	4.137391000
1	6.912825000	0.289846000	3.036213000
1	6.271219000	1.931378000	2.892832000
6	4.438425000	-1.015174000	2.535176000
1	4.097771000	-1.023884000	3.579205000
1	3.604962000	-1.359590000	1.912994000
1	5.245408000	-1.751522000	2.451243000
6	5.875810000	-2.150136000	-0.692737000
1	5.080111000	-1.976742000	-1.422567000
1	6.602193000	-2.850526000	-1.126345000
1	5.416821000	-2.645285000	0.170932000
6	5.818247000	2.420467000	-1.688971000
1	6.367659000	1.671131000	-2.272234000
1	4.765546000	2.358032000	-1.983851000
1	6.202814000	3.403112000	-1.989637000

E_{1a}

E_1 = -5417.06850979 A.U.

At No.	X	Y	Z
6	0.217778000	1.692373000	3.788457000
6	1.550195000	1.281442000	3.935103000
6	-0.784560000	0.734118000	3.938895000
6	1.848692000	-0.062809000	4.154152000
6	-0.486490000	-0.598038000	4.215755000
6	0.844649000	-1.015164000	4.292574000
1	-1.815632000	1.056382000	3.848001000
1	2.874249000	-0.408227000	4.210809000
6	-0.153393000	3.130555000	3.472172000
1	-1.160976000	3.332536000	3.842683000
1	0.529741000	3.803947000	4.001431000
6	-0.095903000	3.433582000	1.985415000
6	1.118272000	3.292698000	1.316442000
6	-1.207534000	3.856533000	1.250950000
6	1.246512000	3.526876000	-0.046514000

6	-1.089936000	4.037445000	-0.128294000
6	0.120499000	3.883196000	-0.793694000
1	1.973672000	3.006253000	1.910517000
1	-1.954524000	4.303795000	-0.719832000
6	0.207898000	4.083806000	-2.287899000
1	1.126254000	4.627905000	-2.529655000
1	-0.631199000	4.703649000	-2.615886000
6	0.211291000	2.764471000	-3.027532000
6	1.425009000	2.179831000	-3.381744000
6	-0.966681000	2.075285000	-3.309837000
6	1.472582000	0.937234000	-4.002499000
6	-0.921185000	0.837286000	-3.946837000
6	0.289058000	0.241345000	-4.290578000
1	2.330120000	2.708681000	-3.114685000
1	-1.848838000	0.303729000	-4.133535000
6	0.345566000	-1.156436000	-4.868030000
1	1.164474000	-1.223374000	-5.589903000
1	-0.586594000	-1.365399000	-5.398850000
6	0.550209000	-2.199749000	-3.786805000
6	-0.545191000	-2.804625000	-3.161464000
6	1.833554000	-2.557446000	-3.373504000
6	-0.339603000	-3.778899000	-2.186180000
6	2.036118000	-3.504009000	-2.371872000
6	0.941827000	-4.148130000	-1.788158000
1	2.670533000	-2.060040000	-3.848240000
1	-1.173841000	-4.258026000	-1.688807000
6	1.138704000	-5.182600000	-0.700645000
1	0.331221000	-5.917360000	-0.751530000
1	2.083295000	-5.708161000	-0.861792000
6	1.151831000	-4.533471000	0.664786000
6	-0.036501000	-4.332822000	1.373717000
6	2.338693000	-4.042380000	1.196930000
6	-0.006276000	-3.706906000	2.615847000
6	2.360255000	-3.347763000	2.405187000
6	1.184334000	-3.209864000	3.148267000
1	3.238726000	-4.179681000	0.611134000
1	-0.914009000	-3.562179000	3.188596000
6	1.186098000	-2.477704000	4.474554000
1	0.459280000	-2.944110000	5.144641000
1	2.171918000	-2.565280000	4.937645000
8	2.647950000	0.331952000	-4.357888000
6	3.835603000	0.877490000	-3.821057000
1	3.770116000	0.874727000	-2.722966000
1	3.958939000	1.921733000	-4.141412000
8	2.440892000	3.439296000	-0.708368000
6	3.591047000	3.291349000	0.096542000
1	3.509324000	2.382980000	0.706132000
1	3.673900000	4.151834000	0.778917000
8	2.517305000	2.252000000	3.857001000
6	3.865960000	1.838899000	3.921296000
1	4.056639000	1.314438000	4.869053000
1	4.080981000	1.135300000	3.102833000
8	3.493759000	-2.770580000	2.915331000
6	4.621717000	-2.736125000	2.064670000
1	5.040032000	-3.748412000	1.949419000
1	4.321129000	-2.378542000	1.071179000
8	3.270237000	-3.875701000	-1.911619000
6	4.395738000	-3.129164000	-2.316191000
1	4.524718000	-3.193231000	-3.407714000
1	4.261016000	-2.069634000	-2.050304000
8	-1.785760000	-2.399469000	-3.567585000
6	-2.904289000	-3.061125000	-3.007225000

1	-2.821137000	-4.144416000	-3.181756000
1	-2.942255000	-2.887734000	-1.922495000
8	-1.173685000	-4.781569000	0.763671000
6	-2.420345000	-4.664487000	1.416411000
1	-2.409796000	-5.236227000	2.357659000
1	-2.631776000	-3.612020000	1.658887000
8	-1.424215000	-1.556180000	4.478844000
6	-2.743034000	-1.409688000	3.995656000
1	-3.214939000	-0.498379000	4.390649000
1	-2.730079000	-1.331909000	2.898138000
8	-2.373114000	4.103920000	1.927704000
6	-3.391569000	4.775582000	1.213520000
1	-2.982383000	5.681814000	0.742255000
1	-3.779000000	4.134380000	0.405595000
8	-2.172758000	2.587348000	-2.878926000
6	-2.849382000	3.469748000	-3.767312000
1	-2.126457000	3.978905000	-4.418771000
1	-3.528623000	2.891762000	-4.410670000
6	5.614978000	-3.709584000	-1.621513000
1	5.483242000	-3.630849000	-0.535600000
1	5.671724000	-4.779043000	-1.858606000
6	6.906770000	-3.013240000	-2.043807000
1	6.977966000	-3.018246000	-3.140931000
1	6.875208000	-1.955920000	-1.742055000
6	8.160911000	-3.665685000	-1.464584000
1	8.110644000	-3.646418000	-0.367303000
1	8.183576000	-4.726698000	-1.750083000
6	9.450890000	-2.990072000	-1.924855000
1	9.507880000	-3.038682000	-3.020641000
1	9.411751000	-1.923975000	-1.666971000
6	10.706013000	-3.612323000	-1.318353000
1	10.689920000	-3.543099000	-0.224072000
1	10.787184000	-4.673246000	-1.581132000
1	11.610962000	-3.107513000	-1.671710000
6	5.036896000	0.077103000	-4.288316000
1	4.867097000	-0.991026000	-4.113149000
1	5.162762000	0.207981000	-5.369823000
6	6.286757000	0.541802000	-3.539196000
1	6.310208000	1.641357000	-3.516799000
1	6.208899000	0.223593000	-2.488649000
6	7.604326000	0.039988000	-4.124757000
1	7.598996000	-1.057138000	-4.172204000
1	7.698787000	0.394054000	-5.160760000
6	8.810443000	0.508794000	-3.312466000
1	8.781213000	1.602823000	-3.220865000
1	8.719348000	0.118849000	-2.288732000
6	10.151018000	0.084486000	-3.904669000
1	10.217155000	-1.005300000	-3.992643000
1	10.289177000	0.508887000	-4.905596000
1	10.984656000	0.421403000	-3.279268000
6	4.827711000	3.187459000	-0.772311000
1	4.734845000	2.311907000	-1.425017000
1	4.907653000	4.069003000	-1.420053000
6	6.068091000	3.059038000	0.110937000
1	6.156285000	3.961700000	0.731995000
1	5.927449000	2.221959000	0.811575000
6	7.373102000	2.848776000	-0.651843000
1	7.337486000	1.886810000	-1.179035000
1	7.479319000	3.621452000	-1.426736000
6	8.589850000	2.880395000	0.271125000
1	8.617305000	3.846429000	0.792988000
1	8.459292000	2.118950000	1.051595000

6	9.915439000	2.653288000	-0.450007000
1	9.952797000	1.657261000	-0.904995000
1	10.059000000	3.389659000	-1.249353000
1	10.761154000	2.735903000	0.240252000
6	4.773229000	3.049622000	3.807904000
1	4.583609000	3.558683000	2.855106000
1	4.528678000	3.760619000	4.605929000
6	6.240683000	2.630368000	3.898780000
1	6.410748000	2.106081000	4.849881000
1	6.456668000	1.899553000	3.106127000
6	7.228308000	3.789073000	3.781748000
1	7.107817000	4.272558000	2.802591000
1	6.990689000	4.554381000	4.533780000
6	8.680675000	3.343098000	3.952145000
1	8.810972000	2.915694000	4.955144000
1	8.892228000	2.530047000	3.245270000
6	9.683970000	4.474256000	3.740905000
1	9.600669000	4.889313000	2.729632000
1	9.510742000	5.292115000	4.449786000
1	10.713051000	4.125587000	3.875407000
6	5.673955000	-1.802867000	2.634789000
1	5.216848000	-0.827367000	2.843144000
1	6.044843000	-2.193759000	3.590087000
6	6.821160000	-1.644875000	1.636059000
1	7.171051000	-2.643333000	1.337909000
1	6.438075000	-1.173042000	0.719214000
6	8.015713000	-0.850121000	2.157099000
1	7.693707000	0.155775000	2.462265000
1	8.405534000	-1.333640000	3.063689000
6	9.135620000	-0.736729000	1.122367000
1	9.380600000	-1.740749000	0.751248000
1	8.767901000	-0.172373000	0.252968000
6	10.400812000	-0.081855000	1.670025000
1	10.195747000	0.925843000	2.049400000
1	10.815577000	-0.668477000	2.497539000
1	11.171518000	0.003986000	0.897324000
6	-3.511113000	-2.636059000	4.460547000
1	-2.975706000	-3.533771000	4.126646000
1	-3.497038000	-2.657061000	5.557111000
6	-4.947624000	-2.684852000	3.950559000
1	-5.489831000	-1.780851000	4.263392000
1	-4.942916000	-2.673652000	2.852349000
6	-5.699125000	-3.925679000	4.429916000
1	-5.167243000	-4.822667000	4.081731000
1	-5.681895000	-3.966132000	5.528032000
6	-7.145249000	-3.977138000	3.940320000
1	-7.695552000	-3.115258000	4.341143000
1	-7.157280000	-3.865197000	2.848978000
6	-7.856866000	-5.272640000	4.321314000
1	-7.340388000	-6.138982000	3.891981000
1	-7.882863000	-5.404583000	5.409143000
1	-8.888814000	-5.284916000	3.955466000
6	-4.521216000	5.142206000	2.157253000
1	-4.922627000	4.230095000	2.615966000
1	-4.127038000	5.759917000	2.972788000
6	-5.630830000	5.883800000	1.409833000
1	-5.245061000	6.840386000	1.030281000
1	-5.921325000	5.302093000	0.524170000
6	-6.874208000	6.136883000	2.259375000
1	-7.247108000	5.178814000	2.649121000
1	-6.604909000	6.741463000	3.136785000
6	-7.991579000	6.833122000	1.482194000

1	-7.614956000	7.782637000	1.078482000
1	-8.260232000	6.215862000	0.613643000
6	-9.235968000	7.092788000	2.328725000
1	-9.648514000	6.154849000	2.717987000
1	-9.000383000	7.732852000	3.186711000
1	-10.019777000	7.587811000	1.746301000
6	-3.622098000	4.470748000	-2.920132000
1	-4.084168000	3.919489000	-2.093254000
1	-2.914095000	5.175133000	-2.464633000
6	-4.701938000	5.249803000	-3.665274000
1	-4.264269000	5.790668000	-4.515859000
1	-5.435526000	4.548635000	-4.088083000
6	-5.415390000	6.236986000	-2.740365000
1	-5.806522000	5.692581000	-1.868620000
1	-4.683274000	6.954070000	-2.341992000
6	-6.560631000	6.999087000	-3.404161000
1	-6.175093000	7.558713000	-4.266725000
1	-7.288927000	6.280782000	-3.803516000
6	-7.260509000	7.955251000	-2.439635000
1	-7.676627000	7.412055000	-1.583128000
1	-6.558744000	8.700879000	-2.047898000
1	-8.080573000	8.490831000	-2.928723000
6	-4.163211000	-2.520700000	-3.654085000
1	-4.281596000	-1.466003000	-3.376424000
1	-4.056730000	-2.564385000	-4.744947000
6	-5.388996000	-3.313541000	-3.205362000
1	-5.273684000	-4.369144000	-3.489008000
1	-5.437078000	-3.297540000	-2.107799000
6	-6.700964000	-2.778480000	-3.777018000
1	-6.764809000	-1.695617000	-3.596317000
1	-6.704164000	-2.902112000	-4.868884000
6	-7.931004000	-3.458341000	-3.177177000
1	-7.892456000	-4.534905000	-3.391807000
1	-7.890070000	-3.362865000	-2.083695000
6	-9.246022000	-2.873325000	-3.685995000
1	-9.318648000	-1.807831000	-3.437738000
1	-9.325246000	-2.967306000	-4.775164000
1	-10.108377000	-3.380895000	-3.241432000
6	-3.463388000	-5.214407000	0.455523000
1	-3.581761000	-4.506329000	-0.375159000
1	-3.069618000	-6.143365000	0.025544000
6	-4.820747000	-5.486177000	1.100615000
1	-4.716992000	-6.272396000	1.861787000
1	-5.171448000	-4.591922000	1.631318000
6	-5.870094000	-5.902311000	0.069944000
1	-5.958232000	-5.113185000	-0.689850000
1	-5.521095000	-6.798512000	-0.462517000
6	-7.248466000	-6.173373000	0.669326000
1	-7.175728000	-6.990828000	1.399144000
1	-7.577740000	-5.290395000	1.232028000
6	-8.293836000	-6.515983000	-0.390058000
1	-8.411592000	-5.690345000	-1.101928000
1	-8.000723000	-7.405259000	-0.960066000
1	-9.271844000	-6.713145000	0.060861000
6	1.112157000	-0.825733000	0.216104000
1	1.013873000	-1.916416000	0.136076000
6	2.409015000	-0.439722000	-0.427484000
1	2.414999000	-0.486708000	-1.517868000
1	1.126045000	-0.587209000	1.287248000
6	3.521183000	-0.092338000	0.218002000
1	3.542551000	-0.046847000	1.305441000
1	4.444840000	0.149261000	-0.305882000

6	-2.565275000	-0.239564000	-0.860418000
1	-2.579947000	0.855592000	-0.780094000
1	-2.383245000	-0.477521000	-1.915262000
6	-1.423047000	-0.791166000	-0.023350000
1	-1.593855000	-0.572953000	1.040015000
1	-1.392134000	-1.885041000	-0.125608000
6	-0.093240000	-0.187902000	-0.474931000
1	0.008471000	-0.331621000	-1.558112000
1	-0.095224000	0.896792000	-0.303549000
8	-3.817962000	-0.807603000	-0.493622000
14	-5.189181000	0.047622000	-0.055732000
6	-5.262140000	1.653288000	-1.061087000
1	-4.285188000	2.138756000	-0.919237000
6	-5.086405000	0.369672000	1.806210000
1	-4.748350000	-0.591497000	2.214032000
6	-6.595486000	-1.134015000	-0.499061000
1	-6.342650000	-1.453560000	-1.516717000
6	-6.334117000	2.645024000	-0.588895000
1	-6.329821000	3.545596000	-1.218916000
1	-7.344225000	2.224584000	-0.638672000
1	-6.162586000	2.967901000	0.443868000
6	-5.394861000	1.347276000	-2.559761000
1	-4.513629000	0.814267000	-2.932455000
1	-6.280517000	0.737484000	-2.778004000
1	-5.491075000	2.272879000	-3.142250000
6	-8.010311000	-0.544745000	-0.546700000
1	-8.101239000	0.232962000	-1.312482000
1	-8.742764000	-1.326684000	-0.788954000
1	-8.310931000	-0.104057000	0.410713000
6	-6.536028000	-2.373954000	0.398573000
1	-7.252743000	-3.141488000	0.075963000
1	-5.536652000	-2.822410000	0.384590000
1	-6.776575000	-2.124927000	1.438681000
6	-6.403130000	0.722340000	2.510563000
1	-6.847165000	1.646006000	2.121262000
1	-7.150658000	-0.071555000	2.407461000
1	-6.234445000	0.874186000	3.585419000
6	-4.006497000	1.413760000	2.102079000
1	-3.909928000	1.600703000	3.180304000
1	-3.019714000	1.120533000	1.727035000
1	-4.251018000	2.372015000	1.639725000

E_{1b}

E_1 = -5417.06876329 A.U.

At No.	X	Y	Z
6	0.258888000	1.346762000	3.950554000
6	1.577588000	0.893525000	4.091401000
6	-0.769746000	0.407392000	4.019454000
6	1.840514000	-0.468797000	4.227536000
6	-0.508381000	-0.947110000	4.210340000
6	0.810833000	-1.402404000	4.280730000
1	-1.790627000	0.761380000	3.930386000
1	2.856930000	-0.841287000	4.280684000
6	-0.067454000	2.811991000	3.725461000
1	-1.073267000	3.018459000	4.098218000
1	0.627442000	3.429387000	4.305190000
6	0.014346000	3.213212000	2.263253000
6	1.220032000	3.055313000	1.582702000
6	-1.073231000	3.745659000	1.564442000
6	1.363509000	3.378628000	0.238980000
6	-0.938735000	4.035594000	0.206322000
6	0.263213000	3.867140000	-0.471280000

1	2.057603000	2.679666000	2.151636000
1	-1.782478000	4.399677000	-0.362474000
6	0.359518000	4.197473000	-1.941742000
1	1.302454000	4.717378000	-2.138382000
1	-0.450212000	4.884312000	-2.206000000
6	0.297063000	2.955124000	-2.800942000
6	1.479445000	2.373588000	-3.252007000
6	-0.910650000	2.332585000	-3.108850000
6	1.469366000	1.196269000	-3.991140000
6	-0.923173000	1.152554000	-3.848871000
6	0.255893000	0.555688000	-4.285420000
1	2.407667000	2.851721000	-2.969787000
1	-1.872316000	0.663528000	-4.049765000
6	0.246867000	-0.798585000	-4.959689000
1	1.040173000	-0.843604000	-5.711319000
1	-0.707918000	-0.945267000	-5.469857000
6	0.454561000	-1.909659000	-3.948549000
6	-0.632482000	-2.573102000	-3.371922000
6	1.739932000	-2.262160000	-3.541909000
6	-0.413556000	-3.604153000	-2.458799000
6	1.956451000	-3.262305000	-2.597455000
6	0.872854000	-3.969684000	-2.069328000
1	2.566733000	-1.717027000	-3.978814000
1	-1.242567000	-4.133959000	-2.005642000
6	1.088552000	-5.081462000	-1.063933000
1	0.294410000	-5.824283000	-1.176516000
1	2.041084000	-5.576726000	-1.269706000
6	1.095908000	-4.558887000	0.355290000
6	-0.098313000	-4.413852000	1.068992000
6	2.284247000	-4.145356000	0.949226000
6	-0.076416000	-3.911613000	2.366194000
6	2.299238000	-3.584454000	2.225197000
6	1.114229000	-3.493191000	2.960695000
1	3.191209000	-4.236004000	0.364318000
1	-0.989925000	-3.805926000	2.938111000
6	1.109259000	-2.884163000	4.348137000
1	0.355295000	-3.384889000	4.960769000
1	2.082799000	-3.039611000	4.819081000
8	2.612950000	0.607496000	-4.460728000
6	3.836203000	1.132950000	-3.989075000
1	3.869811000	1.045591000	-2.893310000
1	3.911410000	2.200809000	-4.237901000
8	2.546117000	3.249263000	-0.439593000
6	3.699618000	3.010624000	0.340560000
1	3.557279000	2.121204000	0.964906000
1	3.876617000	3.871088000	1.005038000
8	2.565107000	1.844887000	4.087511000
6	3.907144000	1.405735000	4.105569000
1	4.106580000	0.829655000	5.021318000
1	4.096019000	0.744898000	3.246398000
8	3.431806000	-3.095066000	2.821351000
6	4.616433000	-3.082832000	2.053044000
1	4.975411000	-4.111181000	1.891363000
1	4.415193000	-2.635920000	1.071207000
8	3.200889000	-3.620105000	-2.151347000
6	4.279578000	-2.786007000	-2.507605000
1	4.420266000	-2.791775000	-3.599343000
1	4.068534000	-1.750695000	-2.200752000
8	-1.875659000	-2.161836000	-3.762764000
6	-2.994081000	-2.845736000	-3.231254000
1	-2.924658000	-3.917954000	-3.469480000
1	-3.020680000	-2.735219000	-2.138129000

8	-1.235725000	-4.788397000	0.410616000
6	-2.482748000	-4.717107000	1.069714000
1	-2.477219000	-5.365491000	1.959934000
1	-2.685200000	-3.686937000	1.400009000
8	-1.472688000	-1.898330000	4.388947000
6	-2.779893000	-1.682510000	3.901687000
1	-3.235835000	-0.792316000	4.358984000
1	-2.748325000	-1.521788000	2.813673000
8	-2.230413000	3.986642000	2.257812000
6	-3.232106000	4.720349000	1.583399000
1	-2.810127000	5.657992000	1.191014000
1	-3.609242000	4.146691000	0.721767000
8	-2.087428000	2.851402000	-2.609905000
6	-2.756475000	3.799314000	-3.433745000
1	-2.027223000	4.352442000	-4.041302000
1	-3.434601000	3.274621000	-4.122554000
6	5.545401000	-3.285506000	-1.836662000
1	5.378012000	-3.373845000	-0.757048000
1	5.778653000	-4.290408000	-2.209029000
6	6.704721000	-2.330644000	-2.115130000
1	6.732375000	-2.102091000	-3.190674000
1	6.512222000	-1.372390000	-1.608985000
6	8.075550000	-2.855474000	-1.694473000
1	8.085456000	-3.049794000	-0.613720000
1	8.264939000	-3.819908000	-2.185781000
6	9.187644000	-1.872308000	-2.051261000
1	9.157019000	-1.679840000	-3.131442000
1	8.970438000	-0.909845000	-1.569145000
6	10.586200000	-2.338216000	-1.659516000
1	10.661128000	-2.502731000	-0.579272000
1	10.840248000	-3.280344000	-2.158558000
1	11.341392000	-1.595891000	-1.939167000
6	5.009713000	0.403766000	-4.614757000
1	4.927429000	-0.673673000	-4.428340000
1	4.985970000	0.545777000	-5.701920000
6	6.313977000	0.947927000	-4.027354000
1	6.283953000	2.047421000	-4.040728000
1	6.374190000	0.662681000	-2.968544000
6	7.582639000	0.489066000	-4.741376000
1	7.657125000	-0.606723000	-4.700508000
1	7.520930000	0.752169000	-5.806638000
6	8.840702000	1.111453000	-4.134104000
1	8.762784000	2.205563000	-4.194436000
1	8.880535000	0.869459000	-3.064732000
6	10.130846000	0.654596000	-4.810297000
1	10.249906000	-0.433042000	-4.742421000
1	10.134842000	0.921597000	-5.873358000
1	11.008141000	1.115383000	-4.344544000
6	4.897510000	2.771000000	-0.557668000
1	4.702038000	1.885158000	-1.172914000
1	5.037974000	3.617151000	-1.241379000
6	6.148178000	2.554959000	0.294235000
1	6.348404000	3.471596000	0.866594000
1	5.944175000	1.772182000	1.040163000
6	7.405527000	2.175222000	-0.486087000
1	7.266164000	1.191054000	-0.956602000
1	7.564919000	2.887810000	-1.307628000
6	8.642762000	2.138935000	0.410948000
1	8.743217000	3.108450000	0.917783000
1	8.480371000	1.398168000	1.205397000
6	9.938674000	1.821434000	-0.329879000
1	9.907221000	0.824535000	-0.783489000

1	10.119395000	2.544539000	-1.133645000
1	10.797707000	1.847338000	0.348343000
6	4.822813000	2.613497000	4.037237000
1	4.623501000	3.164168000	3.109757000
1	4.589137000	3.290708000	4.867244000
6	6.290561000	2.191837000	4.092291000
1	6.482869000	1.657221000	5.033222000
1	6.490778000	1.470922000	3.286474000
6	7.269784000	3.356457000	3.963508000
1	7.115670000	3.853290000	2.995495000
1	7.051625000	4.109958000	4.733129000
6	8.728604000	2.916140000	4.079184000
1	8.897553000	2.479569000	5.072351000
1	8.915550000	2.111733000	3.356534000
6	9.718866000	4.052981000	3.839689000
1	9.594827000	4.475711000	2.835577000
1	9.569838000	4.864732000	4.560960000
1	10.753673000	3.707468000	3.931679000
6	5.668349000	-2.259410000	2.773418000
1	5.257498000	-1.264068000	2.986768000
1	5.904430000	-2.721400000	3.739706000
6	6.927659000	-2.132378000	1.917772000
1	7.284013000	-3.138531000	1.652827000
1	6.670667000	-1.640941000	0.967266000
6	8.068453000	-1.365955000	2.584010000
1	7.744821000	-0.339420000	2.807856000
1	8.307355000	-1.828735000	3.551605000
6	9.322908000	-1.330869000	1.712566000
1	9.627329000	-2.363019000	1.492392000
1	9.074290000	-0.875071000	0.744615000
6	10.492501000	-0.581834000	2.344363000
1	10.232045000	0.463612000	2.545132000
1	10.785029000	-1.038761000	3.296542000
1	11.366403000	-0.585890000	1.684501000
6	-3.584187000	-2.920645000	4.261765000
1	-3.070879000	-3.803453000	3.860040000
1	-3.578794000	-3.029105000	5.353244000
6	-5.017897000	-2.888262000	3.742805000
1	-5.534644000	-1.993341000	4.118465000
1	-5.005621000	-2.796914000	2.648484000
6	-5.808720000	-4.137527000	4.127981000
1	-5.301944000	-5.022516000	3.717295000
1	-5.798159000	-4.257660000	5.220367000
6	-7.253334000	-4.110596000	3.632387000
1	-7.778078000	-3.259929000	4.087799000
1	-7.256704000	-3.926889000	2.550855000
6	-8.007251000	-5.405051000	3.925558000
1	-7.516610000	-6.257324000	3.441305000
1	-8.042260000	-5.607267000	5.002279000
1	-9.037450000	-5.360246000	3.557326000
6	-4.378448000	5.019140000	2.530556000
1	-4.791020000	4.075753000	2.908811000
1	-4.002243000	5.571598000	3.399466000
6	-5.468767000	5.817072000	1.812856000
1	-5.088548000	6.815720000	1.556441000
1	-5.700970000	5.328230000	0.856810000
6	-6.759936000	5.952316000	2.616337000
1	-7.123530000	4.950379000	2.886550000
1	-6.554857000	6.470558000	3.563543000
6	-7.855972000	6.695034000	1.851928000
1	-7.488586000	7.689972000	1.566418000
1	-8.057963000	6.161791000	0.912408000

6	-9.151245000	6.834874000	2.648816000
1	-9.553988000	5.851799000	2.918656000
1	-8.982175000	7.390557000	3.578307000
1	-9.918206000	7.365724000	2.075575000
6	-3.528527000	4.740447000	-2.521254000
1	-4.026950000	4.133625000	-1.756878000
1	-2.816126000	5.385048000	-1.990012000
6	-4.565638000	5.606884000	-3.230245000
1	-4.084378000	6.211267000	-4.011811000
1	-5.295040000	4.962981000	-3.742042000
6	-5.297862000	6.524578000	-2.250430000
1	-5.758102000	5.913075000	-1.460970000
1	-4.566955000	7.169980000	-1.742284000
6	-6.375248000	7.392562000	-2.897969000
1	-5.919198000	8.019307000	-3.675819000
1	-7.100985000	6.746350000	-3.409461000
6	-7.101155000	8.275559000	-1.884277000
1	-7.590328000	7.667364000	-1.114581000
1	-6.399920000	8.948291000	-1.376721000
1	-7.868623000	8.891151000	-2.364494000
6	-4.249153000	-2.249539000	-3.835405000
1	-4.337956000	-1.206572000	-3.506685000
1	-4.155599000	-2.243444000	-4.928362000
6	-5.490243000	-3.030344000	-3.410291000
1	-5.404532000	-4.075879000	-3.738454000
1	-5.530173000	-3.057430000	-2.312880000
6	-6.790568000	-2.436580000	-3.949857000
1	-6.826321000	-1.362541000	-3.716048000
1	-6.799541000	-2.506106000	-5.046496000
6	-8.036431000	-3.113752000	-3.380572000
1	-8.028759000	-4.177607000	-3.653827000
1	-7.988853000	-3.078950000	-2.283860000
6	-9.336687000	-2.465600000	-3.849366000
1	-9.379552000	-1.414450000	-3.540406000
1	-9.421799000	-2.495160000	-4.941746000
1	-10.211160000	-2.974227000	-3.430420000
6	-3.533524000	-5.175701000	0.070746000
1	-3.644017000	-4.404061000	-0.702106000
1	-3.155263000	-6.074889000	-0.430594000
6	-4.892611000	-5.474806000	0.700523000
1	-4.796614000	-6.314193000	1.403765000
1	-5.229020000	-4.615518000	1.294907000
6	-5.952458000	-5.803059000	-0.350794000
1	-6.036525000	-4.959696000	-1.050089000
1	-5.617044000	-6.661919000	-0.949412000
6	-7.329839000	-6.101912000	0.237536000
1	-7.261355000	-6.970240000	0.906515000
1	-7.645496000	-5.257945000	0.864175000
6	-8.386085000	-6.356625000	-0.835688000
1	-8.499673000	-5.480659000	-1.485234000
1	-8.106618000	-7.205582000	-1.470355000
1	-9.363030000	-6.575573000	-0.392565000
6	1.024112000	-0.877813000	0.235316000
1	0.931901000	-1.951428000	0.025599000
6	2.371792000	-0.441370000	-0.255342000
1	2.439632000	-0.226940000	-1.324105000
1	0.962546000	-0.767759000	1.325751000
6	3.462160000	-0.357842000	0.505381000
1	3.407906000	-0.571716000	1.571182000
1	4.437345000	-0.093945000	0.099208000
6	-2.595912000	-0.171118000	-0.938216000
1	-2.590070000	0.918962000	-0.805421000

1	-2.395894000	-0.362037000	-1.998819000
6	-1.486195000	-0.789331000	-0.105220000
1	-1.697118000	-0.650462000	0.964274000
1	-1.455464000	-1.872827000	-0.286537000
6	-0.135769000	-0.163553000	-0.453687000
1	0.013080000	-0.217333000	-1.540244000
1	-0.137962000	0.902488000	-0.189935000
8	-3.869082000	-0.728115000	-0.630442000
14	-5.198539000	0.126228000	-0.079216000
6	-5.248152000	1.808878000	-0.950795000
1	-4.260516000	2.261015000	-0.776194000
6	-5.037514000	0.304353000	1.798913000
1	-4.699081000	-0.687921000	2.124045000
6	-6.643317000	-0.986680000	-0.574819000
1	-6.419931000	-1.233039000	-1.619577000
6	-6.297058000	2.780681000	-0.392976000
1	-6.278404000	3.728465000	-0.949080000
1	-7.315586000	2.385467000	-0.469440000
1	-6.113999000	3.017027000	0.661072000
6	-5.394200000	1.628583000	-2.468335000
1	-4.534793000	1.093406000	-2.886767000
1	-6.302395000	1.070697000	-2.729552000
1	-5.456968000	2.600749000	-2.975071000
6	-8.045608000	-0.367556000	-0.545000000
1	-8.135831000	0.466664000	-1.248878000
1	-8.799879000	-1.114348000	-0.827919000
1	-8.315581000	0.006117000	0.449392000
6	-6.590139000	-2.291937000	0.225845000
1	-7.325863000	-3.021642000	-0.139141000
1	-5.599449000	-2.755035000	0.160237000
1	-6.808178000	-2.117026000	1.285560000
6	-6.333408000	0.613757000	2.560561000
1	-6.775106000	1.569371000	2.254455000
1	-7.092914000	-0.161839000	2.415137000
1	-6.137594000	0.680655000	3.639485000
6	-3.942988000	1.316623000	2.149837000
1	-3.825163000	1.421609000	3.236828000
1	-2.964739000	1.048940000	1.735723000
1	-4.191750000	2.308141000	1.765880000

E_{1c}

E_1 = -5417.02502461 A.U.

At No.	X	Y	Z
6	0.837993000	-1.870574000	4.102853000
6	-0.296740000	-1.135430000	4.451592000
6	2.040346000	-1.193876000	3.928936000
6	-0.196163000	0.243441000	4.651263000
6	2.126240000	0.186211000	4.064575000
6	1.004989000	0.922825000	4.458977000
1	2.903608000	-1.785291000	3.652882000
1	-1.064319000	0.830240000	4.927404000
6	0.781045000	-3.356901000	3.817559000
1	1.637031000	-3.850673000	4.286486000
1	-0.127715000	-3.782254000	4.248576000
6	0.798933000	-3.613570000	2.324391000
6	-0.385133000	-3.631063000	1.600674000
6	2.002585000	-3.753667000	1.613961000
6	-0.397177000	-3.806767000	0.216982000
6	1.990467000	-3.917944000	0.233764000
6	0.794515000	-3.953182000	-0.487083000
1	-1.324784000	-3.476291000	2.125731000
1	2.911235000	-4.011641000	-0.328094000

6	0.826033000	-4.110147000	-1.992695000
1	-0.102019000	-4.579575000	-2.328585000
1	1.651674000	-4.774147000	-2.265241000
6	0.986350000	-2.783751000	-2.706670000
6	-0.136051000	-2.060168000	-3.100197000
6	2.253162000	-2.228267000	-2.936585000
6	-0.021071000	-0.818326000	-3.722875000
6	2.365641000	-0.999644000	-3.578653000
6	1.241454000	-0.287902000	-3.990324000
1	-1.114216000	-2.472494000	-2.871326000
1	3.330446000	-0.542653000	-3.758913000
6	1.411398000	1.066036000	-4.642614000
1	0.606854000	1.227905000	-5.365366000
1	2.359953000	1.089043000	-5.185253000
6	1.378216000	2.169905000	-3.609275000
6	2.536147000	2.602527000	-2.958329000
6	0.156082000	2.712834000	-3.230637000
6	2.453002000	3.591336000	-1.981669000
6	0.064619000	3.669276000	-2.224212000
6	1.226863000	4.139736000	-1.607167000
1	-0.728064000	2.321223000	-3.715110000
1	3.336767000	3.938855000	-1.458773000
6	1.158776000	5.169771000	-0.501238000
1	2.019817000	5.838910000	-0.575861000
1	0.253761000	5.771133000	-0.617914000
6	1.149103000	4.507705000	0.858463000
6	2.339881000	4.255364000	1.543976000
6	-0.053516000	4.077159000	1.415821000
6	2.305530000	3.619636000	2.782330000
6	-0.083124000	3.409233000	2.636435000
6	1.107529000	3.177926000	3.335852000
1	-0.960488000	4.255476000	0.849160000
1	3.216370000	3.414173000	3.331292000
6	1.093694000	2.423513000	4.647720000
1	2.005365000	2.664690000	5.204040000
1	0.242814000	2.753883000	5.251145000
8	-1.103943000	-0.025857000	-4.016026000
6	-2.251062000	-0.591899000	-4.619672000
1	-2.610986000	-1.462486000	-4.054670000
1	-2.010935000	-0.930567000	-5.638564000
8	-1.595589000	-3.800177000	-0.462680000
6	-2.486861000	-4.870414000	-0.166880000
1	-3.486298000	-4.433569000	-0.092259000
1	-2.240754000	-5.307805000	0.808545000
8	-1.464645000	-1.838803000	4.572661000
6	-2.626877000	-1.130676000	4.942048000
1	-2.506858000	-0.697051000	5.946556000
1	-2.801815000	-0.303183000	4.241951000
8	-1.228527000	2.956180000	3.226879000
6	-2.457806000	3.215530000	2.582905000
1	-2.591504000	4.299655000	2.445009000
1	-2.474451000	2.745945000	1.587453000
8	-1.130017000	4.181857000	-1.792156000
6	-2.297102000	3.634630000	-2.369906000
1	-2.287133000	3.799123000	-3.458095000
1	-2.328031000	2.548446000	-2.202250000
8	3.710207000	2.009731000	-3.343872000
6	4.864302000	2.226234000	-2.561357000
1	5.126120000	3.294867000	-2.542249000
1	4.683319000	1.910651000	-1.521574000
8	3.495038000	4.615665000	0.895403000
6	4.709732000	4.745922000	1.602901000

1	4.702838000	5.674888000	2.192142000
1	4.863167000	3.913219000	2.297793000
8	3.279622000	0.881389000	3.820987000
6	4.315307000	0.175053000	3.176419000
1	4.787894000	-0.521233000	3.886782000
1	3.909377000	-0.421282000	2.346508000
8	3.159851000	-3.714201000	2.345935000
6	4.374039000	-3.629680000	1.630932000
1	4.526283000	-4.540653000	1.032408000
1	4.336843000	-2.777123000	0.934793000
8	3.342506000	-2.946061000	-2.510960000
6	4.587590000	-2.279393000	-2.541572000
1	4.837321000	-1.990550000	-3.573059000
1	4.526922000	-1.355139000	-1.945452000
6	-3.521535000	4.305548000	-1.779038000
1	-3.547191000	4.150361000	-0.693197000
1	-3.441659000	5.386620000	-1.947909000
6	-4.792636000	3.758070000	-2.435050000
1	-4.612128000	3.637613000	-3.513618000
1	-5.007067000	2.751661000	-2.048132000
6	-6.016814000	4.654640000	-2.257202000
1	-6.289709000	4.713054000	-1.193659000
1	-5.764776000	5.679910000	-2.562706000
6	-7.211499000	4.167727000	-3.074829000
1	-6.929686000	4.149882000	-4.136350000
1	-7.429736000	3.126311000	-2.808668000
6	-8.463830000	5.020651000	-2.895034000
1	-8.766588000	5.058757000	-1.842501000
1	-8.289699000	6.051257000	-3.224527000
1	-9.304833000	4.620622000	-3.472192000
6	-3.322141000	0.487142000	-4.625859000
1	-3.540806000	0.750366000	-3.582396000
1	-2.914792000	1.391894000	-5.094127000
6	-4.606463000	0.055374000	-5.337007000
1	-4.459596000	0.087009000	-6.424811000
1	-4.832338000	-0.992457000	-5.092618000
6	-5.807364000	0.917200000	-4.946828000
1	-5.941861000	0.856845000	-3.857089000
1	-5.592134000	1.973028000	-5.164518000
6	-7.102929000	0.504671000	-5.644302000
1	-6.985755000	0.628005000	-6.729106000
1	-7.276580000	-0.565936000	-5.474377000
6	-8.318933000	1.296299000	-5.166541000
1	-8.486266000	1.149372000	-4.092365000
1	-8.183100000	2.370714000	-5.334000000
1	-9.228904000	0.986068000	-5.690371000
6	-2.456734000	-5.930235000	-1.256690000
1	-2.608707000	-5.435502000	-2.224768000
1	-1.460393000	-6.390698000	-1.285434000
6	-3.527727000	-6.999368000	-1.046831000
1	-3.378506000	-7.485375000	-0.072280000
1	-4.516160000	-6.518378000	-1.000647000
6	-3.537687000	-8.061035000	-2.145932000
1	-3.685628000	-7.572545000	-3.119502000
1	-2.551780000	-8.544623000	-2.192804000
6	-4.615300000	-9.126746000	-1.945894000
1	-4.465952000	-9.613371000	-0.972788000
1	-5.599112000	-8.640807000	-1.898048000
6	-4.619025000	-10.181782000	-3.050588000
1	-4.796808000	-9.723702000	-4.030420000
1	-3.656221000	-10.703470000	-3.098440000
1	-5.398882000	-10.932164000	-2.884913000

6	-3.803322000	-2.086858000	4.907242000
1	-3.834511000	-2.564378000	3.919007000
1	-3.648667000	-2.886913000	5.641292000
6	-5.119482000	-1.359711000	5.173145000
1	-5.078017000	-0.861945000	6.152365000
1	-5.242082000	-0.556315000	4.432362000
6	-6.338506000	-2.277773000	5.122535000
1	-6.343561000	-2.819478000	4.166040000
1	-6.256750000	-3.042542000	5.907542000
6	-7.655433000	-1.519172000	5.277550000
1	-7.655965000	-0.984285000	6.237010000
1	-7.715693000	-0.748176000	4.498546000
6	-8.880856000	-2.425756000	5.193556000
1	-8.914853000	-2.950236000	4.230961000
1	-8.864231000	-3.186260000	5.982756000
1	-9.809161000	-1.854390000	5.296155000
6	-3.571586000	2.651760000	3.444988000
1	-3.474589000	1.560993000	3.479072000
1	-3.444797000	3.013551000	4.472519000
6	-4.954691000	3.043823000	2.924692000
1	-5.113810000	4.115341000	3.109222000
1	-5.002023000	2.910531000	1.833854000
6	-6.087151000	2.236305000	3.564097000
1	-6.128144000	1.245471000	3.089428000
1	-5.868449000	2.056717000	4.626383000
6	-7.447603000	2.923209000	3.442503000
1	-7.453720000	3.817013000	4.079955000
1	-7.578364000	3.285998000	2.415306000
6	-8.613531000	2.012456000	3.817922000
1	-8.674772000	1.153804000	3.137263000
1	-8.494884000	1.618482000	4.833967000
1	-9.570984000	2.543165000	3.775203000
6	5.348728000	1.147306000	2.643215000
1	4.879274000	1.791736000	1.890542000
1	5.693353000	1.792746000	3.461607000
6	6.530710000	0.389718000	2.040255000
1	6.868318000	-0.371104000	2.758261000
1	6.199012000	-0.161701000	1.147697000
6	7.720462000	1.274705000	1.681566000
1	7.432364000	1.991923000	0.900037000
1	8.002979000	1.872244000	2.560182000
6	8.929925000	0.466432000	1.217514000
1	9.149354000	-0.308419000	1.964046000
1	8.669747000	-0.067272000	0.292257000
6	10.172986000	1.321499000	0.995275000
1	9.975419000	2.117776000	0.270428000
1	10.492350000	1.794600000	1.931015000
1	11.009723000	0.724784000	0.616808000
6	5.524075000	-3.460265000	2.605606000
1	5.378632000	-2.540259000	3.184084000
1	5.517436000	-4.291895000	3.320079000
6	6.858150000	-3.409678000	1.862257000
1	6.987353000	-4.341069000	1.292324000
1	6.832959000	-2.598400000	1.119492000
6	8.069892000	-3.208353000	2.769244000
1	7.975452000	-2.249771000	3.299003000
1	8.085440000	-3.985679000	3.545641000
6	9.386488000	-3.233409000	1.994006000
1	9.509387000	-4.222042000	1.531256000
1	9.327635000	-2.514881000	1.166132000
6	10.604805000	-2.913805000	2.855814000
1	10.525782000	-1.909877000	3.289868000

1	10.702008000	-3.625699000	3.683373000
1	11.527915000	-2.951899000	2.268188000
6	5.685631000	-3.182322000	-2.009945000
1	5.433342000	-3.530125000	-1.001518000
1	5.758617000	-4.071932000	-2.647082000
6	7.018335000	-2.429368000	-1.995151000
1	7.160847000	-1.938998000	-2.969150000
1	6.971632000	-1.616811000	-1.253923000
6	8.236785000	-3.305404000	-1.711448000
1	8.128870000	-3.789591000	-0.732044000
1	8.281204000	-4.114989000	-2.453235000
6	9.543003000	-2.512812000	-1.749782000
1	9.625466000	-2.008399000	-2.721806000
1	9.502033000	-1.713050000	-0.998074000
6	10.783718000	-3.369305000	-1.512757000
1	10.745490000	-3.854484000	-0.531093000
1	10.868361000	-4.156758000	-2.270364000
1	11.695676000	-2.764639000	-1.553457000
6	5.995667000	1.434929000	-3.194705000
1	5.712810000	0.375739000	-3.243059000
1	6.107186000	1.777237000	-4.230974000
6	7.324770000	1.586104000	-2.454663000
1	7.506166000	2.648382000	-2.234943000
1	7.271655000	1.075667000	-1.481513000
6	8.503254000	1.040422000	-3.262058000
1	8.316830000	-0.012210000	-3.517354000
1	8.558805000	1.580771000	-4.217534000
6	9.842318000	1.153767000	-2.538386000
1	9.992127000	2.194529000	-2.219122000
1	9.806080000	0.553431000	-1.622095000
6	11.027755000	0.707489000	-3.390527000
1	10.914781000	-0.335348000	-3.708908000
1	11.117006000	1.321550000	-4.293965000
1	11.968287000	0.787096000	-2.835644000
6	5.828148000	4.745234000	0.573744000
1	5.797233000	3.785074000	0.039402000
1	5.628976000	5.525958000	-0.170886000
6	7.208276000	4.948342000	1.194945000
1	7.251308000	5.931511000	1.683617000
1	7.368491000	4.203395000	1.986816000
6	8.337696000	4.842115000	0.170780000
1	8.299710000	3.853228000	-0.310242000
1	8.173168000	5.576581000	-0.630480000
6	9.724827000	5.052178000	0.776125000
1	9.756766000	6.029170000	1.276363000
1	9.892535000	4.300119000	1.558434000
6	10.843781000	4.973688000	-0.260373000
1	10.853050000	3.997806000	-0.760369000
1	10.716269000	5.739507000	-1.034068000
1	11.826442000	5.120476000	0.199306000
6	-9.682939000	3.428203000	0.371269000
1	-8.981701000	4.273644000	0.309504000
6	-11.049825000	3.877200000	-0.056644000
1	-11.124124000	4.266189000	-1.073432000
1	-9.707402000	3.118075000	1.422401000
6	-12.143707000	3.811742000	0.696670000
1	-12.110094000	3.426054000	1.713738000
1	-13.111578000	4.142097000	0.330965000
6	-7.193736000	0.763934000	-1.017162000
1	-7.866064000	-0.098863000	-0.886133000
1	-7.233354000	1.036234000	-2.082015000
6	-7.698273000	1.920535000	-0.172110000

1	-7.599734000	1.647382000	0.885278000
1	-7.044848000	2.787422000	-0.331030000
6	-9.147738000	2.279462000	-0.491749000
1	-9.235536000	2.551847000	-1.552646000
1	-9.790449000	1.400784000	-0.347134000
8	-5.865782000	0.439183000	-0.668023000
14	-5.304180000	-1.145021000	-0.571396000
6	-5.243666000	-1.856757000	-2.330001000
1	-4.760945000	-1.046203000	-2.892453000
6	-6.498774000	-2.140726000	0.531725000
1	-7.355655000	-2.406549000	-0.105678000
6	-3.556131000	-1.004160000	0.119250000
1	-3.145021000	-2.020156000	0.201914000
6	-3.532591000	-0.359041000	1.510006000
1	-3.986184000	0.638464000	1.478842000
1	-4.081643000	-0.952011000	2.249925000
1	-2.501442000	-0.249507000	1.871728000
6	-2.642153000	-0.223220000	-0.837799000
1	-1.620221000	-0.168590000	-0.445152000
1	-2.577834000	-0.686922000	-1.826454000
1	-3.005835000	0.803227000	-0.972268000
6	-4.360256000	-3.102238000	-2.479789000
1	-4.816373000	-3.975992000	-1.996947000
1	-4.229742000	-3.360471000	-3.539927000
1	-3.364985000	-2.964536000	-2.043517000
6	-6.620654000	-2.102390000	-2.960398000
1	-6.518217000	-2.439304000	-4.000814000
1	-7.172058000	-2.884268000	-2.423498000
1	-7.243924000	-1.201820000	-2.971384000
6	-5.871979000	-3.456140000	1.017879000
1	-6.571812000	-4.010631000	1.656838000
1	-5.602296000	-4.114317000	0.184736000
1	-4.964502000	-3.275448000	1.608363000
6	-7.052358000	-1.346714000	1.722985000
1	-7.571633000	-0.435706000	1.413299000
1	-7.765912000	-1.955074000	2.293940000
1	-6.256313000	-1.048300000	2.413004000

E$_{1d}$

E$_1$ = -5417.02193566 A.U.

At No.	X	Y	Z
6	-2.386950000	1.792376000	4.120359000
6	-1.237716000	1.010539000	4.275187000
6	-3.629777000	1.163272000	4.124487000
6	-1.345072000	-0.374777000	4.384479000
6	-3.737463000	-0.218951000	4.254162000
6	-2.586663000	-1.006700000	4.362124000
1	-4.510519000	1.784955000	4.006922000
1	-0.459783000	-0.994488000	4.487263000
6	-2.280139000	3.286529000	3.902505000
1	-3.213536000	3.762056000	4.215302000
1	-1.474853000	3.693634000	4.519473000
6	-2.008539000	3.617027000	2.449266000
6	-0.713171000	3.827856000	1.982750000
6	-3.052109000	3.698133000	1.521378000
6	-0.452082000	4.080115000	0.638127000
6	-2.795671000	4.023402000	0.196814000
6	-1.498272000	4.192194000	-0.278733000
1	0.112569000	3.783279000	2.684635000
1	-3.615126000	4.132177000	-0.503164000
6	-1.253912000	4.441058000	-1.752747000
1	-0.231239000	4.798743000	-1.892188000

1	-1.933405000	5.221847000	-2.106641000
6	-1.463537000	3.178982000	-2.564817000
6	-0.396490000	2.316727000	-2.812837000
6	-2.730958000	2.816972000	-3.030736000
6	-0.571842000	1.115578000	-3.497637000
6	-2.905042000	1.623817000	-3.726685000
6	-1.841909000	0.759448000	-3.965735000
1	0.583783000	2.607464000	-2.449801000
1	-3.883814000	1.323164000	-4.081980000
6	-2.080900000	-0.551714000	-4.683374000
1	-1.207571000	-0.801020000	-5.292401000
1	-2.933253000	-0.436989000	-5.358863000
6	-2.349390000	-1.688198000	-3.719240000
6	-3.638832000	-1.960885000	-3.249982000
6	-1.293862000	-2.465329000	-3.248982000
6	-3.847203000	-3.027400000	-2.377265000
6	-1.497170000	-3.497225000	-2.335772000
6	-2.792565000	-3.809069000	-1.914266000
1	-0.299204000	-2.221461000	-3.602990000
1	-4.836108000	-3.263352000	-2.002892000
6	-3.056112000	-4.907305000	-0.904762000
1	-4.036115000	-5.350559000	-1.096733000
1	-2.305188000	-5.694724000	-1.009698000
6	-3.001658000	-4.347688000	0.499889000
6	-4.148310000	-3.879649000	1.158643000
6	-1.778935000	-4.184009000	1.129074000
6	-4.051126000	-3.331719000	2.434774000
6	-1.668837000	-3.543136000	2.358731000
6	-2.808049000	-3.145915000	3.051916000
1	-0.883726000	-4.526601000	0.620756000
1	-4.933968000	-2.998809000	2.967294000
6	-2.695846000	-2.514628000	4.425637000
1	-3.571173000	-2.792384000	5.019368000
1	-1.811728000	-2.915096000	4.927849000
8	0.443100000	0.239116000	-3.768347000
6	1.701497000	0.441454000	-3.160844000
1	1.597384000	0.424712000	-2.064166000
1	2.116868000	1.421287000	-3.441469000
8	0.839820000	4.268506000	0.200267000
6	1.665219000	3.115591000	0.225426000
1	1.128932000	2.259058000	-0.209717000
1	1.924633000	2.847910000	1.260882000
8	-0.053682000	1.691505000	4.338845000
6	1.151994000	1.020798000	4.034169000
1	1.363337000	0.230259000	4.769066000
1	1.073190000	0.547418000	3.043483000
8	-0.416591000	-3.298342000	2.882672000
6	0.352434000	-2.418583000	2.071964000
1	0.294434000	-2.728221000	1.019768000
1	-0.065071000	-1.402502000	2.131292000
8	-0.477116000	-4.245257000	-1.809201000
6	0.841255000	-3.776767000	-1.992835000
1	1.139609000	-3.876254000	-3.048273000
1	0.899953000	-2.708834000	-1.730333000
8	-4.648584000	-1.145935000	-3.691750000
6	-5.925532000	-1.349012000	-3.126629000
1	-6.285219000	-2.362520000	-3.361896000
1	-5.868592000	-1.264545000	-2.030184000
8	-5.324456000	-4.007437000	0.471995000
6	-6.476545000	-3.359521000	0.965700000
1	-6.793724000	-3.813569000	1.917194000
1	-6.261781000	-2.296439000	1.158962000

8	-4.929255000	-0.888558000	4.305635000
6	-6.107949000	-0.206423000	3.941754000
1	-6.267985000	0.670928000	4.585510000
1	-6.026793000	0.151469000	2.904185000
8	-4.345177000	3.501806000	1.951314000
6	-5.086398000	2.522772000	1.235950000
1	-4.927979000	2.628572000	0.155297000
1	-4.734796000	1.519573000	1.518974000
8	-3.759259000	3.684111000	-2.769955000
6	-5.078770000	3.206966000	-2.928996000
1	-5.308617000	3.056584000	-3.994822000
1	-5.185862000	2.233542000	-2.425456000
6	1.774382000	-4.582434000	-1.107421000
1	1.529522000	-4.388322000	-0.054696000
1	1.601071000	-5.650912000	-1.280491000
6	3.238389000	-4.235863000	-1.376298000
1	3.508474000	-4.561820000	-2.390629000
1	3.367997000	-3.142962000	-1.364194000
6	4.199336000	-4.858542000	-0.366432000
1	3.954343000	-4.483292000	0.637022000
1	4.042039000	-5.945579000	-0.332001000
6	5.667048000	-4.567146000	-0.676456000
1	5.920335000	-4.999487000	-1.653666000
1	5.808775000	-3.483411000	-0.777761000
6	6.622344000	-5.105824000	0.385345000
1	6.399309000	-4.674541000	1.368555000
1	6.539768000	-6.194856000	0.476169000
1	7.663142000	-4.866237000	0.142713000
6	2.626657000	-0.664901000	-3.639335000
1	2.184635000	-1.635833000	-3.384224000
1	2.679240000	-0.620984000	-4.733977000
6	4.029674000	-0.562700000	-3.043291000
1	4.444317000	0.435914000	-3.243537000
1	3.970801000	-0.654985000	-1.949583000
6	4.983662000	-1.623994000	-3.591473000
1	4.583506000	-2.622009000	-3.367336000
1	5.015403000	-1.547483000	-4.687473000
6	6.406763000	-1.519163000	-3.044016000
1	6.817050000	-0.530185000	-3.286416000
1	6.385501000	-1.579769000	-1.947056000
6	7.328815000	-2.603797000	-3.594061000
1	6.963494000	-3.601303000	-3.324976000
1	7.385287000	-2.553637000	-4.687622000
1	8.343943000	-2.500933000	-3.200000000
6	2.920464000	3.436724000	-0.565428000
1	2.645576000	3.622050000	-1.612616000
1	3.333456000	4.377720000	-0.180901000
6	3.975506000	2.338418000	-0.479603000
1	4.254463000	2.192601000	0.571524000
1	3.552885000	1.382167000	-0.818581000
6	5.230362000	2.653193000	-1.292989000
1	4.968479000	2.694736000	-2.360411000
1	5.592387000	3.657575000	-1.030888000
6	6.355048000	1.641508000	-1.083601000
1	6.660469000	1.651049000	-0.029337000
1	5.974213000	0.633128000	-1.281373000
6	7.571546000	1.906800000	-1.963573000
1	7.303723000	1.892020000	-3.026813000
1	8.011663000	2.887069000	-1.746346000
1	8.345157000	1.147831000	-1.805611000
6	2.252818000	2.069400000	4.047637000
1	1.924692000	2.905419000	3.419239000

1	2.353733000	2.470387000	5.063050000
6	3.602215000	1.565182000	3.537821000
1	4.034790000	0.844057000	4.244803000
1	3.457255000	1.021741000	2.592863000
6	4.579480000	2.715189000	3.290391000
1	4.131231000	3.392169000	2.547891000
1	4.694489000	3.310731000	4.206236000
6	5.956001000	2.271162000	2.790929000
1	6.516118000	1.800350000	3.609249000
1	5.831397000	1.493023000	2.027101000
6	6.757234000	3.430525000	2.201898000
1	6.213981000	3.886680000	1.365170000
1	6.930607000	4.212861000	2.949579000
1	7.730872000	3.101671000	1.825063000
6	1.796495000	-2.454999000	2.527910000
1	1.888565000	-2.017726000	3.530770000
1	2.103979000	-3.505261000	2.610308000
6	2.706185000	-1.728571000	1.541266000
1	2.577126000	-2.173635000	0.544140000
1	2.393742000	-0.678291000	1.442201000
6	4.181758000	-1.787637000	1.921131000
1	4.335685000	-1.294603000	2.890884000
1	4.482123000	-2.835655000	2.059843000
6	5.077415000	-1.138313000	0.872647000
1	4.874686000	-1.596306000	-0.104980000
1	4.803094000	-0.080939000	0.770958000
6	6.560829000	-1.258648000	1.193539000
1	6.779831000	-0.840386000	2.182836000
1	6.886351000	-2.304815000	1.195350000
1	7.170076000	-0.721524000	0.459656000
6	-7.271445000	-1.170113000	4.090433000
1	-7.042099000	-2.093591000	3.545243000
1	-7.371664000	-1.444715000	5.147443000
6	-8.576913000	-0.578171000	3.566175000
1	-8.746590000	0.410402000	4.017064000
1	-8.486815000	-0.408862000	2.484318000
6	-9.789860000	-1.466702000	3.831499000
1	-9.589375000	-2.478231000	3.450832000
1	-9.937378000	-1.571452000	4.915332000
6	-11.065579000	-0.931354000	3.185397000
1	-11.251562000	0.091297000	3.541724000
1	-10.907217000	-0.854926000	2.102559000
6	-12.289210000	-1.801450000	3.459299000
1	-12.123933000	-2.829251000	3.115077000
1	-12.511583000	-1.844428000	4.531597000
1	-13.175911000	-1.415545000	2.945925000
6	-6.558184000	2.712310000	1.551512000
1	-6.732091000	2.548500000	2.623268000
1	-6.816457000	3.759642000	1.348602000
6	-7.452832000	1.792167000	0.723326000
1	-7.217774000	1.924178000	-0.342309000
1	-7.225629000	0.740050000	0.951151000
6	-8.941874000	2.054642000	0.941515000
1	-9.191688000	1.908639000	2.001454000
1	-9.158645000	3.108740000	0.718273000
6	-9.836821000	1.167716000	0.081086000
1	-9.545221000	1.281913000	-0.970878000
1	-9.649927000	0.114743000	0.330972000
6	-11.322500000	1.477470000	0.239309000
1	-11.642562000	1.342225000	1.278452000
1	-11.538932000	2.514079000	-0.043606000
1	-11.938762000	0.825523000	-0.389495000

6	-6.033662000	4.219032000	-2.322847000
1	-5.841930000	4.294163000	-1.244230000
1	-5.827124000	5.207521000	-2.749322000
6	-7.492283000	3.833193000	-2.560895000
1	-7.714941000	3.869372000	-3.636337000
1	-7.648880000	2.788584000	-2.255104000
6	-8.475266000	4.720209000	-1.800577000
1	-8.243230000	4.671024000	-0.726903000
1	-8.330608000	5.768677000	-2.095825000
6	-9.932680000	4.320540000	-2.022369000
1	-10.177123000	4.410841000	-3.089155000
1	-10.050852000	3.258622000	-1.772628000
6	-10.914332000	5.145648000	-1.194445000
1	-10.713216000	5.033007000	-0.122254000
1	-10.836949000	6.211861000	-1.435691000
1	-11.948689000	4.834616000	-1.375522000
6	-6.902051000	-0.326456000	-3.677851000
1	-6.588475000	0.682290000	-3.381628000
1	-6.879749000	-0.361501000	-4.773601000
6	-8.313862000	-0.616797000	-3.168403000
1	-8.606006000	-1.627045000	-3.489904000
1	-8.309803000	-0.637510000	-2.067801000
6	-9.374207000	0.376641000	-3.637962000
1	-9.154566000	1.371810000	-3.227226000
1	-9.327785000	0.483111000	-4.730508000
6	-10.783775000	-0.050637000	-3.226661000
1	-11.047716000	-0.969682000	-3.767064000
1	-10.782289000	-0.316868000	-2.161356000
6	-11.841340000	1.020027000	-3.479212000
1	-11.629945000	1.923205000	-2.895128000
1	-11.869705000	1.306747000	-4.536704000
1	-12.839309000	0.666761000	-3.199168000
6	-7.570763000	-3.511997000	-0.077655000
1	-7.239152000	-3.042058000	-1.012664000
1	-7.699553000	-4.580326000	-0.289649000
6	-8.900697000	-2.904797000	0.365253000
1	-9.204446000	-3.345504000	1.325354000
1	-8.769367000	-1.829236000	0.551202000
6	-10.018257000	-3.116029000	-0.656872000
1	-9.726423000	-2.667195000	-1.616113000
1	-10.132113000	-4.193043000	-0.844461000
6	-11.362552000	-2.537432000	-0.218010000
1	-11.631117000	-2.948557000	0.764058000
1	-11.261519000	-1.453797000	-0.076282000
6	-12.487379000	-2.813921000	-1.212631000
1	-12.254792000	-2.391072000	-2.196309000
1	-12.641962000	-3.891249000	-1.341843000
1	-13.432797000	-2.377282000	-0.874541000
6	17.420840000	-0.273349000	0.080805000
1	17.294740000	-0.642567000	-0.947187000
6	18.697251000	0.511984000	0.177105000
1	18.765509000	1.387522000	-0.470374000
1	17.478724000	-1.157318000	0.728327000
6	19.710114000	0.228518000	0.990933000
1	19.681606000	-0.632969000	1.654980000
1	20.606398000	0.841153000	1.023710000
6	13.666467000	0.601766000	0.709615000
1	13.753111000	0.915700000	1.759290000
1	13.626916000	1.521521000	0.104847000
6	14.883901000	-0.221294000	0.316445000
1	14.909971000	-1.122086000	0.942208000
1	14.757702000	-0.561119000	-0.719311000

6	16.188875000	0.560114000	0.457284000
1	16.150628000	1.456673000	-0.178083000
1	16.303035000	0.918129000	1.489025000
8	12.493778000	-0.167528000	0.552376000
14	11.062350000	0.339474000	-0.151003000
6	11.359377000	1.094234000	-1.884539000
1	10.492414000	0.734233000	-2.461601000
6	10.181704000	1.596004000	0.979089000
1	9.380636000	2.046662000	0.370963000
6	10.080414000	-1.272235000	-0.371168000
1	9.046470000	-0.979621000	-0.615375000
6	10.613199000	-2.110455000	-1.545345000
1	11.659791000	-2.393049000	-1.380995000
1	10.037639000	-3.040163000	-1.642699000
1	10.551865000	-1.584146000	-2.503834000
6	10.049866000	-2.152578000	0.888197000
1	9.576173000	-1.661189000	1.740672000
1	9.492065000	-3.078211000	0.692698000
1	11.066561000	-2.430724000	1.186013000
6	12.618736000	0.545690000	-2.577338000
1	13.526886000	0.954402000	-2.119555000
1	12.690585000	-0.544406000	-2.527435000
1	12.633546000	0.834960000	-3.635936000
6	11.344344000	2.626014000	-1.985797000
1	12.230687000	3.066426000	-1.513389000
1	11.352259000	2.941574000	-3.036959000
1	10.460420000	3.068669000	-1.515534000
6	11.116894000	2.727086000	1.442224000
1	10.550022000	3.491644000	1.989088000
1	11.879654000	2.339794000	2.127304000
1	11.632983000	3.227969000	0.618532000
6	9.532895000	0.955919000	2.215315000
1	8.694538000	0.308461000	1.947697000
1	10.258130000	0.359748000	2.781646000
1	9.147501000	1.726688000	2.895337000

E_{1a}’

E_1 = -5417.05153300 A.U.

At No.	X	Y	Z
6	1.438254000	-3.308173000	2.460846000
6	2.637926000	-3.523901000	1.773839000
6	0.235782000	-3.599137000	1.818769000
6	2.616041000	-4.030920000	0.477473000
6	0.216704000	-4.085558000	0.514113000
6	1.415476000	-4.318411000	-0.165069000
1	-0.685138000	-3.406401000	2.358359000
1	3.531632000	-4.191019000	-0.080048000
6	1.434065000	-2.704427000	3.848502000
1	0.547952000	-3.047029000	4.389153000
1	2.314320000	-3.043962000	4.401082000
6	1.436890000	-1.191538000	3.788325000
6	2.641181000	-0.496116000	3.706150000
6	0.243725000	-0.465161000	3.761616000
6	2.676085000	0.892894000	3.611915000
6	0.278488000	0.926182000	3.692510000
6	1.482414000	1.621053000	3.622966000
1	3.551346000	-1.082727000	3.696450000
1	-0.633591000	1.508783000	3.656059000
6	1.481349000	3.130796000	3.524077000
1	2.349110000	3.531543000	4.055547000
1	0.584375000	3.518136000	4.015478000
6	1.517770000	3.614515000	2.090967000

6	2.735691000	3.890918000	1.479977000
6	0.342872000	3.770075000	1.345672000
6	2.805361000	4.324002000	0.157941000
6	0.409594000	4.237047000	0.035345000
6	1.631673000	4.523446000	-0.572818000
1	3.632554000	3.735843000	2.067345000
1	-0.488685000	4.366737000	-0.558073000
6	1.674485000	4.934743000	-2.029565000
1	2.579206000	5.515545000	-2.225310000
1	0.812940000	5.564876000	-2.259729000
6	1.661898000	3.698102000	-2.901797000
6	0.474006000	3.181272000	-3.417843000
6	2.838240000	2.984146000	-3.101119000
6	0.482282000	1.976815000	-4.118815000
6	2.849271000	1.772551000	-3.785819000
6	1.659132000	1.254632000	-4.308922000
1	3.739965000	3.392347000	-2.662839000
1	-0.439717000	1.558402000	-4.508149000
6	1.644701000	-0.065338000	-5.049839000
1	0.782619000	-0.079968000	-5.723546000
1	2.543686000	-0.141015000	-5.668634000
6	1.580330000	-1.275409000	-4.140500000
6	0.355526000	-1.738449000	-3.642558000
6	2.740548000	-1.965534000	-3.795922000
6	0.310902000	-2.892267000	-2.863908000
6	2.695849000	-3.101978000	-2.991235000
6	1.471280000	-3.585908000	-2.531320000
1	3.684450000	-1.585207000	-4.170472000
1	-0.624389000	-3.268137000	-2.467146000
6	1.409219000	-4.781455000	-1.605618000
1	0.500606000	-5.354027000	-1.805274000
1	2.265825000	-5.434649000	-1.785180000
8	3.992007000	4.572428000	-0.484993000
6	5.168984000	4.098993000	0.132390000
1	5.057509000	3.030470000	0.373945000
1	5.351409000	4.636006000	1.075871000
8	3.839005000	1.613214000	3.519824000
6	5.031874000	0.893928000	3.298138000
1	4.942553000	0.293714000	2.378569000
1	5.218173000	0.198343000	4.130311000
8	3.793316000	-3.218504000	2.445645000
6	5.006539000	-3.219575000	1.725519000
1	5.207668000	-4.219636000	1.312811000
1	4.946599000	-2.516514000	0.878982000
8	3.806449000	-3.818991000	-2.624491000
6	5.051414000	-3.160852000	-2.616633000
1	5.364214000	-2.903206000	-3.639969000
1	4.975234000	-2.222691000	-2.044059000
8	3.997838000	1.042813000	-3.973187000
6	5.027661000	1.292966000	-3.040464000
1	5.384172000	2.327883000	-3.131455000
1	4.627607000	1.171895000	-2.021749000
8	-0.660859000	3.917941000	-3.197116000
6	-1.886983000	3.225807000	-3.115713000
1	-2.216738000	2.886899000	-4.109533000
1	-1.759972000	2.331733000	-2.487914000
8	-0.757124000	-1.014777000	-3.974047000
6	-1.993355000	-1.445543000	-3.443726000
1	-2.242579000	-2.444699000	-3.834489000
1	-1.924718000	-1.518720000	-2.349291000
8	-0.924492000	-4.356924000	-0.185611000
6	-2.179461000	-4.082173000	0.396399000

1	-2.359846000	-4.751044000	1.252014000
1	-2.211335000	-3.047618000	0.765491000
8	-0.921517000	-1.187872000	3.797411000
6	-2.129561000	-0.458241000	3.800338000
1	-2.151439000	0.234078000	4.655127000
1	-2.202306000	0.144098000	2.883302000
8	-0.821593000	3.447150000	1.982040000
6	-2.049310000	3.644416000	1.313141000
1	-2.176736000	4.709936000	1.064912000
1	-2.074150000	3.070258000	0.375929000
6	6.217310000	0.372807000	-3.254225000
1	5.876321000	-0.646457000	-3.458502000
1	6.787454000	0.701676000	-4.131252000
6	7.087799000	0.407916000	-1.992841000
1	7.050693000	1.419066000	-1.560969000
1	6.640988000	-0.251111000	-1.233590000
6	8.556145000	0.043087000	-2.189218000
1	8.648883000	-0.920702000	-2.706330000
1	9.029137000	0.791554000	-2.841102000
6	9.294470000	-0.014560000	-0.853248000
1	9.055648000	0.893479000	-0.283406000
1	8.895556000	-0.850928000	-0.262805000
6	10.807803000	-0.150945000	-0.984200000
1	11.075417000	-1.046378000	-1.555791000
1	11.235559000	0.715639000	-1.500199000
1	11.289665000	-0.224867000	-0.002607000
6	6.341587000	4.304491000	-0.807379000
1	6.170828000	3.731850000	-1.726928000
1	6.391518000	5.360686000	-1.096986000
6	7.656194000	3.874106000	-0.156876000
1	7.855607000	4.515925000	0.712961000
1	7.555329000	2.851459000	0.237114000
6	8.848515000	3.926690000	-1.110231000
1	8.699605000	3.194565000	-1.917603000
1	8.885052000	4.912477000	-1.594684000
6	10.185086000	3.655503000	-0.420676000
1	10.336412000	4.397187000	0.375473000
1	10.147909000	2.678308000	0.077965000
6	11.368149000	3.690155000	-1.384845000
1	11.233071000	2.965385000	-2.196440000
1	11.474649000	4.680291000	-1.842435000
1	12.306756000	3.451556000	-0.874262000
6	6.194438000	1.865443000	3.195424000
1	5.986898000	2.610006000	2.418304000
1	6.283396000	2.410641000	4.142942000
6	7.497342000	1.125688000	2.887781000
1	7.591276000	0.269585000	3.571242000
1	7.449922000	0.699617000	1.874209000
6	8.750389000	1.990346000	3.018795000
1	8.694345000	2.837832000	2.322621000
1	8.786473000	2.423480000	4.028189000
6	10.033413000	1.201537000	2.761760000
1	10.067820000	0.345323000	3.448370000
1	9.997600000	0.776131000	1.750924000
6	11.302547000	2.034427000	2.920431000
1	11.306507000	2.882582000	2.226588000
1	11.384896000	2.436019000	3.936899000
1	12.197296000	1.434843000	2.722742000
6	6.124565000	-2.833976000	2.679024000
1	5.881195000	-1.876299000	3.154684000
1	6.168790000	-3.581969000	3.480026000
6	7.478979000	-2.736220000	1.975778000

1	7.626688000	-3.618718000	1.336500000
1	7.480355000	-1.866892000	1.302272000
6	8.652824000	-2.631702000	2.949662000
1	8.515288000	-1.756962000	3.600272000
1	8.646839000	-3.508449000	3.612461000
6	10.008540000	-2.536461000	2.252486000
1	10.126384000	-3.396676000	1.581159000
1	10.024763000	-1.643034000	1.614865000
6	11.185132000	-2.492125000	3.224294000
1	11.107162000	-1.636264000	3.904521000
1	11.221629000	-3.399485000	3.838032000
1	12.137524000	-2.409089000	2.690613000
6	6.072659000	-4.097470000	-1.996385000
1	5.735742000	-4.385442000	-0.992686000
1	6.109421000	-5.017205000	-2.592746000
6	7.457602000	-3.461727000	-1.918898000
1	7.733069000	-3.066397000	-2.907164000
1	7.425668000	-2.597357000	-1.240069000
6	8.544077000	-4.428095000	-1.452608000
1	8.268420000	-4.847597000	-0.474260000
1	8.595857000	-5.279932000	-2.144912000
6	9.916141000	-3.765698000	-1.350443000
1	10.184685000	-3.339463000	-2.326917000
1	9.850645000	-2.919704000	-0.655318000
6	11.014099000	-4.720070000	-0.888377000
1	10.773563000	-5.152240000	0.090341000
1	11.138270000	-5.549947000	-1.593338000
1	11.976884000	-4.206356000	-0.799030000
6	-3.215842000	-4.295104000	-0.692569000
1	-3.045236000	-3.551022000	-1.482935000
1	-3.037905000	-5.276543000	-1.148568000
6	-4.659825000	-4.209049000	-0.203634000
1	-4.854911000	-5.018923000	0.512671000
1	-4.818818000	-3.270848000	0.343483000
6	-5.646853000	-4.308659000	-1.367077000
1	-5.458490000	-3.486093000	-2.073147000
1	-5.438598000	-5.233926000	-1.923628000
6	-7.120139000	-4.291780000	-0.962085000
1	-7.293417000	-5.042846000	-0.179522000
1	-7.376942000	-3.321262000	-0.517906000
6	-8.036774000	-4.576322000	-2.150607000
1	-7.893882000	-3.832583000	-2.943978000
1	-7.826412000	-5.562924000	-2.579824000
1	-9.092293000	-4.554976000	-1.861396000
6	-3.302914000	-1.415441000	3.887491000
1	-3.245400000	-2.145130000	3.068640000
1	-3.243215000	-1.982702000	4.824066000
6	-4.614420000	-0.634637000	3.814724000
1	-4.598714000	0.166288000	4.567968000
1	-4.675302000	-0.127338000	2.841772000
6	-5.870867000	-1.475650000	4.021203000
1	-5.918593000	-2.273116000	3.265703000
1	-5.815637000	-1.978726000	4.996677000
6	-7.142901000	-0.630617000	3.955007000
1	-7.046322000	0.211131000	4.654311000
1	-7.232227000	-0.192923000	2.952122000
6	-8.409711000	-1.416301000	4.274860000
1	-8.536397000	-2.250893000	3.577403000
1	-8.374148000	-1.827964000	5.290021000
1	-9.298128000	-0.778727000	4.202801000
6	-3.145991000	3.172222000	2.251678000
1	-3.079621000	2.080752000	2.344584000

1	-2.944700000	3.589425000	3.246106000
6	-4.550437000	3.575415000	1.807227000
1	-4.605801000	4.669830000	1.719391000
1	-4.759682000	3.177276000	0.804428000
6	-5.620811000	3.095673000	2.787800000
1	-5.607675000	1.997591000	2.839546000
1	-5.359945000	3.447372000	3.796249000
6	-7.033484000	3.564663000	2.445539000
1	-7.032470000	4.654993000	2.312680000
1	-7.335893000	3.140495000	1.479141000
6	-8.049955000	3.184324000	3.519871000
1	-8.069474000	2.100150000	3.675106000
1	-7.796425000	3.650184000	4.479108000
1	-9.062388000	3.502452000	3.250474000
6	-2.915082000	4.170778000	-2.520168000
1	-2.539367000	4.546367000	-1.560072000
1	-3.016656000	5.041708000	-3.179069000
6	-4.271495000	3.502540000	-2.315240000
1	-4.618437000	3.069818000	-3.264131000
1	-4.159445000	2.662076000	-1.615385000
6	-5.330283000	4.464645000	-1.782282000
1	-4.979285000	4.906824000	-0.839494000
1	-5.445049000	5.299715000	-2.487333000
6	-6.693006000	3.811104000	-1.556859000
1	-7.031119000	3.352254000	-2.495761000
1	-6.589170000	2.988901000	-0.832702000
6	-7.739733000	4.807816000	-1.064580000
1	-7.446432000	5.236782000	-0.099839000
1	-7.853528000	5.633598000	-1.776195000
1	-8.719656000	4.338281000	-0.937810000
6	-3.068944000	-0.447591000	-3.834474000
1	-2.904022000	0.491570000	-3.291805000
1	-2.969771000	-0.220026000	-4.902987000
6	-4.472882000	-0.982058000	-3.547944000
1	-4.657117000	-1.867089000	-4.173045000
1	-4.535080000	-1.328446000	-2.507400000
6	-5.569001000	0.051222000	-3.795604000
1	-5.445803000	0.888815000	-3.094885000
1	-5.444670000	0.477625000	-4.801478000
6	-6.984767000	-0.508592000	-3.664303000
1	-7.118897000	-1.327662000	-4.383353000
1	-7.116238000	-0.953436000	-2.667884000
6	-8.049316000	0.560509000	-3.899190000
1	-8.006447000	1.334297000	-3.122805000
1	-7.899518000	1.054701000	-4.866222000
1	-9.057025000	0.135734000	-3.893776000
6	-3.403917000	-0.577445000	0.082281000
1	-3.448329000	-1.398834000	-0.647406000
6	-2.201421000	0.276742000	-0.207078000
1	-2.342552000	1.029434000	-0.984025000
1	-3.297425000	-1.054290000	1.064497000
6	-1.007308000	0.163805000	0.370815000
1	-0.806278000	-0.584667000	1.135203000
1	-0.178132000	0.807489000	0.091714000
6	-7.265730000	0.003388000	0.169186000
1	-7.255804000	0.812251000	0.918528000
1	-7.422526000	0.475632000	-0.814769000
6	-5.934845000	-0.726720000	0.189088000
1	-5.865399000	-1.272154000	1.138851000
1	-5.943143000	-1.483993000	-0.604770000
6	-4.727559000	0.186883000	0.017688000
1	-4.797083000	0.703802000	-0.948888000

1	-4.735183000	0.969568000	0.787700000
8	-8.287759000	-0.923201000	0.455302000
14	-9.937637000	-0.595390000	0.477752000
6	-10.510306000	-0.442984000	-1.320708000
1	-9.867400000	0.349897000	-1.733860000
6	-10.182911000	1.041253000	1.407662000
1	-9.403655000	1.022052000	2.185396000
6	-10.683489000	-2.055883000	1.424593000
1	-10.582932000	-1.781694000	2.484380000
6	-11.534537000	1.172166000	2.123276000
1	-12.369827000	1.181809000	1.412341000
1	-11.707507000	0.350380000	2.826794000
1	-11.582786000	2.109553000	2.692492000
6	-9.920335000	2.265208000	0.517453000
1	-9.917994000	3.190876000	1.106597000
1	-8.955703000	2.206026000	0.001503000
1	-10.692568000	2.373569000	-0.252768000
6	-10.219222000	-1.727236000	-2.107216000
1	-9.173360000	-2.031674000	-2.003689000
1	-10.842430000	-2.558725000	-1.756018000
1	-10.429126000	-1.598737000	-3.177325000
6	-11.966242000	0.004569000	-1.518184000
1	-12.672373000	-0.760616000	-1.179685000
1	-12.199170000	0.928151000	-0.977576000
1	-12.171644000	0.186834000	-2.580934000
6	-12.179275000	-2.272525000	1.148517000
1	-12.345086000	-2.626926000	0.124558000
1	-12.588543000	-3.034685000	1.823485000
1	-12.770751000	-1.360145000	1.284273000
6	-9.903388000	-3.362483000	1.216753000
1	-8.842395000	-3.242503000	1.452602000
1	-10.311035000	-4.160259000	1.851054000
1	-9.967189000	-3.704918000	0.177329000

E_{1b}’

E_1 = -5417.03919708 A.U.

At No.	X	Y	Z
6	1.880836000	4.492608000	-0.436857000
6	3.107552000	4.306763000	0.205415000
6	0.710623000	4.262615000	0.282437000
6	3.138839000	3.930499000	1.546334000
6	0.742317000	3.859707000	1.615356000
6	1.969971000	3.704256000	2.266921000
1	-0.227241000	4.377761000	-0.248065000
1	4.078137000	3.774943000	2.063110000
6	1.801799000	4.845686000	-1.906836000
1	0.916294000	5.460403000	-2.086311000
1	2.678819000	5.430623000	-2.195708000
6	1.736718000	3.588997000	-2.747971000
6	2.910081000	2.926473000	-3.088785000
6	0.518356000	3.014894000	-3.118287000
6	2.896879000	1.716241000	-3.778940000
6	0.503005000	1.830551000	-3.851445000
6	1.680283000	1.163440000	-4.185679000
1	3.839348000	3.373850000	-2.758972000
1	-0.430994000	1.368317000	-4.147528000
6	1.618462000	-0.153539000	-4.928840000
1	2.484706000	-0.240436000	-5.590562000
1	0.722649000	-0.163266000	-5.556979000
6	1.590052000	-1.350866000	-4.001764000
6	2.775766000	-2.002438000	-3.666469000
6	0.389192000	-1.825858000	-3.459423000

6	2.781290000	-3.112095000	-2.825268000
6	0.396198000	-2.951719000	-2.638703000
6	1.580853000	-3.603488000	-2.310581000
1	3.698630000	-1.606968000	-4.075872000
1	-0.517241000	-3.334513000	-2.200868000
6	1.572312000	-4.758742000	-1.333981000
1	2.409759000	-5.428503000	-1.542730000
1	0.647834000	-5.329665000	-1.450266000
6	1.679343000	-4.243661000	0.085225000
6	0.533254000	-3.933249000	0.822664000
6	2.925715000	-3.995735000	0.653129000
6	0.649196000	-3.436776000	2.117983000
6	3.044331000	-3.459493000	1.932810000
6	1.897652000	-3.192996000	2.687680000
1	3.800303000	-4.211692000	0.051030000
1	-0.228104000	-3.192424000	2.706483000
6	1.998265000	-2.583268000	4.069176000
1	1.152689000	-2.920177000	4.674461000
1	2.914416000	-2.926369000	4.556896000
6	2.004659000	-1.070802000	4.003938000
6	0.813393000	-0.341344000	4.041668000
6	3.203653000	-0.380474000	3.848804000
6	0.847025000	1.049180000	3.967105000
6	3.236407000	1.008475000	3.745582000
6	2.047634000	1.740537000	3.824224000
1	4.110114000	-0.970255000	3.787651000
1	-0.064753000	1.634142000	3.989130000
6	2.043683000	3.249928000	3.708552000
1	1.188784000	3.648052000	4.262094000
1	2.951284000	3.654707000	4.164860000
8	3.914868000	-3.794020000	-2.467493000
6	5.159357000	-3.152469000	-2.619156000
1	5.147498000	-2.182682000	-2.096454000
1	5.370366000	-2.956708000	-3.681407000
8	4.048314000	1.026713000	-4.078218000
6	5.133811000	1.277198000	-3.210167000
1	4.797321000	1.154213000	-2.169091000
1	5.481540000	2.313383000	-3.321115000
8	4.239280000	4.510792000	-0.542592000
6	5.463141000	4.076673000	0.007740000
1	5.703261000	4.652940000	0.914439000
1	5.385956000	3.017086000	0.297600000
8	4.395389000	1.722792000	3.577756000
6	5.560755000	0.996620000	3.255089000
1	5.811665000	0.296477000	4.066256000
1	5.390226000	0.400521000	2.344385000
8	4.249099000	-3.174836000	2.522006000
6	5.403174000	-3.182670000	1.710332000
1	5.582353000	-4.190898000	1.307186000
1	5.269023000	-2.501701000	0.854511000
8	-0.661215000	-4.141754000	0.188819000
6	-1.860508000	-3.863082000	0.874454000
1	-1.957937000	-4.520533000	1.752338000
1	-1.863258000	-2.823292000	1.236064000
8	-0.343995000	-1.063827000	4.160028000
6	-1.564869000	-0.367440000	4.045032000
1	-1.662348000	0.371457000	4.855220000
1	-1.600337000	0.176572000	3.088727000
8	-0.383399000	3.602604000	2.351205000
6	-1.621657000	3.644435000	1.677170000
1	-1.792589000	4.648971000	1.260405000
1	-1.617454000	2.931229000	0.838589000

8	-0.618128000	3.667369000	-2.717568000
6	-1.836674000	2.971680000	-2.851049000
1	-2.085429000	2.832906000	-3.914345000
1	-1.740864000	1.970904000	-2.404481000
8	-0.755047000	-1.142070000	-3.777339000
6	-1.965068000	-1.619542000	-3.224892000
1	-2.164880000	-2.639493000	-3.588420000
1	-1.889864000	-1.665467000	-2.128965000
6	6.590300000	-2.763567000	2.560311000
1	6.377212000	-1.795484000	3.029414000
1	6.708030000	-3.490133000	3.373531000
6	7.879956000	-2.675623000	1.742470000
1	7.970426000	-3.565772000	1.103049000
1	7.824384000	-1.814297000	1.060924000
6	9.135024000	-2.564517000	2.608115000
1	9.058545000	-1.682127000	3.258358000
1	9.184959000	-3.433975000	3.278608000
6	10.423329000	-2.483381000	1.791506000
1	10.473715000	-3.348608000	1.117935000
1	10.388354000	-1.594022000	1.149083000
6	11.683392000	-2.441747000	2.652413000
1	11.673968000	-1.580194000	3.329886000
1	11.768564000	-3.344820000	3.267640000
1	12.583982000	-2.370553000	2.033795000
6	6.229631000	-4.067123000	-2.050875000
1	5.985230000	-4.304528000	-1.008119000
1	6.208026000	-5.014004000	-2.603935000
6	7.618371000	-3.439429000	-2.130139000
1	7.798333000	-3.078744000	-3.153135000
1	7.655586000	-2.553153000	-1.480889000
6	8.741427000	-4.397521000	-1.739107000
1	8.559280000	-4.784834000	-0.726222000
1	8.724406000	-5.270654000	-2.406178000
6	10.119196000	-3.740753000	-1.788427000
1	10.293964000	-3.343217000	-2.797770000
1	10.123601000	-2.875735000	-1.114173000
6	11.253510000	-4.688225000	-1.406895000
1	11.109493000	-5.087510000	-0.395790000
1	11.303955000	-5.540701000	-2.093807000
1	12.222485000	-4.178927000	-1.429678000
6	6.310726000	0.359249000	-3.494657000
1	5.961194000	-0.662470000	-3.673502000
1	6.825315000	0.685624000	-4.406249000
6	7.255723000	0.403063000	-2.288091000
1	7.241181000	1.416216000	-1.859908000
1	6.856961000	-0.252683000	-1.499912000
6	8.711381000	0.042050000	-2.567757000
1	8.778694000	-0.928588000	-3.076160000
1	9.140661000	0.783605000	-3.256715000
6	9.527470000	0.007378000	-1.276816000
1	9.317812000	0.922832000	-0.707514000
1	9.167811000	-0.821486000	-0.651501000
6	11.031400000	-0.123623000	-1.493516000
1	11.270927000	-1.024531000	-2.069002000
1	11.423835000	0.738693000	-2.043626000
1	11.569068000	-0.182948000	-0.540262000
6	6.561960000	4.262317000	-1.021052000
1	6.328066000	3.662554000	-1.908653000
1	6.580319000	5.310258000	-1.342863000
6	7.925412000	3.860404000	-0.459599000
1	8.174663000	4.516003000	0.386834000
1	7.868955000	2.841149000	-0.048344000

6	9.047310000	3.921559000	-1.494556000
1	8.853739000	3.180803000	-2.284353000
1	9.035204000	4.903730000	-1.987499000
6	10.432598000	3.675379000	-0.898217000
1	10.627323000	4.424609000	-0.118738000
1	10.445451000	2.701159000	-0.392686000
6	11.545092000	3.721333000	-1.942570000
1	11.365114000	2.989147000	-2.738659000
1	11.604277000	4.709847000	-2.412096000
1	12.520499000	3.500253000	-1.497235000
6	6.719052000	1.958684000	3.058035000
1	6.452653000	2.710633000	2.306326000
1	6.895974000	2.496471000	3.997374000
6	7.981963000	1.207260000	2.633589000
1	8.124648000	0.344503000	3.300138000
1	7.841648000	0.789536000	1.625052000
6	9.252131000	2.056126000	2.661040000
1	9.145024000	2.911297000	1.980420000
1	9.382956000	2.478127000	3.667287000
6	10.496874000	1.254560000	2.283433000
1	10.579285000	0.389249000	2.954331000
1	10.366628000	0.842551000	1.274671000
6	11.786801000	2.068403000	2.340424000
1	11.741863000	2.926591000	1.660548000
1	11.964108000	2.453804000	3.351006000
1	12.651963000	1.460060000	2.056434000
6	-2.731652000	3.301235000	2.652372000
1	-2.596708000	2.273862000	3.012750000
1	-2.656910000	3.962681000	3.524020000
6	-4.101216000	3.448965000	1.991532000
1	-4.210427000	4.473656000	1.607475000
1	-4.150469000	2.787465000	1.115984000
6	-5.268685000	3.144538000	2.927455000
1	-5.178869000	2.117913000	3.310492000
1	-5.206333000	3.798821000	3.808272000
6	-6.634978000	3.326535000	2.265997000
1	-6.720196000	4.359782000	1.902452000
1	-6.703338000	2.684584000	1.376323000
6	-7.793794000	3.018051000	3.210943000
1	-7.784469000	1.965120000	3.516885000
1	-7.730107000	3.627065000	4.120114000
1	-8.759305000	3.222254000	2.739188000
6	-2.936948000	3.759384000	-2.164550000
1	-2.704724000	3.857609000	-1.095970000
1	-2.962601000	4.773967000	-2.579976000
6	-4.291362000	3.077912000	-2.349345000
1	-4.483198000	2.934037000	-3.422203000
1	-4.252306000	2.068131000	-1.917931000
6	-5.458997000	3.844878000	-1.734390000
1	-5.295828000	3.969707000	-0.654952000
1	-5.490667000	4.857611000	-2.160011000
6	-6.801943000	3.155954000	-1.969965000
1	-6.928451000	2.965633000	-3.044266000
1	-6.788492000	2.168001000	-1.489656000
6	-7.988428000	3.959338000	-1.447937000
1	-7.926129000	4.092664000	-0.361885000
1	-8.020286000	4.954437000	-1.906269000
1	-8.934734000	3.457175000	-1.671846000
6	-3.104310000	-0.694725000	-3.613316000
1	-2.986559000	0.264261000	-3.094950000
1	-3.055183000	-0.489053000	-4.689463000
6	-4.455386000	-1.318986000	-3.259894000

1	-4.610997000	-2.210684000	-3.883168000
1	-4.430137000	-1.671674000	-2.218640000
6	-5.636242000	-0.362593000	-3.431332000
1	-5.621841000	0.377644000	-2.620003000
1	-5.520528000	0.208794000	-4.363242000
6	-6.990022000	-1.073858000	-3.450165000
1	-7.043479000	-1.718891000	-4.336734000
1	-7.064408000	-1.748482000	-2.585682000
6	-8.170138000	-0.105389000	-3.444494000
1	-8.175876000	0.501492000	-2.531278000
1	-8.119784000	0.583841000	-4.295282000
1	-9.125142000	-0.638471000	-3.501273000
6	-3.000797000	-4.091938000	-0.104431000
1	-3.014343000	-3.274527000	-0.837119000
1	-2.790751000	-5.013663000	-0.659982000
6	-4.362624000	-4.207858000	0.575080000
1	-4.363254000	-5.082412000	1.240201000
1	-4.539450000	-3.336323000	1.220970000
6	-5.506567000	-4.328476000	-0.429465000
1	-5.558180000	-3.409671000	-1.030796000
1	-5.285900000	-5.141628000	-1.135270000
6	-6.862942000	-4.583296000	0.223461000
1	-6.822416000	-5.521291000	0.792405000
1	-7.063598000	-3.793377000	0.960143000
6	-8.002010000	-4.646450000	-0.790786000
1	-8.060698000	-3.719200000	-1.373624000
1	-7.852420000	-5.470050000	-1.498031000
1	-8.969731000	-4.794483000	-0.300511000
6	-2.704552000	-1.366301000	4.116620000
1	-2.642625000	-2.043639000	3.254468000
1	-2.588806000	-1.984632000	5.014529000
6	-4.055706000	-0.653825000	4.128193000
1	-4.158351000	-0.077103000	5.057841000
1	-4.076844000	0.080171000	3.310908000
6	-5.246238000	-1.597544000	3.977779000
1	-5.151030000	-2.147377000	3.029937000
1	-5.222225000	-2.357476000	4.770978000
6	-6.589725000	-0.870055000	3.999587000
1	-6.727279000	-0.384692000	4.974903000
1	-6.560200000	-0.057734000	3.262049000
6	-7.775006000	-1.784819000	3.701915000
1	-7.634004000	-2.305721000	2.747400000
1	-7.889114000	-2.549079000	4.478796000
1	-8.713224000	-1.225494000	3.631182000
6	-5.174039000	0.281933000	0.574574000
1	-5.363675000	0.721977000	1.562775000
6	-3.802783000	-0.332192000	0.584605000
1	-3.712170000	-1.265173000	1.143242000
1	-5.211922000	1.112992000	-0.140822000
6	-2.712274000	0.179422000	0.016078000
1	-2.747890000	1.113190000	-0.541279000
1	-1.741877000	-0.303684000	0.093162000
6	-8.775970000	-1.166973000	0.146630000
1	-8.848778000	-1.266018000	-0.947699000
1	-8.483686000	-2.151129000	0.532761000
6	-7.693985000	-0.148983000	0.495766000
1	-7.834031000	0.766845000	-0.094493000
1	-7.825245000	0.136831000	1.545694000
6	-6.291870000	-0.719805000	0.282164000
1	-6.166179000	-1.592921000	0.937001000
1	-6.187181000	-1.091738000	-0.743741000
8	-10.019856000	-0.845989000	0.723757000

14	-11.333389000	-0.199870000	-0.094170000
6	-11.743583000	-1.267417000	-1.613306000
1	-11.072817000	-0.910584000	-2.410432000
6	-10.833052000	1.545809000	-0.639692000
1	-9.843876000	1.404811000	-1.105841000
6	-12.664528000	-0.182239000	1.248126000
1	-12.154037000	0.288153000	2.102221000
6	-10.636767000	2.444336000	0.589447000
1	-11.586199000	2.630376000	1.105442000
1	-9.953369000	1.981233000	1.309475000
1	-10.221774000	3.420462000	0.310561000
6	-11.733675000	2.205727000	-1.691262000
1	-11.359559000	3.204130000	-1.954085000
1	-11.775771000	1.617925000	-2.614863000
1	-12.760750000	2.332579000	-1.329319000
6	-13.907357000	0.662349000	0.930696000
1	-13.655374000	1.705459000	0.712073000
1	-14.459462000	0.268410000	0.070385000
1	-14.597633000	0.665324000	1.784024000
6	-13.061909000	-1.601748000	1.681166000
1	-13.707599000	-1.572260000	2.568245000
1	-13.622575000	-2.116171000	0.891109000
1	-12.184494000	-2.210408000	1.922882000
6	-13.186054000	-1.068322000	-2.108379000
1	-13.439763000	-0.014468000	-2.264980000
1	-13.344566000	-1.591712000	-3.059763000
1	-13.906064000	-1.478580000	-1.390822000
6	-11.462811000	-2.763218000	-1.402326000
1	-12.096600000	-3.178816000	-0.611011000
1	-11.670424000	-3.328950000	-2.319872000
1	-10.423739000	-2.956611000	-1.121588000

E1c'

E_1 = -5417.01945478 A.U.

At No.	X	Y	Z
6	1.093092000	-3.972100000	0.976127000
6	2.292773000	-4.136485000	0.278208000
6	-0.035412000	-3.553211000	0.273102000
6	2.336491000	-3.902391000	-1.093952000
6	0.015444000	-3.297111000	-1.094579000
6	1.211359000	-3.483032000	-1.795727000
1	-0.948812000	-3.404404000	0.837786000
1	3.257875000	-4.011593000	-1.653114000
6	1.028932000	-4.145718000	2.478874000
1	0.037755000	-4.505586000	2.763652000
1	1.761445000	-4.892159000	2.796790000
6	1.318485000	-2.829184000	3.168925000
6	2.634527000	-2.430299000	3.371500000
6	0.294297000	-1.950929000	3.528338000
6	2.945544000	-1.187239000	3.915789000
6	0.599856000	-0.719173000	4.103540000
6	1.920913000	-0.319488000	4.306182000
1	3.410578000	-3.114882000	3.053637000
1	-0.187981000	-0.028336000	4.383834000
6	2.239129000	1.026991000	4.922065000
1	3.131234000	0.933476000	5.547768000
1	1.411239000	1.319990000	5.574478000
6	2.474378000	2.119928000	3.900296000
6	3.764560000	2.441518000	3.483271000
6	1.401008000	2.833723000	3.355731000
6	3.994449000	3.454699000	2.554235000
6	1.634981000	3.864947000	2.450539000

6	2.924148000	4.185467000	2.036743000
1	4.585425000	1.866526000	3.897043000
1	0.819697000	4.426374000	2.011289000
6	3.137330000	5.225667000	0.958480000
1	4.127877000	5.674423000	1.061050000
1	2.394513000	6.020321000	1.061815000
6	3.010217000	4.574882000	-0.402359000
6	1.774089000	4.497629000	-1.048628000
6	4.109104000	3.948010000	-0.985425000
6	1.666381000	3.831307000	-2.266578000
6	3.996290000	3.258760000	-2.190080000
6	2.765337000	3.209082000	-2.852336000
1	5.049485000	3.989199000	-0.448370000
1	0.715376000	3.749184000	-2.779751000
6	2.602219000	2.454208000	-4.153474000
1	1.813687000	2.928047000	-4.744449000
1	3.528933000	2.514198000	-4.730583000
6	2.255944000	0.997997000	-3.927430000
6	0.928525000	0.574572000	-3.811714000
6	3.269671000	0.052580000	-3.794638000
6	0.646097000	-0.777872000	-3.633875000
6	2.988186000	-1.293256000	-3.570247000
6	1.660025000	-1.725190000	-3.516628000
1	4.290524000	0.410639000	-3.851159000
1	-0.374388000	-1.131591000	-3.551201000
6	1.313027000	-3.178549000	-3.274320000
1	0.362314000	-3.406736000	-3.763792000
1	2.078719000	-3.818598000	-3.720761000
8	5.240039000	3.810522000	2.106300000
6	6.289763000	2.880993000	2.239802000
1	5.994900000	1.916339000	1.796694000
1	6.521659000	2.704289000	3.301074000
8	4.241603000	-0.764227000	4.090729000
6	5.186884000	-1.362653000	3.229347000
1	4.833236000	-1.273799000	2.190486000
1	5.283559000	-2.434079000	3.451649000
8	3.388207000	-4.527629000	1.004982000
6	4.644619000	-4.457010000	0.367243000
1	4.683428000	-5.154574000	-0.483395000
1	4.805361000	-3.441547000	-0.027412000
8	3.959039000	-2.248286000	-3.406530000
6	5.278986000	-1.797630000	-3.194559000
1	5.641448000	-1.248079000	-4.076544000
1	5.304519000	-1.104163000	-2.338993000
8	5.043684000	2.610730000	-2.791173000
6	6.229716000	2.430148000	-2.048614000
1	6.668854000	3.403694000	-1.783155000
1	6.011400000	1.897078000	-1.109179000
8	0.714130000	5.099091000	-0.421343000
6	-0.579581000	4.808198000	-0.900815000
1	-0.698693000	5.172880000	-1.932347000
1	-0.736492000	3.718154000	-0.914519000
8	-0.040518000	1.540834000	-3.890037000
6	-1.368542000	1.148142000	-3.618706000
1	-1.712683000	0.426114000	-4.375413000
1	-1.418667000	0.649472000	-2.638661000
8	-1.054731000	-2.861392000	-1.827993000
6	-2.276443000	-2.614589000	-1.168343000
1	-2.644261000	-3.535111000	-0.688661000
1	-2.141125000	-1.858772000	-0.378838000
8	-0.984794000	-2.385291000	3.299078000
6	-2.005179000	-1.426038000	3.123125000

1	-2.217339000	-0.898612000	4.065278000
1	-1.688462000	-0.675412000	2.382361000
8	0.146377000	2.466178000	3.761788000
6	-0.944510000	3.105696000	3.138364000
1	-0.938690000	4.181111000	3.376051000
1	-0.865616000	3.007994000	2.044874000
6	7.213635000	1.650087000	-2.903566000
1	6.742166000	0.718917000	-3.240004000
1	7.431403000	2.237572000	-3.803867000
6	8.509263000	1.337524000	-2.153202000
1	8.859600000	2.240419000	-1.632215000
1	8.308684000	0.591679000	-1.370390000
6	9.626012000	0.833940000	-3.067724000
1	9.286541000	-0.061545000	-3.606298000
1	9.828214000	1.593388000	-3.836066000
6	10.919803000	0.516078000	-2.320590000
1	11.231591000	1.402407000	-1.753149000
1	10.725719000	-0.271324000	-1.580732000
6	12.057698000	0.082129000	-3.241263000
1	11.785037000	-0.809556000	-3.817344000
1	12.307404000	0.873967000	-3.956754000
1	12.962159000	-0.152405000	-2.670553000
6	7.512088000	3.451122000	1.542932000
1	7.263520000	3.667083000	0.496395000
1	7.764901000	4.409794000	2.011925000
6	8.703019000	2.499311000	1.609917000
1	8.863547000	2.188685000	2.652355000
1	8.470413000	1.583046000	1.048628000
6	9.996302000	3.106062000	1.070244000
1	9.841549000	3.441739000	0.034650000
1	10.244845000	4.007149000	1.648078000
6	11.168621000	2.128515000	1.114709000
1	11.313594000	1.785388000	2.148437000
1	10.909443000	1.237303000	0.530254000
6	12.470641000	2.723230000	0.584659000
1	12.355196000	3.062341000	-0.451755000
1	12.781495000	3.588165000	1.181628000
1	13.282254000	1.988662000	0.607106000
6	6.561858000	-0.732139000	3.373694000
1	6.474016000	0.355946000	3.450243000
1	7.035585000	-1.080882000	4.299041000
6	7.400400000	-1.120626000	2.150382000
1	7.118973000	-2.135310000	1.832135000
1	7.128391000	-0.464886000	1.309918000
6	8.913268000	-1.102342000	2.346508000
1	9.239576000	-0.135084000	2.750159000
1	9.188875000	-1.859563000	3.094542000
6	9.641444000	-1.387460000	1.034291000
1	9.186388000	-2.271949000	0.568663000
1	9.458072000	-0.556794000	0.338838000
6	11.142341000	-1.609174000	1.189042000
1	11.622853000	-0.745096000	1.660828000
1	11.346131000	-2.486812000	1.812252000
1	11.624590000	-1.771170000	0.218228000
6	5.728683000	-4.810320000	1.367233000
1	5.700290000	-4.093805000	2.196686000
1	5.516753000	-5.797505000	1.794484000
6	7.111320000	-4.804662000	0.715743000
1	7.147221000	-5.580129000	-0.062535000
1	7.271341000	-3.846794000	0.197985000
6	8.248127000	-5.031969000	1.710485000
1	8.281966000	-4.192653000	2.420704000

1	8.035409000	-5.929394000	2.308134000
6	9.613809000	-5.185101000	1.042169000
1	9.579496000	-6.031438000	0.342599000
1	9.825011000	-4.296461000	0.433387000
6	10.745010000	-5.395227000	2.045525000
1	10.790434000	-4.567535000	2.763220000
1	10.598049000	-6.318599000	2.617271000
1	11.716498000	-5.459274000	1.544801000
6	6.186002000	-2.990025000	-2.946388000
1	5.797184000	-3.582953000	-2.110212000
1	6.168793000	-3.637900000	-3.831180000
6	7.616277000	-2.533602000	-2.654331000
1	7.920800000	-1.805194000	-3.419510000
1	7.643535000	-1.993923000	-1.695537000
6	8.640148000	-3.667555000	-2.629659000
1	8.374725000	-4.393961000	-1.849892000
1	8.599904000	-4.211689000	-3.583617000
6	10.063243000	-3.162884000	-2.395601000
1	10.309123000	-2.422132000	-3.167932000
1	10.101347000	-2.625442000	-1.439618000
6	11.111817000	-4.271959000	-2.400593000
1	10.904878000	-5.013569000	-1.620910000
1	11.125019000	-4.796207000	-3.363006000
1	12.114838000	-3.869701000	-2.223580000
6	-3.269536000	-2.132031000	-2.211751000
1	-2.928549000	-1.170247000	-2.615694000
1	-3.262582000	-2.844165000	-3.045701000
6	-4.689261000	-1.996840000	-1.664697000
1	-5.030142000	-2.974864000	-1.295843000
1	-4.695905000	-1.323443000	-0.794332000
6	-5.675514000	-1.481760000	-2.713021000
1	-5.413146000	-0.449039000	-2.983151000
1	-5.569368000	-2.072351000	-3.633737000
6	-7.130782000	-1.529234000	-2.252000000
1	-7.417410000	-2.570809000	-2.053347000
1	-7.220830000	-1.006331000	-1.290755000
6	-8.092939000	-0.917551000	-3.266930000
1	-7.811383000	0.114867000	-3.505615000
1	-8.088921000	-1.486154000	-4.203815000
1	-9.118964000	-0.902811000	-2.887153000
6	-3.240977000	-2.175719000	2.657397000
1	-2.993098000	-2.728608000	1.742428000
1	-3.491796000	-2.927368000	3.415844000
6	-4.448802000	-1.277731000	2.398434000
1	-4.674333000	-0.684069000	3.295366000
1	-4.214235000	-0.555208000	1.601997000
6	-5.679179000	-2.094048000	2.004179000
1	-5.431517000	-2.716708000	1.133033000
1	-5.921014000	-2.792692000	2.817360000
6	-6.912462000	-1.255413000	1.677916000
1	-7.175213000	-0.633832000	2.545157000
1	-6.672785000	-0.554091000	0.867767000
6	-8.105361000	-2.122839000	1.282612000
1	-7.858529000	-2.762668000	0.427408000
1	-8.410254000	-2.774188000	2.109854000
1	-8.972763000	-1.517758000	1.000436000
6	-2.236307000	2.475584000	3.622212000
1	-2.284875000	1.436064000	3.276344000
1	-2.235051000	2.450395000	4.718584000
6	-3.446944000	3.259412000	3.118674000
1	-3.416856000	4.274363000	3.540250000
1	-3.376966000	3.384023000	2.027034000

6	-4.785417000	2.609043000	3.460948000
1	-4.893402000	1.675746000	2.891144000
1	-4.798893000	2.327826000	4.523465000
6	-5.973636000	3.520289000	3.161697000
1	-5.907779000	4.420801000	3.787100000
1	-5.898174000	3.867568000	2.123029000
6	-7.318375000	2.833126000	3.378841000
1	-7.398257000	1.939910000	2.749464000
1	-7.436785000	2.521491000	4.422967000
1	-8.154305000	3.495734000	3.129120000
6	-1.597756000	5.487452000	-0.002897000
1	-1.491787000	5.112916000	1.023264000
1	-1.379563000	6.561723000	0.026659000
6	-3.020461000	5.254318000	-0.507537000
1	-3.087838000	5.573990000	-1.557522000
1	-3.238166000	4.175371000	-0.507642000
6	-4.092979000	5.982172000	0.301515000
1	-4.104296000	5.595309000	1.330257000
1	-3.836488000	7.048016000	0.376745000
6	-5.481425000	5.832242000	-0.318634000
1	-5.477659000	6.281053000	-1.321147000
1	-5.688037000	4.766079000	-0.469286000
6	-6.595057000	6.456886000	0.518092000
1	-6.642669000	6.002443000	1.514829000
1	-6.432439000	7.532238000	0.653541000
1	-7.572226000	6.324075000	0.041073000
6	-2.267257000	2.371851000	-3.633312000
1	-1.985773000	3.044373000	-2.813272000
1	-2.104075000	2.921300000	-4.568241000
6	-3.738944000	1.973721000	-3.513160000
1	-4.003544000	1.329530000	-4.364214000
1	-3.882635000	1.360352000	-2.611118000
6	-4.700106000	3.161129000	-3.463545000
1	-4.578656000	3.690351000	-2.509046000
1	-4.437700000	3.880243000	-4.252511000
6	-6.161601000	2.745301000	-3.628888000
1	-6.298137000	2.294461000	-4.621225000
1	-6.394746000	1.961418000	-2.897664000
6	-7.133707000	3.907898000	-3.449396000
1	-7.066086000	4.314914000	-2.433746000
1	-6.910831000	4.721080000	-4.150011000
1	-8.168998000	3.593691000	-3.620345000
6	-8.242675000	1.819478000	-0.696465000
1	-8.713390000	2.510827000	-1.408656000
6	-7.033376000	2.463284000	-0.090825000
1	-7.216871000	3.389083000	0.454808000
1	-7.939313000	0.938238000	-1.269841000
6	-5.795110000	1.975444000	-0.145673000
1	-5.570970000	1.053168000	-0.680003000
1	-4.958992000	2.479376000	0.332972000
6	-11.378698000	0.106753000	0.859173000
1	-10.802421000	-0.386375000	1.653923000
1	-11.867009000	0.975004000	1.318694000
6	-10.414570000	0.580724000	-0.223368000
1	-9.991151000	-0.291246000	-0.737738000
1	-10.962723000	1.157496000	-0.978563000
6	-9.271826000	1.407249000	0.362852000
1	-9.665518000	2.300800000	0.866172000
1	-8.763003000	0.818735000	1.137019000
8	-12.399803000	-0.737528000	0.386410000
14	-12.373710000	-2.361819000	-0.022176000
6	-11.520907000	-3.460533000	1.290293000

1	-10.476002000	-3.560254000	0.960068000
6	-11.410047000	-2.593846000	-1.647985000
1	-10.363880000	-2.369515000	-1.379368000
6	-14.211596000	-2.808062000	-0.140866000
1	-14.264757000	-3.896468000	-0.291791000
6	-11.817006000	-1.625641000	-2.767909000
1	-11.121863000	-1.697451000	-3.614225000
1	-12.813716000	-1.858170000	-3.154397000
1	-11.832584000	-0.585190000	-2.429394000
6	-11.437490000	-4.043492000	-2.156485000
1	-10.985564000	-4.739339000	-1.441908000
1	-12.463067000	-4.381333000	-2.352860000
1	-10.880014000	-4.134821000	-3.097658000
6	-14.942074000	-2.136576000	-1.313173000
1	-14.601075000	-2.519556000	-2.279785000
1	-16.022006000	-2.324318000	-1.251739000
1	-14.794727000	-1.050615000	-1.308236000
6	-14.926722000	-2.480519000	1.181077000
1	-15.987994000	-2.754856000	1.125327000
1	-14.494500000	-3.016941000	2.032730000
1	-14.868000000	-1.408762000	1.399290000
6	-12.125830000	-4.877868000	1.308273000
1	-12.181424000	-5.333202000	0.314080000
1	-11.525296000	-5.542655000	1.941924000
1	-13.141734000	-4.868578000	1.719531000
6	-11.502071000	-2.901203000	2.721435000
1	-11.080033000	-3.640573000	3.415010000
1	-10.895534000	-1.995728000	2.808466000
1	-12.508458000	-2.656521000	3.078161000

I

E_1 = -6018.08787457 A.U.

Zero-point correction= 0.235889 (Hartree/Particle)

Thermal correction to Energy= 0.256202

Thermal correction to Enthalpy= 0.257146

Thermal correction to Gibbs Free Energy= 0.182388

E_2 = -6023.22961460 A.U.

At No.	X	Y	Z
7	-1.481402000	1.371325000	-0.145242000
6	-1.456043000	0.219026000	-0.941272000
6	-1.641108000	1.106421000	1.207202000
6	-2.021109000	-0.379396000	1.300402000
7	-1.588180000	-0.845777000	-0.034691000
8	-1.315056000	0.155590000	-2.128973000
8	-1.572469000	1.888421000	2.114094000
35	-2.279738000	-2.434295000	-0.719331000
35	-0.840873000	2.970893000	-0.772595000
6	4.018773000	0.709844000	0.511509000
6	3.346672000	-0.456971000	0.142089000
6	4.064964000	-1.572350000	-0.294586000
6	5.452412000	-1.516772000	-0.358348000
6	5.405156000	0.760926000	0.446164000
6	6.121769000	-0.352388000	0.011624000
1	3.534775000	-2.473142000	-0.582813000
1	3.438527000	1.563737000	0.845061000
1	6.012711000	-2.382262000	-0.698229000
1	5.928110000	1.667886000	0.733098000
1	7.205900000	-0.312113000	-0.039861000
6	1.864166000	-0.464335000	0.228049000
8	1.312868000	-1.632043000	-0.122025000
8	1.200919000	0.490024000	0.587935000

1	0.343350000	-1.512382000	-0.074615000
6	-3.538706000	-0.466197000	1.499617000
1	-3.805835000	0.051614000	2.424293000
1	-4.075781000	0.000170000	0.667831000
1	-3.851664000	-1.510839000	1.578677000
6	-1.253565000	-1.086368000	2.408303000
1	-0.178943000	-0.937640000	2.287908000
1	-1.553766000	-0.681935000	3.377651000
1	-1.479329000	-2.157447000	2.385187000

J (Imag. Freq → -364.7004 cm^{-1})
E_1 = -6017.99330168 A.U.
Zero-point correction= 0.233519 (Hartree/Particle)
Thermal correction to Energy= 0.253042
Thermal correction to Enthalpy= 0.253986
Thermal correction to Gibbs Free Energy= 0.181369
E_2 = -6023.13356165 A.U.

At No.	X	Y	Z
7	-2.636126000	-0.367547000	-0.365932000
6	-1.271583000	-0.665814000	-0.470340000
6	-2.912652000	0.996981000	-0.332540000
6	-1.559084000	1.679578000	-0.531539000
7	-0.610447000	0.576161000	-0.268980000
8	-0.772189000	-1.733965000	-0.665535000
8	-3.993083000	1.511292000	-0.238843000
35	-0.002854000	0.432388000	2.001849000
35	-3.926222000	-1.668704000	-0.384611000
6	4.473990000	-0.959447000	0.660798000
6	3.822185000	-0.180949000	-0.296923000
6	4.521592000	0.297740000	-1.405846000
6	5.870508000	-0.001463000	-1.554871000
6	5.823951000	-1.254333000	0.509202000
6	6.521937000	-0.776960000	-0.597852000
1	3.995994000	0.902855000	-2.136525000
1	3.915536000	-1.325328000	1.515245000
1	6.415261000	0.370463000	-2.417419000
1	6.332561000	-1.857691000	1.254950000
1	7.576361000	-1.009695000	-0.715443000
6	2.368805000	0.159005000	-0.154289000
8	1.848644000	0.989542000	-0.931702000
8	1.746908000	-0.480017000	0.765082000
1	0.374496000	0.703347000	-0.673942000
6	-1.372156000	2.901558000	0.360272000
1	-2.058060000	3.682514000	0.024360000
1	-1.581438000	2.681480000	1.405971000
1	-0.343946000	3.263722000	0.272344000
6	-1.427784000	2.069444000	-2.017652000
1	-0.440214000	2.505994000	-2.190178000
1	-1.545899000	1.201675000	-2.673947000
1	-2.197479000	2.804560000	-2.266908000

K
E_1 = -6018.07287018 A.U.
Zero-point correction= 0.235797 (Hartree/Particle)
Thermal correction to Energy= 0.256163
Thermal correction to Enthalpy= 0.257108
Thermal correction to Gibbs Free Energy= 0.181567
E_2 = -6023.21406099 A.U.

At No.	X	Y	Z
7	-2.638374000	-0.650933000	0.110654000

6	-1.755816000	-0.242087000	1.149327000
6	-2.091964000	-1.595527000	-0.733590000
6	-0.678256000	-1.859127000	-0.191610000
7	-0.652640000	-1.018934000	0.999452000
8	-1.990080000	0.616717000	1.966211000
8	-2.605909000	-2.105358000	-1.696939000
35	4.288327000	-1.157473000	-0.010858000
35	-4.311197000	0.065805000	-0.090940000
6	1.850385000	2.539855000	-1.071567000
6	1.577718000	1.848807000	0.114619000
6	0.510158000	2.237440000	0.927824000
6	-0.294591000	3.303615000	0.548115000
6	1.049465000	3.613630000	-1.436772000
6	-0.023766000	3.992257000	-0.631144000
1	0.295472000	1.695804000	1.841551000
1	2.675762000	2.232144000	-1.703339000
1	-1.138497000	3.580041000	1.170733000
1	1.256355000	4.151093000	-2.356677000
1	-0.654417000	4.824580000	-0.928811000
6	2.345655000	0.648500000	0.528430000
8	2.085220000	-0.065749000	1.468606000
8	3.377772000	0.413732000	-0.319026000
1	0.212274000	-0.768886000	1.467590000
6	-0.523905000	-3.340429000	0.156349000
1	0.478916000	-3.522785000	0.555610000
1	-1.258164000	-3.638560000	0.909664000
1	-0.666871000	-3.950486000	-0.739619000
6	0.349737000	-1.418908000	-1.239412000
1	0.183498000	-1.967534000	-2.170382000
1	0.264971000	-0.346612000	-1.443015000
1	1.363304000	-1.634229000	-0.885343000
